# Supplementary material for: Dissecting transcriptomic signatures of neuronal differentiation and maturation using iPSCs
Source: Nat Commun. 2020 Jan 23;11:462. doi: 10.1038/s41467-019-14266-z (PMC6978526; doi:10.1038/s41467-019-14266-z)
Supplement: Supplementary file 1 — Supplementary Information [file 41467_2019_14266_MOESM1_ESM.pdf]

Supplementary Information

Dissecting transcriptomic signatures of neuronal differentiation and maturation using iPSCs

Burke, Chenoweth et al.

Table of Contents

Page 1: Supplementary Figures

Page 16: Supplementary Tables

Page 20: Supplementary References

Supplementary Figures

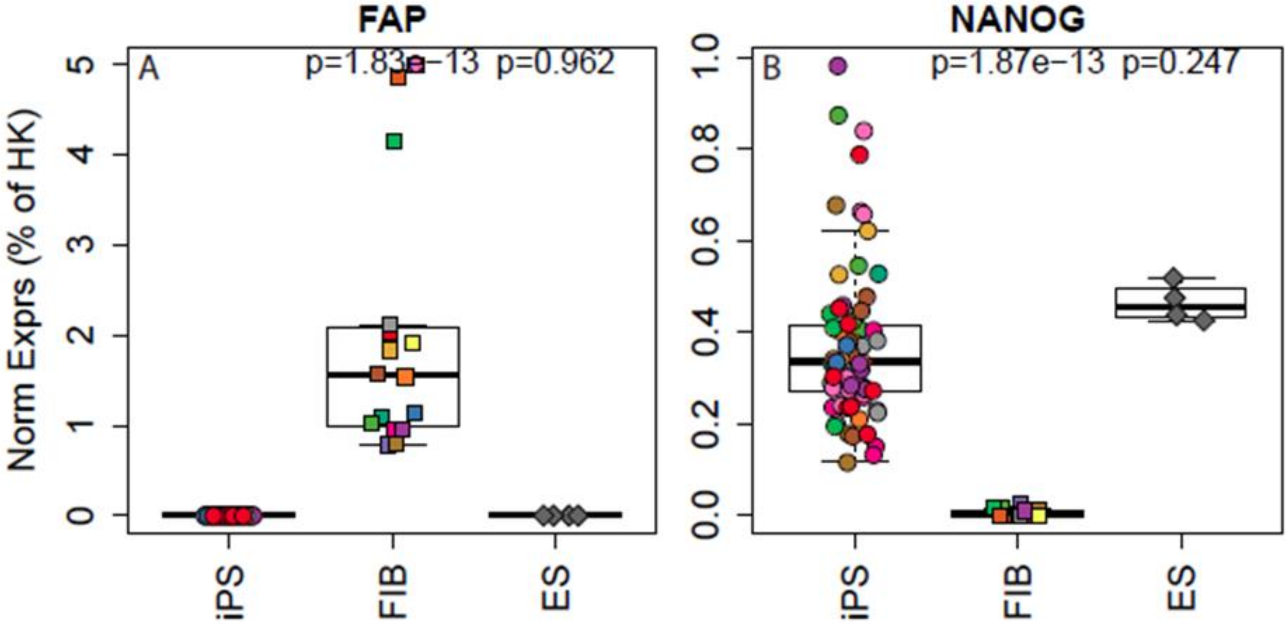

Supplementary Figure 1: Fluidigm-based expression profiling. Fluidigm-based expression profiling confirmed the loss of *FAP* (Fibroblast Activation Protein Alpha) expression across all iPSC lines and the gain of *NANOG* expression.

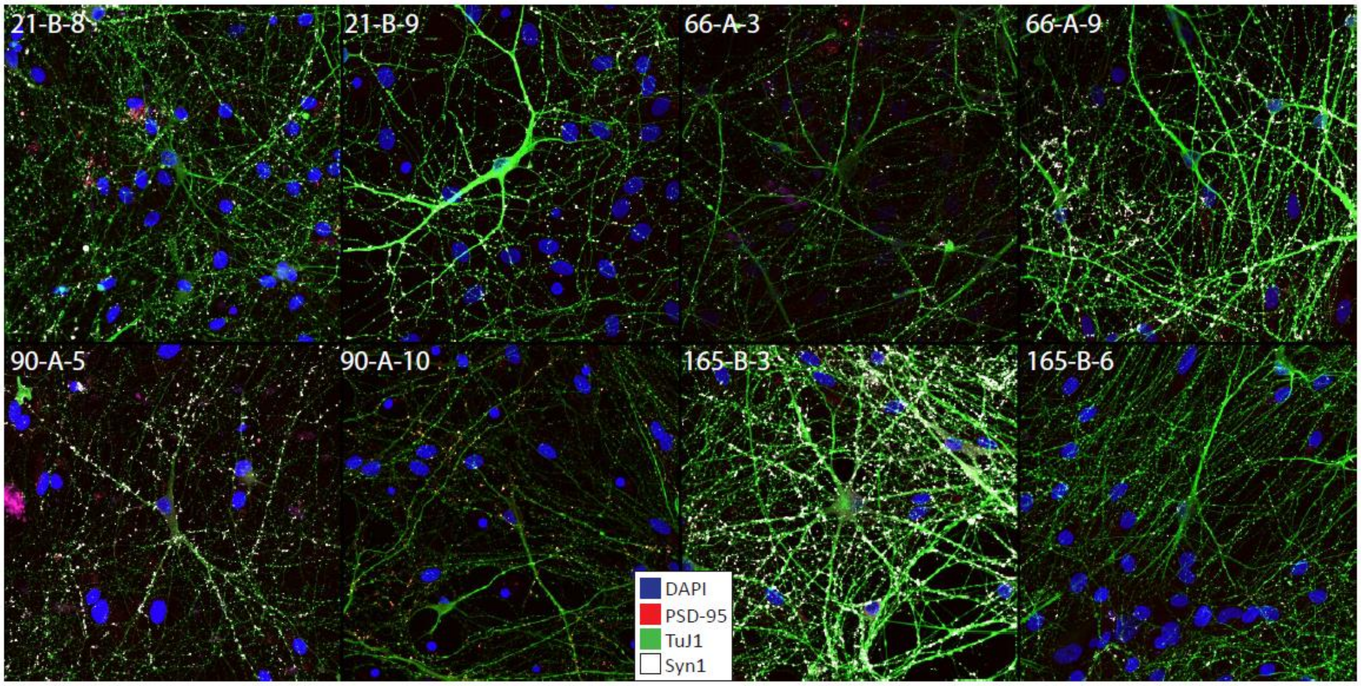

Supplementary Figure 2: Immunocytochemical labeling of neurons. A subset of lines were stained at eight weeks of differentiation to label representative neurons. Images were randomly chosen as single examples of 10-40 fields per coverslip. Blue - DAPI; Red - PSD-95; Green - TuJ1; White - Syn1.

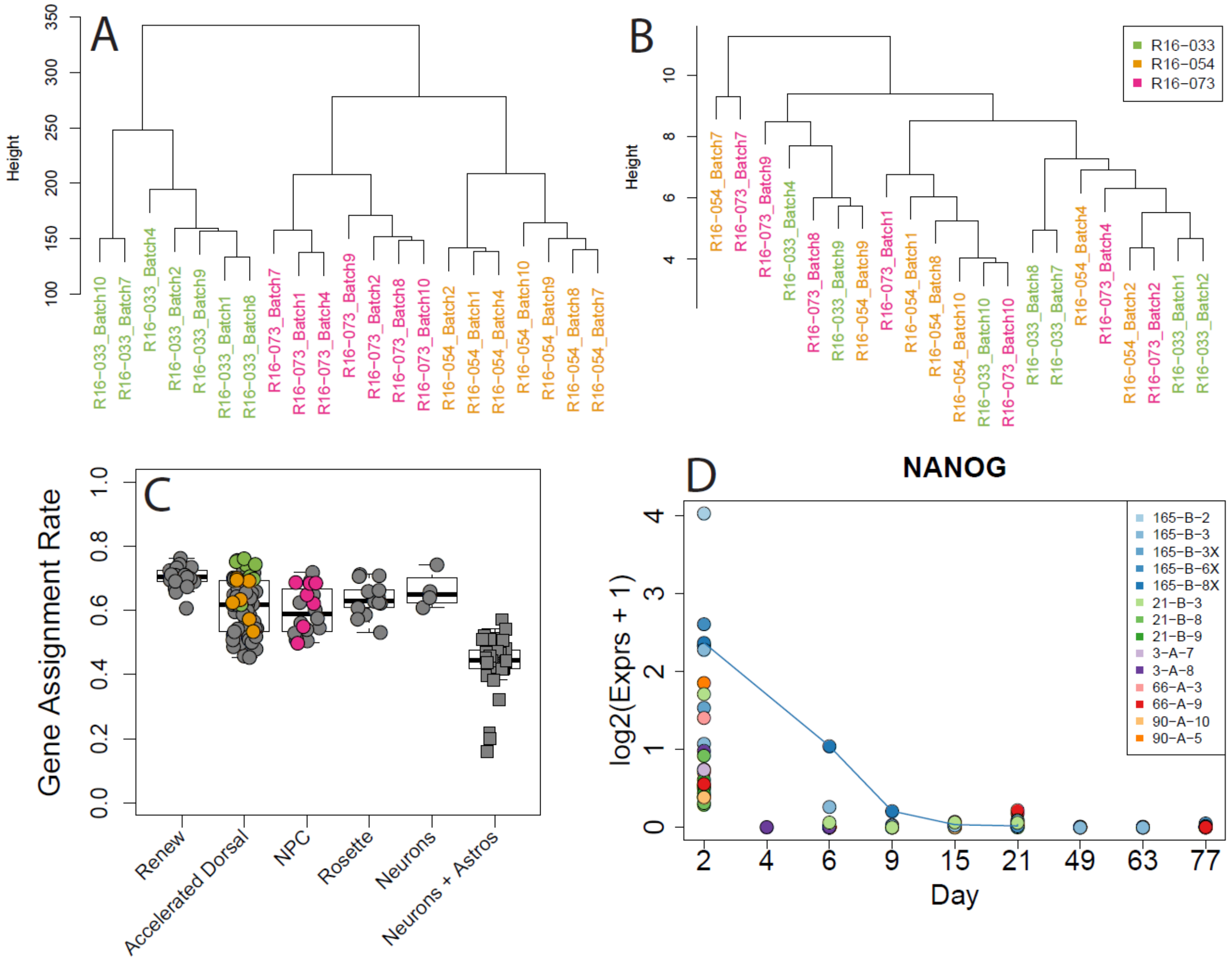

Supplementary Figure 3: RNA-seq quality control. Three RNA replicated were sequenced across seven flow cells to assess batch effect. (A) Gene expression levels of the RNA replicates across flow cells clustered by cell line, and (B) ERCC spike-ins of the replicates clustered more by flow cell (batch number), passing quality control. (C) The percent of aligned reads assigned to genes across cellular conditions, with colored replicate points having similar rates. Additionally, even the neuronal samples co-cultured with rat astrocytes had high proportions of exonic reads. (D) Slow differentiation of cell line 165-B-8X as shown by expression of *NANOG* through time, resulting in us dropping the 6 samples from this line.

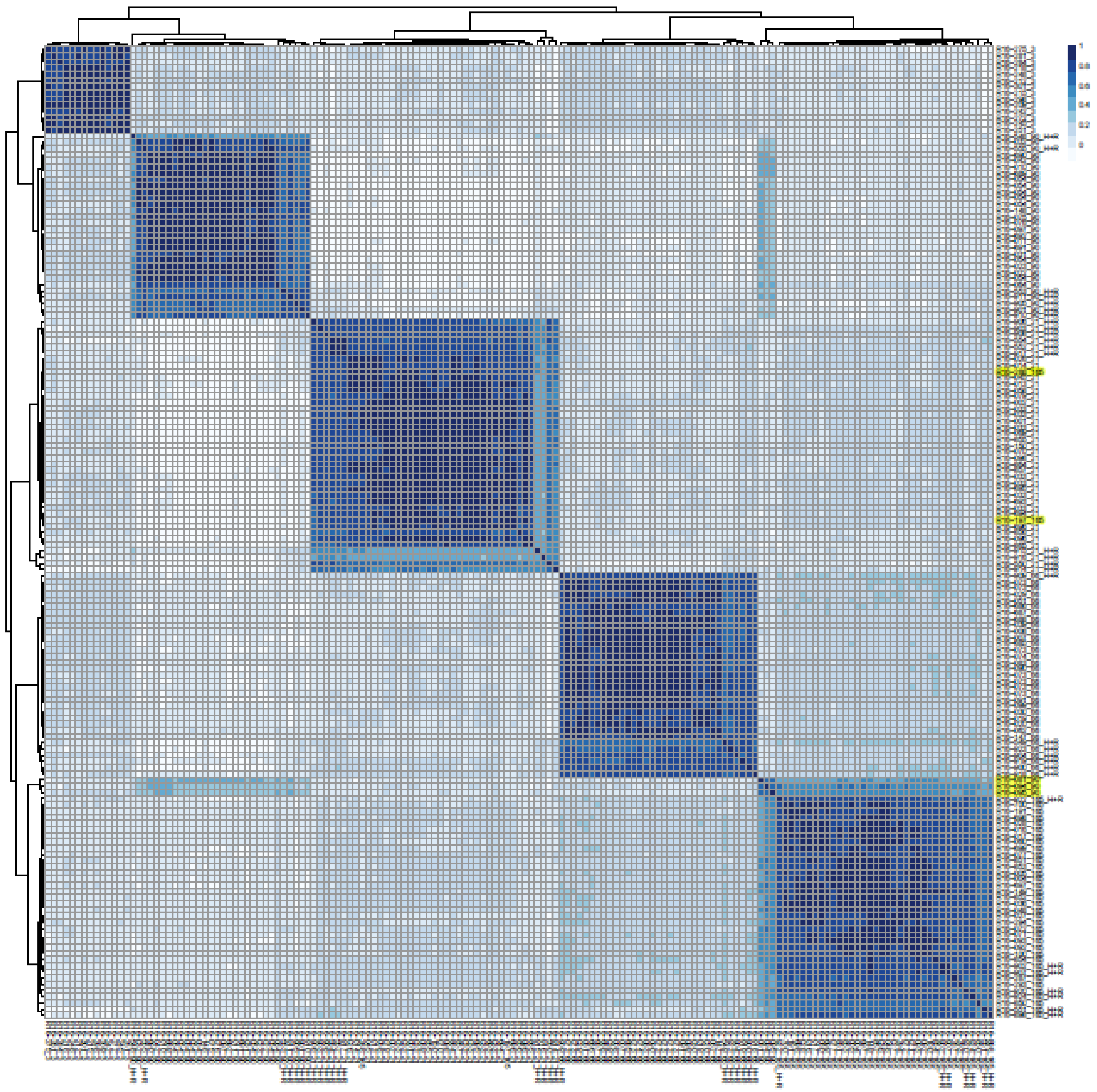

Supplementary Figure 4: Genotyping mismatches. Correlation heatmap of microarray-based genotype calls using called coding variants. Five samples (highlighted in yellow) did not highly correlate with their labeled donor identity and were dropped from all analyses.

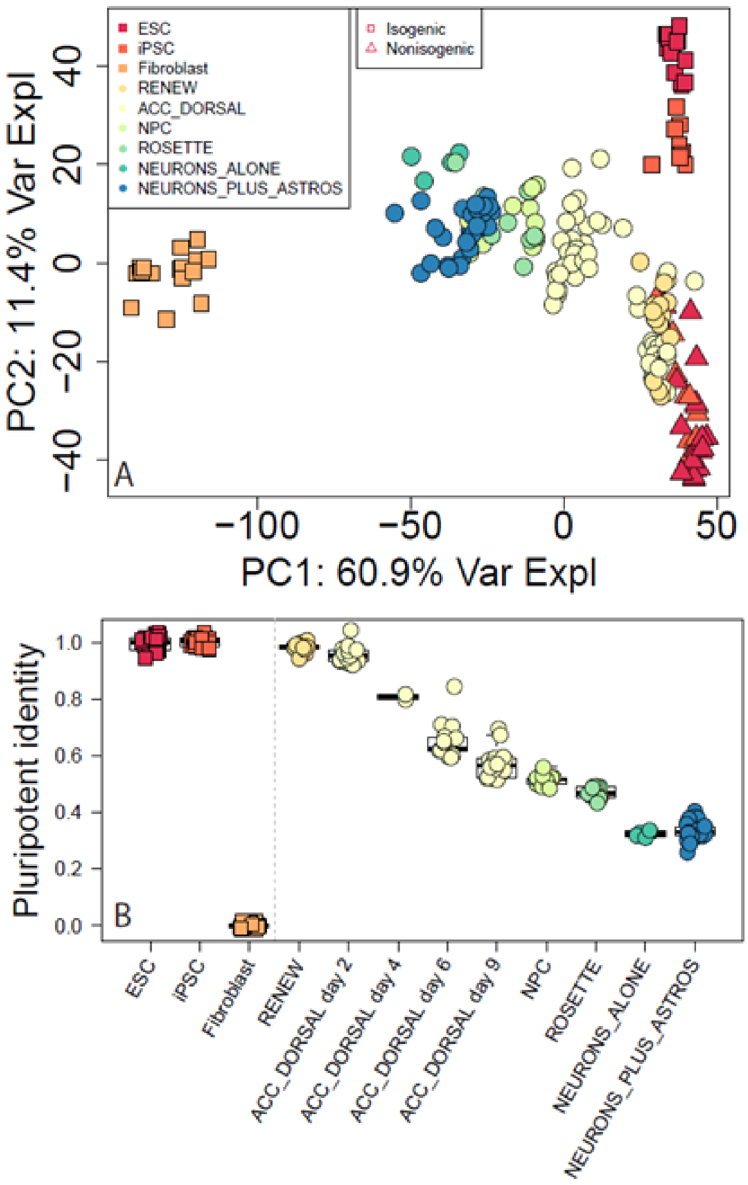

Supplementary Figure 5: Comparison to ScoreCard data [1]. (A) Our data (circles) projected into the first two PCs calculated from the ScoreCard reference data gene expression levels (square and triangle points), to assess the representativeness of our iPSC cell lines and subsequent differentiation data. (B) The pluripotent identity of ScoreCard data and our data, showing our self-renewal and early iPSC lines with a mean 98.1% pluripotency identity which significantly decreased through differentiation.

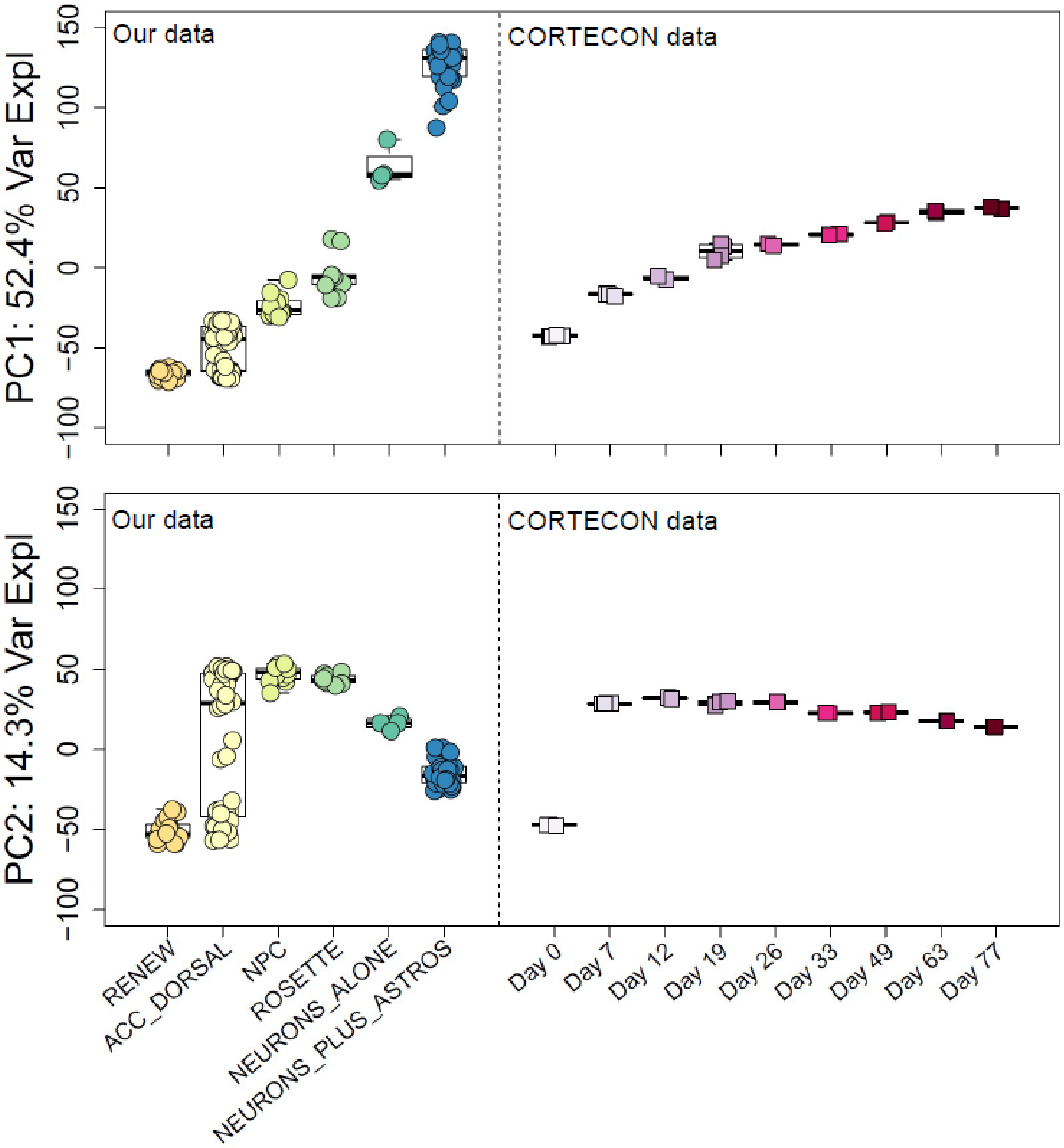

Supplementary Figure 6: PCA and comparison to CORTECON data [2]. PCA of gene expression levels showing PC1 representing corticogenesis, and PC2 separating NPC stage cells from early days as well as mature neurons. Both of these components of variability – a linear trend in PC1 across differentiation and a quadratic trend in PC2 – were significantly conserved when we projected the CORTECON data (square points) into these PCs.

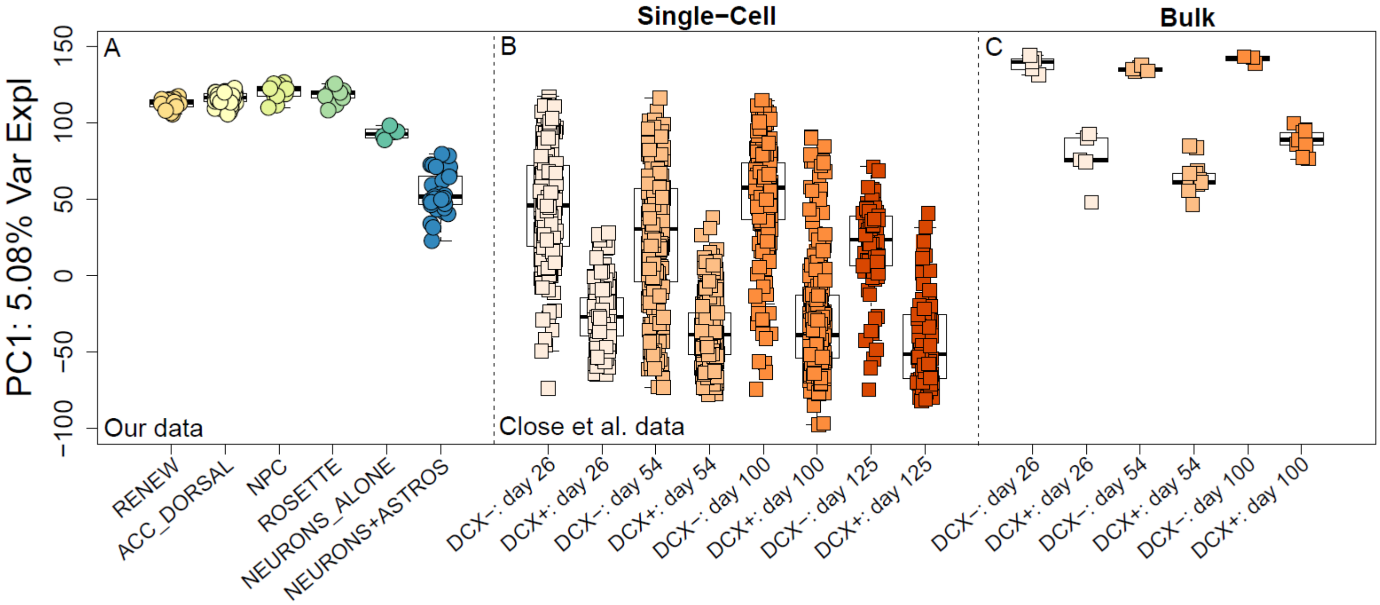

Supplementary Figure 7: PCA of Close et al [3]. PCs based on pooled cell-level data from Close et al., with our data projected in. Our samples (A) clustered with the Close et al. pooled cells (C), with our more mature neuronal lines clustering with DCX+ samples and early NPC lines clustering with DCX- samples. The single cell samples are also shown in (B).

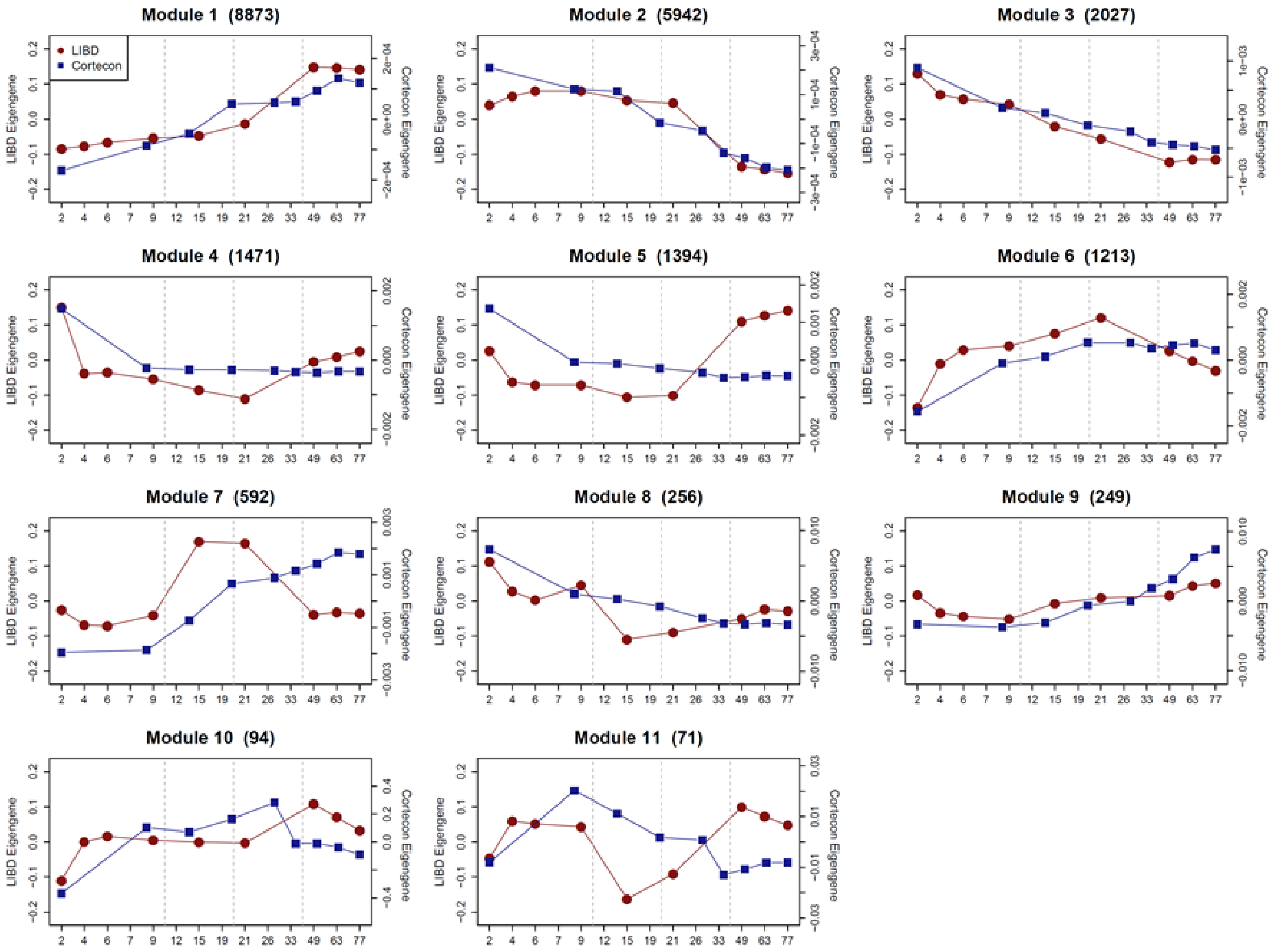

Supplementary Figure 8: Eigengenes of WGCNA modules. The eigengenes of the WGCNA modules calculated from our data, plotted with the CORTECON eigengenes calculated after separating that data into the same eleven gene sets, showing similar developmental patterns across most of the top modules.

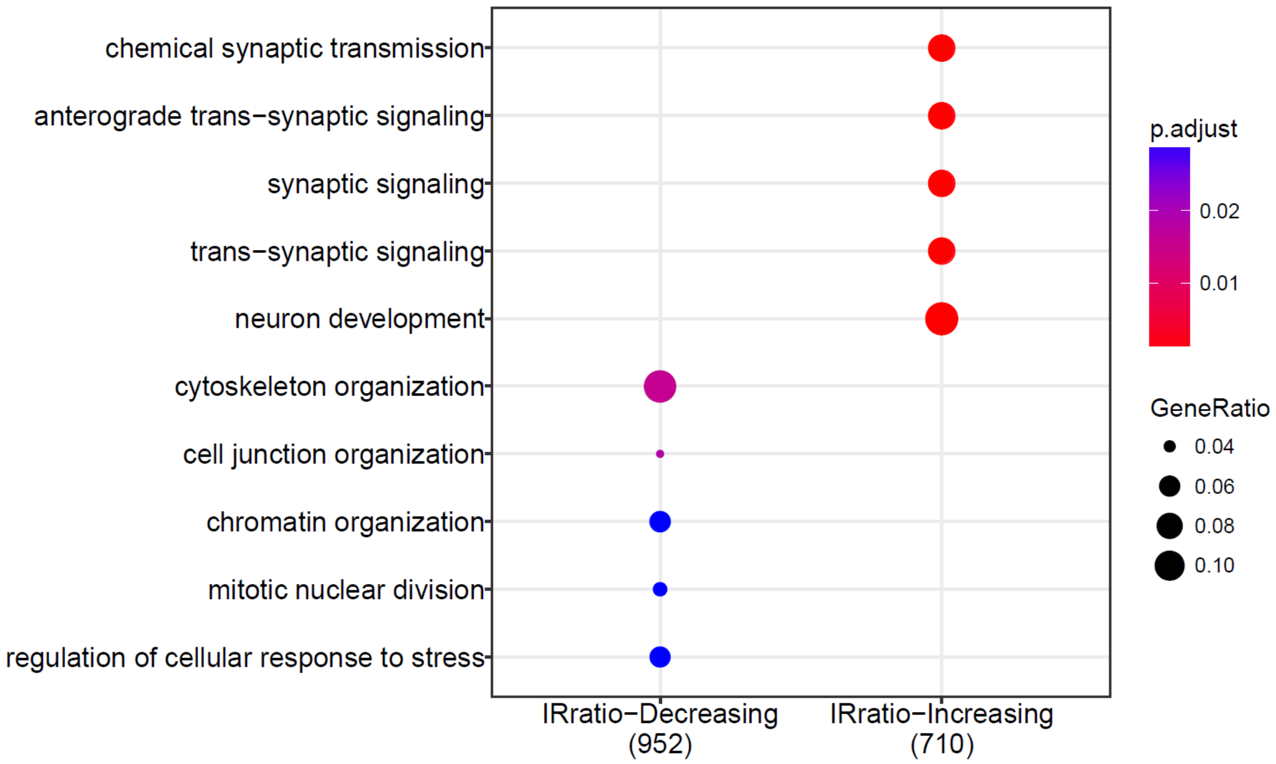

Supplementary Figure 9: GO analysis of intron retention ratios. Enrichment of the 2847 genes with significantly (FDR < 0.001%) decreasing (1518/2847) or increasing (1329/2847) intron retention ratios. As expected, increasing intron retention was enriched for neuron development terms.

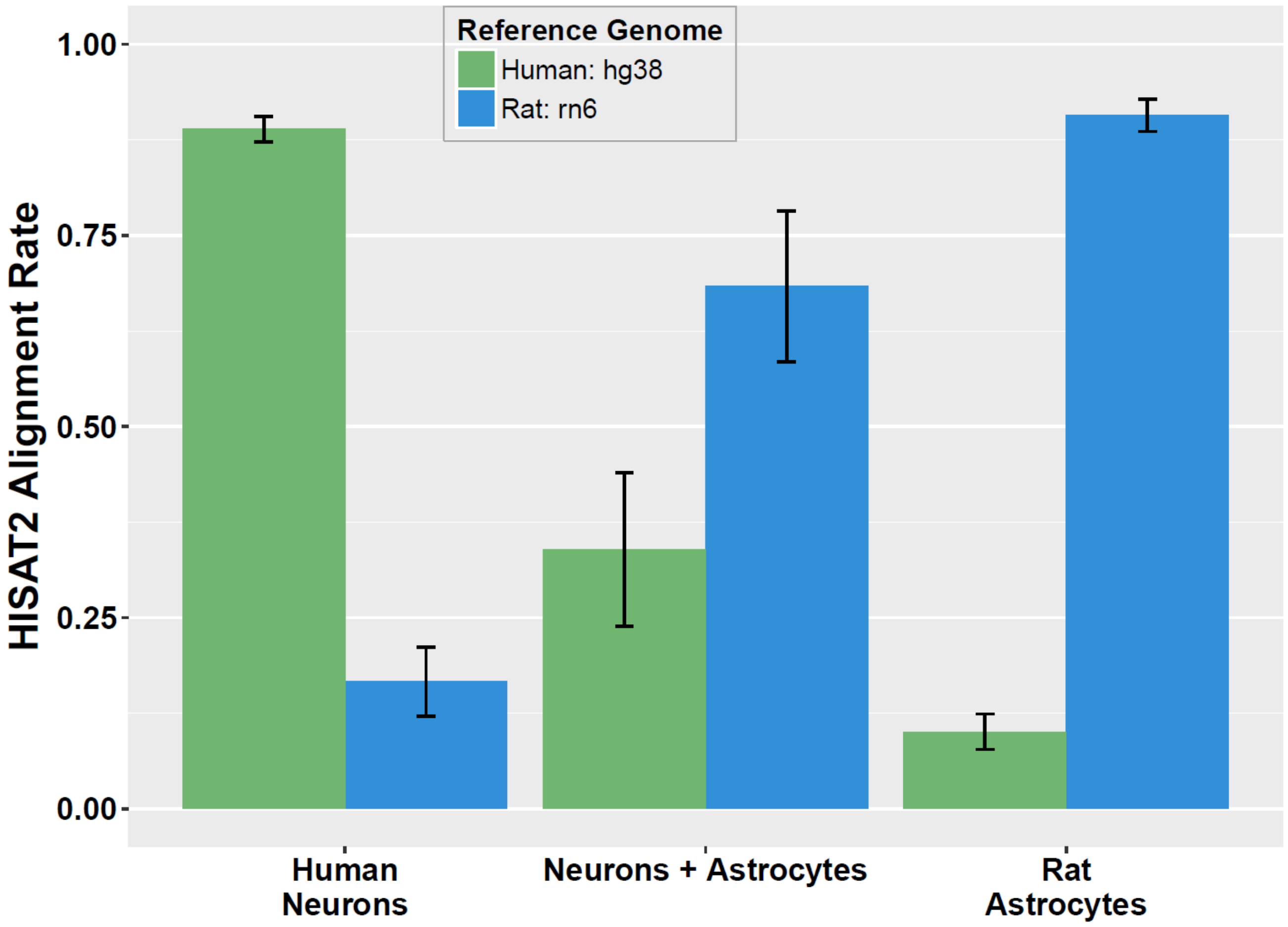

Supplementary Figure 10: Cross-species mapping in RNA-seq alignments. Alignment rates of human neurons alone, rat astrocytes alone, and co-cultured samples mapped to human and rat genomes. Human neuron samples had a low alignment rate to the rat genome (mean=16.6%) and rat astrocytes lowly aligned to the human genome (mean=10.1%). We took these two sets of aligned reads and mapped them back to human and rat, respectively, and found that in each case only 3 genes accounted for the majority of cross-mapped expression (Supplementary Table 4).

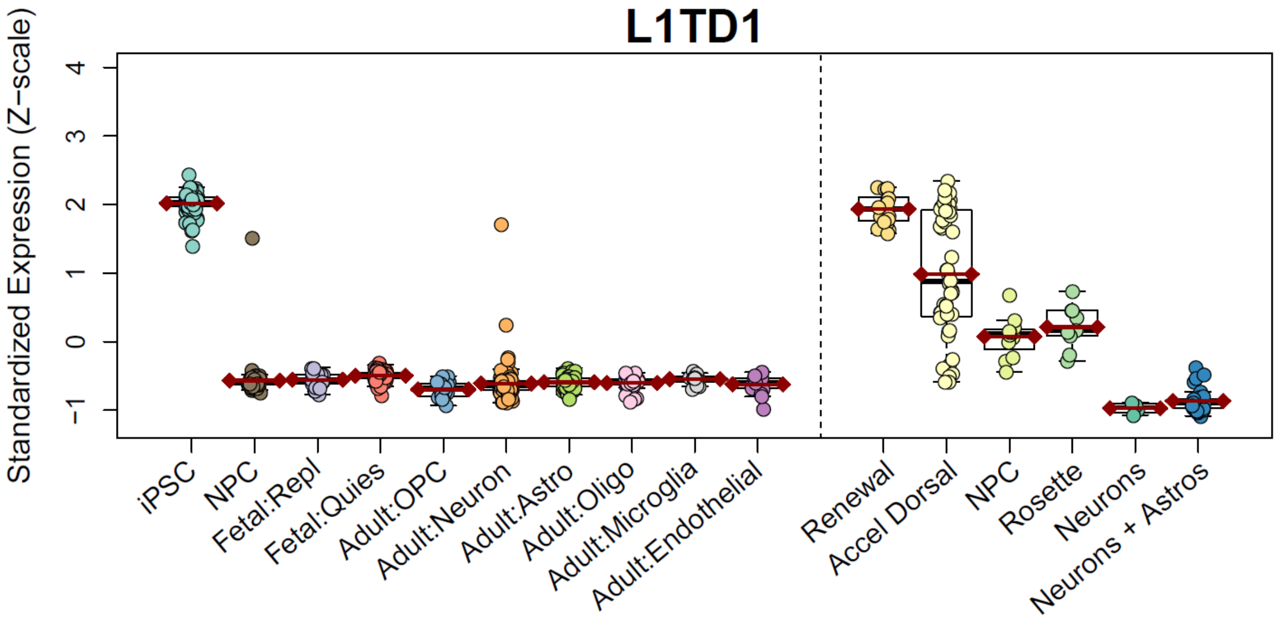

Supplementary Figure 11: Cell type deconvolution proportions of 131 signature genes. Boxplot of the standardized expression level of L1TD1, one of the 131 genes that distinguish iPSCs, NPCs, fetal replicating neurons, fetal quiescent neurons, adult neurons, and adult endothelial cells.

Note above figure of L1TD1 shows only the first plot of 131 genes. For full set of panels, see: **Supplementary Data 5**

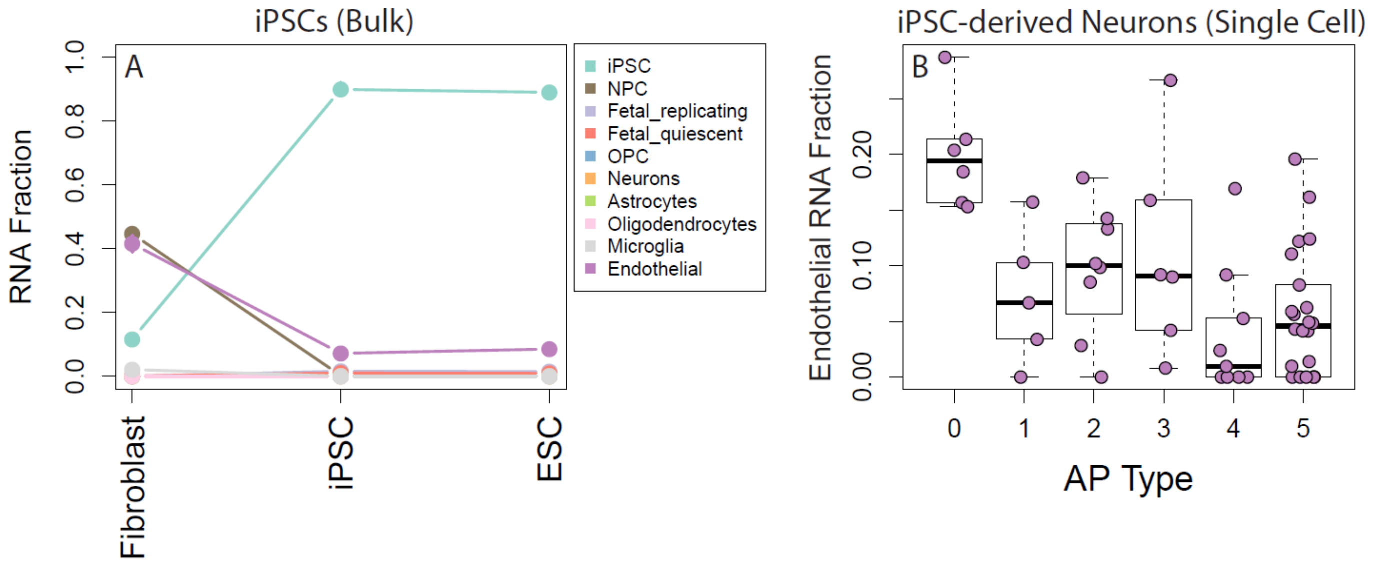

Supplementary Figure 12: Deconvolution using public datasets. (A) The ScoreCard manuscript [1] had reported a residual fibroblast-like signature which was not supported by applying this deconvolution to pure fibroblast and iPSC data. (B) A dataset of iPSC-derived neurons combined with activity state electrophysiology data suggest that the endothelial RNA fractions are associated with less mature astrocyte cells (Type 0, ~20% RNA Fraction).

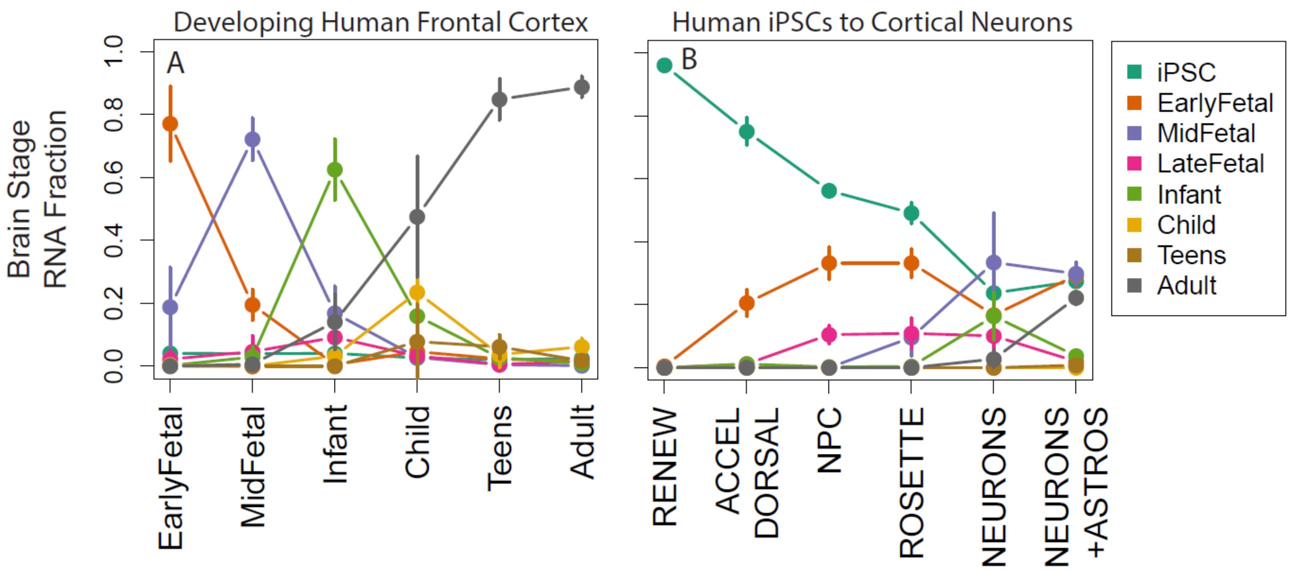

Supplementary Figure 13: Brain stage deconvolution. We designed a second deconvolution model to estimate RNA fractions from eight developmental stages. We applied the deconvolution to a large postmortem brain tissue dataset [4] (A) and our stem cell data (B). In our data, we found loss of pluripotency, rise and fall of the early-fetal signature, and then rise of both mid-fetal but also adult cortical signatures. We again saw that neurons co-cultured with astrocytes showed a higher percentage of mature neuron signature than neurons grown alone, with 22.2% of RNA analogous to adult neocortex in the co-cultured samples.

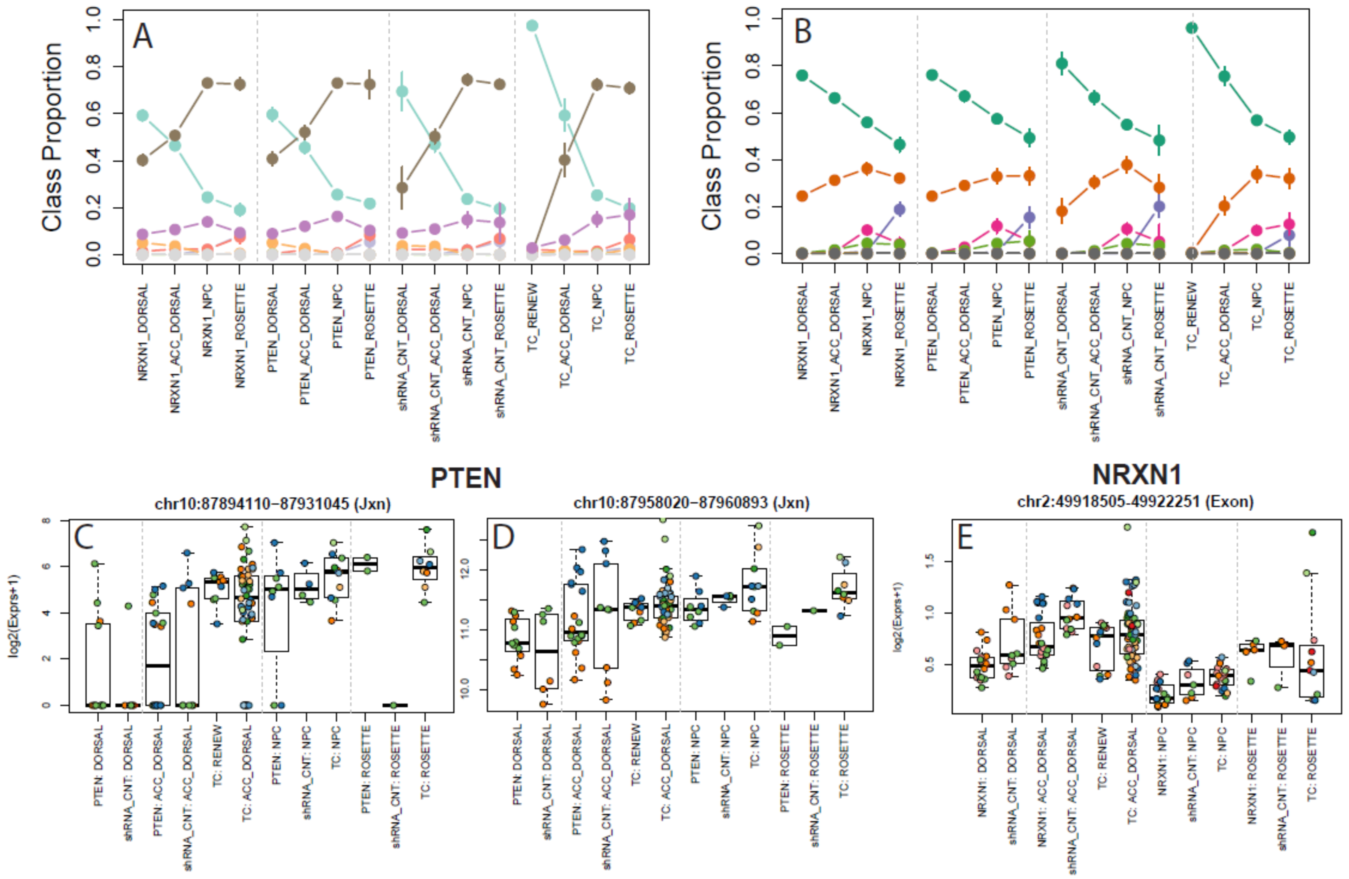

Supplementary Figure 14: shRNA experiment deconvolutions. We calculated the cell type (A) and brain stage (B) estimated fractions for knock down experiments of *NRXN1* and *PTEN* using three complementary short hairpin RNAs (shRNAs). The figure groups by experiment to compare the RNA fraction trajectories of the *NRXN1* knockdowns, *PTEN* knockdowns, shRNA control samples (shRNA_CNT), and our time-course samples (TC). The lower panels show the expected expression of the *PTEN* junctions (C-D) and *NRXN1* exon (E) targeted in the knock down experiments.

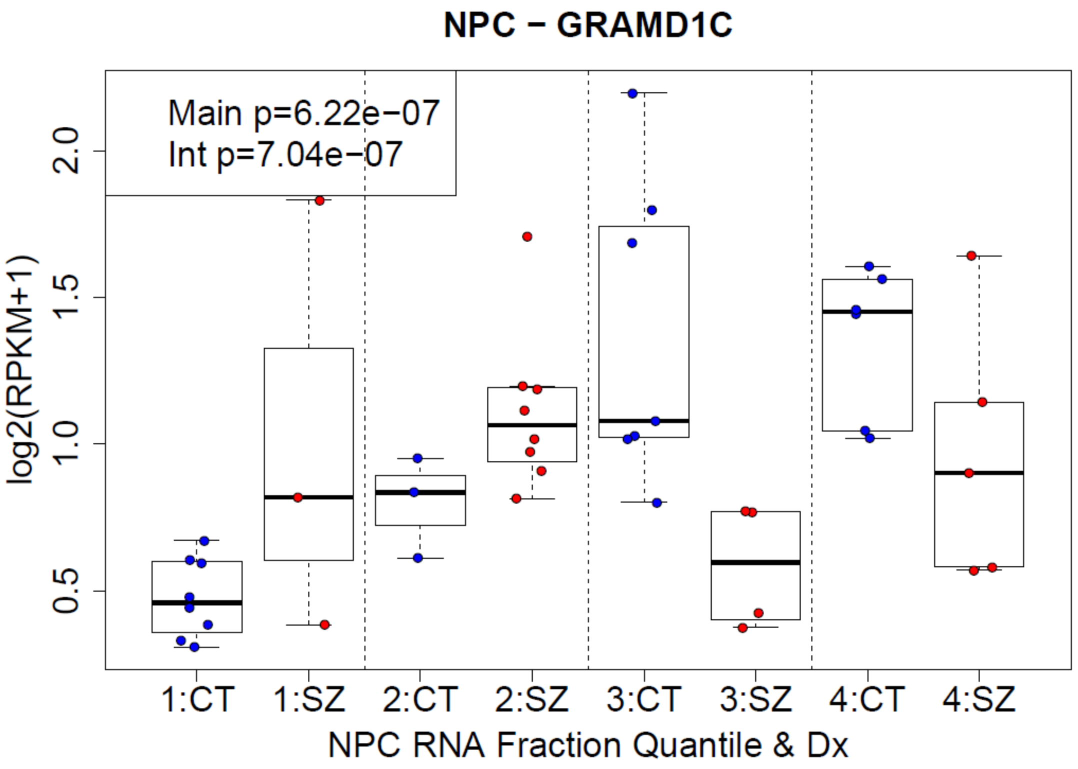

Supplementary Figure 15: Disease-associated effects within cell types. We used the Hoffman et al. data [5] comprised of healthy control and schizophrenia samples to show that our RNA fractions can be used for interaction modeling proposed by Zheng et al. [6] to identify cell type-dependent differential expression of covariates such as disease state in 78 genes that would otherwise be missed with more standard DE modeling techniques in bulk samples. The p-values show the significance of the diagnosis and diagnosis/NPC interaction terms from the interaction model.

Note above figure of GRAMD1C shows only the first page of 78 panels. For the full set of 78 panels, see:

Supplementary Data 8

Supplementary Tables

FEATURES	AD / NPC	NPC / Rosette	Rosette / Neuron	Across time-course	total tested
Genes	9,067	1,994	12,951	20,220	25,466
Exons	115,449	20,569	189,437	294,126	356,663
Junctions	57,074	9,816	107,110	181,657	281,497
Transcripts	12,616	2,109	31,672	50,733	73,140

FEATURES (PERCENTS)	AD / NPC	NPC / Rosette	Rosette / Neuron	Across time-course
Genes	35.60%	7.83%	50.86%	79.40%
Exons	32.37%	5.77%	53.11%	82.47%
Junctions	20.28%	3.49%	38.05%	64.53%
Transcripts	17.25%	2.88%	43.30%	69.36%

UNIQUE GENES	AD / NPC	NPC / Rosette	Rosette / Neuron	Across time-course	total tested
Genes	9,067	1,994	12,951	20,220	25,466
Exons	10,221	3,156	14,173	17,954	
Junctions	9,665	3,065	13,185	16,861	
Transcripts	7,868	1,752	14,262	18,359	

UNIQUE GENES (PERCENTS)	AD / NPC	NPC / Rosette	Rosette / Neuron	Across time-course
Genes	35.60%	7.83%	50.86%	79.40%
Exons	40.14%	12.39%	55.65%	70.50%
Junctions	37.95%	12.04%	51.77%	66.21%
Transcripts	30.90%	6.88%	56.00%	72.09%

Supplementary Table 1: Gene and feature-level differential expression at FDR < 1%, including the number of DE features, the number of unique genes, and the percent of DE features out of those tested.

CLASSES OF DE JUNCTIONS	AD / NPC	NPC / Rosette	Rosette / Neuron	Across time-course	total tested
Total	55,229	9,343	103,157	173,494	258,789
Fully annotated	50,106	8,305	92,625	151,194	202,339
Alt Start/End (% in snaptron)	3,325 (90.2)	704 (86.9)	7,199 (85.8)	15,002 (88.9)	38,147
Exon Skip (% in snaptron)	1,798 (95.8)	334 (91.5)	3,333 (92.8)	7,298 (94.1)	18,303

JUNCTION CLASSES (PERCENTS)	AD / NPC	NPC / Rosette	Rosette / Neuron	Across time-course
Total	20.28%	3.49%	38.05%	64.53%
Fully annotated	24.76%	4.10%	45.78%	74.72%
Alt Start/End	8.72%	1.85%	18.87%	39.33%
Exon Skip	9.82%	1.82%	18.21%	39.87%

Supplementary Table 2: Annotation classes of differentially expressed exon-exon junctions, including the number of DE features, and the percent of DE features out of those tested.

(A)

Gene	meanCounts	percentCounts	Sym	human_ortholog	BLAST_matches
ENSRNOG00000058555	420,864.40	32.59%	7SK.281		RN7SK, GSTA4
ENSRNOG00000058083	279,471	21.64%	Metazoa_SRP.22		RPS29
ENSRNOG00000056247	194,134.60	15.04%	7SK.191	RN7SKP176 (ENSG00000260682)	RN7SK, GSTA4
		69.27%			

(B)

Gene	meanCounts	percentCounts	Sym	rat_ortholog	BLAST_matches
ENSG00000274012.1	403,426	56.92%	RN7SL2	Metazoa_SRP (ENSRNOG00000054045)	
ENSG00000265735.2	94,335	13.31%	RN7SL5P	Metazoa_SRP (ENSRNOG00000055749, ENSRNOG00000051865, ENSRNOG00000055825, ENSRNOG00000048993)	
ENSG00000204628.11	29,452.75	4.16%	RACK1	Rack1 (ENSRNOG00000052620), AABR07047089.1 (ENSRNOG00000055063)	
		74.39%			

Supplementary Table 3: Details of the two sets of three highly expressed genes that contributed to the majority of reads that mapped across rat and human:

(A) Expressed rn6 genes after the purified rat astrocyte samples were mapped to the human hg38 genome, then the aligned reads were mapped back to rat.

(B) Expressed hg38 genes after human neuron samples were mapped to the rat rn6 genome, then the aligned reads were mapped back to human.

Gene	Study
RTN1	https://www.ncbi.nlm.nih.gov/pubmed/30352262
SNAP25	https://www.ncbi.nlm.nih.gov/pubmed/17728451
SCG2	https://www.ncbi.nlm.nih.gov/pubmed/24111984
SYNPR	--
MEG3	https://www.ncbi.nlm.nih.gov/pmc/articles/PMC5413565/<br>https://www.ncbi.nlm.nih.gov/pmc/articles/PMC4996761/
GABRA1	https://www.ncbi.nlm.nih.gov/pubmed/25352779<br>https://www.ncbi.nlm.nih.gov/pubmed/26050032
CCK	https://www.ncbi.nlm.nih.gov/pmc/articles/PMC3005241/
TSPAN7	https://www.ncbi.nlm.nih.gov/pubmed/22445342
VSNL1	--
ATP1B1	https://www.ncbi.nlm.nih.gov/pmc/articles/PMC2744953/
SCG5	https://www.ncbi.nlm.nih.gov/pubmed/23172224
GAD1	https://www.ncbi.nlm.nih.gov/pmc/articles/PMC3388776/
NAP1L3	--
GABRG2	https://www.ncbi.nlm.nih.gov/pubmed/10461223/
SYT1	https://www.ncbi.nlm.nih.gov/pubmed/29403034
SERPINI1	https://www.ncbi.nlm.nih.gov/pmc/articles/PMC4970828/
GABRB2	https://www.ncbi.nlm.nih.gov/pubmed/30013074
AC124303.2	--
VIP	https://www.ncbi.nlm.nih.gov/pubmed/12576099/<br>https://www.ncbi.nlm.nih.gov/pmc/articles/PMC1628303/
LINC00632	--
FGF12	https://www.ncbi.nlm.nih.gov/pmc/articles/PMC4555190/<br>https://www.ncbi.nlm.nih.gov/pmc/articles/PMC2974323/
PEG3	https://www.ncbi.nlm.nih.gov/pubmed/19224563
SCN2A	https://www.ncbi.nlm.nih.gov/pmc/articles/PMC6115194/
PNMA2	--
NRIP3	--

Supplementary Table 4: List of published studies including gene manipulation of the 25 neuronal markers used in our cell type deconvolution, showing evidence of studies exploring 18 of the 25 genes.

	Lab site	Day in Vitro	Cell Line
**iPSC**	2.37E-09	0.78	0.023
**NPC**	2.23E-03	6.25E-05	2.33E-03
**Fetal_replicating**	1.84E-07	0.017	0.41
**Fetal_quiescent**	0.034	0.17	0.54
**OPC**	0.029	0.50	0.15
**Neurons**	2.63E-13	0.16	2.07E-05
**Astrocytes**	1.43E-08	6.12E-11	2.16E-05
**Oligodendrocytes**	4.89E-07	0.33	6.38E-03
**Microglia**	3.39E-03	0.39	0.015
**Endothelial**	1.50E-07	0.050	8.68E-04

Supplementary Table 5: P-values of cell type proportion ANOVAs showing contributions to variance in Volpato et al. data [7], with the largest amount of variance being from technical variability contributed by lab site in eight of the ten cell types.

Supplementary References

1. Tsankov, A. M. *et al.* A qPCR ScoreCard quantifies the differentiation potential of human pluripotent stem cells. *Nat. Biotechnol.* **33,** 1182–1192 (2015).
2. van de Leemput, J. *et al.* CORTECON: a temporal transcriptome analysis of in vitro human cerebral cortex development from human embryonic stem cells. *Neuron* **83,** 51–68 (2014).
3. Close, J. L. *et al.* Single-Cell Profiling of an In Vitro Model of Human Interneuron Development Reveals Temporal Dynamics of Cell Type Production and Maturation. *Neuron* **96,** 949 (2017).
4. Jaffe, A. E. *et al.* Developmental and genetic regulation of the human cortex transcriptome illuminate schizophrenia pathogenesis. *Nat. Neurosci.* **21,** 1117–1125 (2018).
5. Hoffman, G. E. *et al.* Transcriptional signatures of schizophrenia in hiPSC-derived NPCs and neurons are concordant with post-mortem adult brains. *Nat. Commun.* **8,** 2225 (2017).
6. Zheng, S. C., Breeze, C. E., Beck, S. & Teschendorff, A. E. Identification of differentially methylated cell types in epigenome-wide association studies. *Nat. Methods* **15,** 1059–1066 (2018).
7. Volpato, V. *et al.* Reproducibility of Molecular Phenotypes after Long-Term Differentiation to Human iPSC-Derived Neurons: A Multi-Site Omics Study. *Stem Cell Rep.* **11,** 897–911 (2018).
