# Supplementary material for: Dissecting transcriptomic signatures of neuronal differentiation and maturation using iPSCs
Source: Nat Commun. 2020 Jan 23;11:462. doi: 10.1038/s41467-019-14266-z (PMC6978526; doi:10.1038/s41467-019-14266-z)
Supplement: Supplementary file 3 — Reporting Summary [file 41467_2019_14266_MOESM3_ESM.pdf]

nature research

Corresponding author(s): Andrew Jaffe; Ronald McKay

Reporting Summary

Nature Research wishes to improve the reproducibility of the work that we publish. This form provides structure for consistency and transparency in reporting. For further information on Nature Research policies, see Authors & Referees and the Editorial Policy Checklist.

Statistical parameters

When statistical analyses are reported, confirm that the following items are present in the relevant location (e.g. figure legend, table legend, main text, or Methods section).

n/a	Confirmed	
☐	☒	The exact sample size (n) for each experimental group/condition, given as a discrete number and unit of measurement
☐	☒	An indication of whether measurements were taken from distinct samples or whether the same sample was measured repeatedly
☐	☒	The statistical test(s) used AND whether they are one- or two-sided<br>*Only common tests should be described solely by name; describe more complex techniques in the Methods section.*
☐	☒	A description of all covariates tested
☐	☒	A description of any assumptions or corrections, such as tests of normality and adjustment for multiple comparisons
☐	☒	A full description of the statistics including central tendency (e.g. means) or other basic estimates (e.g. regression coefficient) AND variation (e.g. standard deviation) or associated estimates of uncertainty (e.g. confidence intervals)
☐	☒	For null hypothesis testing, the test statistic (e.g. F, t, r) with confidence intervals, effect sizes, degrees of freedom and P value noted<br>*Give P values as exact values whenever suitable.*
☒	☐	For Bayesian analysis, information on the choice of priors and Markov chain Monte Carlo settings
☒	☐	For hierarchical and complex designs, identification of the appropriate level for tests and full reporting of outcomes
☒	☐	Estimates of effect sizes (e.g. Cohen's d, Pearson's r), indicating how they were calculated
☐	☒	Clearly defined error bars<br>*State explicitly what error bars represent (e.g. SD, SE, CI)*

Our web collection on statistics for biologists may be useful.

Software and code

Policy information about availability of computer code

Data collection	No software was used.
Data analysis	HISAT2 v2.0.4, featureCounts/subread v1.5.0-p3, regtools v0.1.0, Salmon v0.7.2, Kallisto v0.43.0, bcftools v1.2, R v3.3 and v 3.4, Illustrator v22.0.1

For manuscripts utilizing custom algorithms or software that are central to the research but not yet described in published literature, software must be made available to editors/reviewers upon request. We strongly encourage code deposition in a community repository (e.g. GitHub). See the Nature Research guidelines for submitting code & software for further information.

Data

Policy information about availability of data

All manuscripts must include a data availability statement. This statement should provide the following information, where applicable:

- Accession codes, unique identifiers, or web links for publicly available datasets
- A list of figures that have associated raw data
- A description of any restrictions on data availability

Data availability: Links for downloading all sequencing reads, including both the time-course data and all reprocessed public data, are available at http://stemcell.libd.org/scb/.

Field-specific reporting

Please select the best fit for your research. If you are not sure, read the appropriate sections before making your selection.

☒ Life sciences ☐ Behavioural & social sciences ☐ Ecological, evolutionary & environmental sciences

For a reference copy of the document with all sections, see nature.com/authors/policies/ReportingSummary-flat.pdf

Life sciences study design

All studies must disclose on these points even when the disclosure is negative.

Sample size	The sample size was picked based on the literature and our previous experiences with developmental changes occurring during stem cell differentiation
Data exclusions	Data were excluded based on RNA sequencing quality control metrics and genotype identity checks.
Replication	We downloaded and identically reprocessed other publicly available RNA-seq datasets, and attempted to replicate many of our key results.
Randomization	Samples were randomized for sequencing - other outcomes of interest, like developmental time, were not randomized.
Blinding	Investigators were not blind to the outcome as cells at early timepoints look much differently than cells at the end of the time course.

Reporting for specific materials, systems and methods

Materials & experimental systems

n/a	Involved in the study	
☐	☐	Unique biological materials
☒	☐	Antibodies
☐	☒	Eukaryotic cell lines
☒	☐	Palaeontology
☐	☒	Animals and other organisms
☐	☒	Human research participants

Methods

n/a	Involved in the study	
☒	☐	ChIP-seq
☒	☐	Flow cytometry
☒	☐	MRI-based neuroimaging

Unique biological materials

Policy information about availability of materials

Obtaining unique materials	Unique materials are available from the authors

Eukaryotic cell lines

Policy information about cell lines

Cell line source(s)	iPSCs derived from skin
Authentication	We genotyped coding variants from each RNA-seq sample to confirm identity. We also performed Fluidigm-based PCR to confirm pluripotency
Mycoplasma contamination	Cell lines were not tested for mycoplasma contamination
Commonly misidentified lines (See ICLAC register)	N/A

Animals and other organisms

Policy information about studies involving animals; ARRIVE guidelines recommended for reporting animal research

Laboratory animals	The study did not involve animals

Wild animals	The study did not involve wild animals
Field-collected samples	The study did not samples collected in the field

Human research participants

Policy information about studies involving human research participants

Population characteristics	N/A
Recruitment	N/A
