# Supplementary material for: Dissecting transcriptomic signatures of neuronal differentiation and maturation using iPSCs
Source: Nat Commun. 2020 Jan 23;11:462. doi: 10.1038/s41467-019-14266-z (PMC6978526; doi:10.1038/s41467-019-14266-z)
Supplement: Supplementary file 4 — Description of Additional Supplementary Files [file 41467_2019_14266_MOESM4_ESM.pdf]

Description of Additional Supplementary Files

File Name: Supplementary Data 1
Description: List of gene set enrichment analysis terms and significance levels of WGCNA modules.

File Name: Supplementary Data 2
Description: Details of the 3214 genes differentially expressed (FDR<0.05) between the neurons alone and neurons co-cultured on rodent astrocytes.

File Name: Supplementary Data 3
Description: Cellular component GO enrichment terms of differentially expressed genes, separated by up-regulation and down-regulation, from the astrocyte analysis comparing neurons alone to neurons on astrocytes.

File Name: Supplementary Data 4
Description: List and Z-scores of 228 unique genes selected for the regression calibration design matrix for cellular proportion model.

File Name: Supplementary Data 5
Description: Boxplots of the standardized expression levels of all 131 genes that distinguish iPSCs, NPCs, fetal replicating neurons, fetal quiescent neurons, adult neurons, and adult endothelial cells.

File Name: Supplementary Data 6
Description: List and Z-scores of the 169 unique genes of the regression calibration design matrix for the developmental brain stage model

File Name: Supplementary Data 7
Description: Boxplots of 78 genes showing RNA fractions used in interaction modeling to identify cell type-dependent differential expression by disease state that would otherwise be missed with more standard DE modeling techniques in bulk samples.

File Name: Supplementary Data 8
Description: Differential expression results listing the 78 genes found significantly differentially expressed by schizophrenia diagnosis in the interaction modeling strategy, showing both the main term and interaction term results.
