# Supplementary material for: Dissecting transcriptomic signatures of neuronal differentiation and maturation using iPSCs
Source: Nat Commun. 2020 Jan 23;11:462. doi: 10.1038/s41467-019-14266-z (PMC6978526; doi:10.1038/s41467-019-14266-z)
Supplement: Supplementary file 9 — Supplementary Data 5 [file 41467_2019_14266_MOESM9_ESM.pdf]

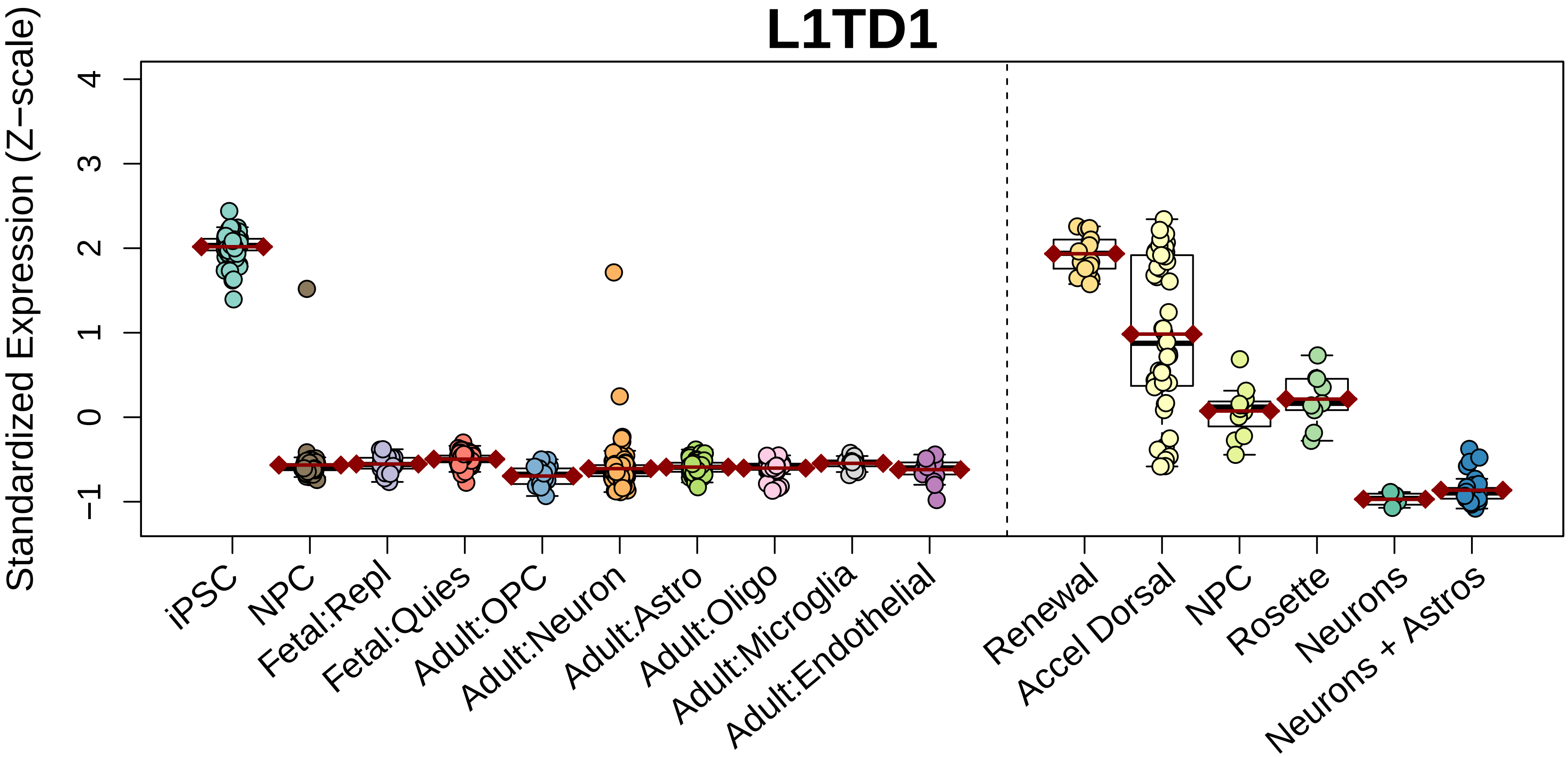

L1TD1
Standardized Expression (Z−scale)
4
3
2
1
0
−1
iPSC
NPC
Fetal:Repl
Fetal:Quies
Adult:OPC
Adult:Neuron
Adult:Astro
Adult:Oligo
Adult:Microglia
Adult:Endothelial
Renewal
Accel Dorsal
NPC
Rosette
Neurons
Neurons + Astros

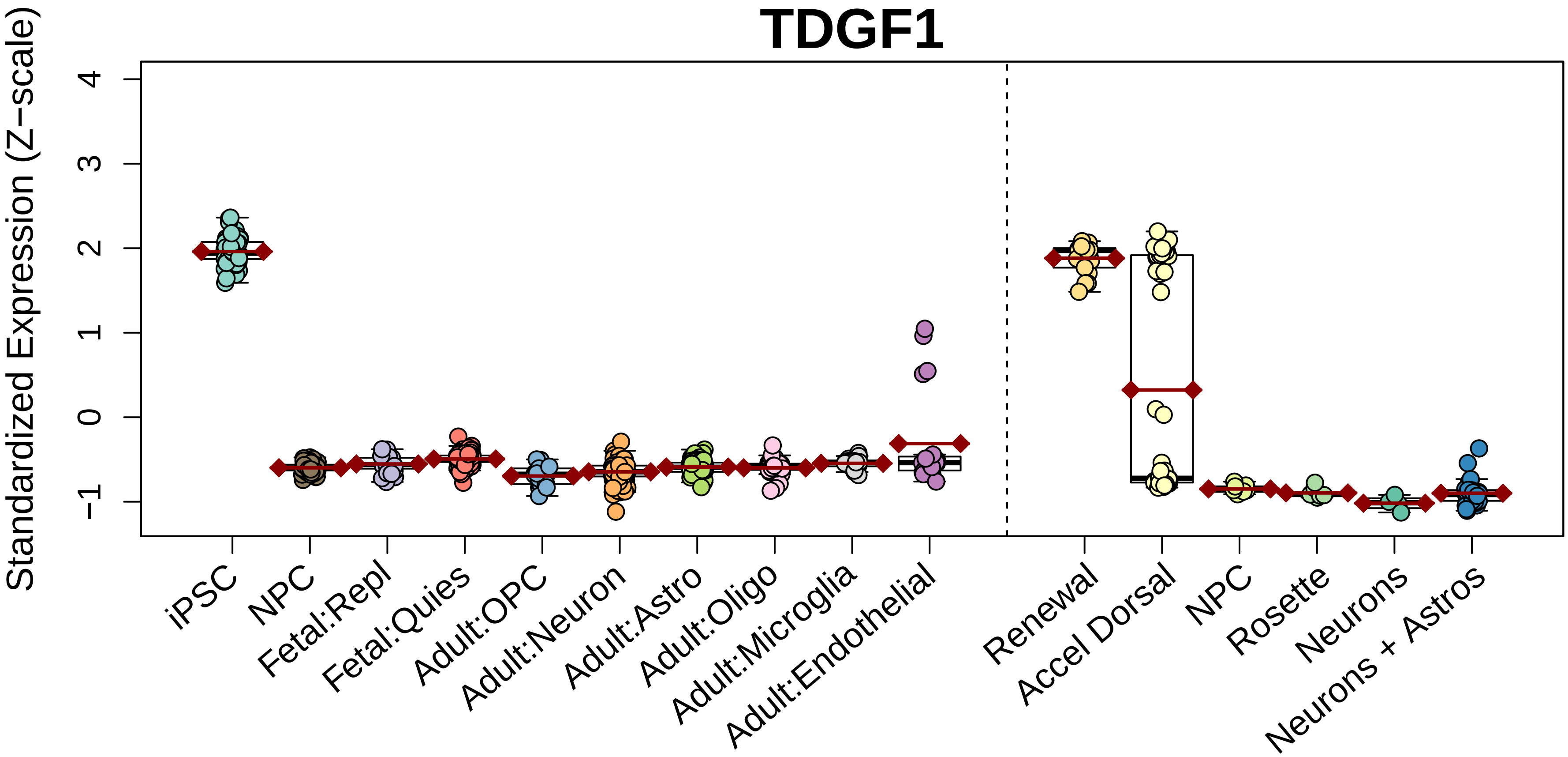

TDGF1
Standardized Expression (Z−scale)
4
3
2
1
0
−1
iPSC
NPC
Fetal:Repl
Fetal:Quies
Adult:OPC
Adult:Neuron
Adult:Astro
Adult:Oligo
Adult:Microglia
Adult:Endothelial
Renewal
Accel Dorsal
NPC
Rosette
Neurons
Neurons + Astros

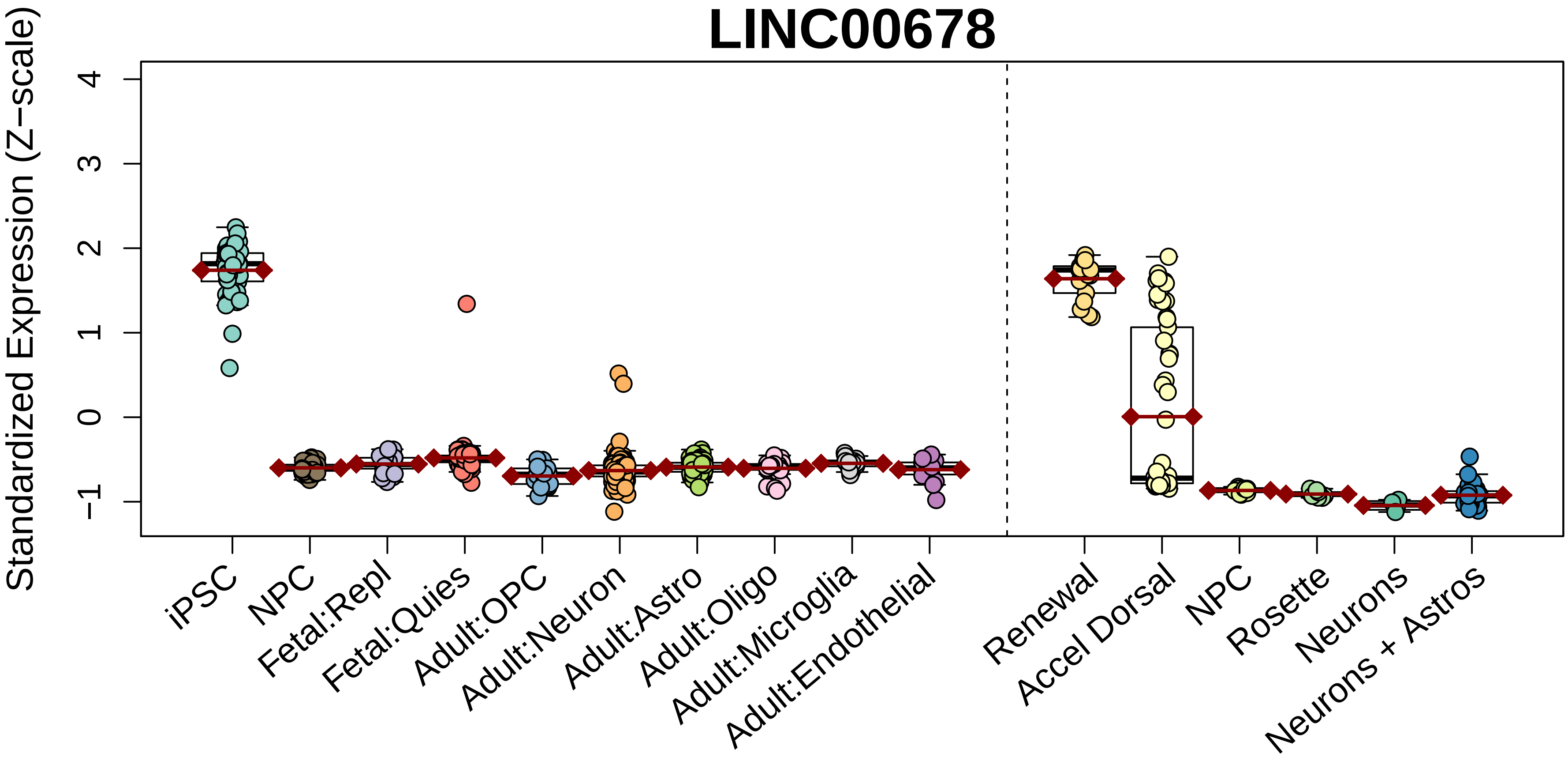
LINC00678
Standardized Expression (Z−scale)
4
3
2
1
0
−1
iPSC
NPC
Fetal:Repl
Fetal:Quies
Adult:OPC
Adult:Neuron
Adult:Astro
Adult:Oligo
Adult:Microglia
Adult:Endothelial
Renewal
Accel Dorsal
NPC
Rosette
Neurons
Neurons + Astros

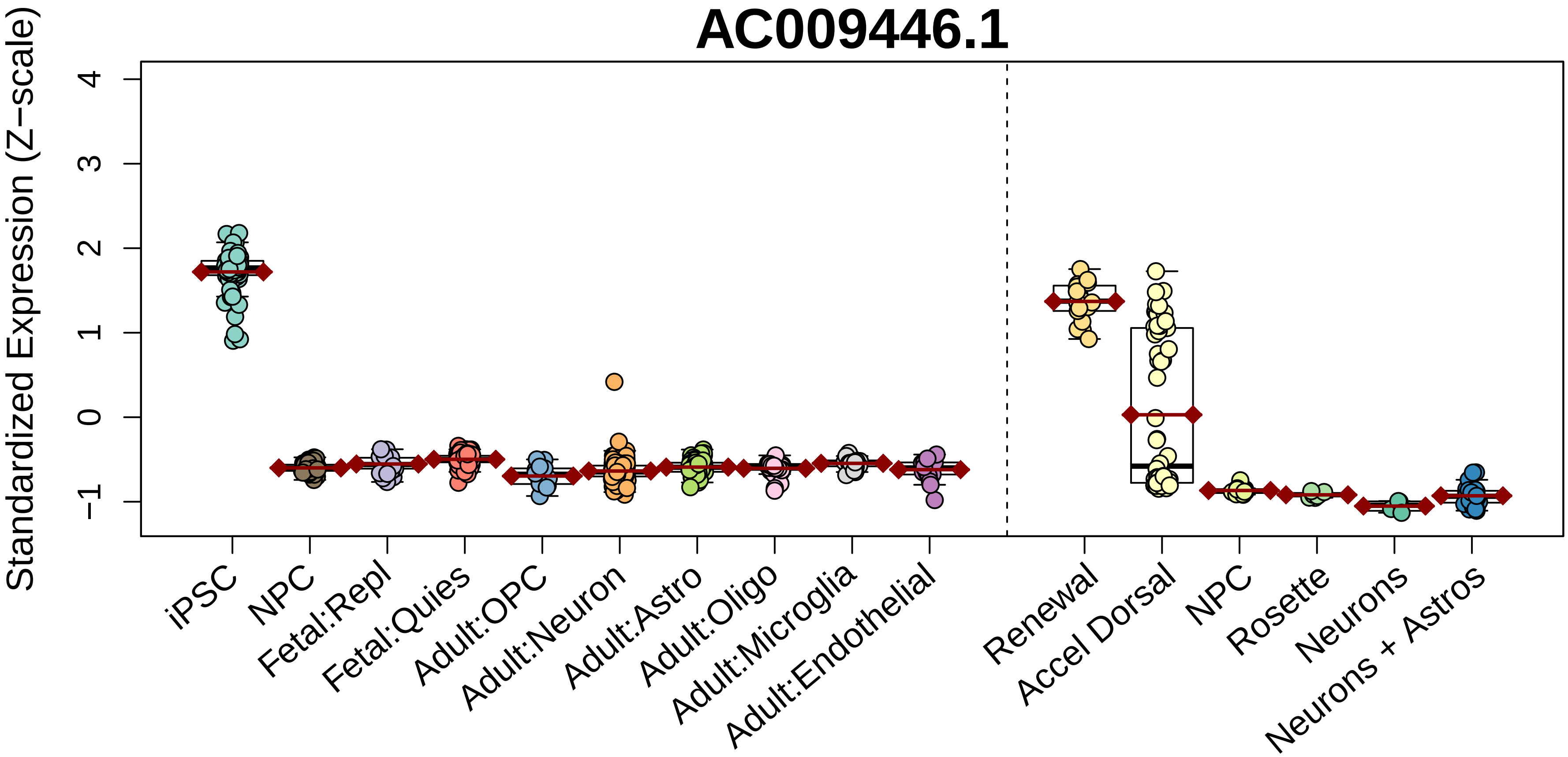
AC009446.1
Standardized Expression (Z−scale)
4
3
2
1
0
−1
iPSC
NPC
Fetal:Repl
Fetal:Quies
Adult:OPC
Adult:Neuron
Adult:Astro
Adult:Oligo
Adult:Microglia
Adult:Endothelial
Renewal
Accel Dorsal
NPC
Rosette
Neurons
Neurons + Astros

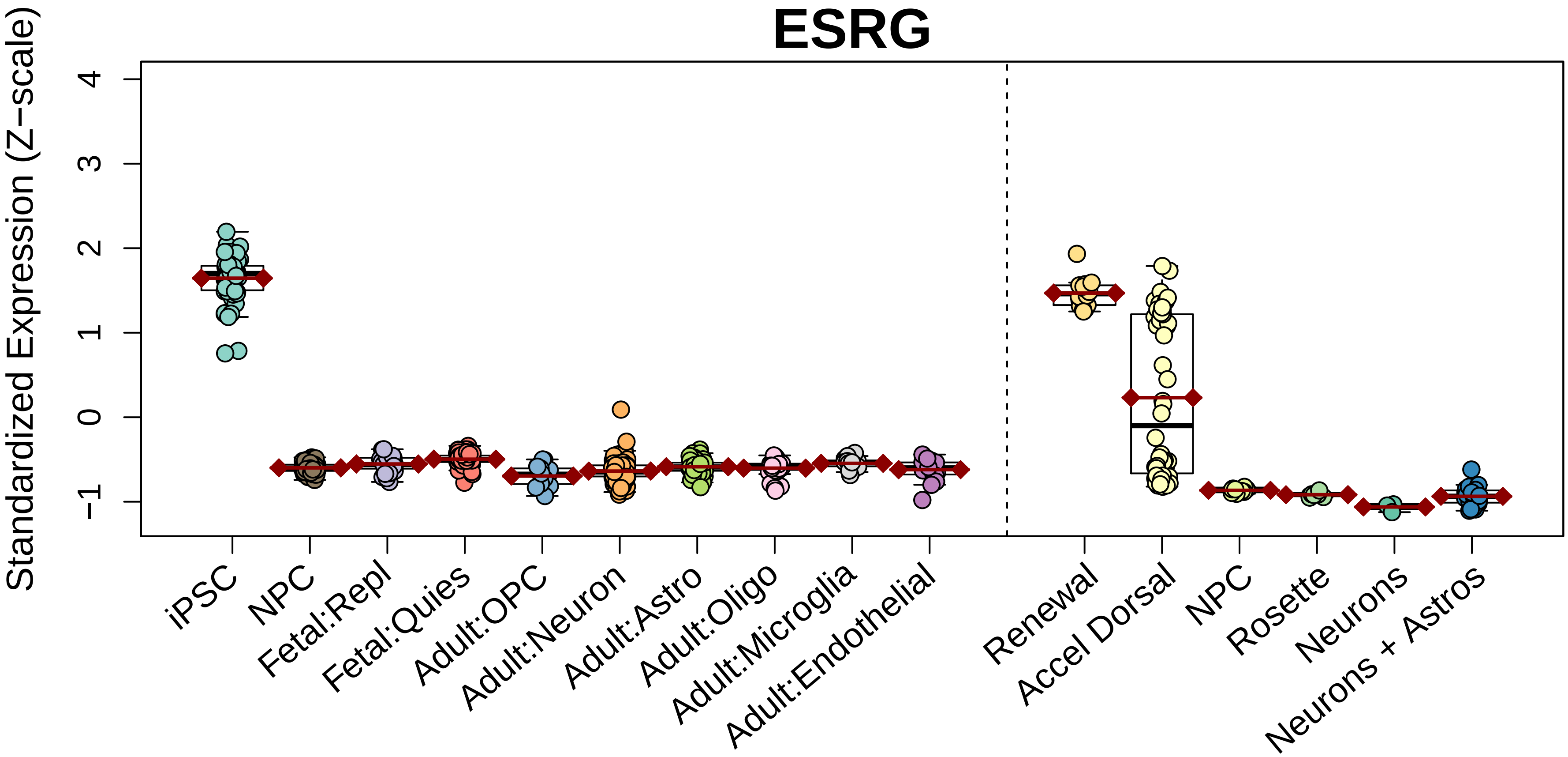

ESRG
Standardized Expression (Z−scale)
4
3
2
1
0
−1
iPSC
NPC
Fetal:Repl
Fetal:Quies
Adult:OPC
Adult:Neuron
Adult:Astro
Adult:Oligo
Adult:Microglia
Adult:Endothelial
Renewal
Accel Dorsal
NPC
Rosette
Neurons
Neurons + Astros

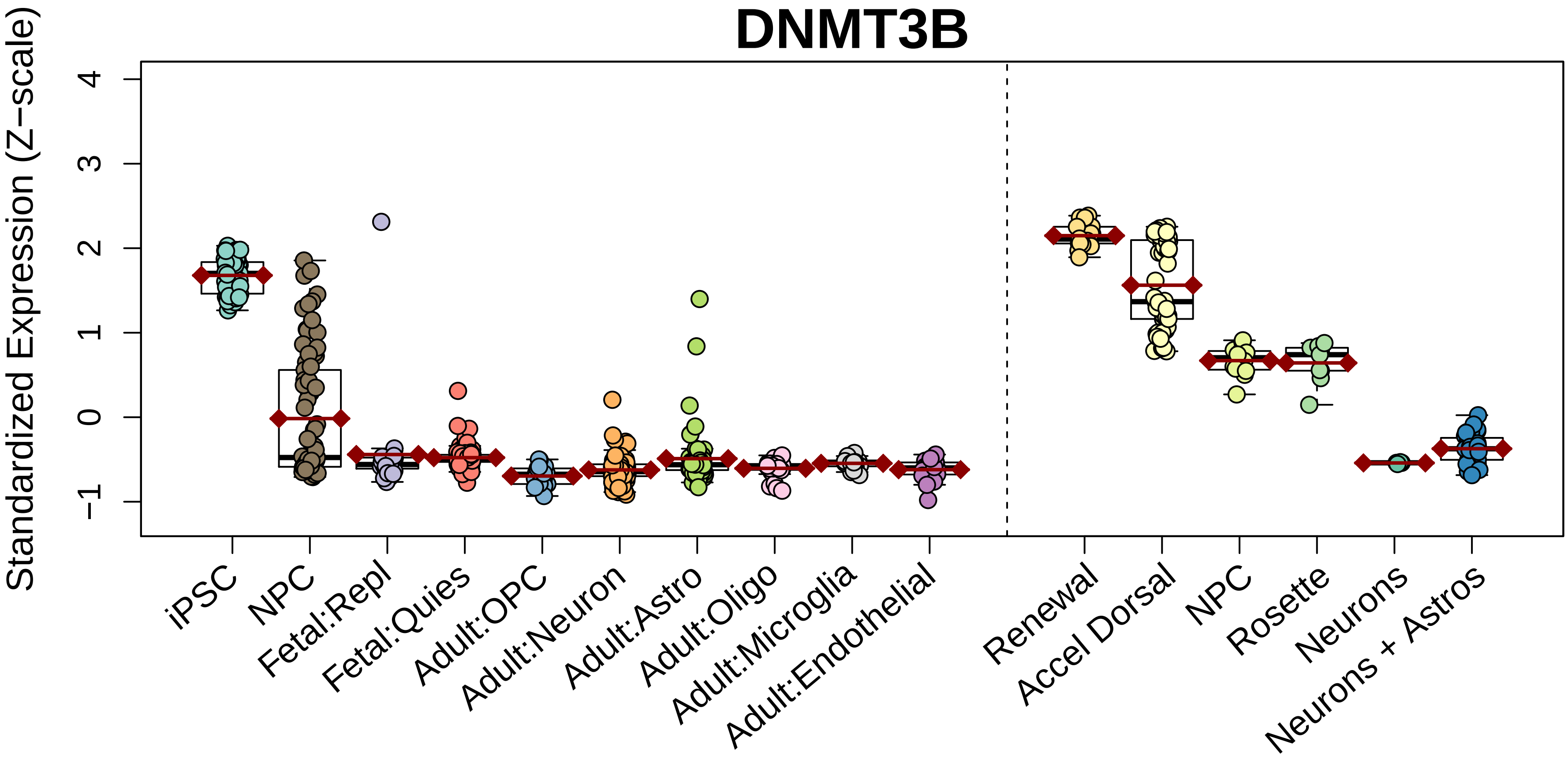

DNMT3B
Standardized Expression (Z−scale)
4
3
2
1
0
−1
iPSC
NPC
Fetal:Repl
Fetal:Quies
Adult:OPC
Adult:Neuron
Adult:Astro
Adult:Oligo
Adult:Microglia
Adult:Endothelial
Renewal
Accel Dorsal
NPC
Rosette
Neurons
Neurons + Astros

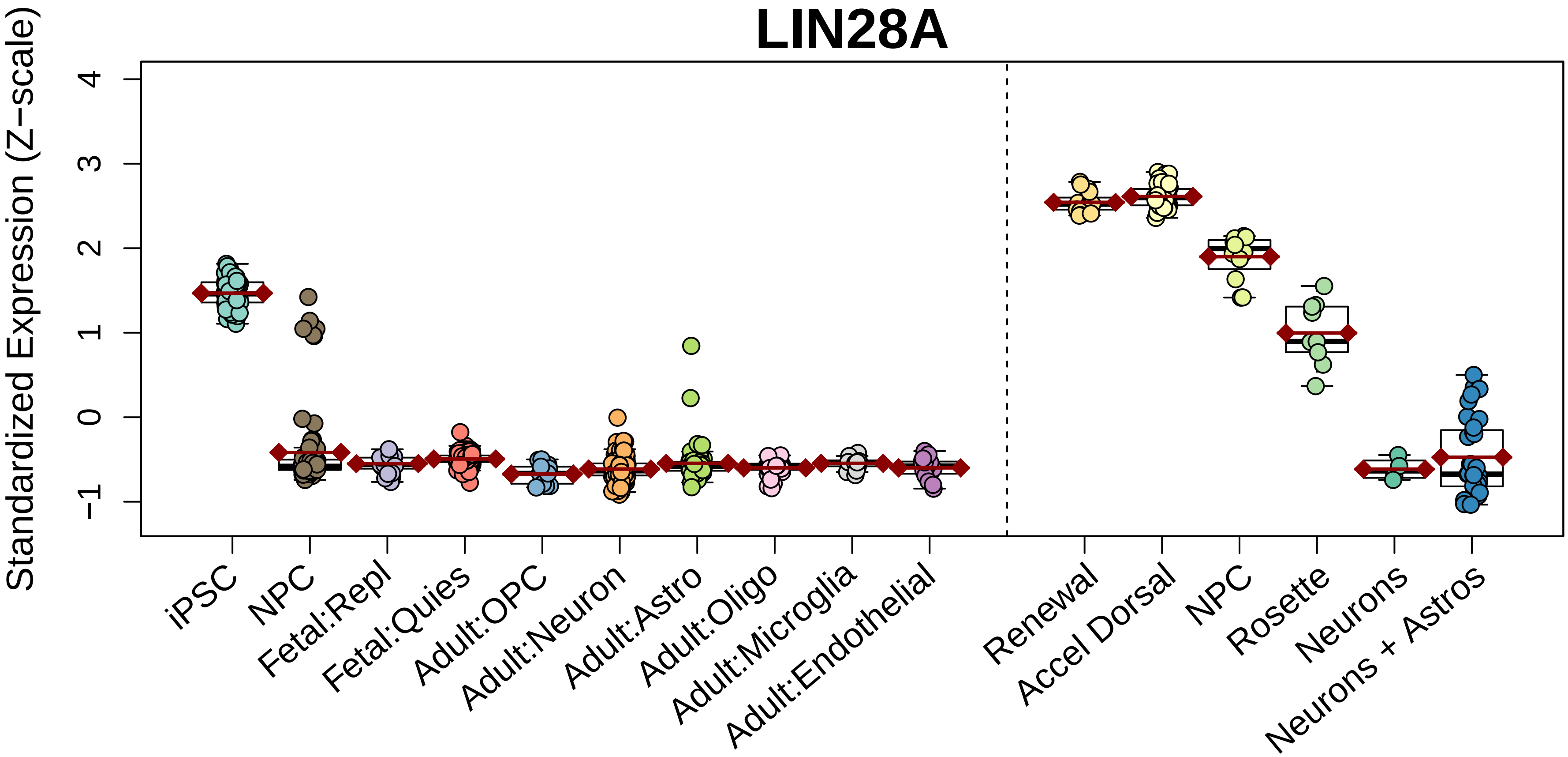
LIN28A
Standardized Expression (Z−scale)
−1
0
1
2
3
4
iPSC
NPC
Fetal:Repl
Fetal:Quies
Adult:OPC
Adult:Neuron
Adult:Astro
Adult:Oligo
Adult:Microglia
Adult:Endothelial
Renewal
Accel Dorsal
NPC
Rosette
Neurons
Neurons + Astros

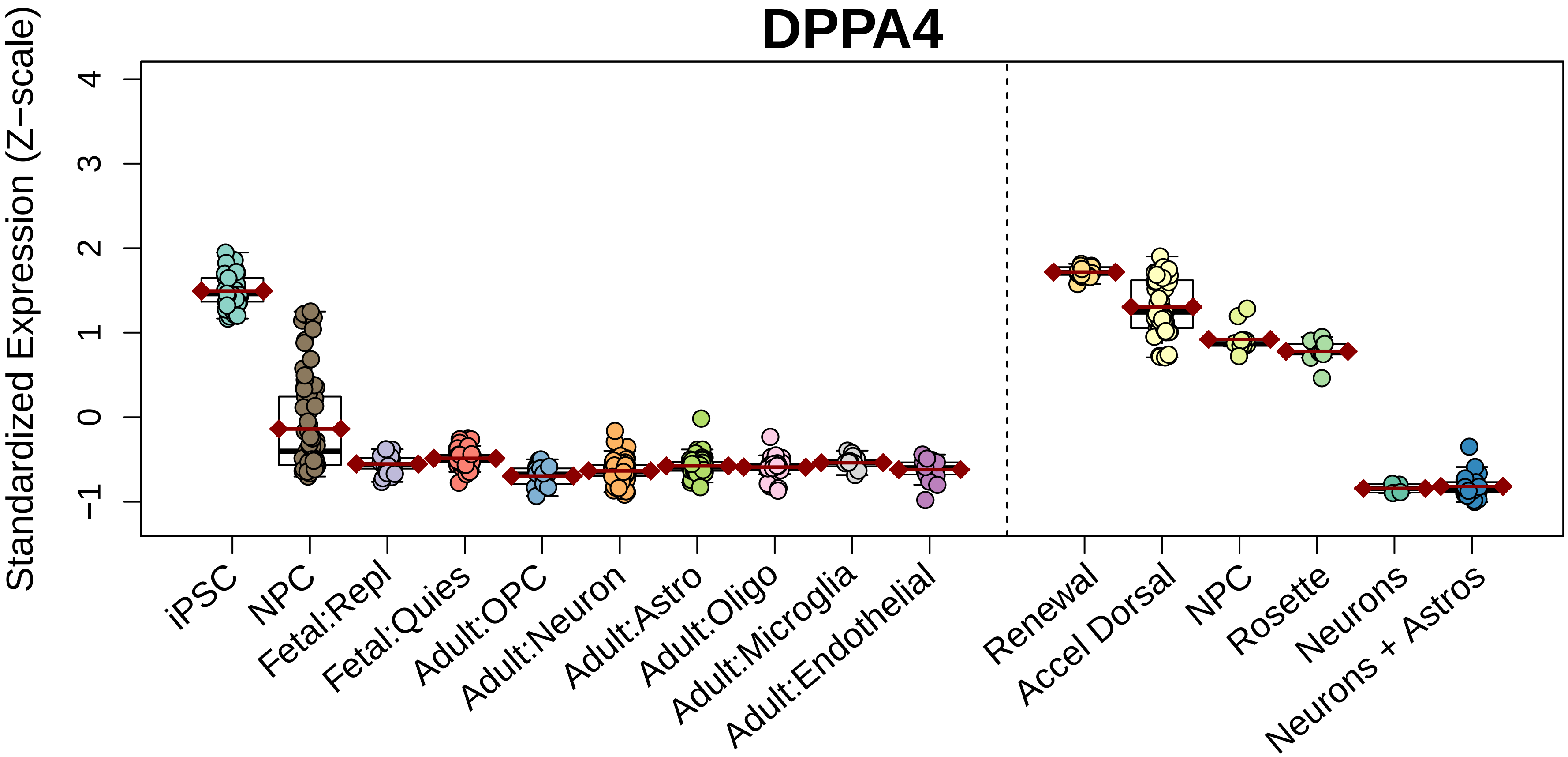

DPPA4
Standardized Expression (Z−scale)
4
3
2
1
0
−1
iPSC
NPC
Fetal:Repl
Fetal:Quies
Adult:OPC
Adult:Neuron
Adult:Astro
Adult:Oligo
Adult:Microglia
Adult:Endothelial
Renewal
Accel Dorsal
NPC
Rosette
Neurons
Neurons + Astros

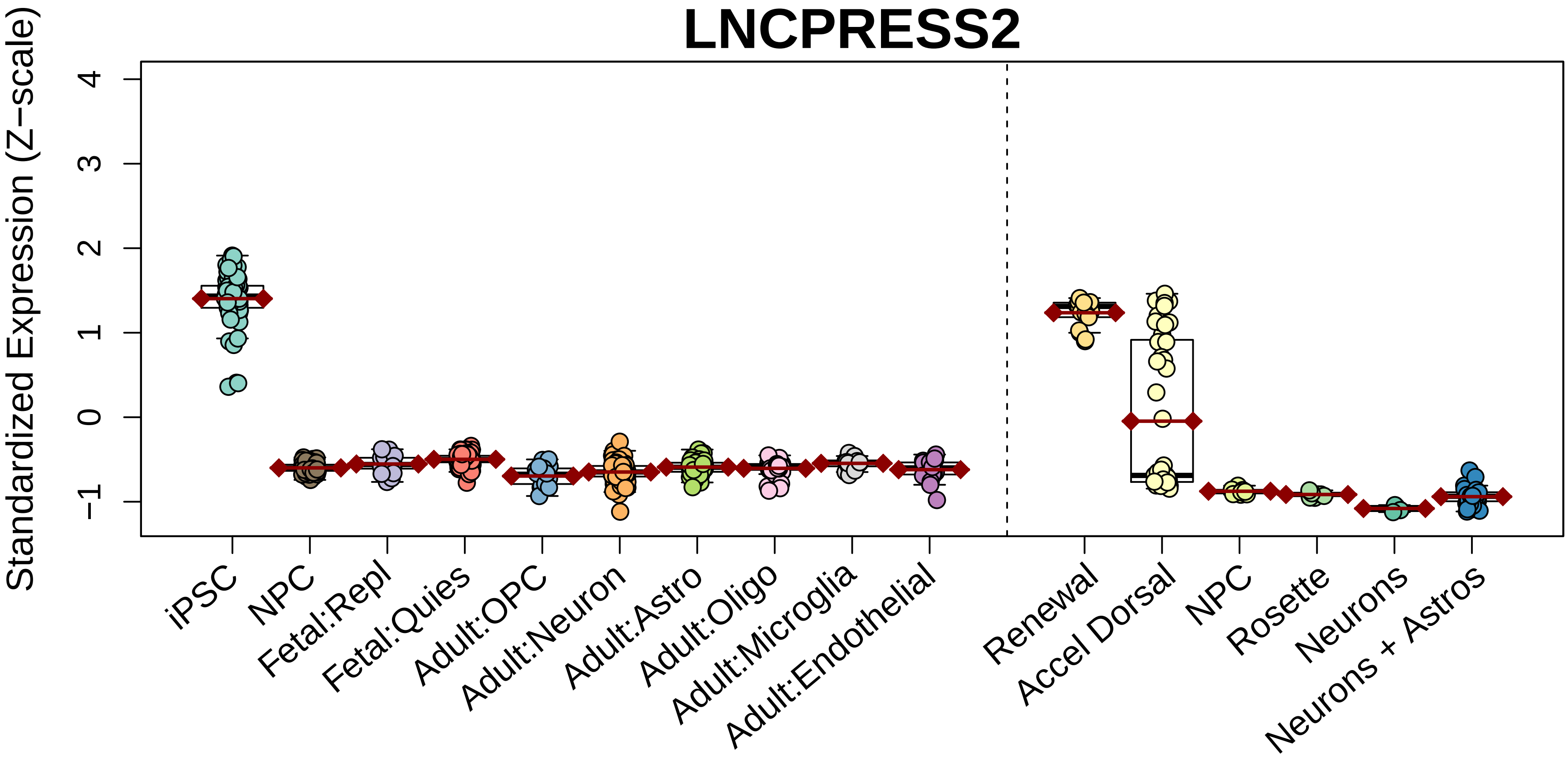

LNCPRESS2
Standardized Expression (Z−scale)
4
3
2
1
0
−1
iPSC
NPC
Fetal:Repl
Fetal:Quies
Adult:OPC
Adult:Neuron
Adult:Astro
Adult:Oligo
Adult:Microglia
Adult:Endothelial
Renewal
Accel Dorsal
NPC
Rosette
Neurons
Neurons + Astros

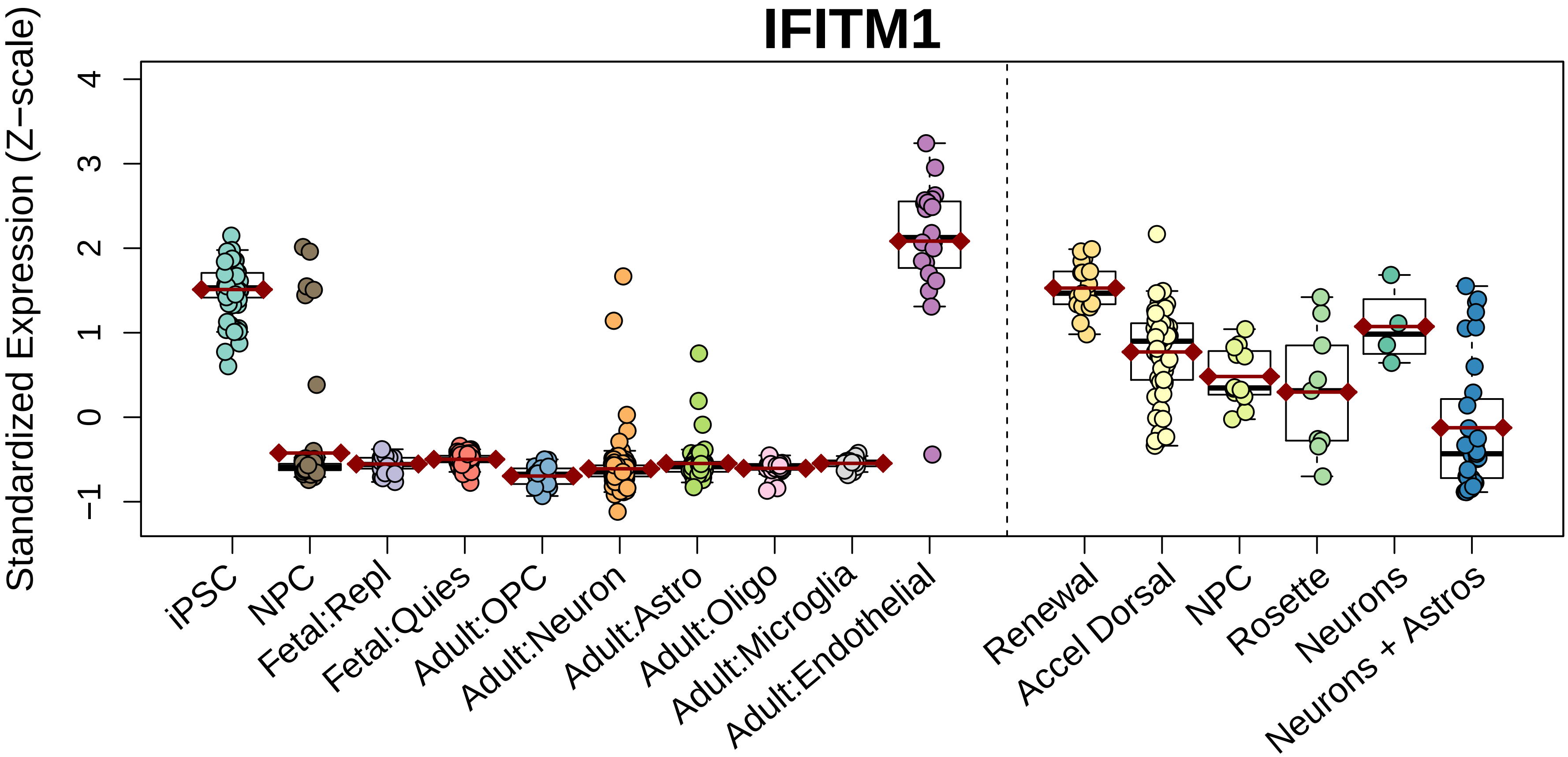

IFITM1
Standardized Expression (Z−scale)
4
3
2
1
0
−1
iPSC
NPC
Fetal:Repl
Fetal:Quies
Adult:OPC
Adult:Neuron
Adult:Astro
Adult:Oligo
Adult:Microglia
Adult:Endothelial
Renewal
Accel Dorsal
NPC
Rosette
Neurons
Neurons + Astros

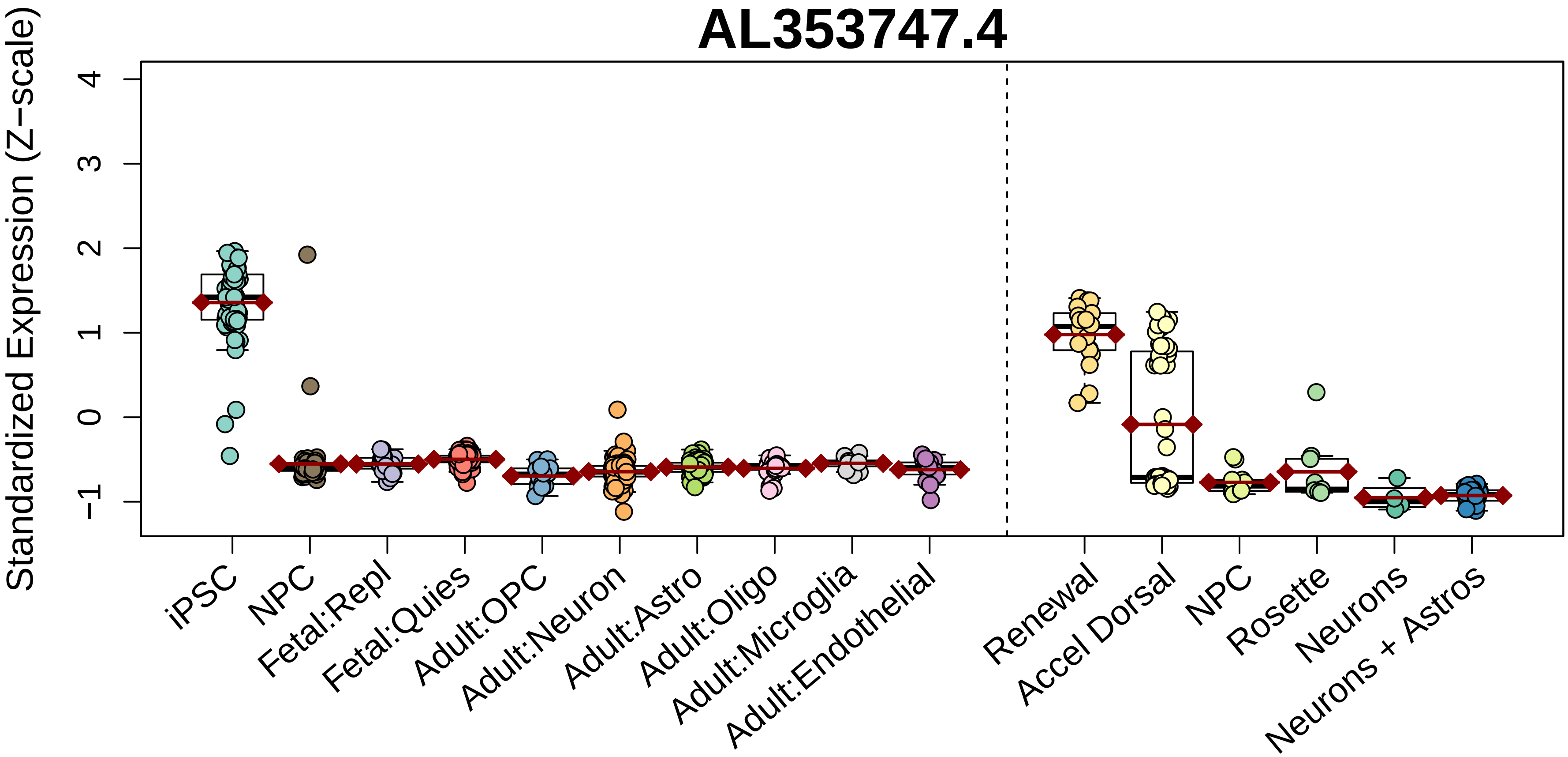

AL353747.4
Standardized Expression (Z−scale)
4
3
2
1
0
−1
iPSC
NPC
Fetal:Repl
Fetal:Quies
Adult:OPC
Adult:Neuron
Adult:Astro
Adult:Oligo
Adult:Microglia
Adult:Endothelial
Renewal
Accel Dorsal
NPC
Rosette
Neurons
Neurons + Astros

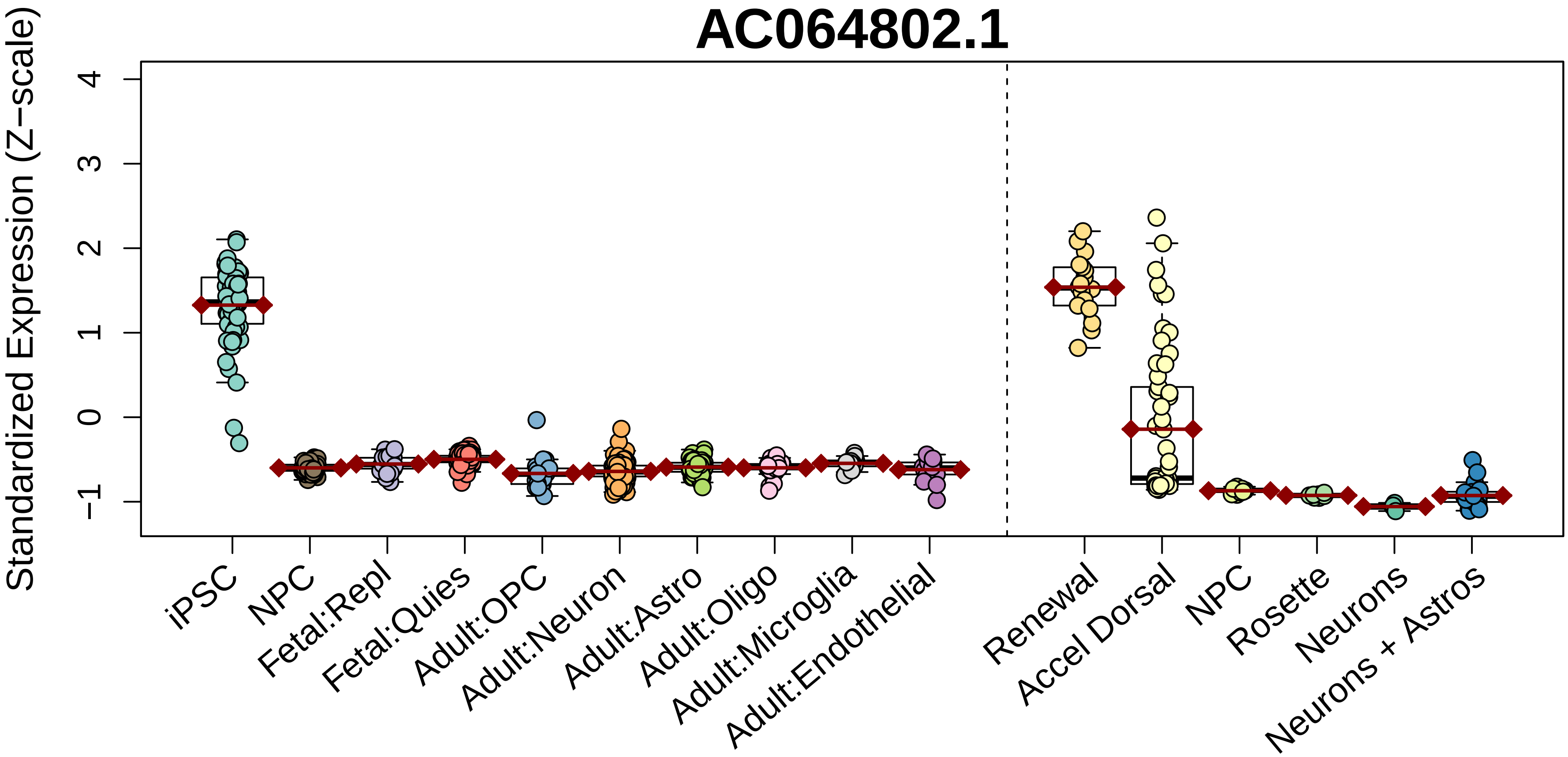

AC064802.1
Standardized Expression (Z−scale)
4
3
2
1
0
−1
iPSC
NPC
Fetal:Repl
Fetal:Quies
Adult:OPC
Adult:Neuron
Adult:Astro
Adult:Oligo
Adult:Microglia
Adult:Endothelial
Renewal
Accel Dorsal
NPC
Rosette
Neurons
Neurons + Astros

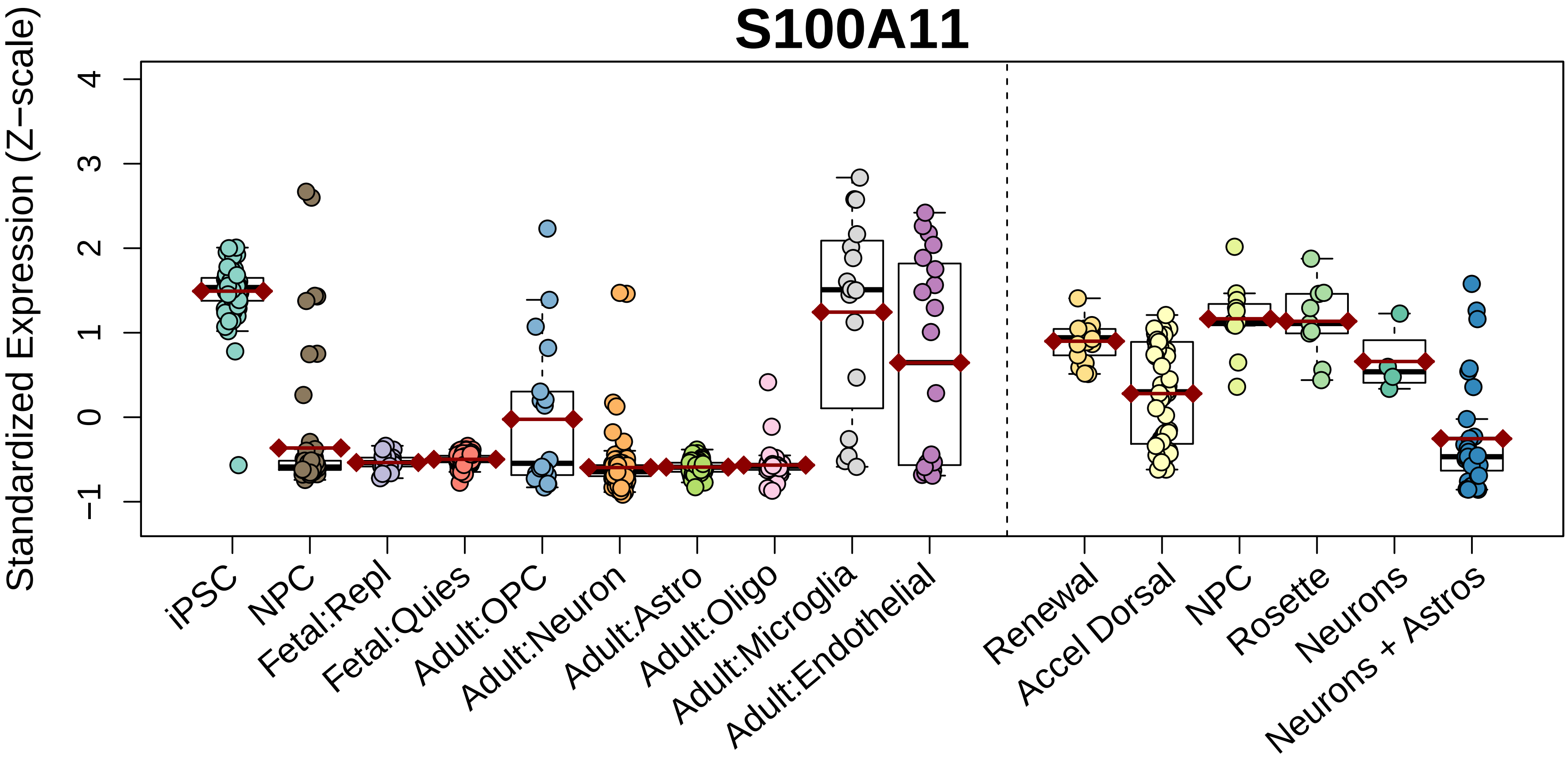

S100A11
Standardized Expression (Z−scale)
4
3
2
1
0
−1
iPSC
NPC
Fetal:Repl
Fetal:Quies
Adult:OPC
Adult:Neuron
Adult:Astro
Adult:Oligo
Adult:Microglia
Adult:Endothelial
Renewal
Accel Dorsal
NPC
Rosette
Neurons
Neurons + Astros

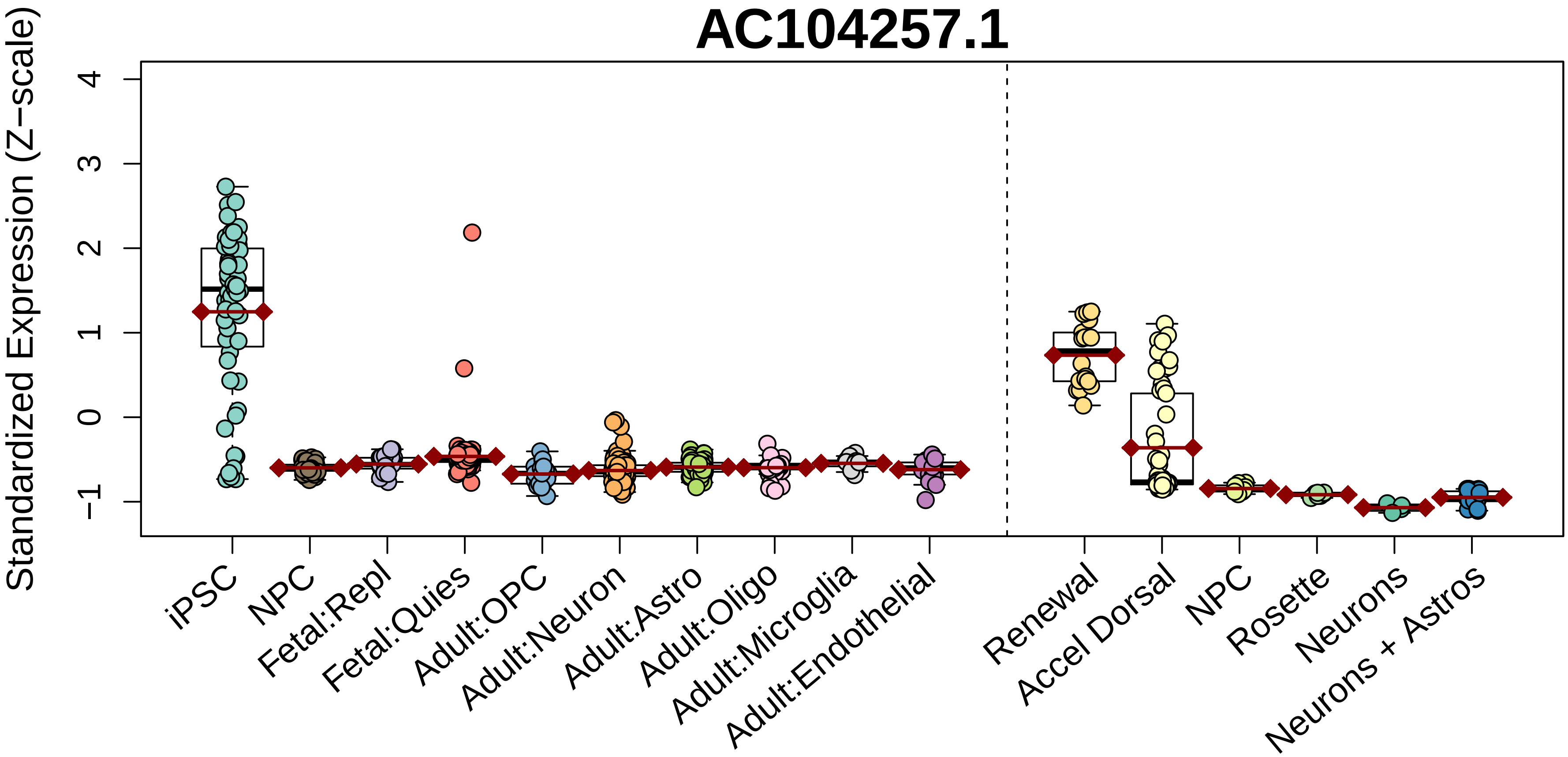
AC104257.1
Standardized Expression (Z−scale)
4
3
2
1
0
−1
iPSC
NPC
Fetal:Repl
Fetal:Quies
Adult:OPC
Adult:Neuron
Adult:Astro
Adult:Oligo
Adult:Microglia
Adult:Endothelial
Renewal
Accel Dorsal
NPC
Rosette
Neurons
Neurons + Astros

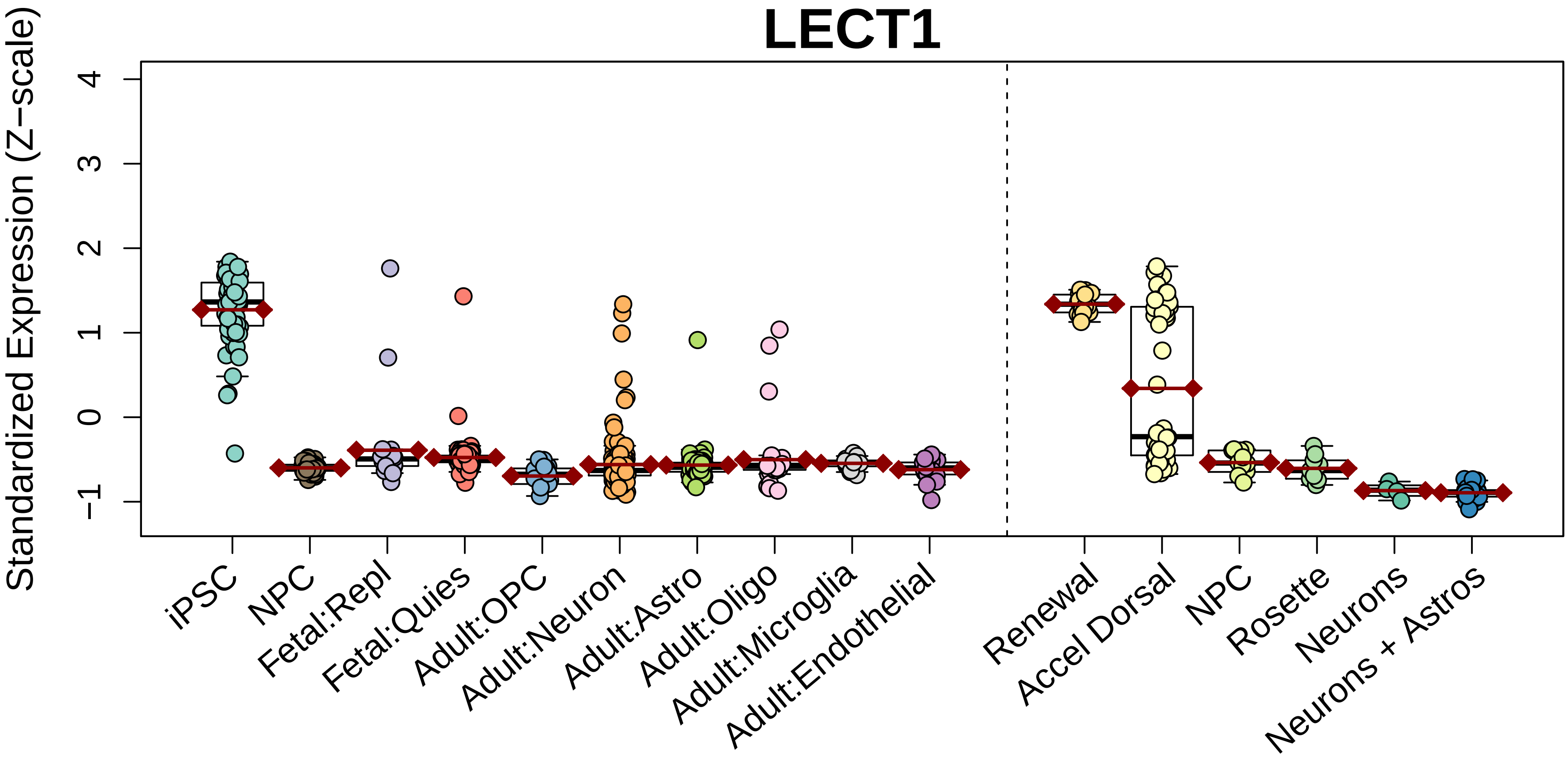

LECT1
Standardized Expression (Z−scale)
4
3
2
1
0
−1
iPSC
NPC
Fetal:Repl
Fetal:Quies
Adult:OPC
Adult:Neuron
Adult:Astro
Adult:Oligo
Adult:Microglia
Adult:Endothelial
Renewal
Accel Dorsal
NPC
Rosette
Neurons
Neurons + Astros

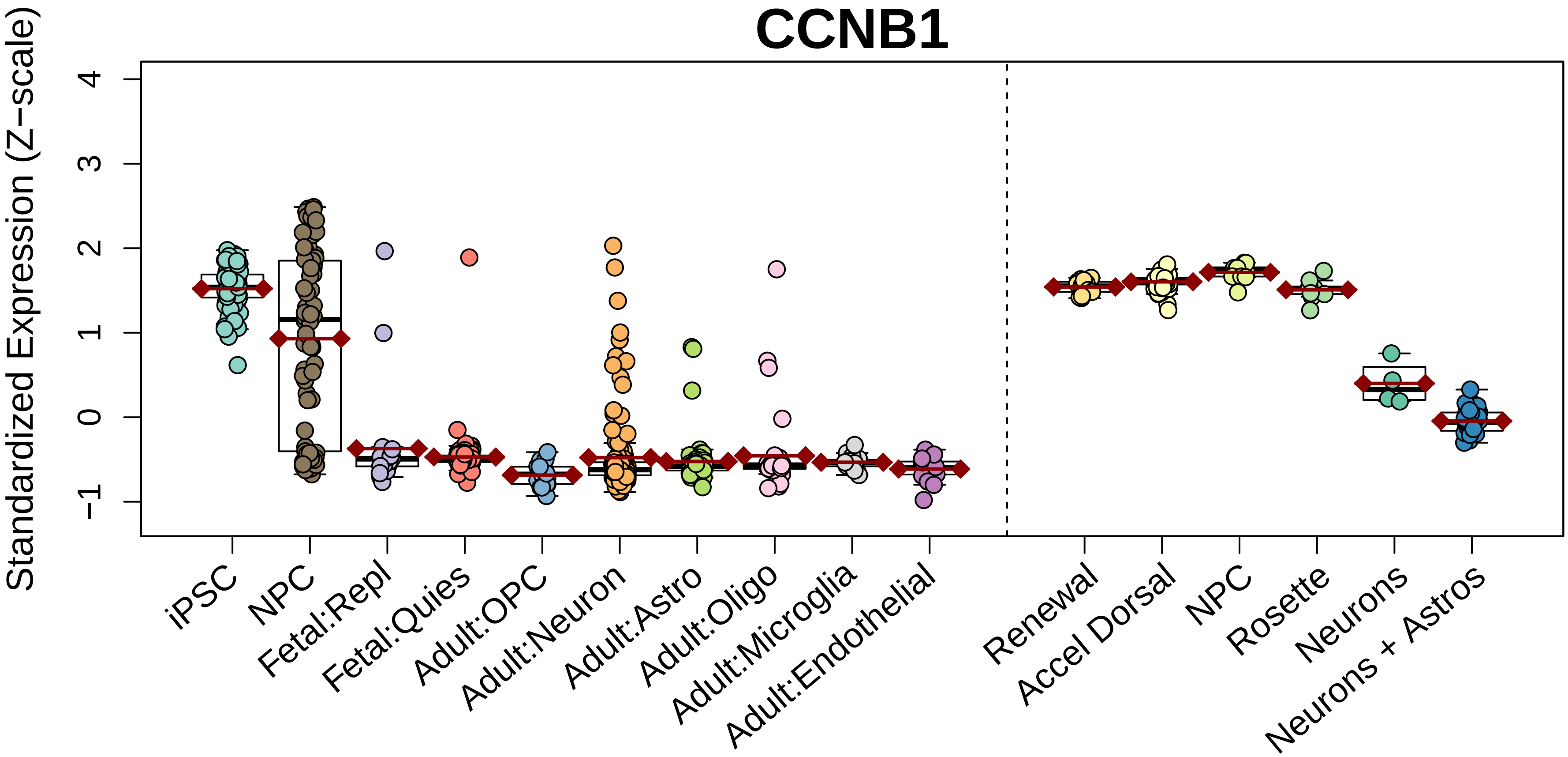

CCNB1
Standardized Expression (Z−scale)
−1
0
1
2
3
4
iPSC
NPC
Fetal:Repl
Fetal:Quies
Adult:OPC
Adult:Neuron
Adult:Astro
Adult:Oligo
Adult:Microglia
Adult:Endothelial
Renewal
Accel Dorsal
NPC
Rosette
Neurons
Neurons + Astros

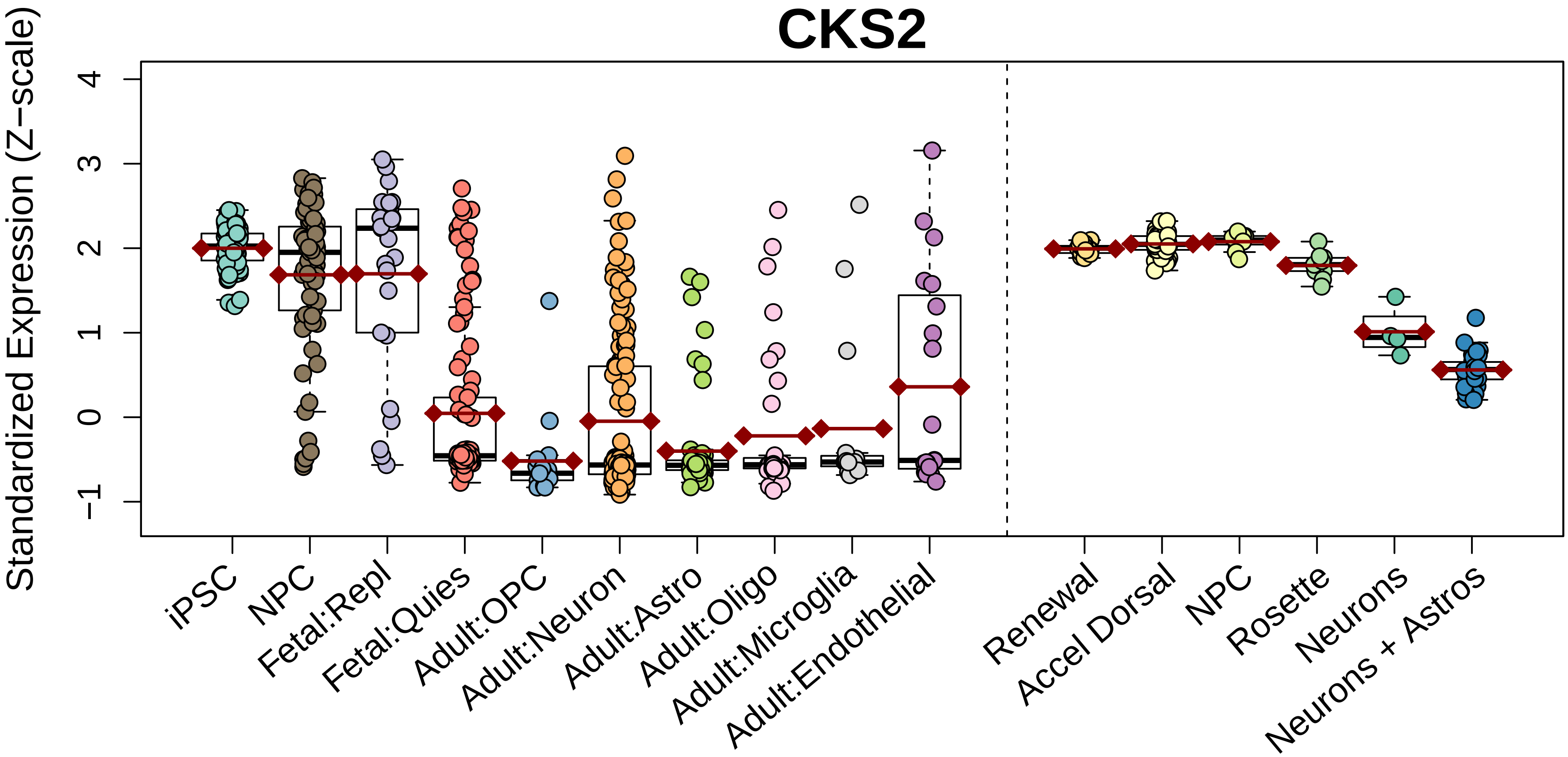

CKS2
Standardized Expression (Z−scale)
4
3
2
1
0
−1
iPSC
NPC
Fetal:Repl
Fetal:Quies
Adult:OPC
Adult:Neuron
Adult:Astro
Adult:Oligo
Adult:Microglia
Adult:Endothelial
Renewal
Accel Dorsal
NPC
Rosette
Neurons
Neurons + Astros

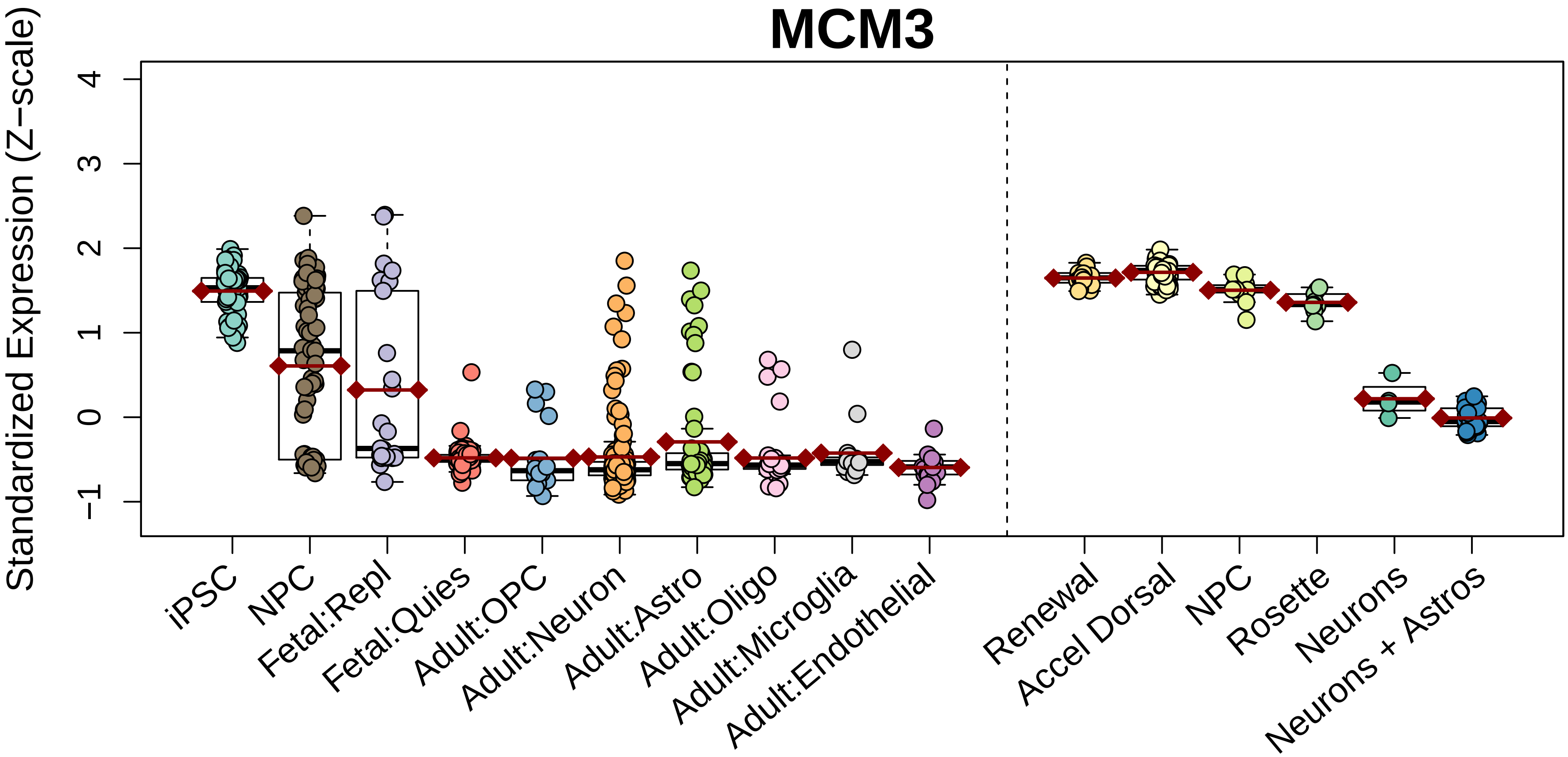

MCM3
Standardized Expression (Z−scale)
4
3
2
1
0
−1
iPSC
NPC
Fetal:Repl
Fetal:Quies
Adult:OPC
Adult:Neuron
Adult:Astro
Adult:Oligo
Adult:Microglia
Adult:Endothelial
Renewal
Accel Dorsal
NPC
Rosette
Neurons
Neurons + Astros

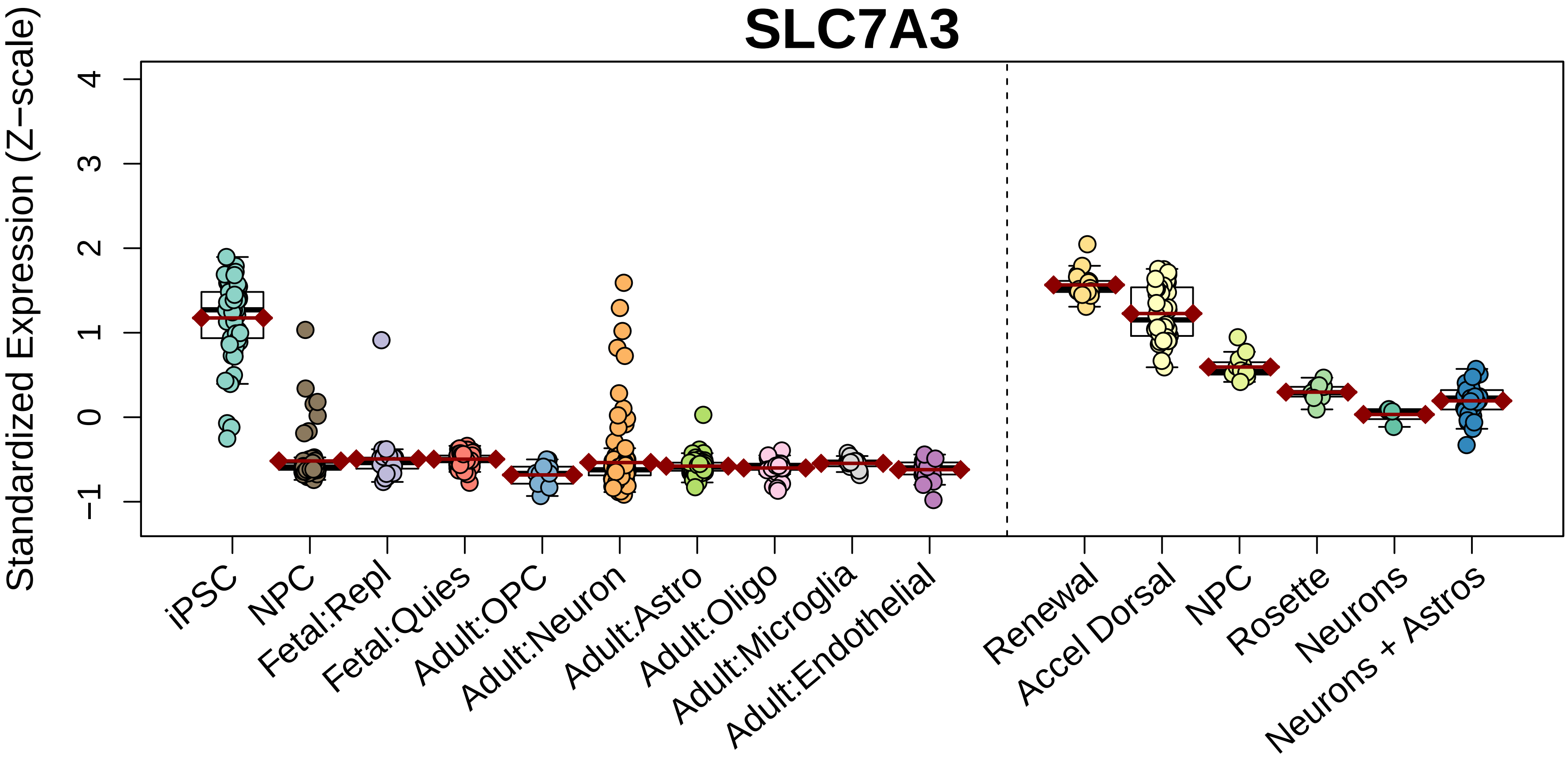
SLC7A3
Standardized Expression (Z−scale)
4
3
2
1
0
−1
iPSC
NPC
Fetal:Repl
Fetal:Quies
Adult:OPC
Adult:Neuron
Adult:Astro
Adult:Oligo
Adult:Microglia
Adult:Endothelial
Renewal
Accel Dorsal
NPC
Rosette
Neurons
Neurons + Astros

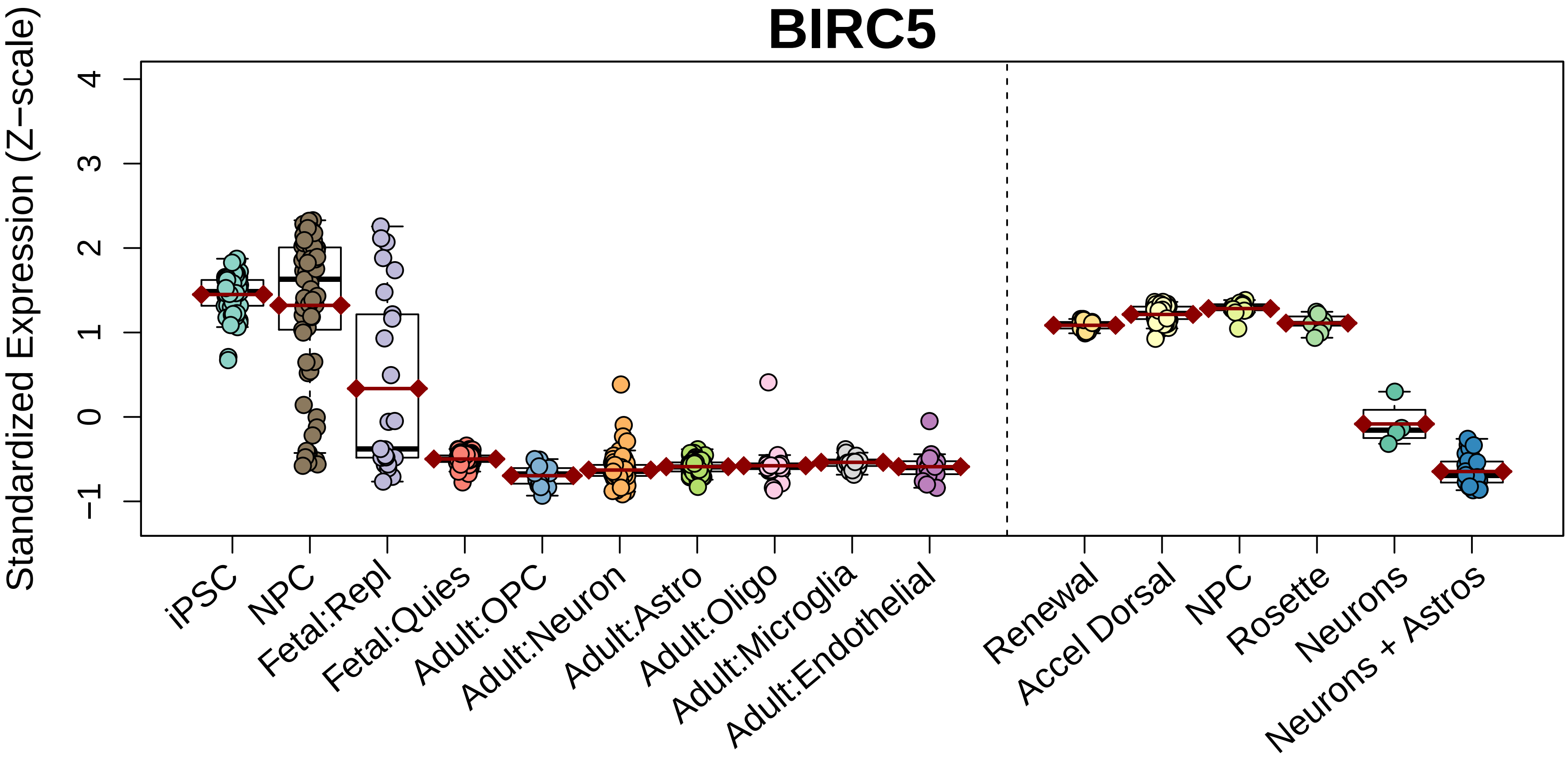

BIRC5
Standardized Expression (Z−scale)
4
3
2
1
0
−1
iPSC
NPC
Fetal:Repl
Fetal:Quies
Adult:OPC
Adult:Neuron
Adult:Astro
Adult:Oligo
Adult:Microglia
Adult:Endothelial
Renewal
Accel Dorsal
NPC
Rosette
Neurons
Neurons + Astros

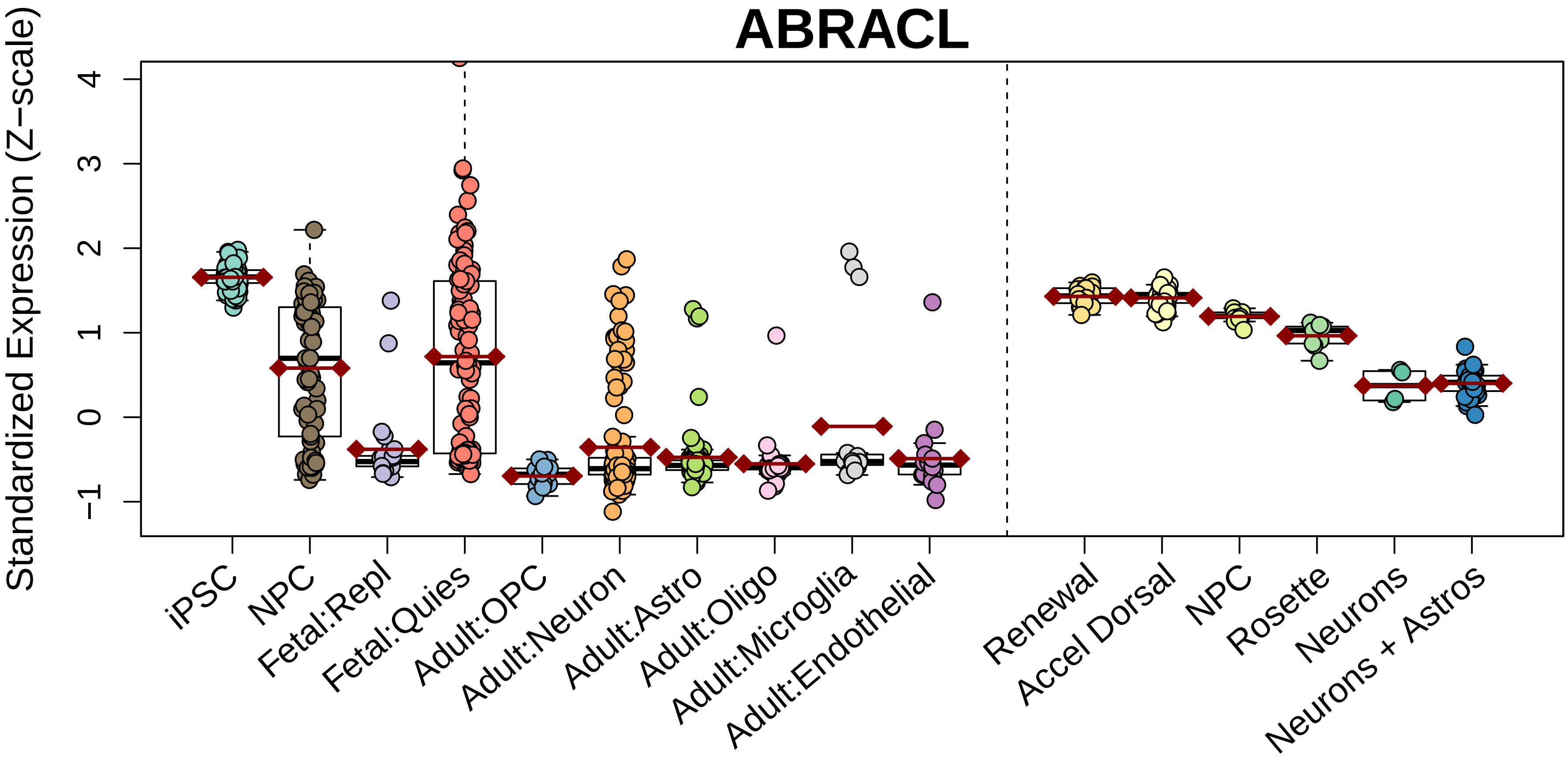

ABRACL
Standardized Expression (Z−scale)
4
3
2
1
0
−1
iPSC
NPC
Fetal:Repl
Fetal:Quies
Adult:OPC
Adult:Neuron
Adult:Astro
Adult:Oligo
Adult:Microglia
Adult:Endothelial
Renewal
Accel Dorsal
NPC
Rosette
Neurons
Neurons + Astros

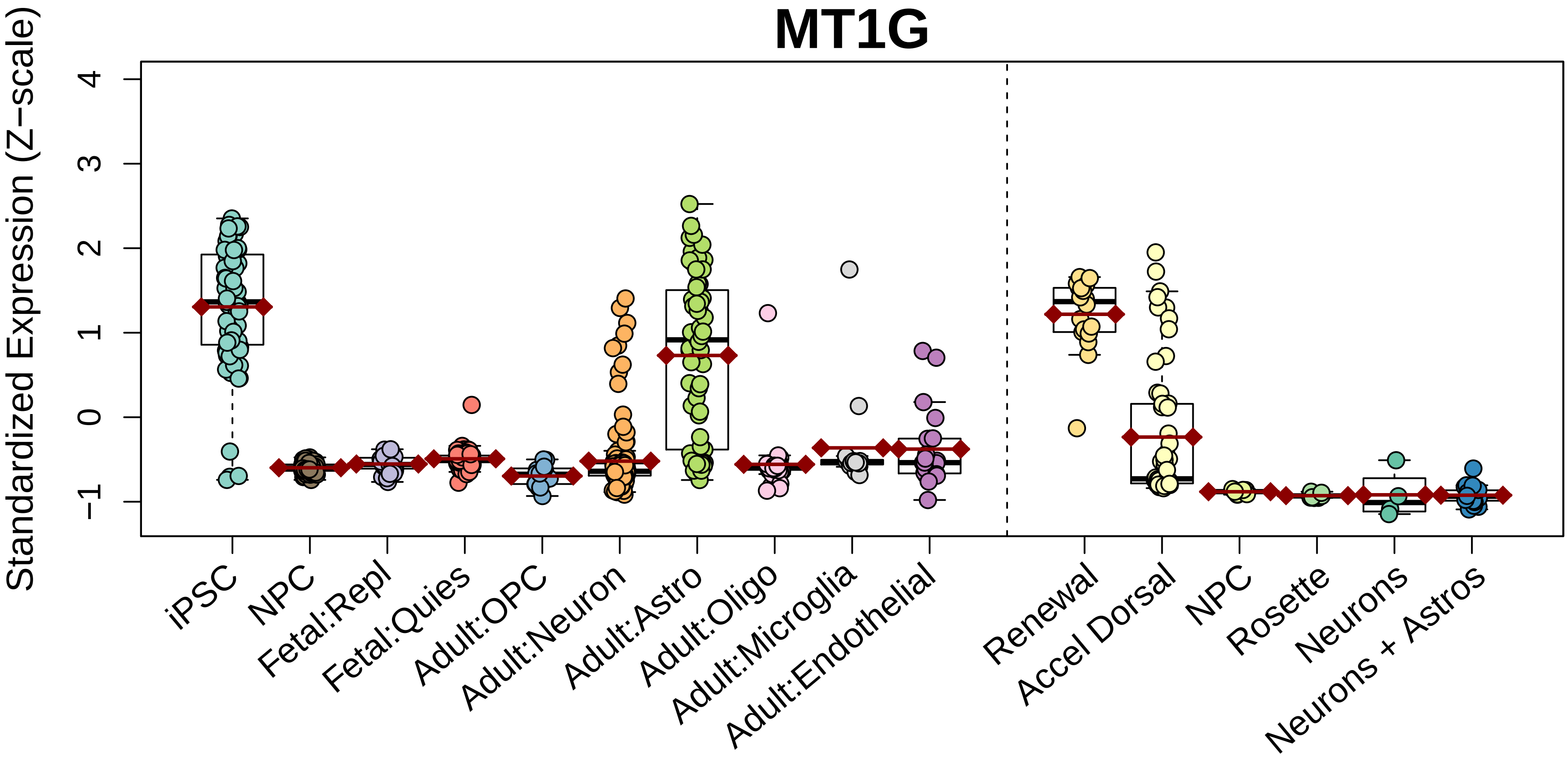
MT1G
Standardized Expression (Z−scale)
−1
0
1
2
3
4
iPSC
NPC
Fetal:Repl
Fetal:Quies
Adult:OPC
Adult:Neuron
Adult:Astro
Adult:Oligo
Adult:Microglia
Adult:Endothelial
Renewal
Accel Dorsal
NPC
Rosette
Neurons
Neurons + Astros

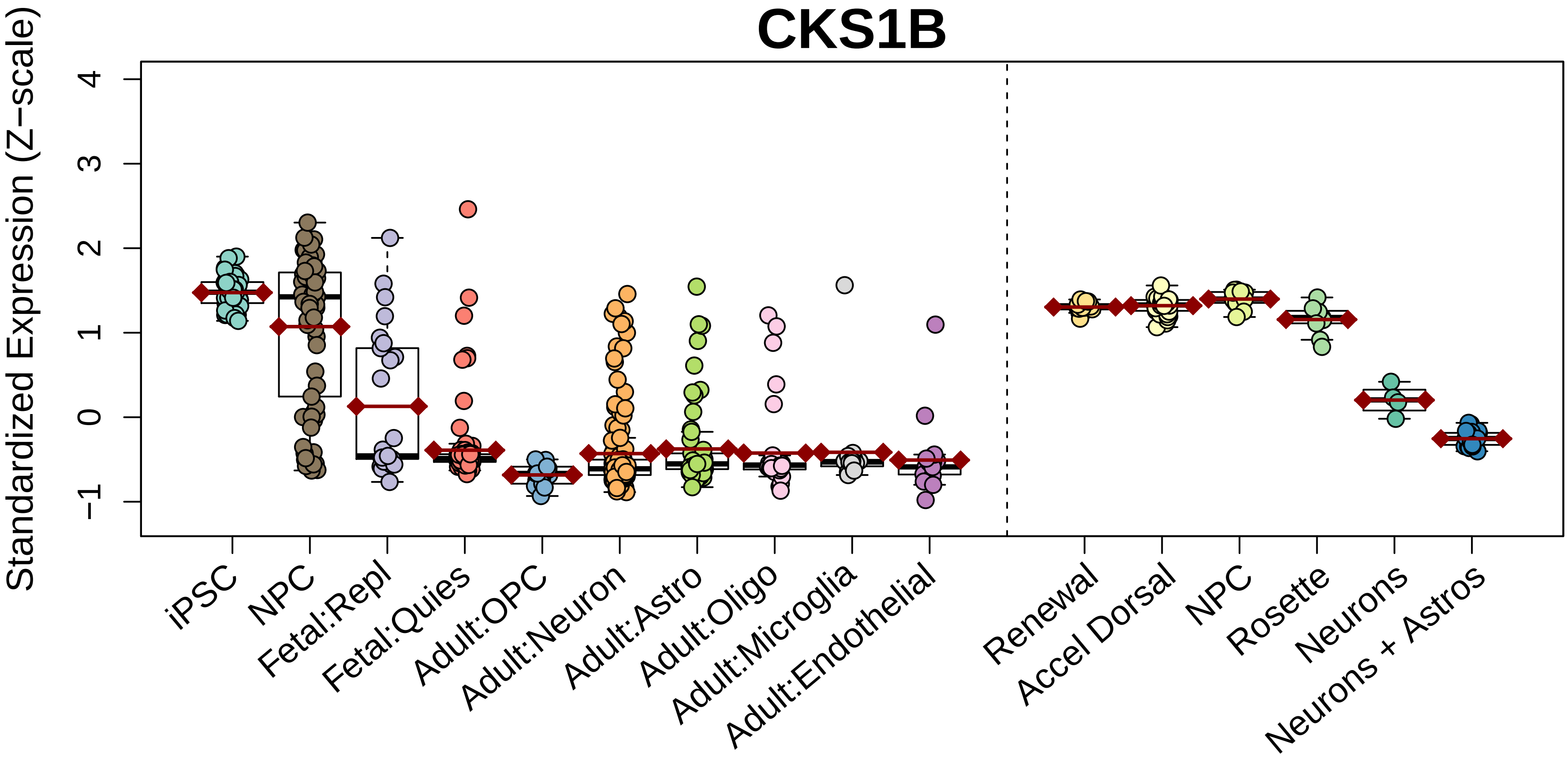

CKS1B
Standardized Expression (Z−scale)
−1
0
1
2
3
4
iPSC
NPC
Fetal:Repl
Fetal:Quies
Adult:OPC
Adult:Neuron
Adult:Astro
Adult:Oligo
Adult:Microglia
Adult:Endothelial
Renewal
Accel Dorsal
NPC
Rosette
Neurons
Neurons + Astros

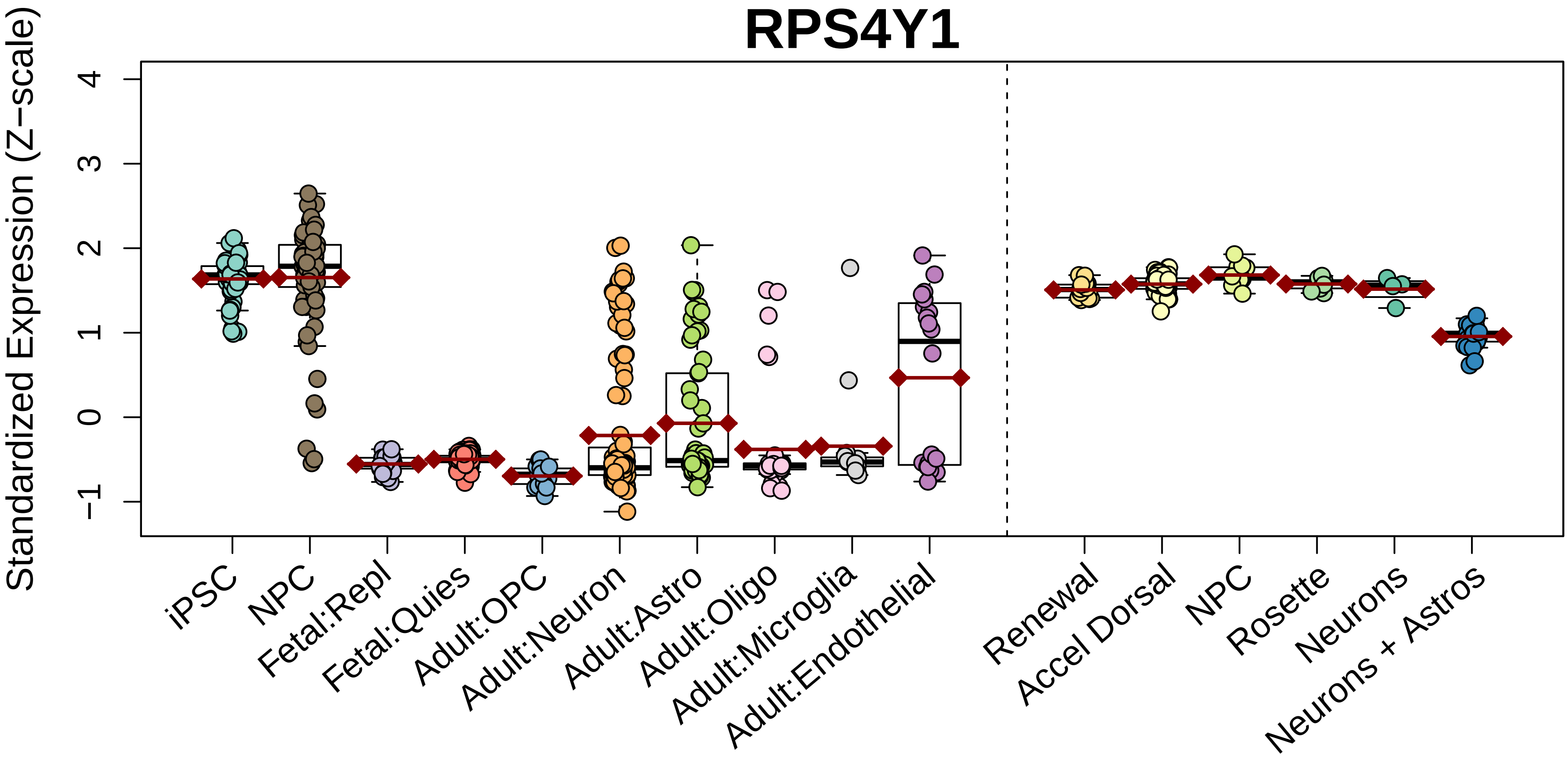
RPS4Y1
Standardized Expression (Z−scale)
−1
0
1
2
3
4
iPSC
NPC
Fetal:Repl
Fetal:Quies
Adult:OPC
Adult:Neuron
Adult:Astro
Adult:Oligo
Adult:Microglia
Adult:Endothelial
Renewal
Accel Dorsal
NPC
Rosette
Neurons
Neurons + Astros

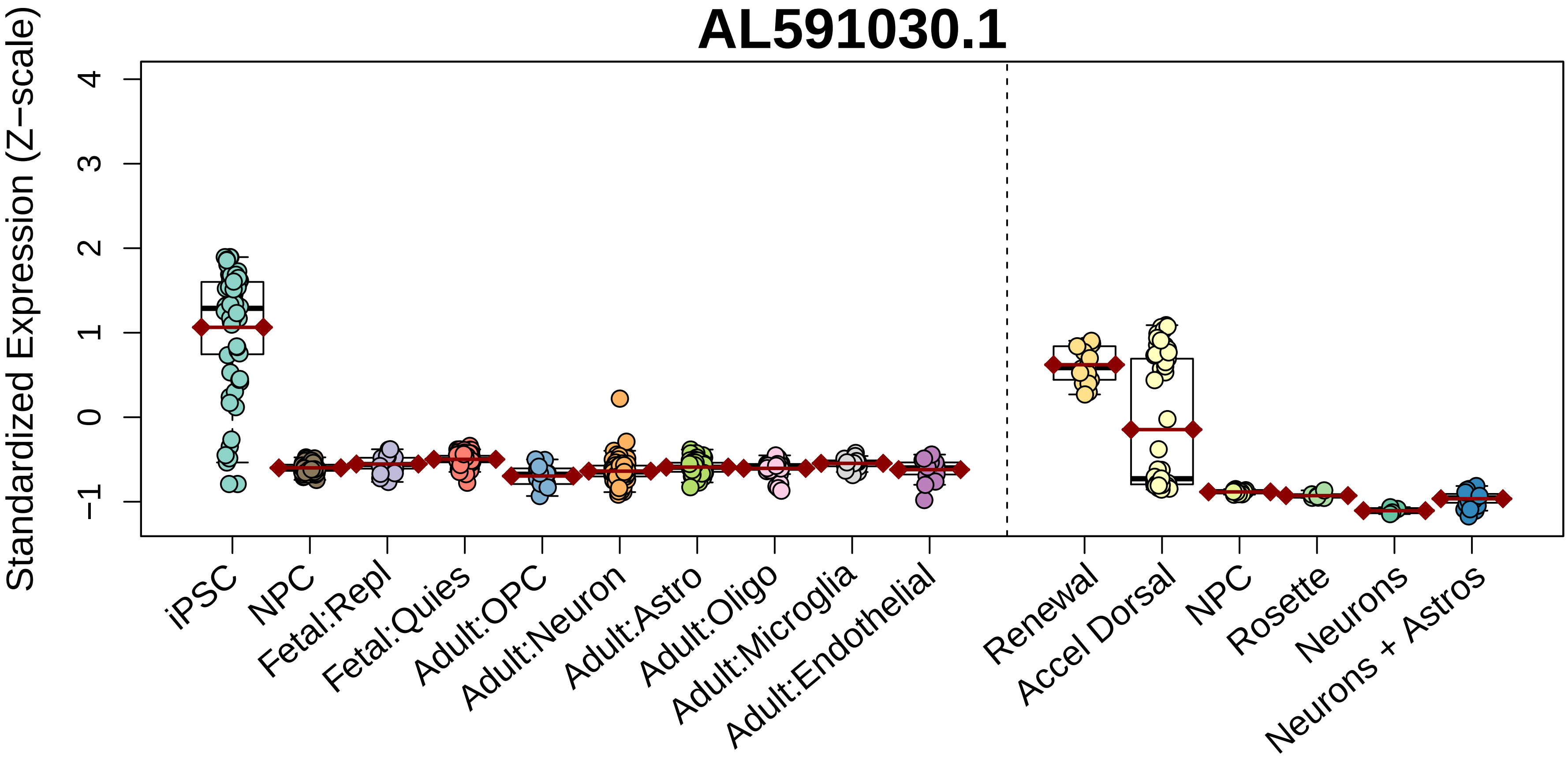

AL591030.1
Standardized Expression (Z−scale)
4
3
2
1
0
−1
iPSC
NPC
Fetal:Repl
Fetal:Quies
Adult:OPC
Adult:Neuron
Adult:Astro
Adult:Oligo
Adult:Microglia
Adult:Endothelial
Renewal
Accel Dorsal
NPC
Rosette
Neurons
Neurons + Astros

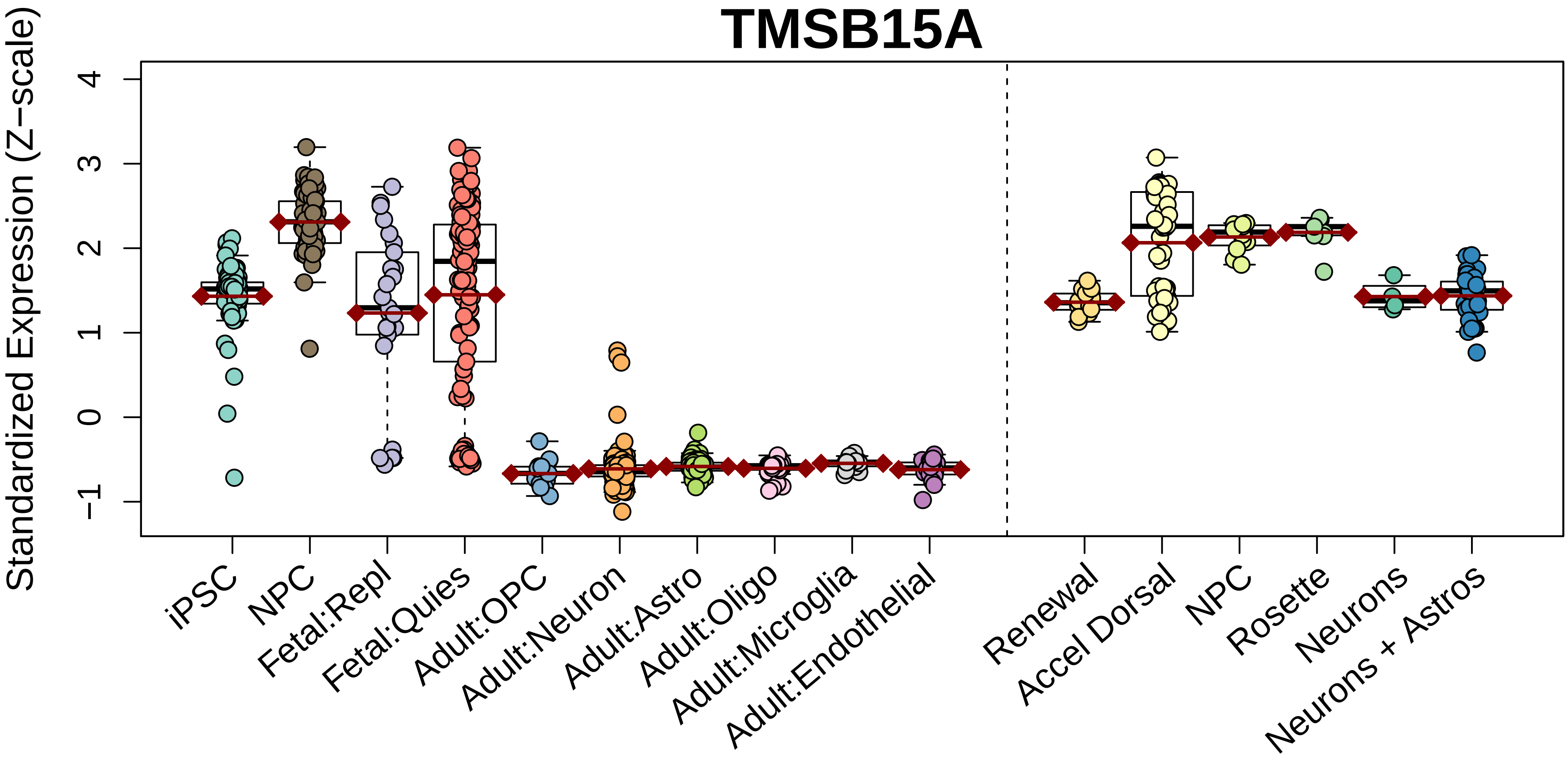

TMSB15A
Standardized Expression (Z−scale)
4
3
2
1
0
−1
iPSC
NPC
Fetal:Repl
Fetal:Quies
Adult:OPC
Adult:Neuron
Adult:Astro
Adult:Oligo
Adult:Microglia
Adult:Endothelial
Renewal
Accel Dorsal
NPC
Rosette
Neurons
Neurons + Astros

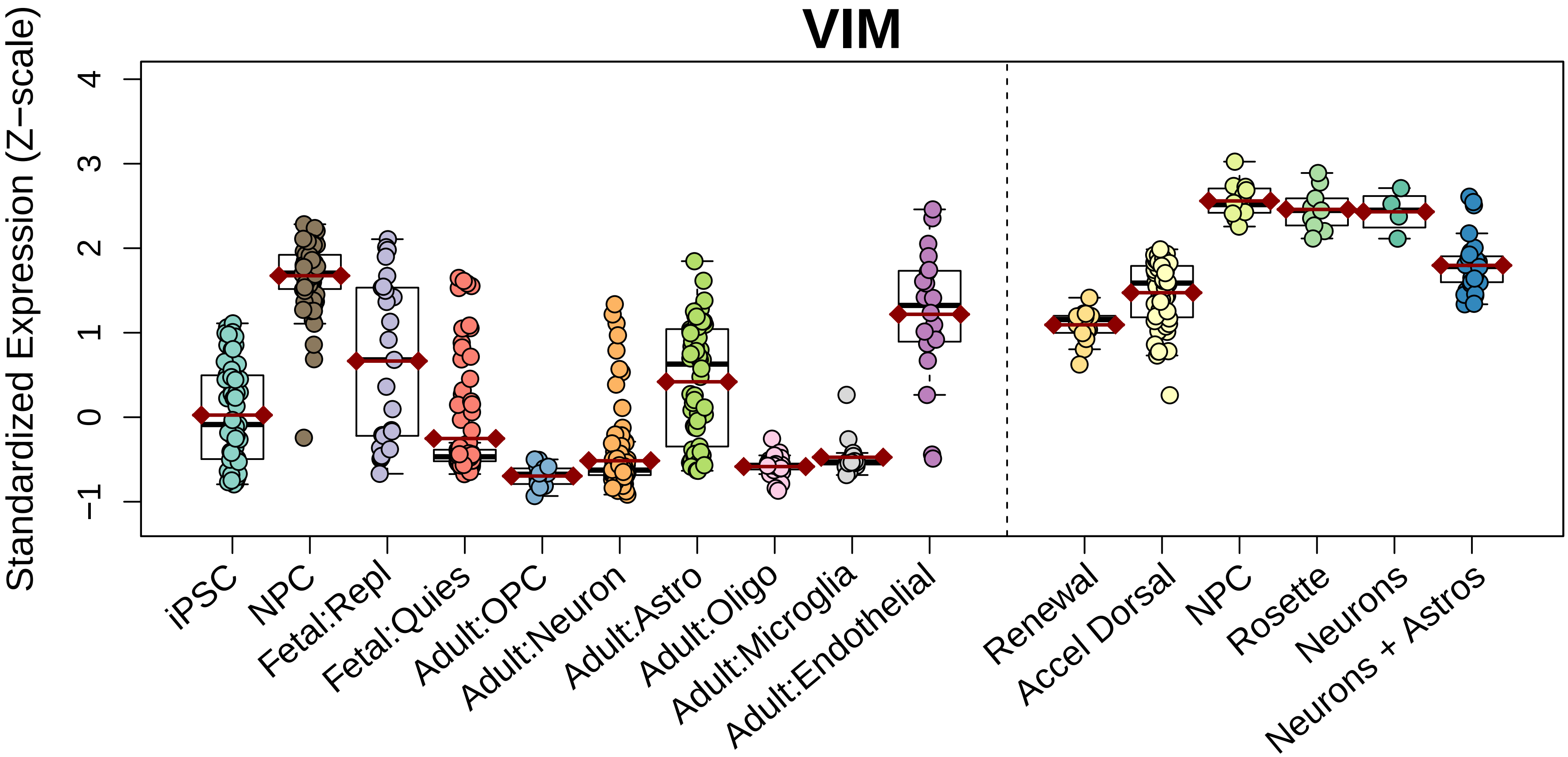

VIM
Standardized Expression (Z−scale)
4
3
2
1
0
−1
iPSC
NPC
Fetal:Repl
Fetal:Quies
Adult:OPC
Adult:Neuron
Adult:Astro
Adult:Oligo
Adult:Microglia
Adult:Endothelial
Renewal
Accel Dorsal
NPC
Rosette
Neurons
Neurons + Astros

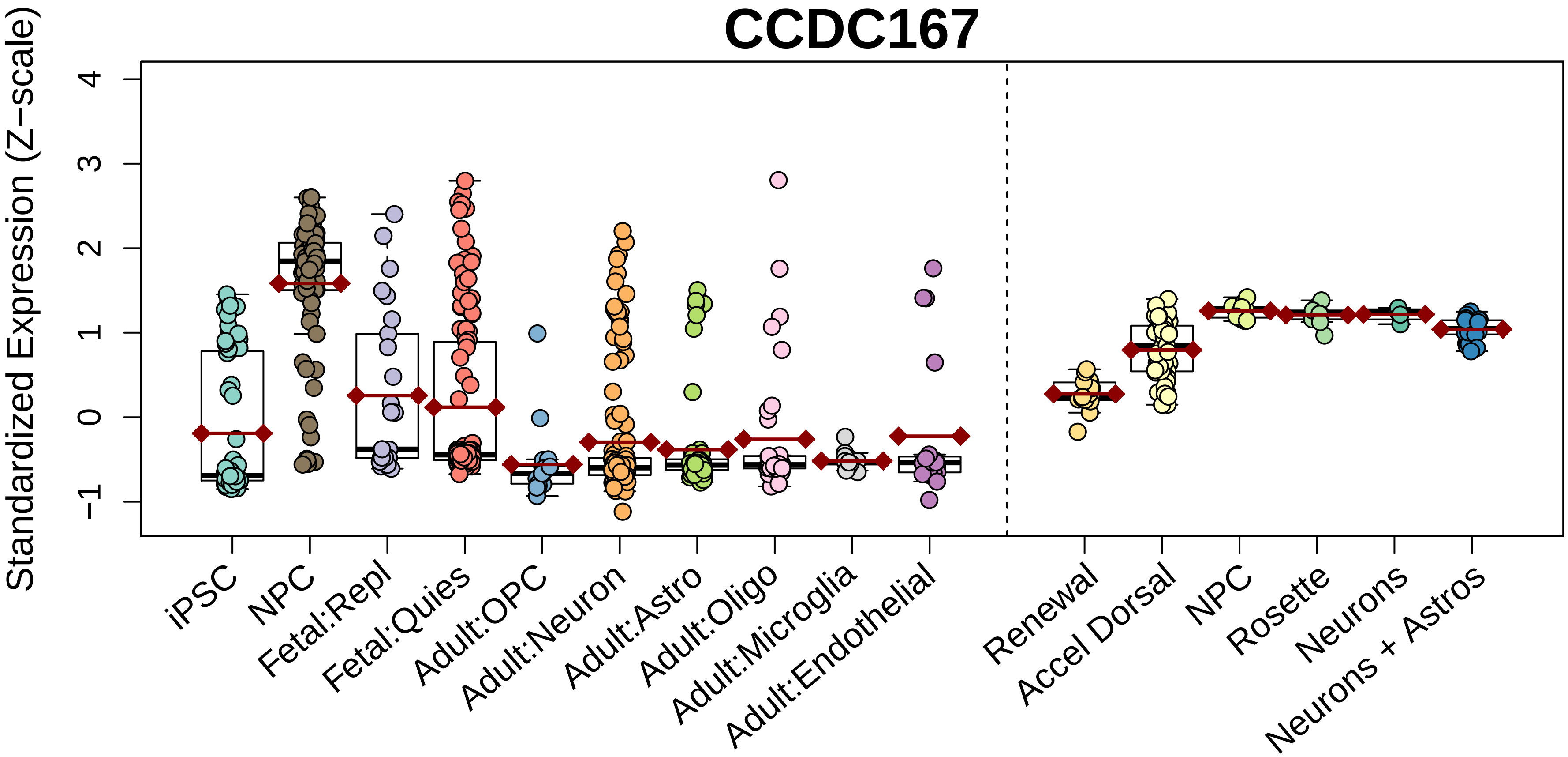

CCDC167
Standardized Expression (Z−scale)
4
3
2
1
0
−1
iPSC
NPC
Fetal:Repl
Fetal:Quies
Adult:OPC
Adult:Neuron
Adult:Astro
Adult:Oligo
Adult:Microglia
Adult:Endothelial
Renewal
Accel Dorsal
NPC
Rosette
Neurons
Neurons + Astros

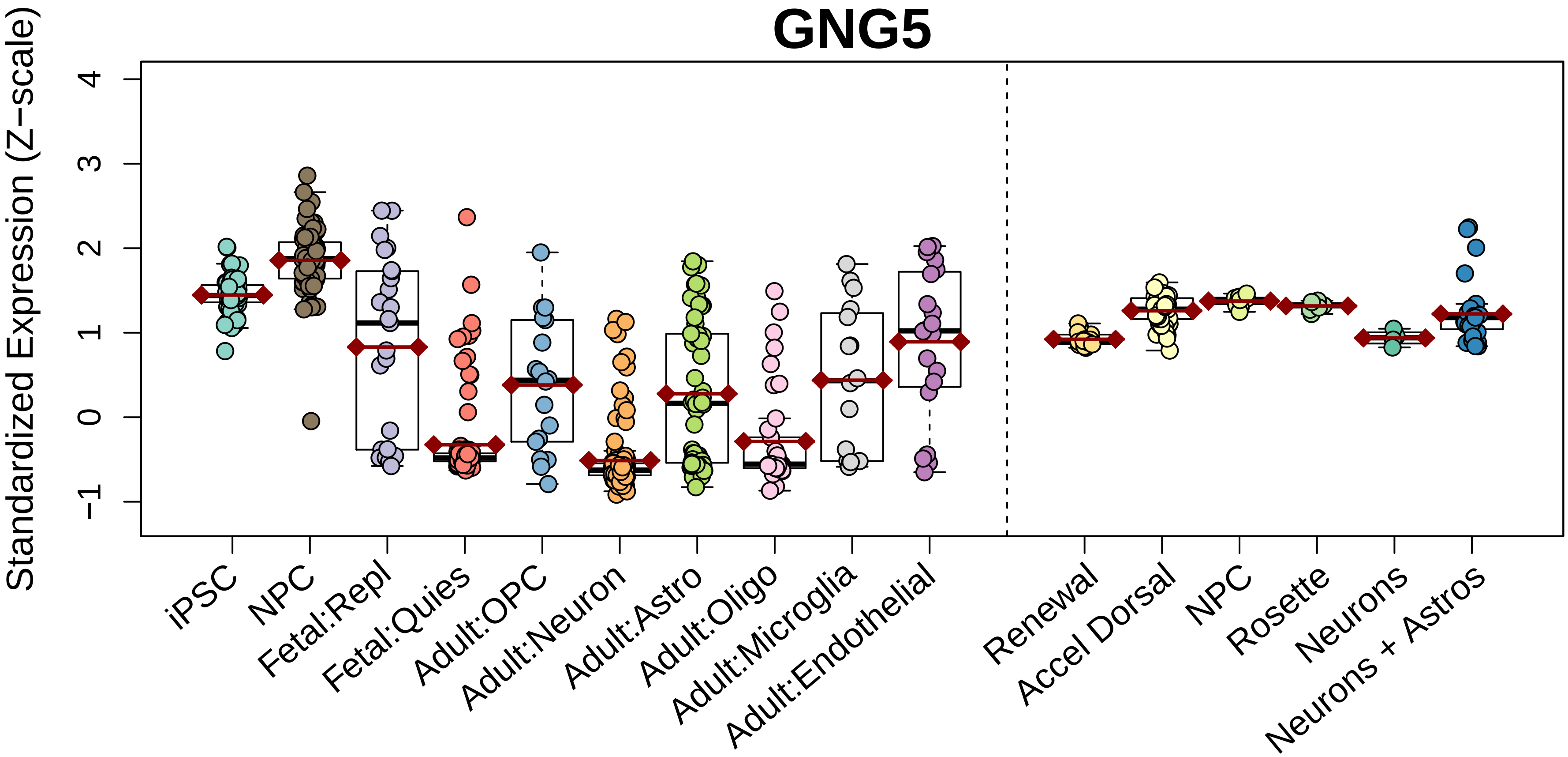

GNG5
Standardized Expression (Z−scale)
4
3
2
1
0
−1
iPSC
NPC
Fetal:Repl
Fetal:Quies
Adult:OPC
Adult:Neuron
Adult:Astro
Adult:Oligo
Adult:Microglia
Adult:Endothelial
Renewal
Accel Dorsal
NPC
Rosette
Neurons
Neurons + Astros

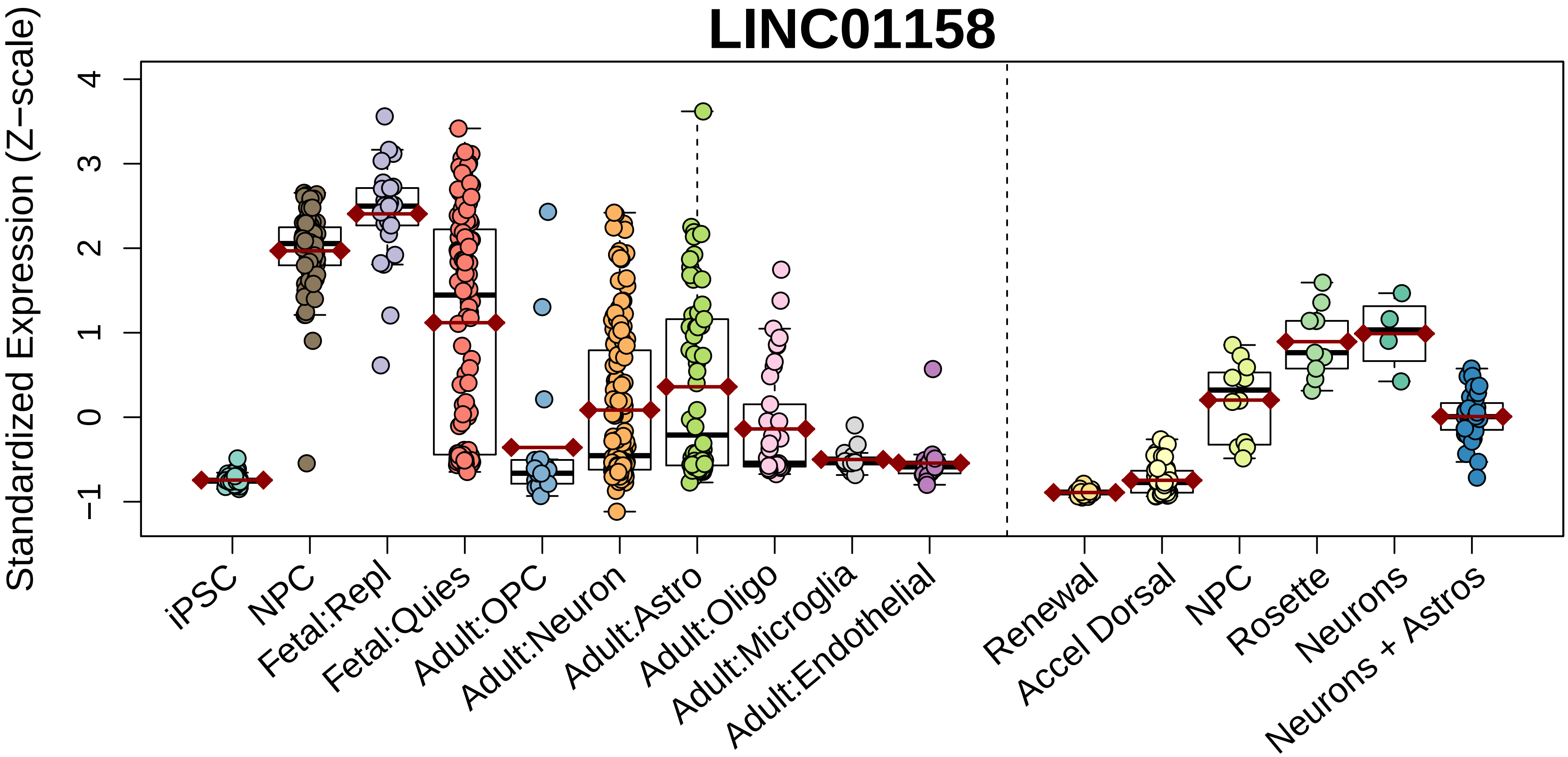

LINC01158
Standardized Expression (Z−scale)
4
3
2
1
0
−1
iPSC
NPC
Fetal:Repl
Fetal:Quies
Adult:OPC
Adult:Neuron
Adult:Astro
Adult:Oligo
Adult:Microglia
Adult:Endothelial
Renewal
Accel Dorsal
NPC
Rosette
Neurons
Neurons + Astros

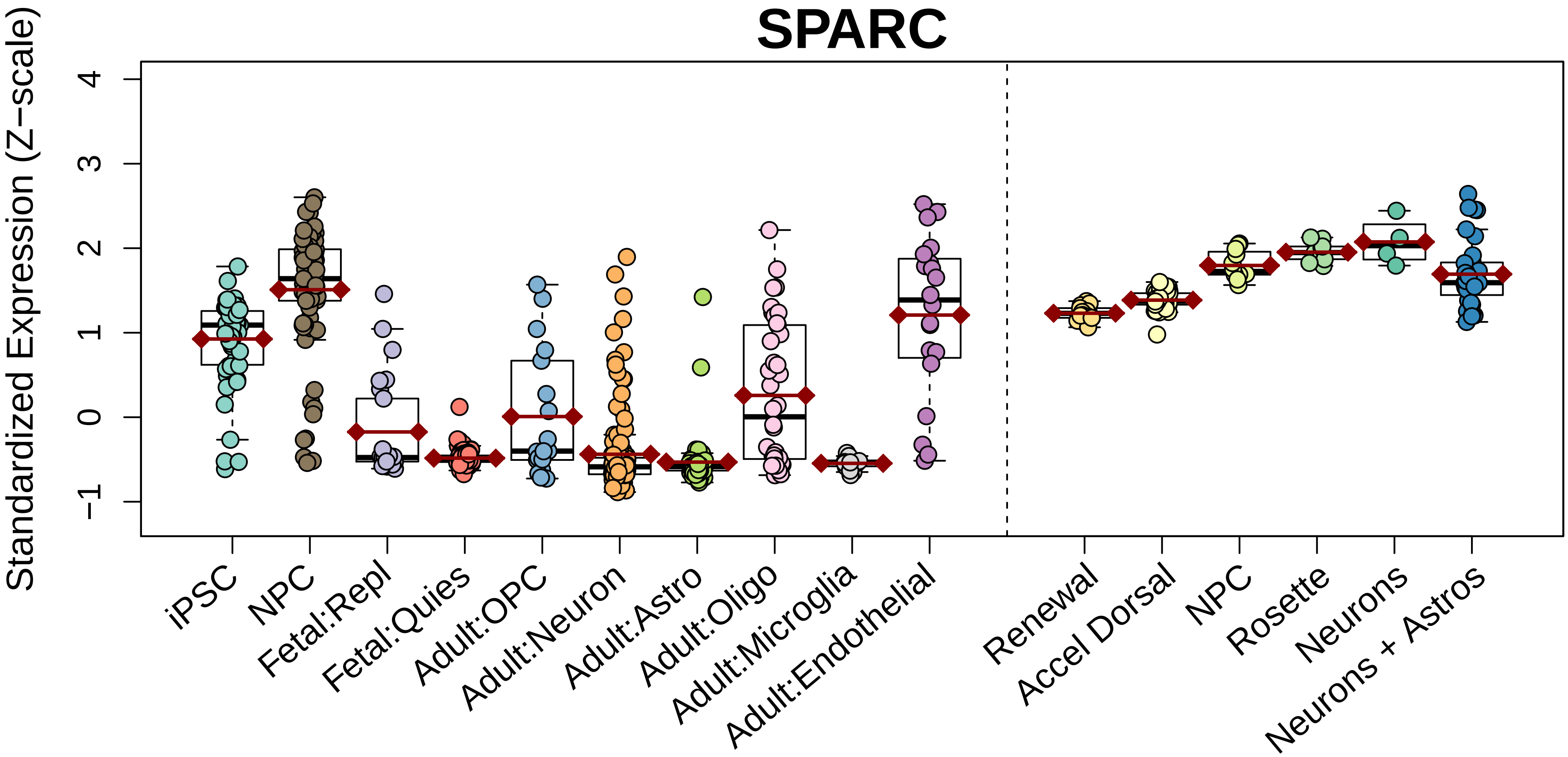
SPARC
Standardized Expression (Z−scale)
4
3
2
1
0
−1
iPSC
NPC
Fetal:Repl
Fetal:Quies
Adult:OPC
Adult:Neuron
Adult:Astro
Adult:Oligo
Adult:Microglia
Adult:Endothelial
Renewal
Accel Dorsal
NPC
Rosette
Neurons
Neurons + Astros

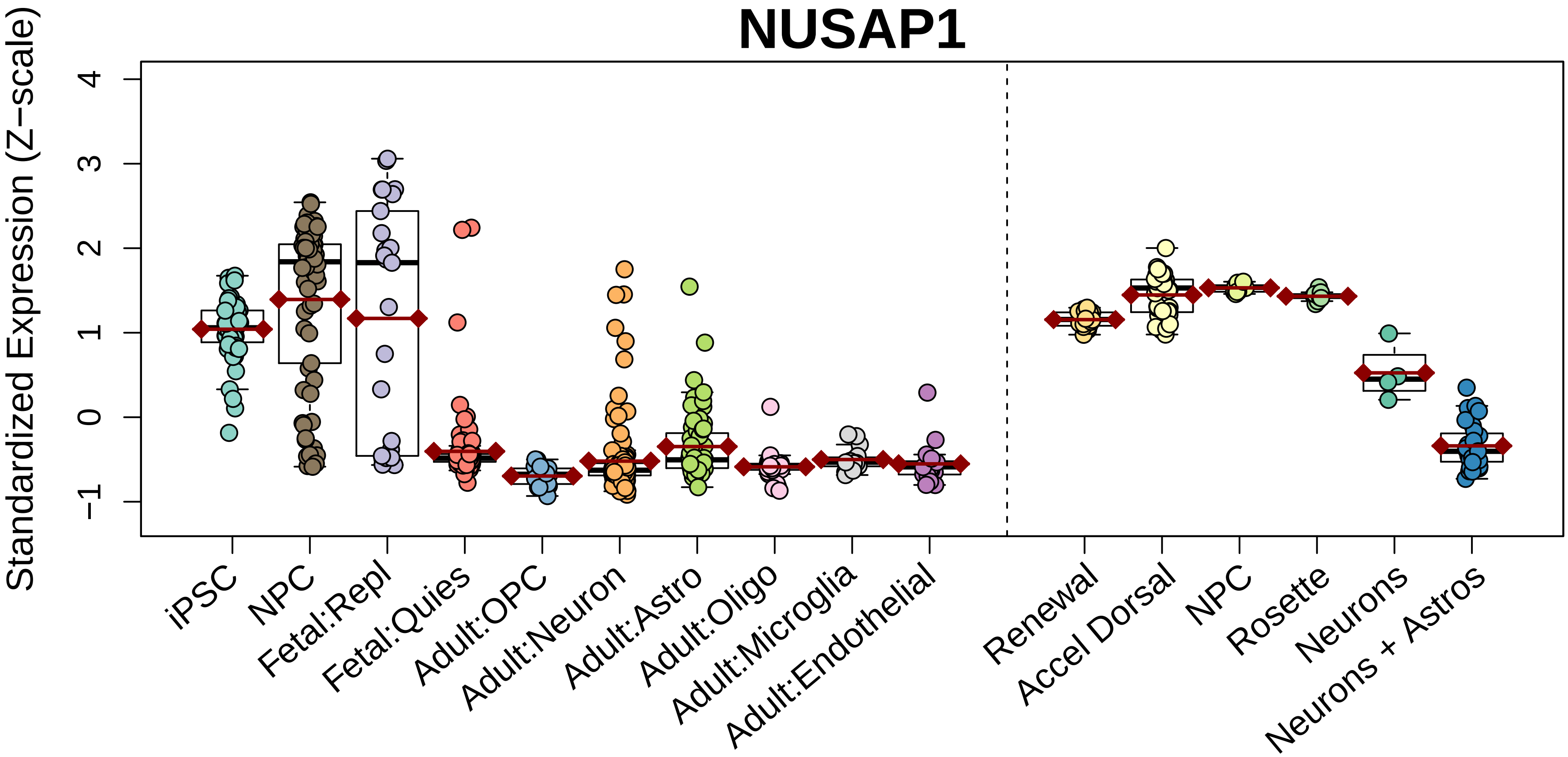

NUSAP1
Standardized Expression (Z−scale)
4
3
2
1
0
−1
iPSC
NPC
Fetal:Repl
Fetal:Quies
Adult:OPC
Adult:Neuron
Adult:Astro
Adult:Oligo
Adult:Microglia
Adult:Endothelial
Renewal
Accel Dorsal
NPC
Rosette
Neurons
Neurons + Astros

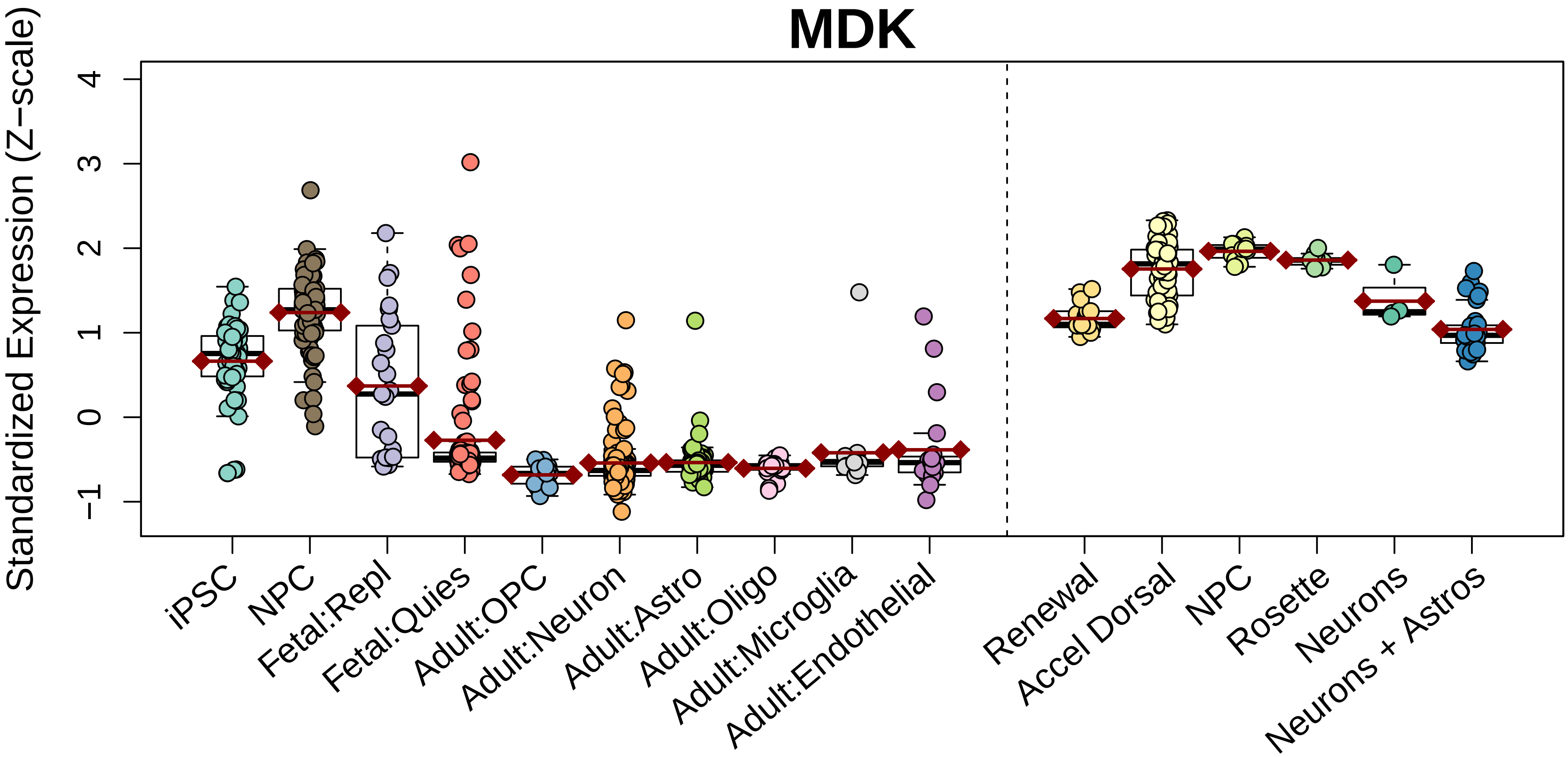
MDK
Standardized Expression (Z−scale)
4
3
2
1
0
−1
iPSC
NPC
Fetal:Repl
Fetal:Quies
Adult:OPC
Adult:Neuron
Adult:Astro
Adult:Oligo
Adult:Microglia
Adult:Endothelial
Renewal
Accel Dorsal
NPC
Rosette
Neurons
Neurons + Astros

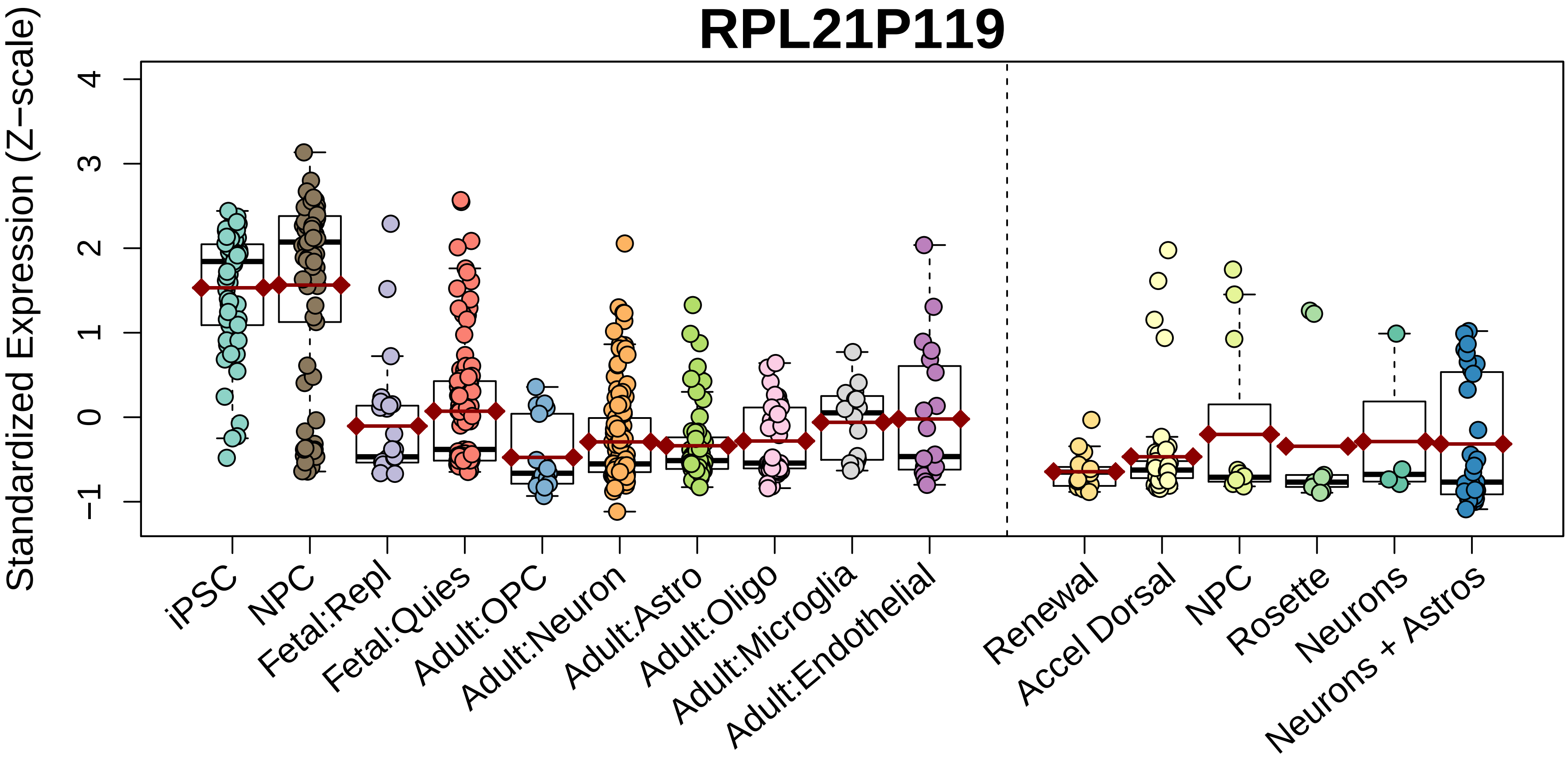
RPL21P119
Standardized Expression (Z−scale)
4
3
2
1
0
−1
iPSC
NPC
Fetal:Repl
Fetal:Quies
Adult:OPC
Adult:Neuron
Adult:Astro
Adult:Oligo
Adult:Microglia
Adult:Endothelial
Renewal
Accel Dorsal
NPC
Rosette
Neurons
Neurons + Astros

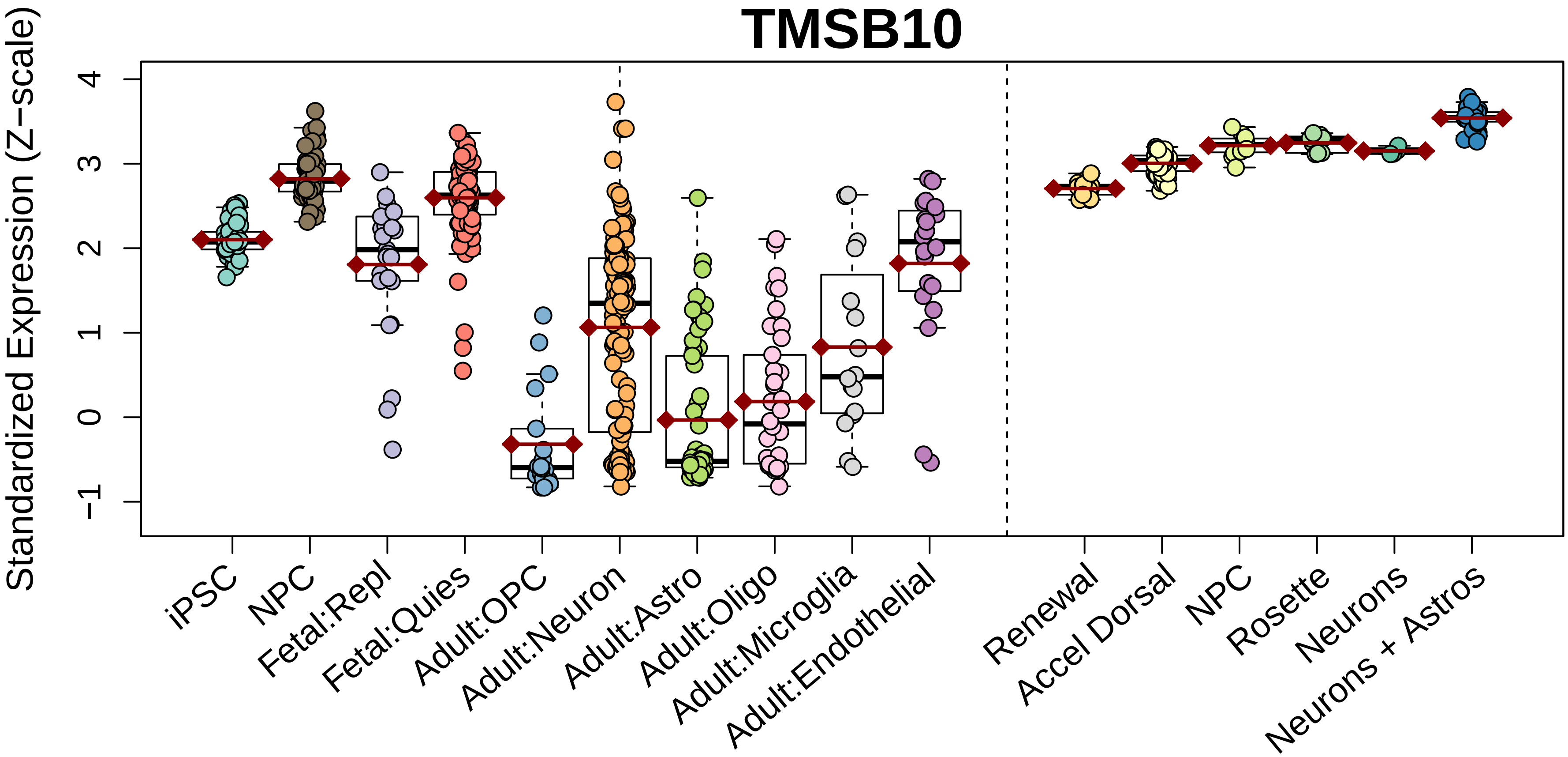

TMSB10
Standardized Expression (Z−scale)
4
3
2
1
0
−1
iPSC
NPC
Fetal:Repl
Fetal:Quies
Adult:OPC
Adult:Neuron
Adult:Astro
Adult:Oligo
Adult:Microglia
Adult:Endothelial
Renewal
Accel Dorsal
NPC
Rosette
Neurons
Neurons + Astros

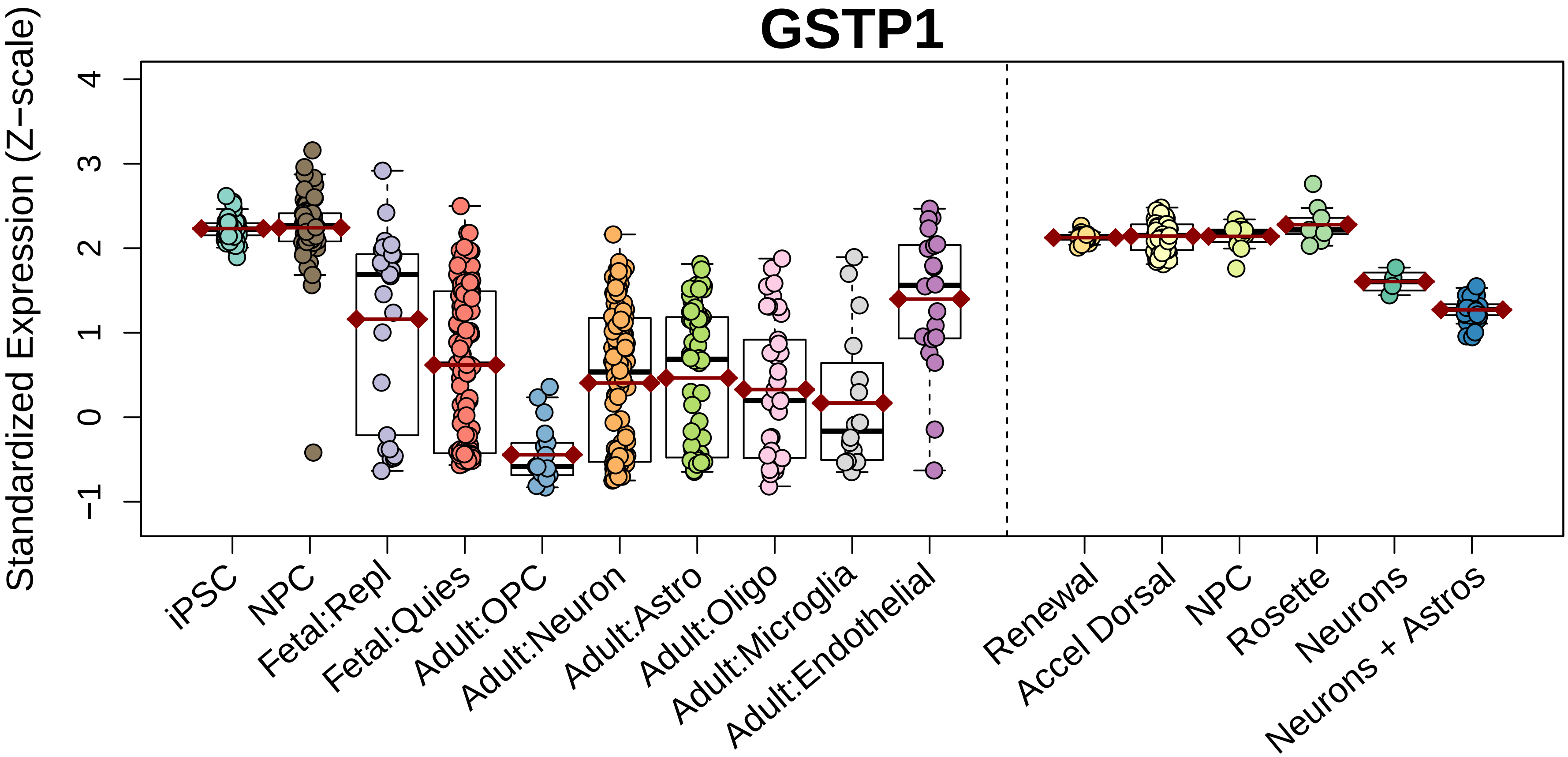
GSTP1
Standardized Expression (Z−scale)
4
3
2
1
0
−1
iPSC
NPC
Fetal:Repl
Fetal:Quies
Adult:OPC
Adult:Neuron
Adult:Astro
Adult:Oligo
Adult:Microglia
Adult:Endothelial
Renewal
Accel Dorsal
NPC
Rosette
Neurons
Neurons + Astros

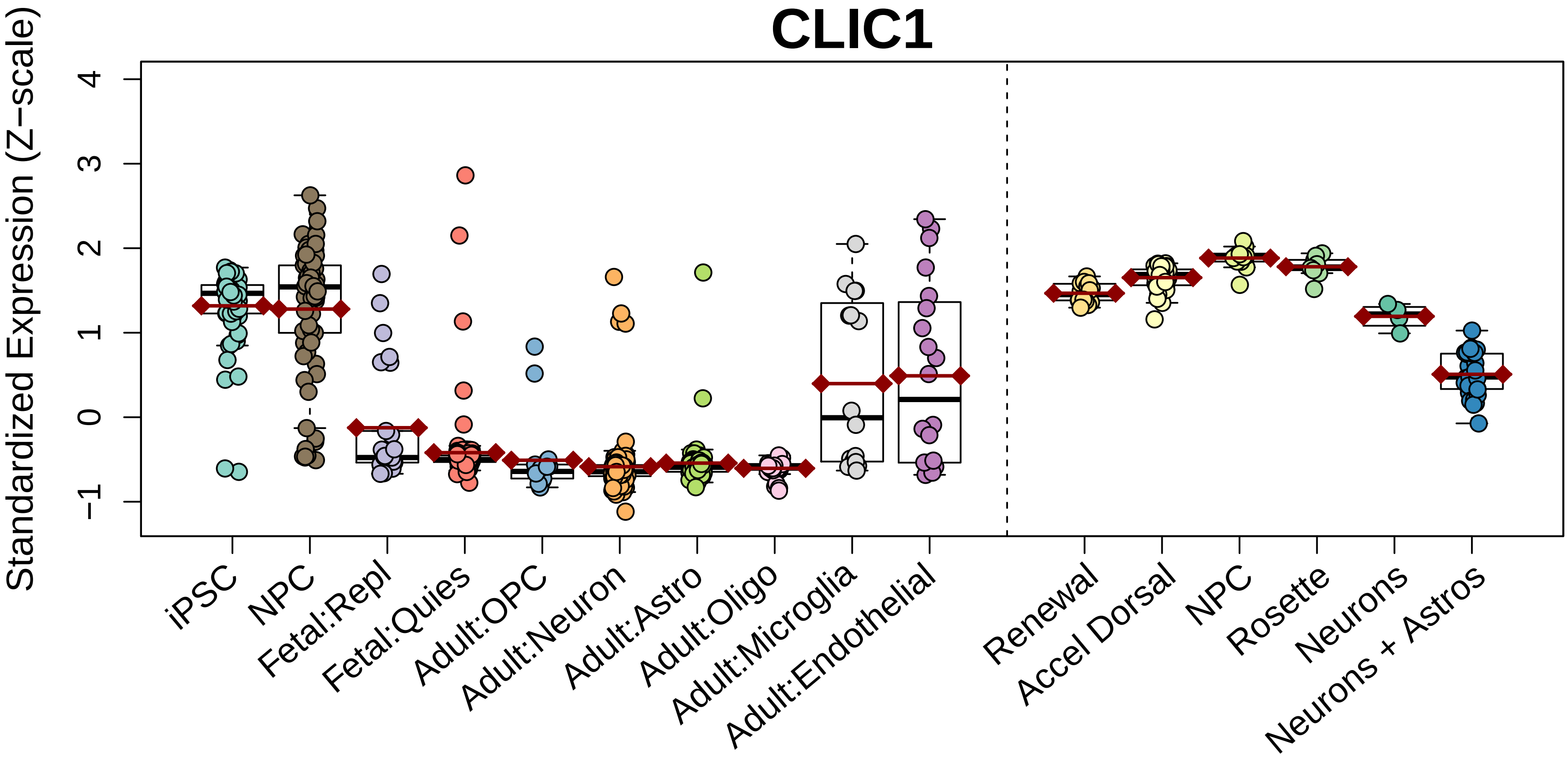

CLIC1
Standardized Expression (Z−scale)
4
3
2
1
0
−1
iPSC
NPC
Fetal:Repl
Fetal:Quies
Adult:OPC
Adult:Neuron
Adult:Astro
Adult:Oligo
Adult:Microglia
Adult:Endothelial
Renewal
Accel Dorsal
NPC
Rosette
Neurons
Neurons + Astros

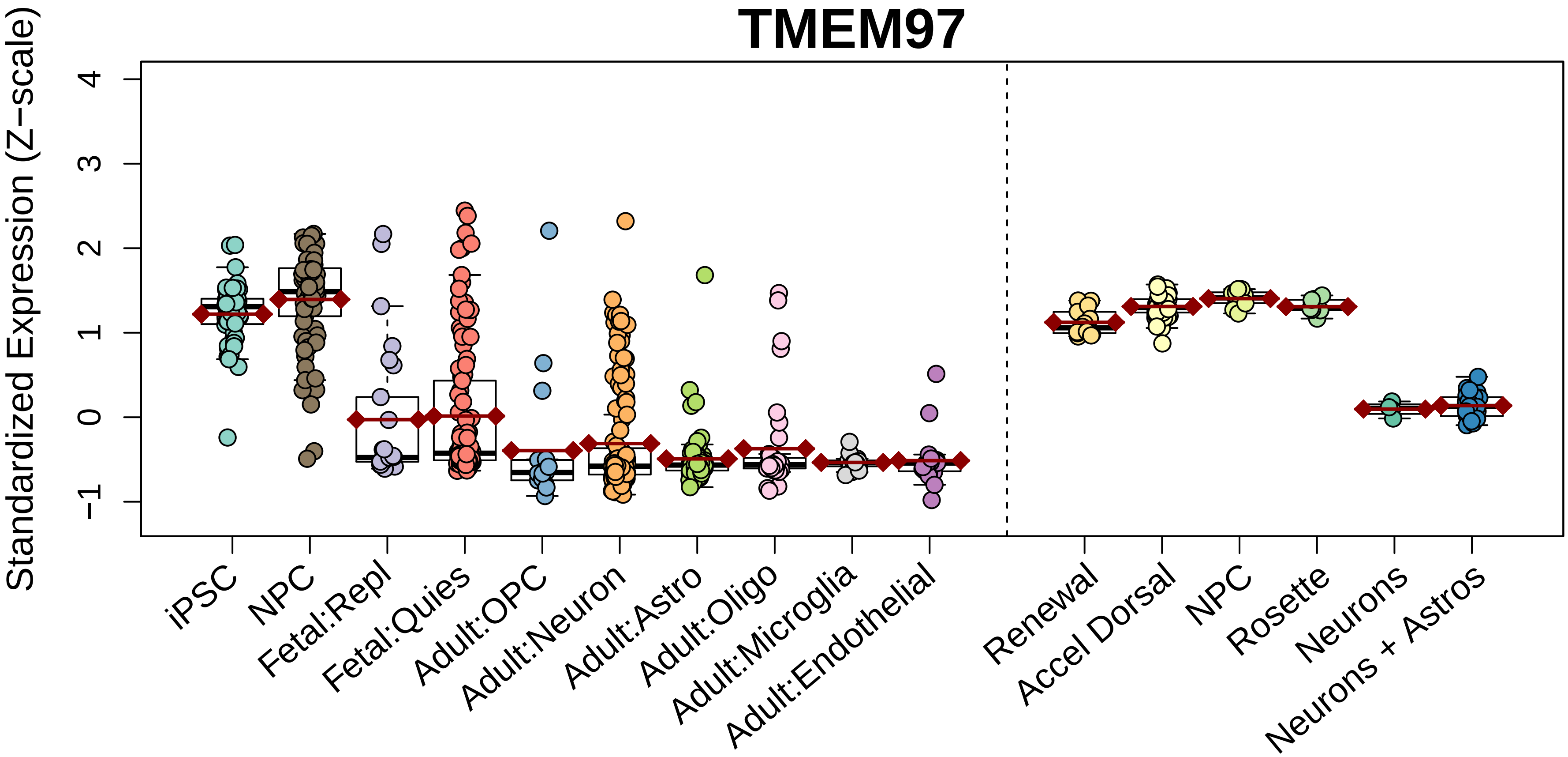

TMEM97
Standardized Expression (Z−scale)
4
3
2
1
0
−1
iPSC
NPC
Fetal:Repl
Fetal:Quies
Adult:OPC
Adult:Neuron
Adult:Astro
Adult:Oligo
Adult:Microglia
Adult:Endothelial
Renewal
Accel Dorsal
NPC
Rosette
Neurons
Neurons + Astros

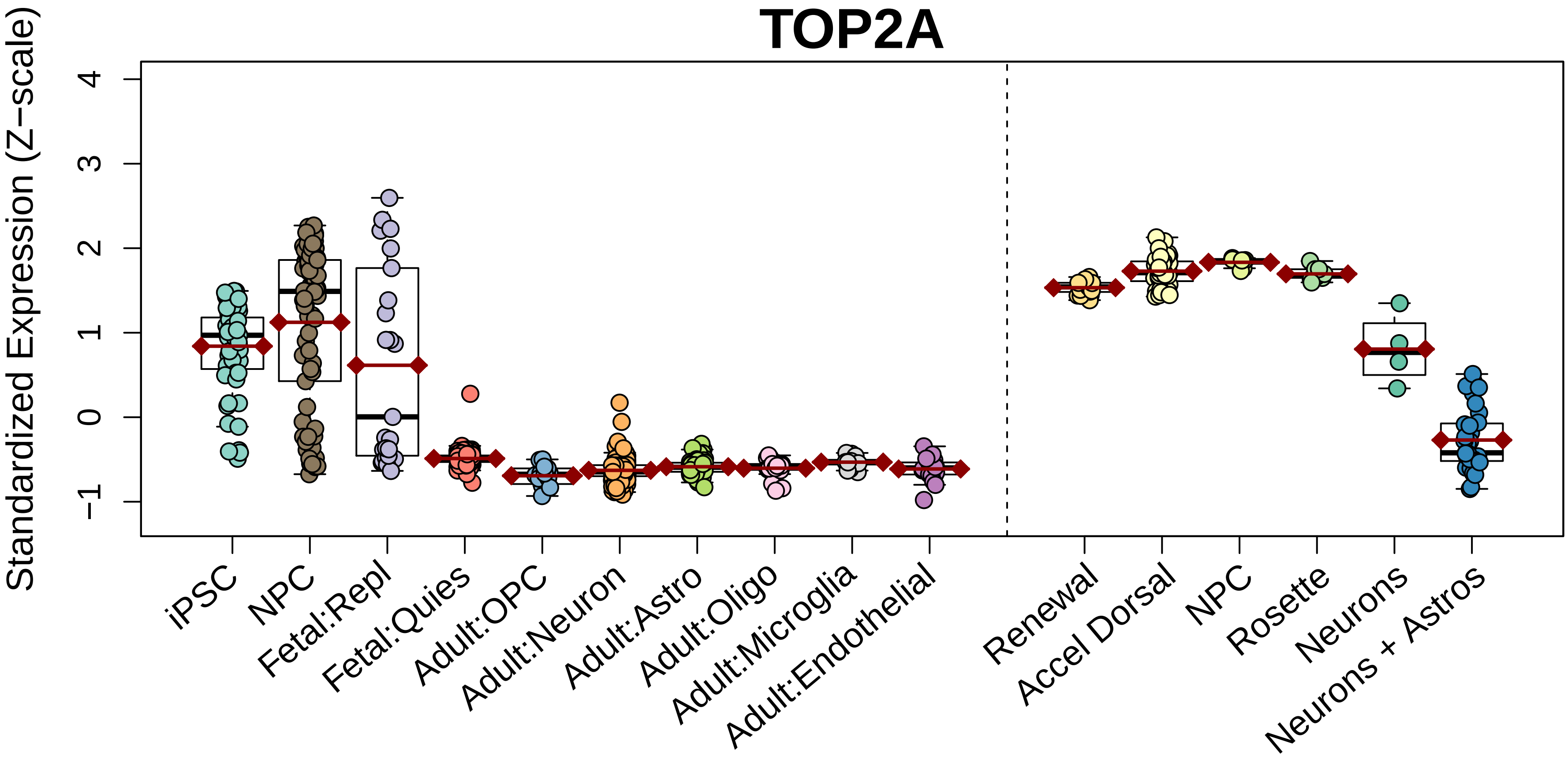
TOP2A
Standardized Expression (Z−scale)
4
3
2
1
0
−1
iPSC
NPC
Fetal:Repl
Fetal:Quies
Adult:OPC
Adult:Neuron
Adult:Astro
Adult:Oligo
Adult:Microglia
Adult:Endothelial
Renewal
Accel Dorsal
NPC
Rosette
Neurons
Neurons + Astros

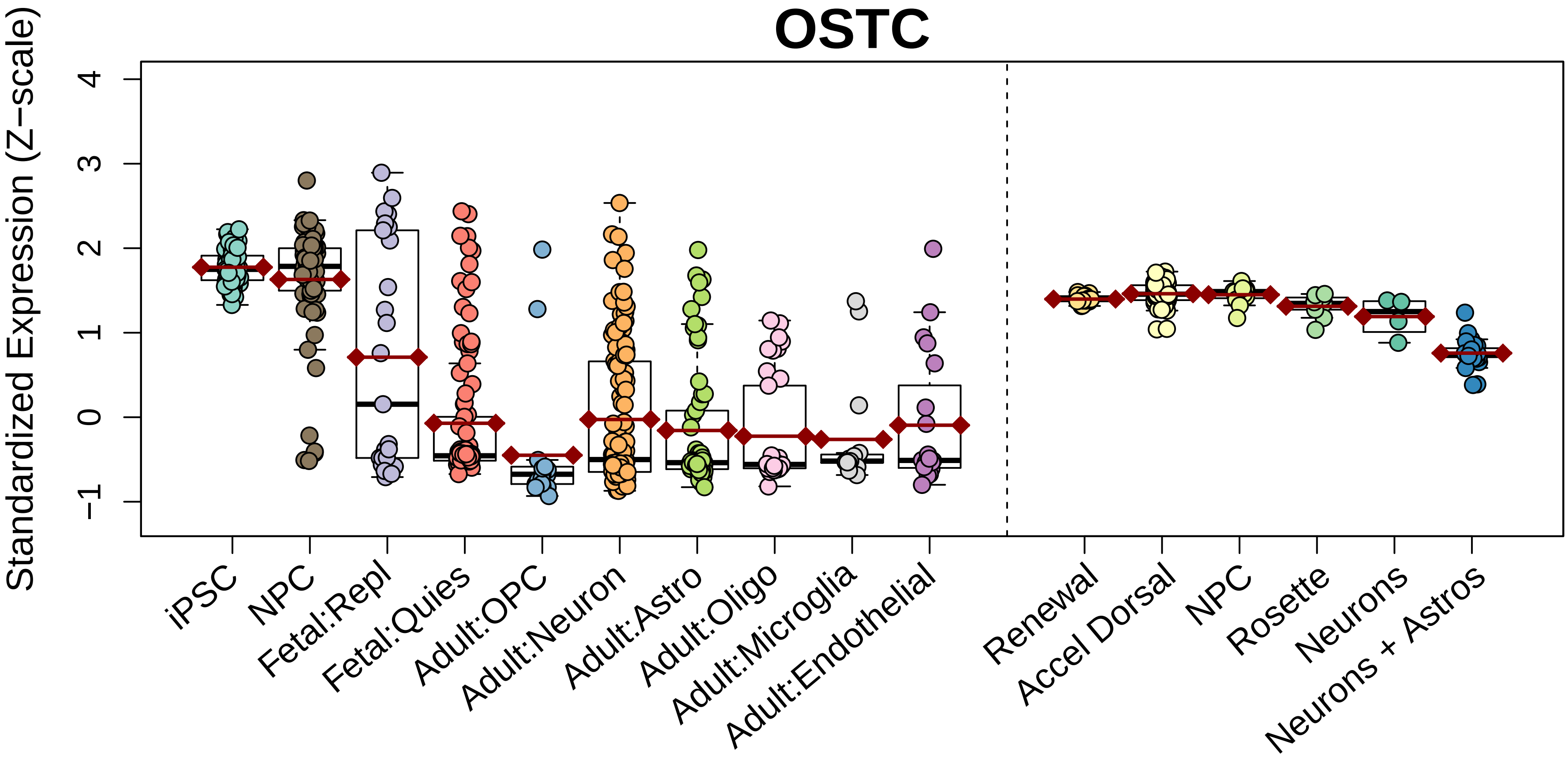

OSTC
Standardized Expression (Z−scale)
4
3
2
1
0
−1
iPSC
NPC
Fetal:Repl
Fetal:Quies
Adult:OPC
Adult:Neuron
Adult:Astro
Adult:Oligo
Adult:Microglia
Adult:Endothelial
Renewal
Accel Dorsal
NPC
Rosette
Neurons
Neurons + Astros

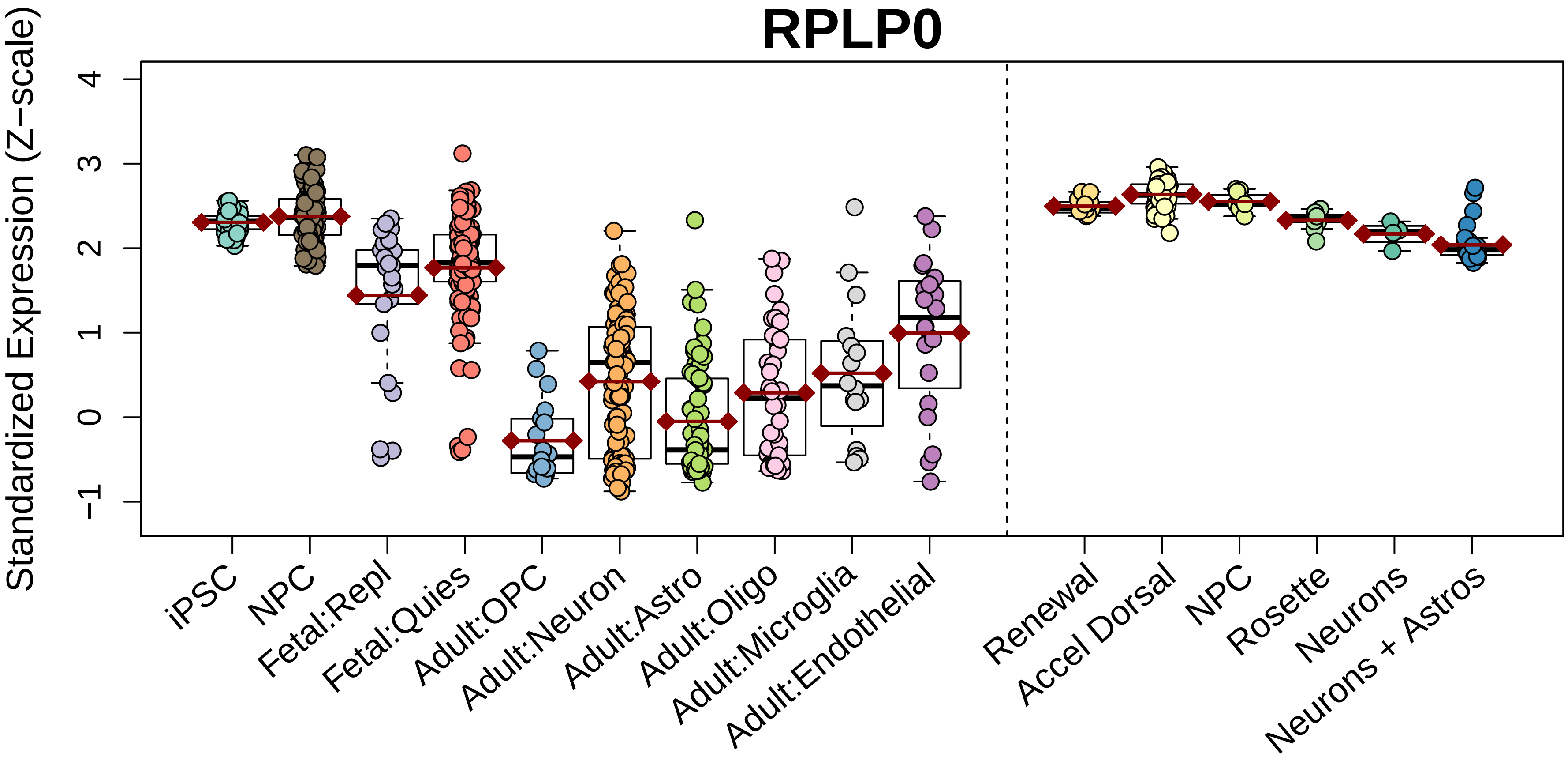

RPLP0
Standardized Expression (Z−scale)
4
3
2
1
0
−1
iPSC
NPC
Fetal:Repl
Fetal:Quies
Adult:OPC
Adult:Neuron
Adult:Astro
Adult:Oligo
Adult:Microglia
Adult:Endothelial
Renewal
Accel Dorsal
NPC
Rosette
Neurons
Neurons + Astros

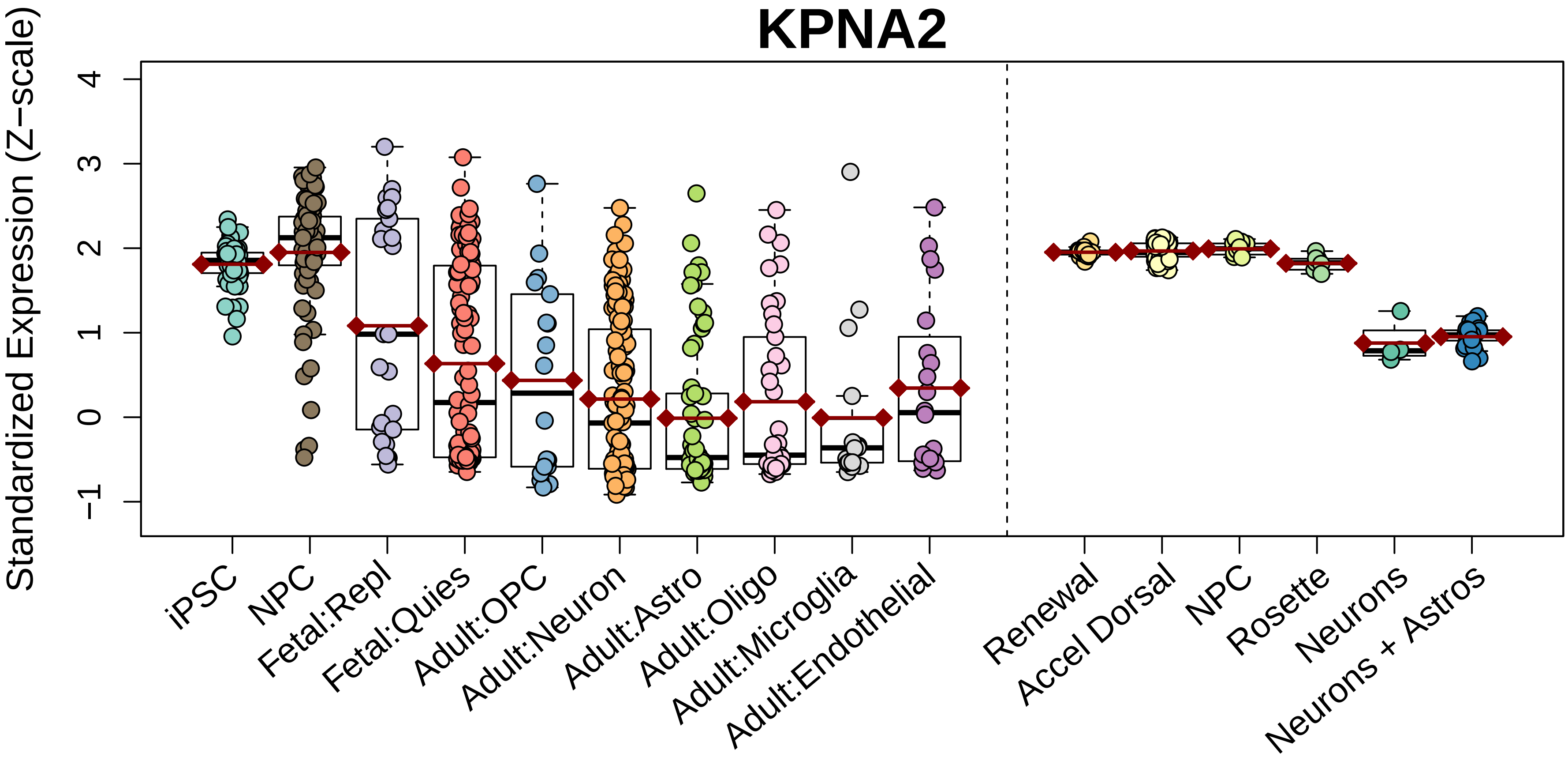

KPNA2
Standardized Expression (Z−scale)
4
3
2
1
0
−1
iPSC
NPC
Fetal:Repl
Fetal:Quies
Adult:OPC
Adult:Neuron
Adult:Astro
Adult:Oligo
Adult:Microglia
Adult:Endothelial
Renewal
Accel Dorsal
NPC
Rosette
Neurons
Neurons + Astros

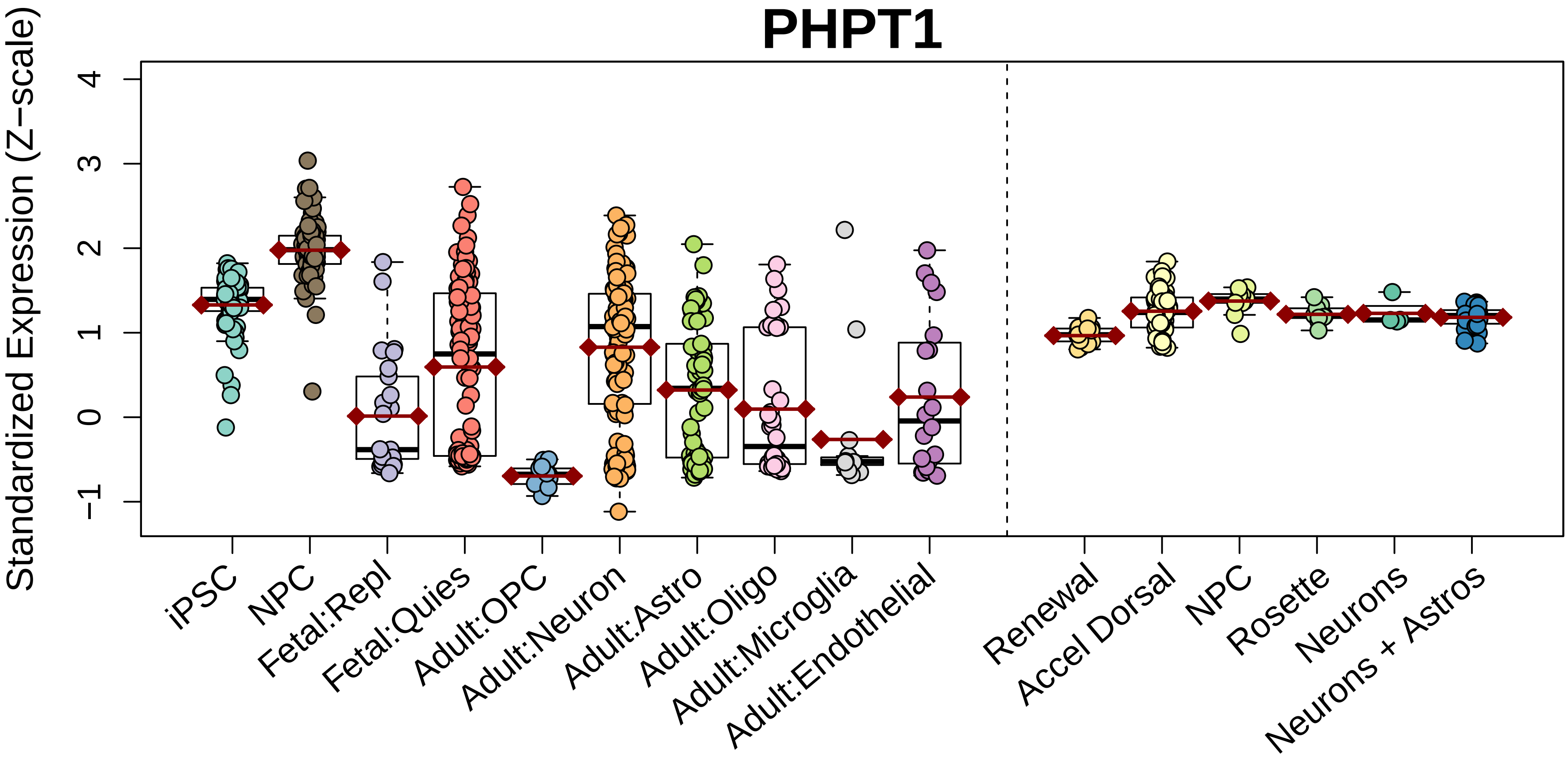
PHPT1
Standardized Expression (Z−scale)
4
3
2
1
0
−1
iPSC
NPC
Fetal:Repl
Fetal:Quies
Adult:OPC
Adult:Neuron
Adult:Astro
Adult:Oligo
Adult:Microglia
Adult:Endothelial
Renewal
Accel Dorsal
NPC
Rosette
Neurons
Neurons + Astros

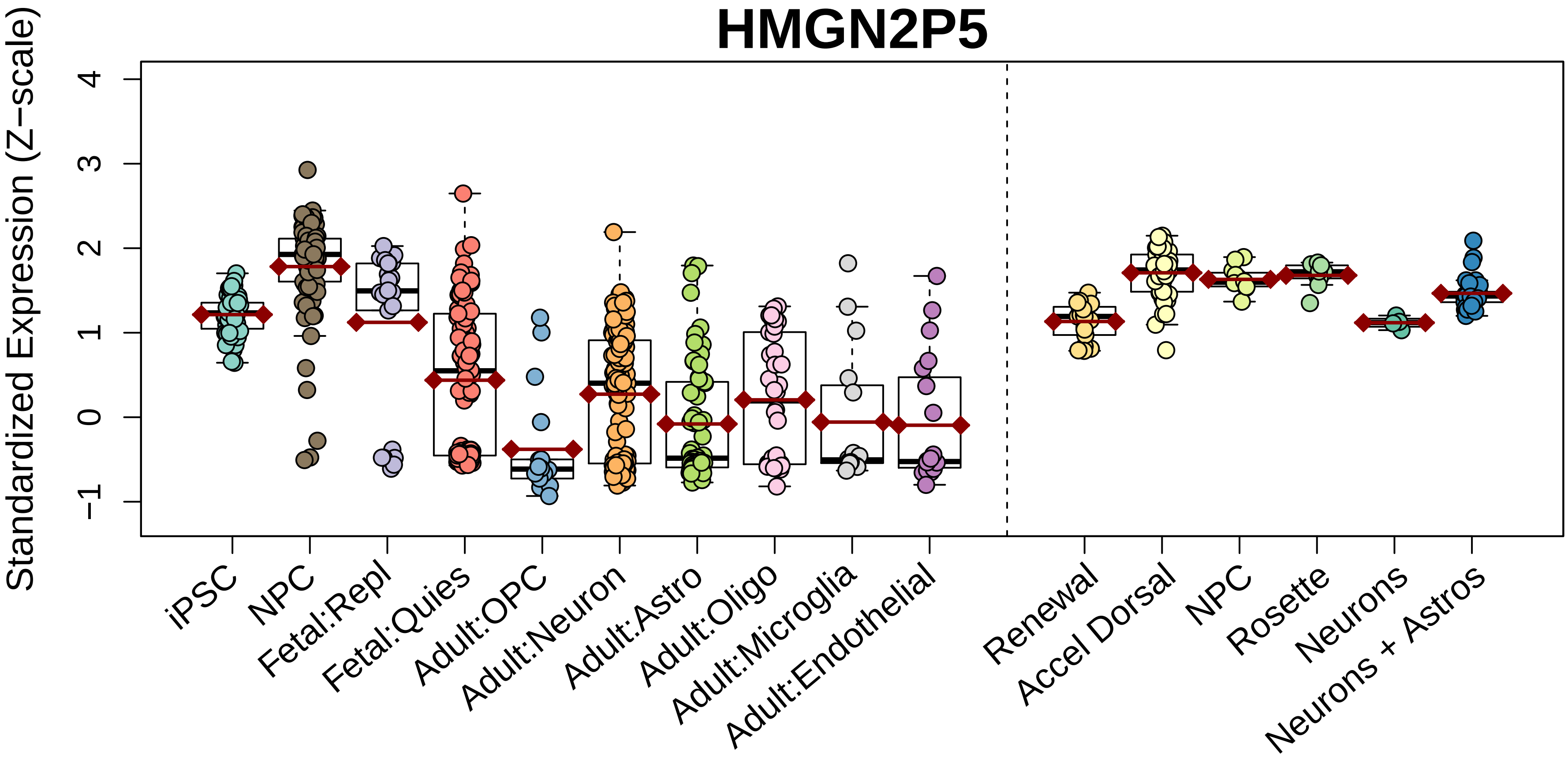

HMGN2P5
Standardized Expression (Z−scale)
4
3
2
1
0
−1
iPSC
NPC
Fetal:Repl
Fetal:Quies
Adult:OPC
Adult:Neuron
Adult:Astro
Adult:Oligo
Adult:Microglia
Adult:Endothelial
Renewal
Accel Dorsal
NPC
Rosette
Neurons
Neurons + Astros

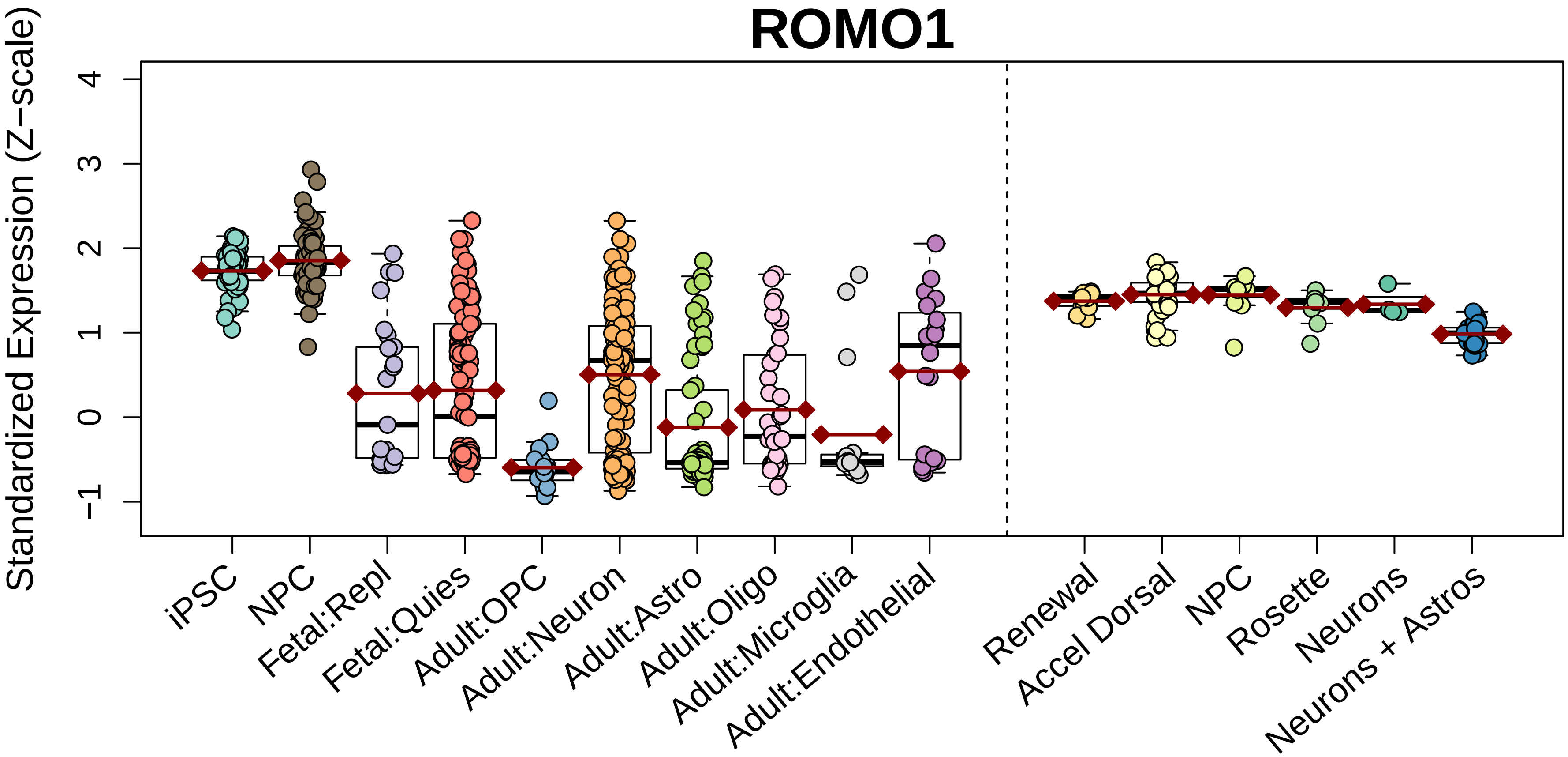
ROMO1
Standardized Expression (Z−scale)
4
3
2
1
0
−1
iPSC
NPC
Fetal:Repl
Fetal:Quies
Adult:OPC
Adult:Neuron
Adult:Astro
Adult:Oligo
Adult:Microglia
Adult:Endothelial
Renewal
Accel Dorsal
NPC
Rosette
Neurons
Neurons + Astros

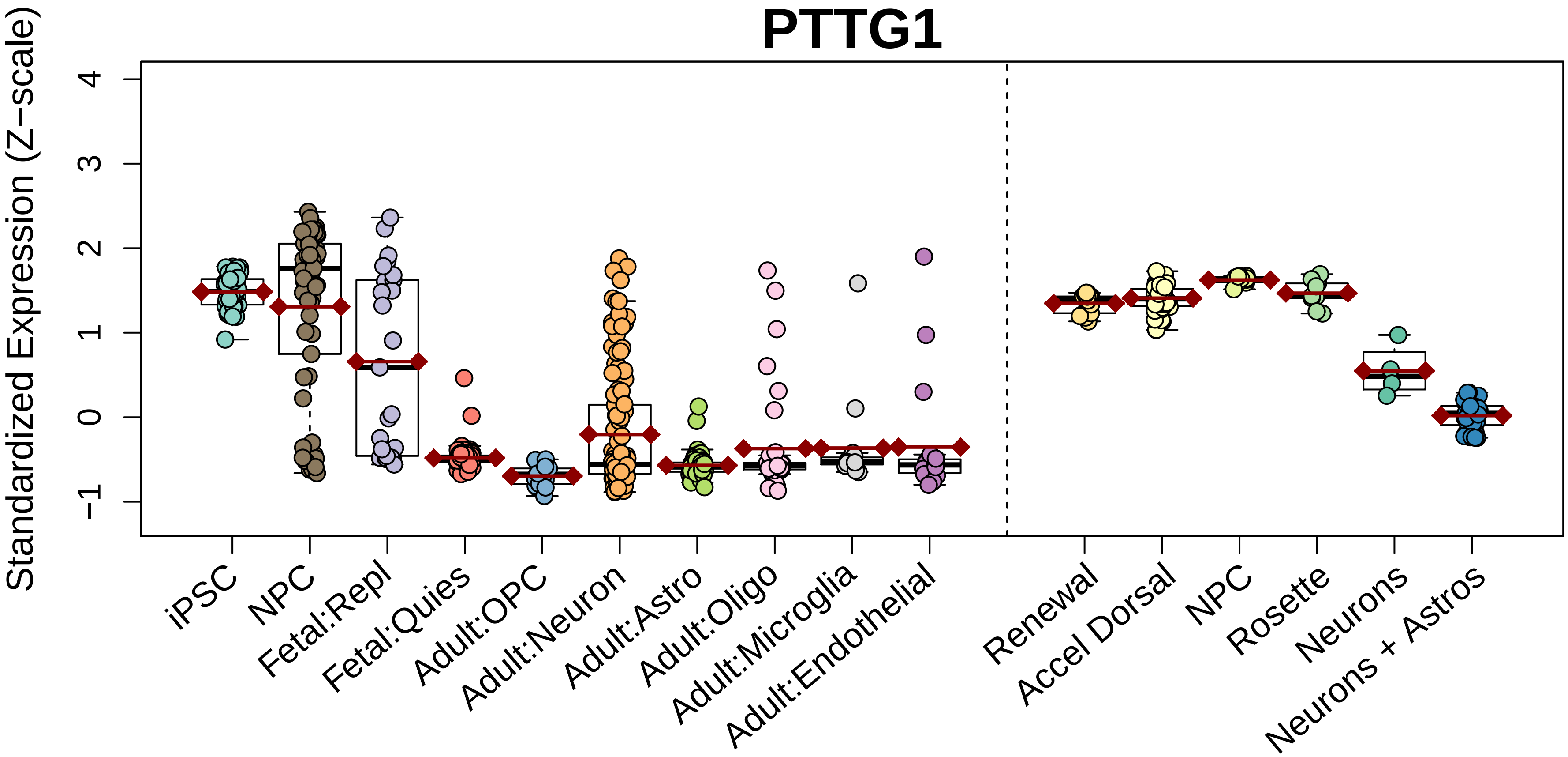
PTTG1
Standardized Expression (Z−scale)
4
3
2
1
0
−1
iPSC
NPC
Fetal:Repl
Fetal:Quies
Adult:OPC
Adult:Neuron
Adult:Astro
Adult:Oligo
Adult:Microglia
Adult:Endothelial
Renewal
Accel Dorsal
NPC
Rosette
Neurons
Neurons + Astros

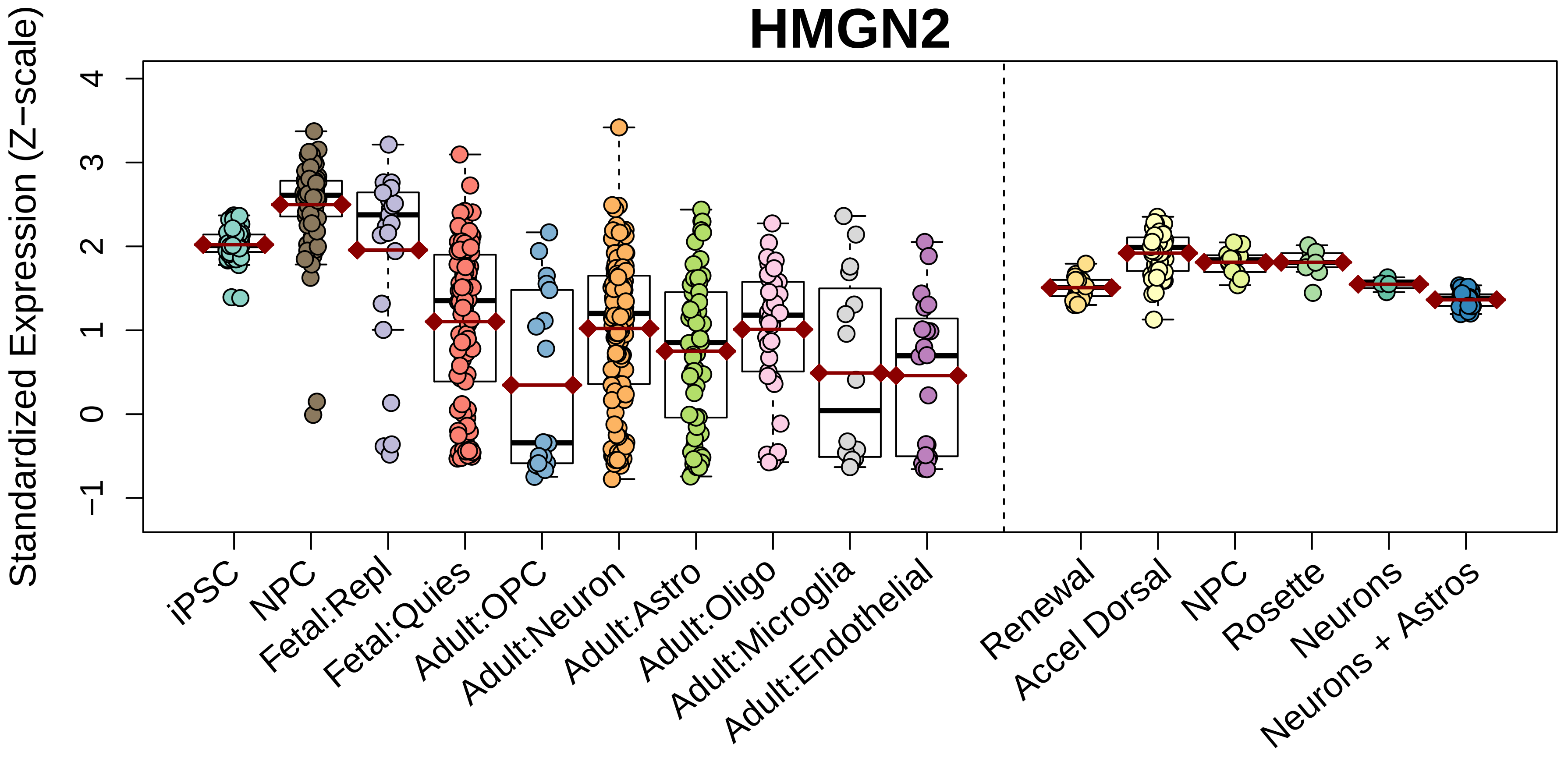
HMGN2
Standardized Expression (Z−scale)
4
3
2
1
0
−1
iPSC
NPC
Fetal:Repl
Fetal:Quies
Adult:OPC
Adult:Neuron
Adult:Astro
Adult:Oligo
Adult:Microglia
Adult:Endothelial
Renewal
Accel Dorsal
NPC
Rosette
Neurons
Neurons + Astros

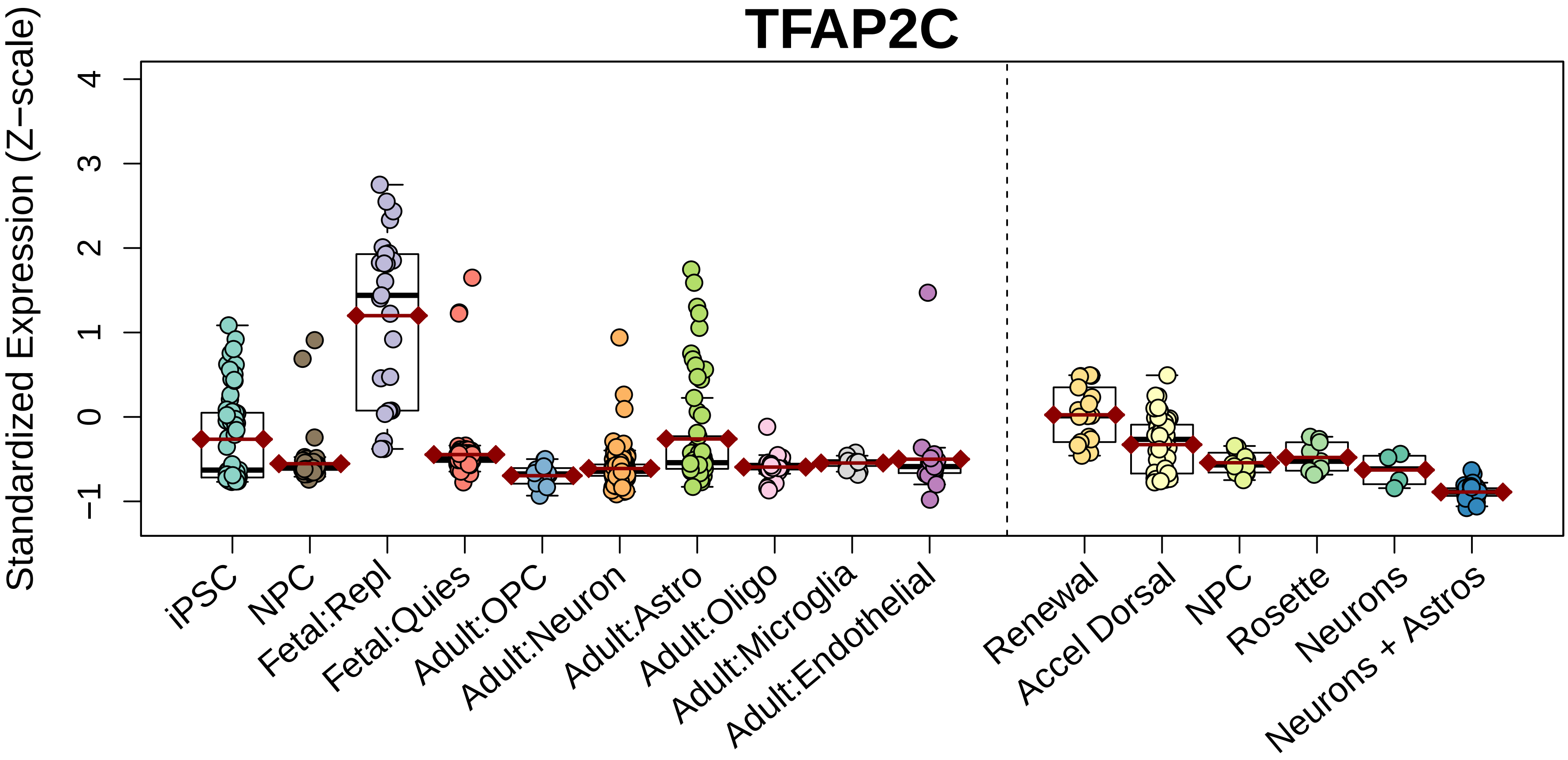
TFAP2C
Standardized Expression (Z−scale)
4
3
2
1
0
−1
iPSC
NPC
Fetal:Repl
Fetal:Quies
Adult:OPC
Adult:Neuron
Adult:Astro
Adult:Oligo
Adult:Microglia
Adult:Endothelial
Renewal
Accel Dorsal
NPC
Rosette
Neurons
Neurons + Astros

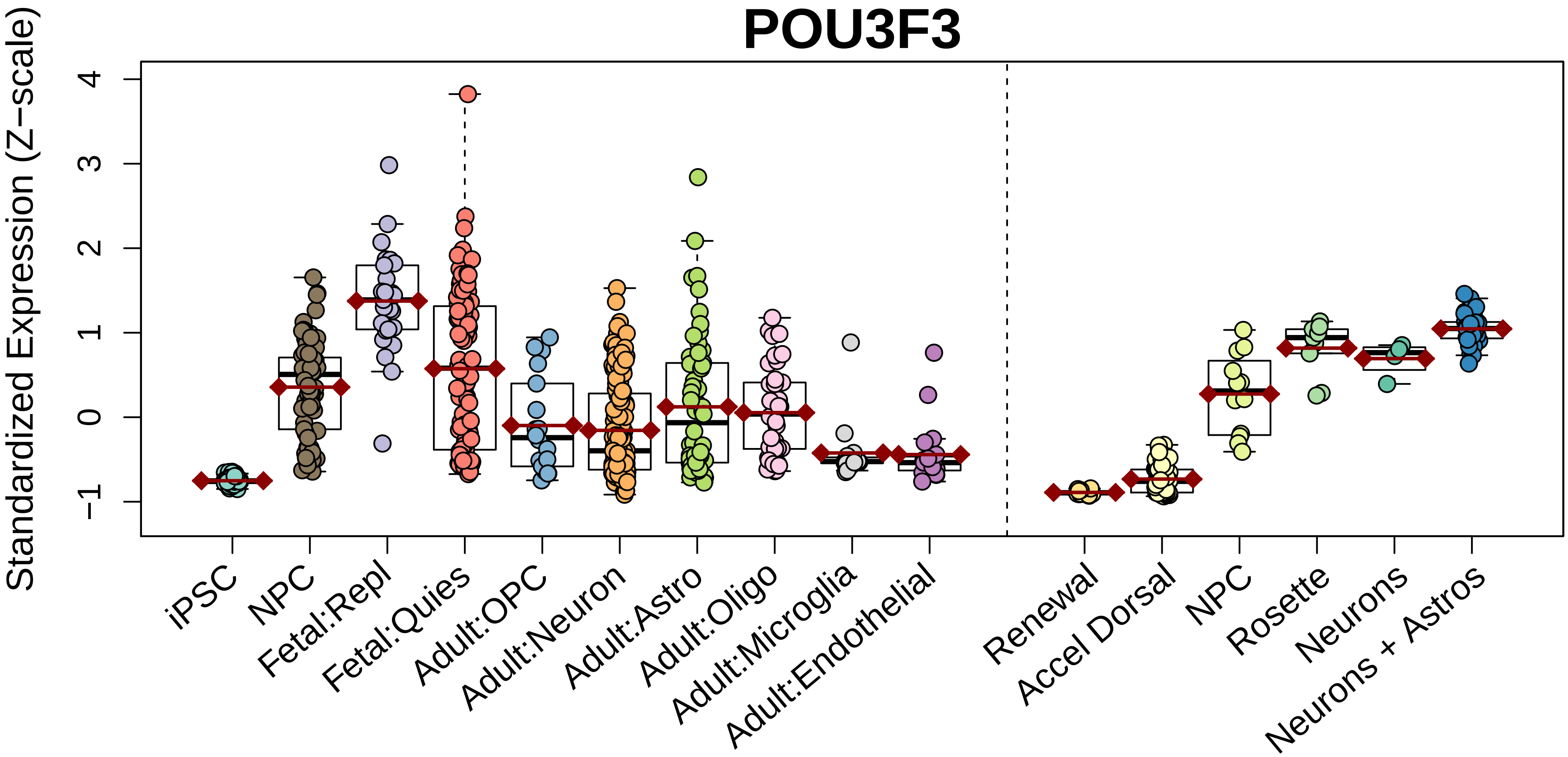

POU3F3
Standardized Expression (Z−scale)
4
3
2
1
0
−1
iPSC
NPC
Fetal:Repl
Fetal:Quies
Adult:OPC
Adult:Neuron
Adult:Astro
Adult:Oligo
Adult:Microglia
Adult:Endothelial
Renewal
Accel Dorsal
NPC
Rosette
Neurons
Neurons + Astros

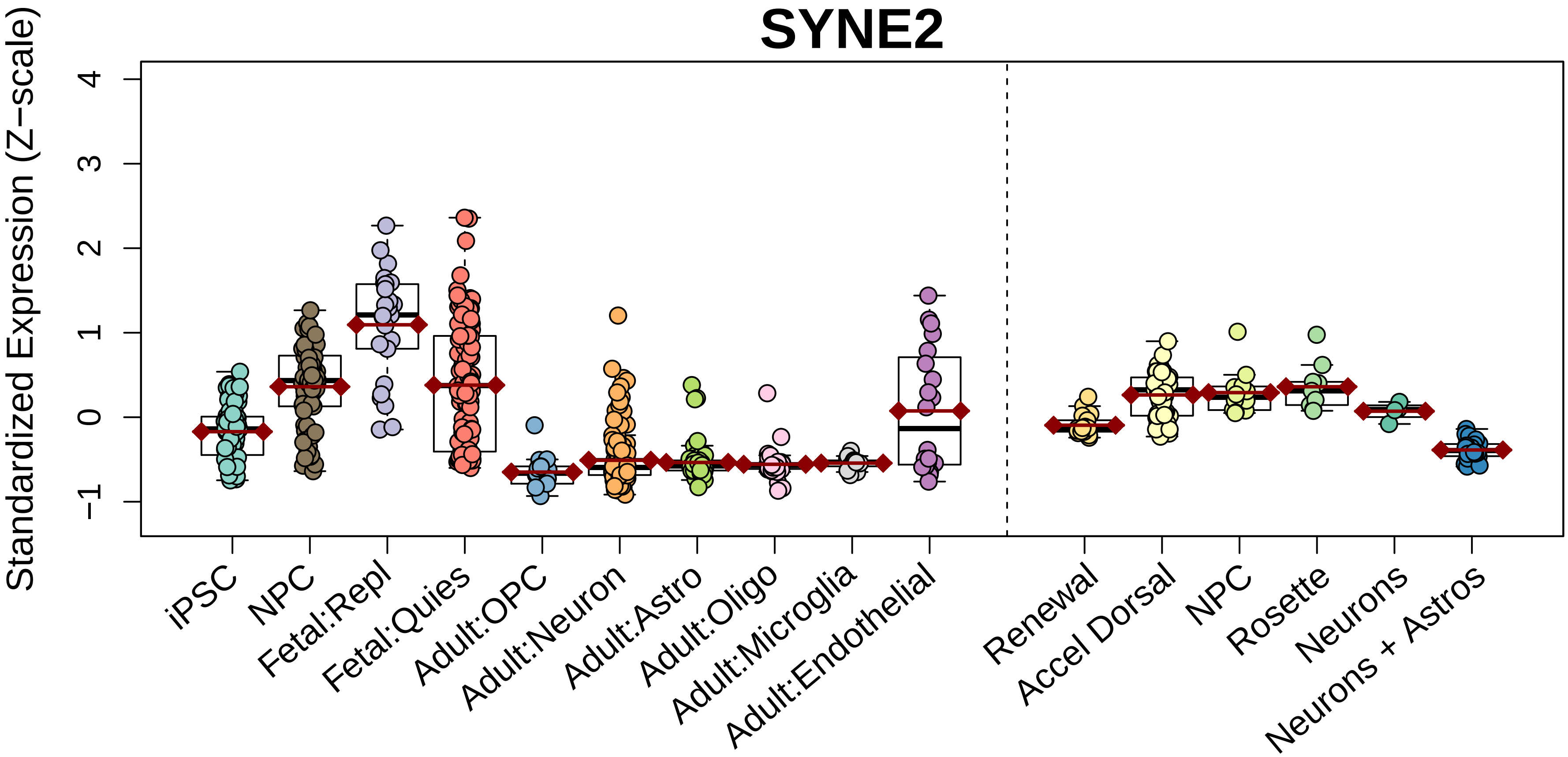

SYNE2
Standardized Expression (Z−scale)
4
3
2
1
0
−1
iPSC
NPC
Fetal:Repl
Fetal:Quies
Adult:OPC
Adult:Neuron
Adult:Astro
Adult:Oligo
Adult:Microglia
Adult:Endothelial
Renewal
Accel Dorsal
NPC
Rosette
Neurons
Neurons + Astros

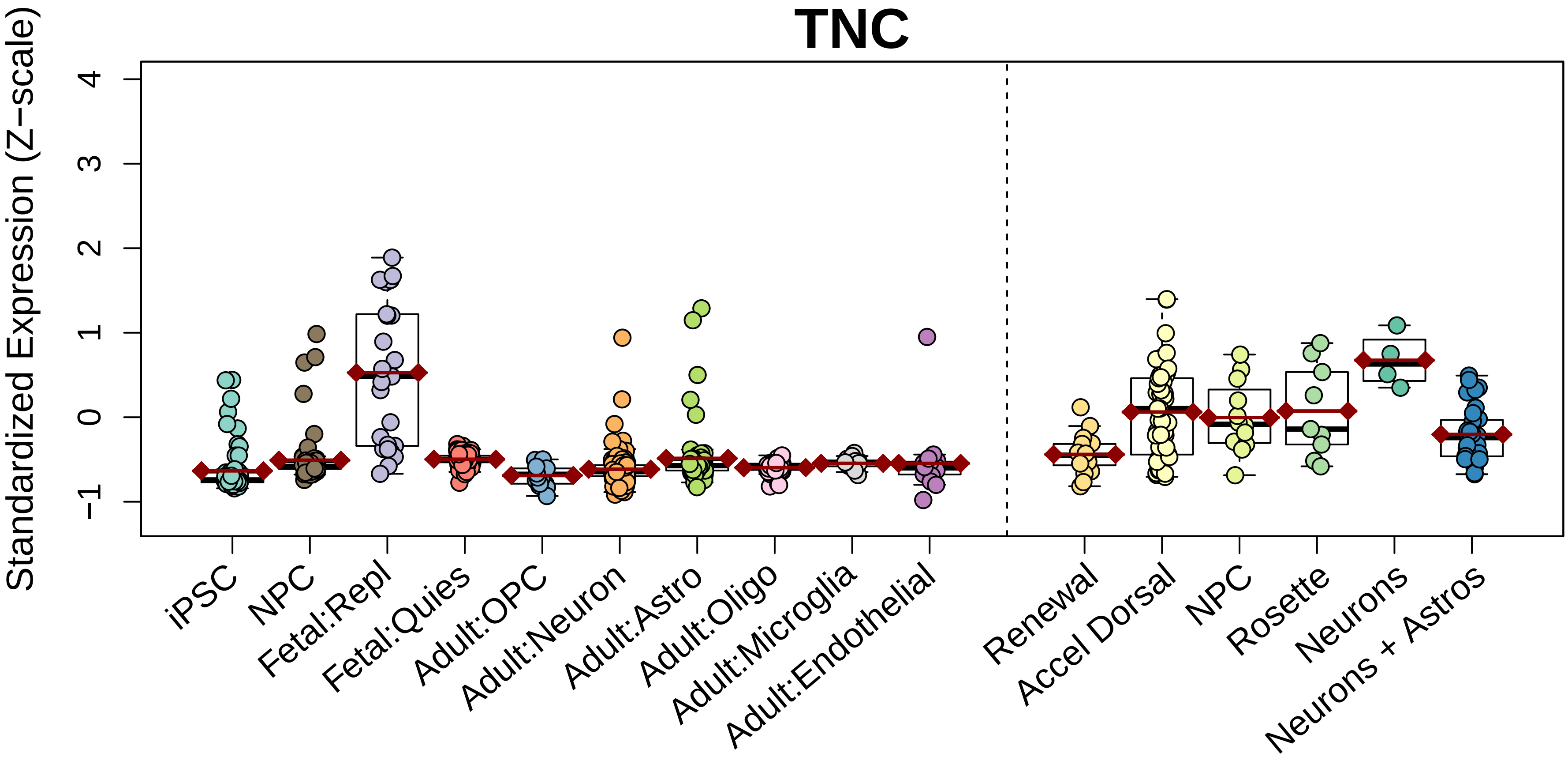

TNC
Standardized Expression (Z−scale)
4
3
2
1
0
−1
iPSC
NPC
Fetal:Repl
Fetal:Quies
Adult:OPC
Adult:Neuron
Adult:Astro
Adult:Oligo
Adult:Microglia
Adult:Endothelial
Renewal
Accel Dorsal
NPC
Rosette
Neurons
Neurons + Astros

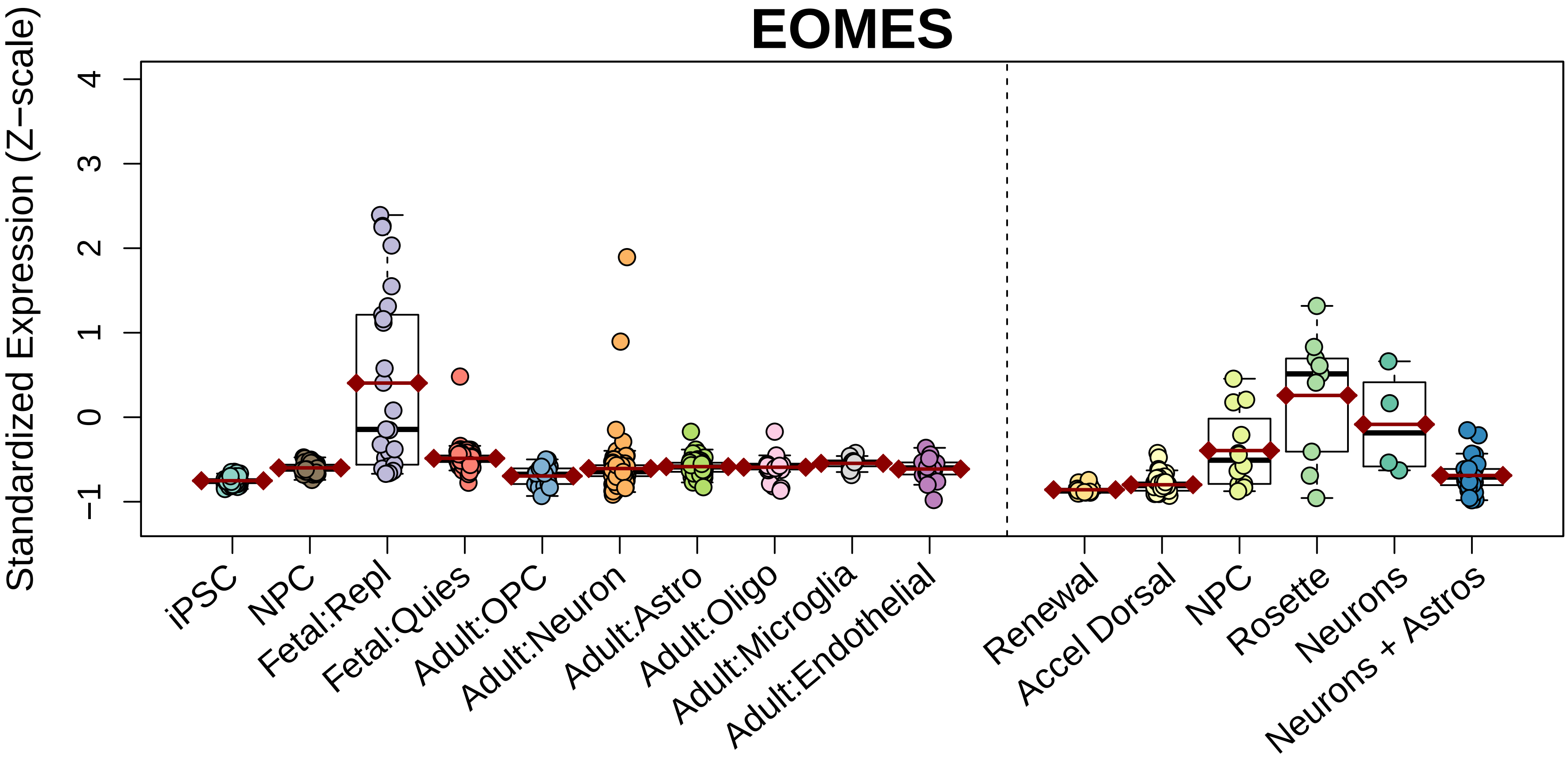
EOMES
Standardized Expression (Z−scale)
4
3
2
1
0
−1
iPSC
NPC
Fetal:Repl
Fetal:Quies
Adult:OPC
Adult:Neuron
Adult:Astro
Adult:Oligo
Adult:Microglia
Adult:Endothelial
Renewal
Accel Dorsal
NPC
Rosette
Neurons
Neurons + Astros

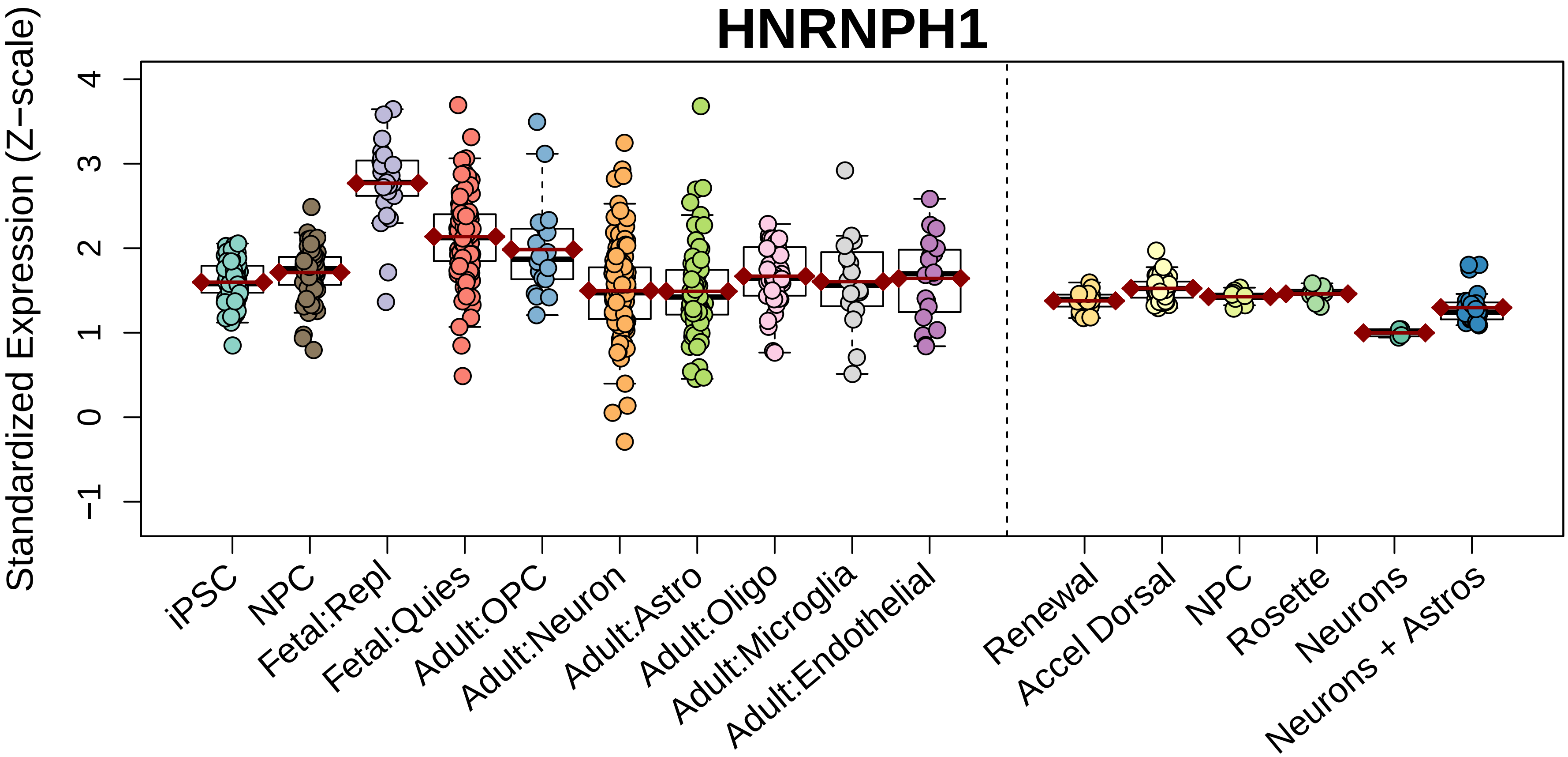

HNRNPH1
Standardized Expression (Z−scale)
−1
0
1
2
3
4
iPSC
NPC
Fetal:Repl
Fetal:Quies
Adult:OPC
Adult:Neuron
Adult:Astro
Adult:Oligo
Adult:Microglia
Adult:Endothelial
Renewal
Accel Dorsal
NPC
Rosette
Neurons
Neurons + Astros

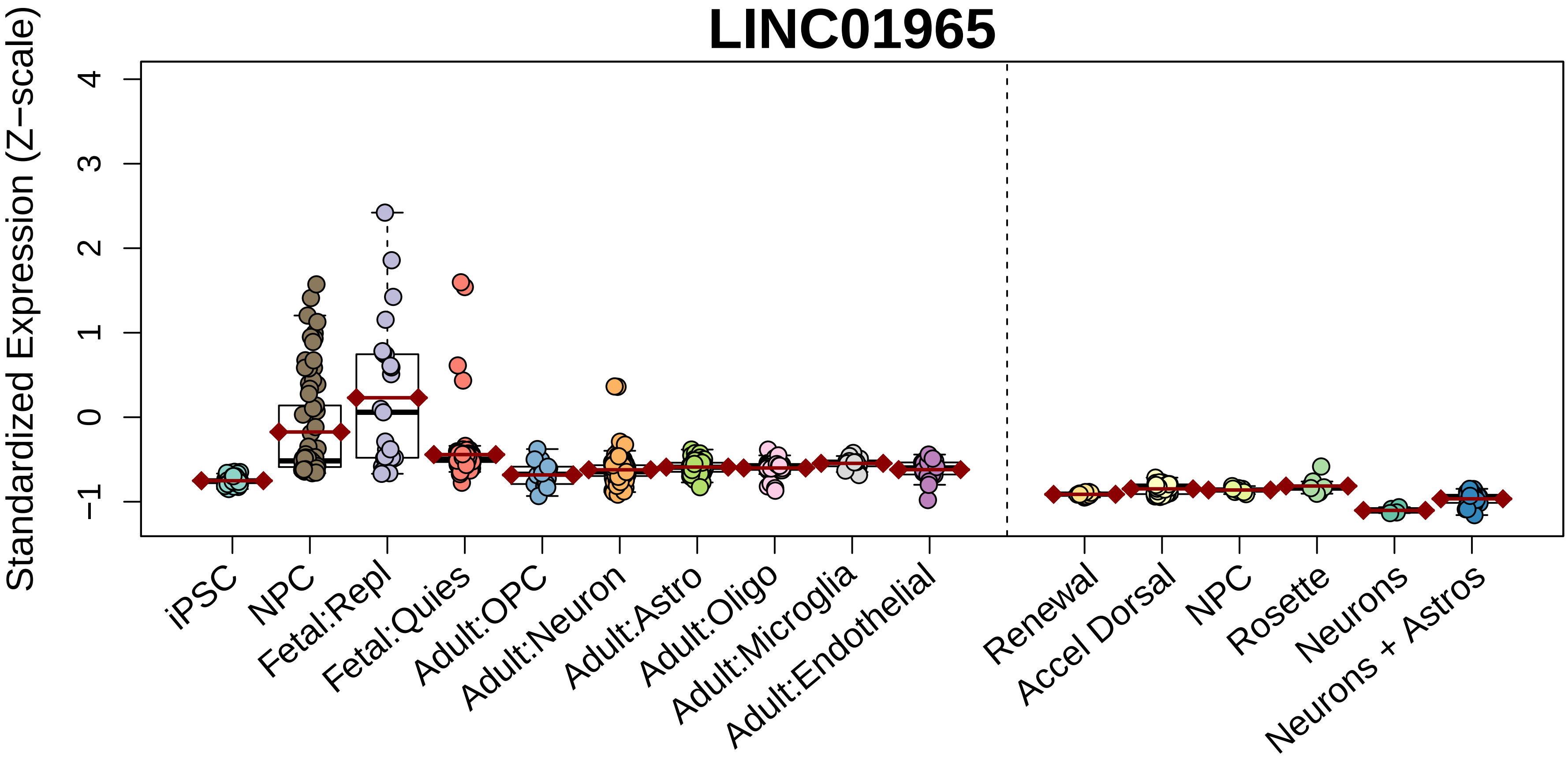
LINC01965
Standardized Expression (Z−scale)
4
3
2
1
0
−1
iPSC
NPC
Fetal:Repl
Fetal:Quies
Adult:OPC
Adult:Neuron
Adult:Astro
Adult:Oligo
Adult:Microglia
Adult:Endothelial
Renewal
Accel Dorsal
NPC
Rosette
Neurons
Neurons + Astros

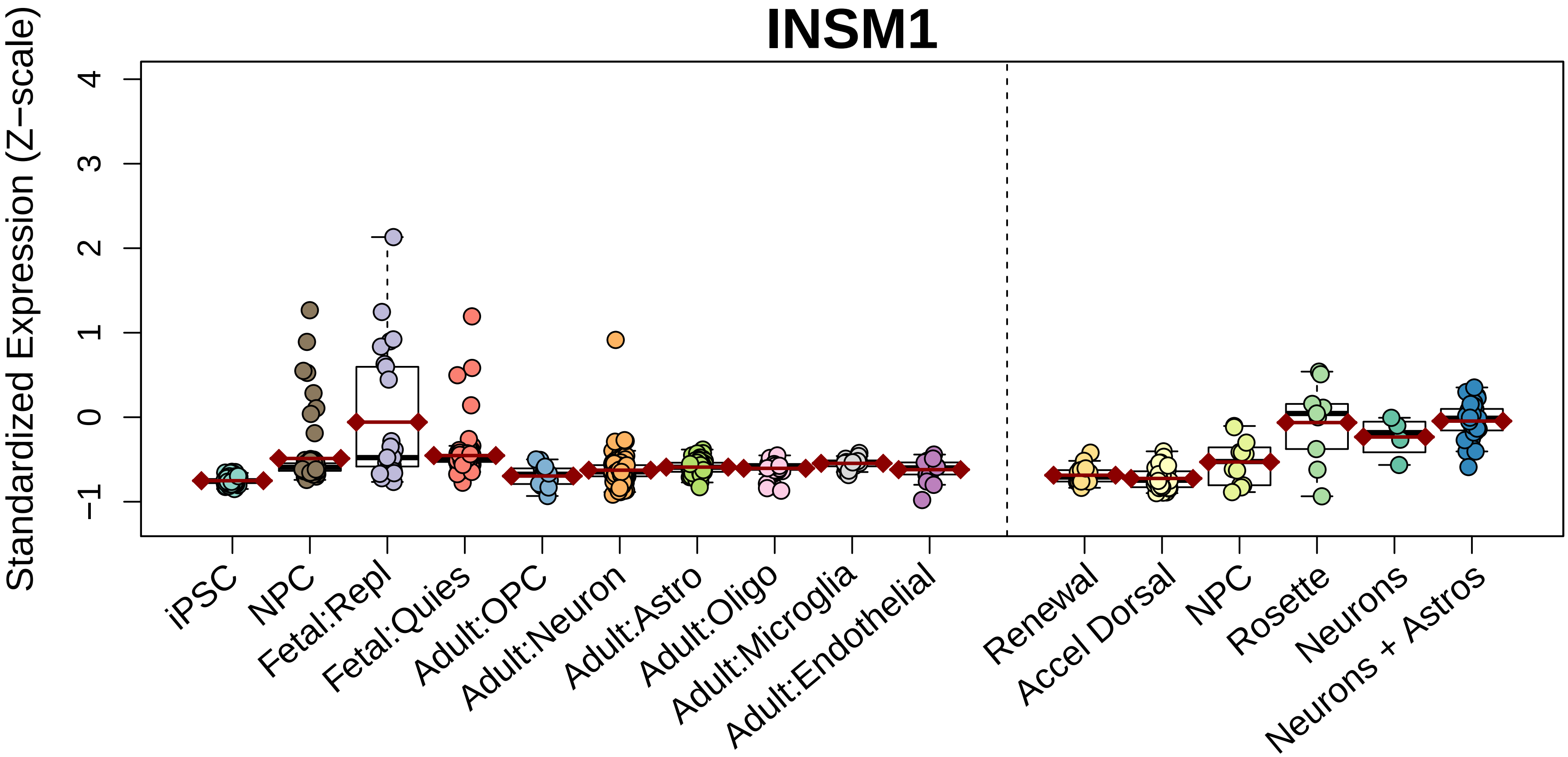

INSM1
Standardized Expression (Z−scale)
4
3
2
1
0
−1
iPSC
NPC
Fetal:Repl
Fetal:Quies
Adult:OPC
Adult:Neuron
Adult:Astro
Adult:Oligo
Adult:Microglia
Adult:Endothelial
Renewal
Accel Dorsal
NPC
Rosette
Neurons
Neurons + Astros

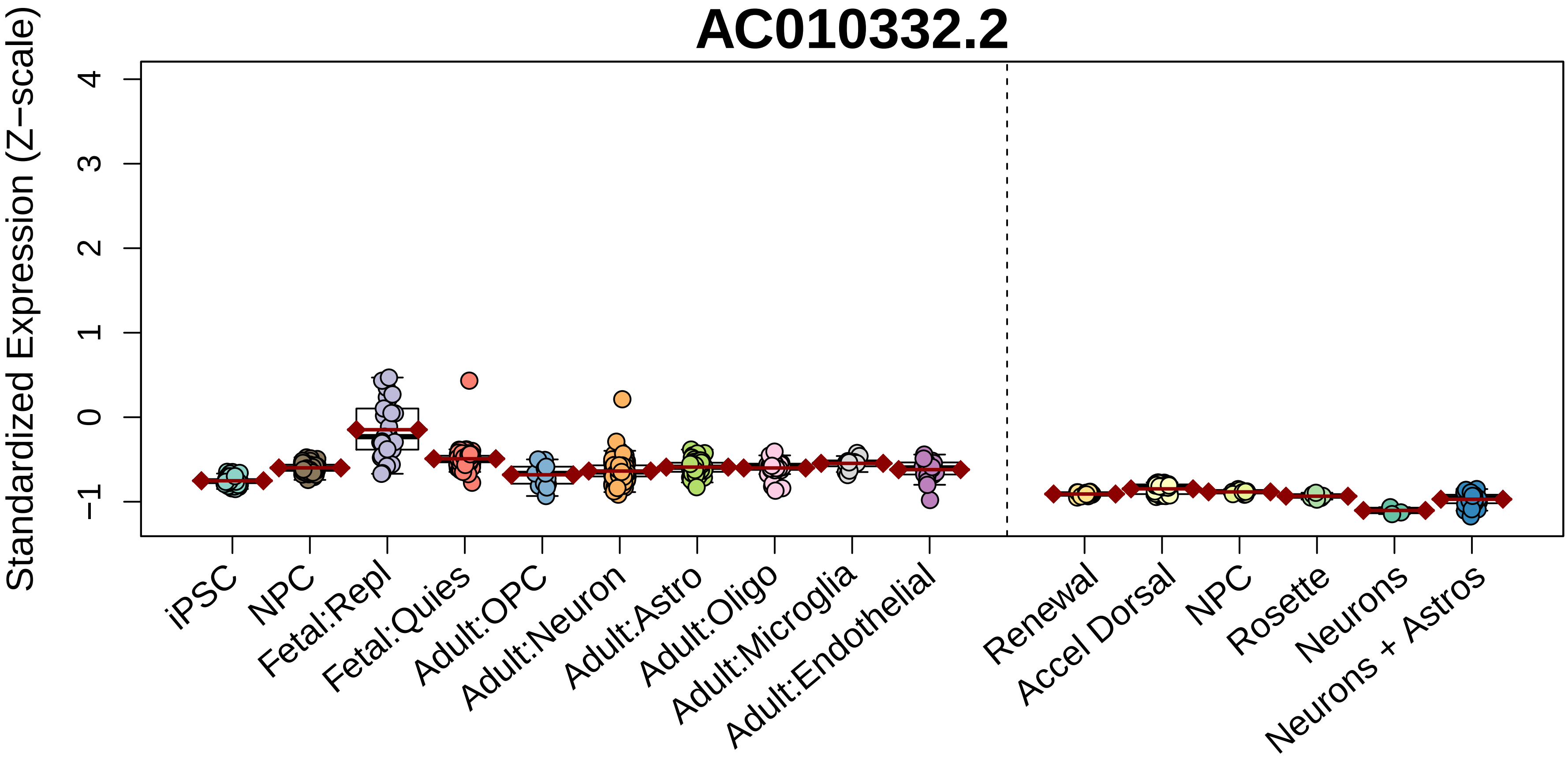

AC010332.2
Standardized Expression (Z−scale)
4
3
2
1
0
−1
iPSC
NPC
Fetal:Repl
Fetal:Quies
Adult:OPC
Adult:Neuron
Adult:Astro
Adult:Oligo
Adult:Microglia
Adult:Endothelial
Renewal
Accel Dorsal
NPC
Rosette
Neurons
Neurons + Astros

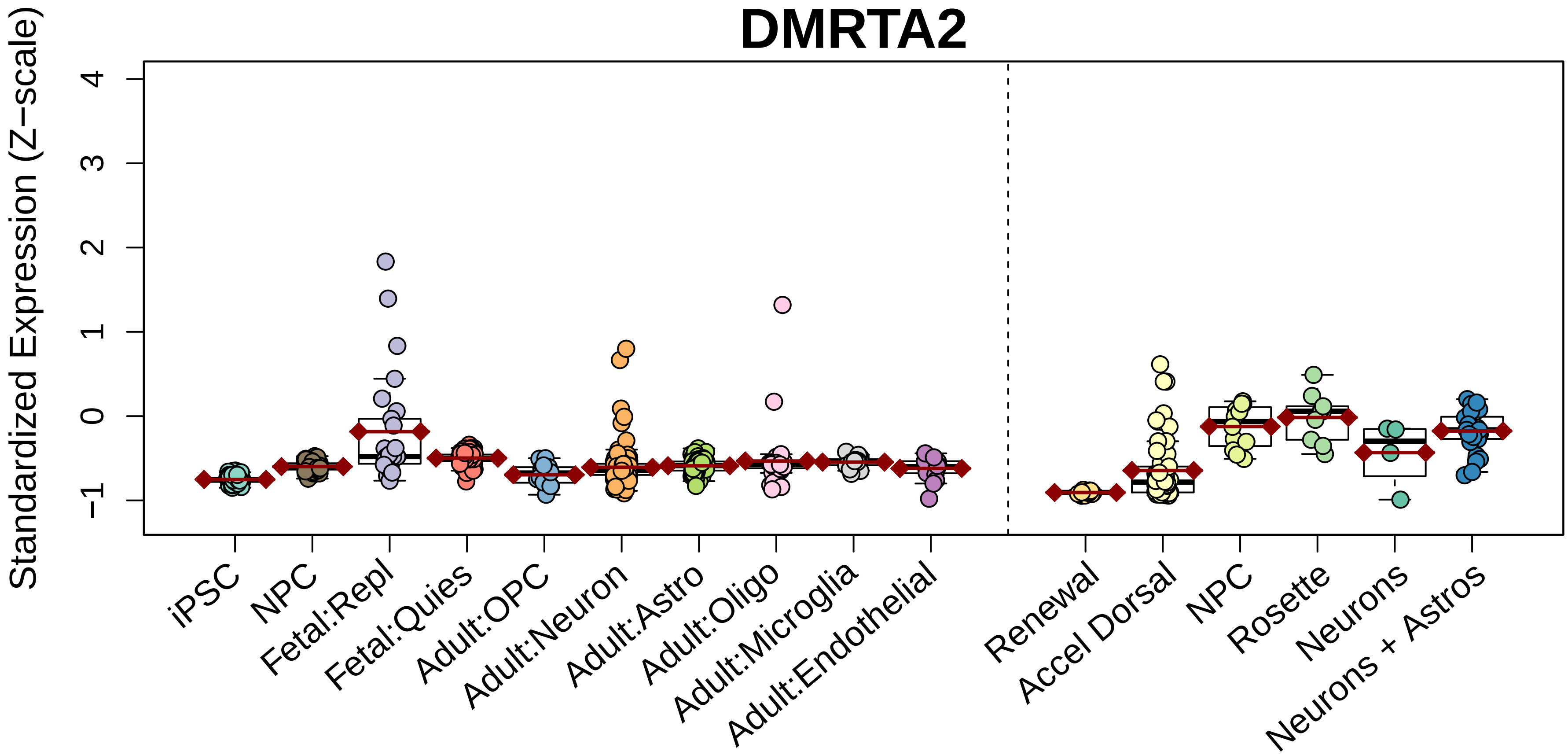

DMRTA2
Standardized Expression (Z−scale)
4
3
2
1
0
−1
iPSC
NPC
Fetal:Repl
Fetal:Quies
Adult:OPC
Adult:Neuron
Adult:Astro
Adult:Oligo
Adult:Microglia
Adult:Endothelial
Renewal
Accel Dorsal
NPC
Rosette
Neurons
Neurons + Astros

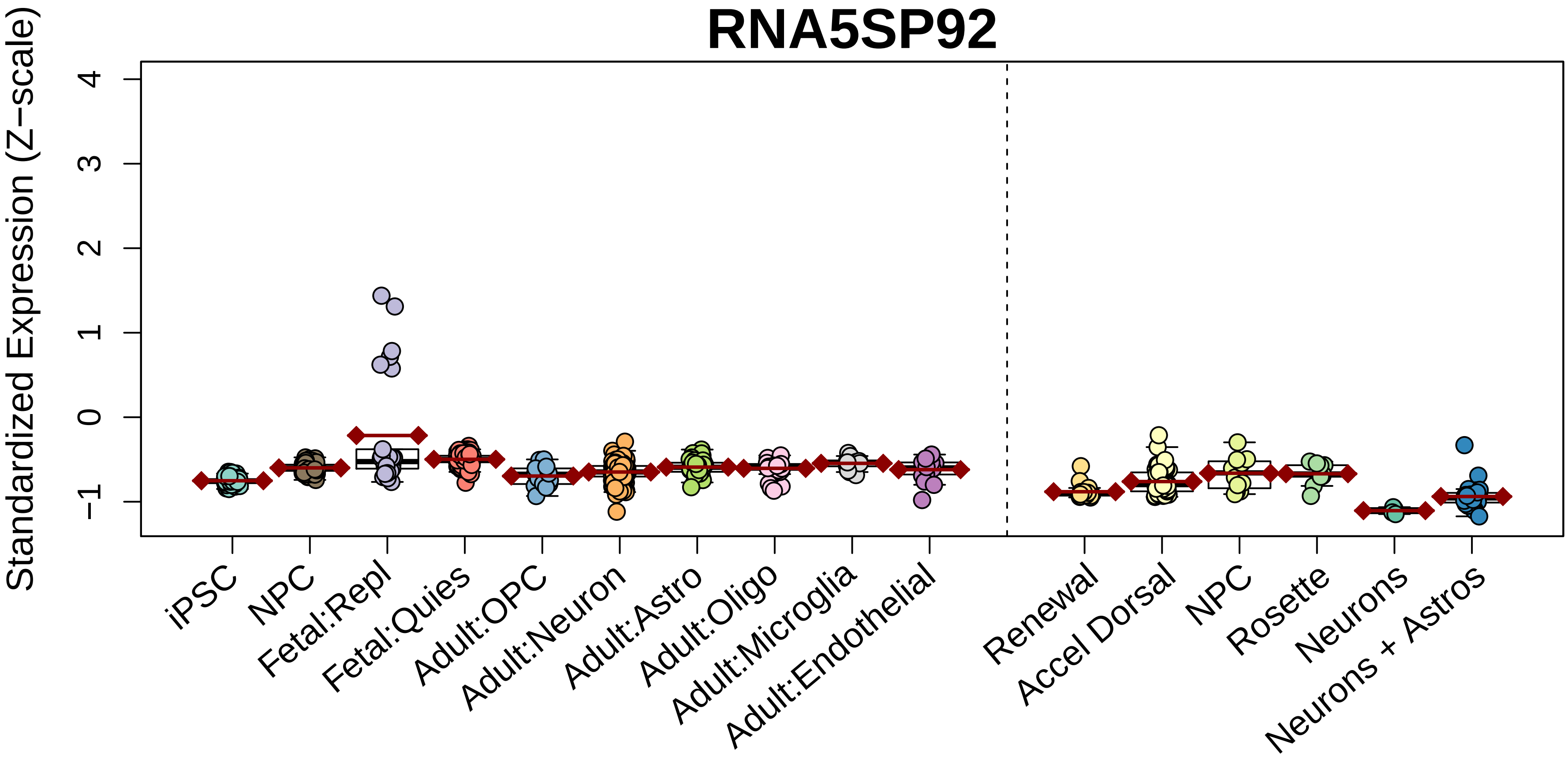
RNA5SP92
Standardized Expression (Z−scale)
4
3
2
1
0
−1
iPSC
NPC
Fetal:Repl
Fetal:Quies
Adult:OPC
Adult:Neuron
Adult:Astro
Adult:Oligo
Adult:Microglia
Adult:Endothelial
Renewal
Accel Dorsal
NPC
Rosette
Neurons
Neurons + Astros

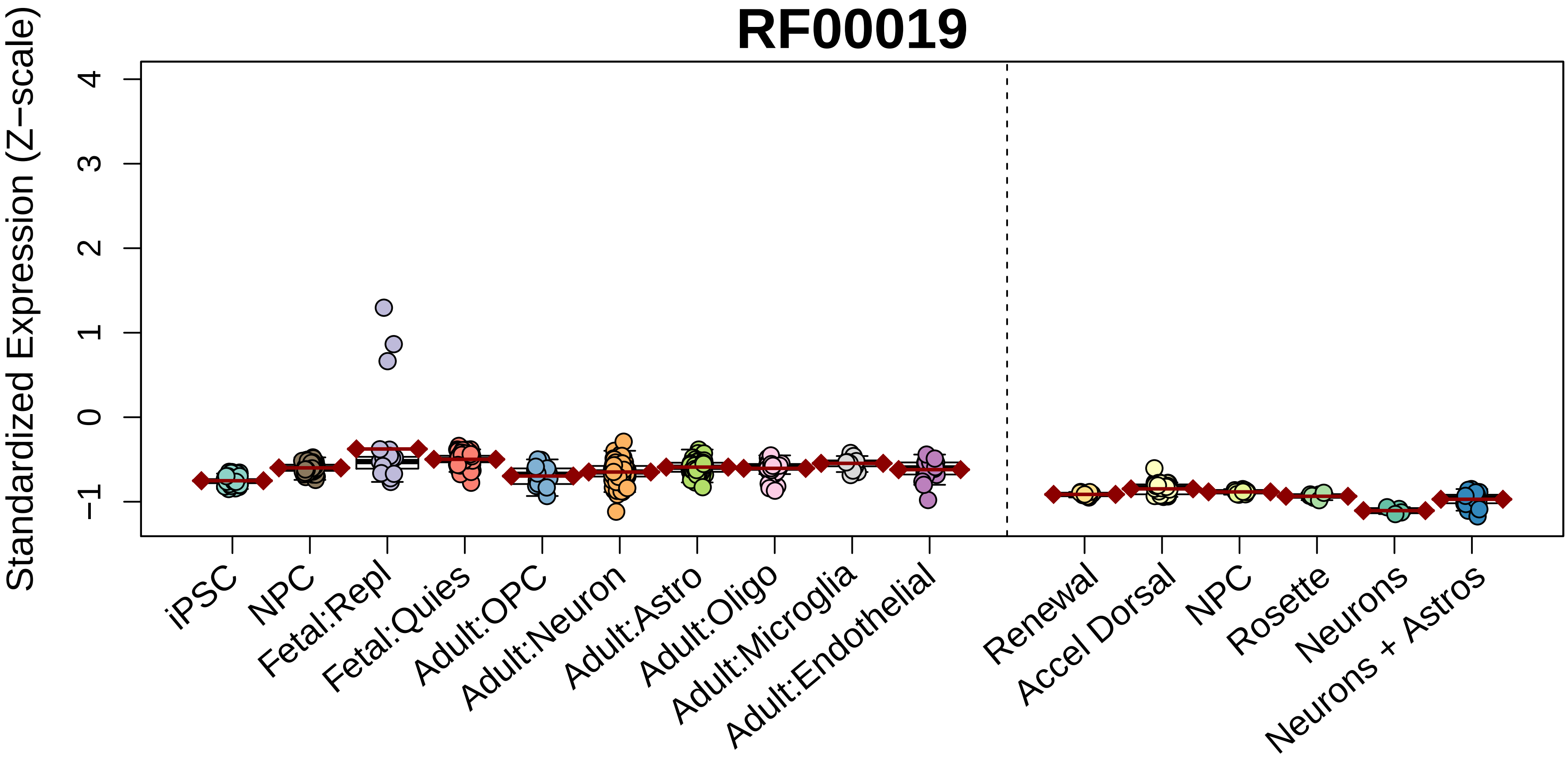
RF00019
Standardized Expression (Z−scale)
4
3
2
1
0
−1
iPSC
NPC
Fetal:Repl
Fetal:Quies
Adult:OPC
Adult:Neuron
Adult:Astro
Adult:Oligo
Adult:Microglia
Adult:Endothelial
Renewal
Accel Dorsal
NPC
Rosette
Neurons
Neurons + Astros

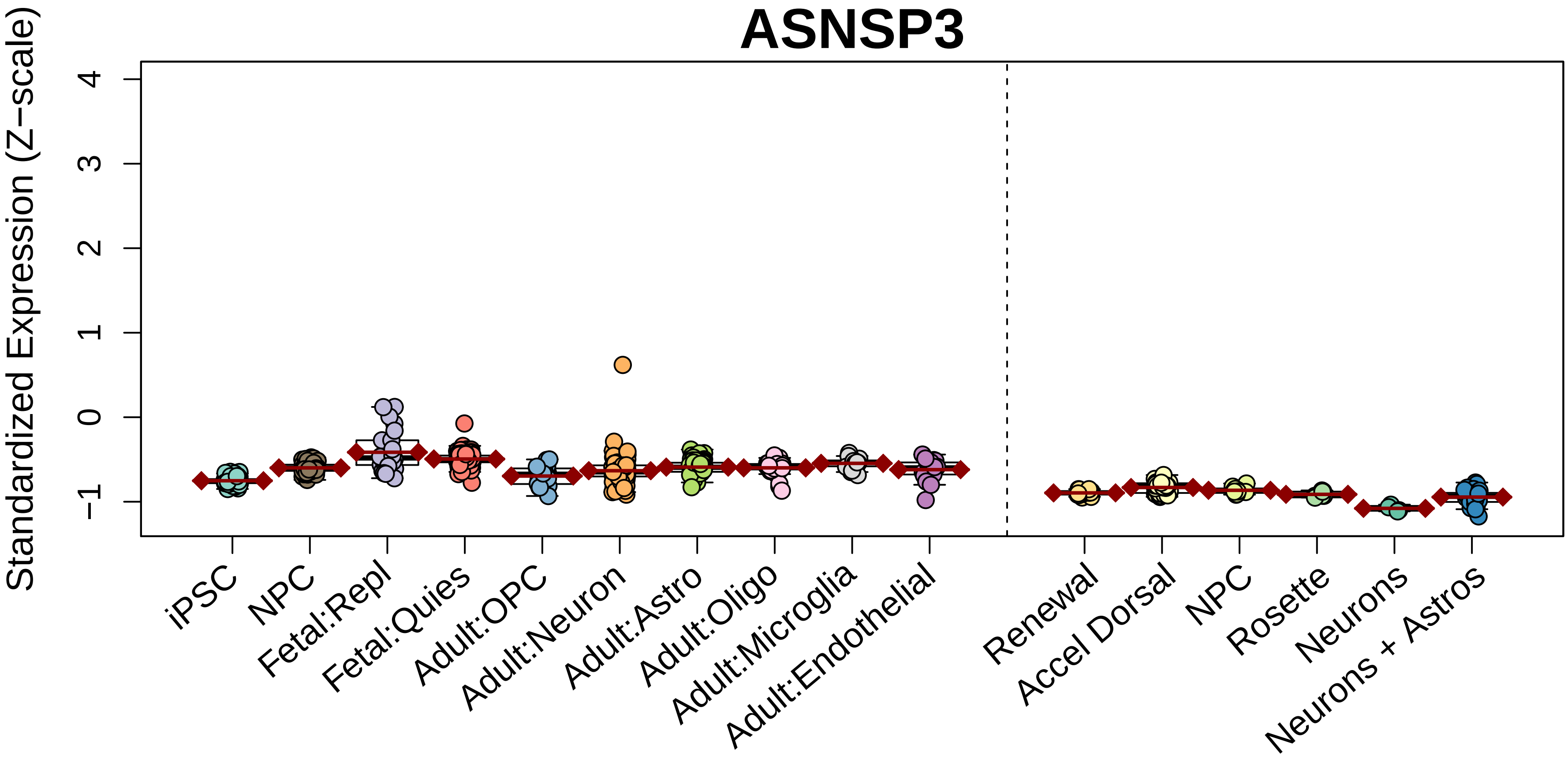
ASNSP3
Standardized Expression (Z−scale)
4
3
2
1
0
−1
iPSC
NPC
Fetal:Repl
Fetal:Quies
Adult:OPC
Adult:Neuron
Adult:Astro
Adult:Oligo
Adult:Microglia
Adult:Endothelial
Renewal
Accel Dorsal
NPC
Rosette
Neurons
Neurons + Astros

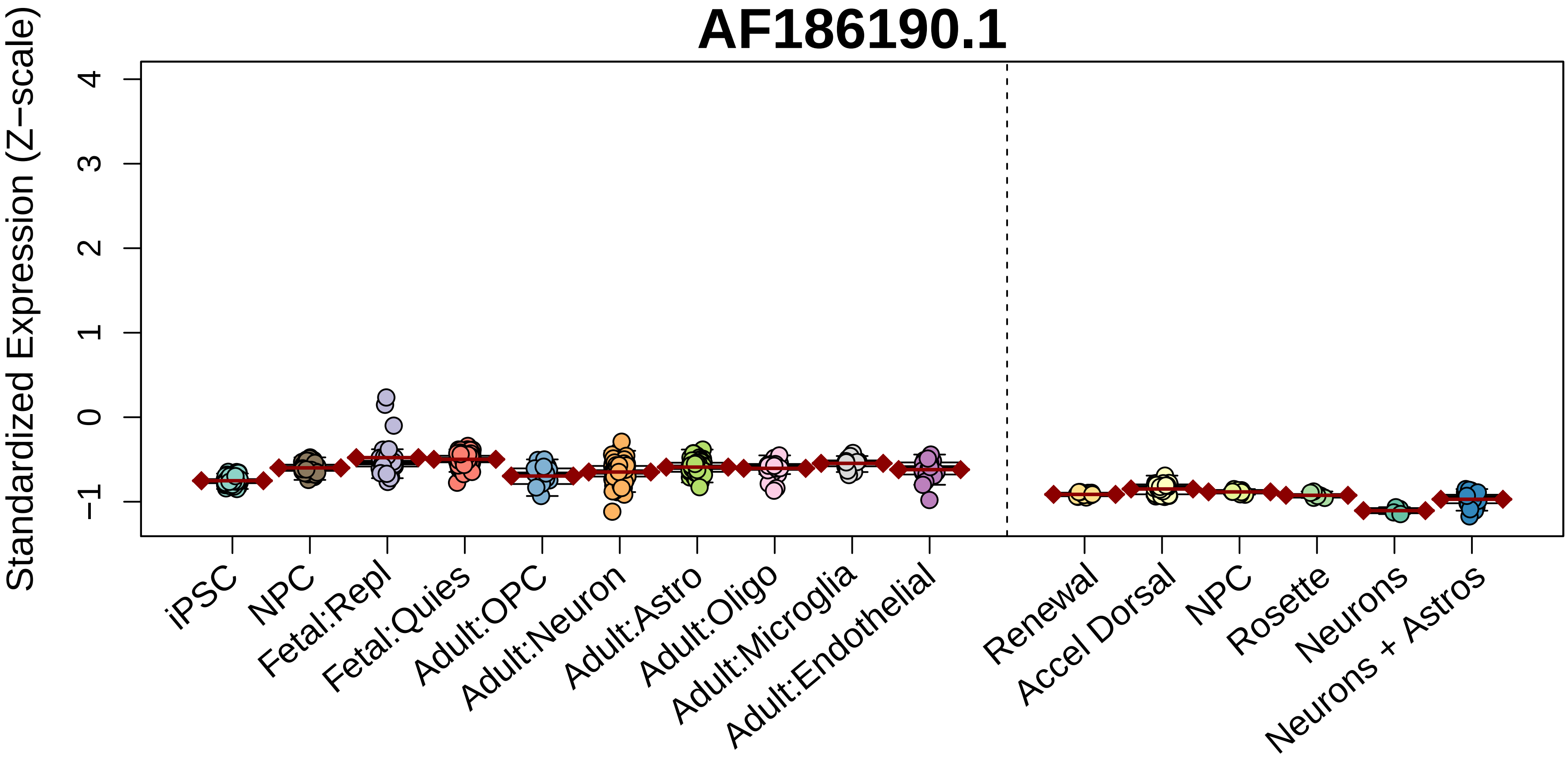
AF186190.1
Standardized Expression (Z−scale)
4
3
2
1
0
−1
iPSC
NPC
Fetal:Repl
Fetal:Quies
Adult:OPC
Adult:Neuron
Adult:Astro
Adult:Oligo
Adult:Microglia
Adult:Endothelial
Renewal
Accel Dorsal
NPC
Rosette
Neurons
Neurons + Astros

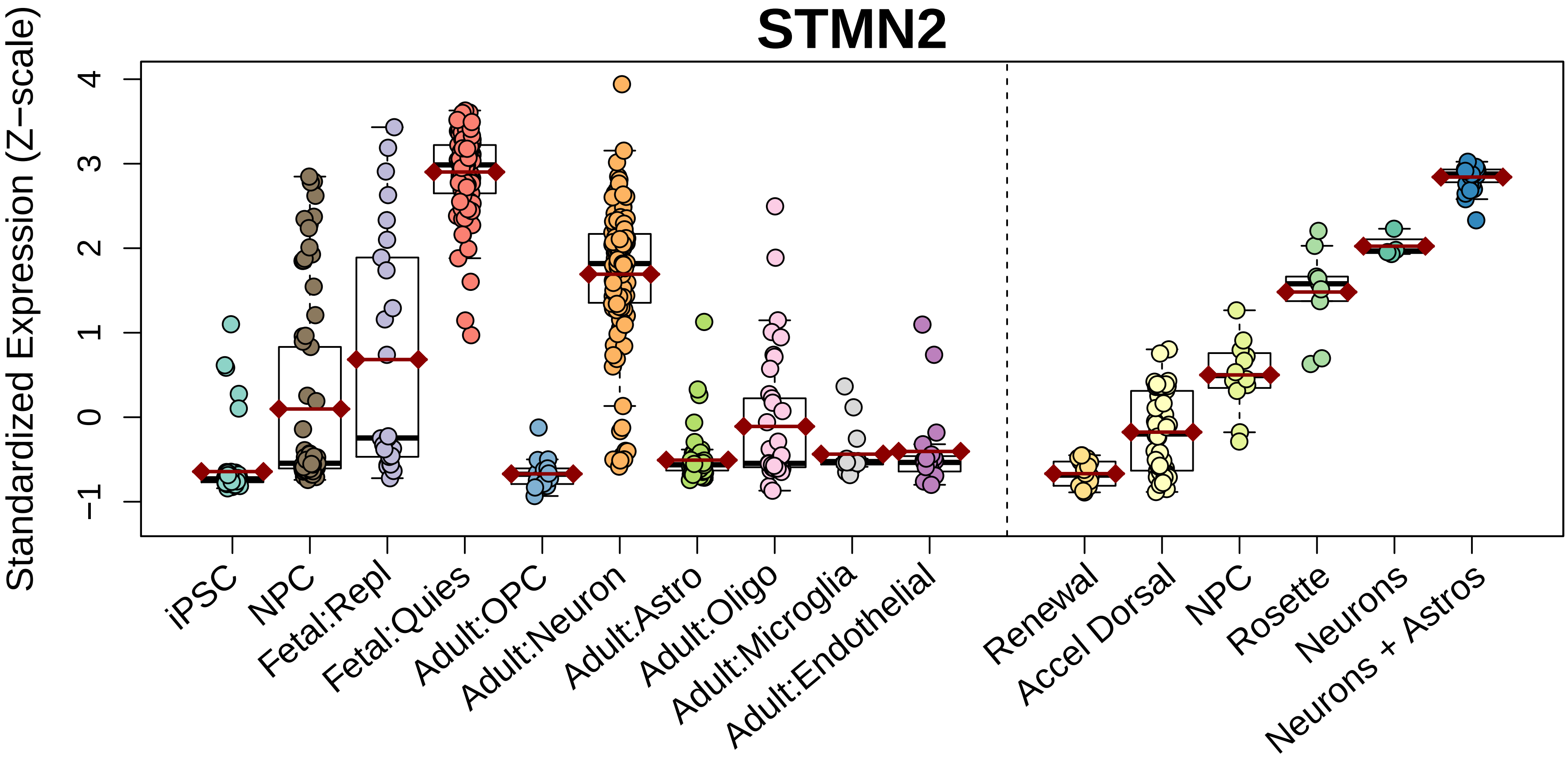
STMN2
Standardized Expression (Z−scale)
4
3
2
1
0
−1
iPSC
NPC
Fetal:Repl
Fetal:Quies
Adult:OPC
Adult:Neuron
Adult:Astro
Adult:Oligo
Adult:Microglia
Adult:Endothelial
Renewal
Accel Dorsal
NPC
Rosette
Neurons
Neurons + Astros

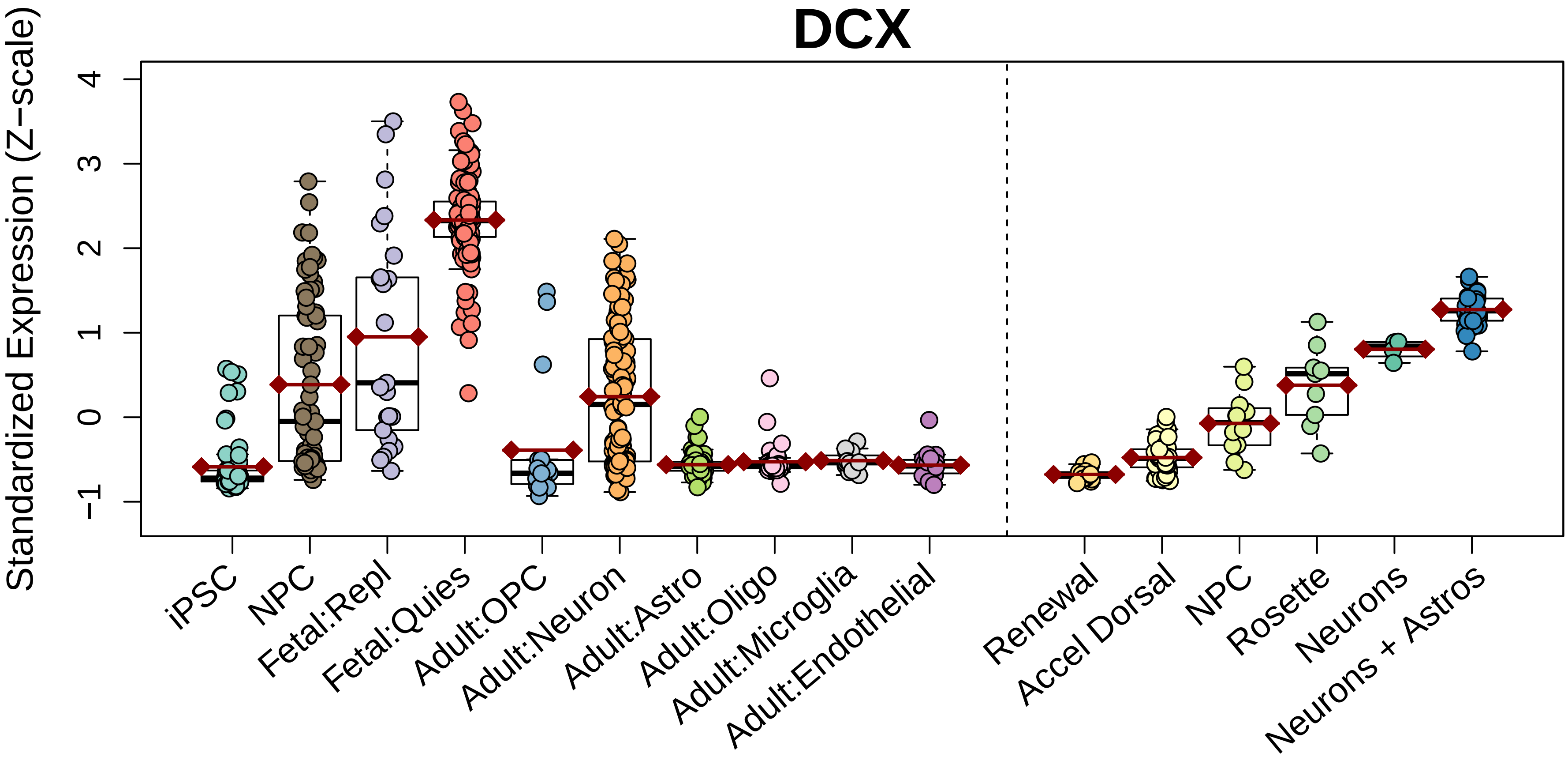

DCX
Standardized Expression (Z−scale)
4
3
2
1
0
−1
iPSC
NPC
Fetal:Repl
Fetal:Quies
Adult:OPC
Adult:Neuron
Adult:Astro
Adult:Oligo
Adult:Microglia
Adult:Endothelial
Renewal
Accel Dorsal
NPC
Rosette
Neurons
Neurons + Astros

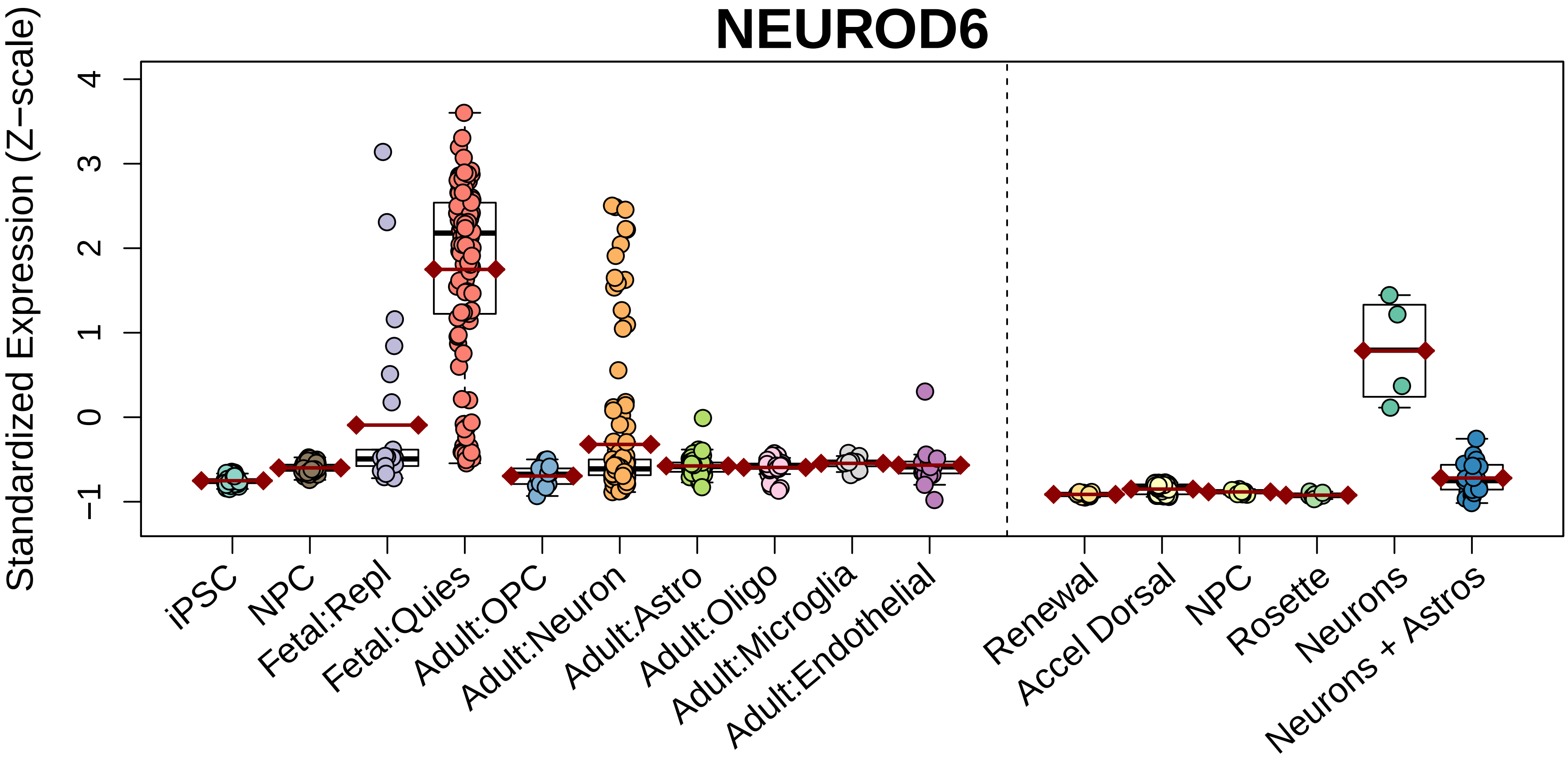

NEUROD6
Standardized Expression (Z−scale)
4
3
2
1
0
−1
iPSC
NPC
Fetal:Repl
Fetal:Quies
Adult:OPC
Adult:Neuron
Adult:Astro
Adult:Oligo
Adult:Microglia
Adult:Endothelial
Renewal
Accel Dorsal
NPC
Rosette
Neurons
Neurons + Astros

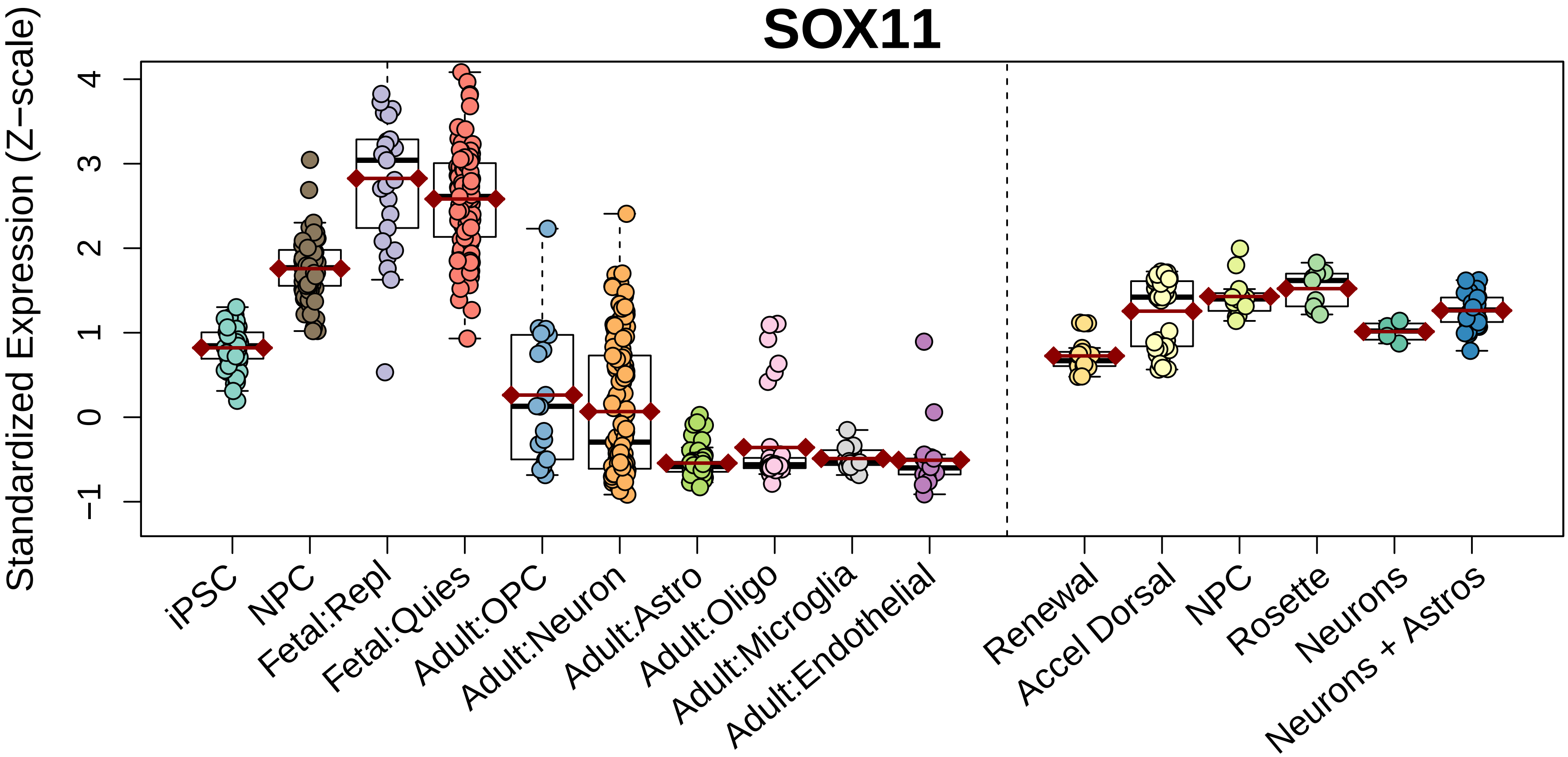

SOX11
Standardized Expression (Z−scale)
4
3
2
1
0
−1
iPSC
NPC
Fetal:Repl
Fetal:Quies
Adult:OPC
Adult:Neuron
Adult:Astro
Adult:Oligo
Adult:Microglia
Adult:Endothelial
Renewal
Accel Dorsal
NPC
Rosette
Neurons
Neurons + Astros

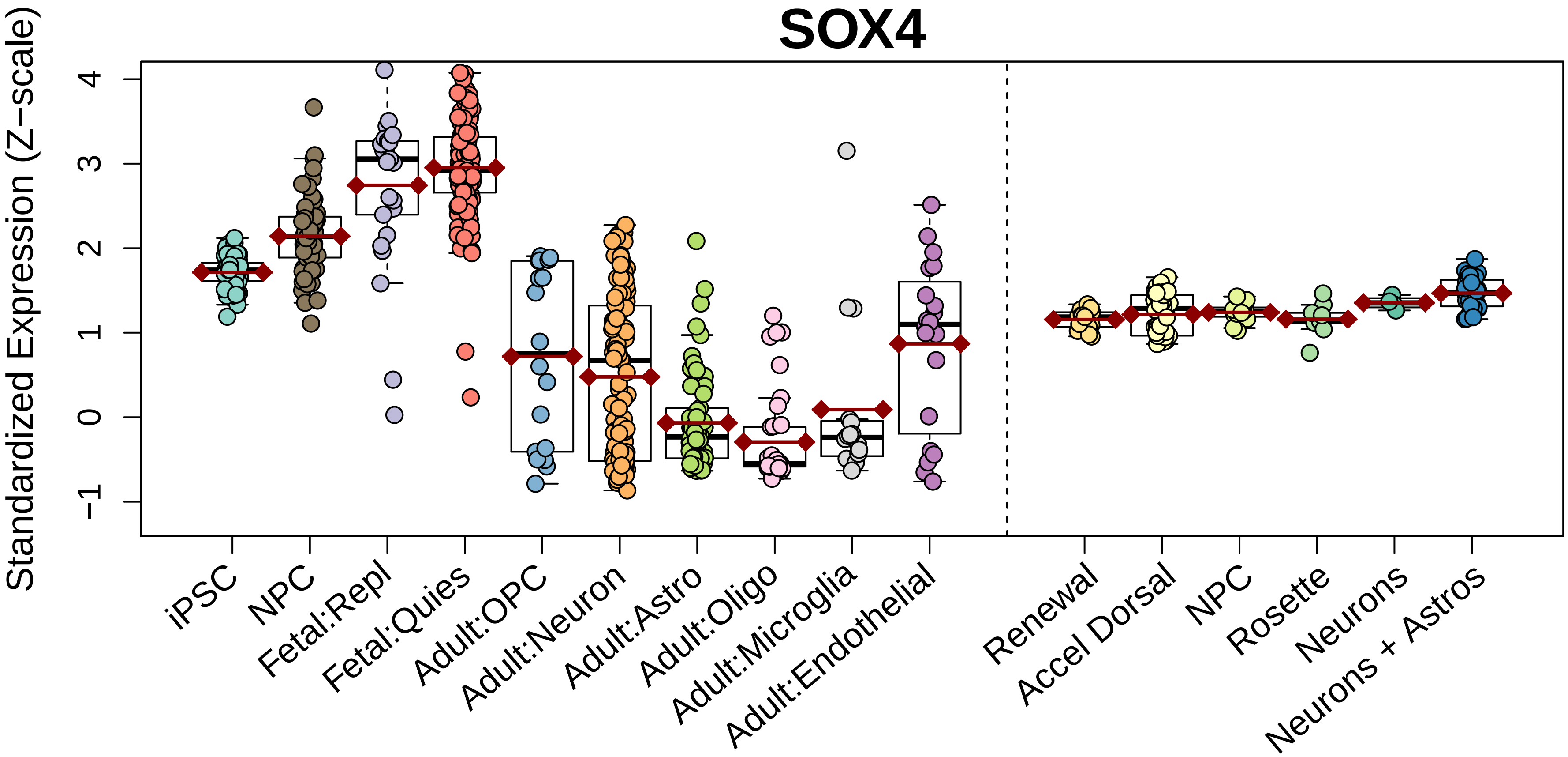
SOX4
Standardized Expression (Z−scale)
4
3
2
1
0
−1
iPSC
NPC
Fetal:Repl
Fetal:Quies
Adult:OPC
Adult:Neuron
Adult:Astro
Adult:Oligo
Adult:Microglia
Adult:Endothelial
Renewal
Accel Dorsal
NPC
Rosette
Neurons
Neurons + Astros

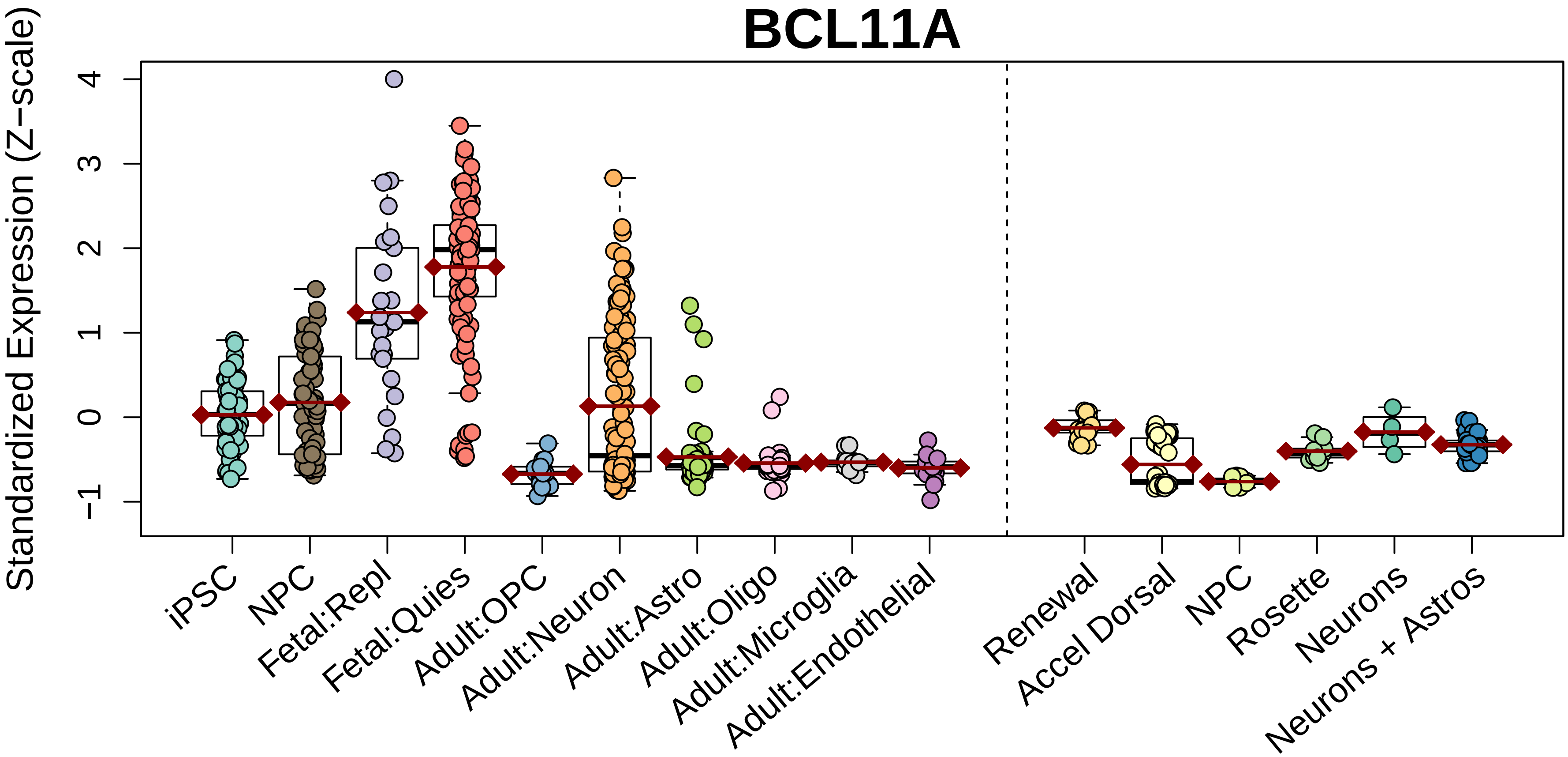

BCL11A
Standardized Expression (Z−scale)
4
3
2
1
0
−1
iPSC
NPC
Fetal:Repl
Fetal:Quies
Adult:OPC
Adult:Neuron
Adult:Astro
Adult:Oligo
Adult:Microglia
Adult:Endothelial
Renewal
Accel Dorsal
NPC
Rosette
Neurons
Neurons + Astros

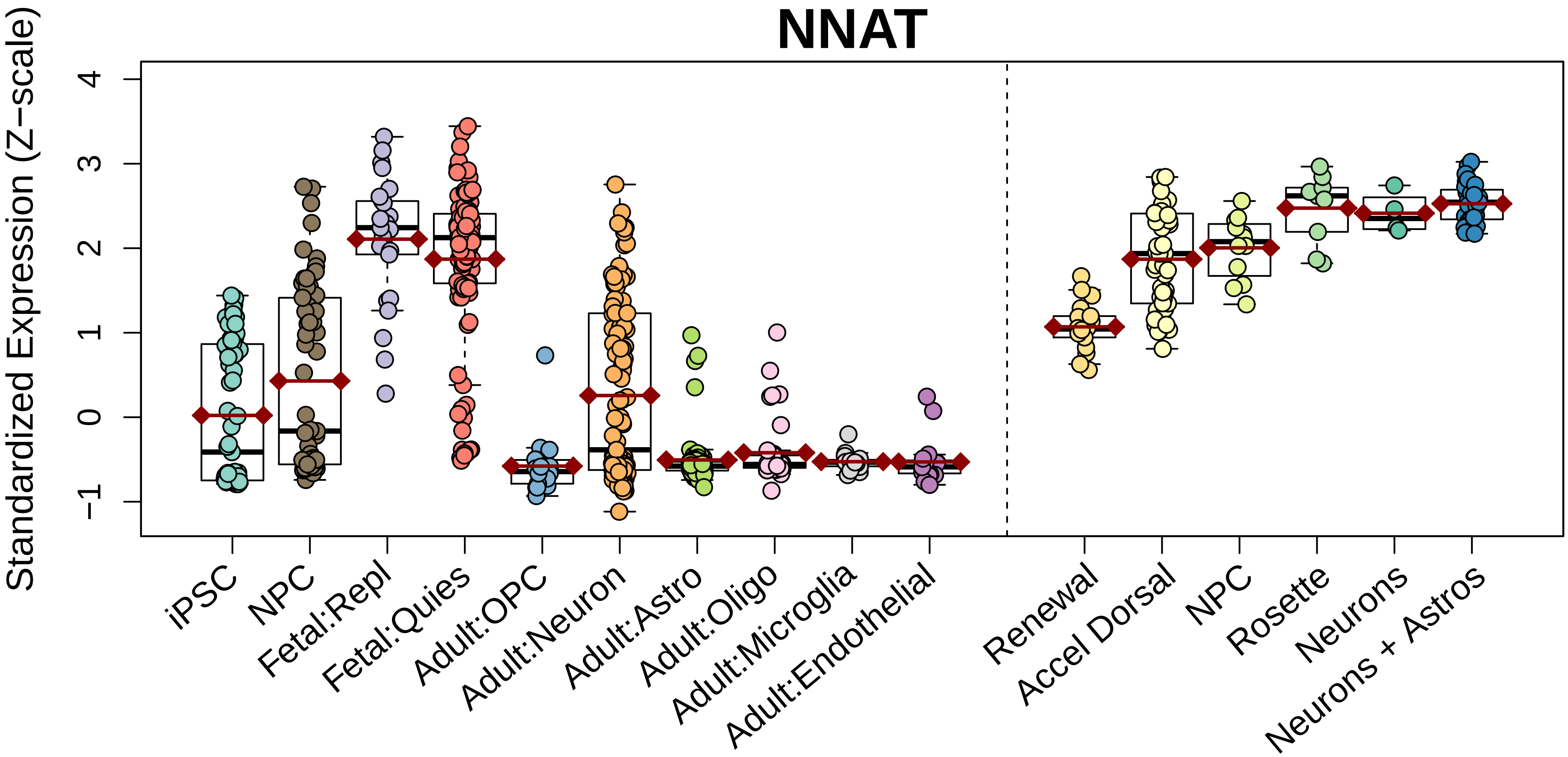

NNAT
Standardized Expression (Z−scale)
4
3
2
1
0
−1
iPSC
NPC
Fetal:Repl
Fetal:Quies
Adult:OPC
Adult:Neuron
Adult:Astro
Adult:Oligo
Adult:Microglia
Adult:Endothelial
Renewal
Accel Dorsal
NPC
Rosette
Neurons
Neurons + Astros

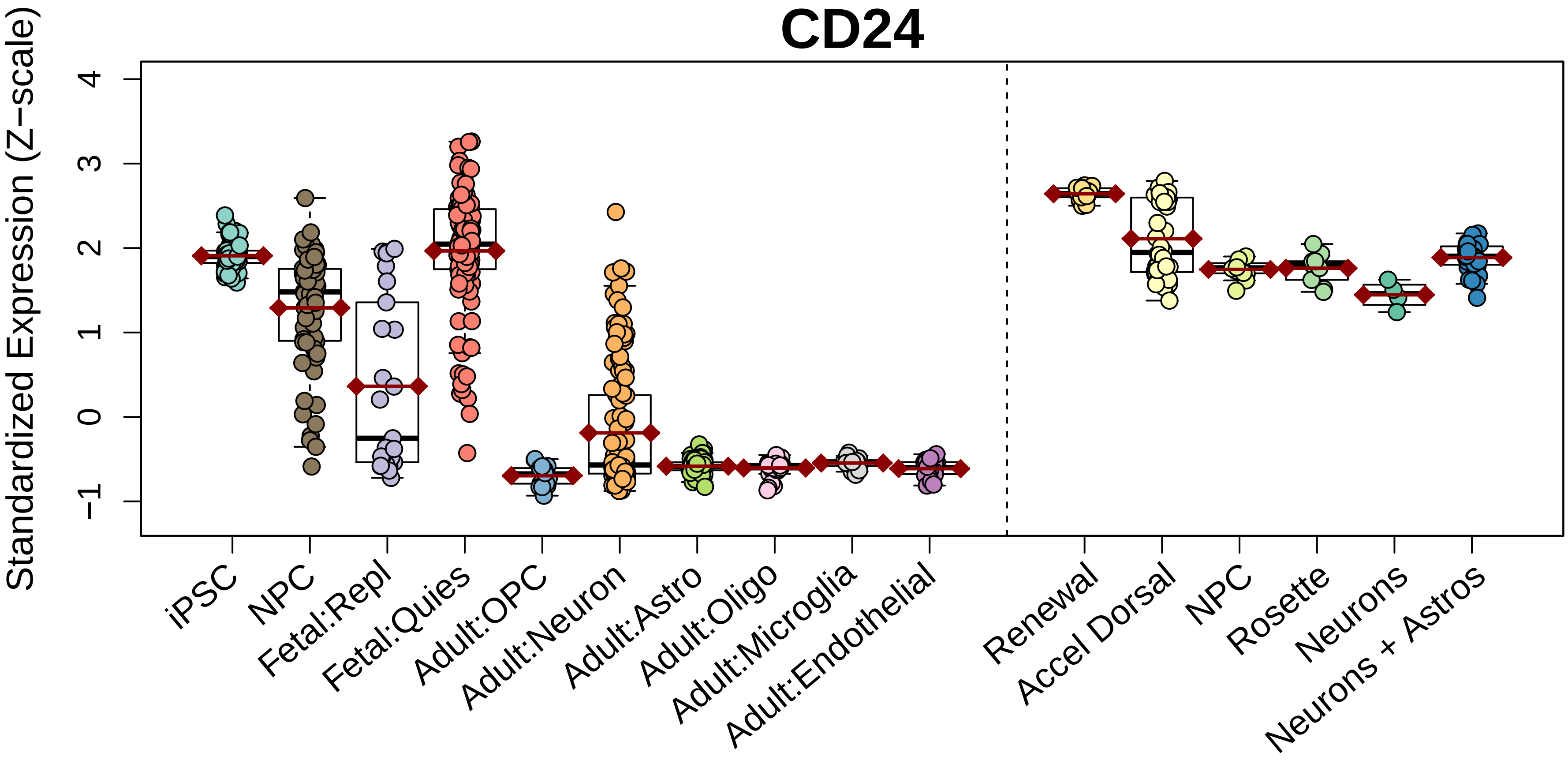

CD24
Standardized Expression (Z−scale)
4
3
2
1
0
−1
iPSC
NPC
Fetal:Repl
Fetal:Quies
Adult:OPC
Adult:Neuron
Adult:Astro
Adult:Oligo
Adult:Microglia
Adult:Endothelial
Renewal
Accel Dorsal
NPC
Rosette
Neurons
Neurons + Astros

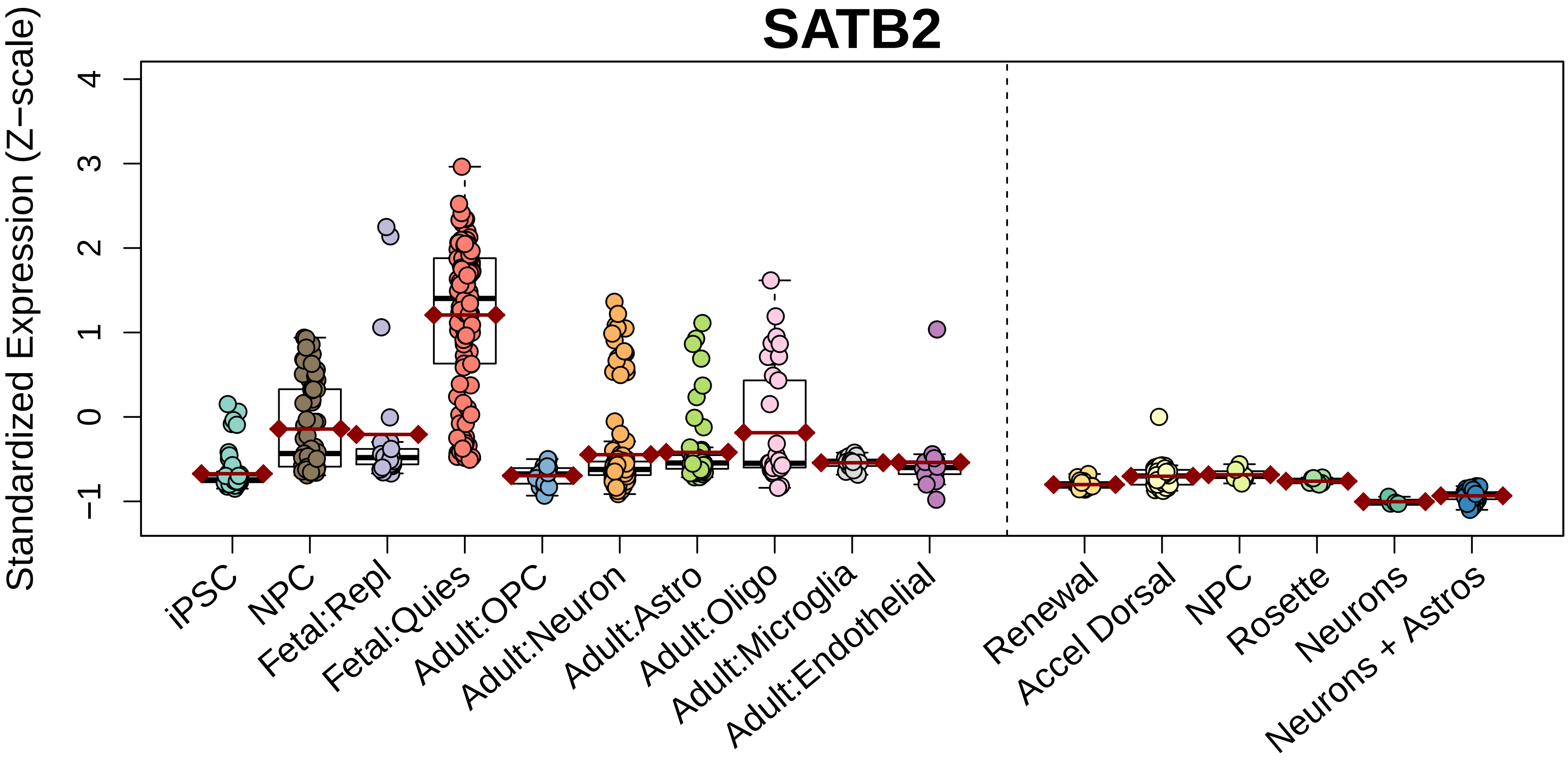

SATB2
Standardized Expression (Z−scale)
4
3
2
1
0
−1
iPSC
NPC
Fetal:Repl
Fetal:Quies
Adult:OPC
Adult:Neuron
Adult:Astro
Adult:Oligo
Adult:Microglia
Adult:Endothelial
Renewal
Accel Dorsal
NPC
Rosette
Neurons
Neurons + Astros

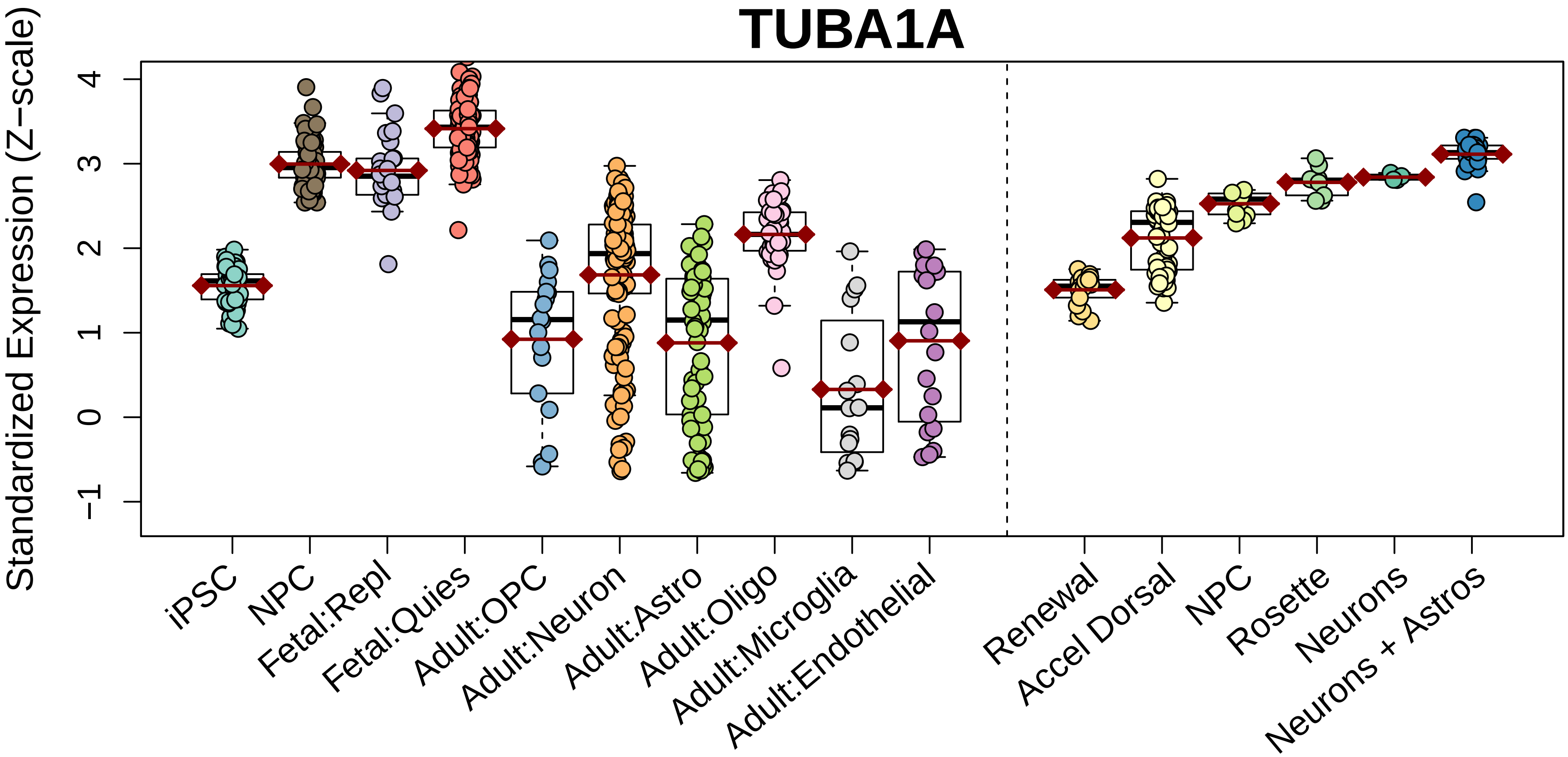
TUBA1A
Standardized Expression (Z−scale)
−1
0
1
2
3
4
iPSC
NPC
Fetal:Repl
Fetal:Quies
Adult:OPC
Adult:Neuron
Adult:Astro
Adult:Oligo
Adult:Microglia
Adult:Endothelial
Renewal
Accel Dorsal
NPC
Rosette
Neurons
Neurons + Astros

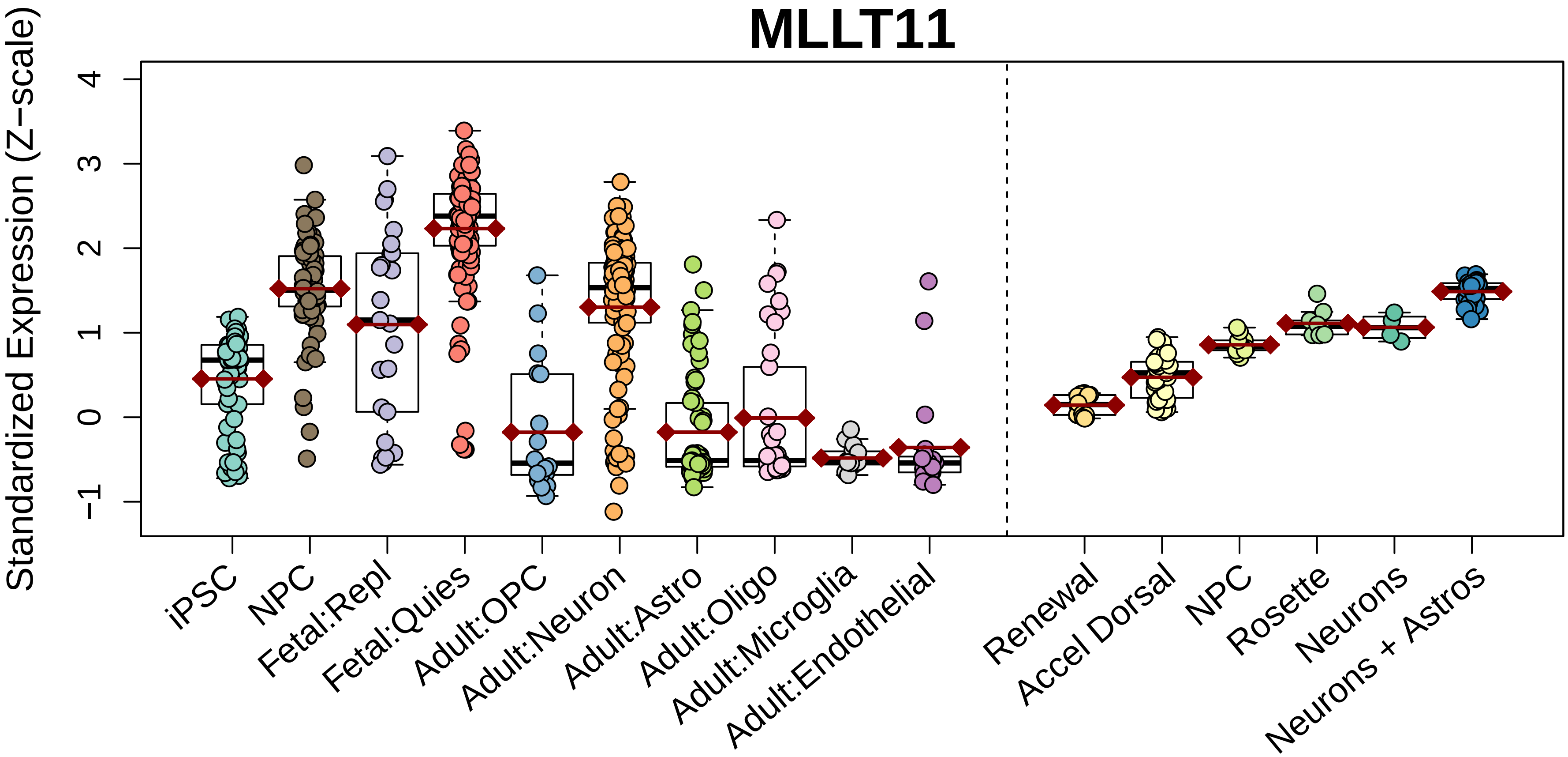
MLLT11
Standardized Expression (Z−scale)
−1
0
1
2
3
4
iPSC
NPC
Fetal:Repl
Fetal:Quies
Adult:OPC
Adult:Neuron
Adult:Astro
Adult:Oligo
Adult:Microglia
Adult:Endothelial
Renewal
Accel Dorsal
NPC
Rosette
Neurons
Neurons + Astros

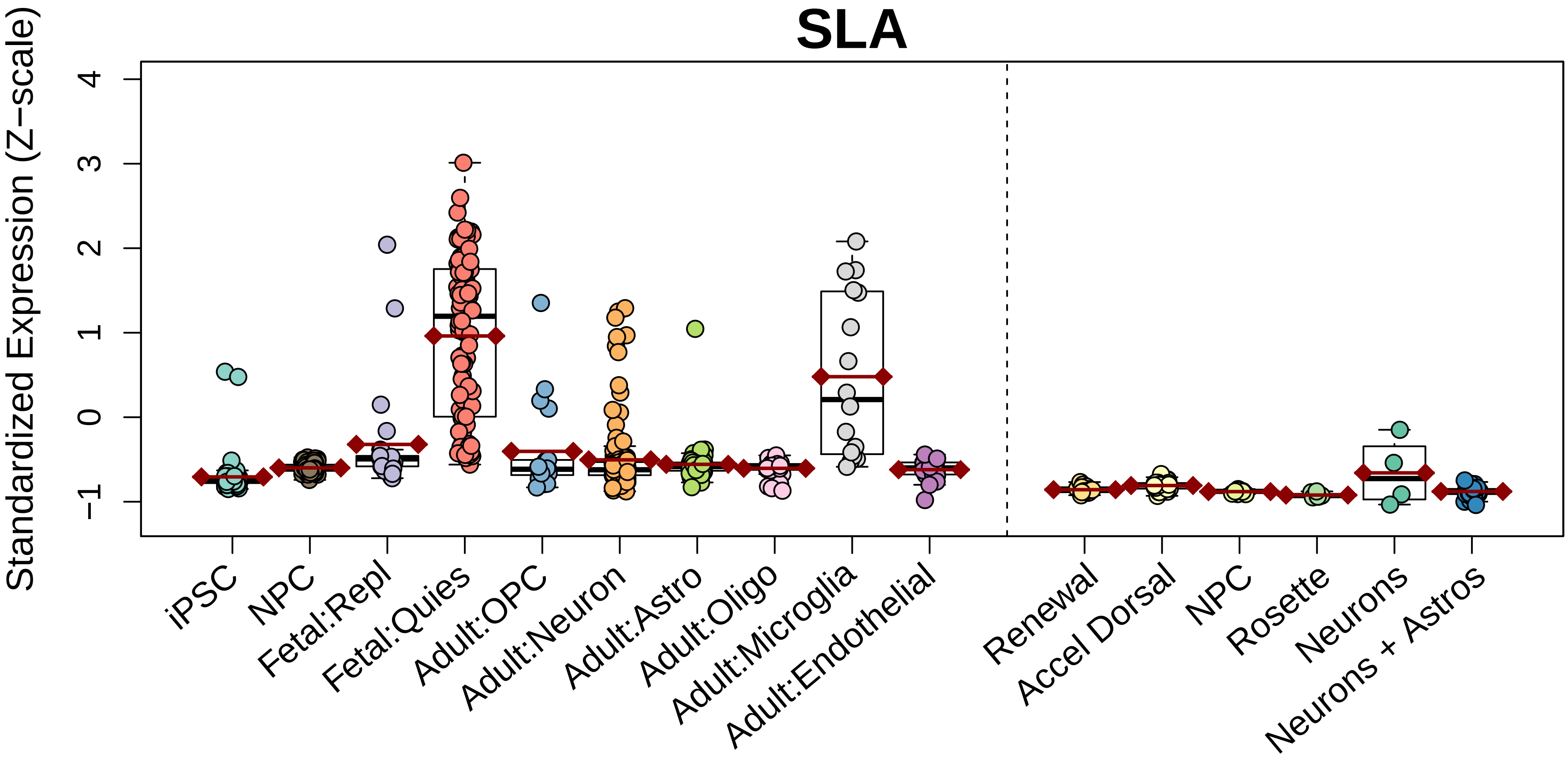

SLA
Standardized Expression (Z−scale)
4
3
2
1
0
−1
iPSC
NPC
Fetal:Repl
Fetal:Quies
Adult:OPC
Adult:Neuron
Adult:Astro
Adult:Oligo
Adult:Microglia
Adult:Endothelial
Renewal
Accel Dorsal
NPC
Rosette
Neurons
Neurons + Astros

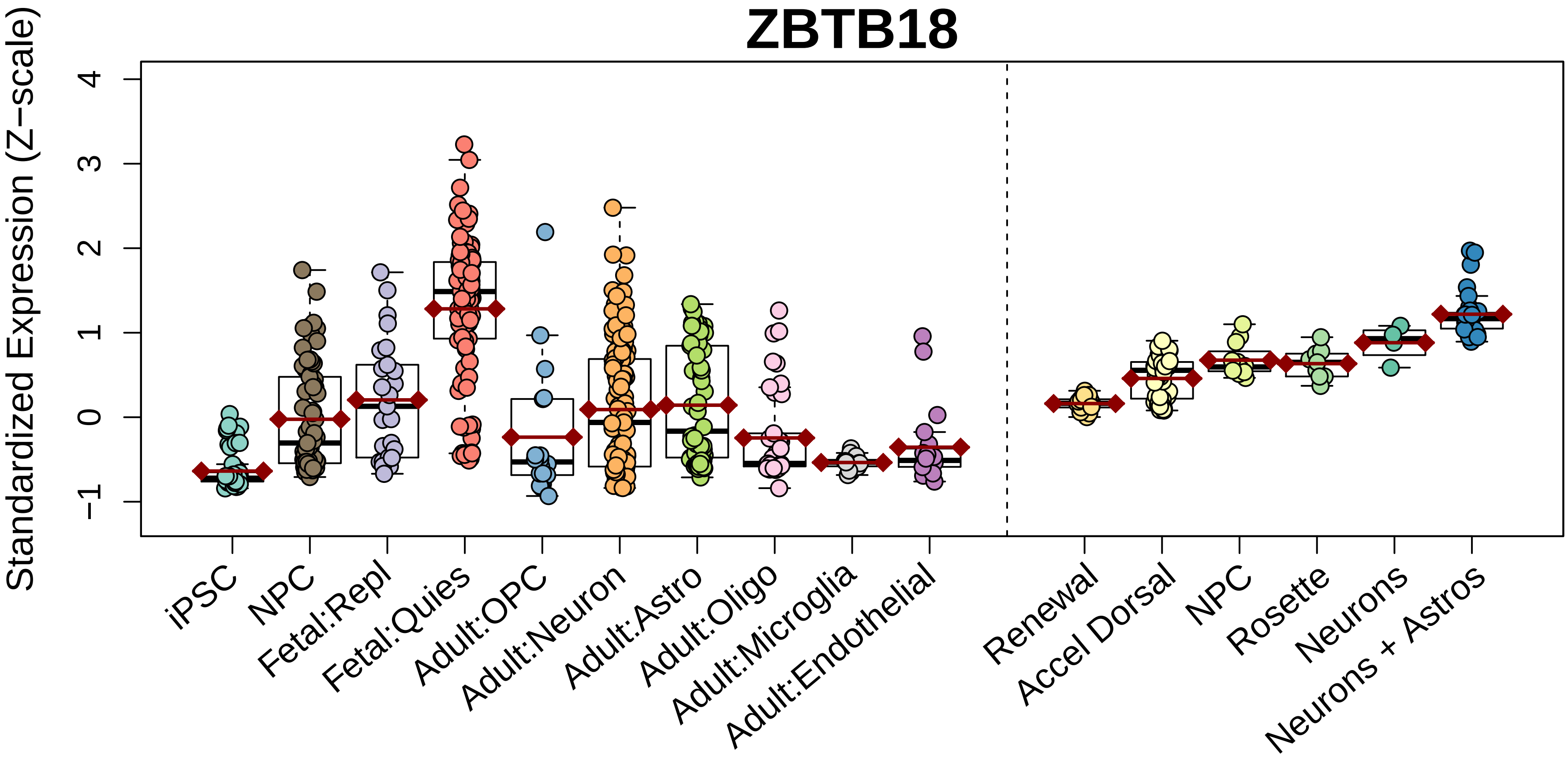

ZBTB18
Standardized Expression (Z−scale)
4
3
2
1
0
−1
iPSC
NPC
Fetal:Repl
Fetal:Quies
Adult:OPC
Adult:Neuron
Adult:Astro
Adult:Oligo
Adult:Microglia
Adult:Endothelial
Renewal
Accel Dorsal
NPC
Rosette
Neurons
Neurons + Astros

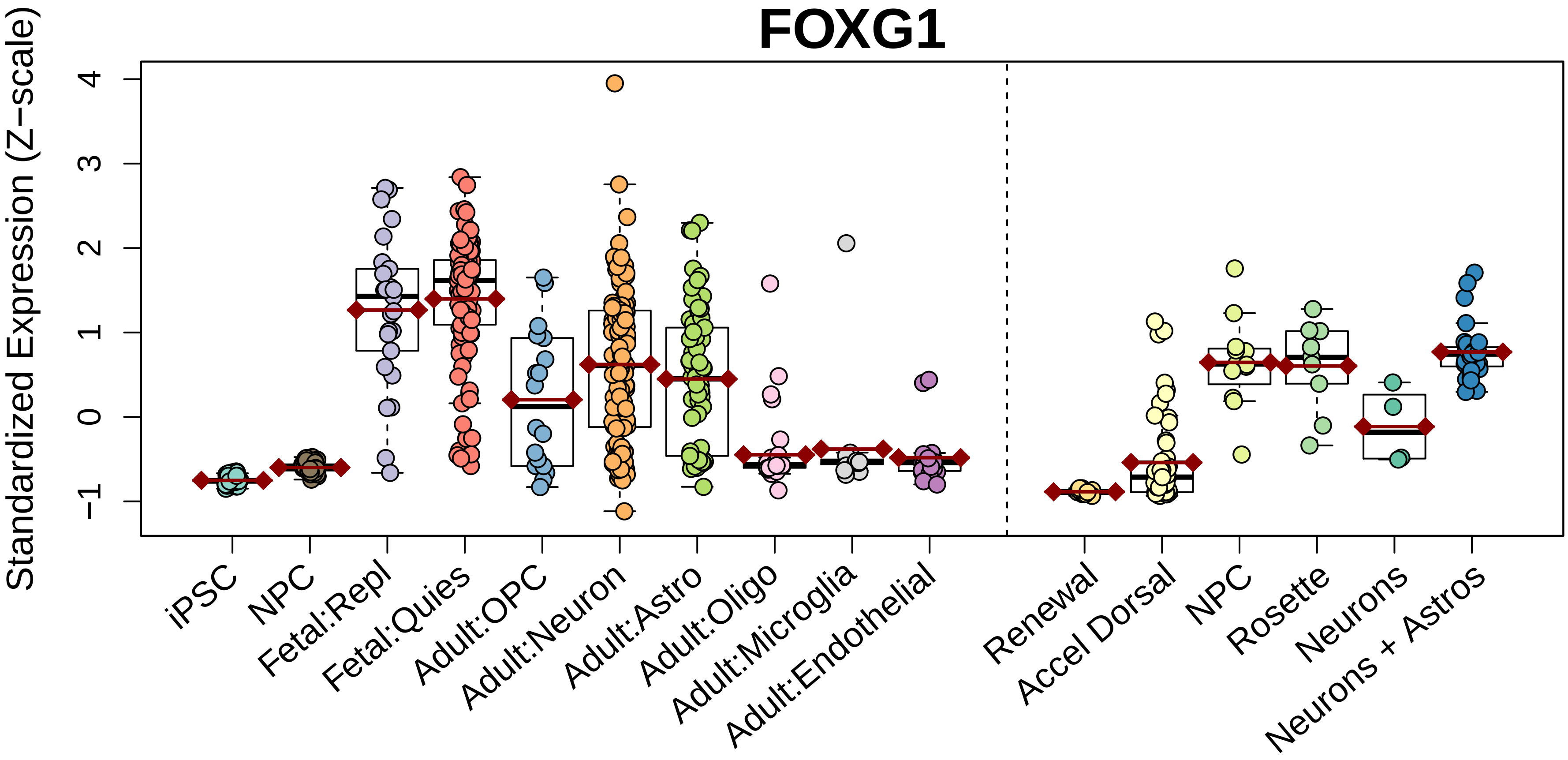
FOXG1
Standardized Expression (Z−scale)
4
3
2
1
0
−1
iPSC
NPC
Fetal:Repl
Fetal:Quies
Adult:OPC
Adult:Neuron
Adult:Astro
Adult:Oligo
Adult:Microglia
Adult:Endothelial
Renewal
Accel Dorsal
NPC
Rosette
Neurons
Neurons + Astros

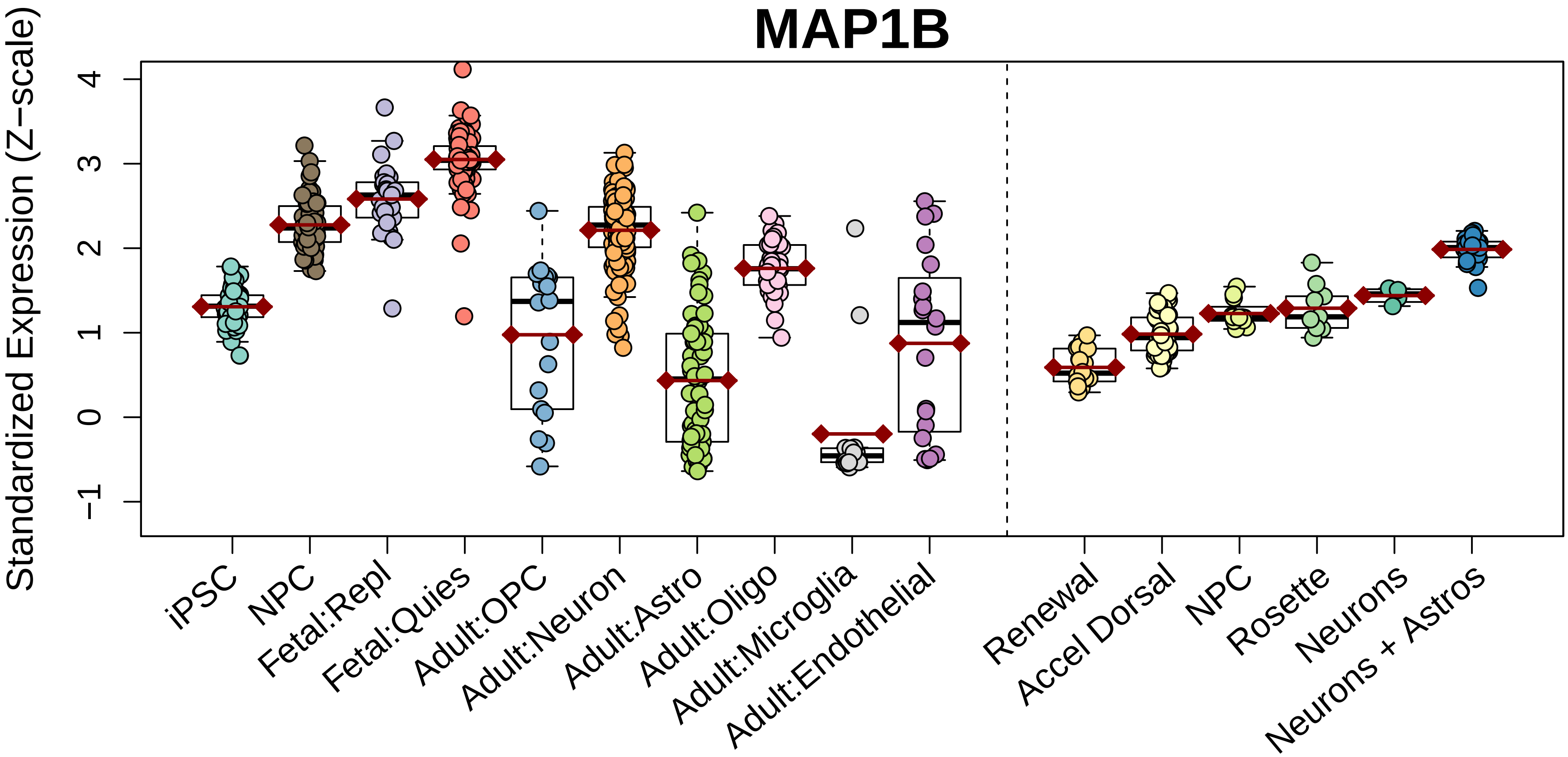
MAP1B
Standardized Expression (Z−scale)
4
3
2
1
0
−1
iPSC
NPC
Fetal:Repl
Fetal:Quies
Adult:OPC
Adult:Neuron
Adult:Astro
Adult:Oligo
Adult:Microglia
Adult:Endothelial
Renewal
Accel Dorsal
NPC
Rosette
Neurons
Neurons + Astros

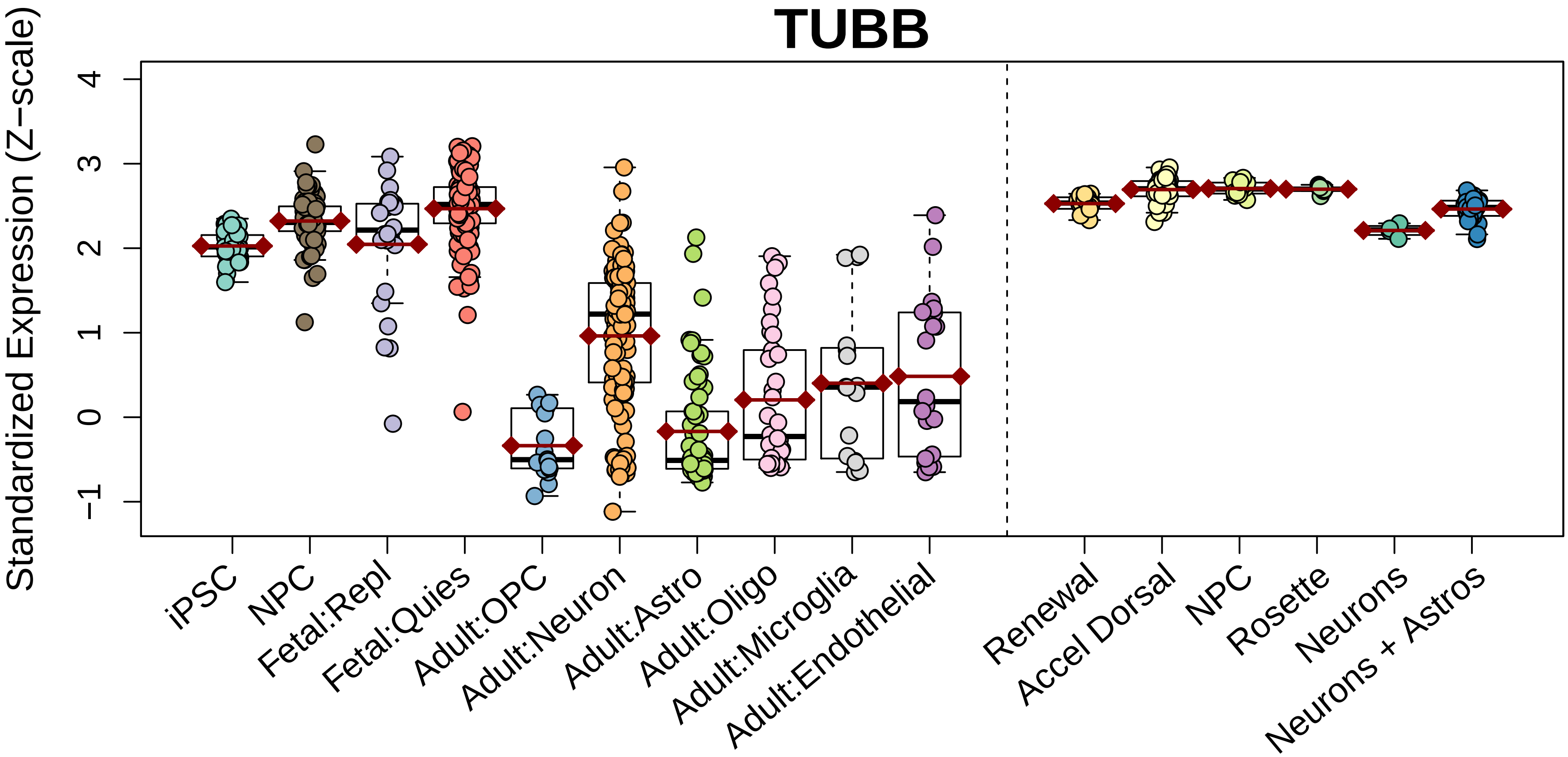

TUBB
Standardized Expression (Z−scale)
−1
0
1
2
3
4
iPSC
NPC
Fetal:Repl
Fetal:Quies
Adult:OPC
Adult:Neuron
Adult:Astro
Adult:Oligo
Adult:Microglia
Adult:Endothelial
Renewal
Accel Dorsal
NPC
Rosette
Neurons
Neurons + Astros

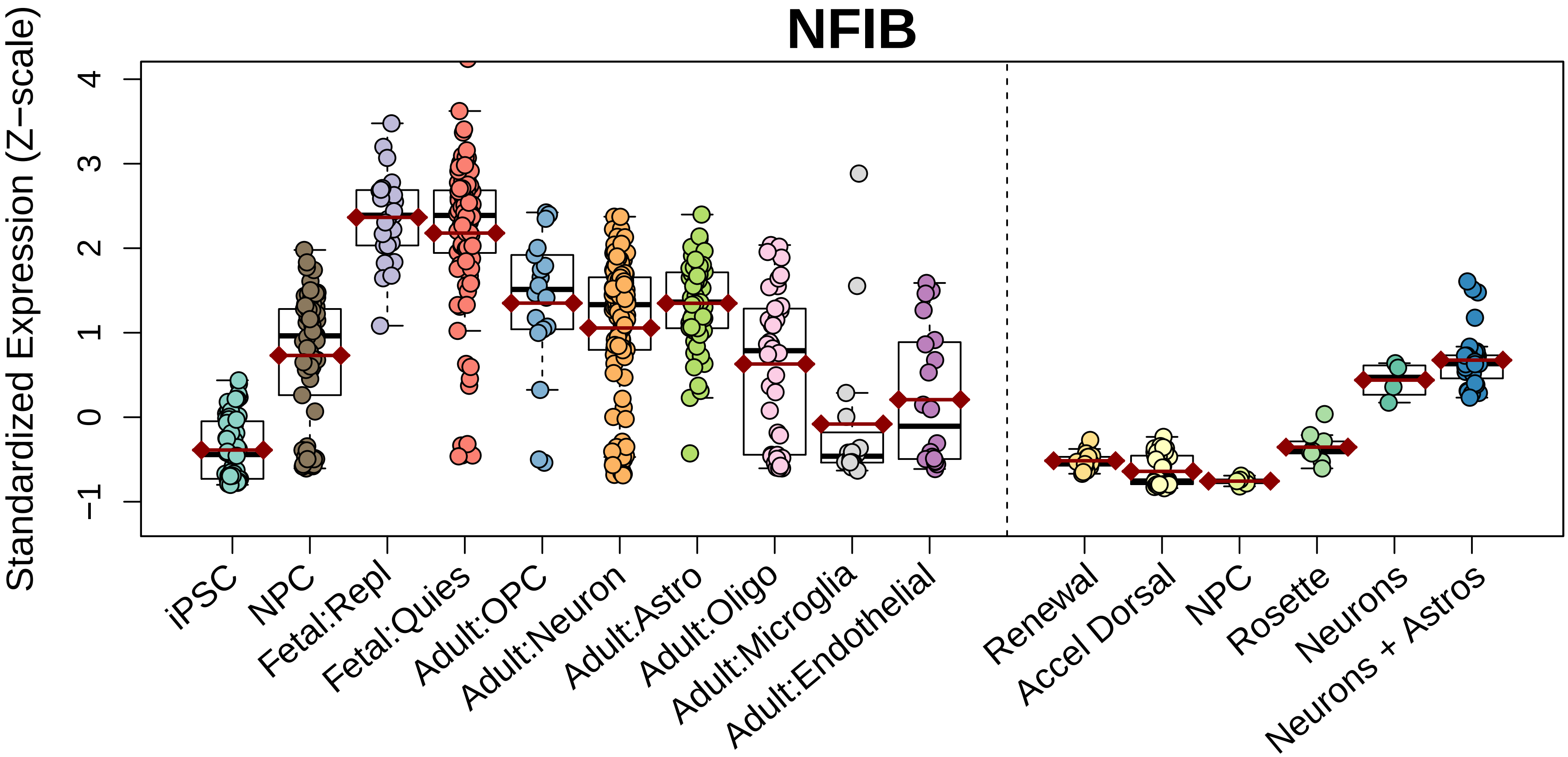

NFIB
Standardized Expression (Z−scale)
−1
0
1
2
3
4
iPSC
NPC
Fetal:Repl
Fetal:Quies
Adult:OPC
Adult:Neuron
Adult:Astro
Adult:Oligo
Adult:Microglia
Adult:Endothelial
Renewal
Accel Dorsal
NPC
Rosette
Neurons
Neurons + Astros

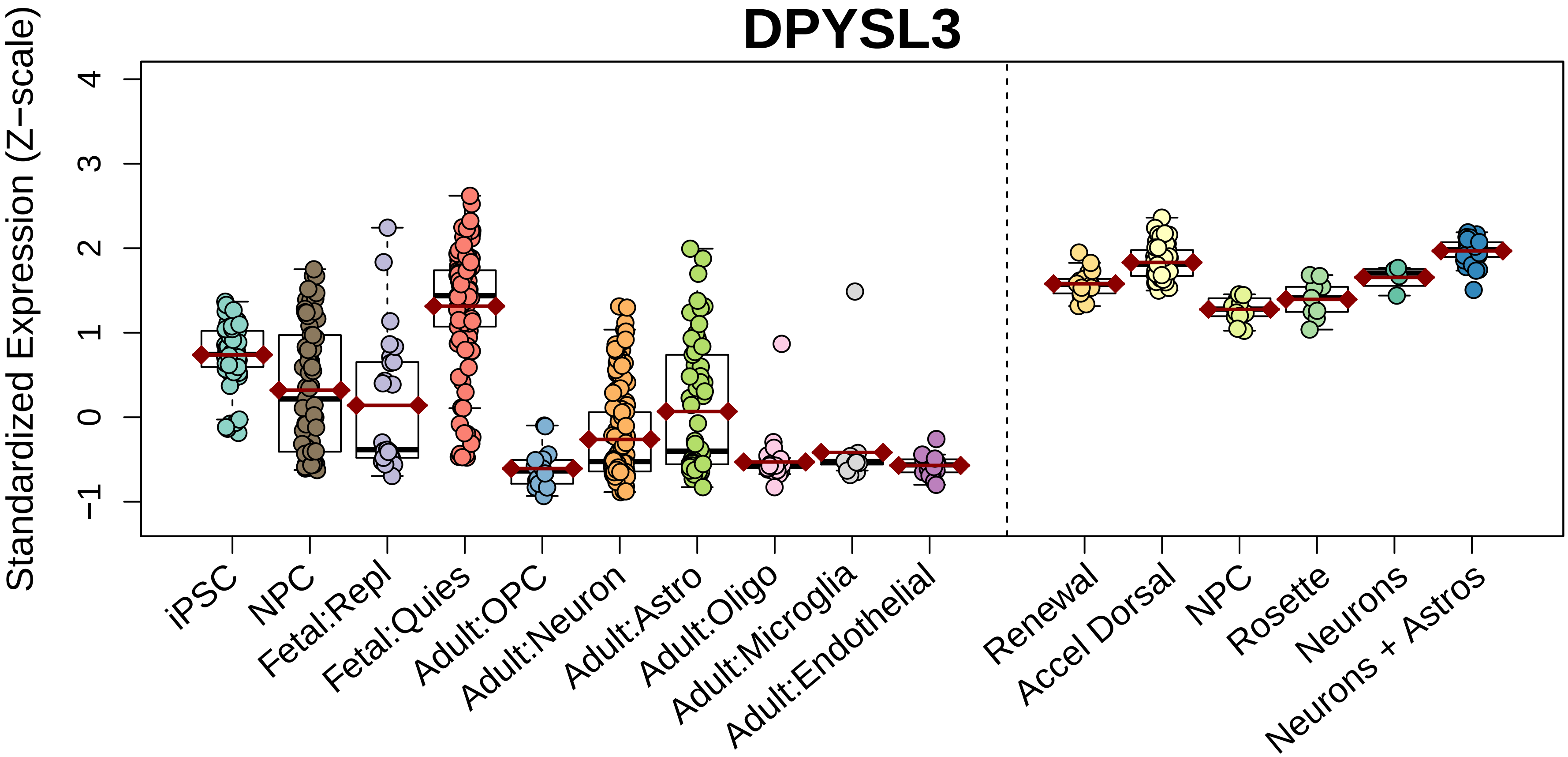

DPYSL3
Standardized Expression (Z−scale)
4
3
2
1
0
−1
iPSC
NPC
Fetal:Repl
Fetal:Quies
Adult:OPC
Adult:Neuron
Adult:Astro
Adult:Oligo
Adult:Microglia
Adult:Endothelial
Renewal
Accel Dorsal
NPC
Rosette
Neurons
Neurons + Astros

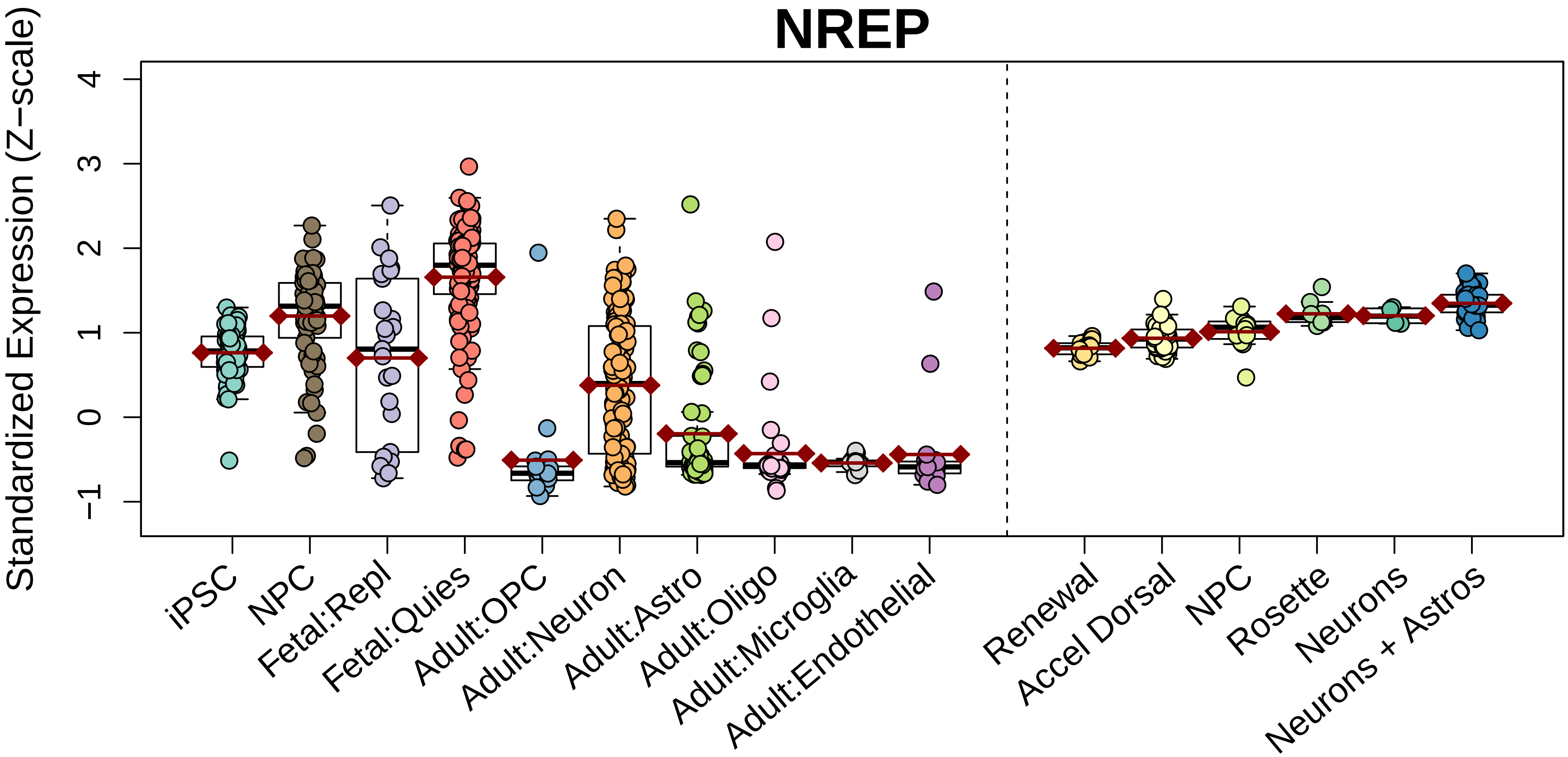

NREP
Standardized Expression (Z−scale)
4
3
2
1
0
−1
iPSC
NPC
Fetal:Repl
Fetal:Quies
Adult:OPC
Adult:Neuron
Adult:Astro
Adult:Oligo
Adult:Microglia
Adult:Endothelial
Renewal
Accel Dorsal
NPC
Rosette
Neurons
Neurons + Astros

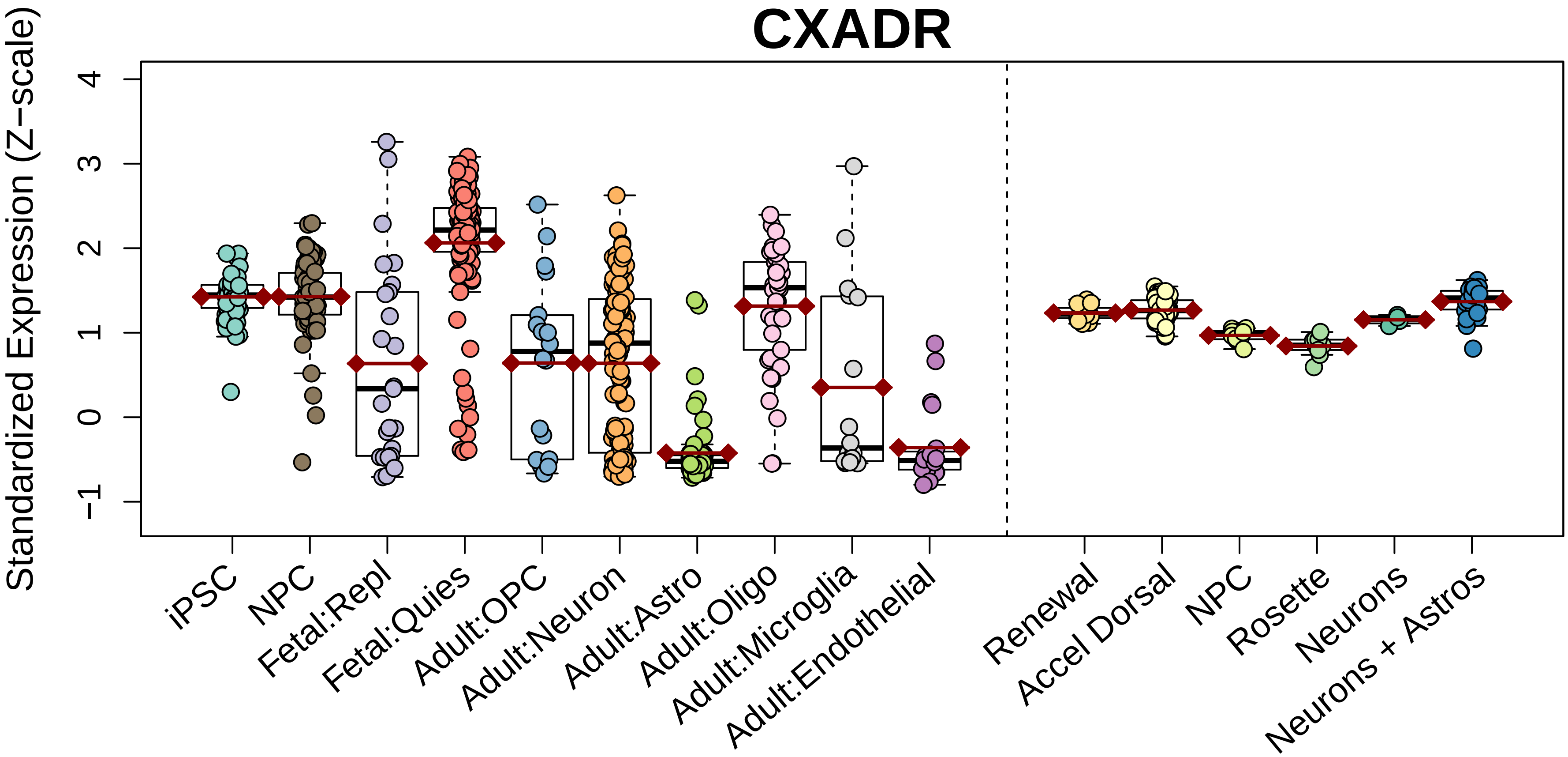

CXADR
Standardized Expression (Z−scale)
4
3
2
1
0
−1
iPSC
NPC
Fetal:Repl
Fetal:Quies
Adult:OPC
Adult:Neuron
Adult:Astro
Adult:Oligo
Adult:Microglia
Adult:Endothelial
Renewal
Accel Dorsal
NPC
Rosette
Neurons
Neurons + Astros

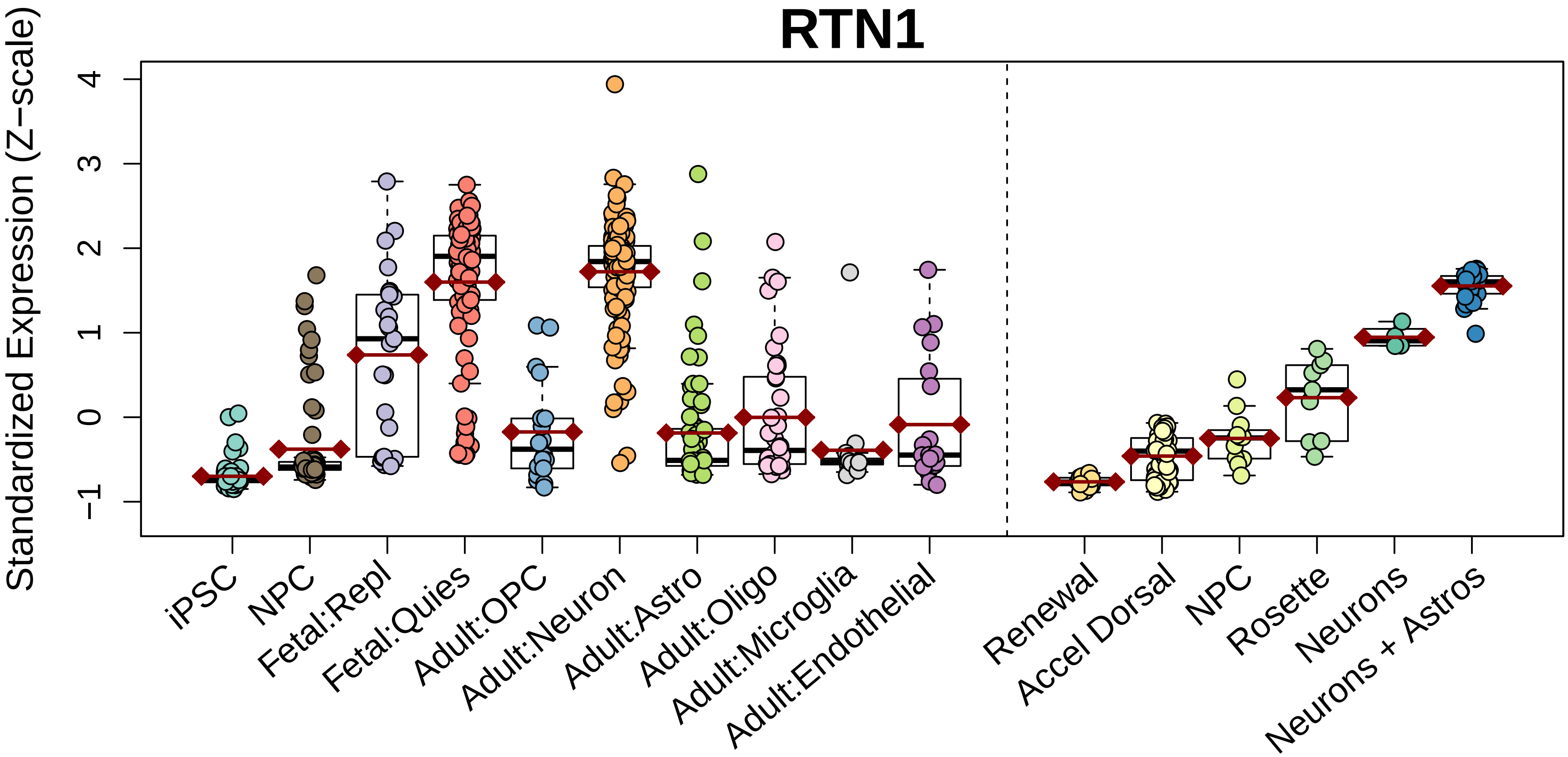

RTN1
Standardized Expression (Z−scale)
4
3
2
1
0
−1
iPSC
NPC
Fetal:Repl
Fetal:Quies
Adult:OPC
Adult:Neuron
Adult:Astro
Adult:Oligo
Adult:Microglia
Adult:Endothelial
Renewal
Accel Dorsal
NPC
Rosette
Neurons
Neurons + Astros

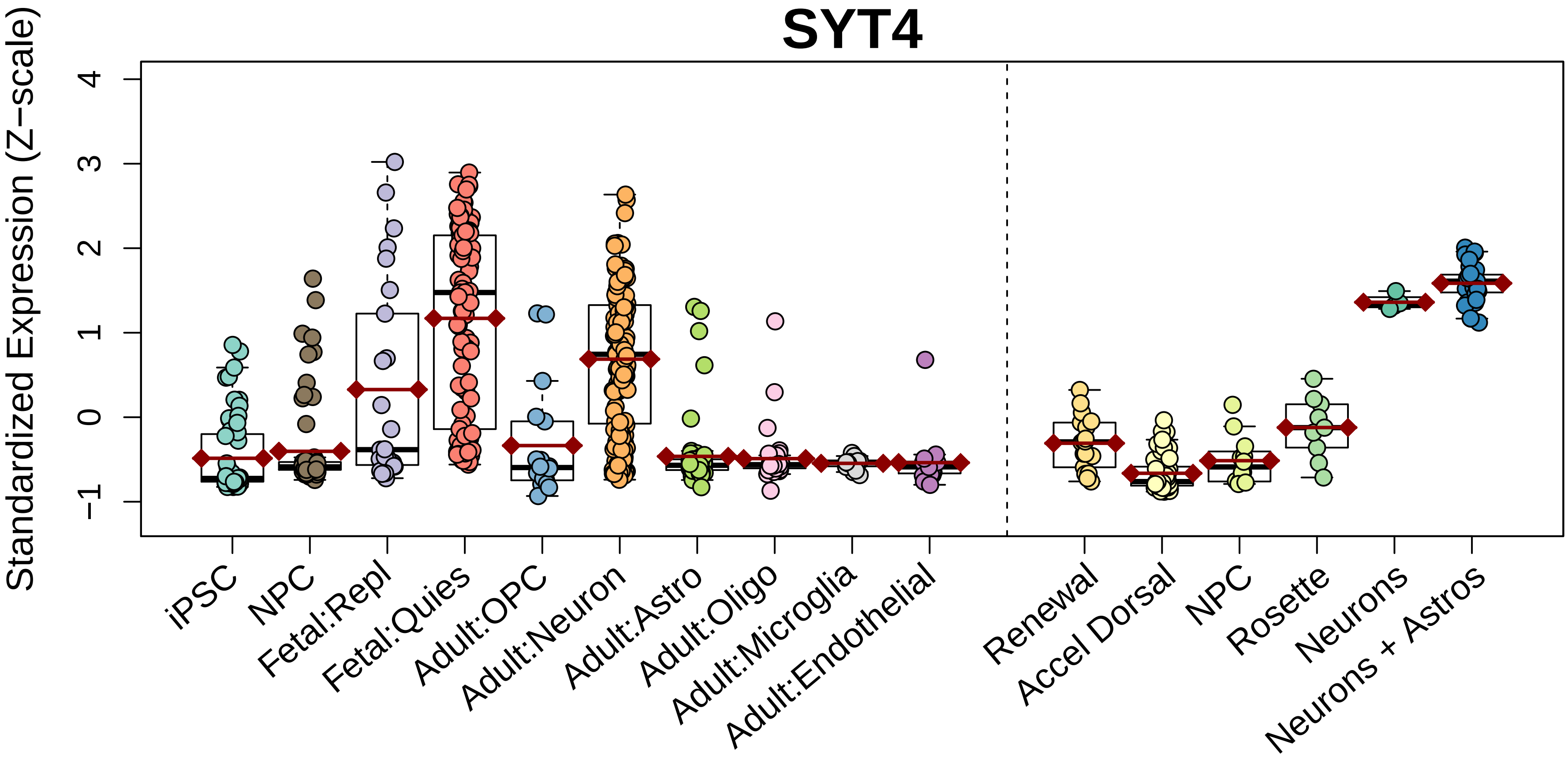

SYT4
Standardized Expression (Z−scale)
4
3
2
1
0
−1
iPSC
NPC
Fetal:Repl
Fetal:Quies
Adult:OPC
Adult:Neuron
Adult:Astro
Adult:Oligo
Adult:Microglia
Adult:Endothelial
Renewal
Accel Dorsal
NPC
Rosette
Neurons
Neurons + Astros

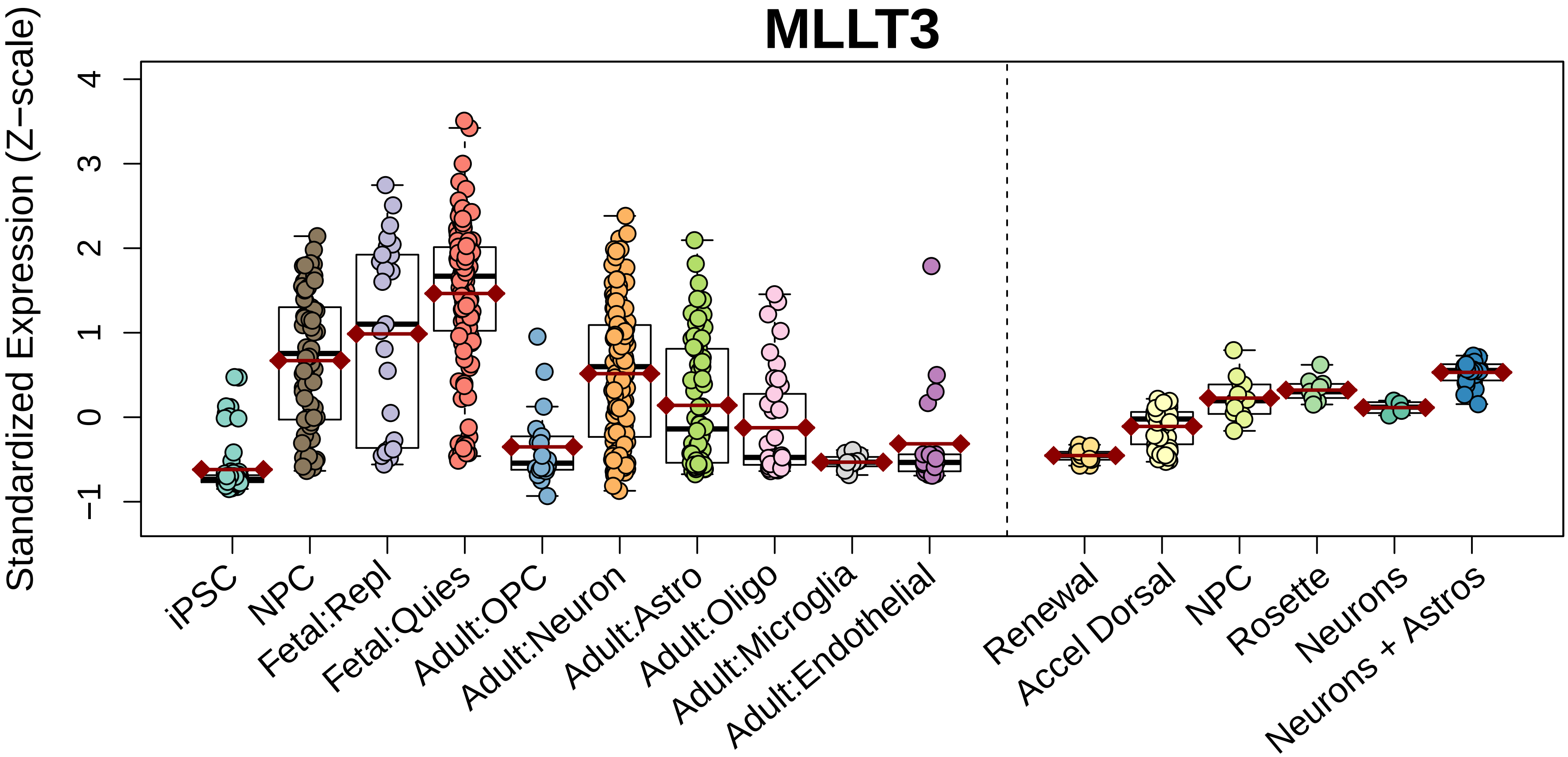

MLLT3
Standardized Expression (Z−scale)
4
3
2
1
0
−1
iPSC
NPC
Fetal:Repl
Fetal:Quies
Adult:OPC
Adult:Neuron
Adult:Astro
Adult:Oligo
Adult:Microglia
Adult:Endothelial
Renewal
Accel Dorsal
NPC
Rosette
Neurons
Neurons + Astros

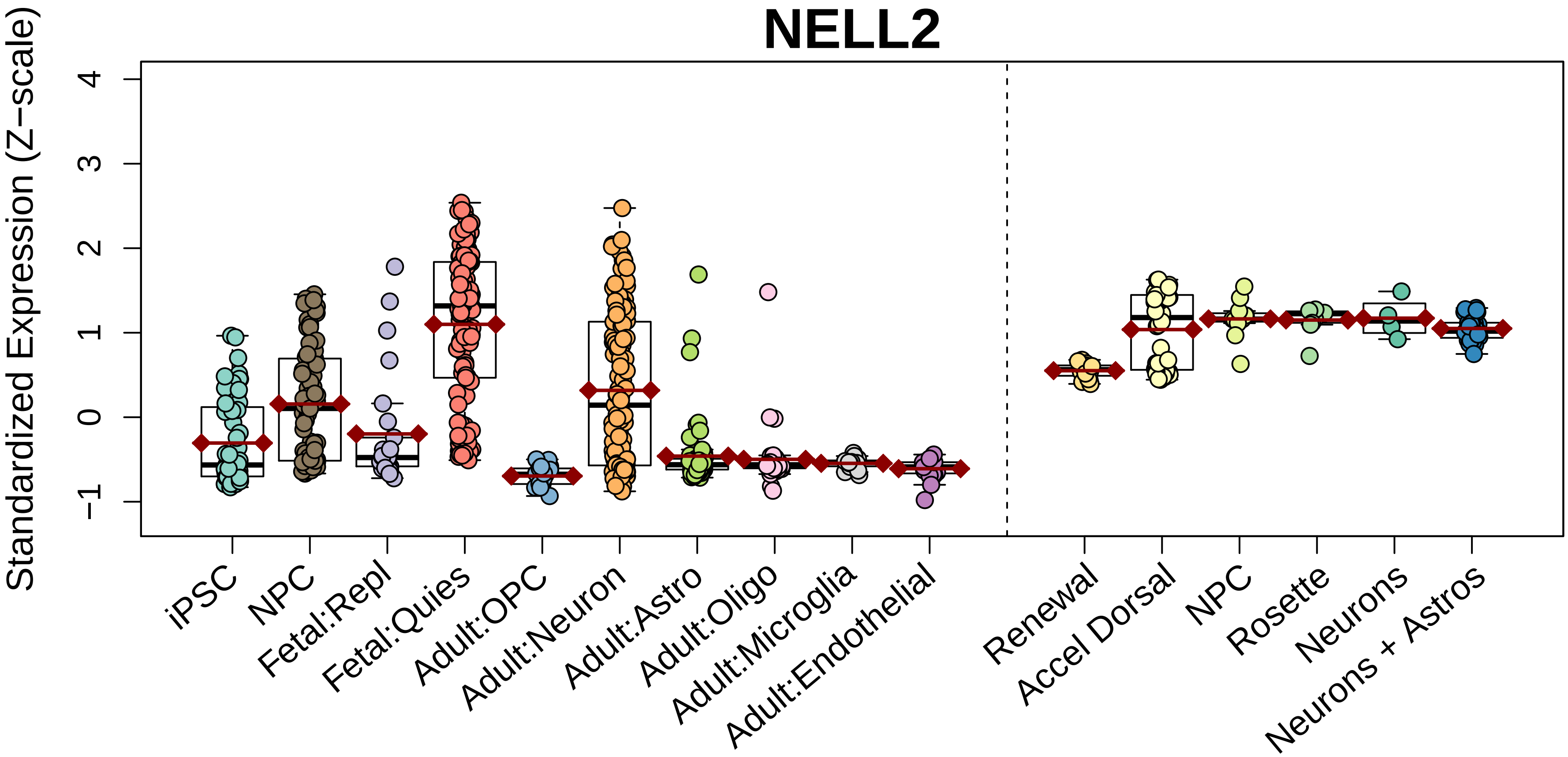

NELL2
Standardized Expression (Z−scale)
4
3
2
1
0
−1
iPSC
NPC
Fetal:Repl
Fetal:Quies
Adult:OPC
Adult:Neuron
Adult:Astro
Adult:Oligo
Adult:Microglia
Adult:Endothelial
Renewal
Accel Dorsal
NPC
Rosette
Neurons
Neurons + Astros

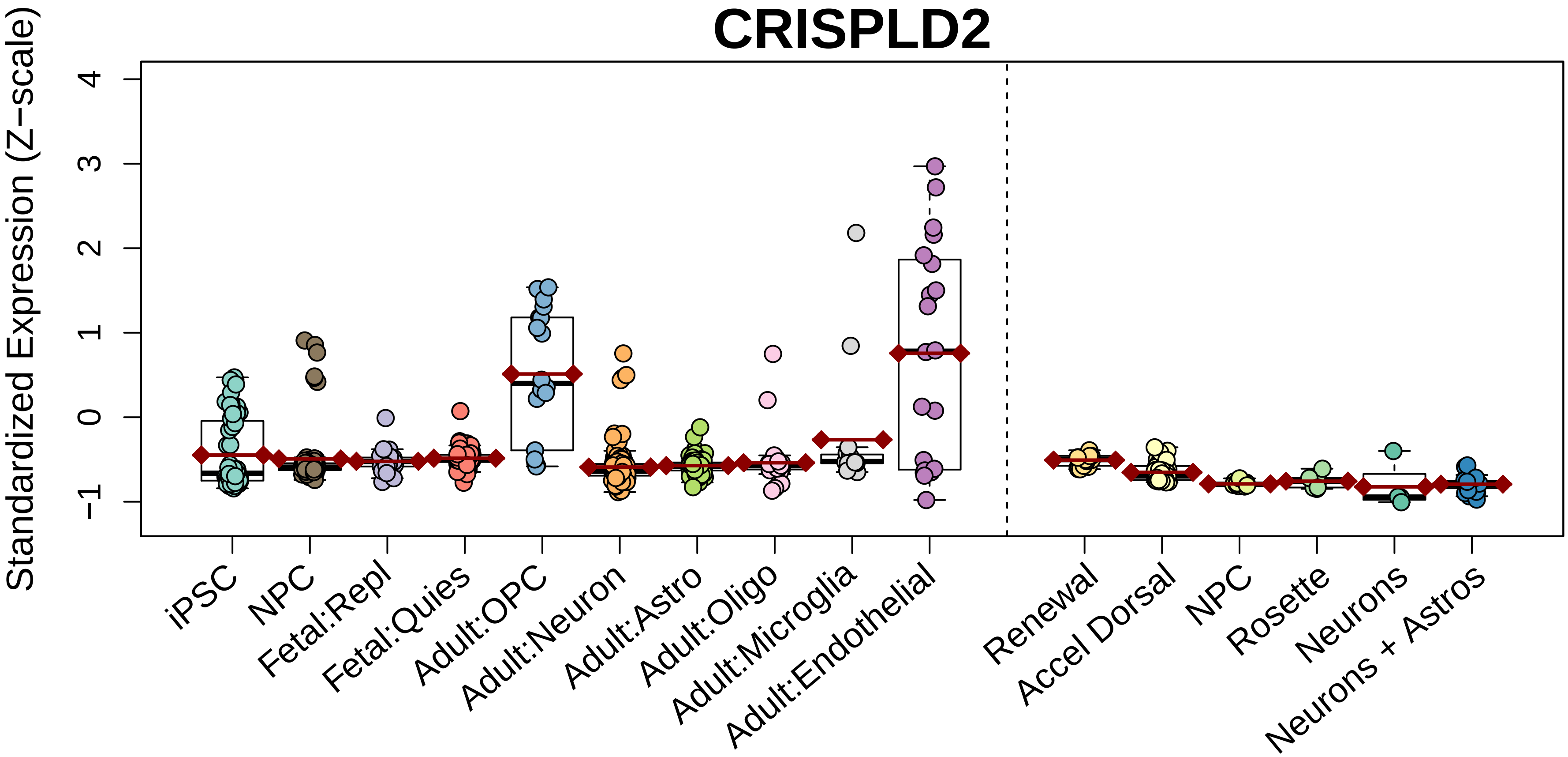

CRISPLD2
Standardized Expression (Z−scale)
4
3
2
1
0
−1
iPSC
NPC
Fetal:Repl
Fetal:Quies
Adult:OPC
Adult:Neuron
Adult:Astro
Adult:Oligo
Adult:Microglia
Adult:Endothelial
Renewal
Accel Dorsal
NPC
Rosette
Neurons
Neurons + Astros

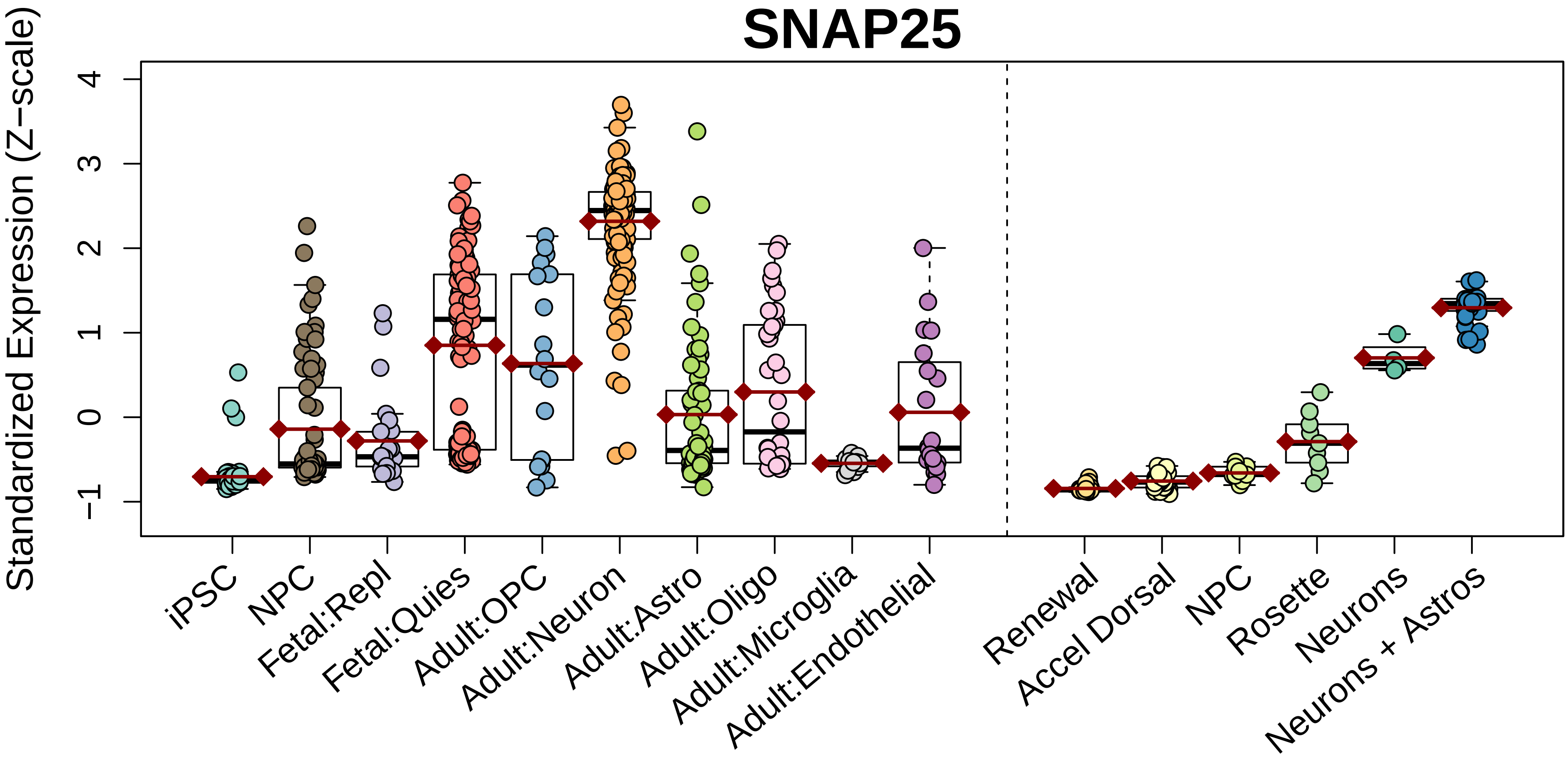
SNAP25
Standardized Expression (Z−scale)
4
3
2
1
0
−1
iPSC
NPC
Fetal:Repl
Fetal:Quies
Adult:OPC
Adult:Neuron
Adult:Astro
Adult:Oligo
Adult:Microglia
Adult:Endothelial
Renewal
Accel Dorsal
NPC
Rosette
Neurons
Neurons + Astros

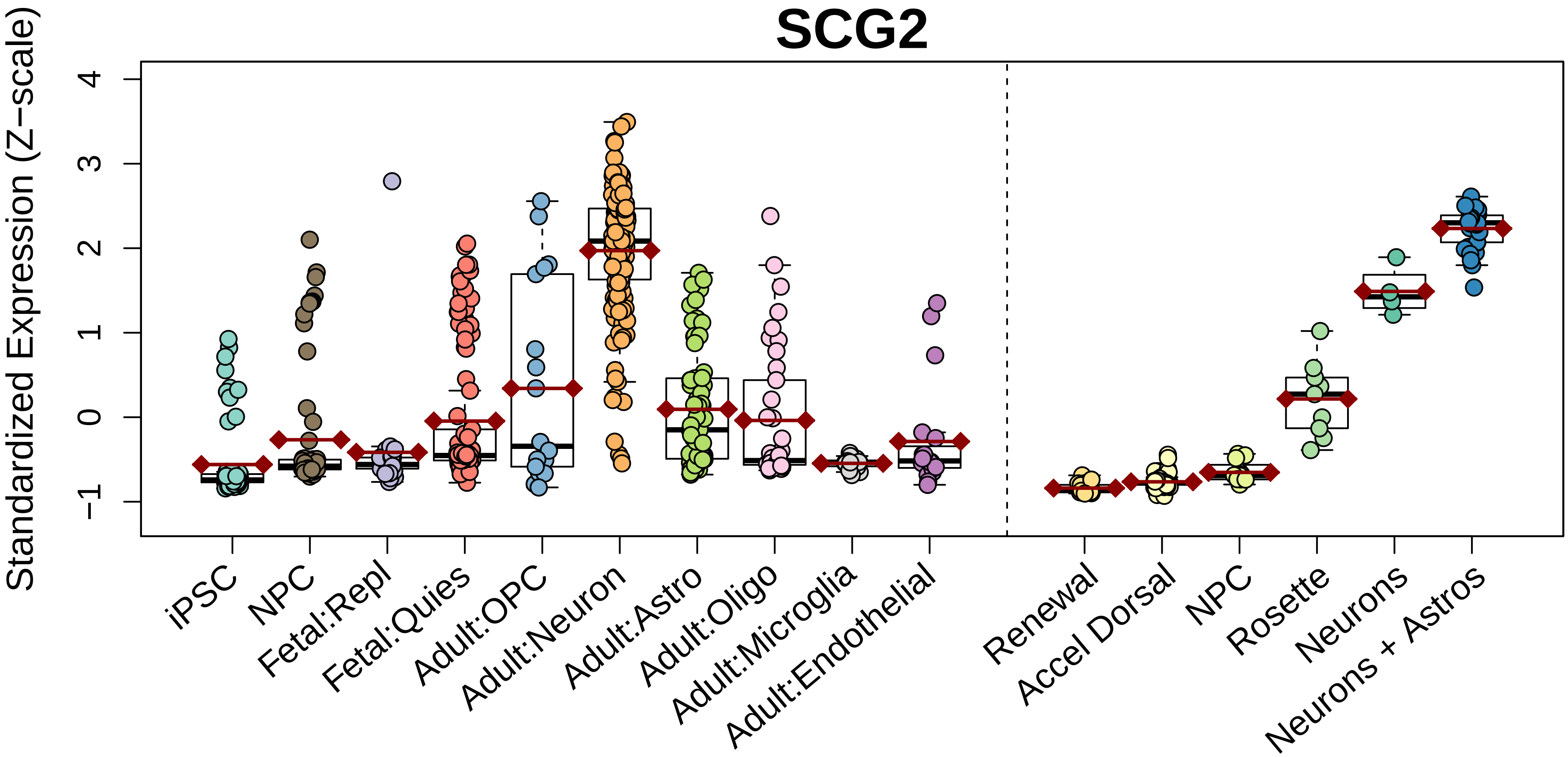
SCG2
Standardized Expression (Z−scale)
4
3
2
1
0
−1
iPSC
NPC
Fetal:Repl
Fetal:Quies
Adult:OPC
Adult:Neuron
Adult:Astro
Adult:Oligo
Adult:Microglia
Adult:Endothelial
Renewal
Accel Dorsal
NPC
Rosette
Neurons
Neurons + Astros

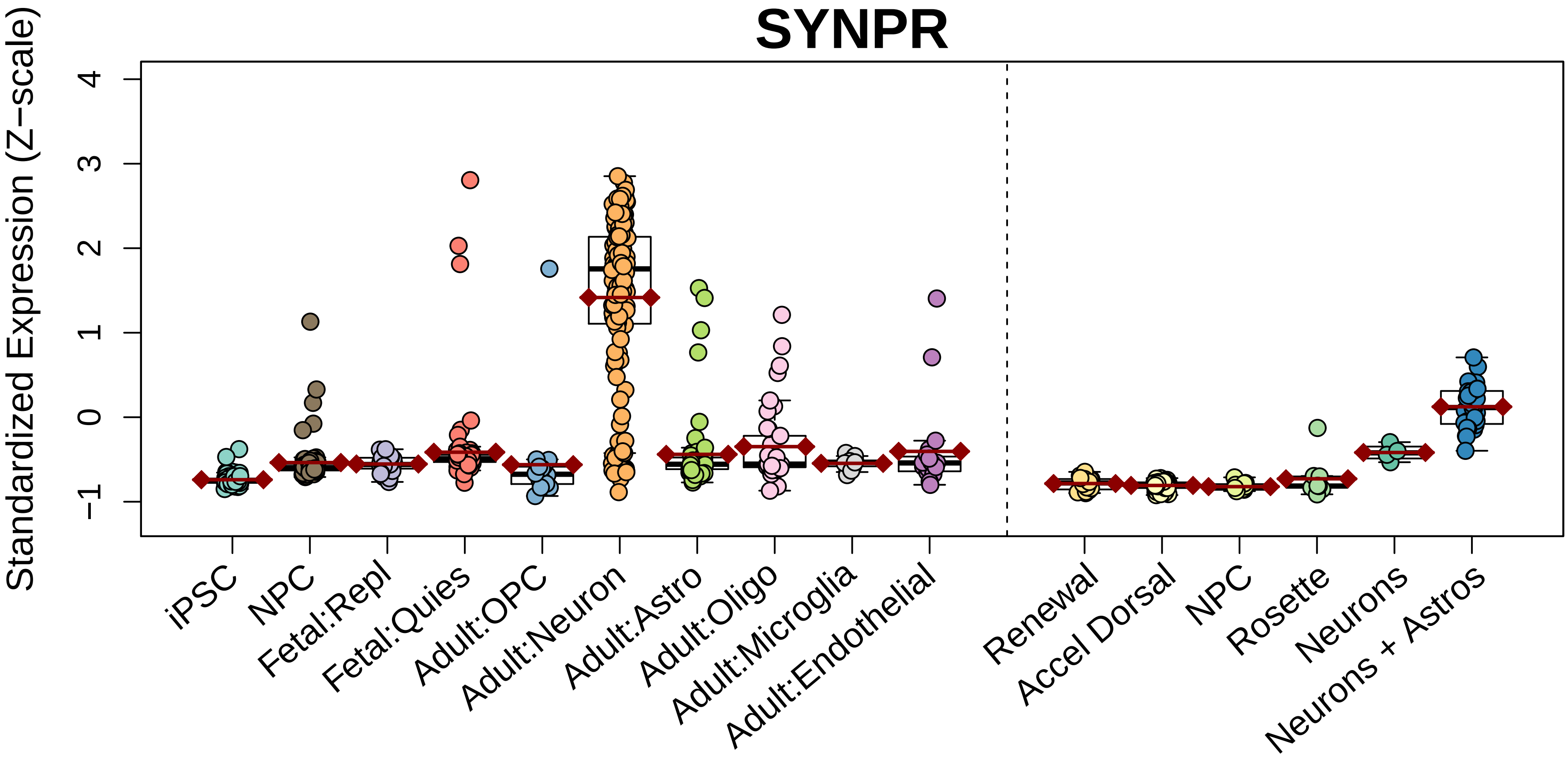
SYNPR
Standardized Expression (Z−scale)
4
3
2
1
0
−1
iPSC
NPC
Fetal:Repl
Fetal:Quies
Adult:OPC
Adult:Neuron
Adult:Astro
Adult:Oligo
Adult:Microglia
Adult:Endothelial
Renewal
Accel Dorsal
NPC
Rosette
Neurons
Neurons + Astros

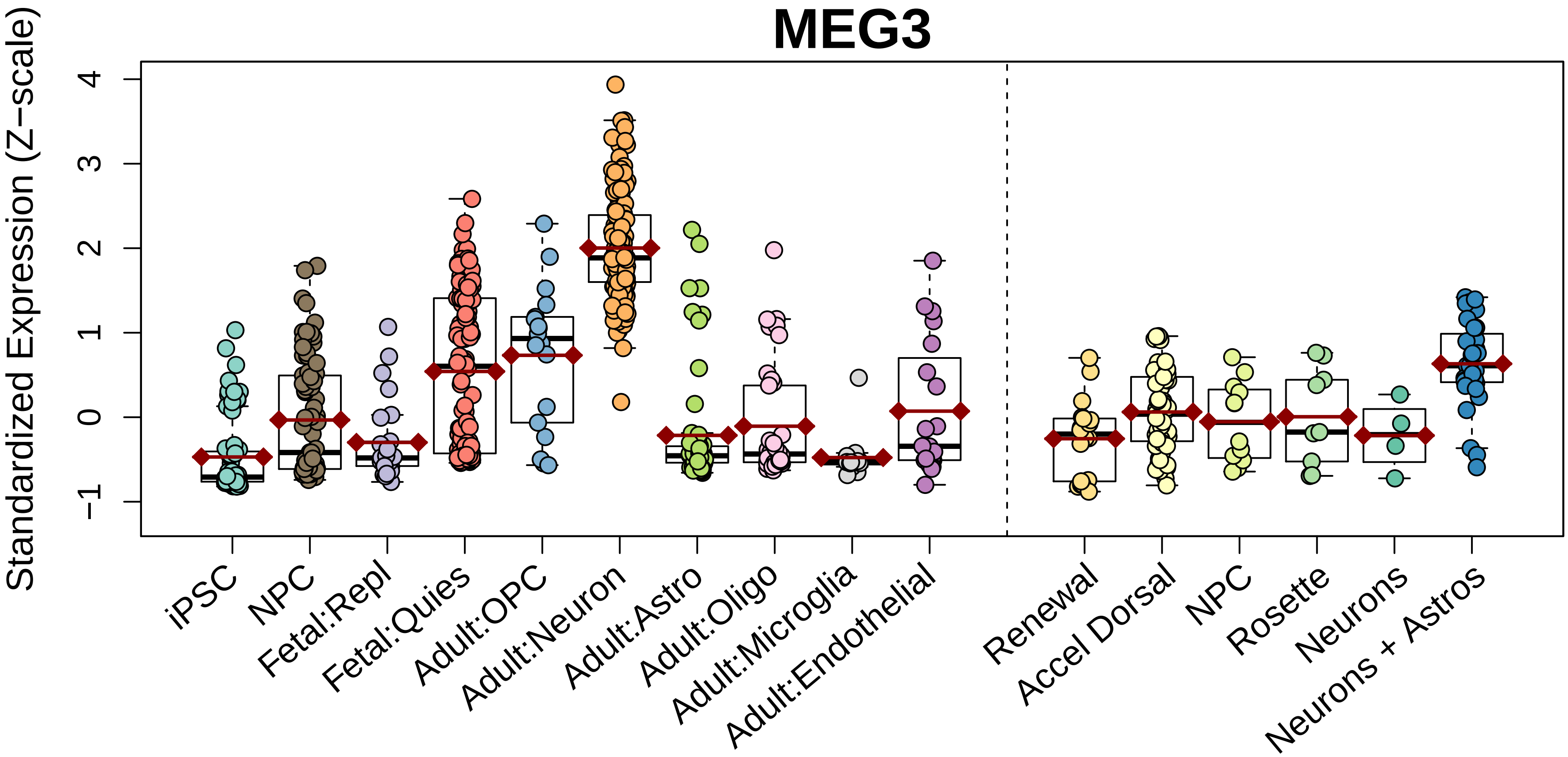
MEG3
Standardized Expression (Z−scale)
4
3
2
1
0
−1
iPSC
NPC
Fetal:Repl
Fetal:Quies
Adult:OPC
Adult:Neuron
Adult:Astro
Adult:Oligo
Adult:Microglia
Adult:Endothelial
Renewal
Accel Dorsal
NPC
Rosette
Neurons
Neurons + Astros

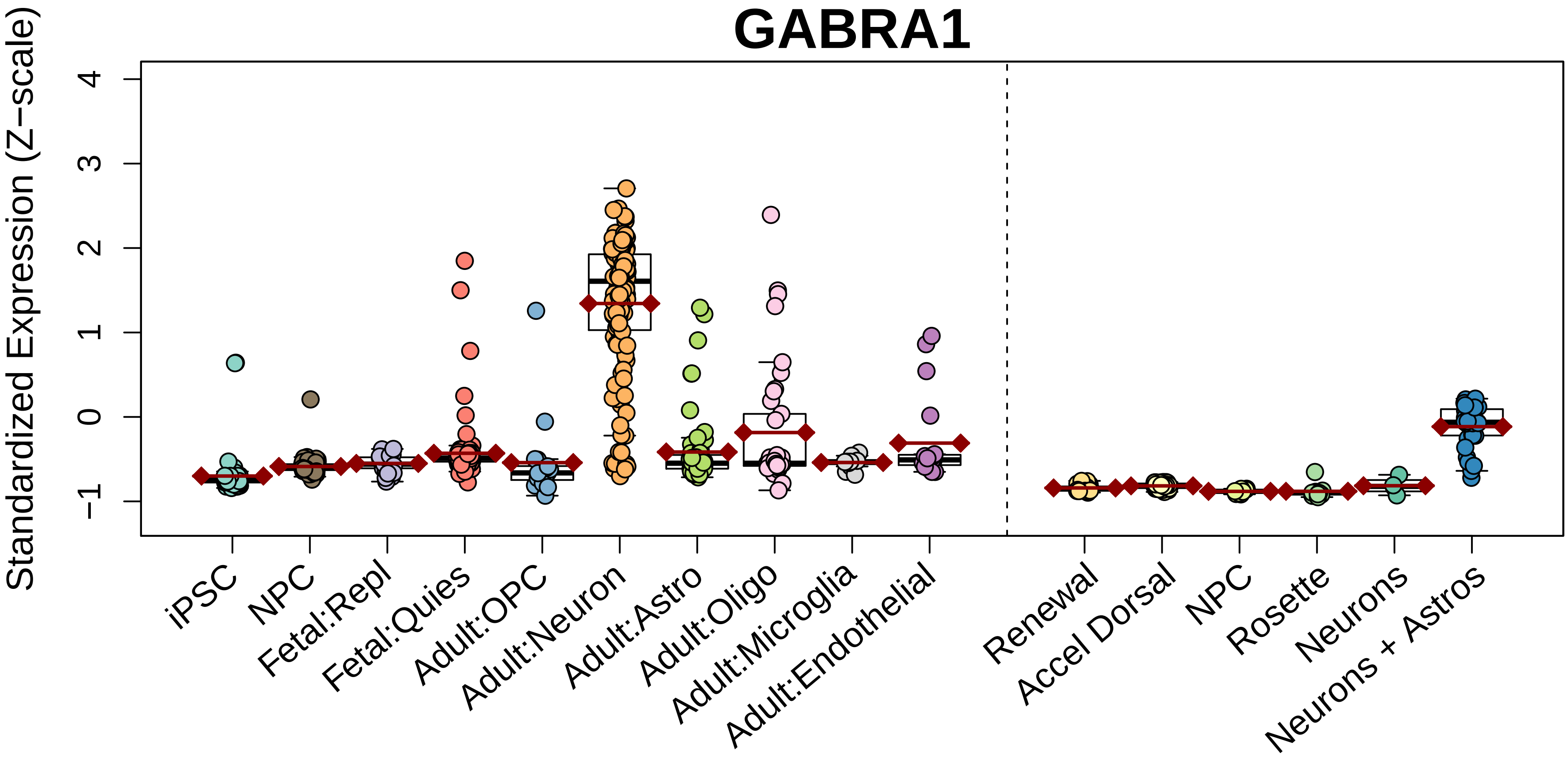

GABRA1
Standardized Expression (Z−scale)
4
3
2
1
0
−1
iPSC
NPC
Fetal:Repl
Fetal:Quies
Adult:OPC
Adult:Neuron
Adult:Astro
Adult:Oligo
Adult:Microglia
Adult:Endothelial
Renewal
Accel Dorsal
NPC
Rosette
Neurons
Neurons + Astros

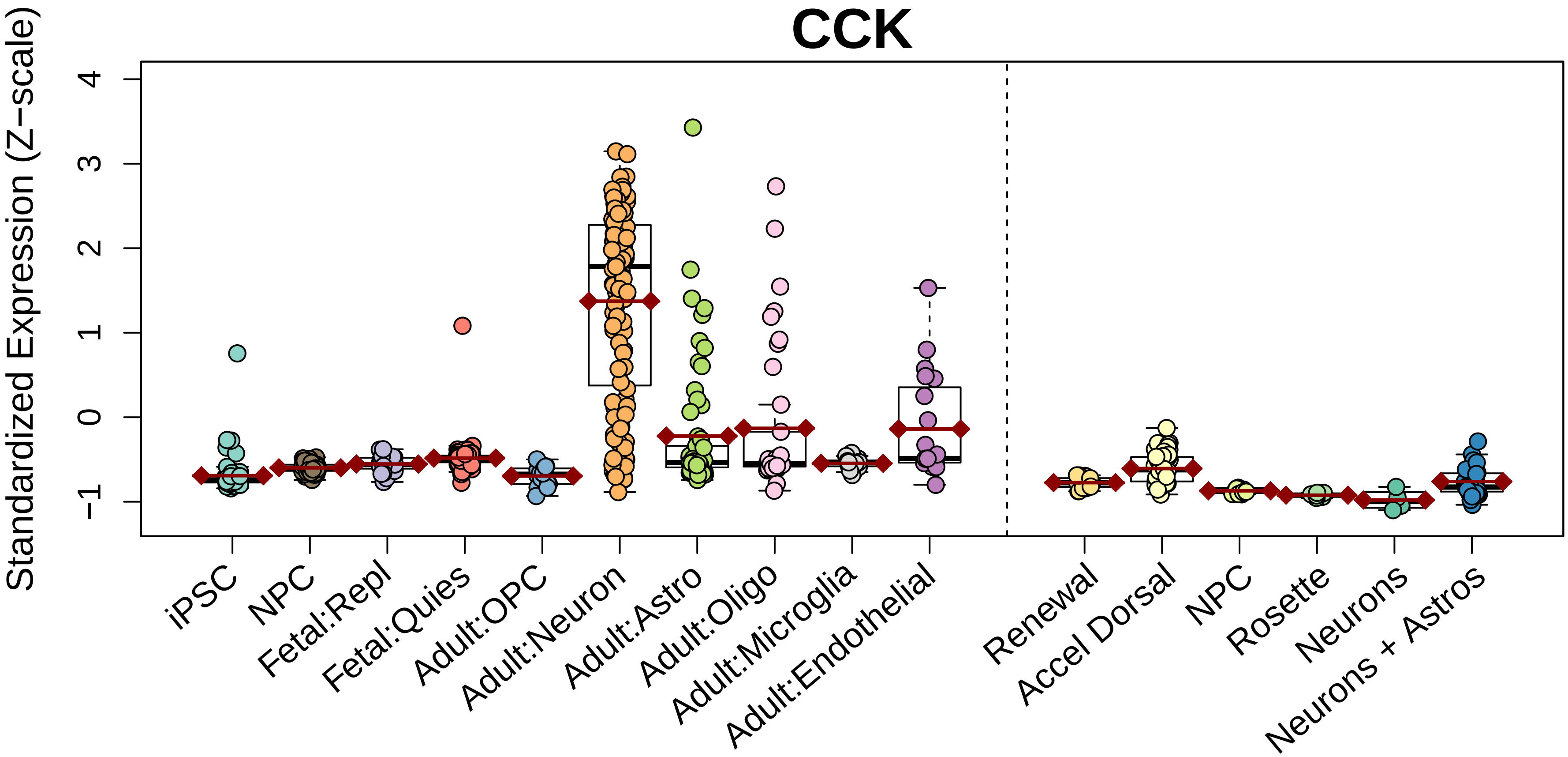

CCK
Standardized Expression (Z−scale)
4
3
2
1
0
−1
iPSC
NPC
Fetal:Repl
Fetal:Quies
Adult:OPC
Adult:Neuron
Adult:Astro
Adult:Oligo
Adult:Microglia
Adult:Endothelial
Renewal
Accel Dorsal
NPC
Rosette
Neurons
Neurons + Astros

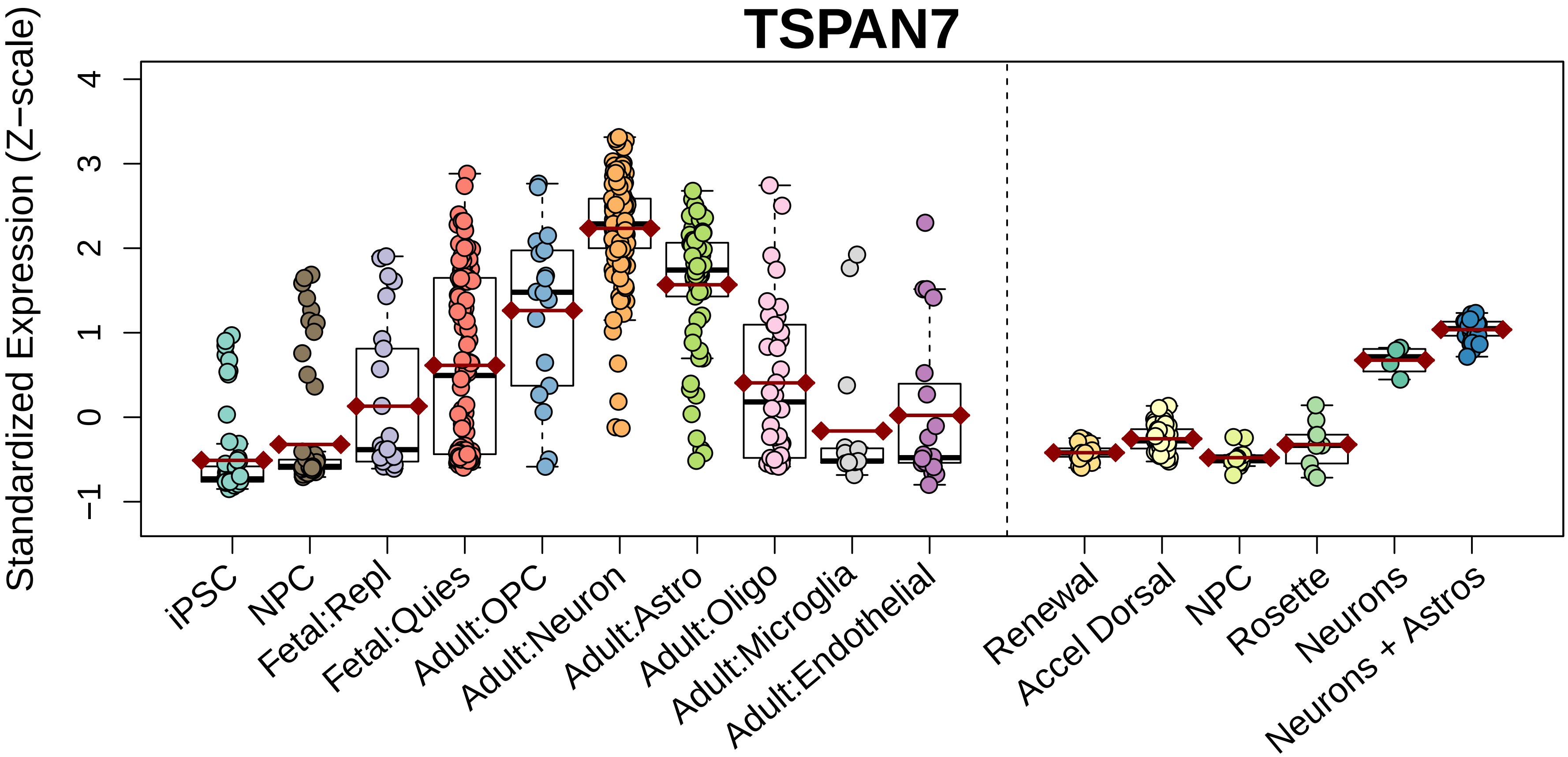
TSPAN7
Standardized Expression (Z−scale)
4
3
2
1
0
−1
iPSC
NPC
Fetal:Repl
Fetal:Quies
Adult:OPC
Adult:Neuron
Adult:Astro
Adult:Oligo
Adult:Microglia
Adult:Endothelial
Renewal
Accel Dorsal
NPC
Rosette
Neurons
Neurons + Astros

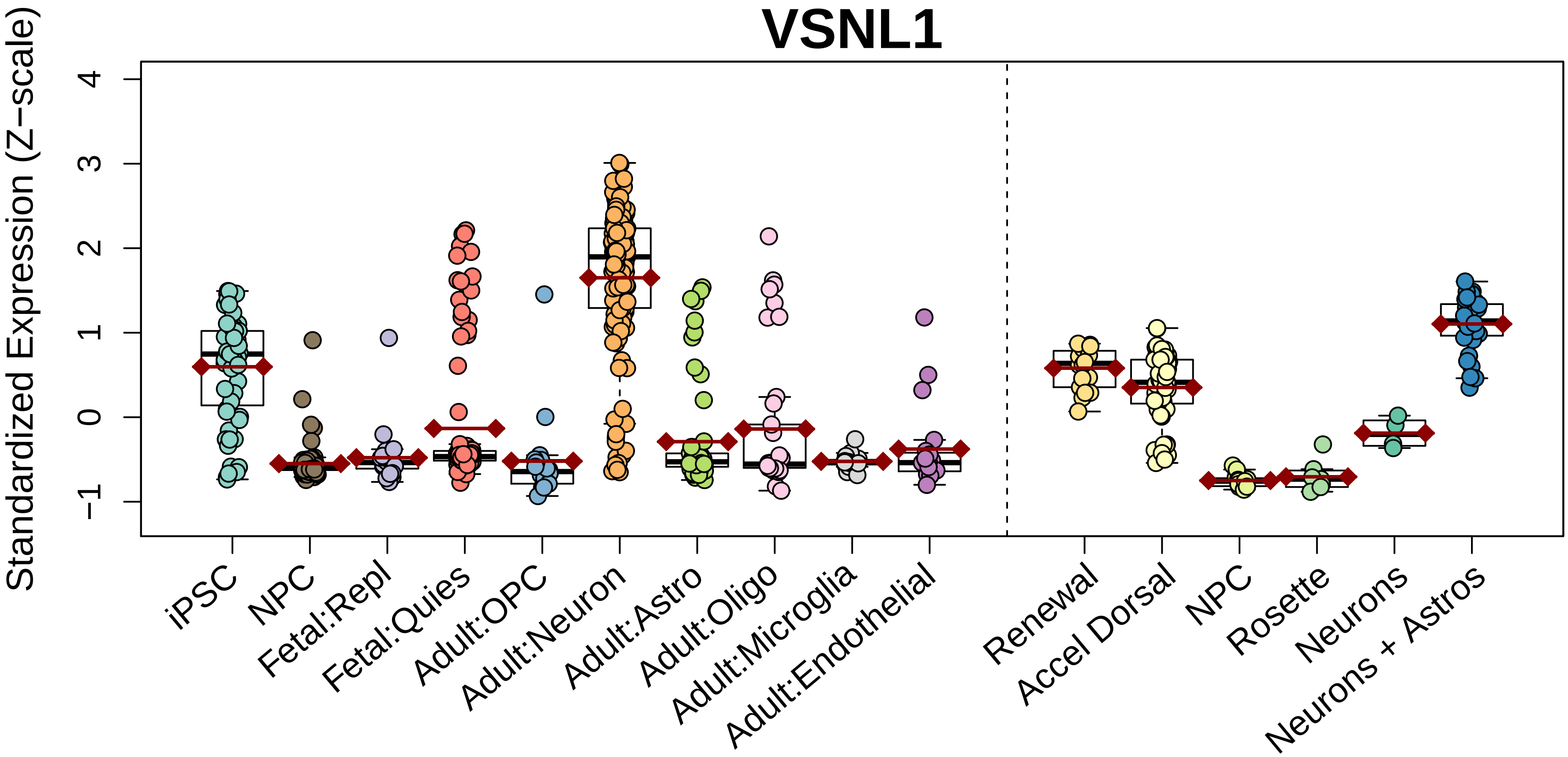
VSNL1
Standardized Expression (Z−scale)
−1
0
1
2
3
4
iPSC
NPC
Fetal:Repl
Fetal:Quies
Adult:OPC
Adult:Neuron
Adult:Astro
Adult:Oligo
Adult:Microglia
Adult:Endothelial
Renewal
Accel Dorsal
NPC
Rosette
Neurons
Neurons + Astros

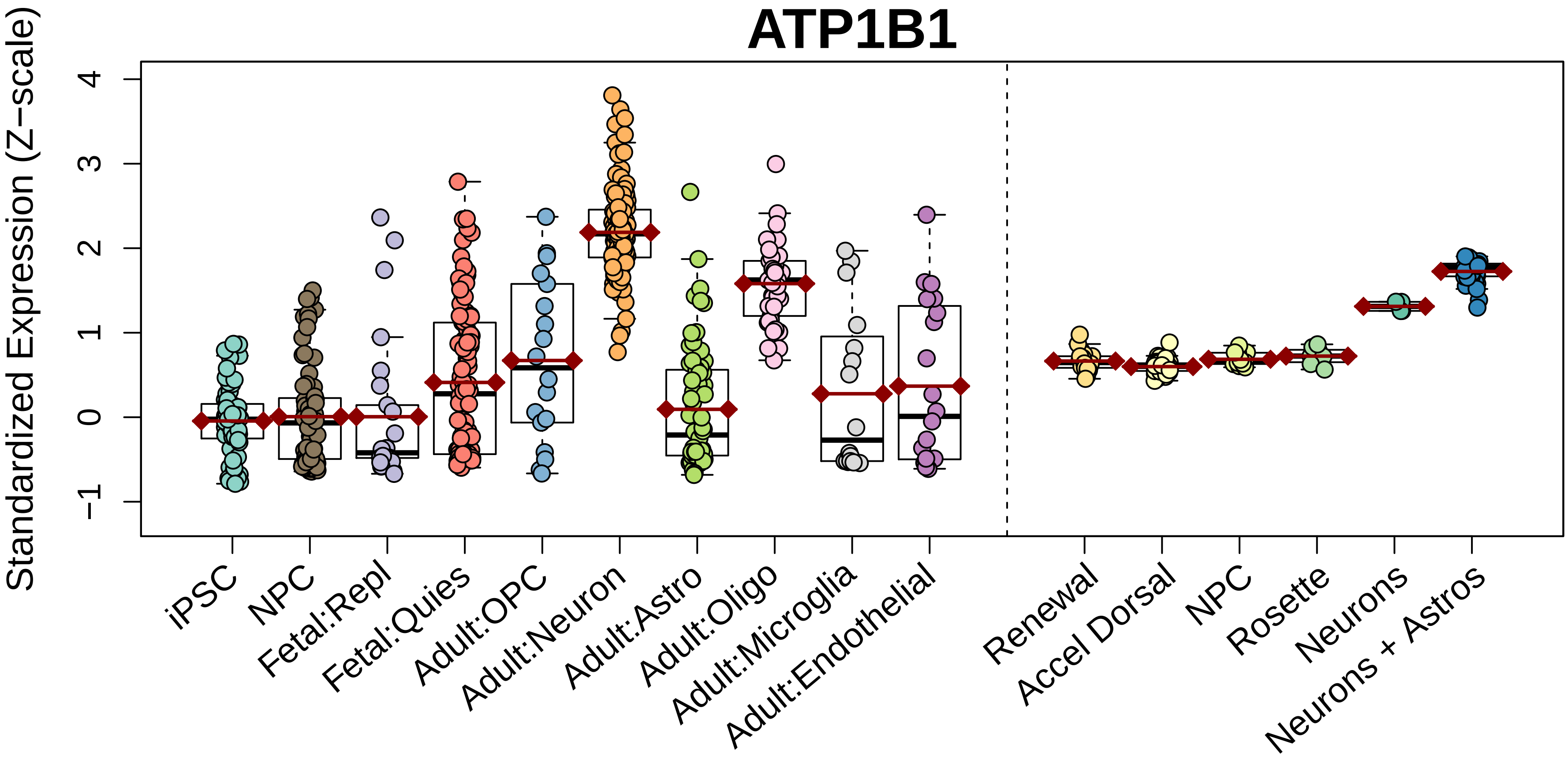
ATP1B1
Standardized Expression (Z−scale)
4
3
2
1
0
−1
iPSC
NPC
Fetal:Repl
Fetal:Quies
Adult:OPC
Adult:Neuron
Adult:Astro
Adult:Oligo
Adult:Microglia
Adult:Endothelial
Renewal
Accel Dorsal
NPC
Rosette
Neurons
Neurons + Astros

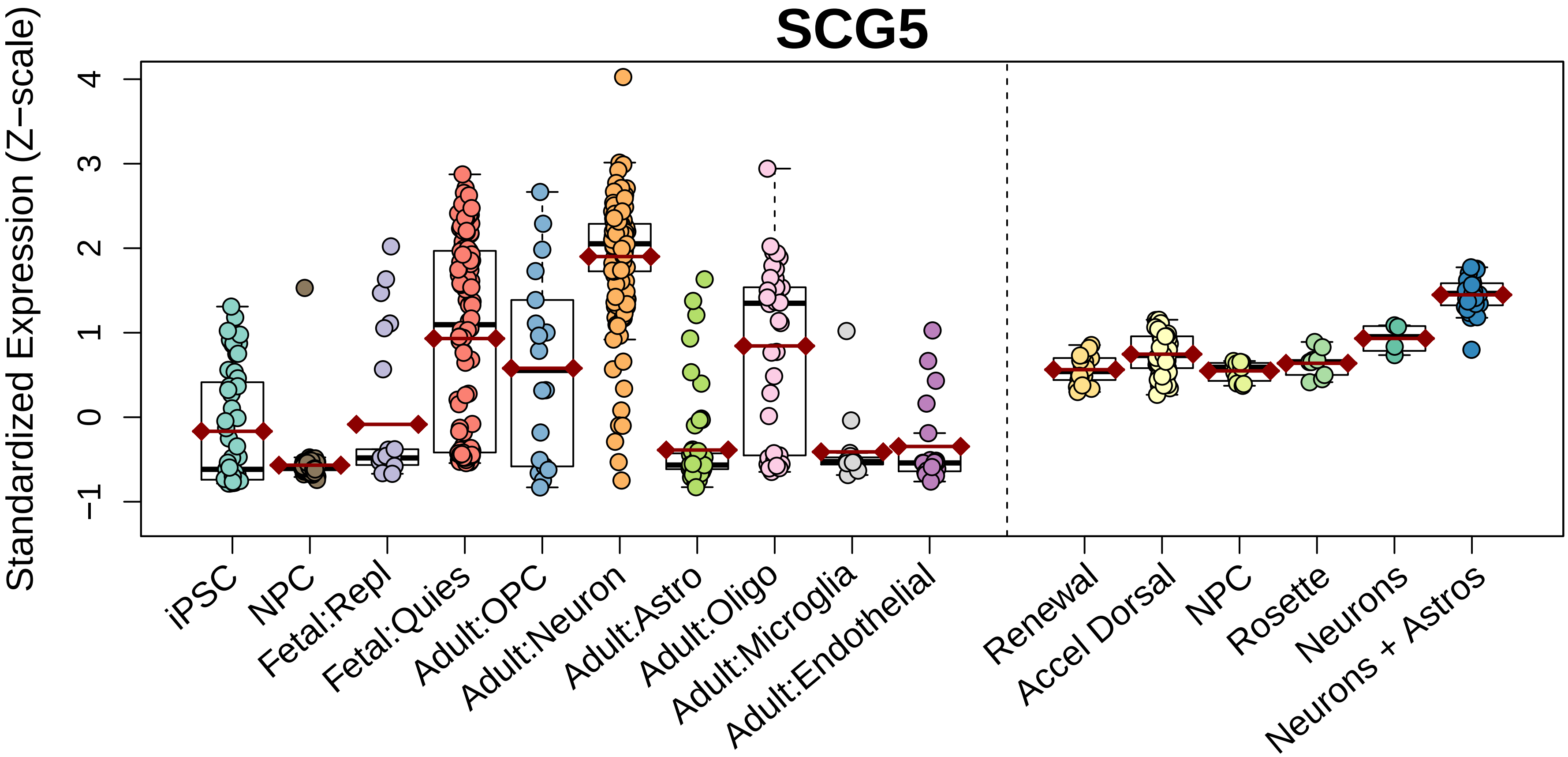
SCG5
Standardized Expression (Z−scale)
4
3
2
1
0
−1
iPSC
NPC
Fetal:Repl
Fetal:Quies
Adult:OPC
Adult:Neuron
Adult:Astro
Adult:Oligo
Adult:Microglia
Adult:Endothelial
Renewal
Accel Dorsal
NPC
Rosette
Neurons
Neurons + Astros

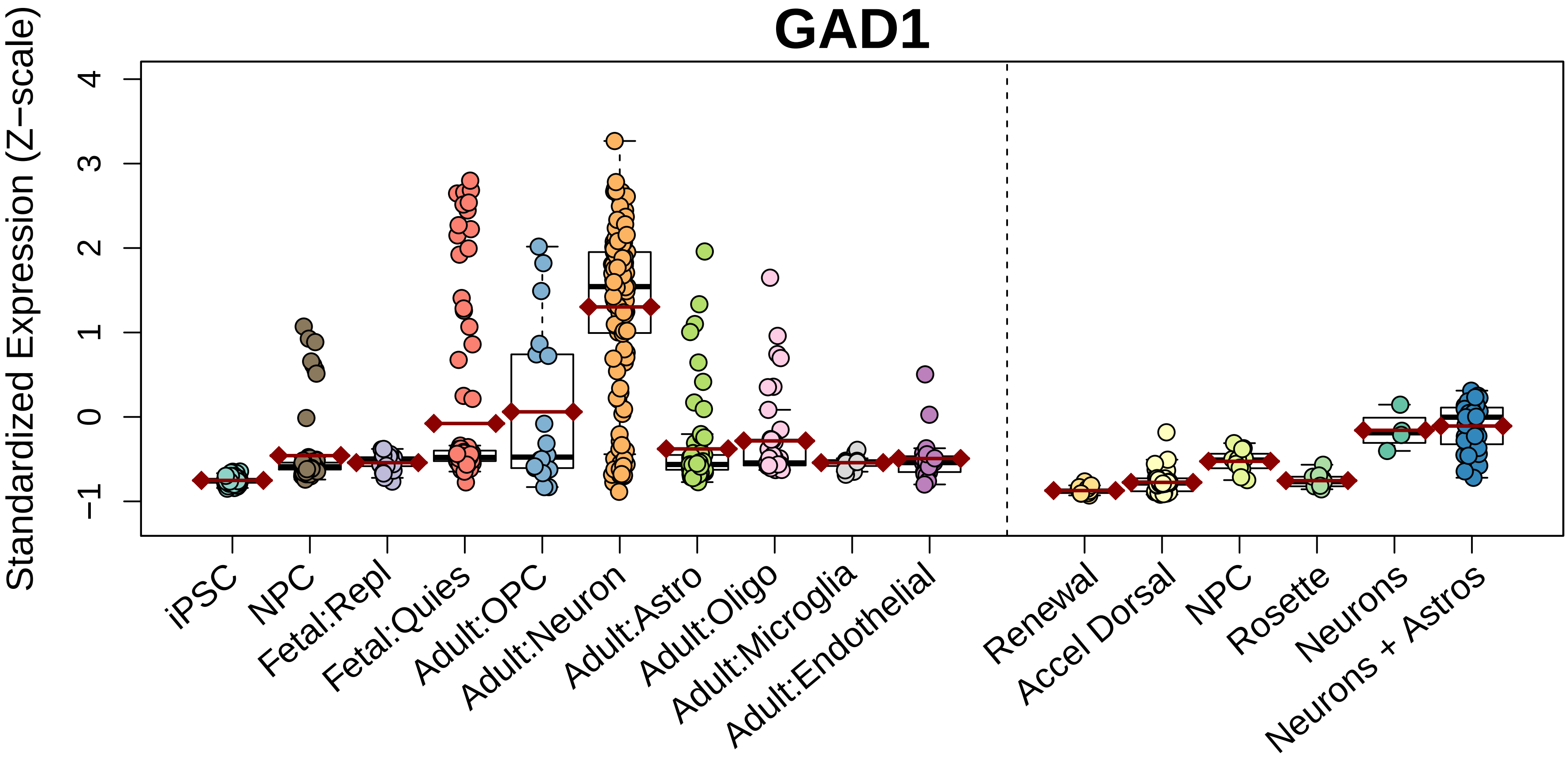

GAD1
Standardized Expression (Z−scale)
4
3
2
1
0
−1
iPSC
NPC
Fetal:Repl
Fetal:Quies
Adult:OPC
Adult:Neuron
Adult:Astro
Adult:Oligo
Adult:Microglia
Adult:Endothelial
Renewal
Accel Dorsal
NPC
Rosette
Neurons
Neurons + Astros

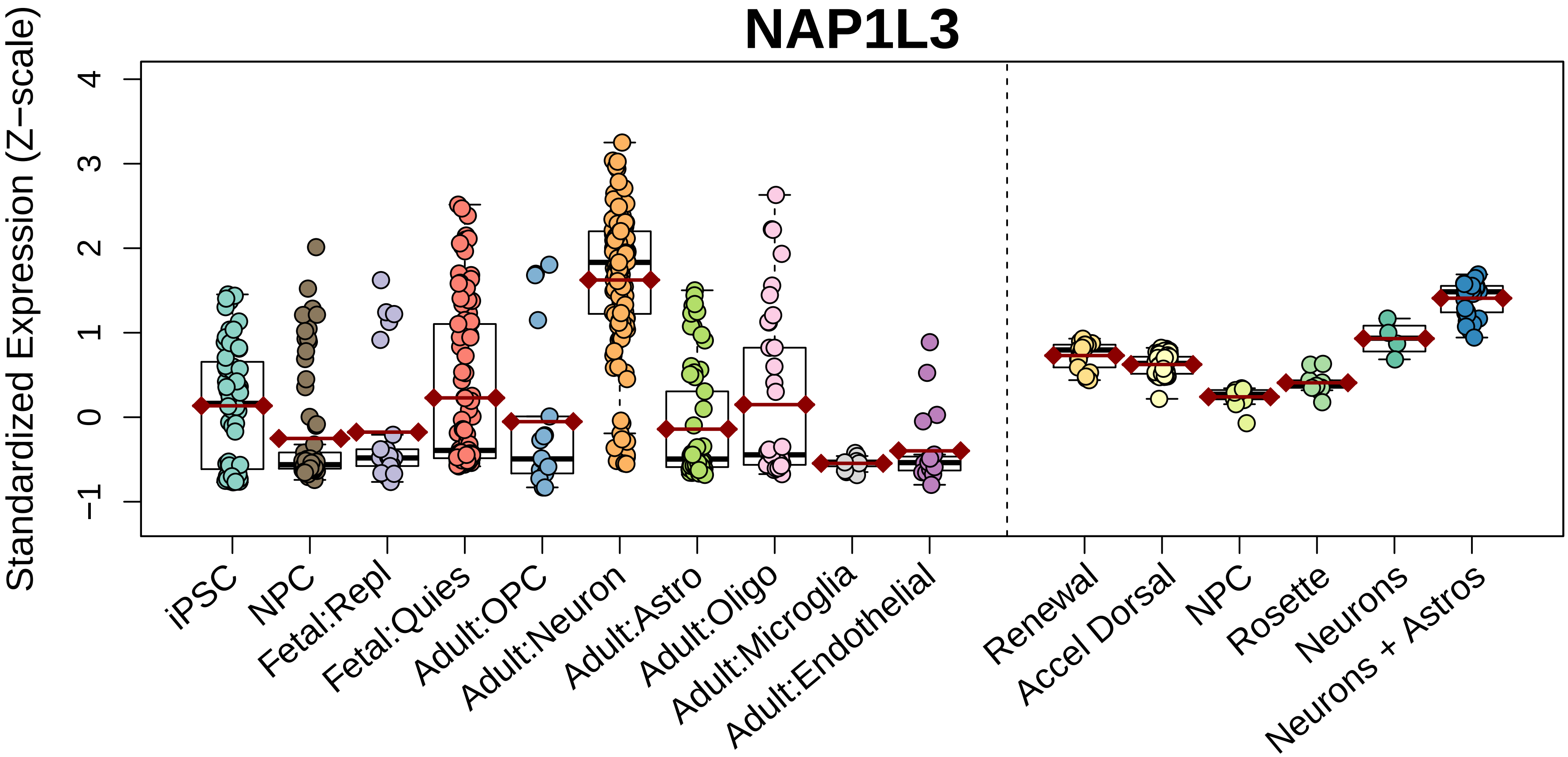
NAP1L3
Standardized Expression (Z−scale)
4
3
2
1
0
−1
iPSC
NPC
Fetal:Repl
Fetal:Quies
Adult:OPC
Adult:Neuron
Adult:Astro
Adult:Oligo
Adult:Microglia
Adult:Endothelial
Renewal
Accel Dorsal
NPC
Rosette
Neurons
Neurons + Astros

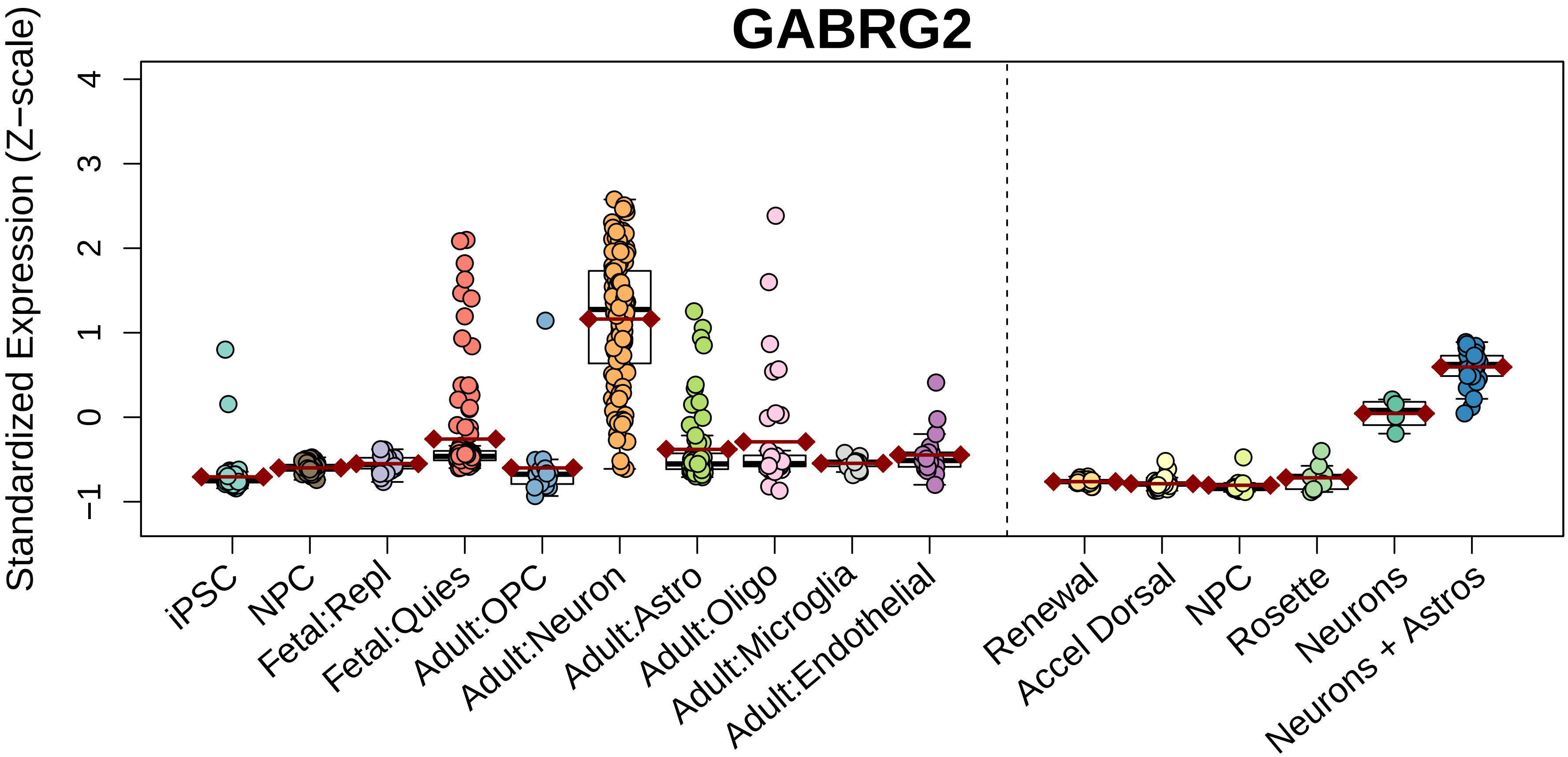

GABRG2
Standardized Expression (Z−scale)
4
3
2
1
0
−1
iPSC
NPC
Fetal:Repl
Fetal:Quies
Adult:OPC
Adult:Neuron
Adult:Astro
Adult:Oligo
Adult:Microglia
Adult:Endothelial
Renewal
Accel Dorsal
NPC
Rosette
Neurons
Neurons + Astros

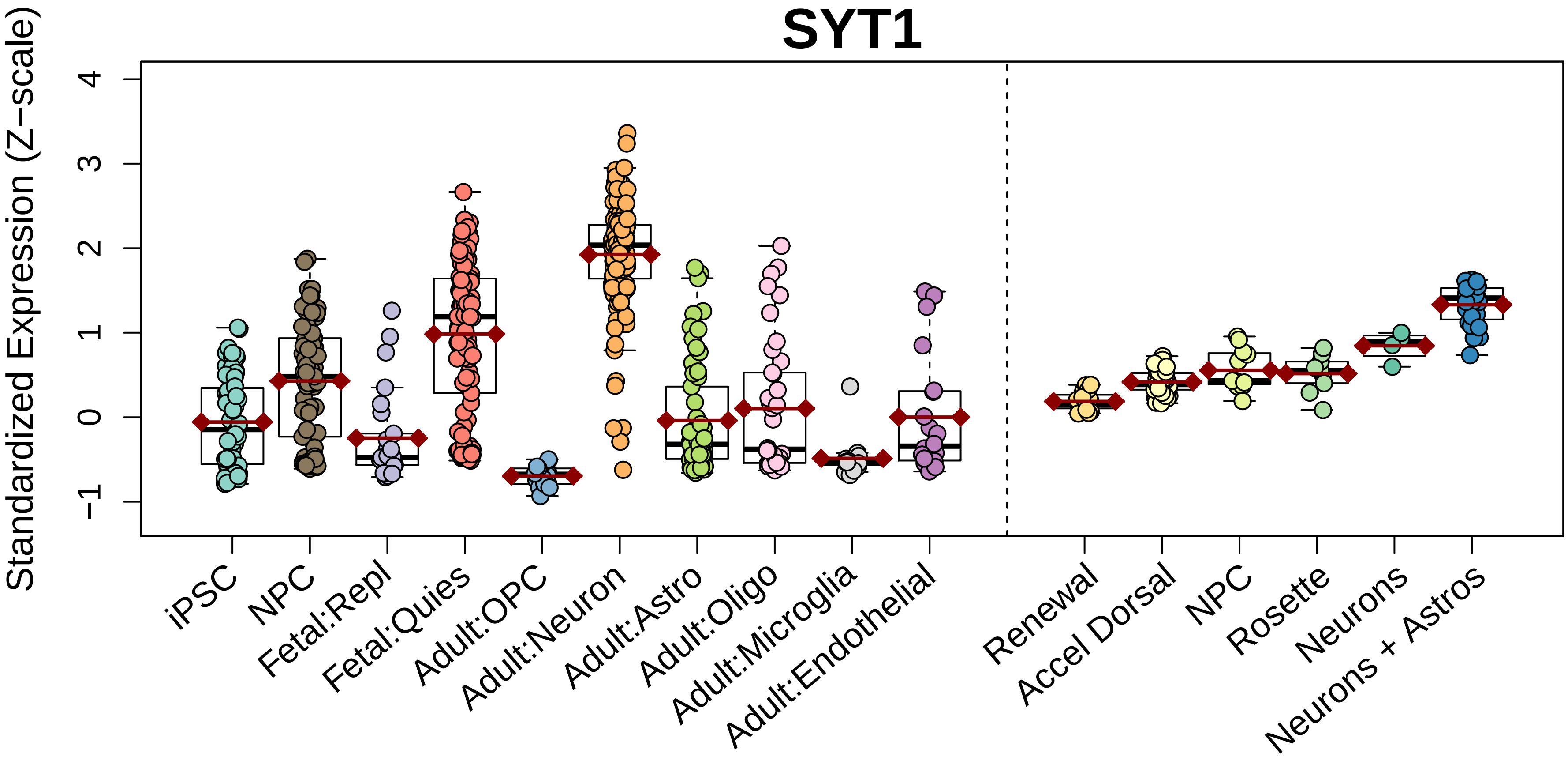

SYT1
Standardized Expression (Z−scale)
4
3
2
1
0
−1
iPSC
NPC
Fetal:Repl
Fetal:Quies
Adult:OPC
Adult:Neuron
Adult:Astro
Adult:Oligo
Adult:Microglia
Adult:Endothelial
Renewal
Accel Dorsal
NPC
Rosette
Neurons
Neurons + Astros

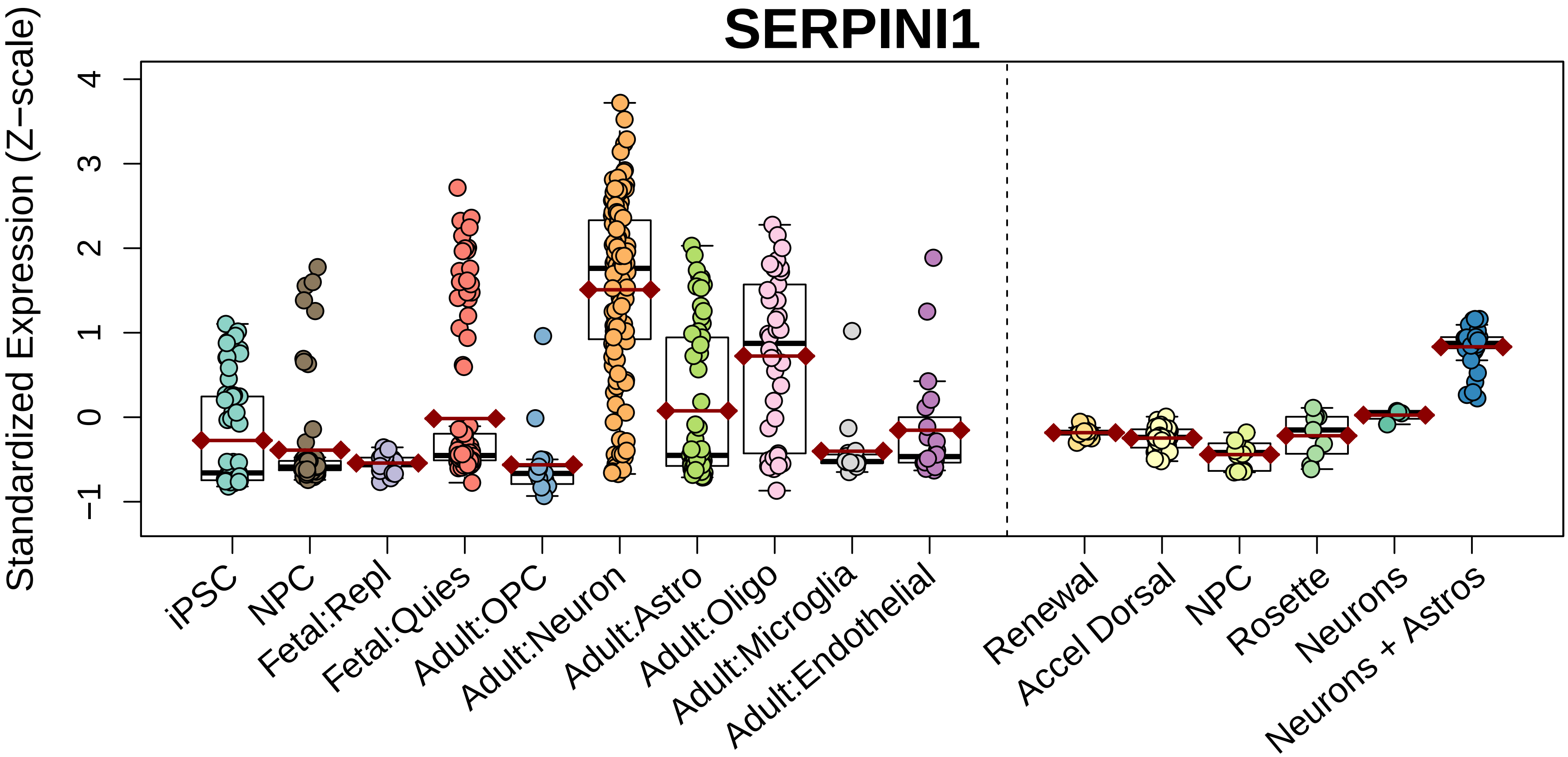
SERPINI1
Standardized Expression (Z−scale)
4
3
2
1
0
−1
iPSC
NPC
Fetal:Repl
Fetal:Quies
Adult:OPC
Adult:Neuron
Adult:Astro
Adult:Oligo
Adult:Microglia
Adult:Endothelial
Renewal
Accel Dorsal
NPC
Rosette
Neurons
Neurons + Astros

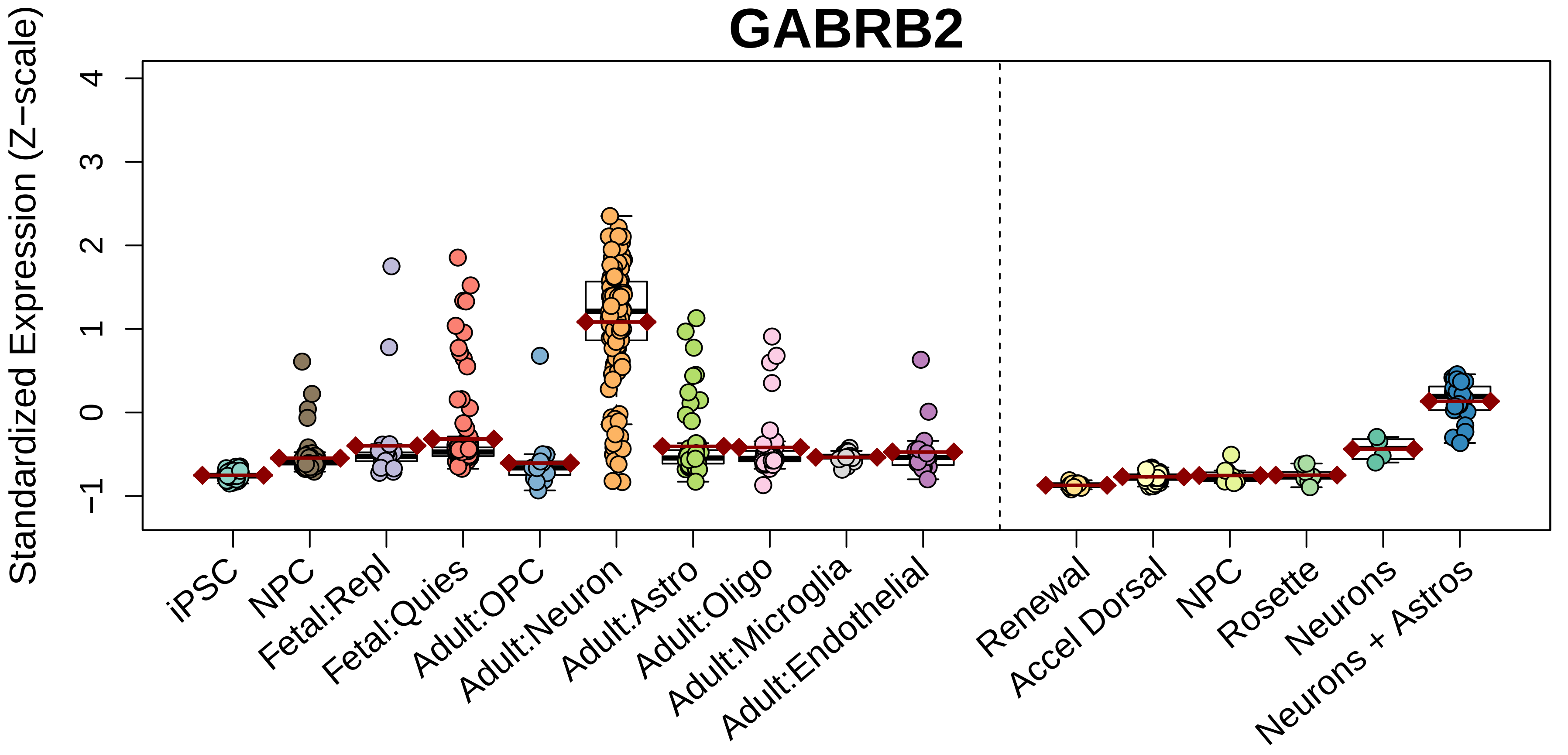
GABRB2
Standardized Expression (Z−scale)
4
3
2
1
0
−1
iPSC
NPC
Fetal:Repl
Fetal:Quies
Adult:OPC
Adult:Neuron
Adult:Astro
Adult:Oligo
Adult:Microglia
Adult:Endothelial
Renewal
Accel Dorsal
NPC
Rosette
Neurons
Neurons + Astros

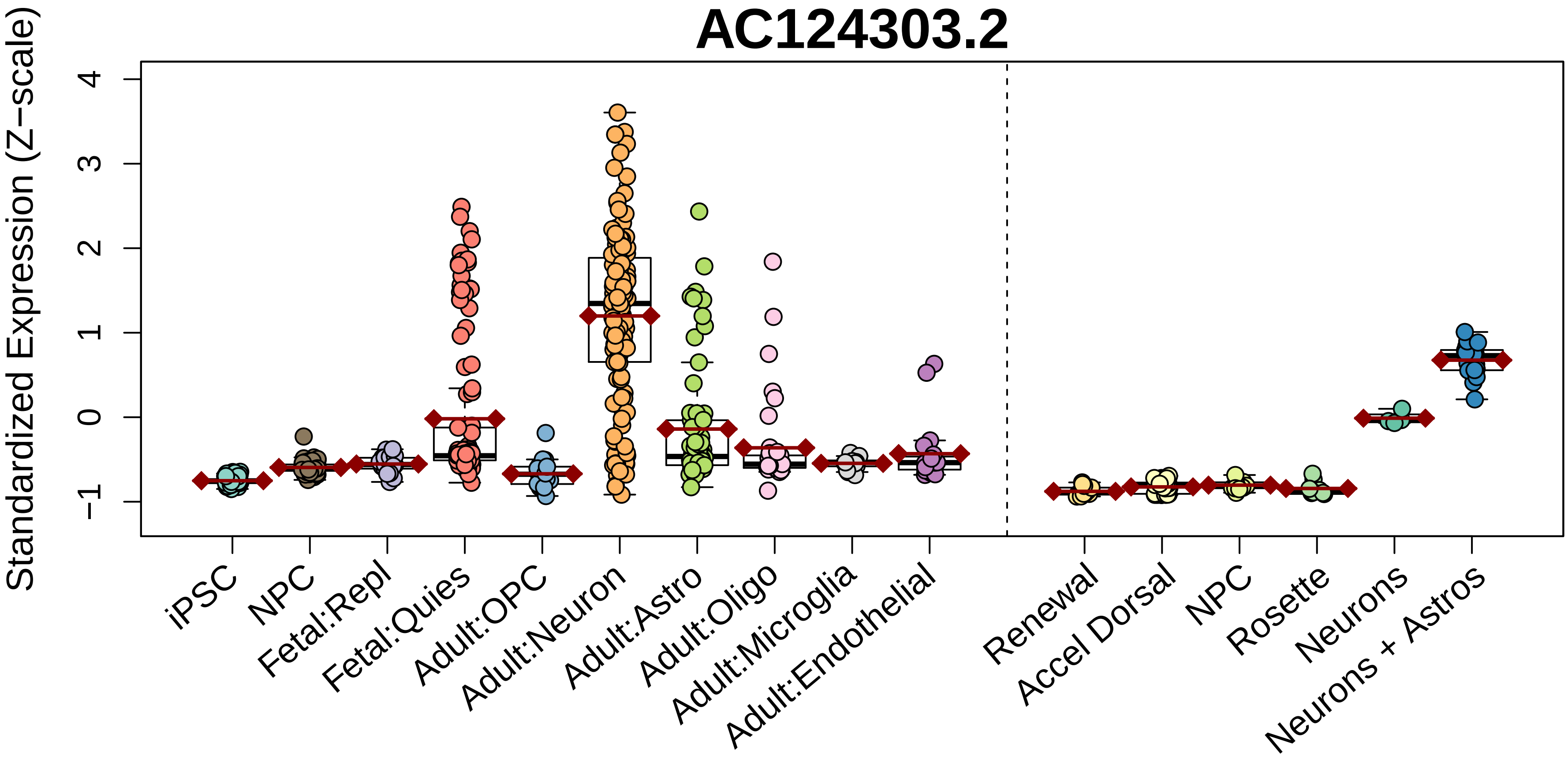
AC124303.2
Standardized Expression (Z−scale)
4
3
2
1
0
−1
iPSC
NPC
Fetal:Repl
Fetal:Quies
Adult:OPC
Adult:Neuron
Adult:Astro
Adult:Oligo
Adult:Microglia
Adult:Endothelial
Renewal
Accel Dorsal
NPC
Rosette
Neurons
Neurons + Astros

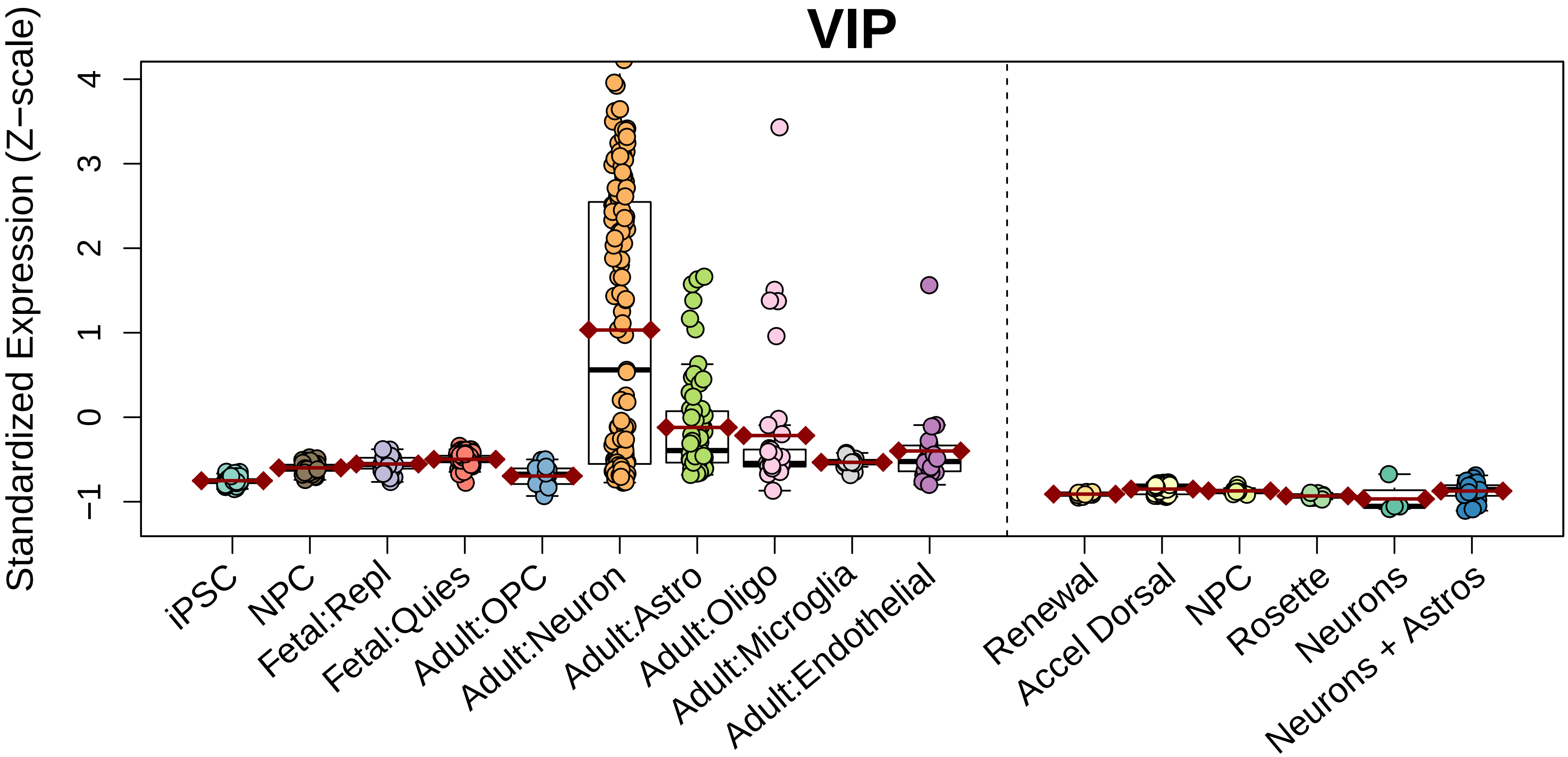

VIP
Standardized Expression (Z−scale)
4
3
2
1
0
−1
iPSC
NPC
Fetal:Repl
Fetal:Quies
Adult:OPC
Adult:Neuron
Adult:Astro
Adult:Oligo
Adult:Microglia
Adult:Endothelial
Renewal
Accel Dorsal
NPC
Rosette
Neurons
Neurons + Astros

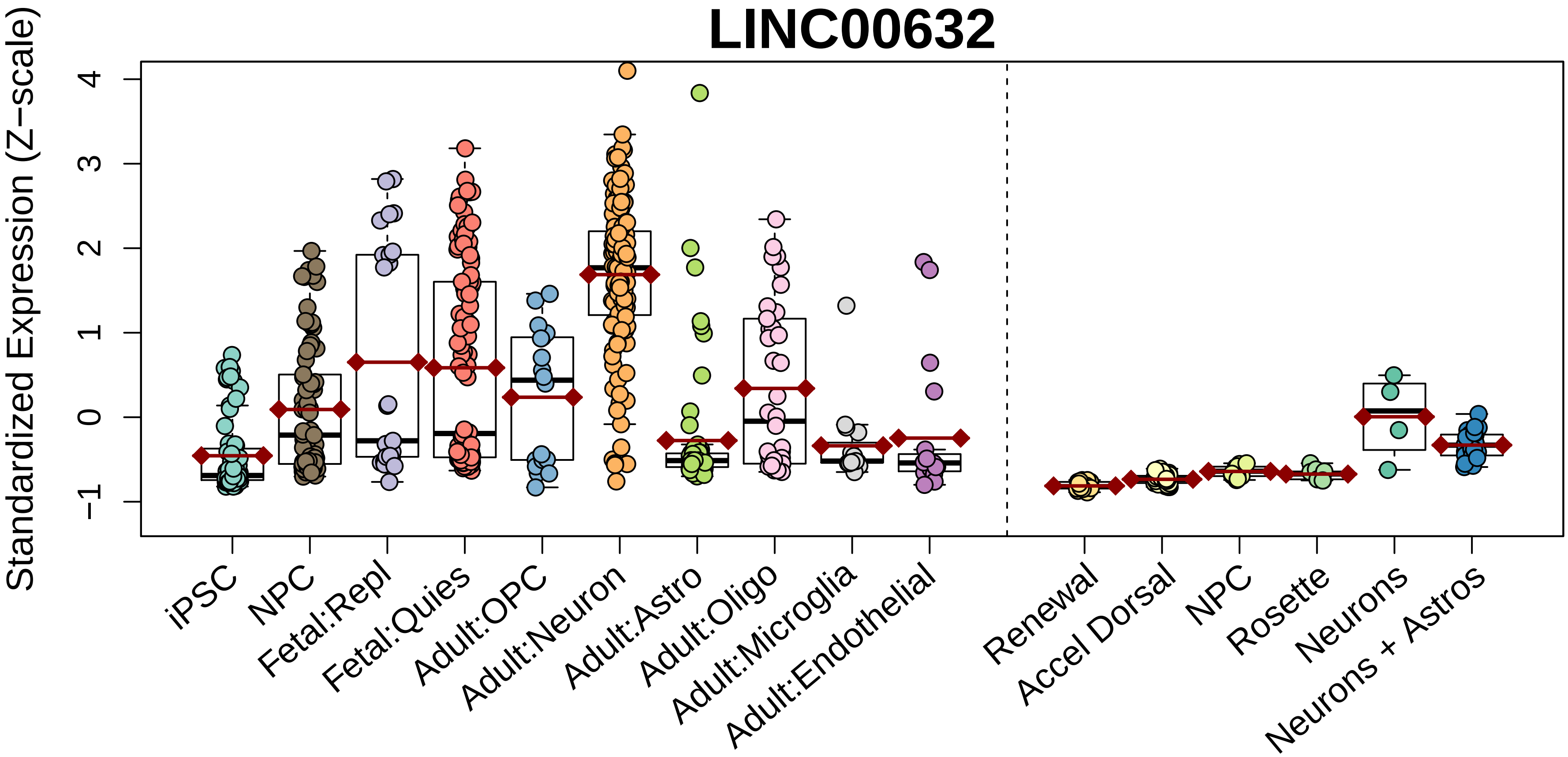
LINC00632
Standardized Expression (Z−scale)
4
3
2
1
0
−1
iPSC
NPC
Fetal:Repl
Fetal:Quies
Adult:OPC
Adult:Neuron
Adult:Astro
Adult:Oligo
Adult:Microglia
Adult:Endothelial
Renewal
Accel Dorsal
NPC
Rosette
Neurons
Neurons + Astros

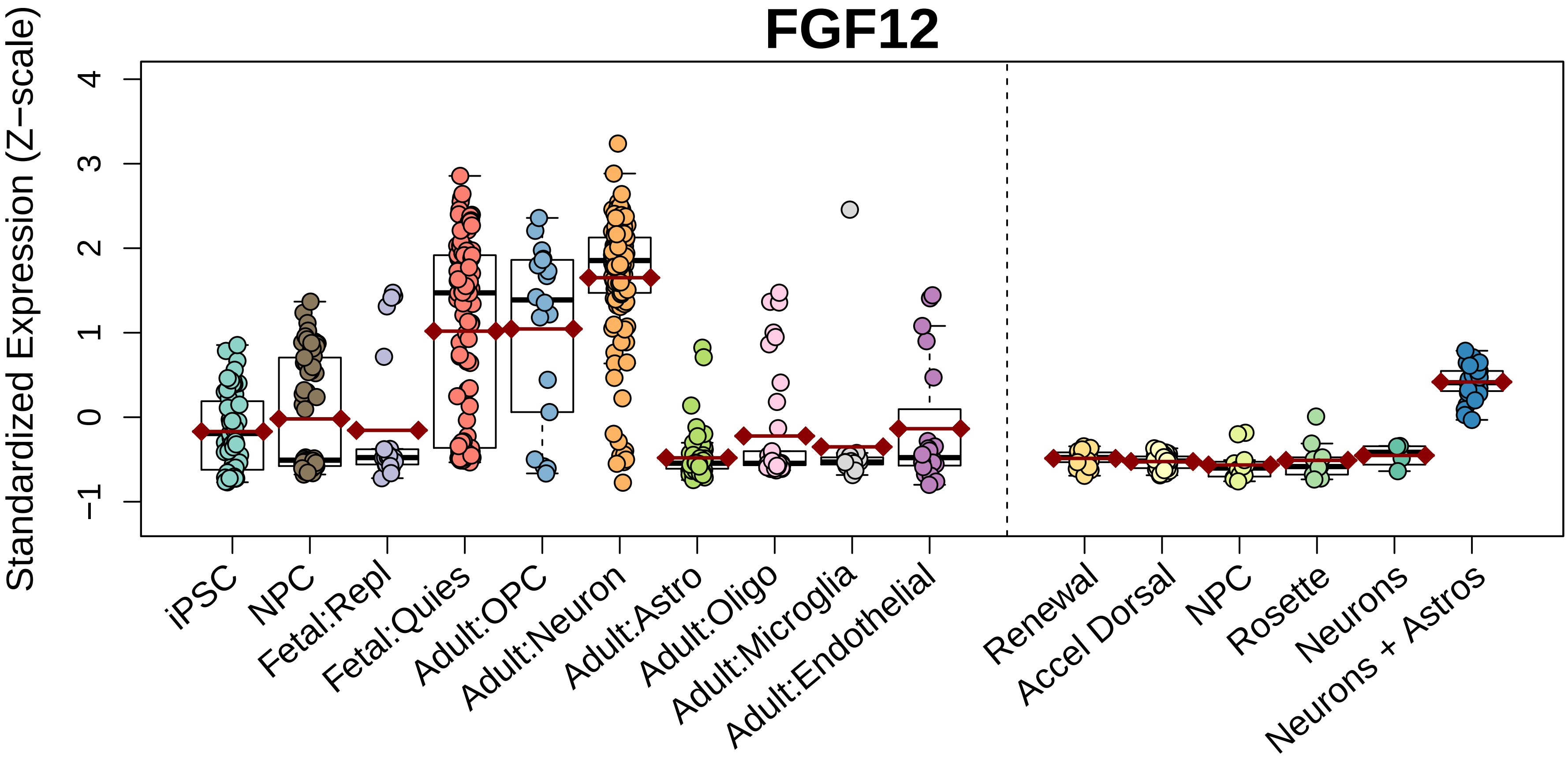

FGF12
Standardized Expression (Z−scale)
4
3
2
1
0
−1
iPSC
NPC
Fetal:Repl
Fetal:Quies
Adult:OPC
Adult:Neuron
Adult:Astro
Adult:Oligo
Adult:Microglia
Adult:Endothelial
Renewal
Accel Dorsal
NPC
Rosette
Neurons
Neurons + Astros

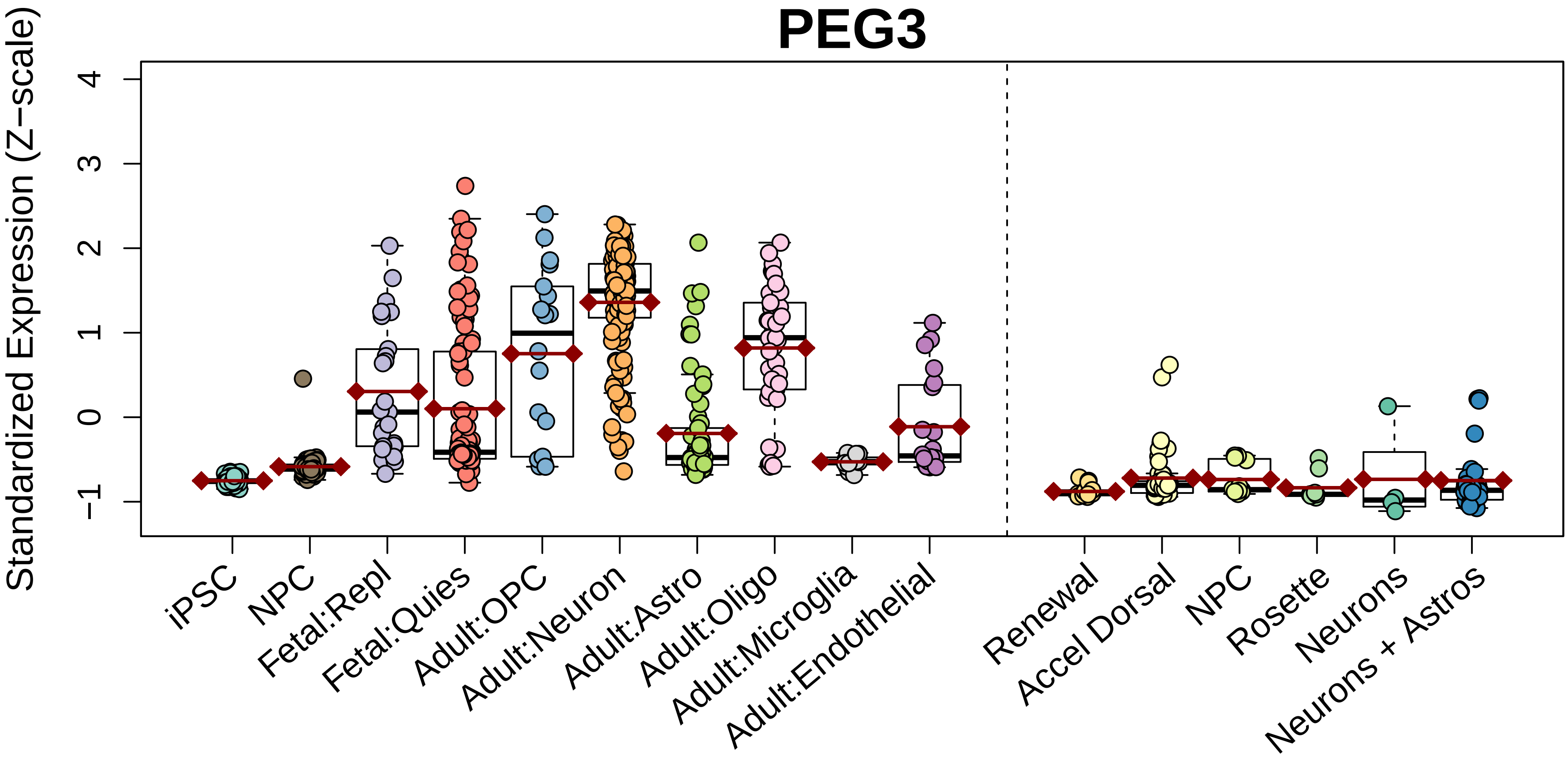

PEG3
Standardized Expression (Z−scale)
4
3
2
1
0
−1
iPSC
NPC
Fetal:Repl
Fetal:Quies
Adult:OPC
Adult:Neuron
Adult:Astro
Adult:Oligo
Adult:Microglia
Adult:Endothelial
Renewal
Accel Dorsal
NPC
Rosette
Neurons
Neurons + Astros

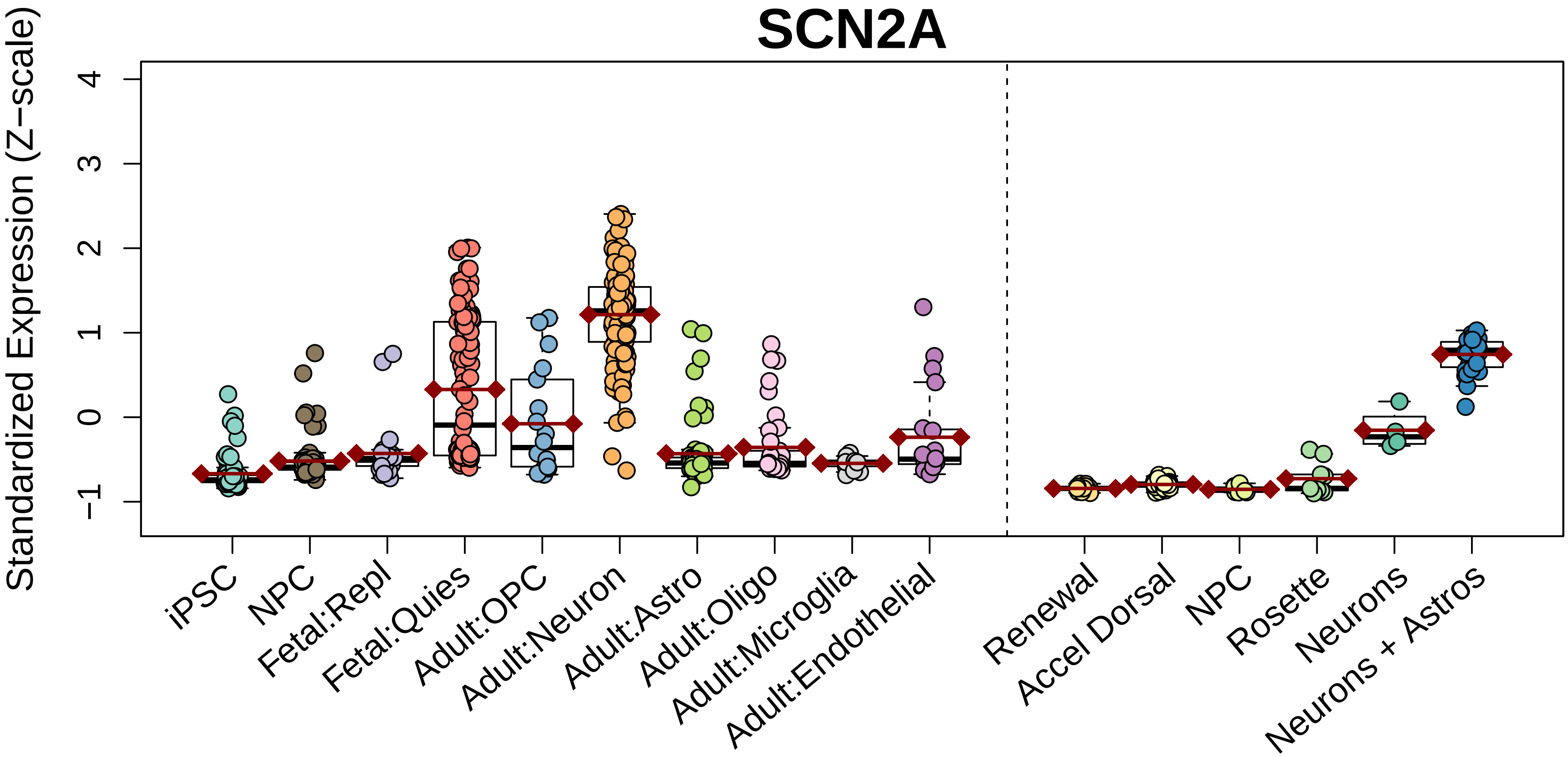

SCN2A
Standardized Expression (Z−scale)
4
3
2
1
0
−1
iPSC
NPC
Fetal:Repl
Fetal:Quies
Adult:OPC
Adult:Neuron
Adult:Astro
Adult:Oligo
Adult:Microglia
Adult:Endothelial
Renewal
Accel Dorsal
NPC
Rosette
Neurons
Neurons + Astros

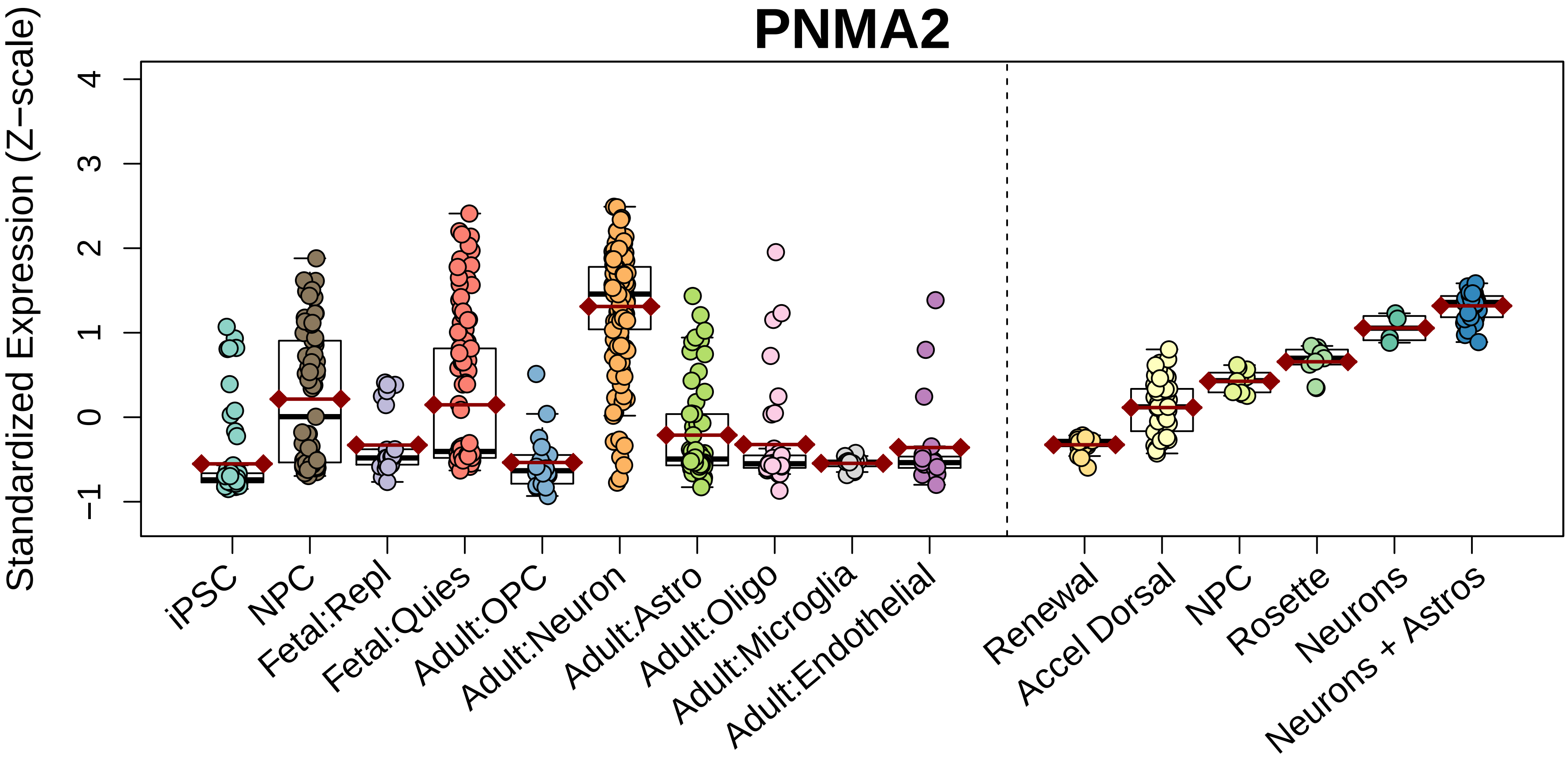
PNMA2
Standardized Expression (Z−scale)
4
3
2
1
0
−1
iPSC
NPC
Fetal:Repl
Fetal:Quies
Adult:OPC
Adult:Neuron
Adult:Astro
Adult:Oligo
Adult:Microglia
Adult:Endothelial
Renewal
Accel Dorsal
NPC
Rosette
Neurons
Neurons + Astros

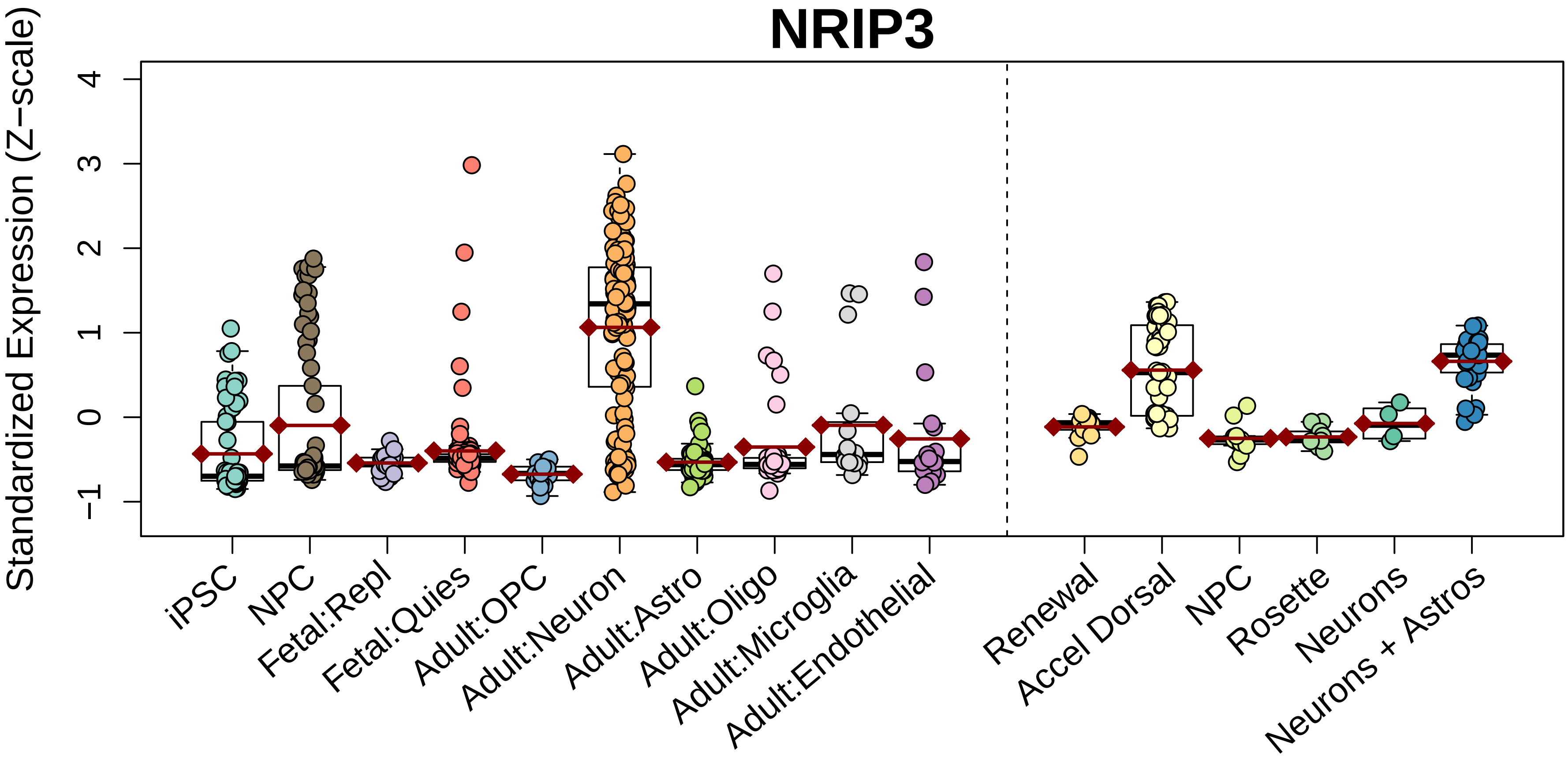
NRIP3
Standardized Expression (Z−scale)
−1
0
1
2
3
4
iPSC
NPC
Fetal:Repl
Fetal:Quies
Adult:OPC
Adult:Neuron
Adult:Astro
Adult:Oligo
Adult:Microglia
Adult:Endothelial
Renewal
Accel Dorsal
NPC
Rosette
Neurons
Neurons + Astros

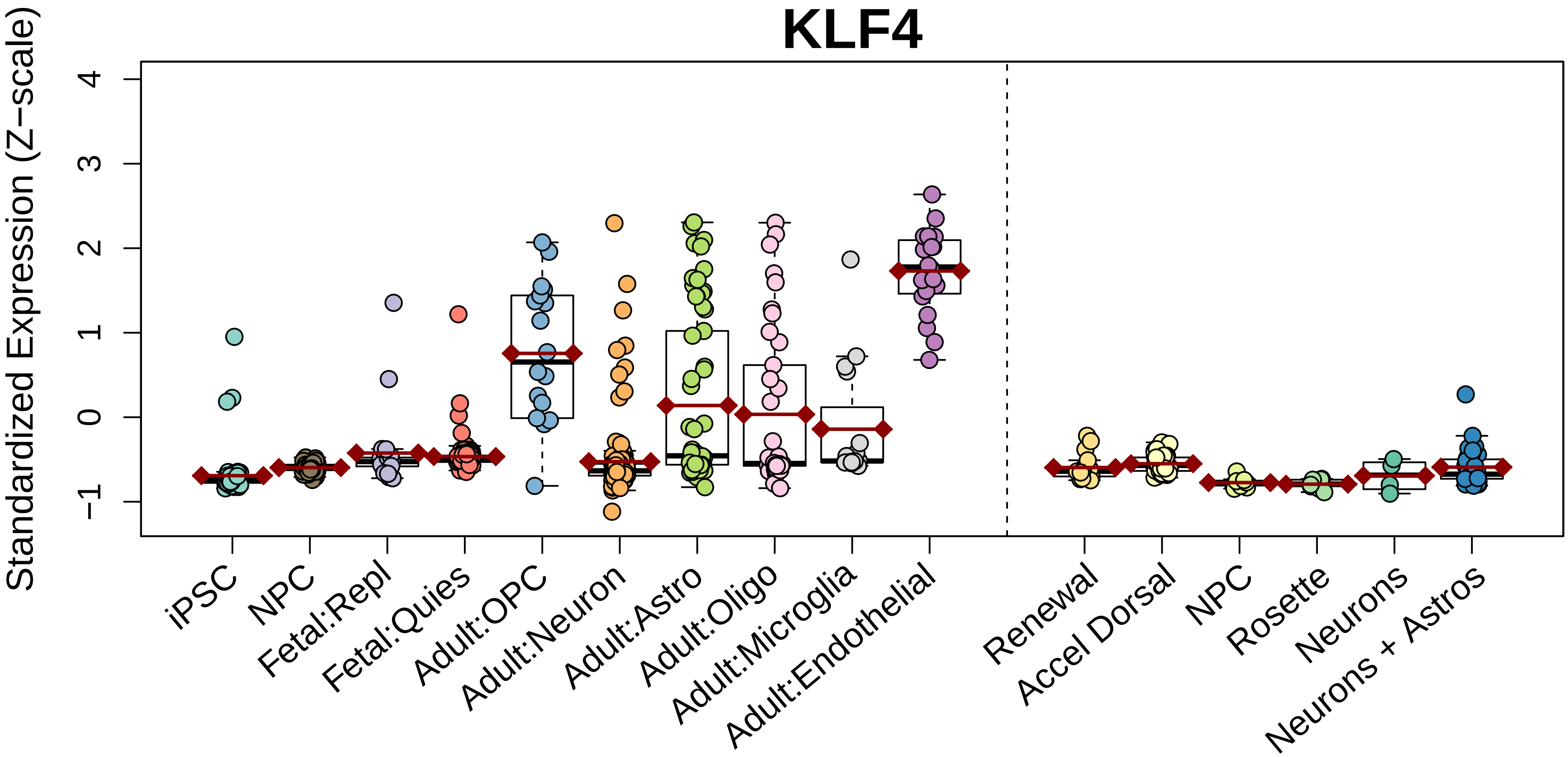

KLF4
Standardized Expression (Z−scale)
−1
0
1
2
3
4
iPSC
NPC
Fetal:Repl
Fetal:Quies
Adult:OPC
Adult:Neuron
Adult:Astro
Adult:Oligo
Adult:Microglia
Adult:Endothelial
Renewal
Accel Dorsal
NPC
Rosette
Neurons
Neurons + Astros

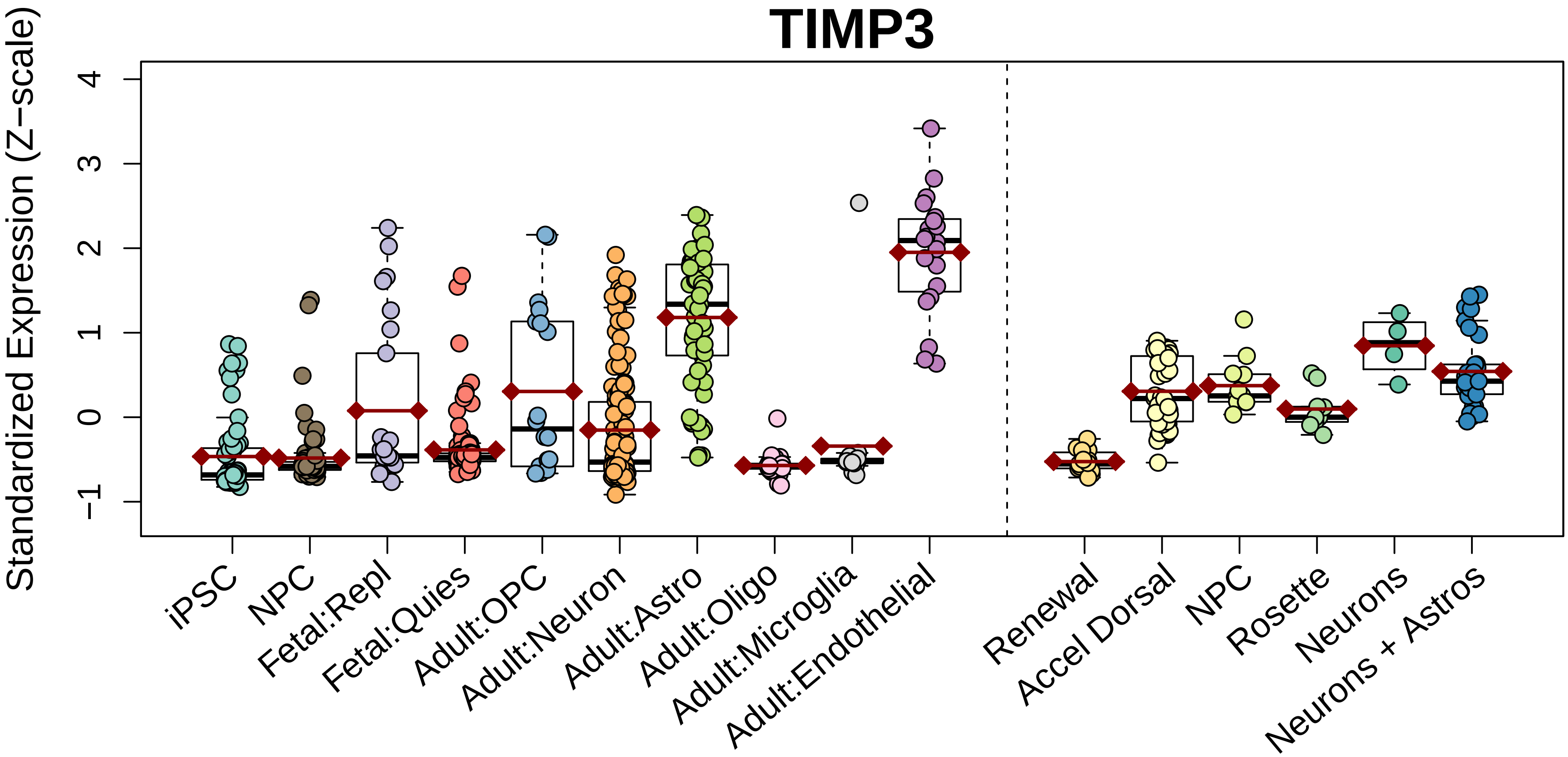

TIMP3
Standardized Expression (Z−scale)
4
3
2
1
0
−1
iPSC
NPC
Fetal:Repl
Fetal:Quies
Adult:OPC
Adult:Neuron
Adult:Astro
Adult:Oligo
Adult:Microglia
Adult:Endothelial
Renewal
Accel Dorsal
NPC
Rosette
Neurons
Neurons + Astros

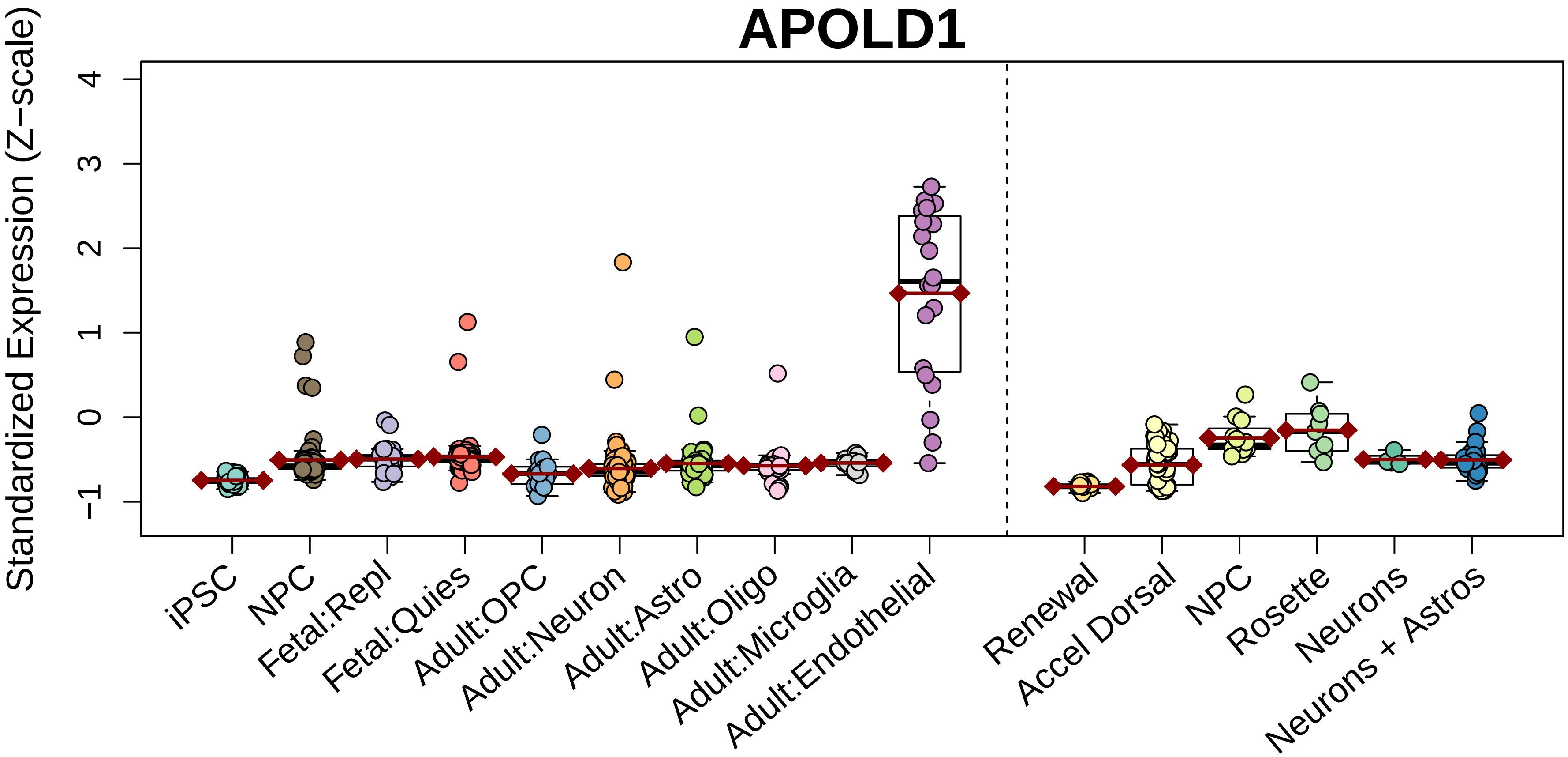

APOLD1
Standardized Expression (Z−scale)
4
3
2
1
0
−1
iPSC
NPC
Fetal:Repl
Fetal:Quies
Adult:OPC
Adult:Neuron
Adult:Astro
Adult:Oligo
Adult:Microglia
Adult:Endothelial
Renewal
Accel Dorsal
NPC
Rosette
Neurons
Neurons + Astros

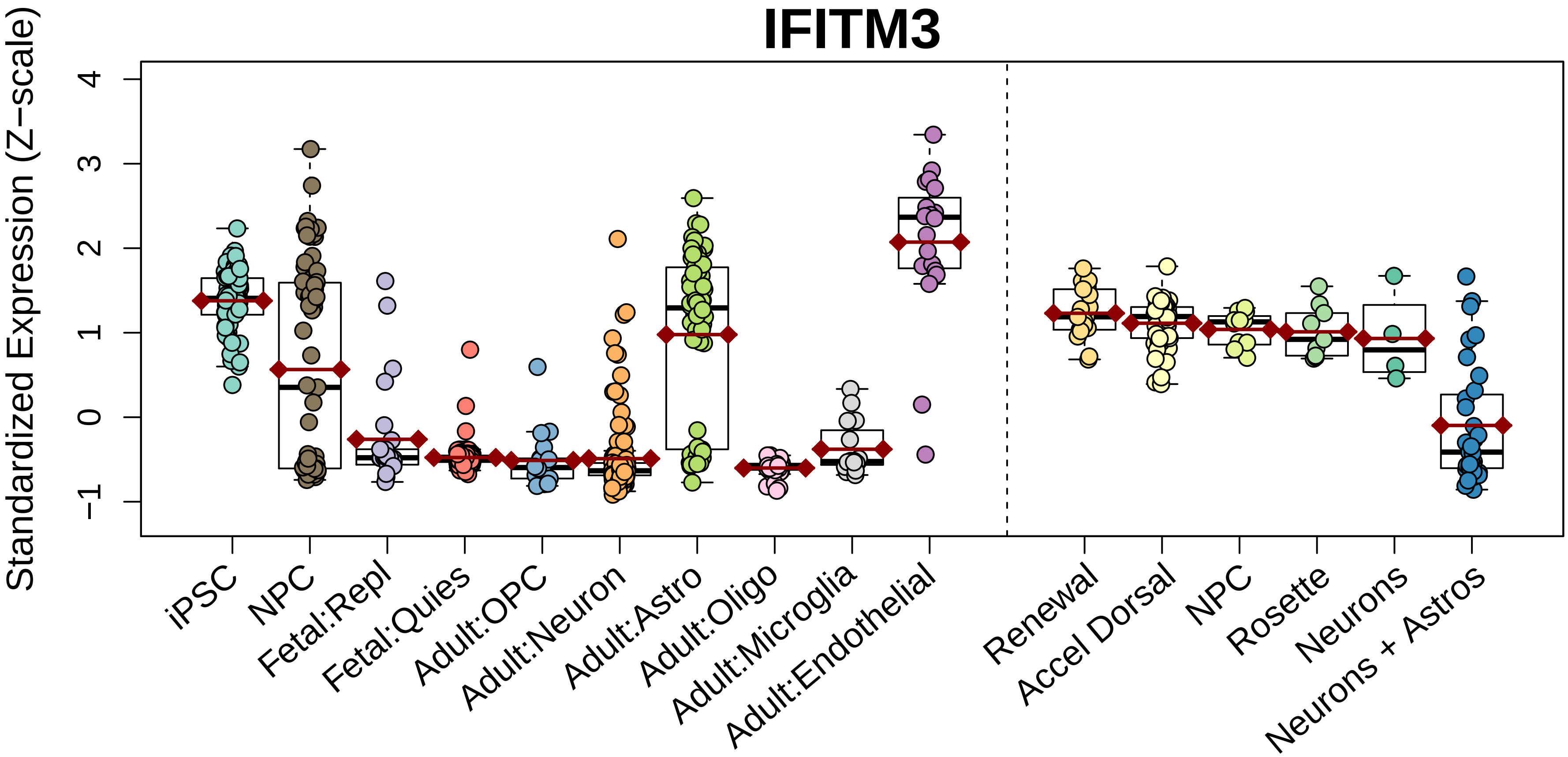
IFITM3
Standardized Expression (Z−scale)
4
3
2
1
0
−1
iPSC
NPC
Fetal:Repl
Fetal:Quies
Adult:OPC
Adult:Neuron
Adult:Astro
Adult:Oligo
Adult:Microglia
Adult:Endothelial
Renewal
Accel Dorsal
NPC
Rosette
Neurons
Neurons + Astros

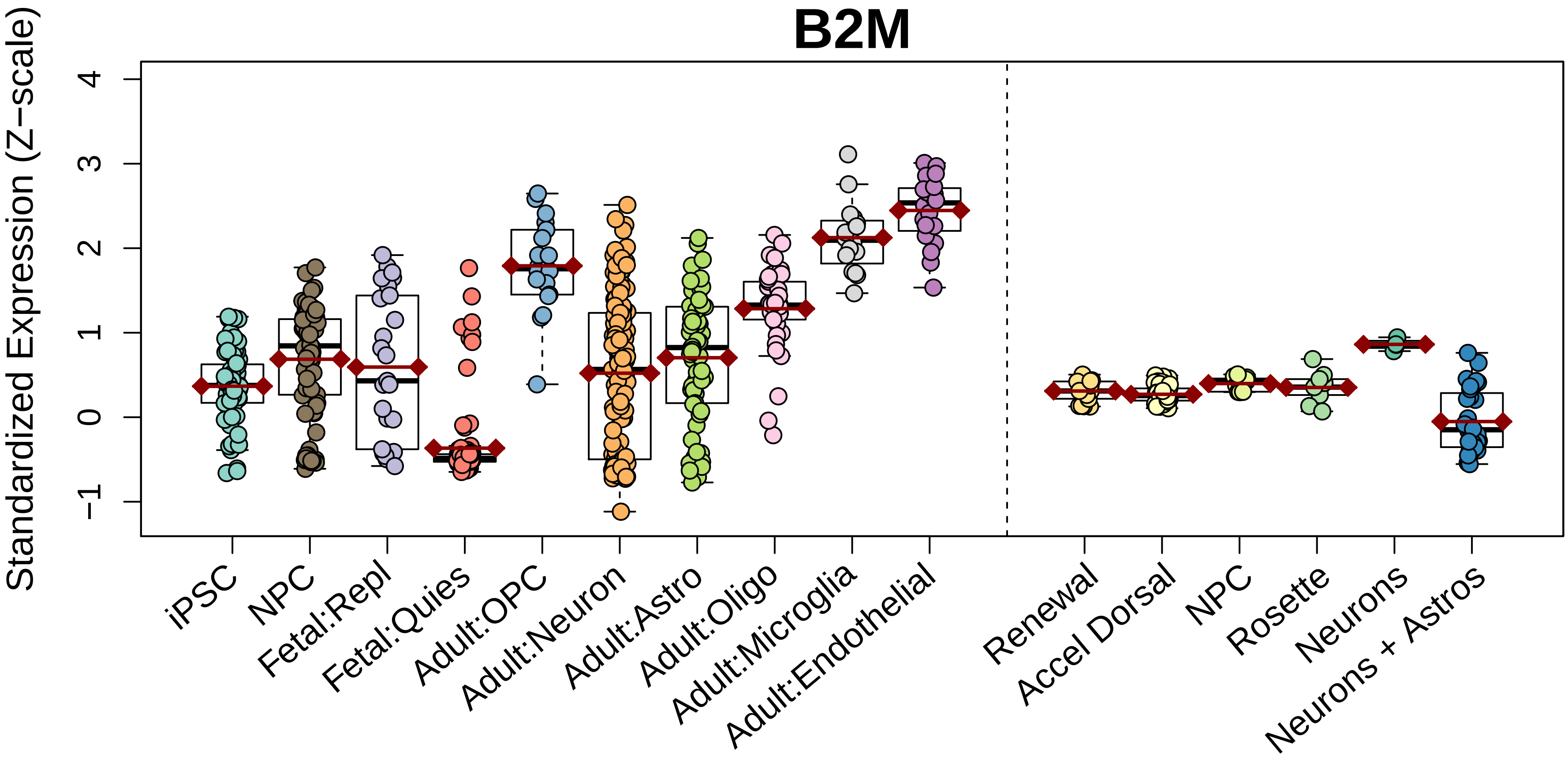

B2M
Standardized Expression (Z−scale)
−1
0
1
2
3
4
iPSC
NPC
Fetal:Repl
Fetal:Quies
Adult:OPC
Adult:Neuron
Adult:Astro
Adult:Oligo
Adult:Microglia
Adult:Endothelial
Renewal
Accel Dorsal
NPC
Rosette
Neurons
Neurons + Astros

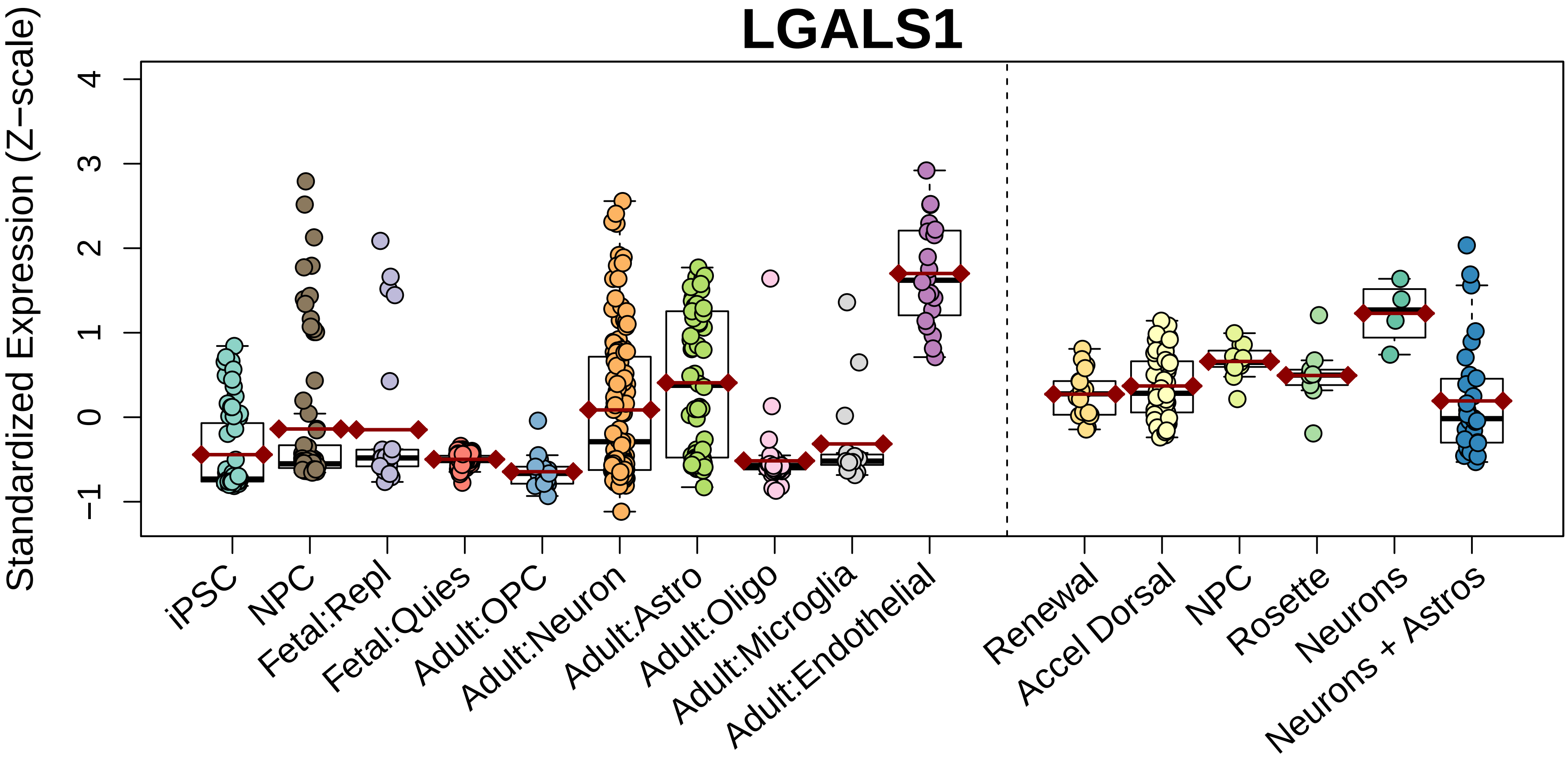
LGALS1
Standardized Expression (Z−scale)
4
3
2
1
0
−1
iPSC
NPC
Fetal:Repl
Fetal:Quies
Adult:OPC
Adult:Neuron
Adult:Astro
Adult:Oligo
Adult:Microglia
Adult:Endothelial
Renewal
Accel Dorsal
NPC
Rosette
Neurons
Neurons + Astros

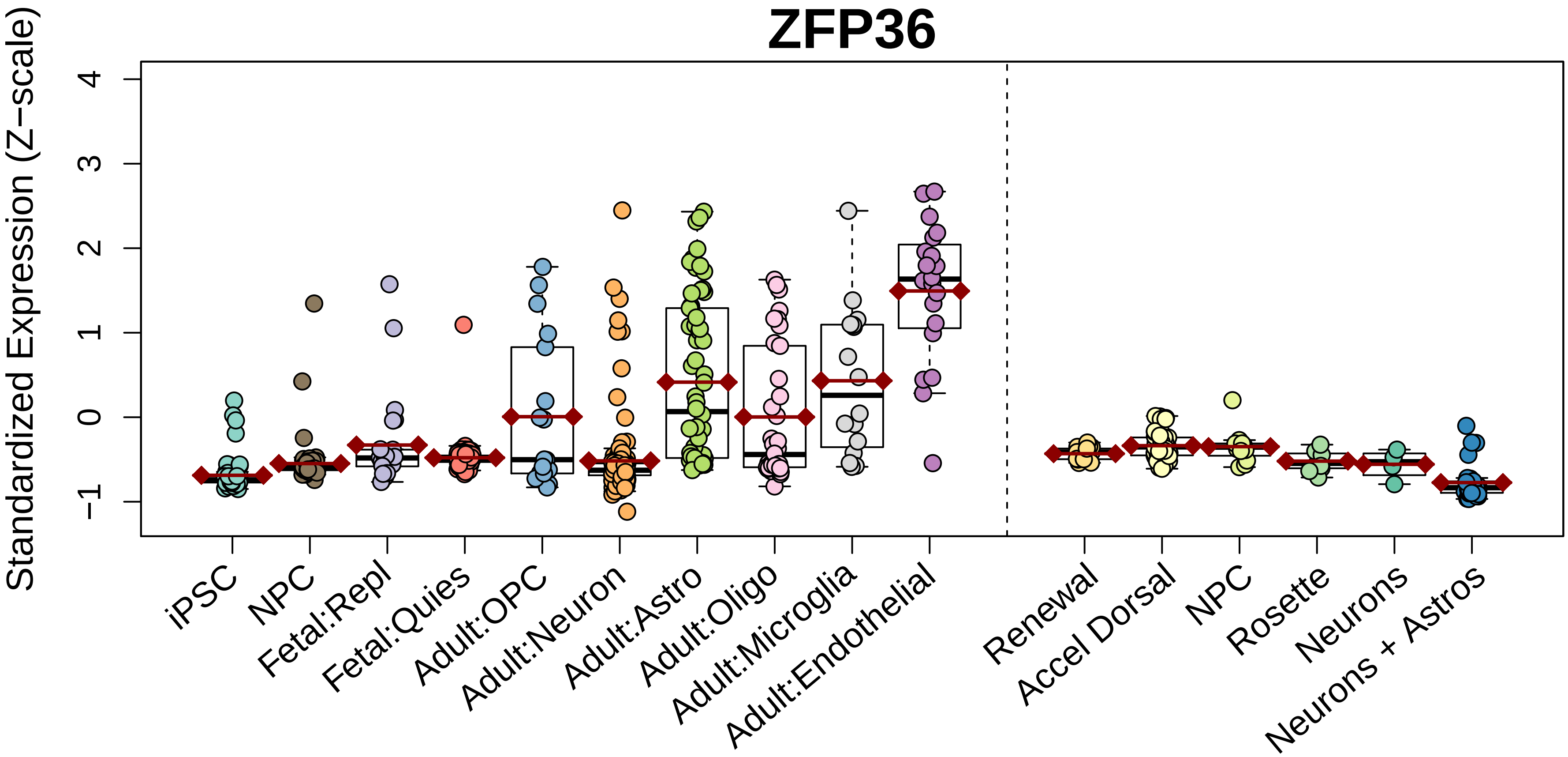
ZFP36
Standardized Expression (Z−scale)
4
3
2
1
0
−1
iPSC
NPC
Fetal:Repl
Fetal:Quies
Adult:OPC
Adult:Neuron
Adult:Astro
Adult:Oligo
Adult:Microglia
Adult:Endothelial
Renewal
Accel Dorsal
NPC
Rosette
Neurons
Neurons + Astros

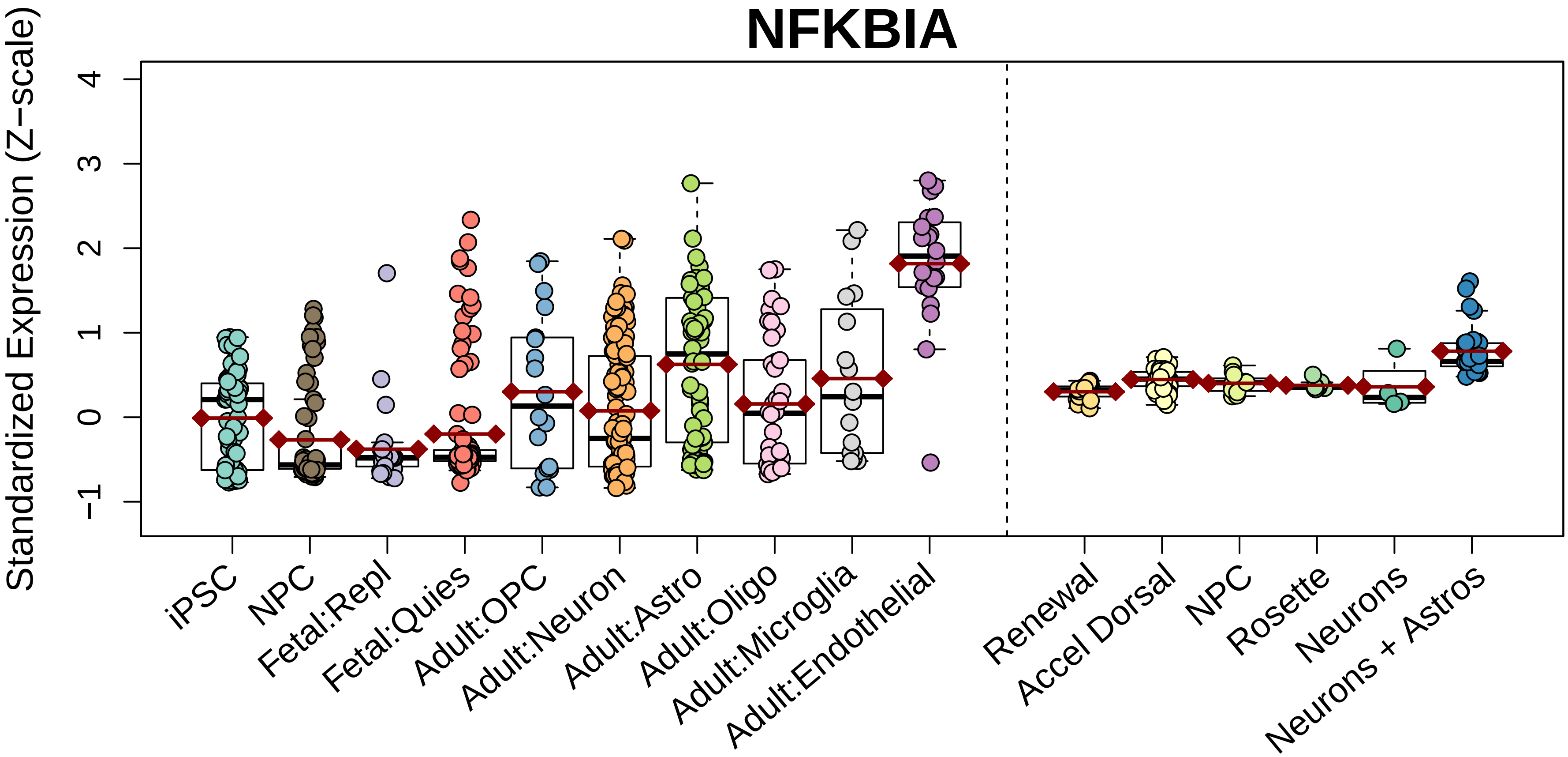
NFKBIA
Standardized Expression (Z−scale)
4
3
2
1
0
−1
iPSC
NPC
Fetal:Repl
Fetal:Quies
Adult:OPC
Adult:Neuron
Adult:Astro
Adult:Oligo
Adult:Microglia
Adult:Endothelial
Renewal
Accel Dorsal
NPC
Rosette
Neurons
Neurons + Astros

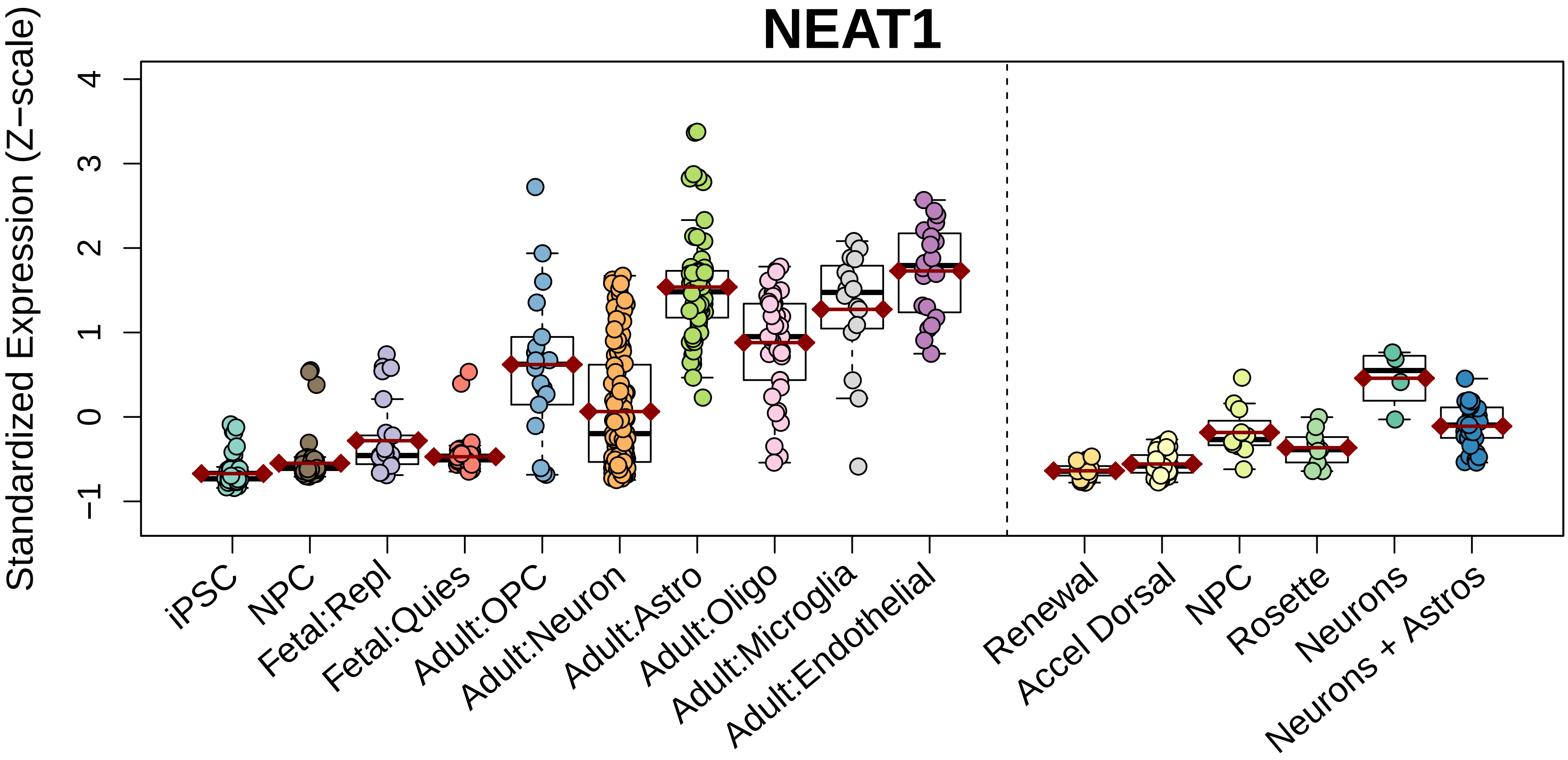

NEAT1
Standardized Expression (Z−scale)
4
3
2
1
0
−1
iPSC
NPC
Fetal:Repl
Fetal:Quies
Adult:OPC
Adult:Neuron
Adult:Astro
Adult:Oligo
Adult:Microglia
Adult:Endothelial
Renewal
Accel Dorsal
NPC
Rosette
Neurons
Neurons + Astros

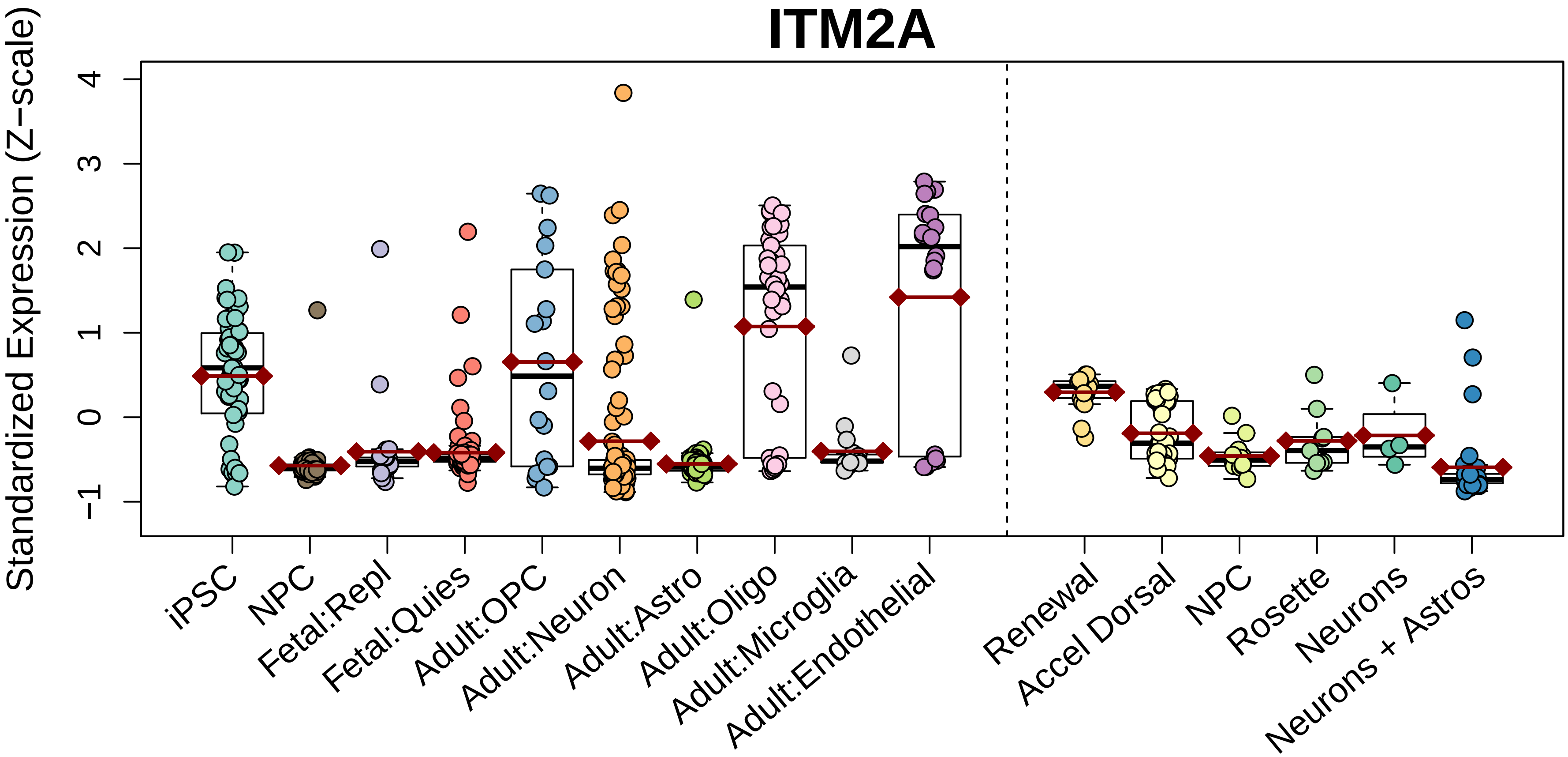

ITM2A
Standardized Expression (Z−scale)
4
3
2
1
0
−1
iPSC
NPC
Fetal:Repl
Fetal:Quies
Adult:OPC
Adult:Neuron
Adult:Astro
Adult:Oligo
Adult:Microglia
Adult:Endothelial
Renewal
Accel Dorsal
NPC
Rosette
Neurons
Neurons + Astros

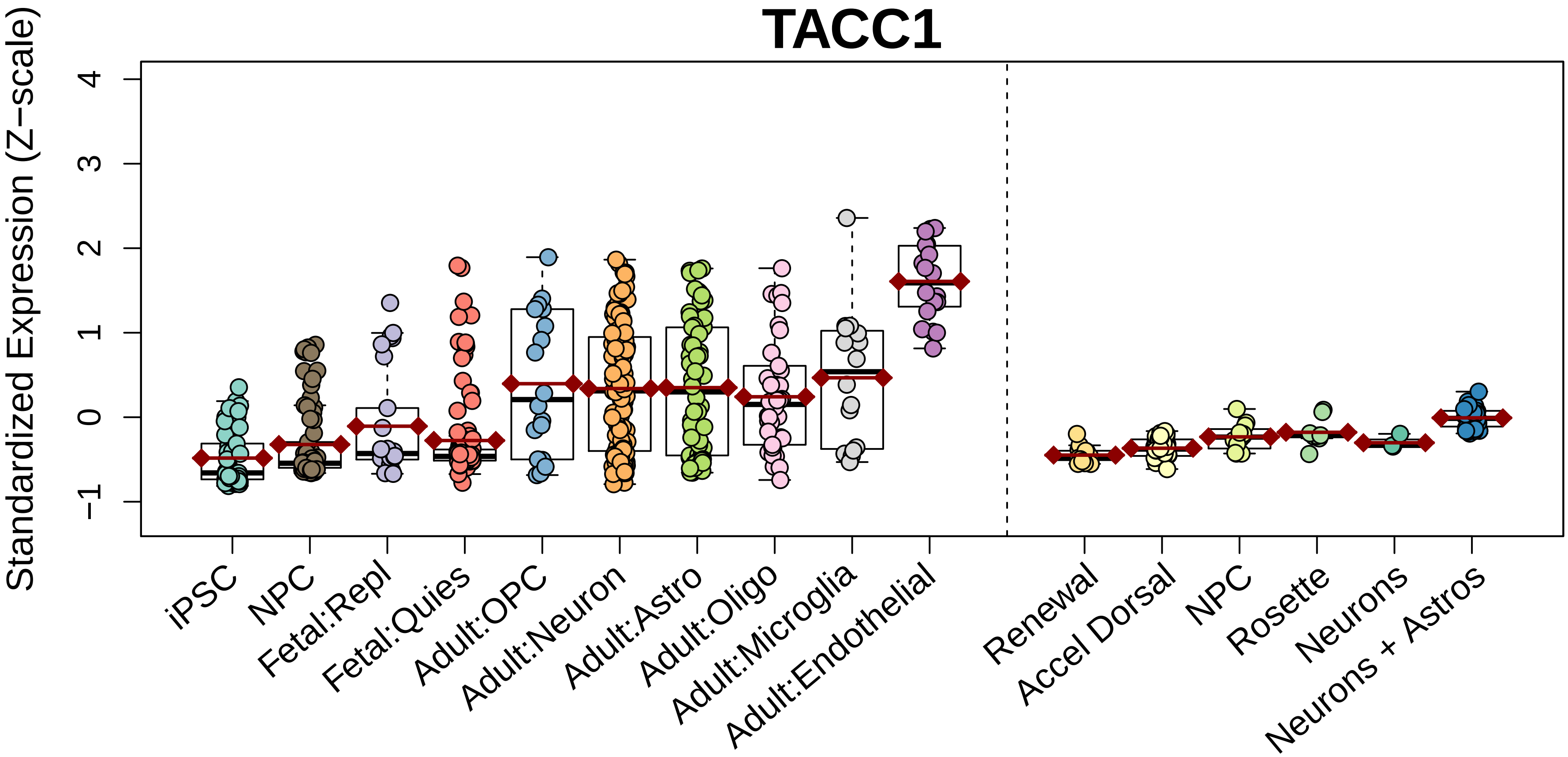

TACC1
Standardized Expression (Z−scale)
4
3
2
1
0
−1
iPSC
NPC
Fetal:Repl
Fetal:Quies
Adult:OPC
Adult:Neuron
Adult:Astro
Adult:Oligo
Adult:Microglia
Adult:Endothelial
Renewal
Accel Dorsal
NPC
Rosette
Neurons
Neurons + Astros

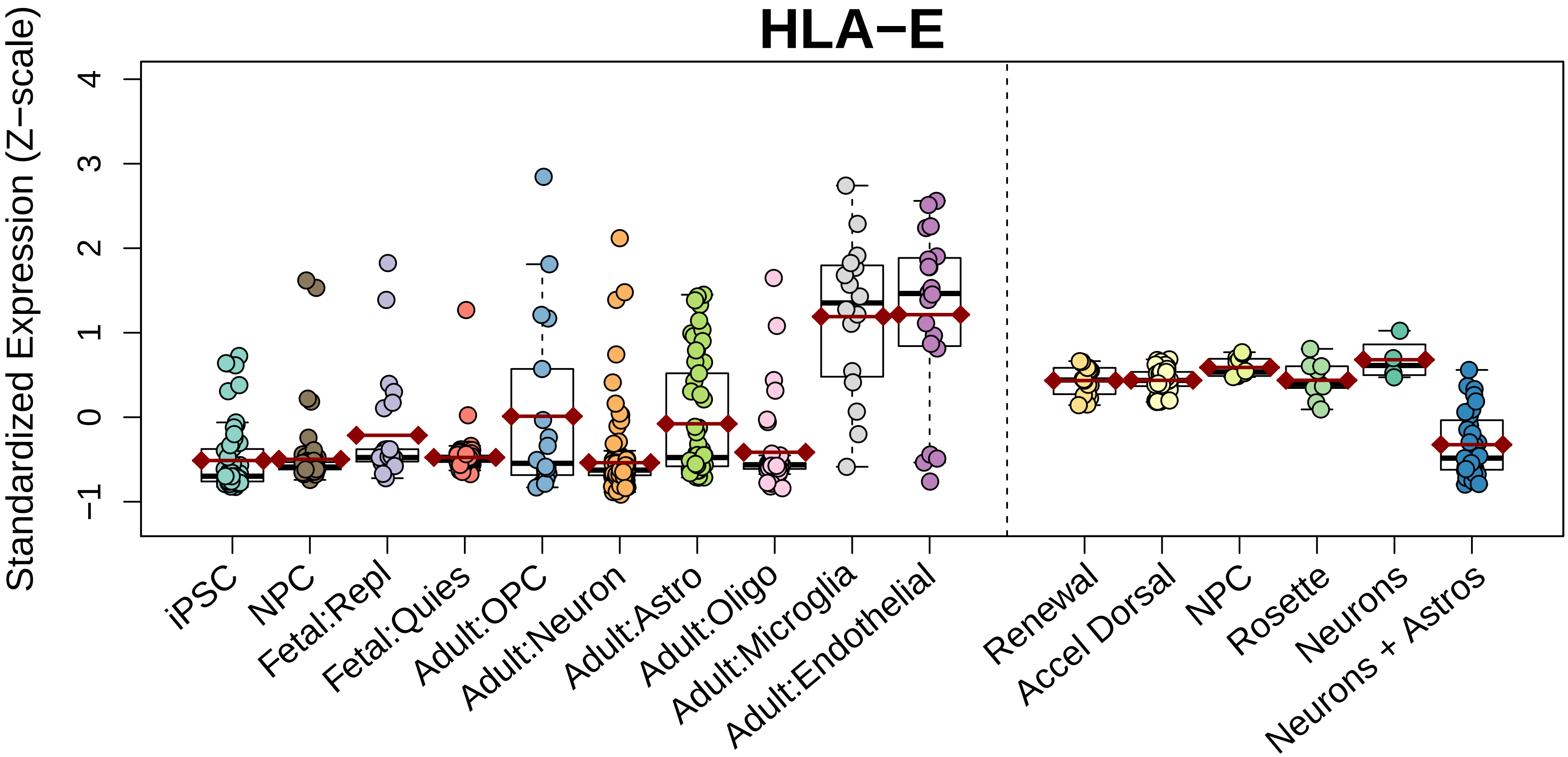
HLA−E
Standardized Expression (Z−scale)
4
3
2
1
0
−1
iPSC
NPC
Fetal:Repl
Fetal:Quies
Adult:OPC
Adult:Neuron
Adult:Astro
Adult:Oligo
Adult:Microglia
Adult:Endothelial
Renewal
Accel Dorsal
NPC
Rosette
Neurons
Neurons + Astros

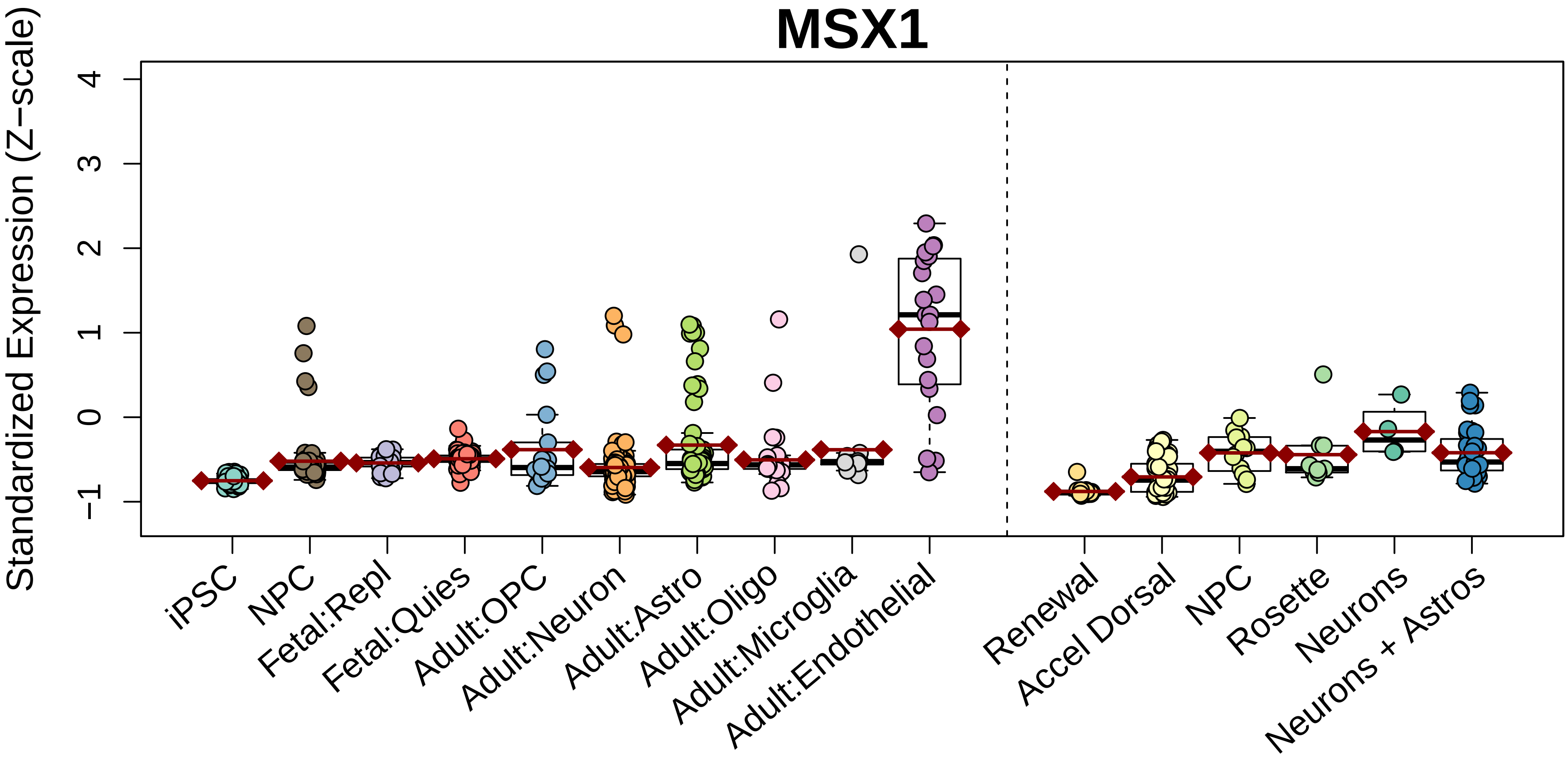

MSX1
Standardized Expression (Z−scale)
4
3
2
1
0
−1
iPSC
NPC
Fetal:Repl
Fetal:Quies
Adult:OPC
Adult:Neuron
Adult:Astro
Adult:Oligo
Adult:Microglia
Adult:Endothelial
Renewal
Accel Dorsal
NPC
Rosette
Neurons
Neurons + Astros

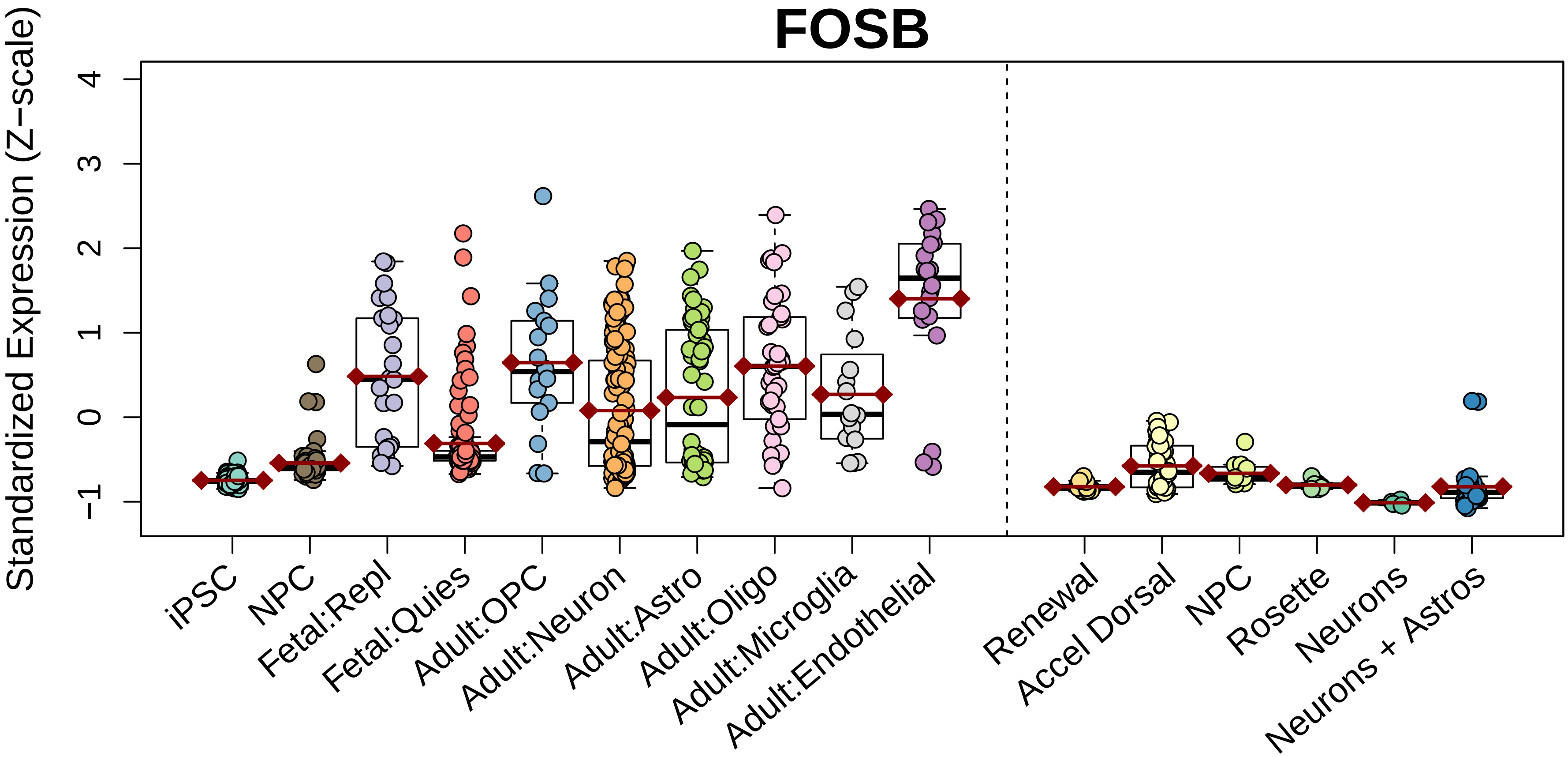

FOSB
Standardized Expression (Z−scale)
4
3
2
1
0
−1
iPSC
NPC
Fetal:Repl
Fetal:Quies
Adult:OPC
Adult:Neuron
Adult:Astro
Adult:Oligo
Adult:Microglia
Adult:Endothelial
Renewal
Accel Dorsal
NPC
Rosette
Neurons
Neurons + Astros

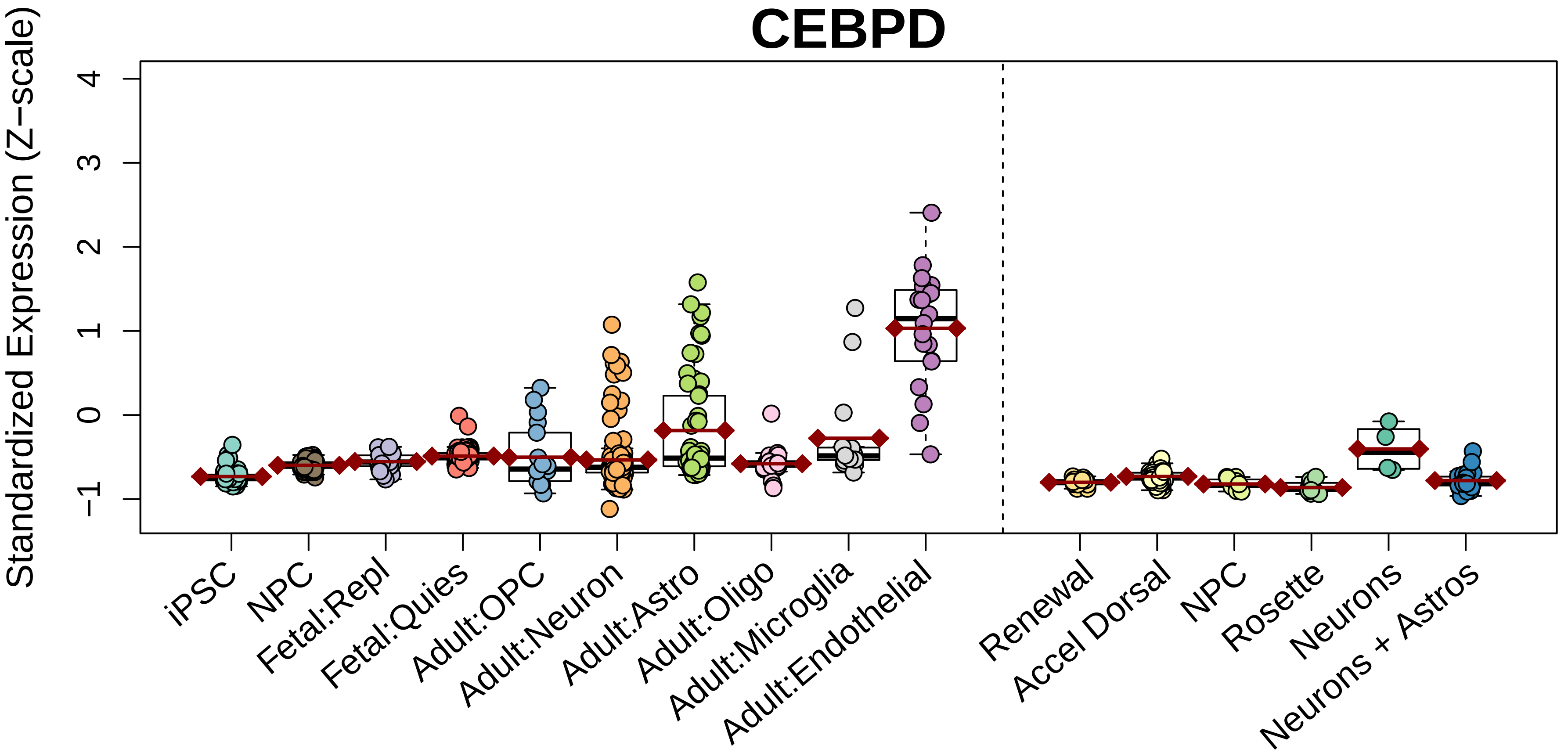

CEBPD
Standardized Expression (Z−scale)
4
3
2
1
0
−1
iPSC
NPC
Fetal:Repl
Fetal:Quies
Adult:OPC
Adult:Neuron
Adult:Astro
Adult:Oligo
Adult:Microglia
Adult:Endothelial
Renewal
Accel Dorsal
NPC
Rosette
Neurons
Neurons + Astros

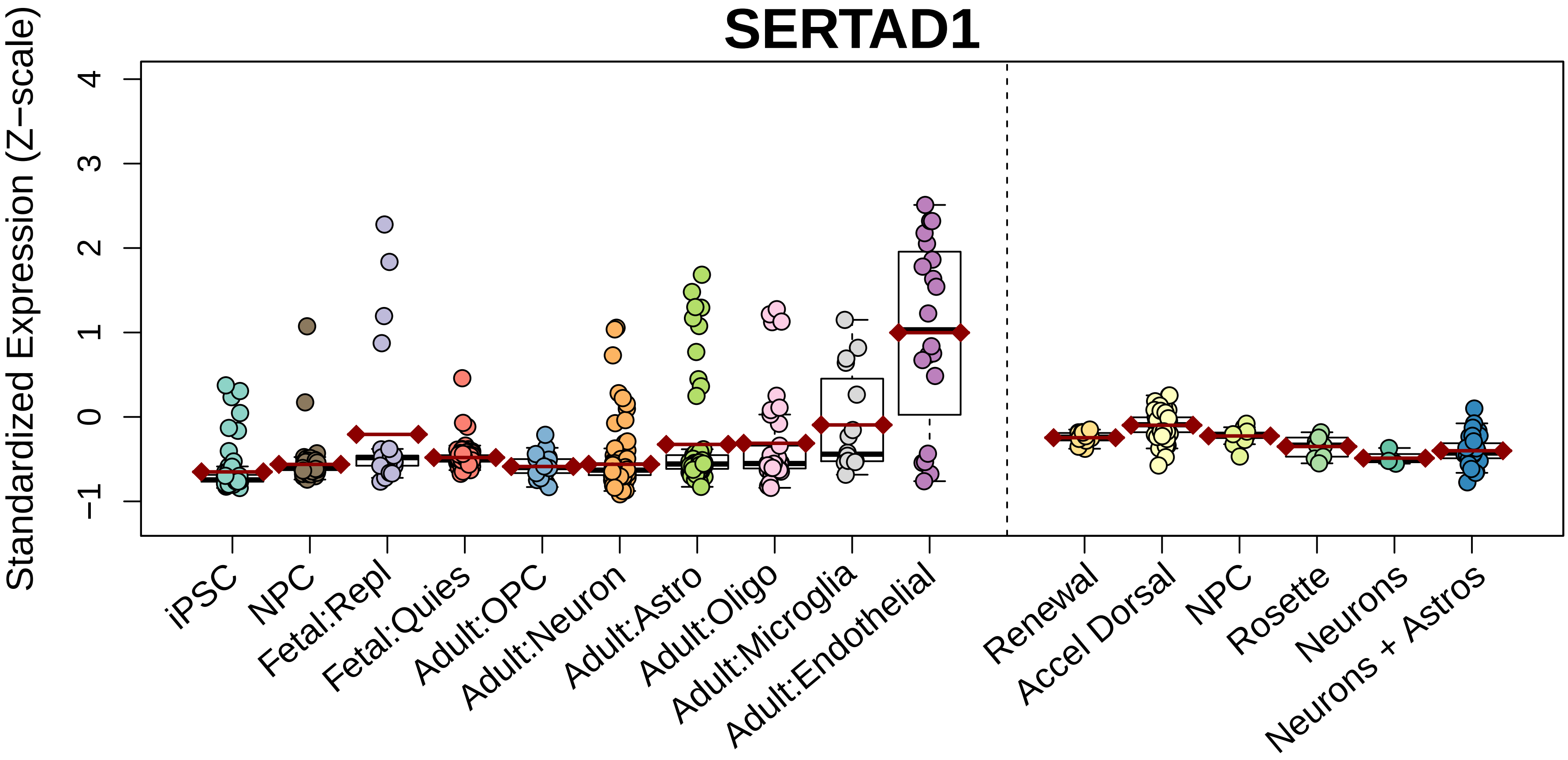
SERTAD1
Standardized Expression (Z−scale)
4
3
2
1
0
−1
iPSC
NPC
Fetal:Repl
Fetal:Quies
Adult:OPC
Adult:Neuron
Adult:Astro
Adult:Oligo
Adult:Microglia
Adult:Endothelial
Renewal
Accel Dorsal
NPC
Rosette
Neurons
Neurons + Astros

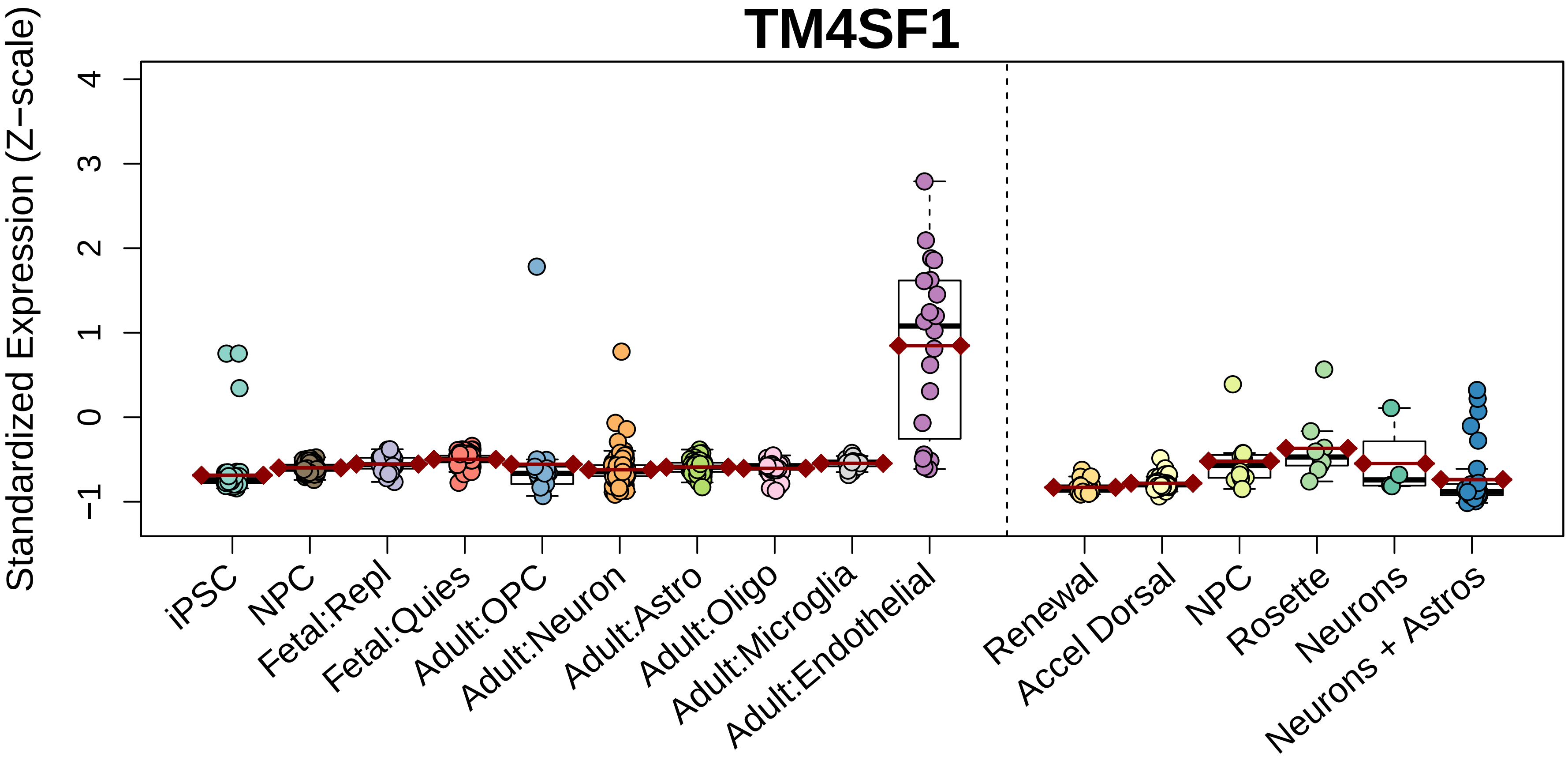
TM4SF1
Standardized Expression (Z−scale)
4
3
2
1
0
−1
iPSC
NPC
Fetal:Repl
Fetal:Quies
Adult:OPC
Adult:Neuron
Adult:Astro
Adult:Oligo
Adult:Microglia
Adult:Endothelial
Renewal
Accel Dorsal
NPC
Rosette
Neurons
Neurons + Astros

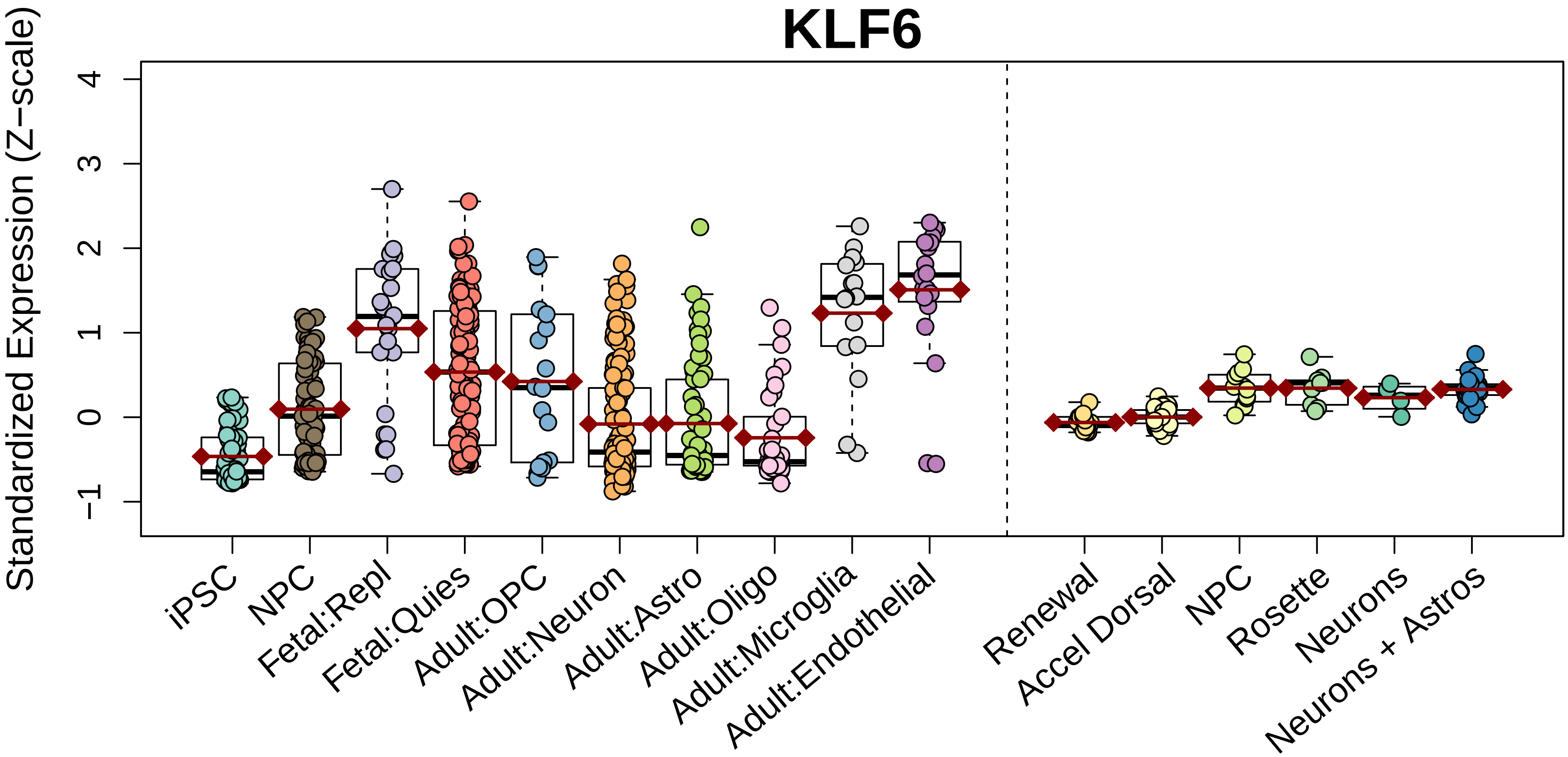
KLF6
Standardized Expression (Z−scale)
4
3
2
1
0
−1
iPSC
NPC
Fetal:Repl
Fetal:Quies
Adult:OPC
Adult:Neuron
Adult:Astro
Adult:Oligo
Adult:Microglia
Adult:Endothelial
Renewal
Accel Dorsal
NPC
Rosette
Neurons
Neurons + Astros

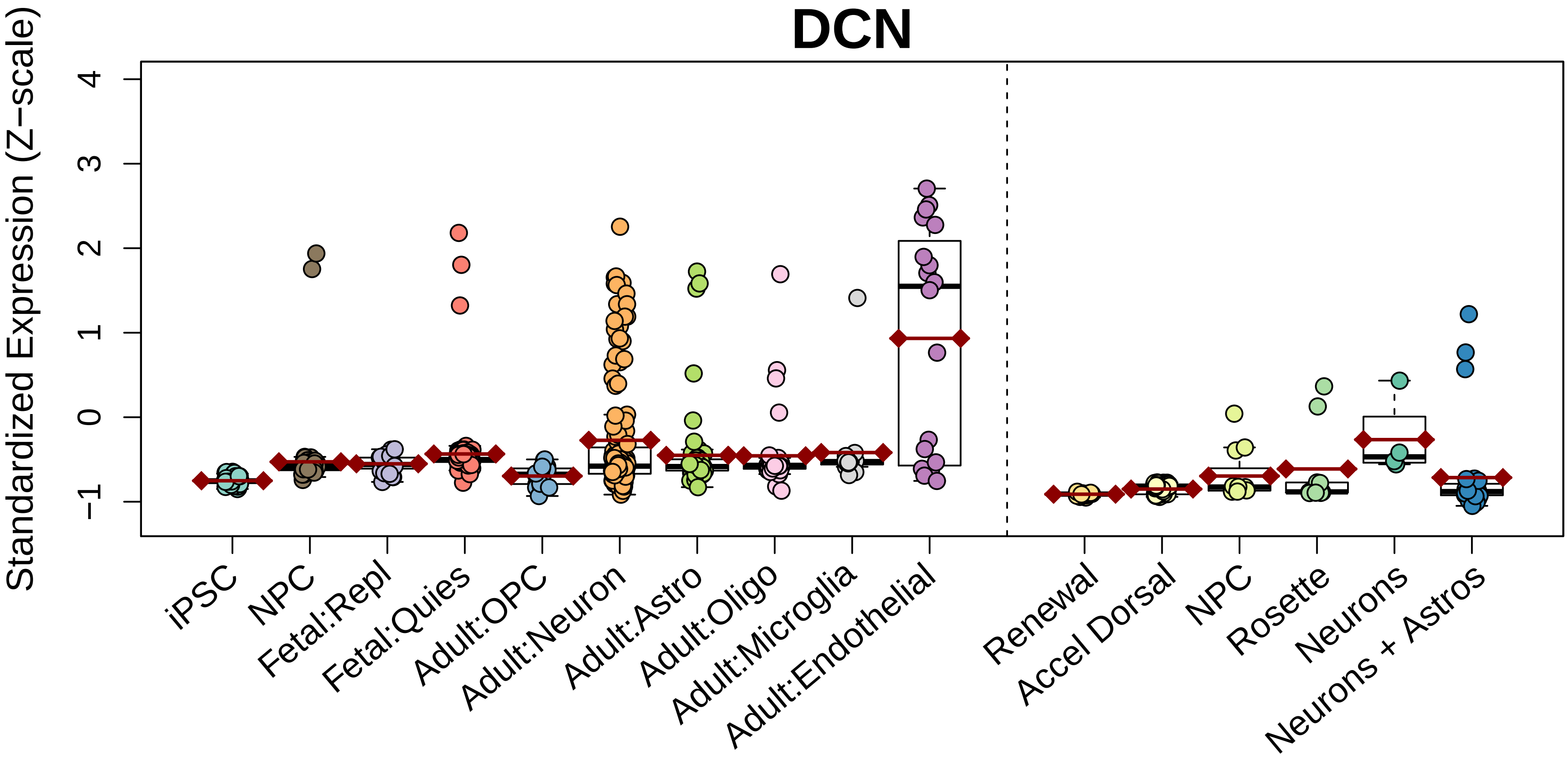

DCN
Standardized Expression (Z−scale)
4
3
2
1
0
−1
iPSC
NPC
Fetal:Repl
Fetal:Quies
Adult:OPC
Adult:Neuron
Adult:Astro
Adult:Oligo
Adult:Microglia
Adult:Endothelial
Renewal
Accel Dorsal
NPC
Rosette
Neurons
Neurons + Astros

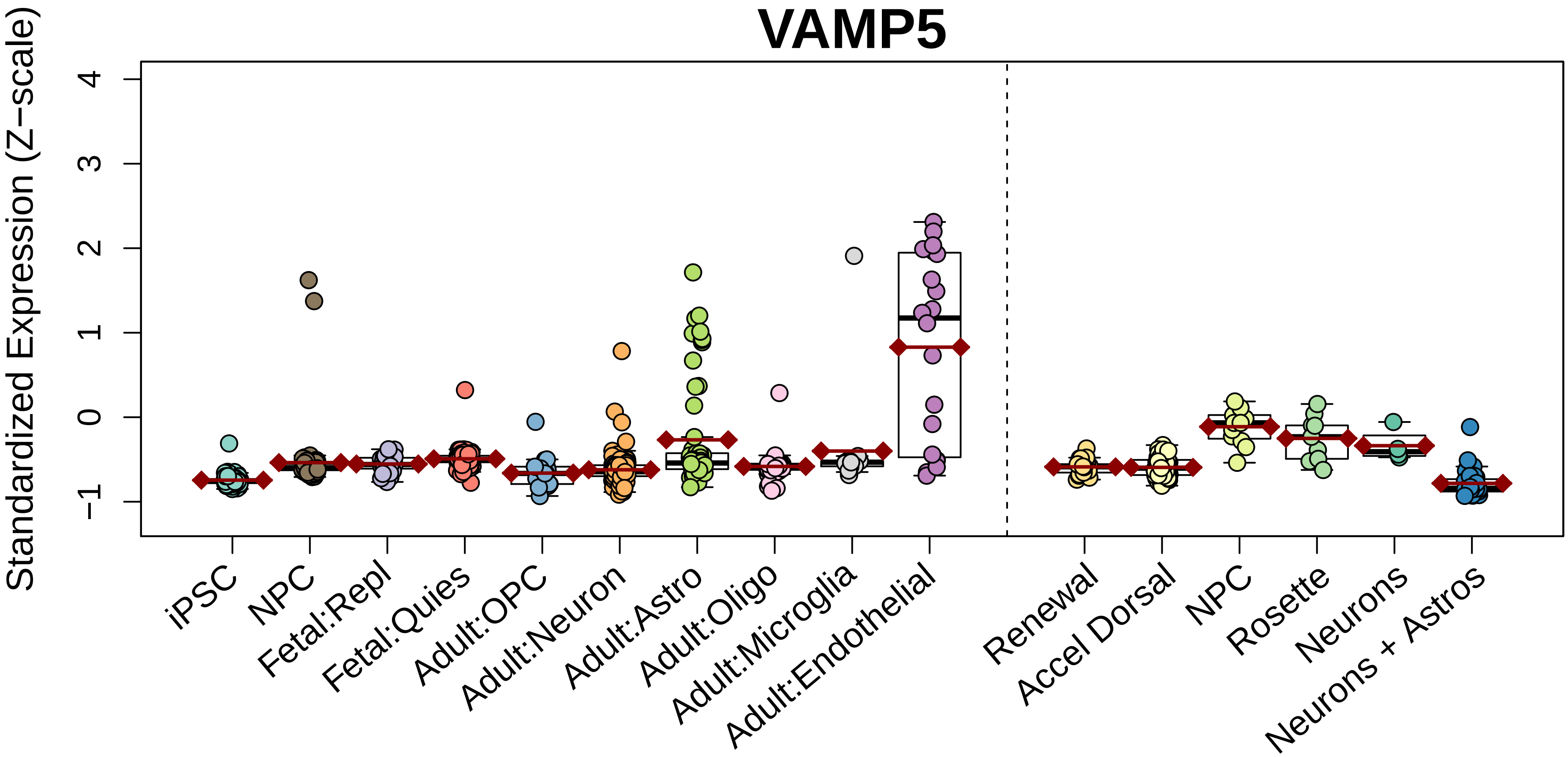
VAMP5
Standardized Expression (Z−scale)
4
3
2
1
0
−1
iPSC
NPC
Fetal:Repl
Fetal:Quies
Adult:OPC
Adult:Neuron
Adult:Astro
Adult:Oligo
Adult:Microglia
Adult:Endothelial
Renewal
Accel Dorsal
NPC
Rosette
Neurons
Neurons + Astros

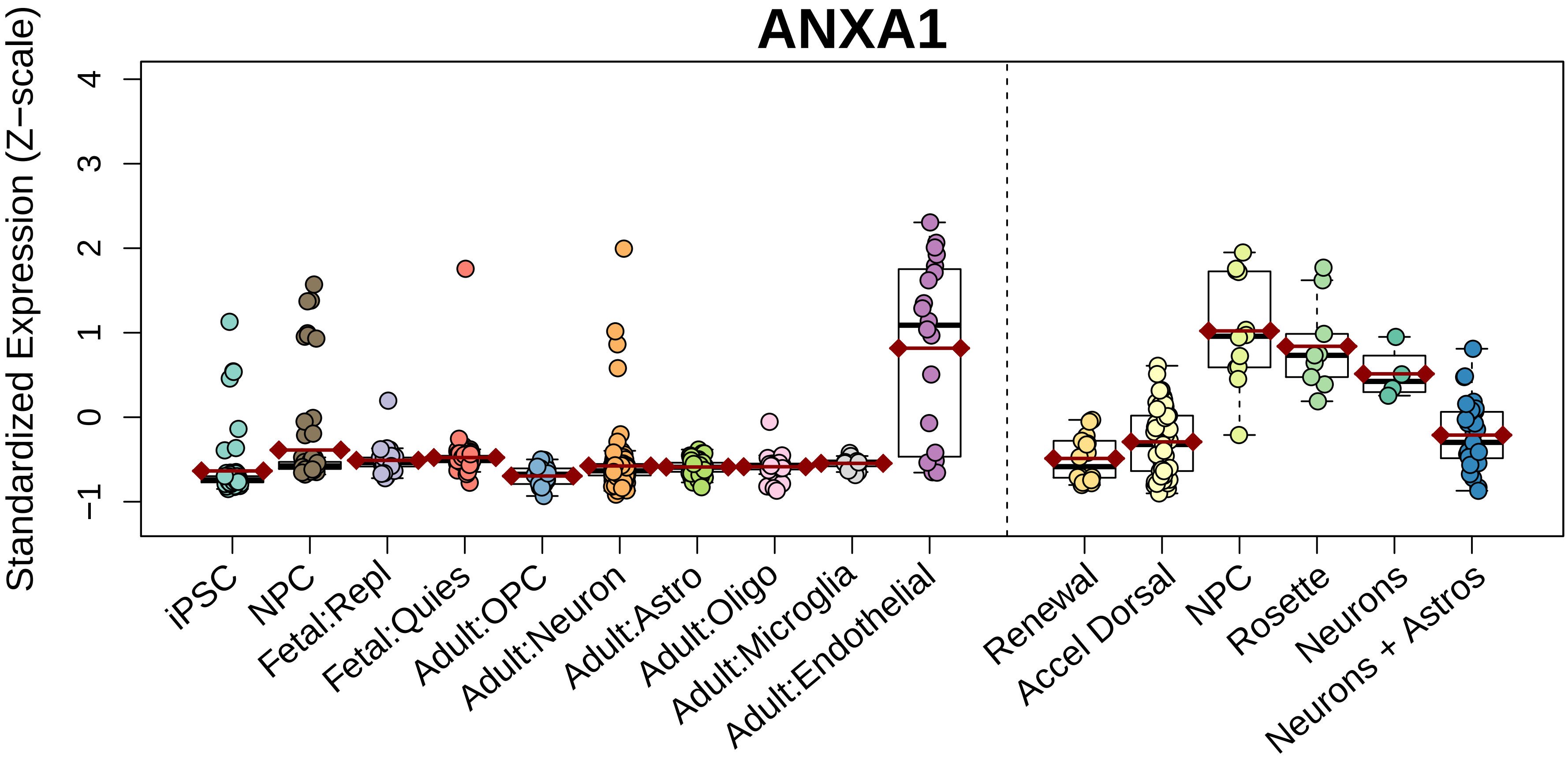
ANXA1
Standardized Expression (Z−scale)
4
3
2
1
0
−1
iPSC
NPC
Fetal:Repl
Fetal:Quies
Adult:OPC
Adult:Neuron
Adult:Astro
Adult:Oligo
Adult:Microglia
Adult:Endothelial
Renewal
Accel Dorsal
NPC
Rosette
Neurons
Neurons + Astros
