# Supplementary material for: Dissecting transcriptomic signatures of neuronal differentiation and maturation using iPSCs
Source: Nat Commun. 2020 Jan 23;11:462. doi: 10.1038/s41467-019-14266-z (PMC6978526; doi:10.1038/s41467-019-14266-z)
Supplement: Supplementary file 11 — Supplementary Data 7 [file 41467_2019_14266_MOESM11_ESM.pdf]

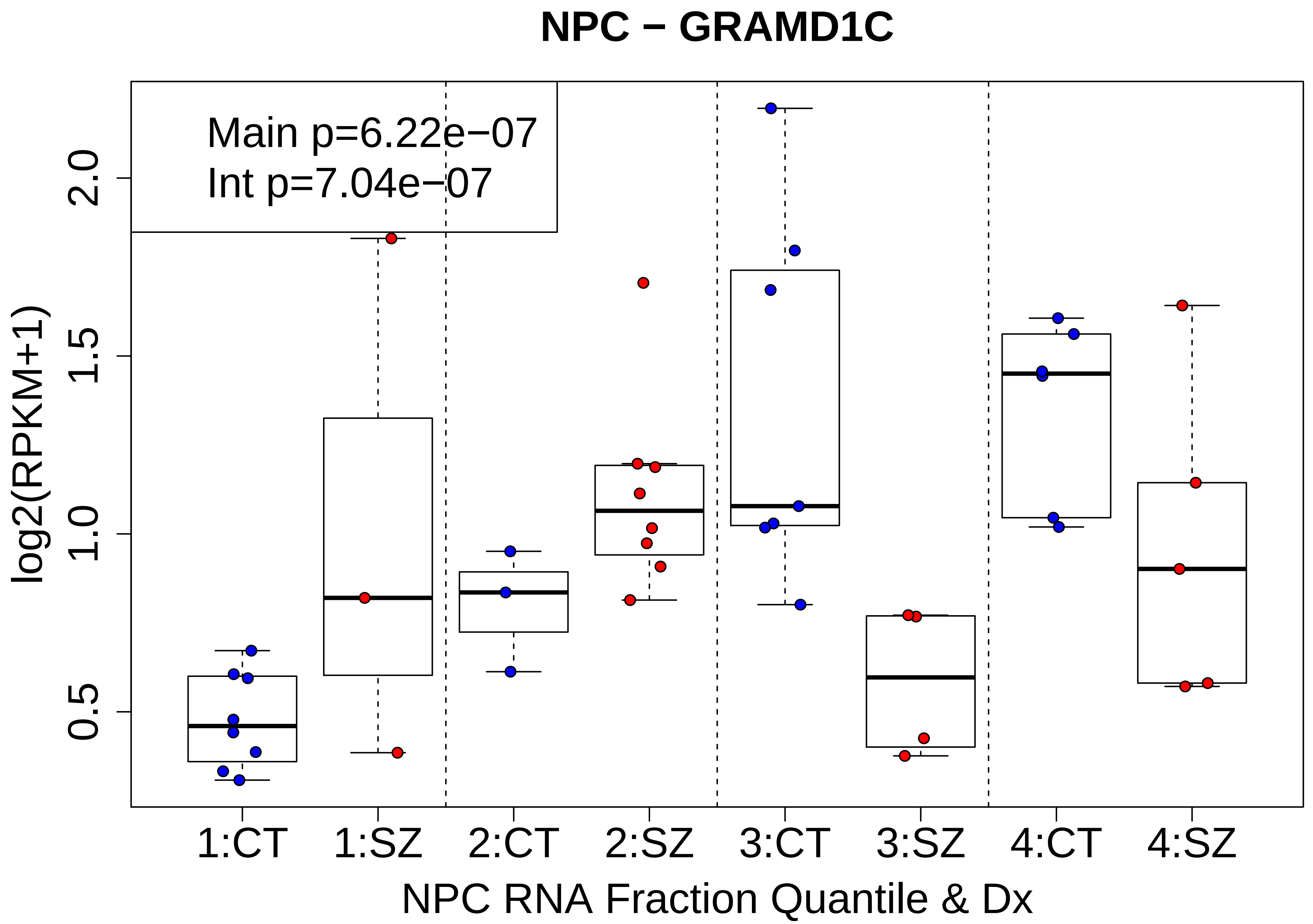

NPC − GRAMD1C
Main p=6.22e−07
Int p=7.04e−07
log2(RPKM+1)
0.5
1.0
1.5
2.0
1:CT
1:SZ
2:CT
2:SZ
3:CT
3:SZ
4:CT
4:SZ
NPC RNA Fraction Quantile & Dx

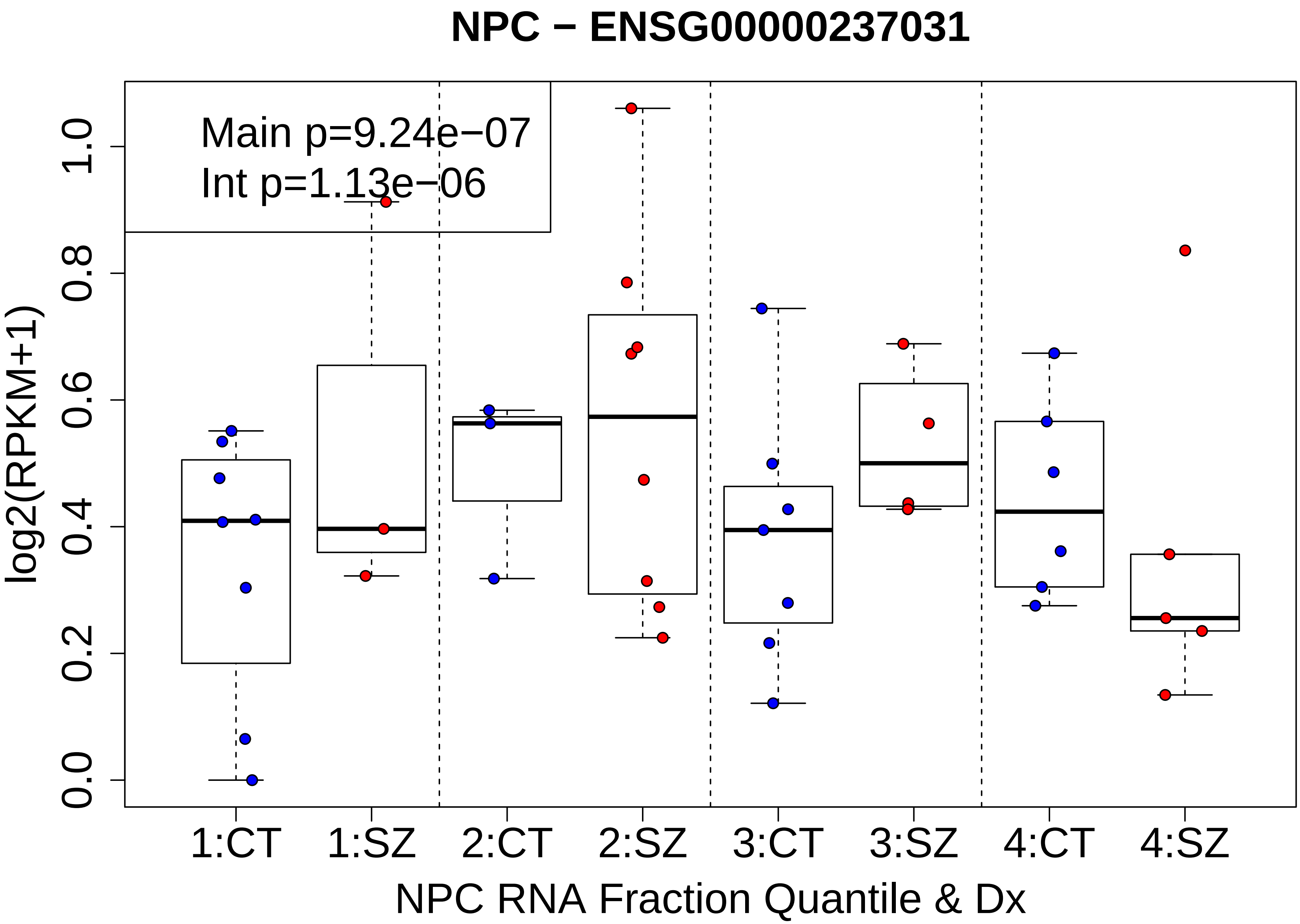

NPC − ENSG00000237031
Main p=9.24e−07
Int p=1.13e−06
log2(RPKM+1)
0.0
0.2
0.4
0.6
0.8
1.0
1:CT
1:SZ
2:CT
2:SZ
3:CT
3:SZ
4:CT
4:SZ
NPC RNA Fraction Quantile & Dx

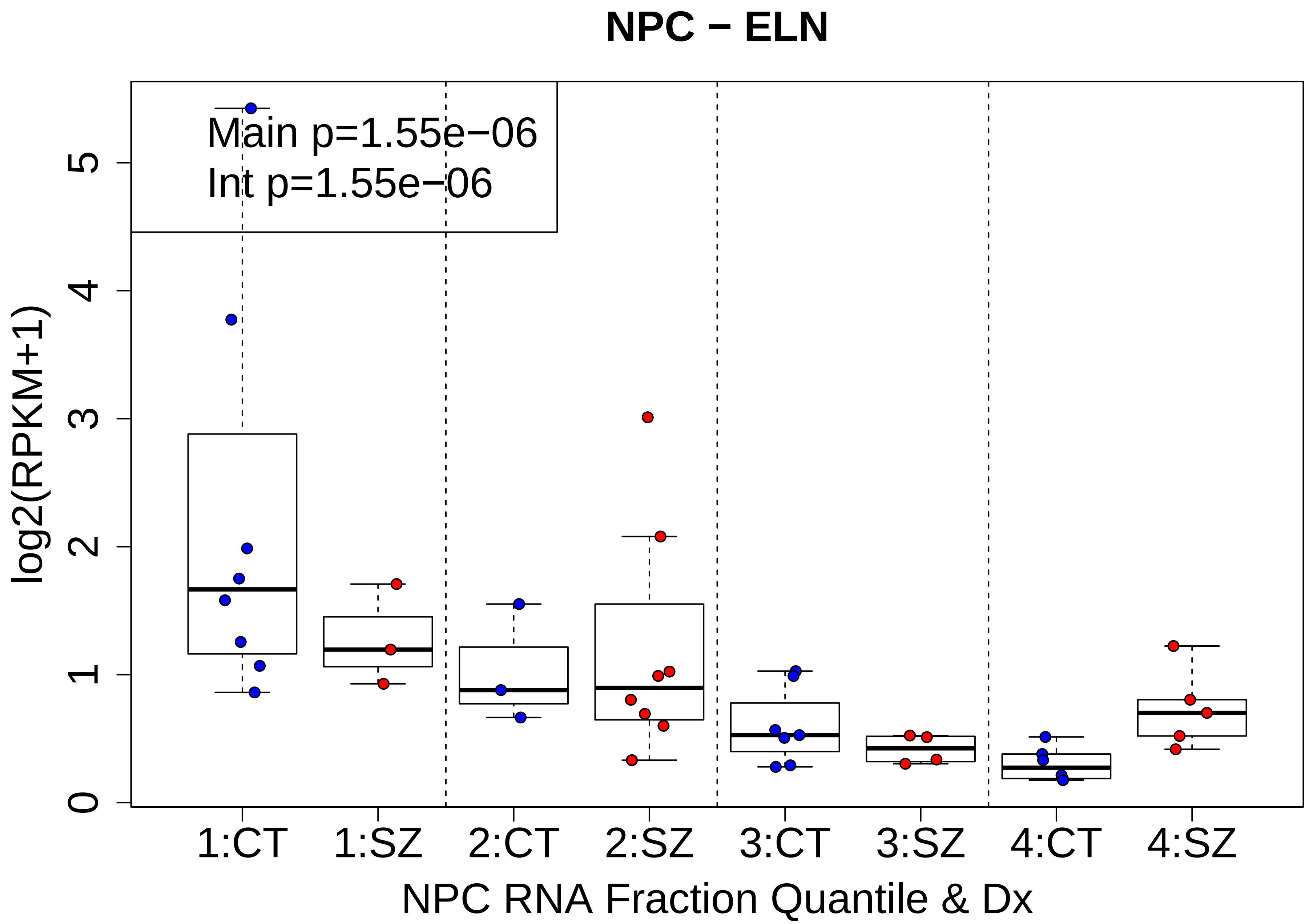
NPC − ELN
Main p=1.55e−06
Int p=1.55e−06
log2(RPKM+1)
0
1
2
3
4
5
1:CT
1:SZ
2:CT
2:SZ
3:CT
3:SZ
4:CT
4:SZ
NPC RNA Fraction Quantile & Dx

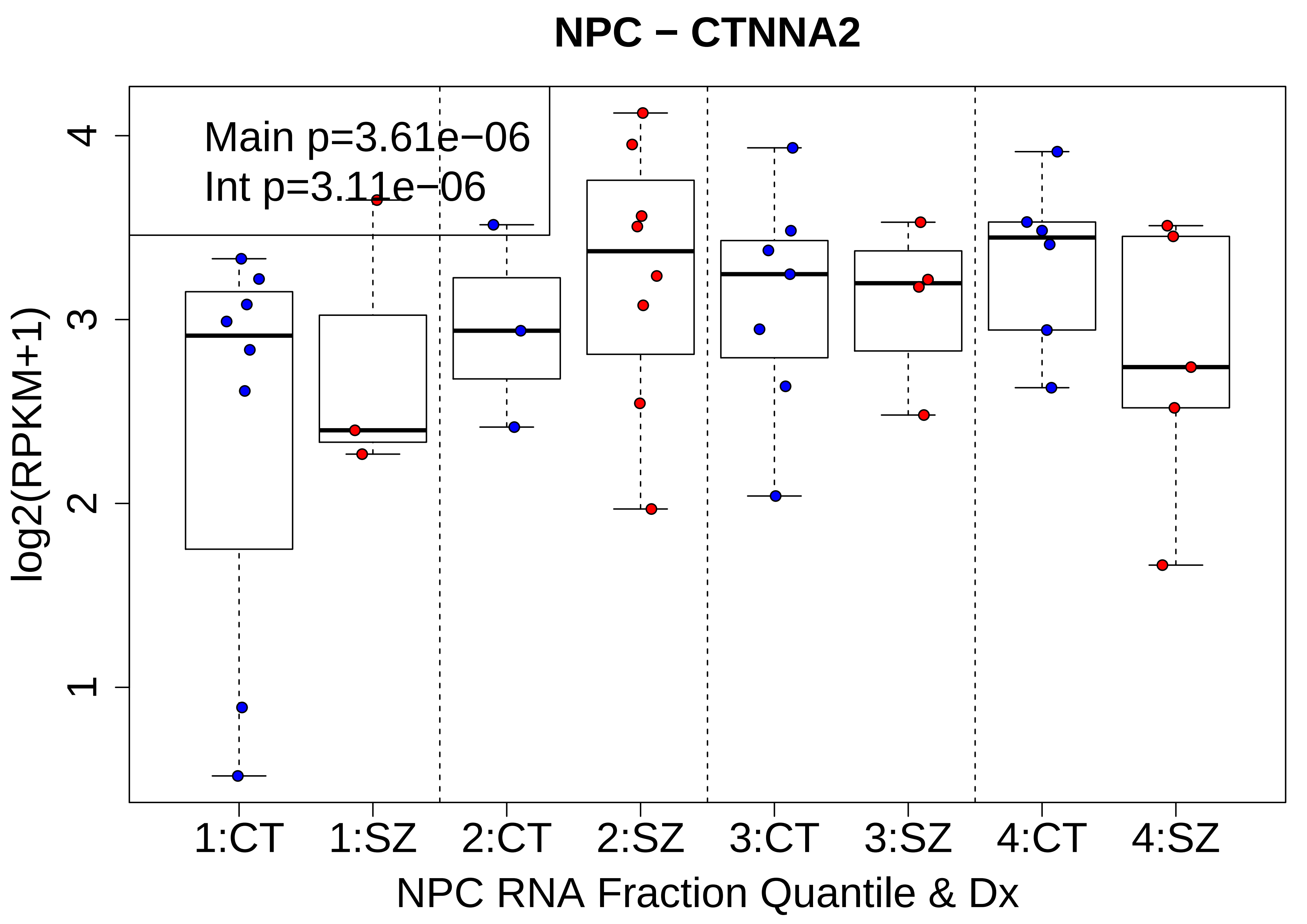

NPC − CTNNA2
Main p=3.61e−06
Int p=3.11e−06
log2(RPKM+1)
1
2
3
4
1:CT
1:SZ
2:CT
2:SZ
3:CT
3:SZ
4:CT
4:SZ
NPC RNA Fraction Quantile & Dx

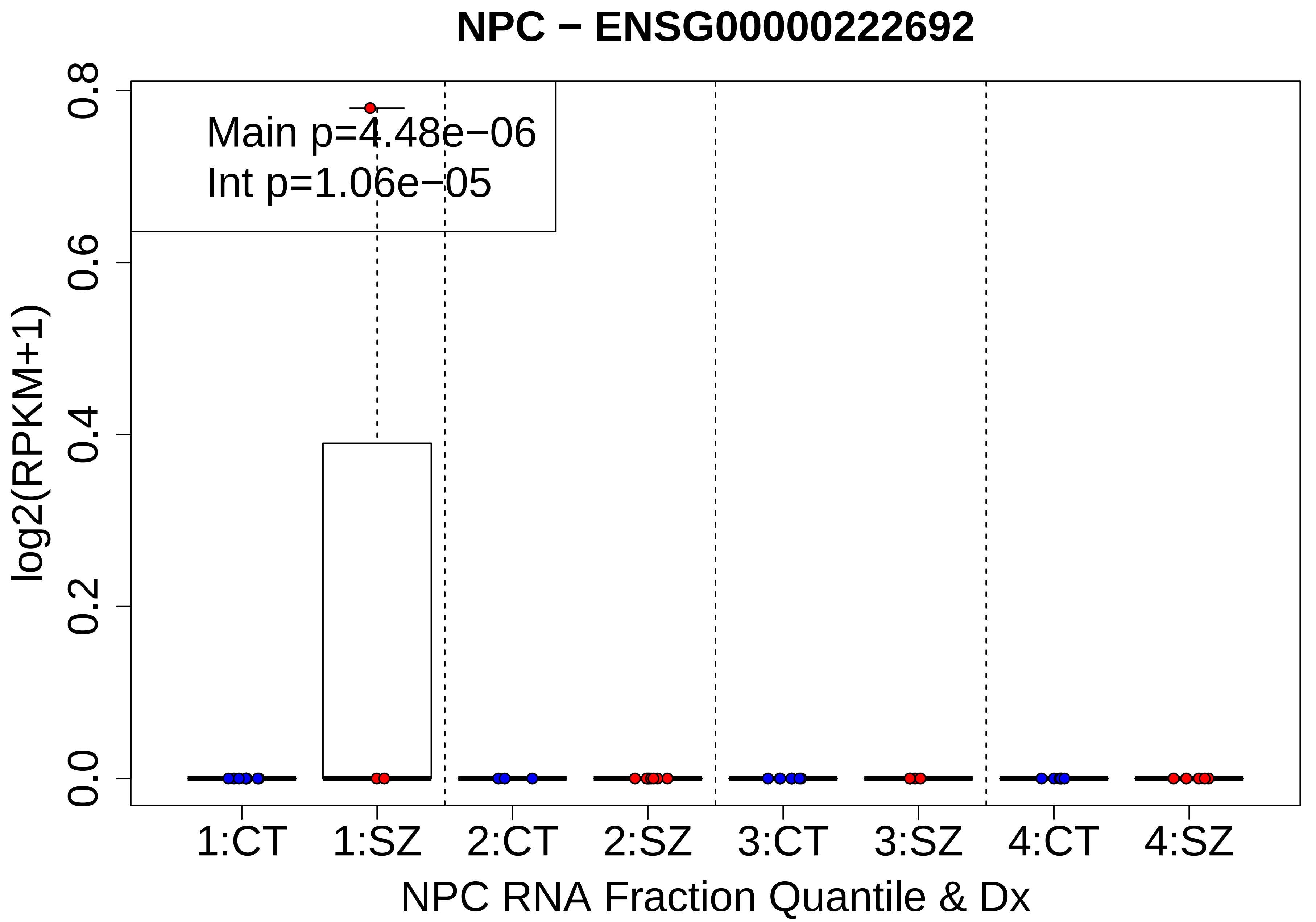
NPC − ENSG00000222692
Main p=4.48e−06
Int p=1.06e−05
log2(RPKM+1)
0.0
0.2
0.4
0.6
0.8
1:CT
1:SZ
2:CT
2:SZ
3:CT
3:SZ
4:CT
4:SZ
NPC RNA Fraction Quantile & Dx

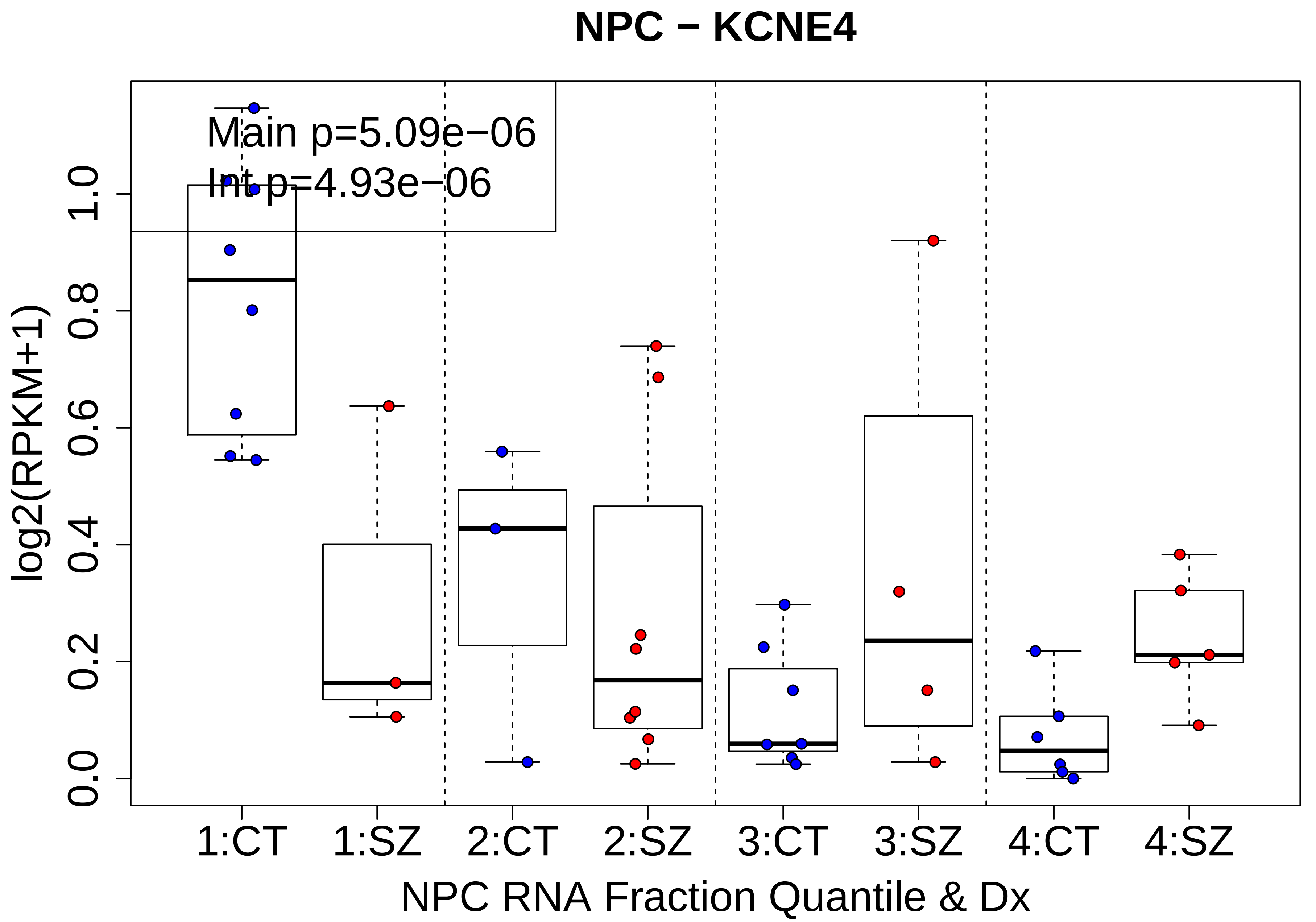
NPC − KCNE4
Main p=5.09e−06
Int p=4.93e−06
log2(RPKM+1)
0.0
0.2
0.4
0.6
0.8
1.0
1:CT
1:SZ
2:CT
2:SZ
3:CT
3:SZ
4:CT
4:SZ
NPC RNA Fraction Quantile & Dx

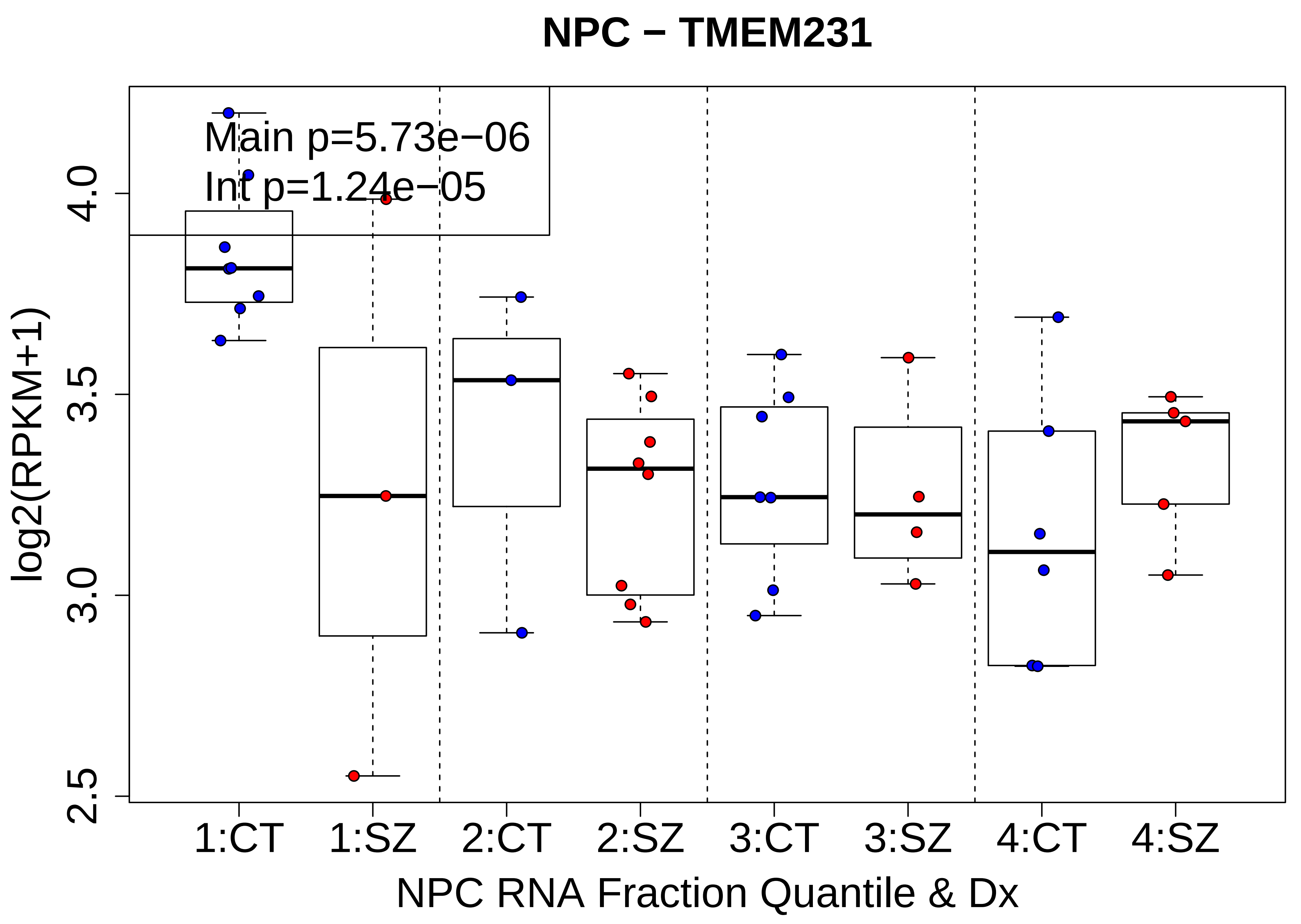
NPC − TMEM231
Main p=5.73e−06
Int p=1.24e−05
log2(RPKM+1)
4.0
3.5
3.0
2.5
1:CT
1:SZ
2:CT
2:SZ
3:CT
3:SZ
4:CT
4:SZ
NPC RNA Fraction Quantile & Dx

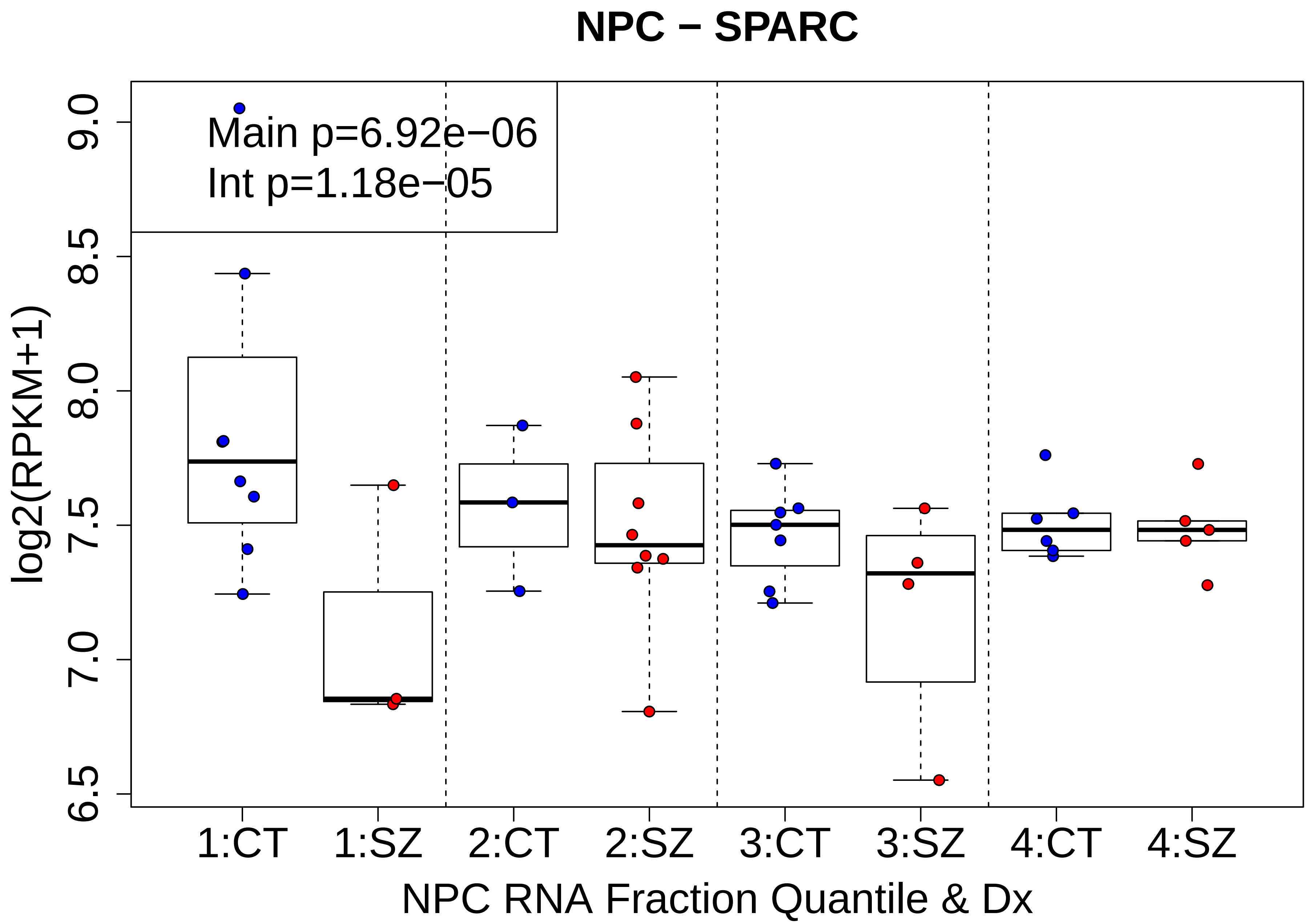
NPC − SPARC
Main p=6.92e−06
Int p=1.18e−05
log2(RPKM+1)
6.5
7.0
7.5
8.0
8.5
9.0
1:CT
1:SZ
2:CT
2:SZ
3:CT
3:SZ
4:CT
4:SZ
NPC RNA Fraction Quantile & Dx

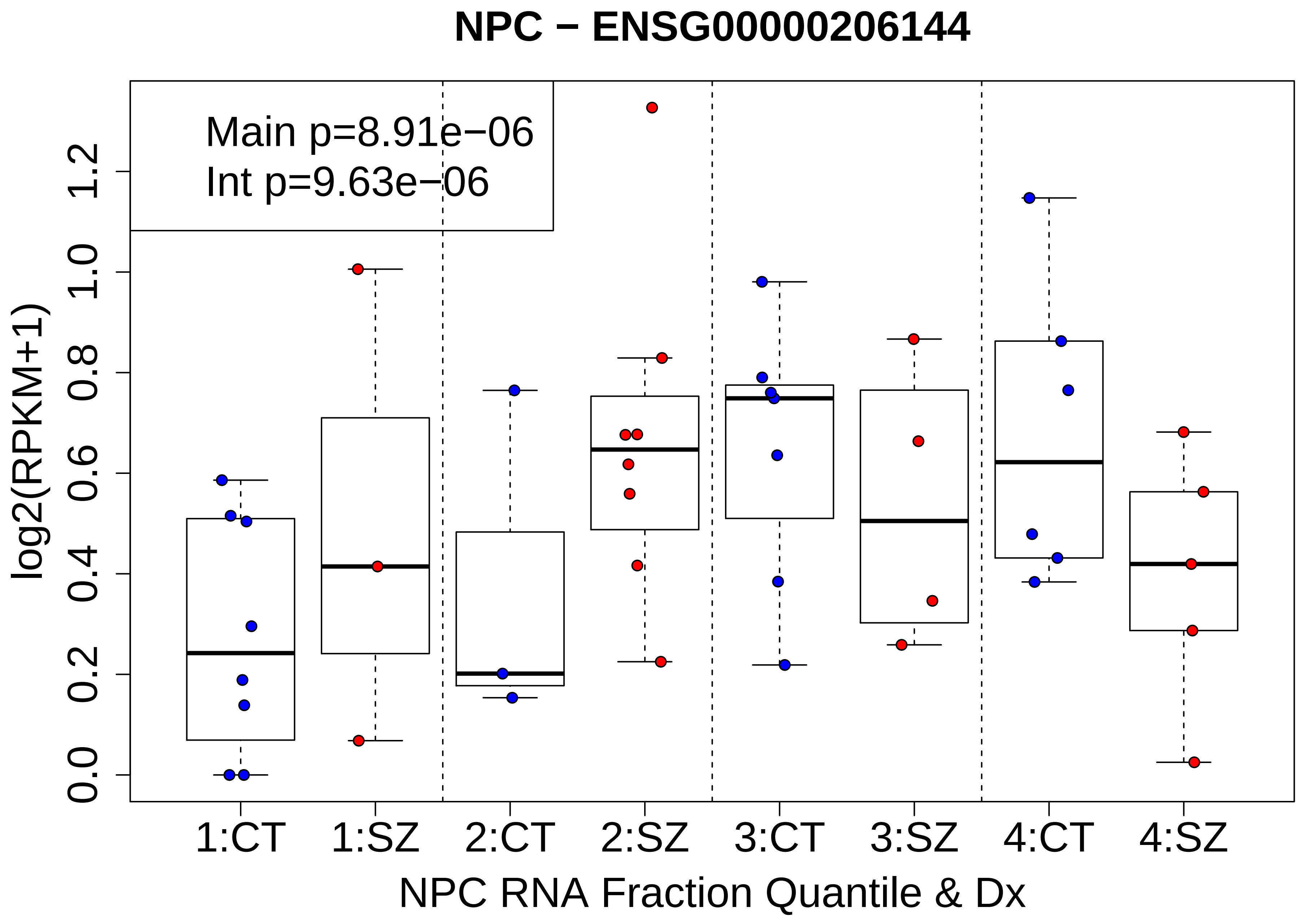
NPC − ENSG00000206144
Main p=8.91e−06
Int p=9.63e−06
log2(RPKM+1)
0.0
0.2
0.4
0.6
0.8
1.0
1.2
1:CT
1:SZ
2:CT
2:SZ
3:CT
3:SZ
4:CT
4:SZ
NPC RNA Fraction Quantile & Dx

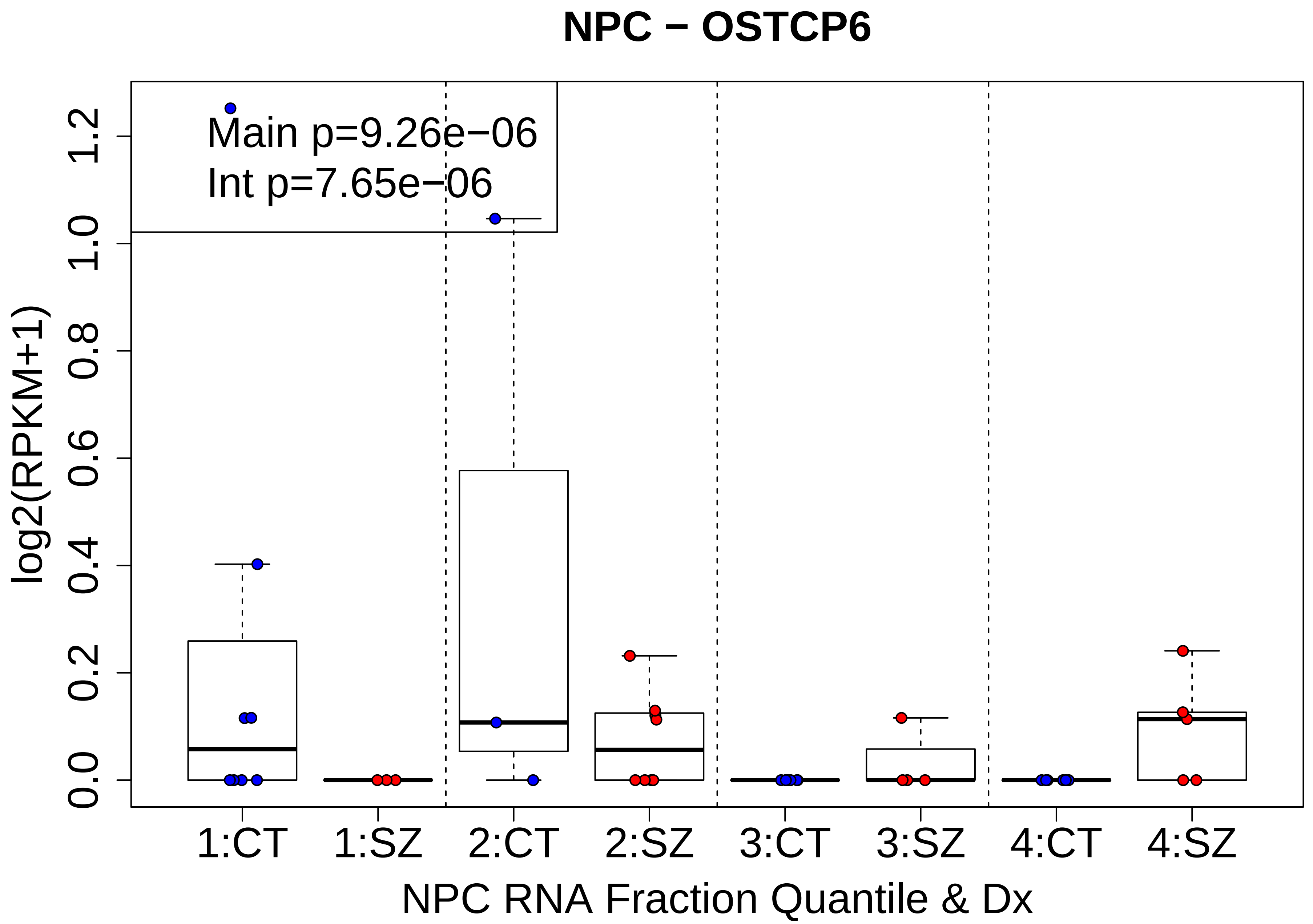
NPC − OSTCP6
Main p=9.26e−06
Int p=7.65e−06
log2(RPKM+1)
0.0
0.2
0.4
0.6
0.8
1.0
1.2
1:CT
1:SZ
2:CT
2:SZ
3:CT
3:SZ
4:CT
4:SZ
NPC RNA Fraction Quantile & Dx

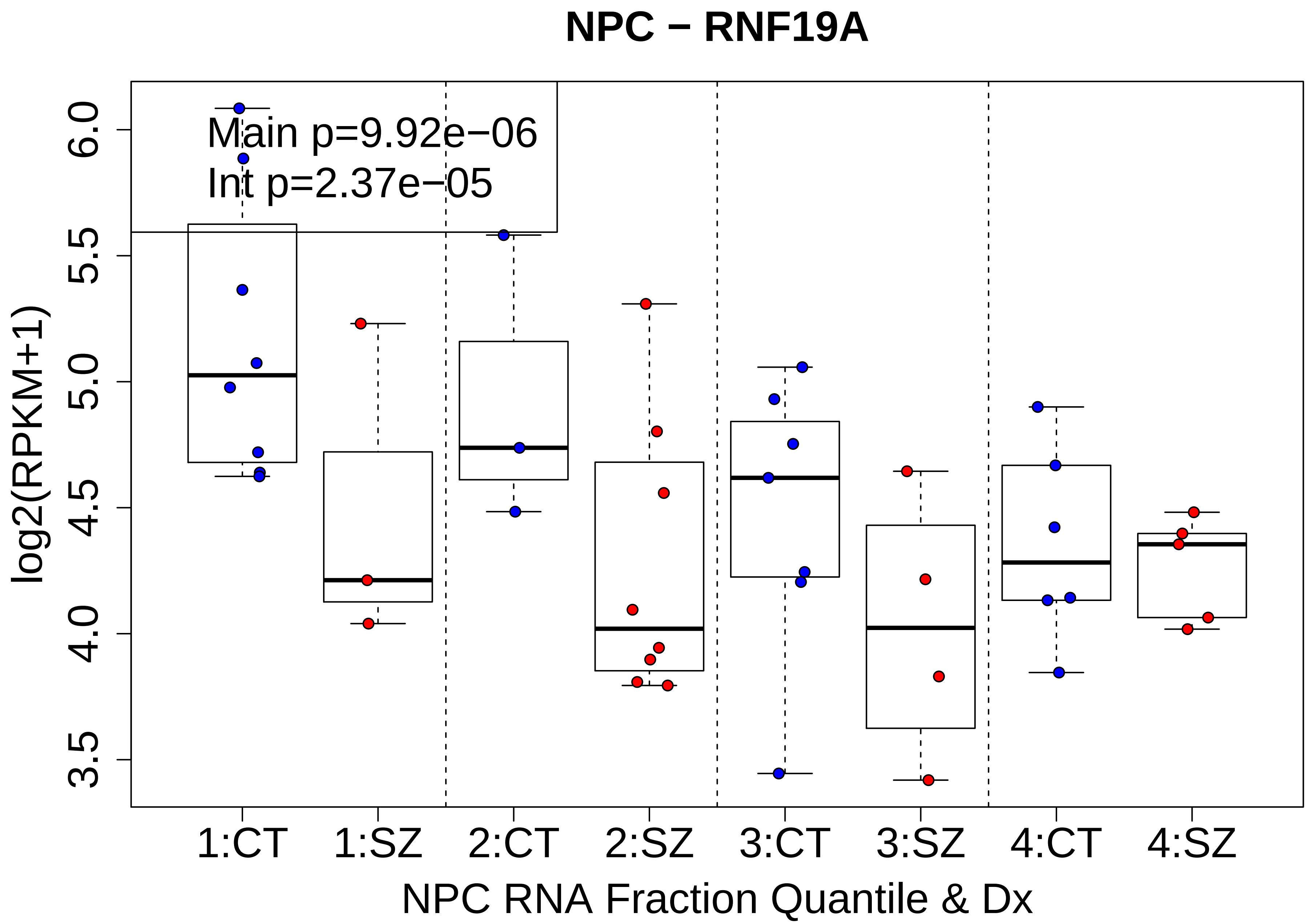
NPC − RNF19A
Main p=9.92e−06
Int p=2.37e−05
log2(RPKM+1)
3.5
4.0
4.5
5.0
5.5
6.0
1:CT
1:SZ
2:CT
2:SZ
3:CT
3:SZ
4:CT
4:SZ
NPC RNA Fraction Quantile & Dx

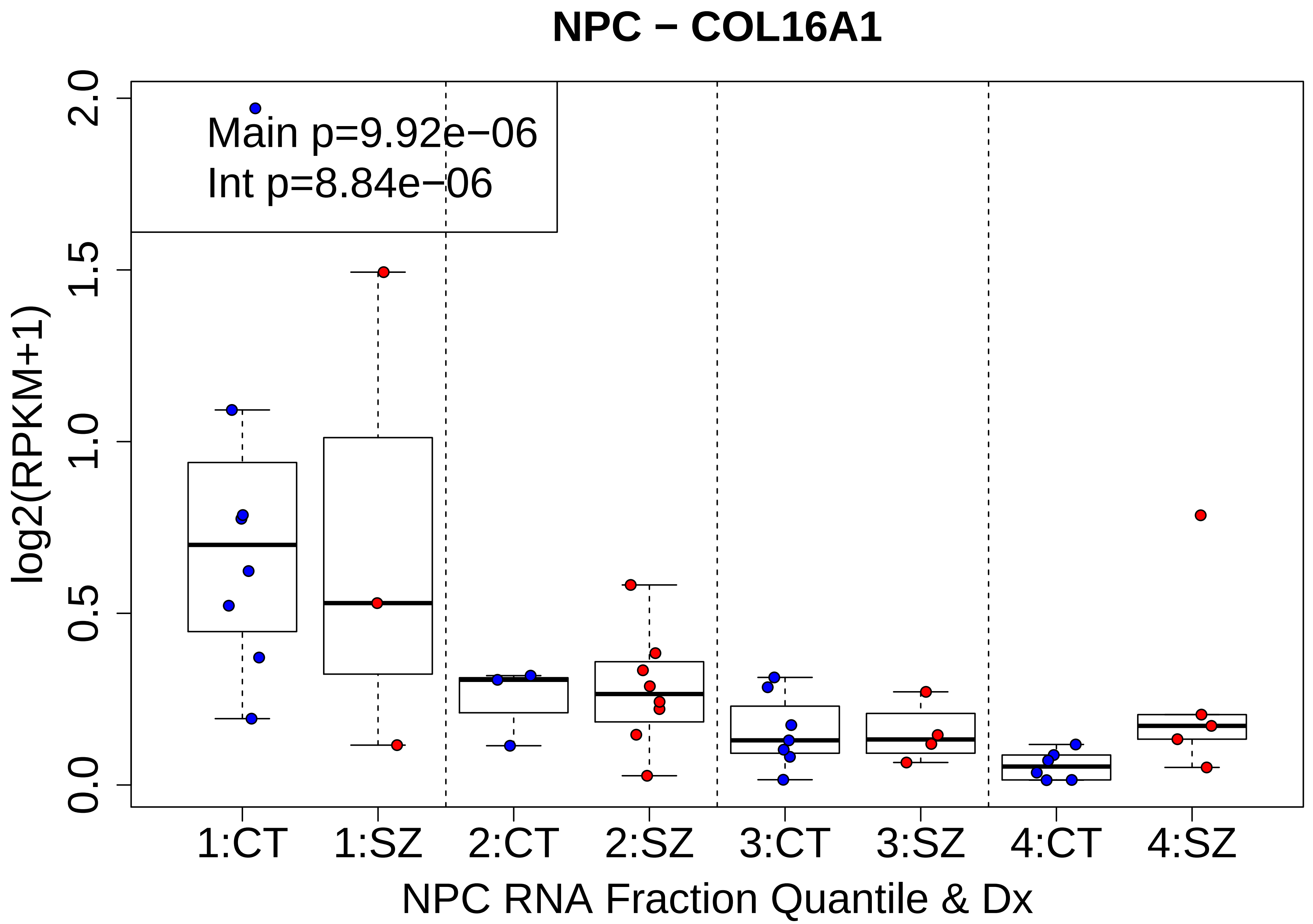

NPC − COL16A1
Main p=9.92e−06
Int p=8.84e−06
log2(RPKM+1)
0.0
0.5
1.0
1.5
2.0
1:CT
1:SZ
2:CT
2:SZ
3:CT
3:SZ
4:CT
4:SZ
NPC RNA Fraction Quantile & Dx

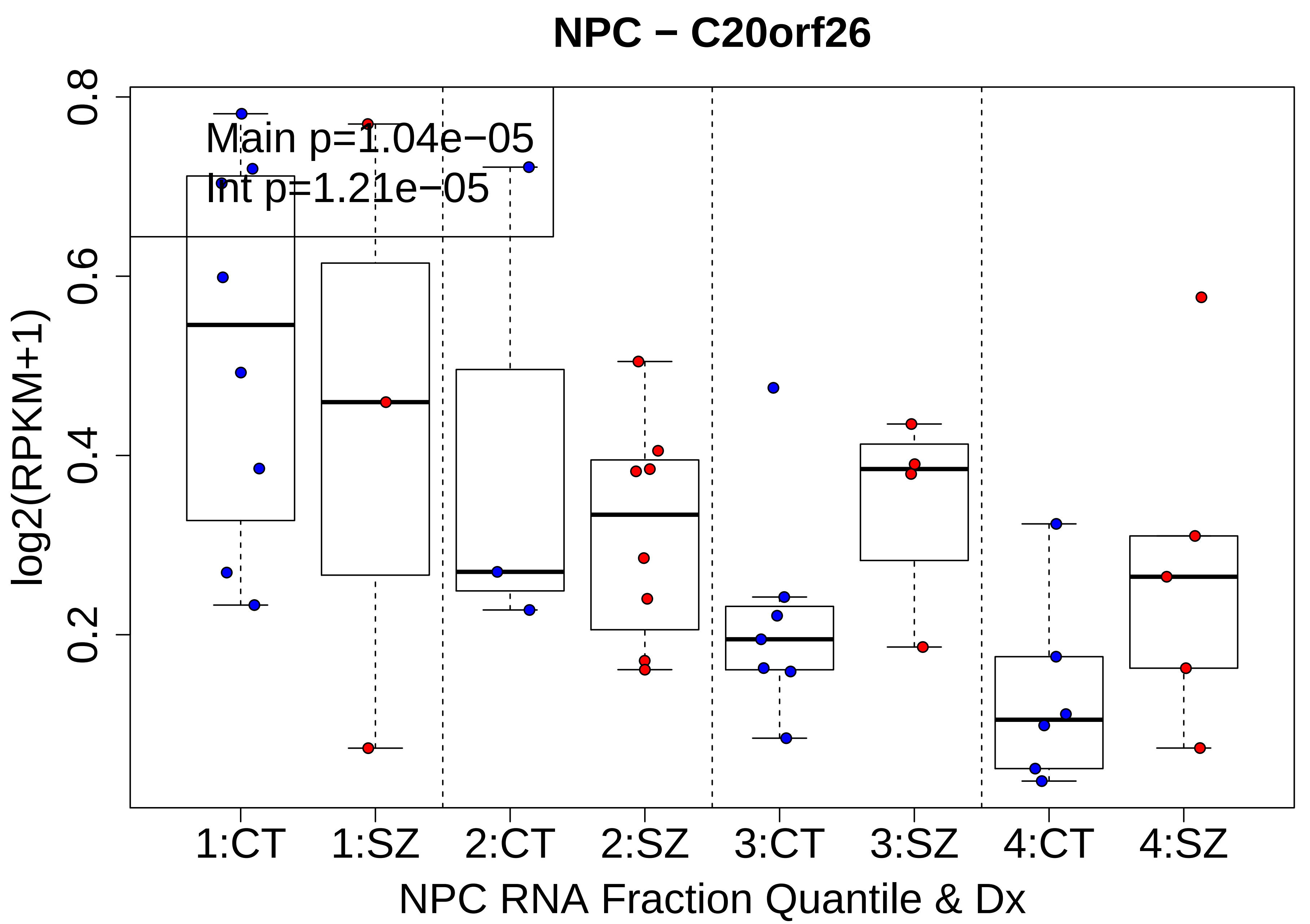

NPC − C20orf26
Main p=1.04e−05
Int p=1.21e−05
log2(RPKM+1)
0.2
0.4
0.6
0.8
1:CT
1:SZ
2:CT
2:SZ
3:CT
3:SZ
4:CT
4:SZ
NPC RNA Fraction Quantile & Dx

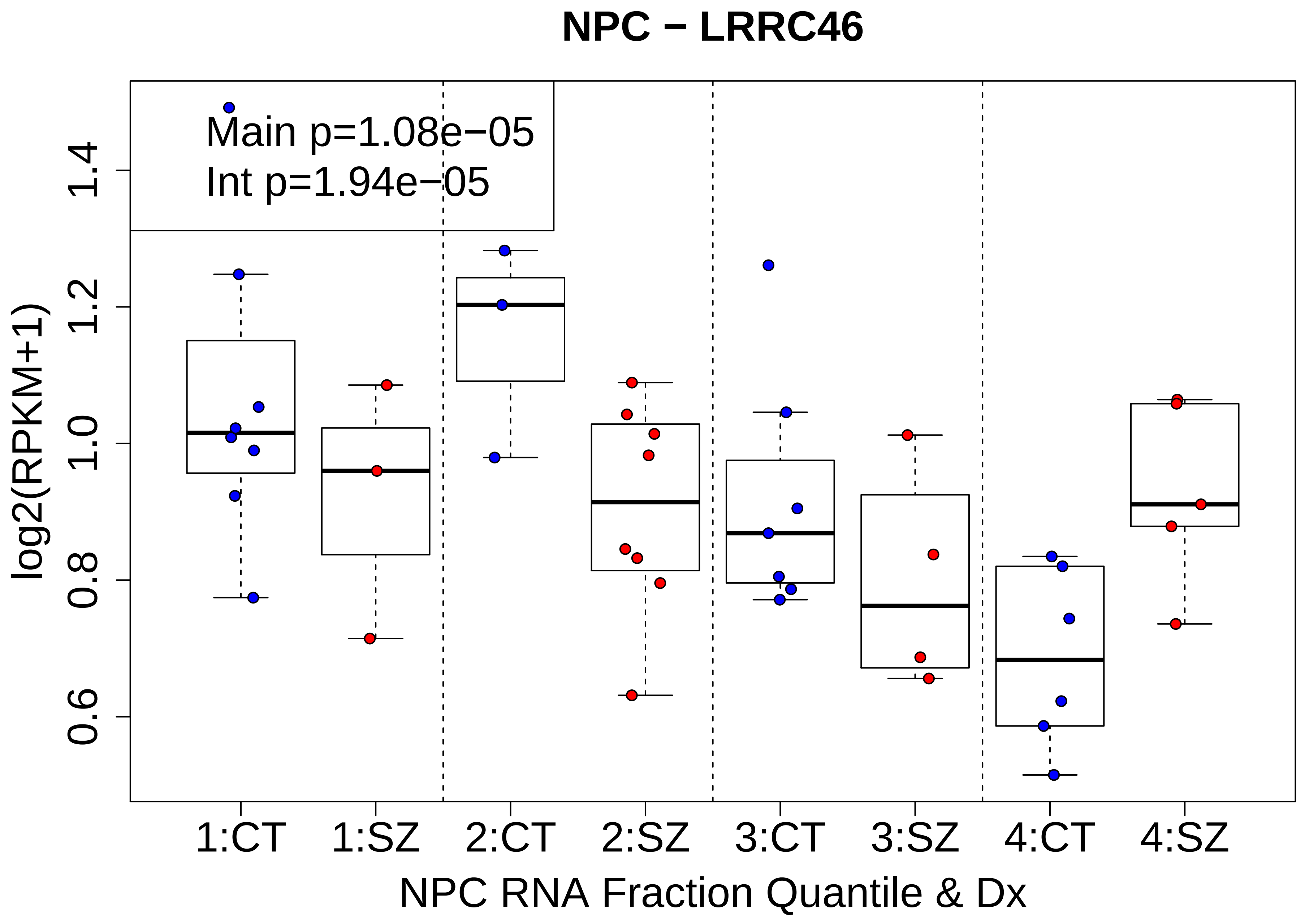

NPC − LRRC46
Main p=1.08e−05
Int p=1.94e−05
log2(RPKM+1)
0.6
0.8
1.0
1.2
1.4
1:CT
1:SZ
2:CT
2:SZ
3:CT
3:SZ
4:CT
4:SZ
NPC RNA Fraction Quantile & Dx

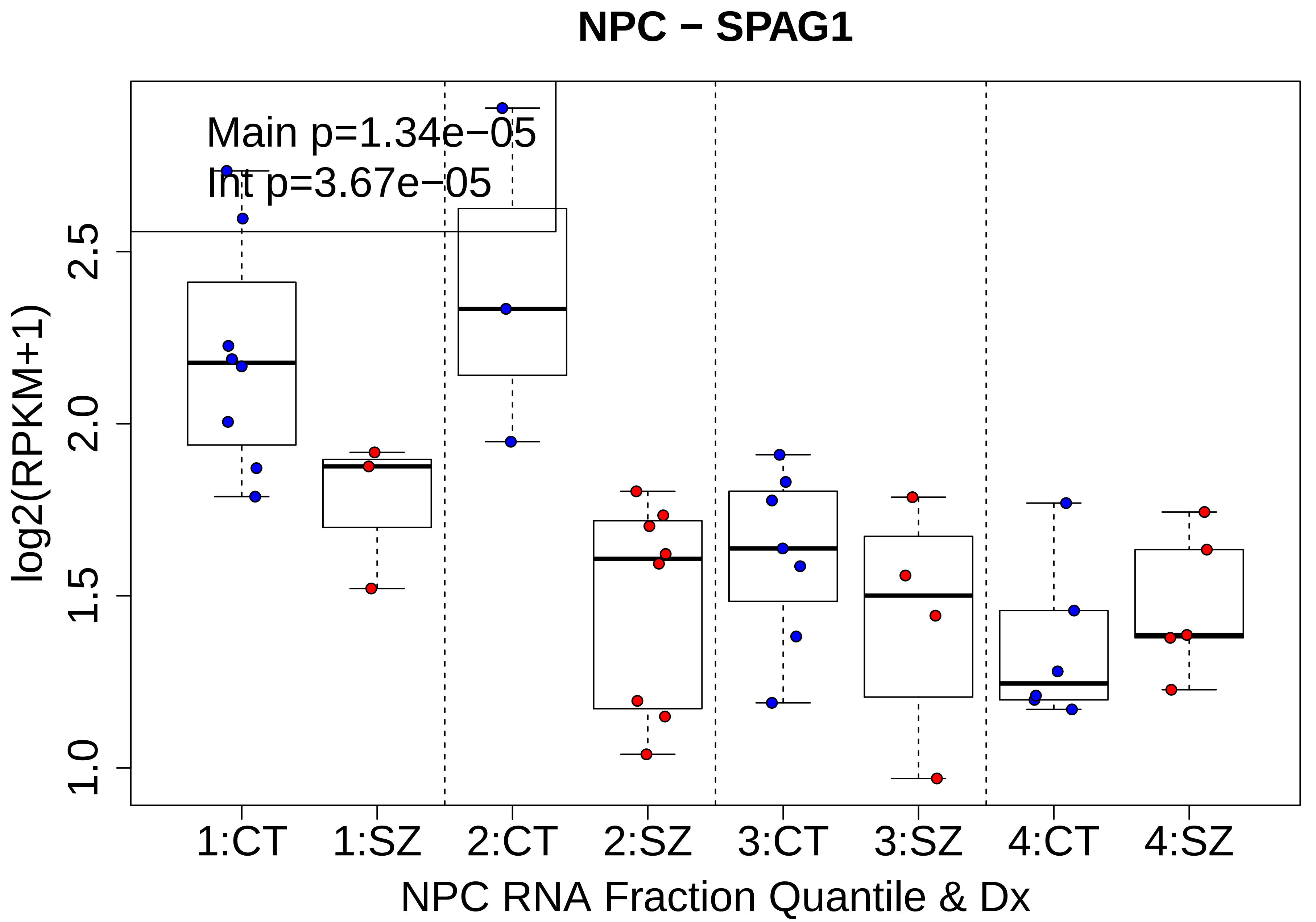

NPC − SPAG1
Main p=1.34e−05
Int p=3.67e−05
log2(RPKM+1)
1.0
1.5
2.0
2.5
1:CT
1:SZ
2:CT
2:SZ
3:CT
3:SZ
4:CT
4:SZ
NPC RNA Fraction Quantile & Dx

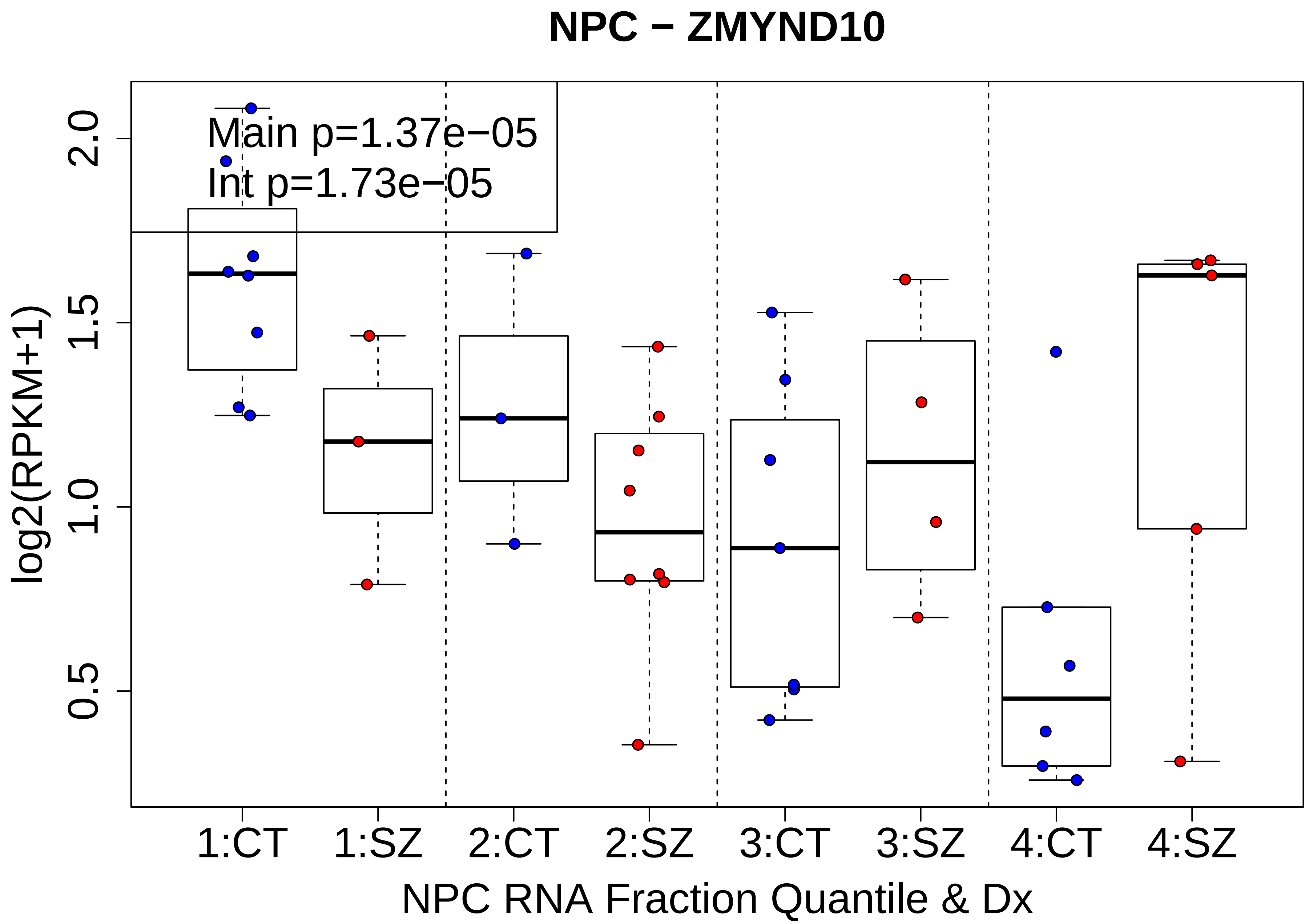
NPC − ZMYND10
Main p=1.37e−05
Int p=1.73e−05
log2(RPKM+1)
2.0
1.5
1.0
0.5
1:CT
1:SZ
2:CT
2:SZ
3:CT
3:SZ
4:CT
4:SZ
NPC RNA Fraction Quantile & Dx

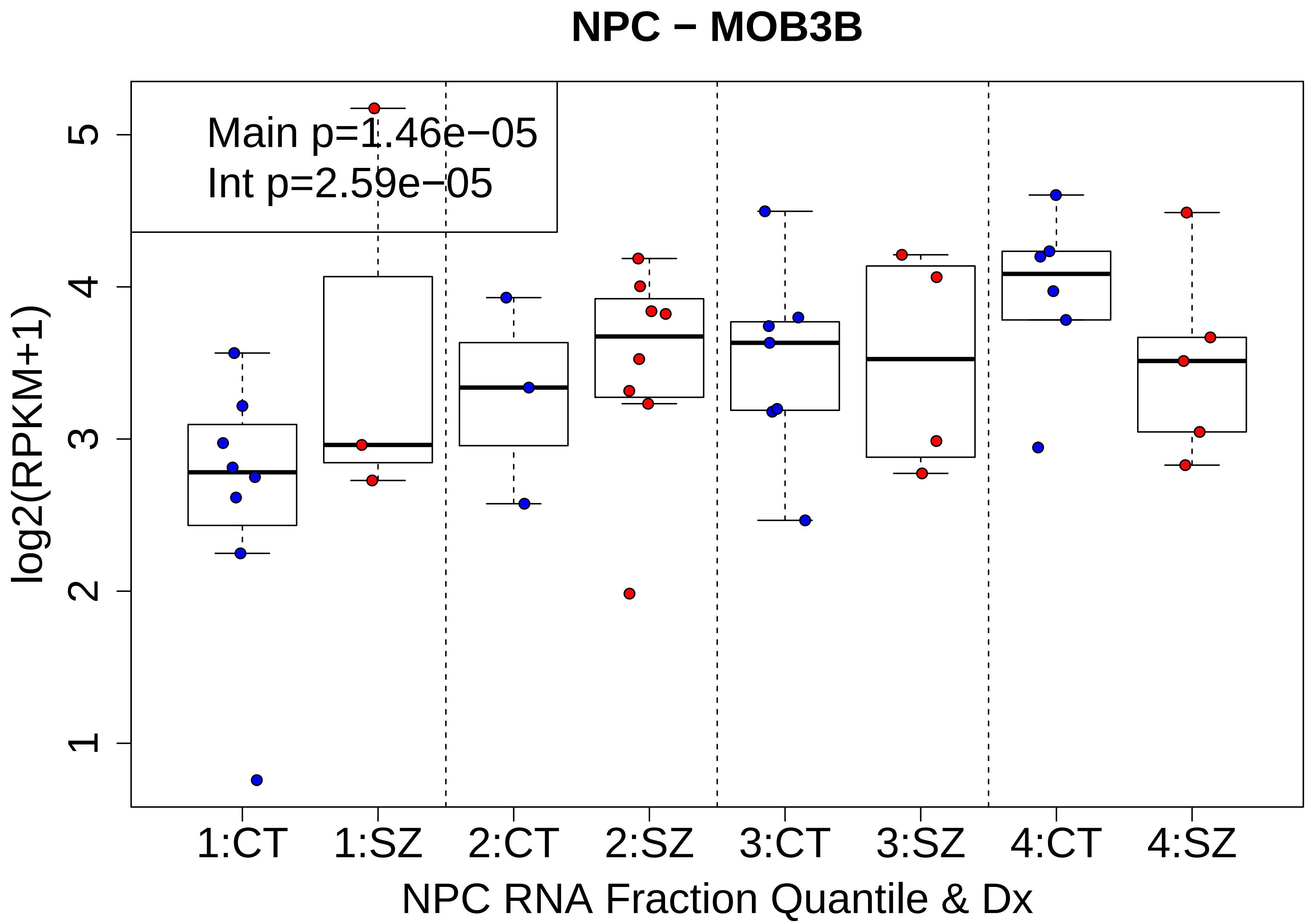

NPC − MOB3B
Main p=1.46e−05
Int p=2.59e−05
log2(RPKM+1)
1
2
3
4
5
1:CT
1:SZ
2:CT
2:SZ
3:CT
3:SZ
4:CT
4:SZ
NPC RNA Fraction Quantile & Dx

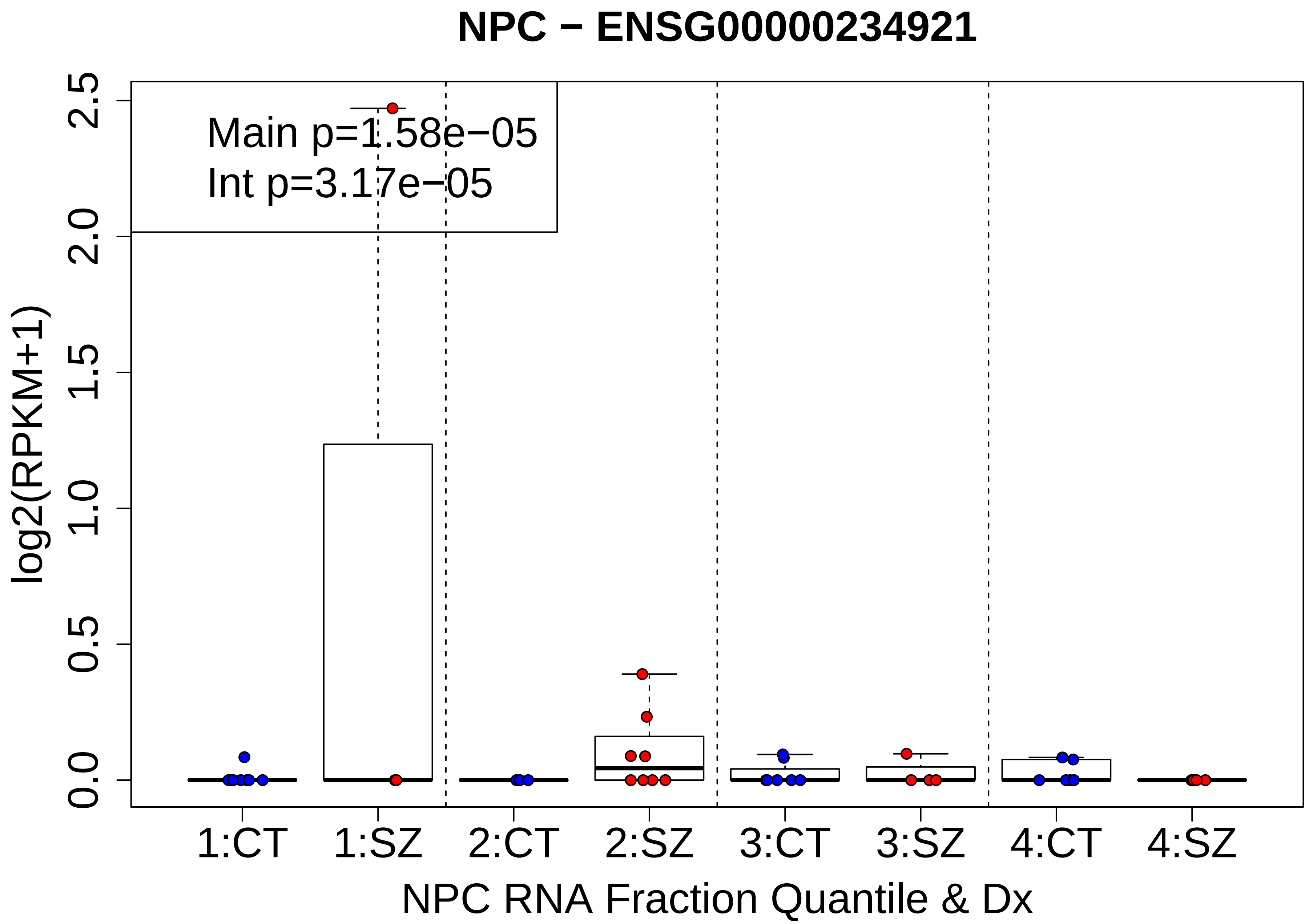

NPC − ENSG00000234921
Main p=1.58e−05
Int p=3.17e−05
log2(RPKM+1)
0.0
0.5
1.0
1.5
2.0
2.5
1:CT
1:SZ
2:CT
2:SZ
3:CT
3:SZ
4:CT
4:SZ
NPC RNA Fraction Quantile & Dx

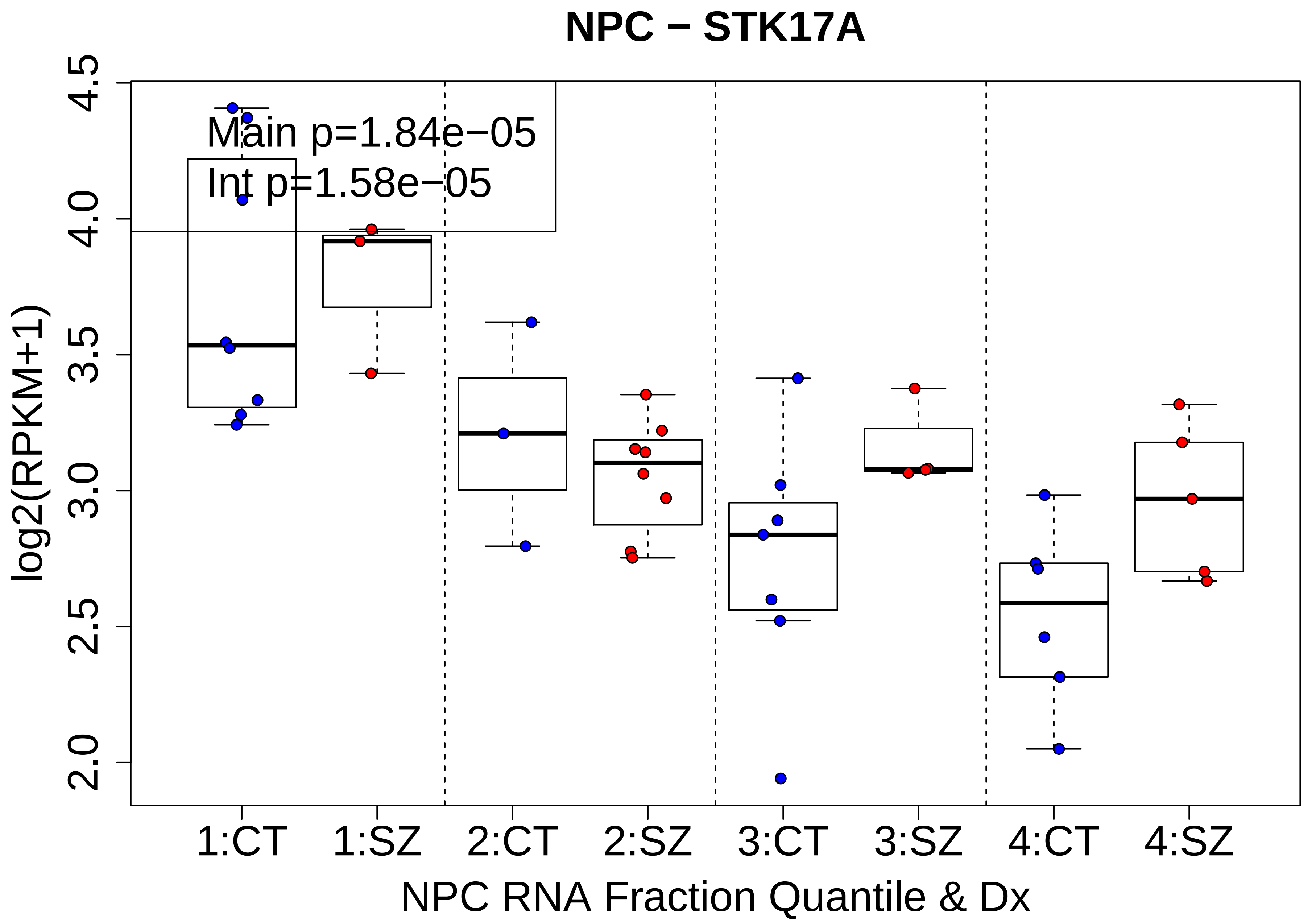

NPC − STK17A
Main p=1.84e−05
Int p=1.58e−05
log2(RPKM+1)
2.0
2.5
3.0
3.5
4.0
4.5
1:CT
1:SZ
2:CT
2:SZ
3:CT
3:SZ
4:CT
4:SZ
NPC RNA Fraction Quantile & Dx

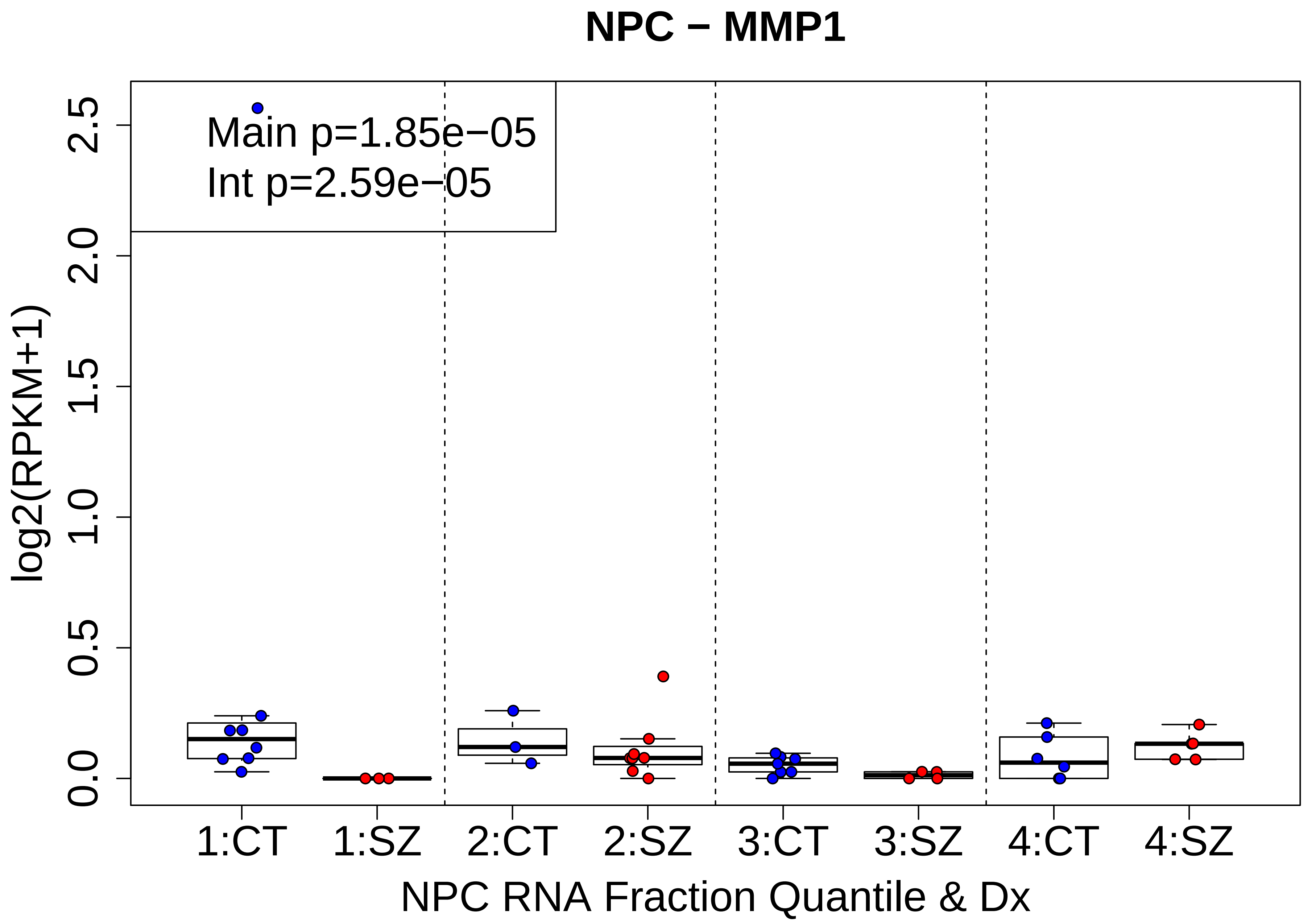

NPC − MMP1
Main p=1.85e−05
Int p=2.59e−05
log2(RPKM+1)
0.0
0.5
1.0
1.5
2.0
2.5
1:CT
1:SZ
2:CT
2:SZ
3:CT
3:SZ
4:CT
4:SZ
NPC RNA Fraction Quantile & Dx

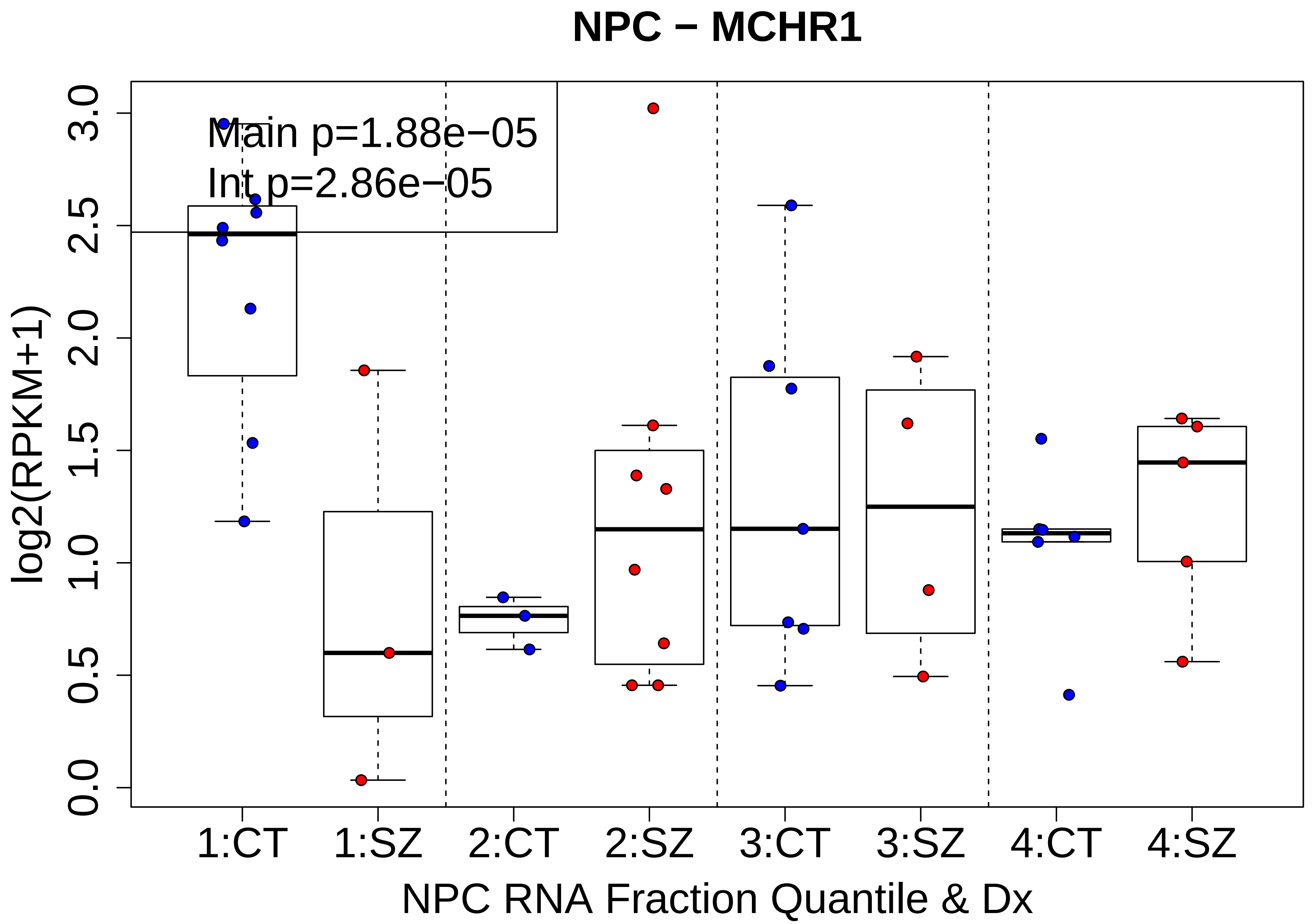

NPC − MCHR1
Main p=1.88e−05
Int p=2.86e−05
log2(RPKM+1)
0.0
0.5
1.0
1.5
2.0
2.5
3.0
1:CT
1:SZ
2:CT
2:SZ
3:CT
3:SZ
4:CT
4:SZ
NPC RNA Fraction Quantile & Dx

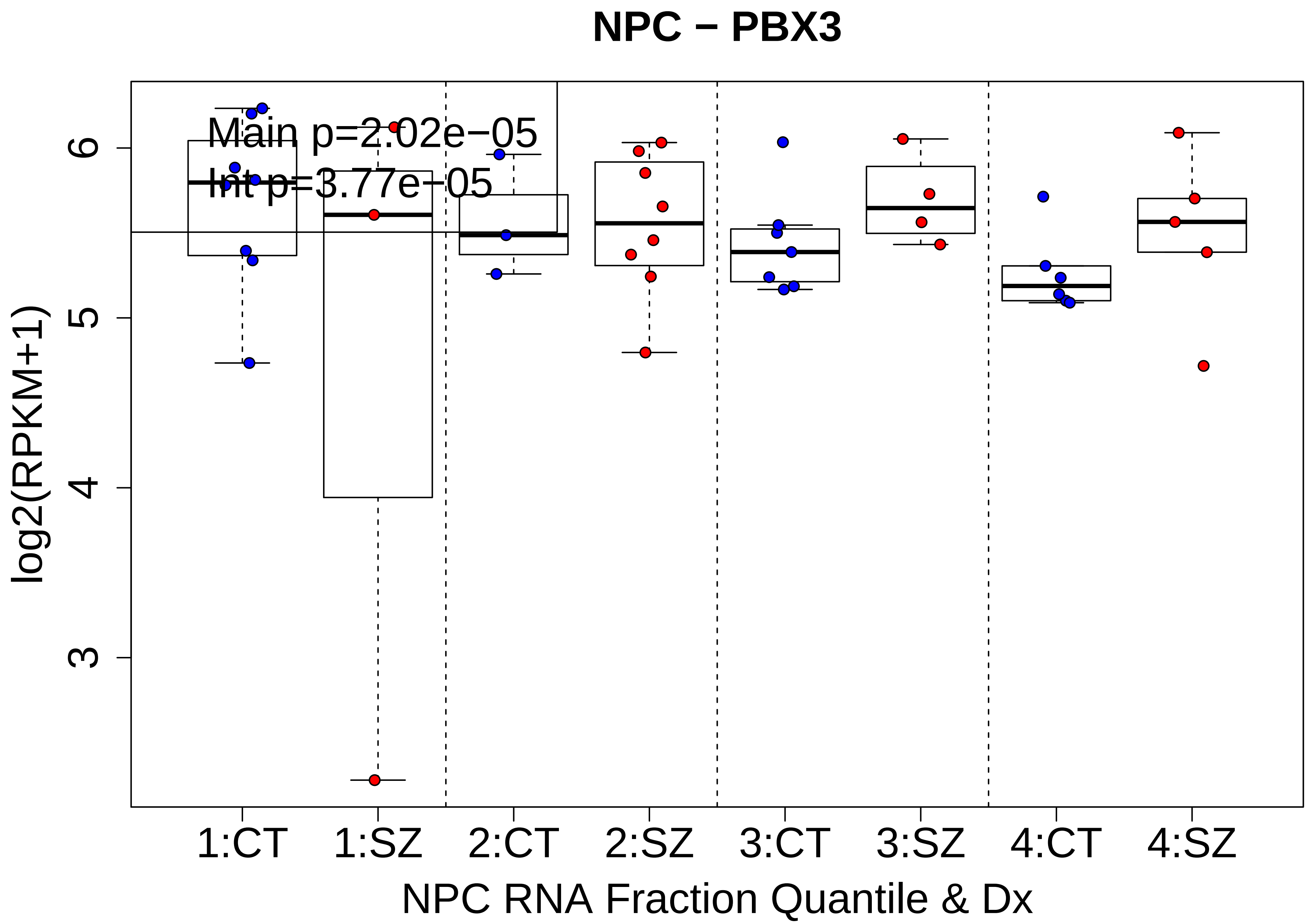

NPC − PBX3
Main p=2.02e−05
Int p=3.77e−05
log2(RPKM+1)
3
4
5
6
1:CT
1:SZ
2:CT
2:SZ
3:CT
3:SZ
4:CT
4:SZ
NPC RNA Fraction Quantile & Dx

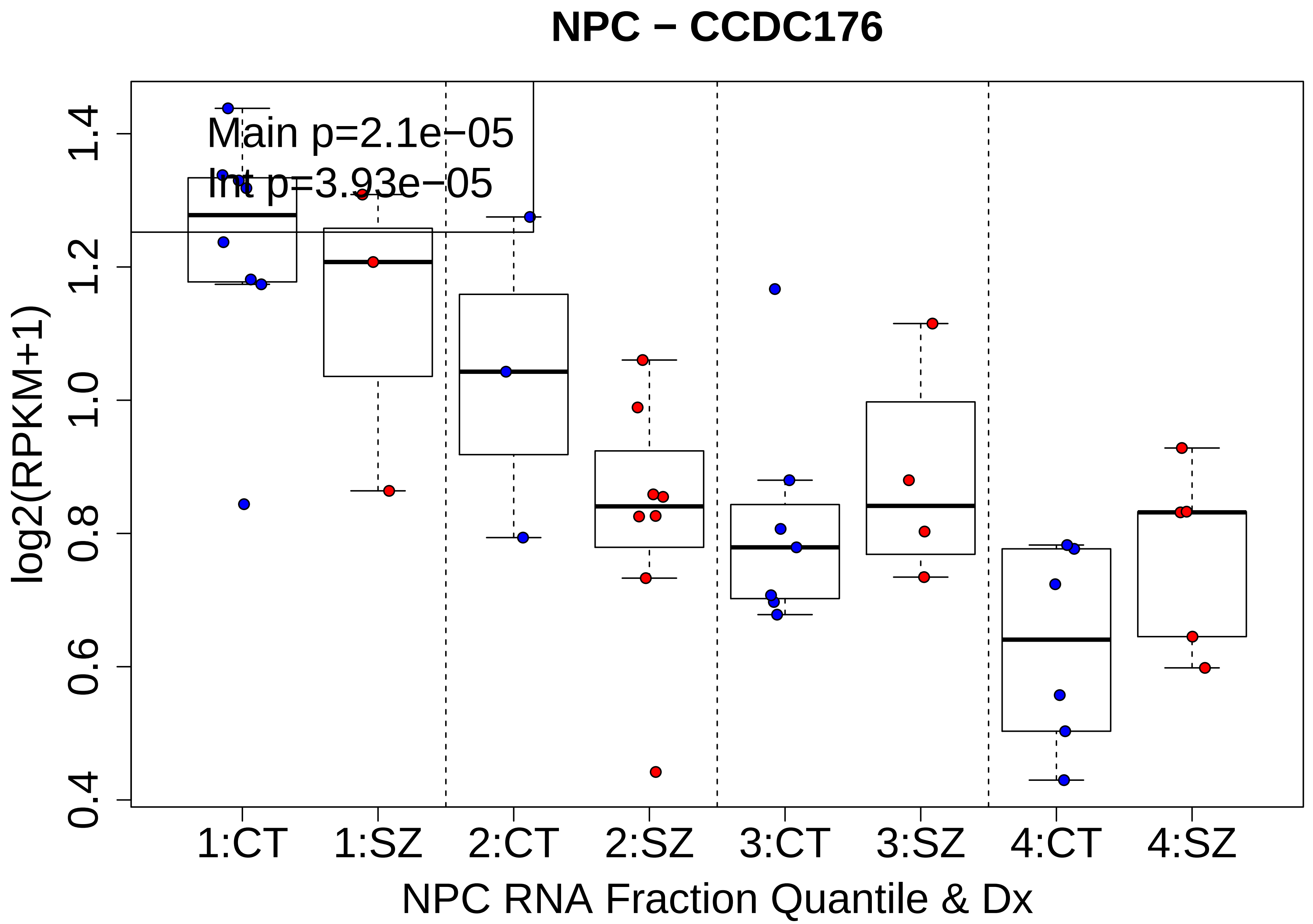

NPC − CCDC176
Main p=2.1e−05
Int p=3.93e−05
log2(RPKM+1)
0.4
0.6
0.8
1.0
1.2
1.4
1:CT
1:SZ
2:CT
2:SZ
3:CT
3:SZ
4:CT
4:SZ
NPC RNA Fraction Quantile & Dx

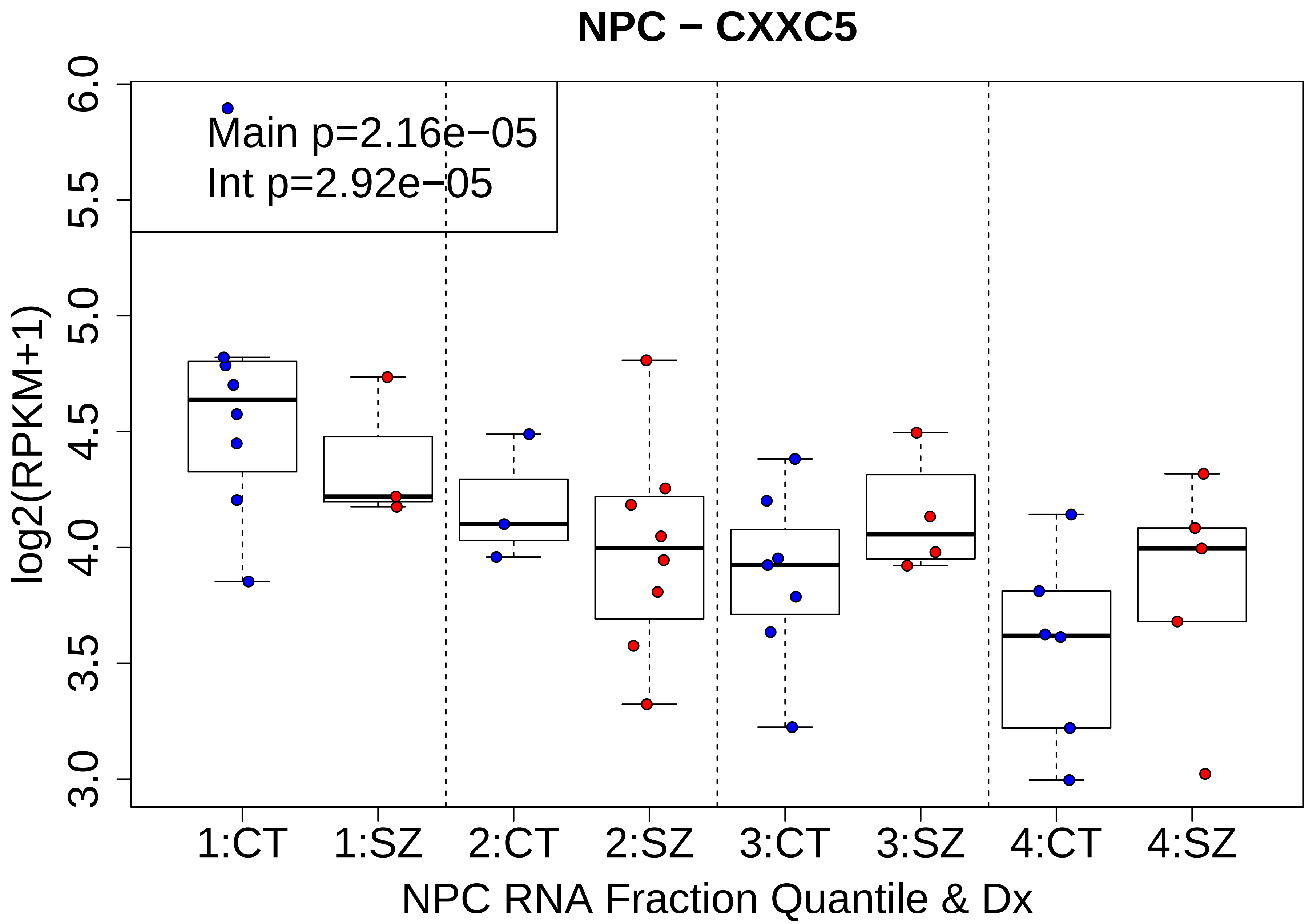
NPC − CXXC5
Main p=2.16e−05
Int p=2.92e−05
log2(RPKM+1)
3.0
3.5
4.0
4.5
5.0
5.5
6.0
1:CT
1:SZ
2:CT
2:SZ
3:CT
3:SZ
4:CT
4:SZ
NPC RNA Fraction Quantile & Dx

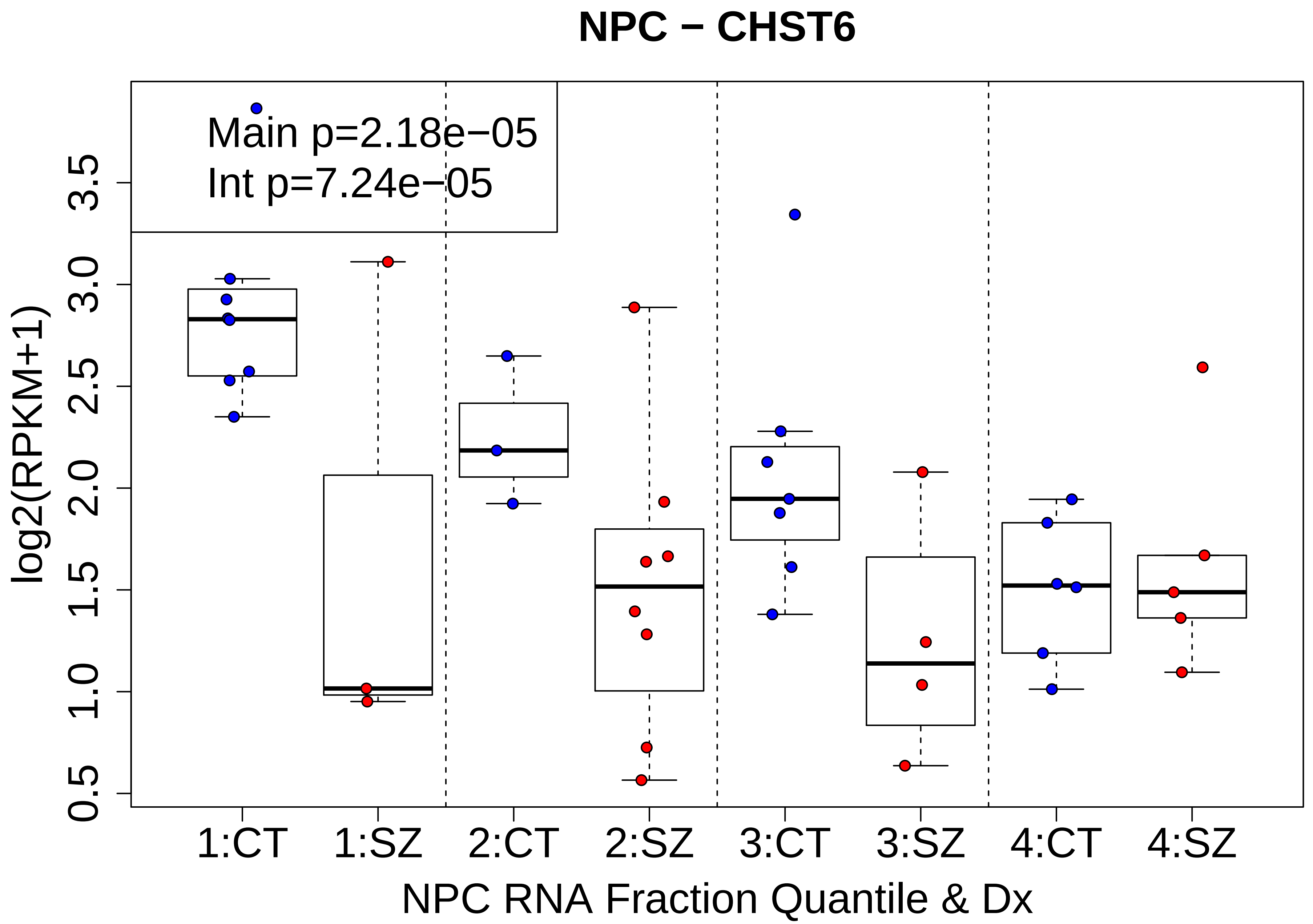
NPC − CHST6
Main p=2.18e−05
Int p=7.24e−05
log2(RPKM+1)
0.5
1.0
1.5
2.0
2.5
3.0
3.5
1:CT
1:SZ
2:CT
2:SZ
3:CT
3:SZ
4:CT
4:SZ
NPC RNA Fraction Quantile & Dx

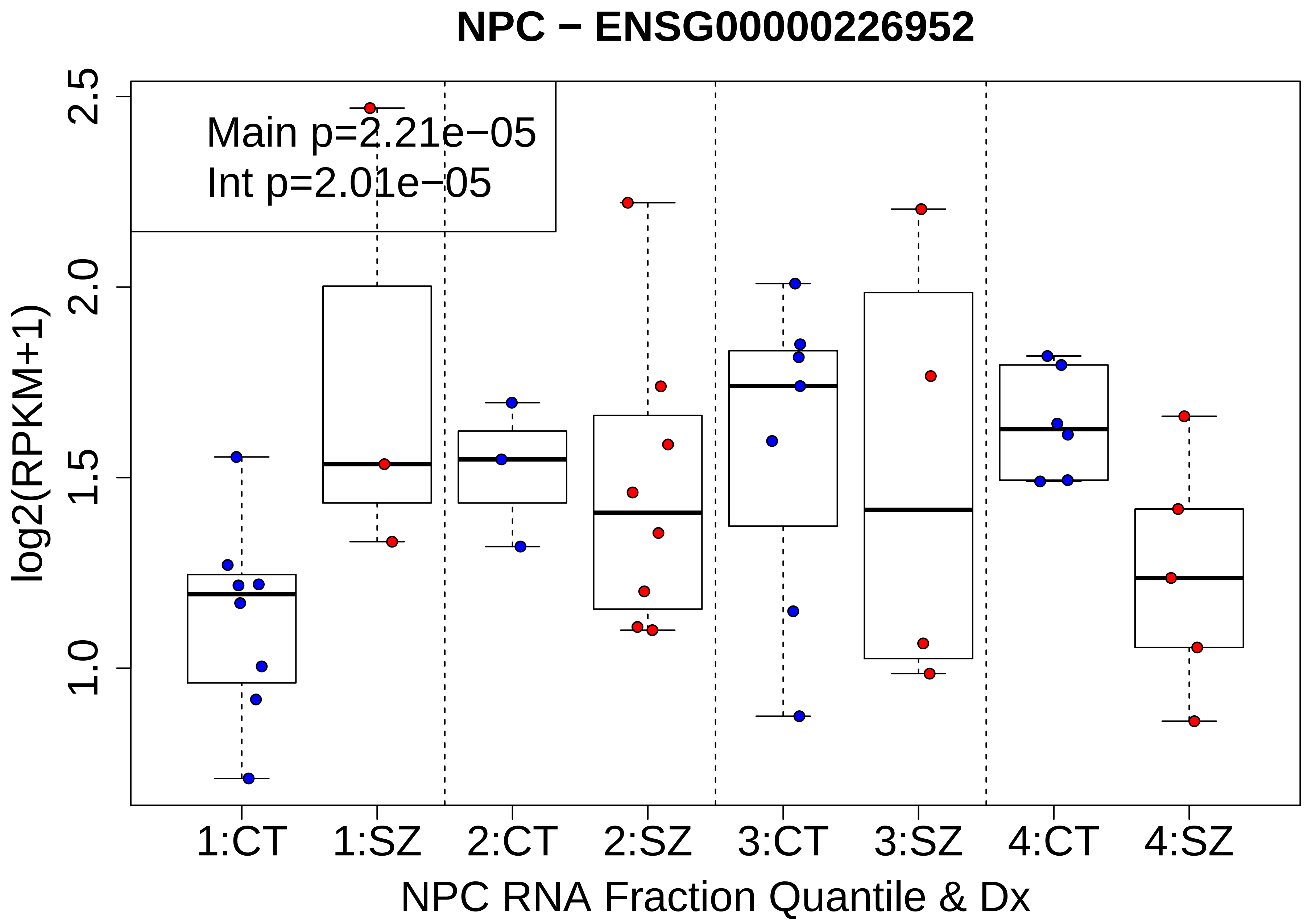

NPC − ENSG00000226952
Main p=2.21e−05
Int p=2.01e−05
log2(RPKM+1)
2.5
2.0
1.5
1.0
1:CT
1:SZ
2:CT
2:SZ
3:CT
3:SZ
4:CT
4:SZ
NPC RNA Fraction Quantile & Dx

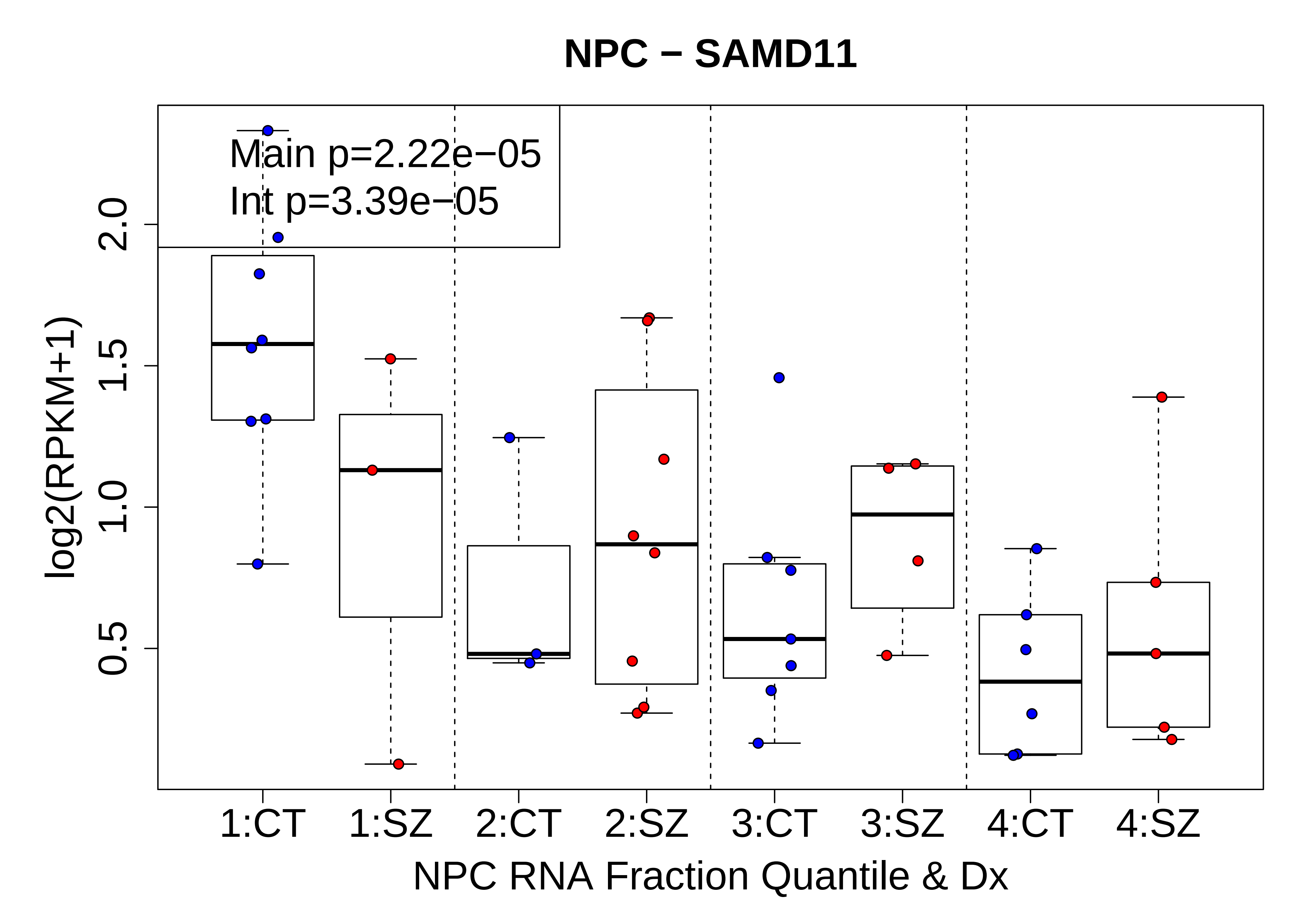

NPC − SAMD11
Main p=2.22e−05
Int p=3.39e−05
log2(RPKM+1)
2.0
1.5
1.0
0.5
1:CT
1:SZ
2:CT
2:SZ
3:CT
3:SZ
4:CT
4:SZ
NPC RNA Fraction Quantile & Dx

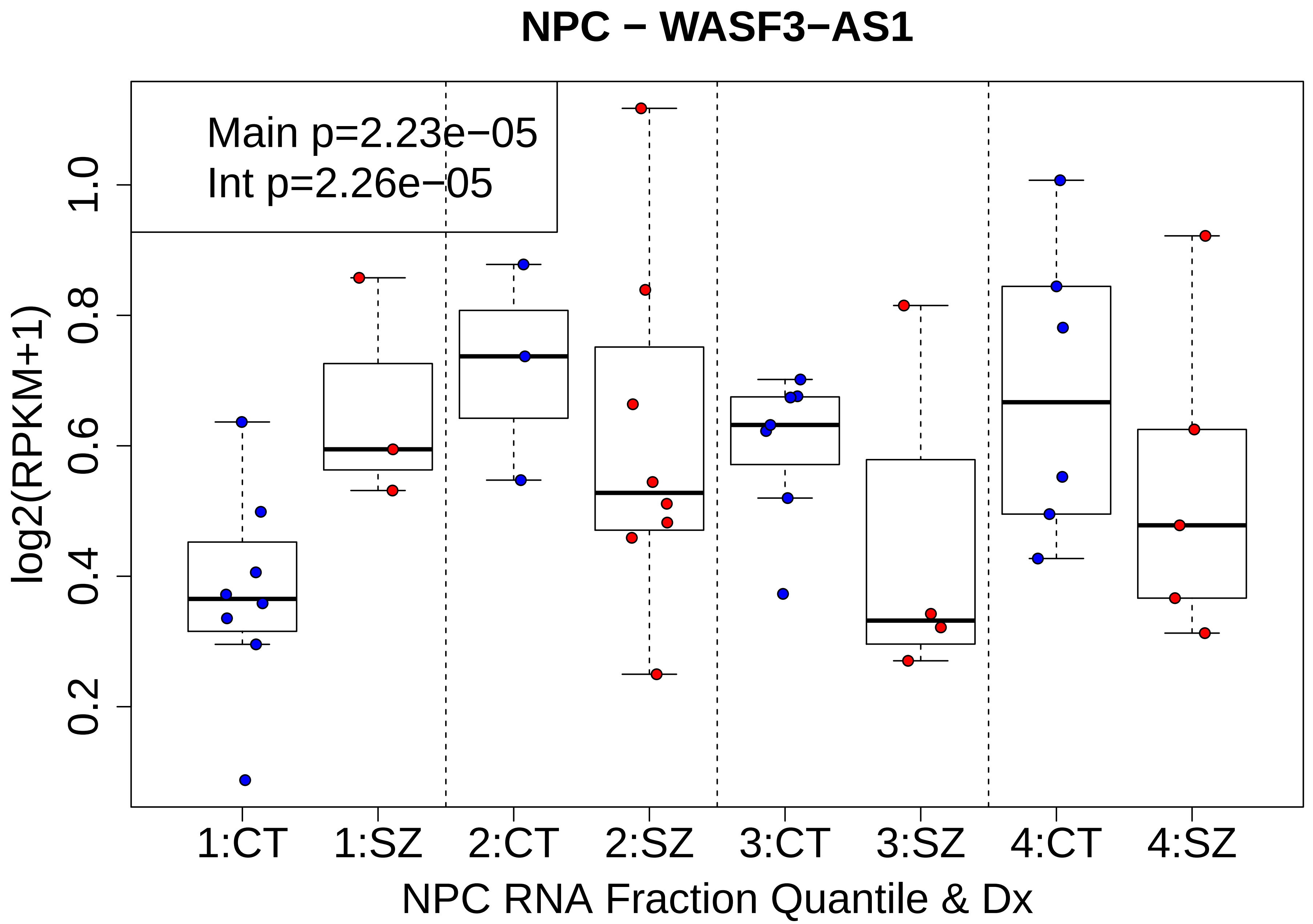

NPC − WASF3−AS1
Main p=2.23e−05
Int p=2.26e−05
log2(RPKM+1)
0.2
0.4
0.6
0.8
1.0
1:CT
1:SZ
2:CT
2:SZ
3:CT
3:SZ
4:CT
4:SZ
NPC RNA Fraction Quantile & Dx

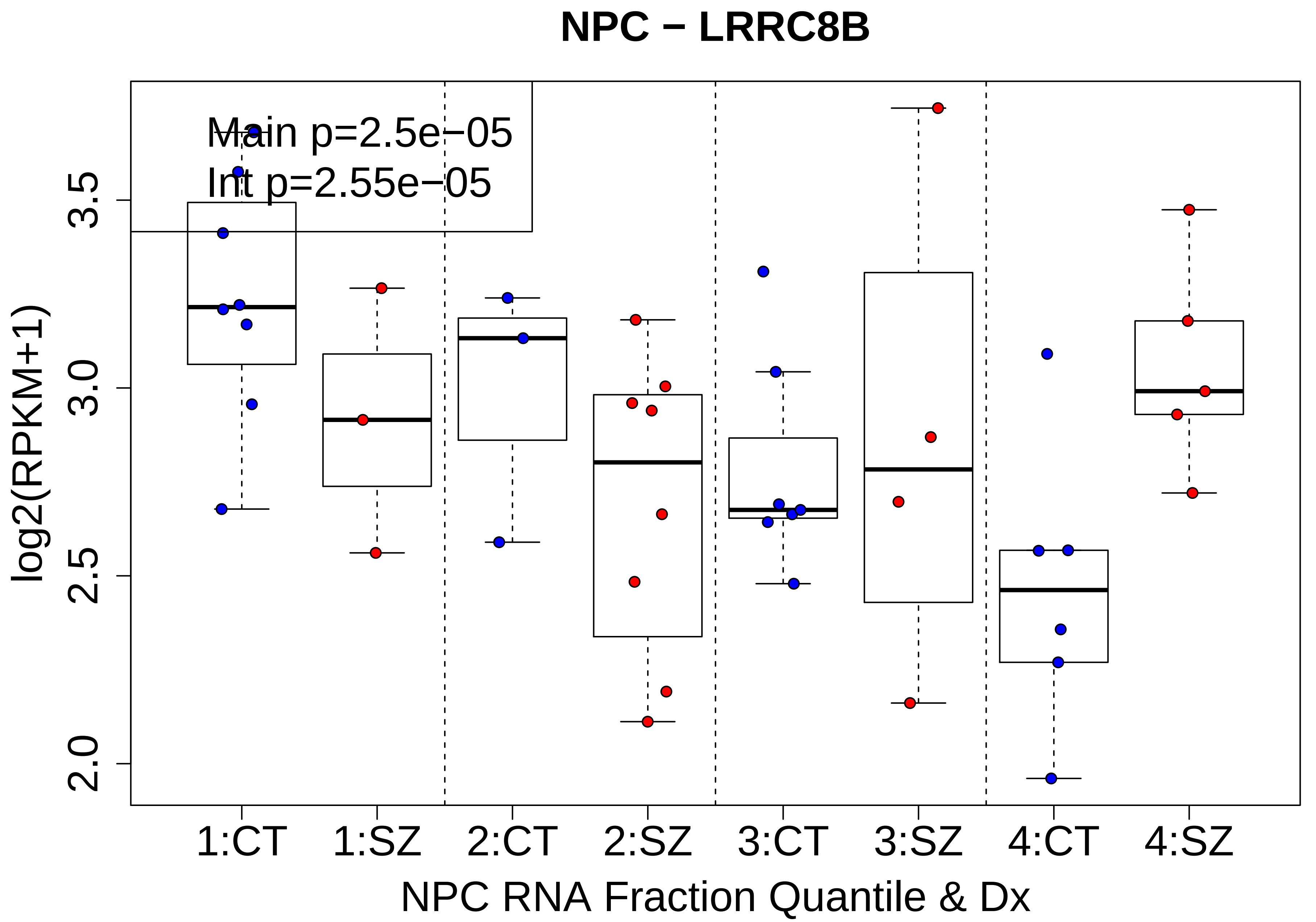

NPC − LRRC8B
Main p=2.5e−05
Int p=2.55e−05
log2(RPKM+1)
2.0
2.5
3.0
3.5
1:CT
1:SZ
2:CT
2:SZ
3:CT
3:SZ
4:CT
4:SZ
NPC RNA Fraction Quantile & Dx

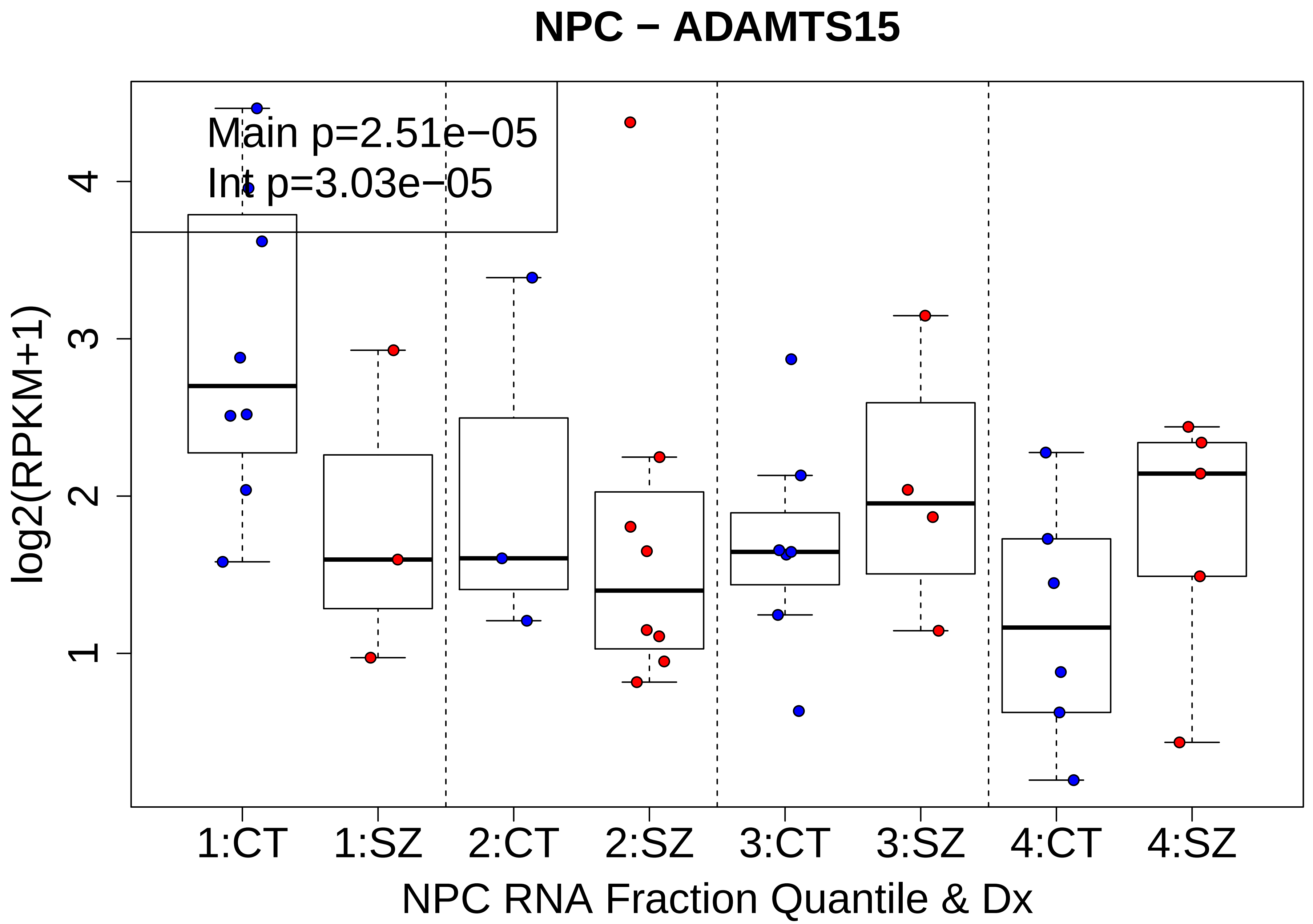

NPC − ADAMTS15
Main p=2.51e−05
Int p=3.03e−05
log2(RPKM+1)
4
3
2
1
1:CT
1:SZ
2:CT
2:SZ
3:CT
3:SZ
4:CT
4:SZ
NPC RNA Fraction Quantile & Dx

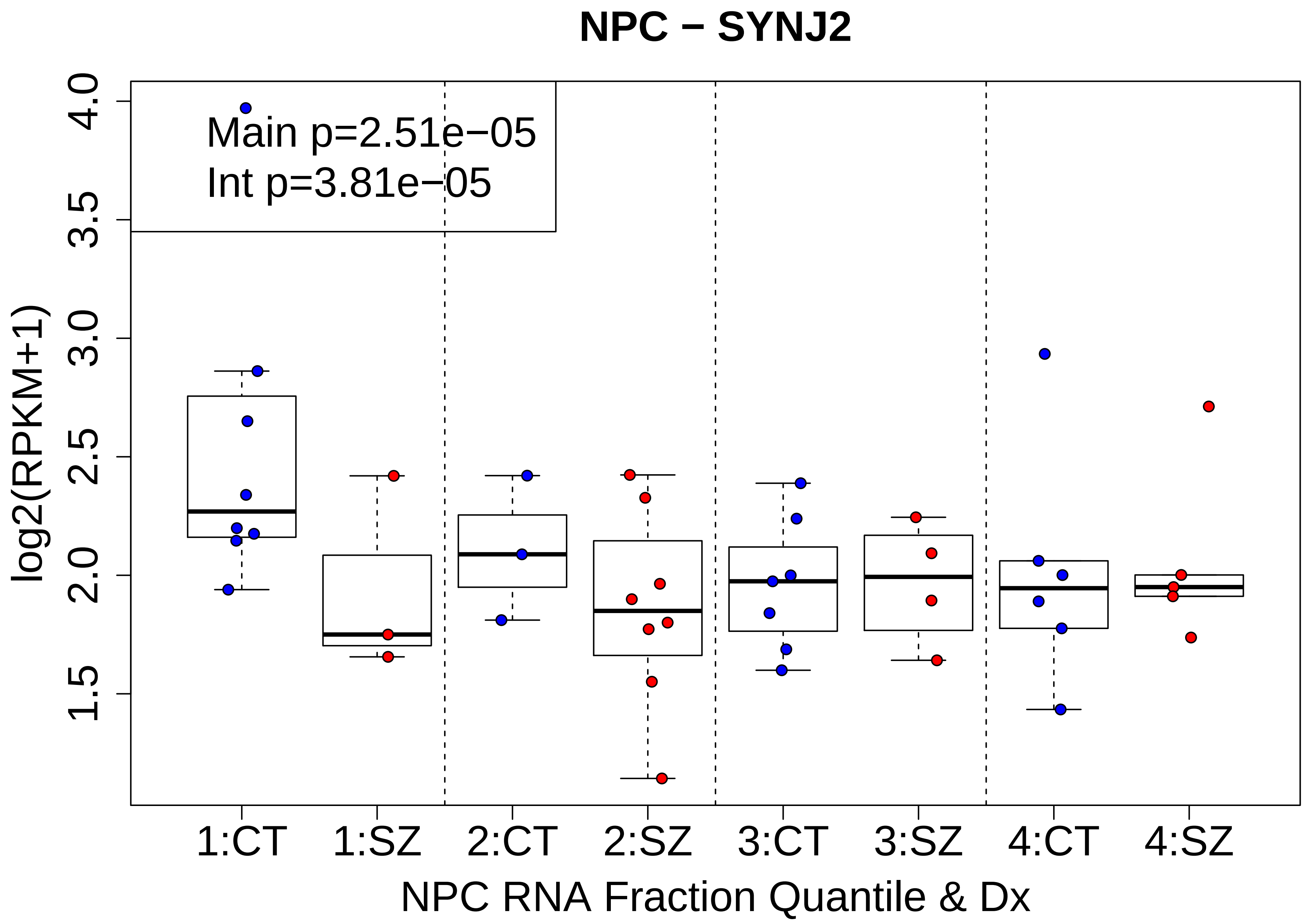
NPC − SYNJ2
Main p=2.51e−05
Int p=3.81e−05
log2(RPKM+1)
4.0
3.5
3.0
2.5
2.0
1.5
1:CT
1:SZ
2:CT
2:SZ
3:CT
3:SZ
4:CT
4:SZ
NPC RNA Fraction Quantile & Dx

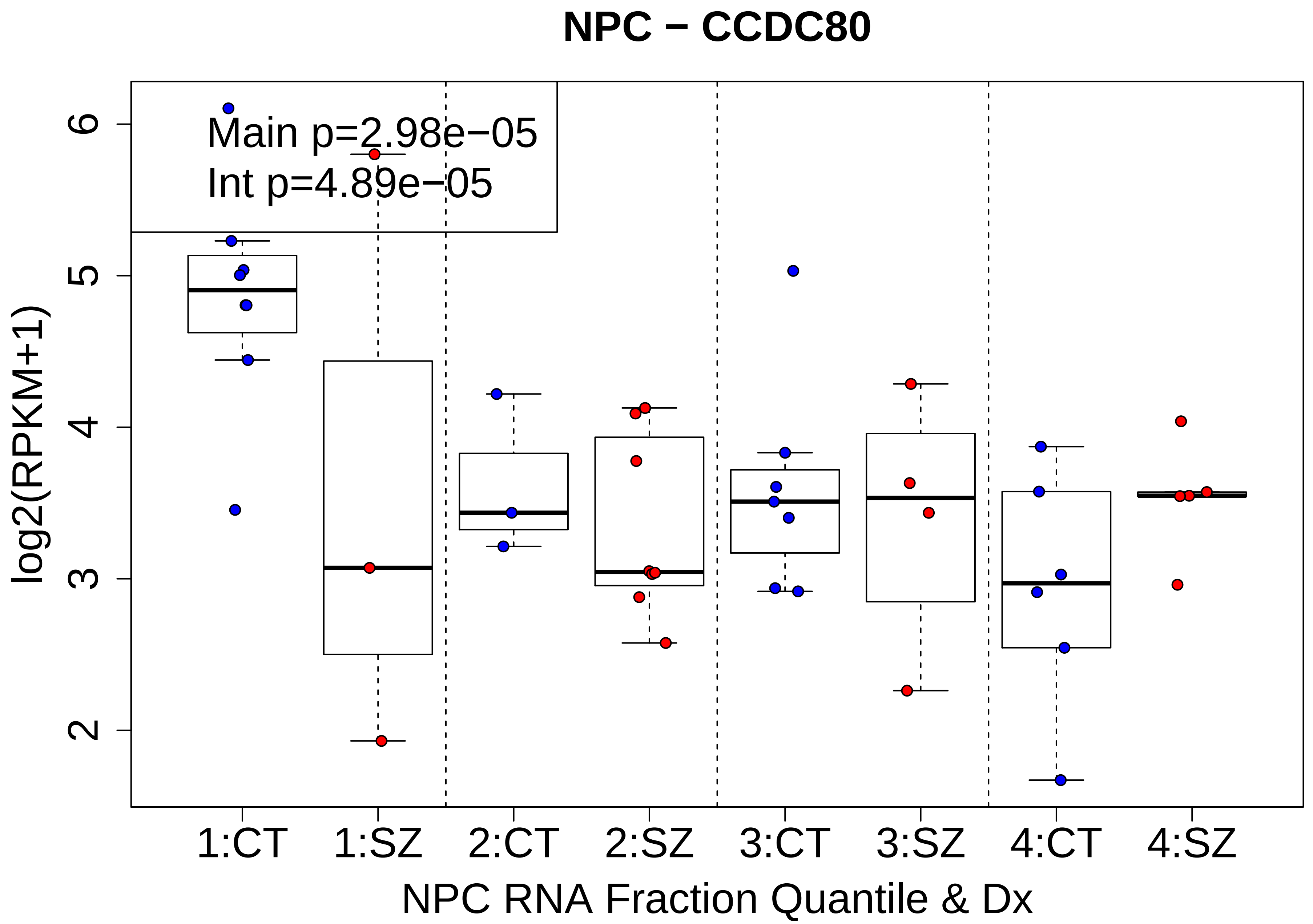
NPC − CCDC80
Main p=2.98e−05
Int p=4.89e−05
log2(RPKM+1)
6
5
4
3
2
1:CT
1:SZ
2:CT
2:SZ
3:CT
3:SZ
4:CT
4:SZ
NPC RNA Fraction Quantile & Dx

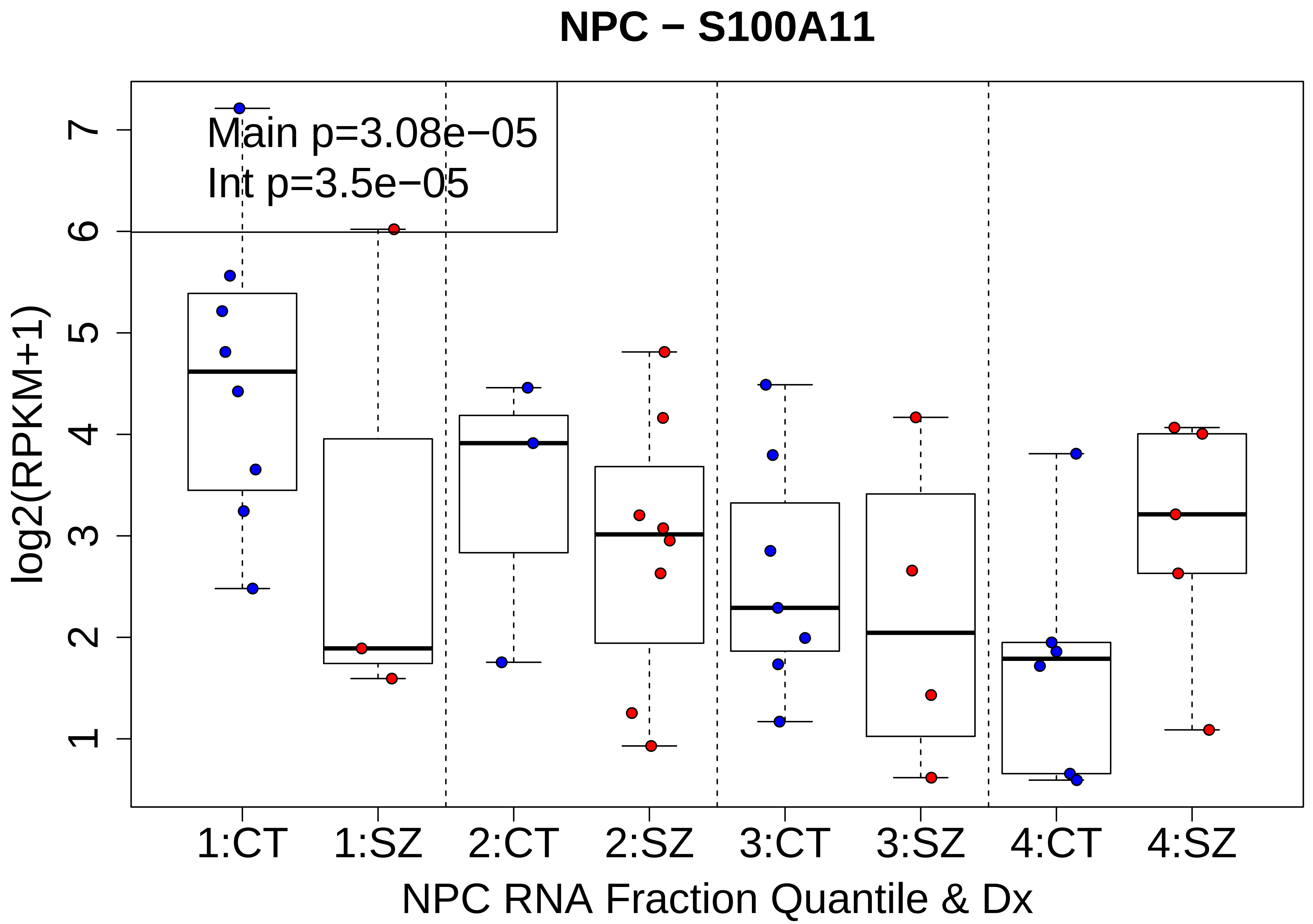
NPC − S100A11
Main p=3.08e−05
Int p=3.5e−05
log2(RPKM+1)
1
2
3
4
5
6
7
1:CT
1:SZ
2:CT
2:SZ
3:CT
3:SZ
4:CT
4:SZ
NPC RNA Fraction Quantile & Dx

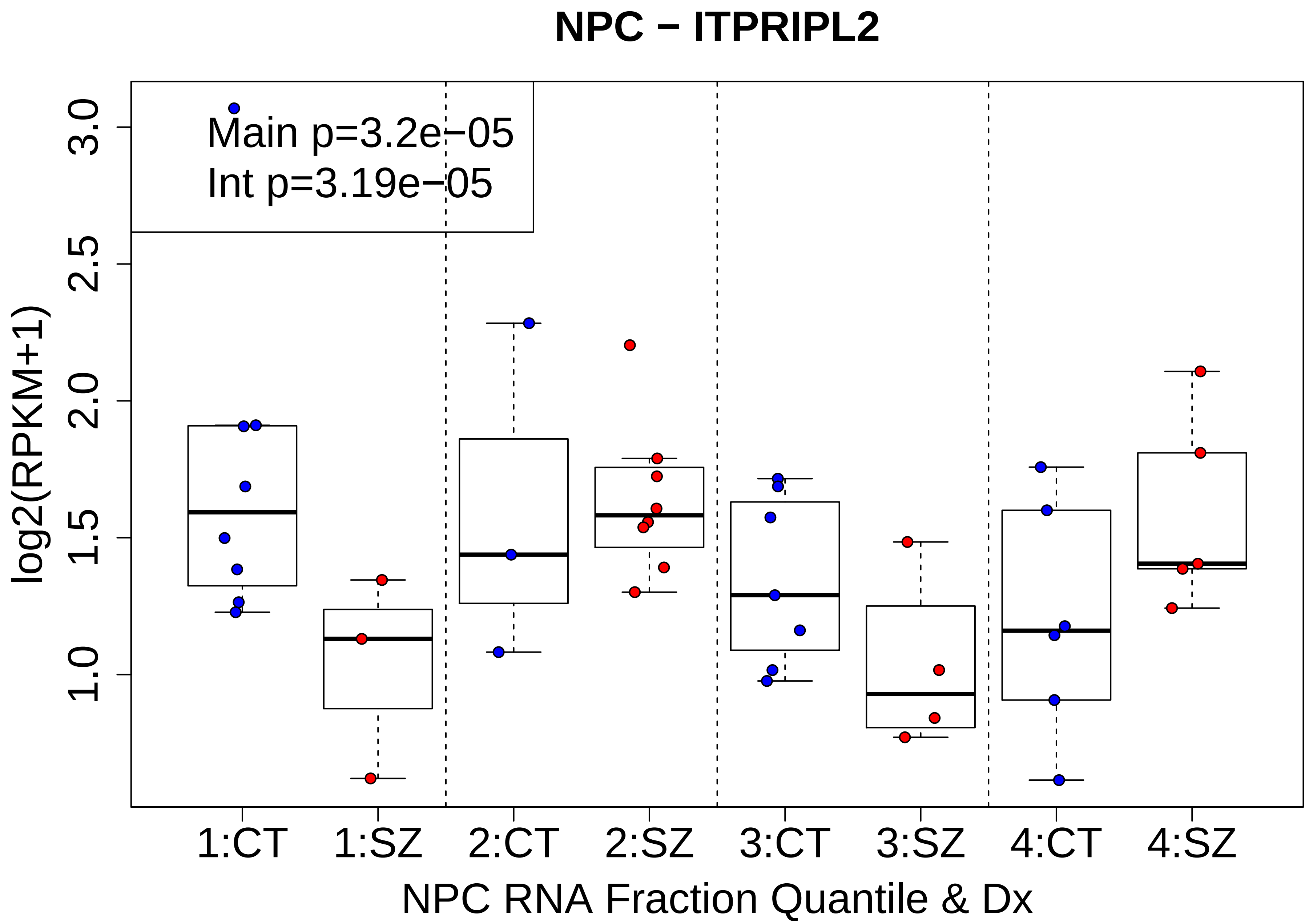
NPC − ITPRIPL2
Main p=3.2e−05
Int p=3.19e−05
log2(RPKM+1)
3.0
2.5
2.0
1.5
1.0
1:CT
1:SZ
2:CT
2:SZ
3:CT
3:SZ
4:CT
4:SZ
NPC RNA Fraction Quantile & Dx

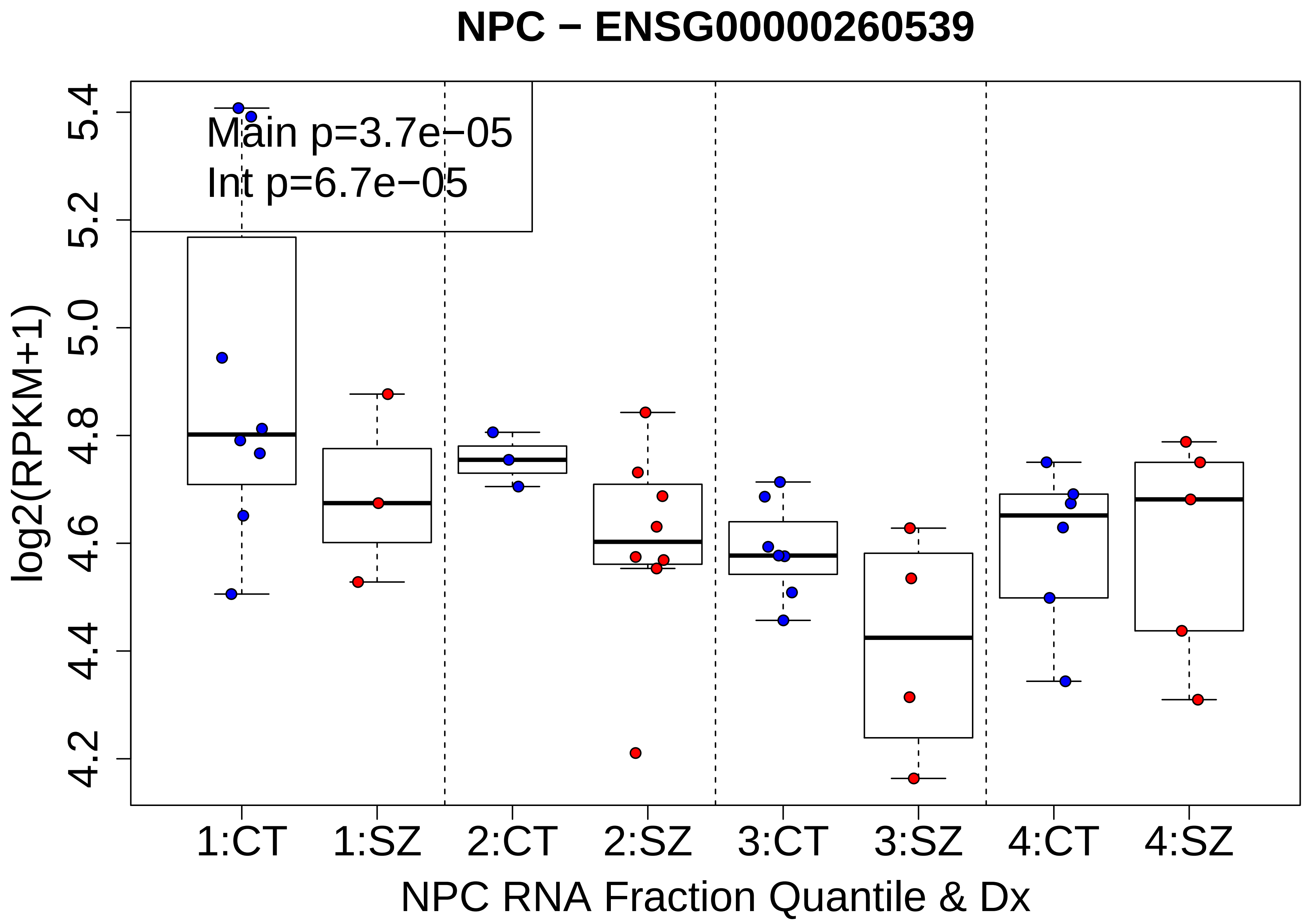

NPC − ENSG00000260539
Main p=3.7e−05
Int p=6.7e−05
log2(RPKM+1)
5.4
5.2
5.0
4.8
4.6
4.4
4.2
1:CT
1:SZ
2:CT
2:SZ
3:CT
3:SZ
4:CT
4:SZ
NPC RNA Fraction Quantile & Dx

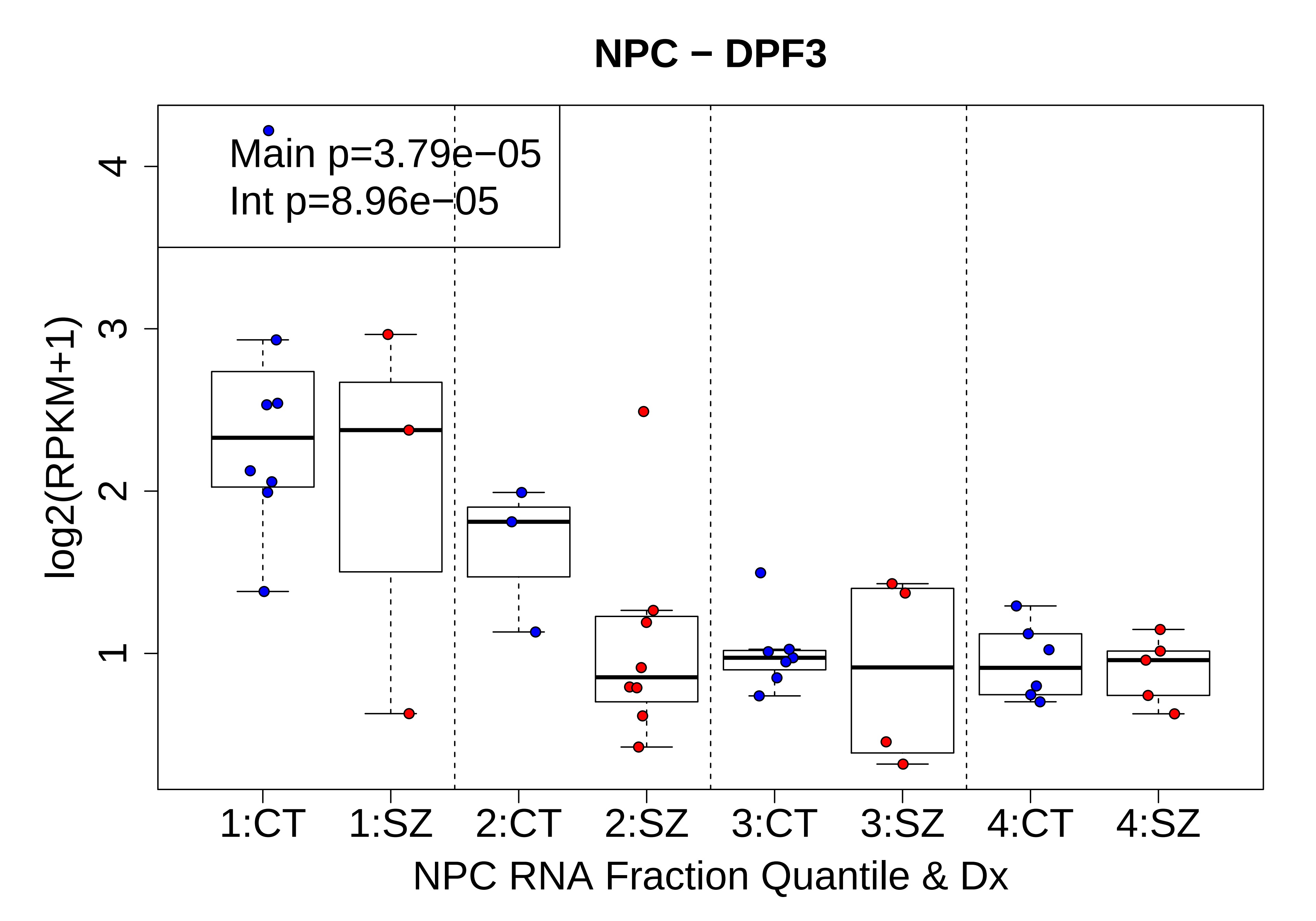

NPC − DPF3
Main p=3.79e−05
Int p=8.96e−05
log2(RPKM+1)
4
3
2
1
1:CT
1:SZ
2:CT
2:SZ
3:CT
3:SZ
4:CT
4:SZ
NPC RNA Fraction Quantile & Dx

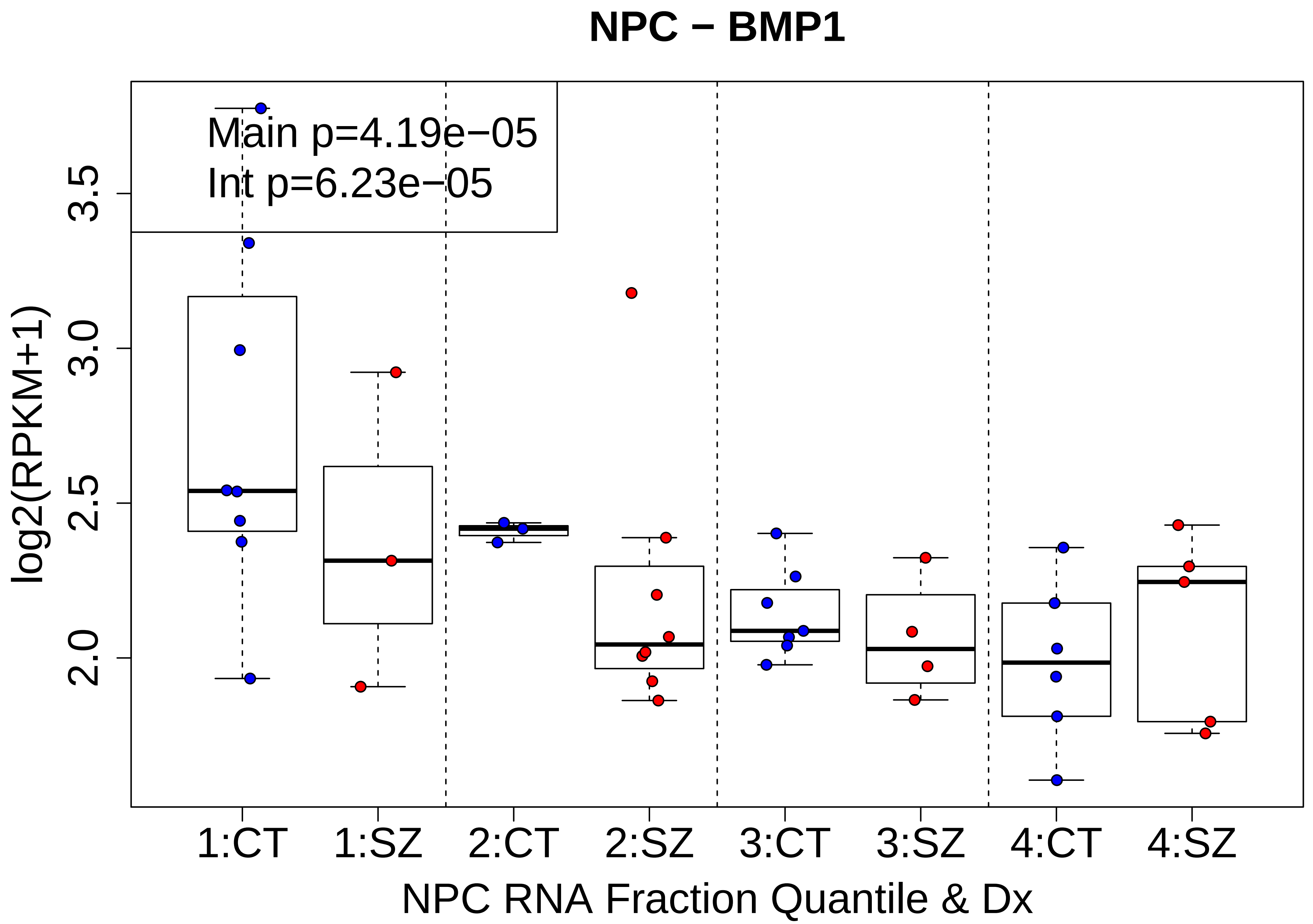

NPC − BMP1
Main p=4.19e−05
Int p=6.23e−05
log2(RPKM+1)
2.0
2.5
3.0
3.5
1:CT
1:SZ
2:CT
2:SZ
3:CT
3:SZ
4:CT
4:SZ
NPC RNA Fraction Quantile & Dx

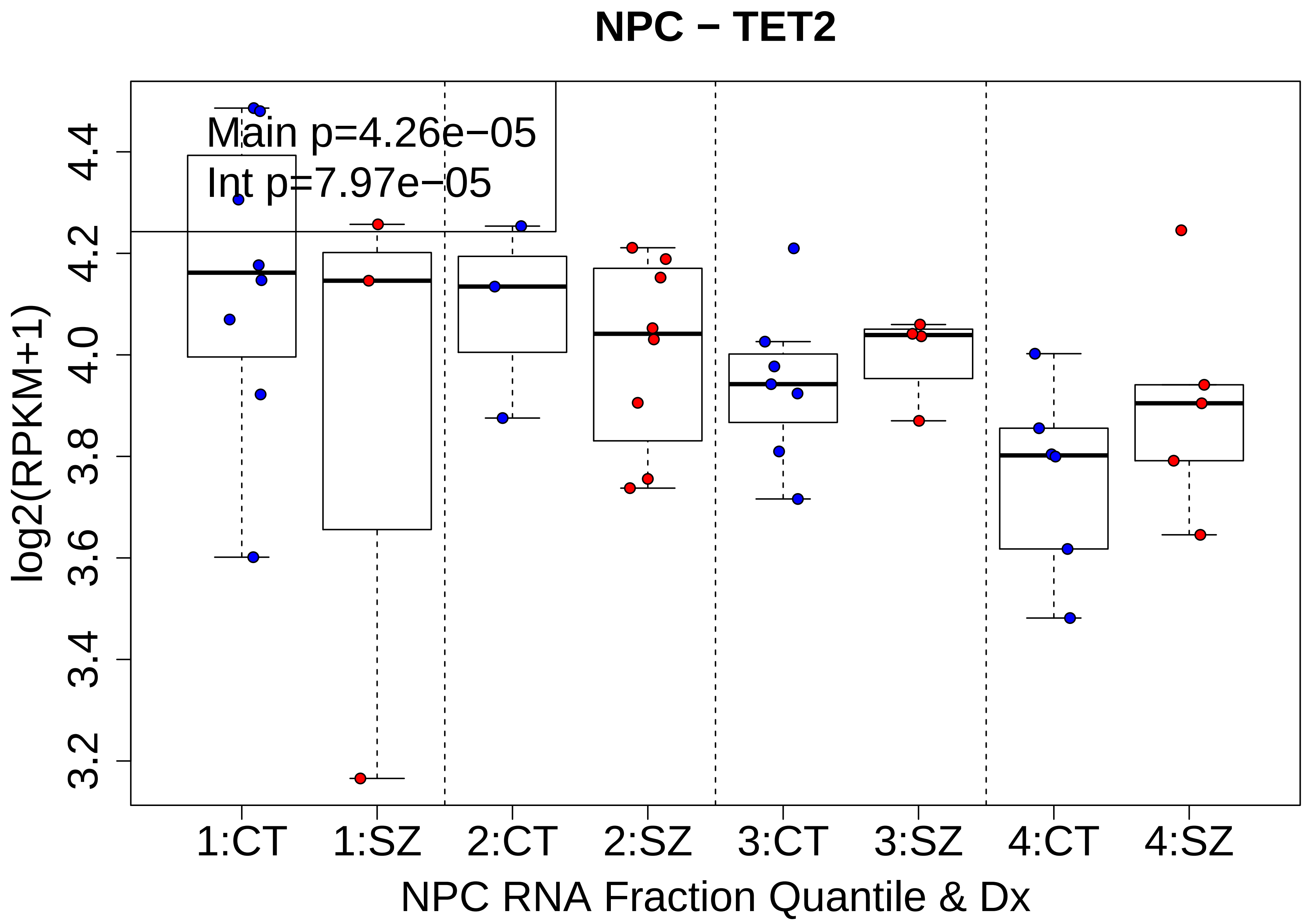
NPC − TET2
Main p=4.26e−05
Int p=7.97e−05
log2(RPKM+1)
3.2
3.4
3.6
3.8
4.0
4.2
4.4
1:CT
1:SZ
2:CT
2:SZ
3:CT
3:SZ
4:CT
4:SZ
NPC RNA Fraction Quantile & Dx

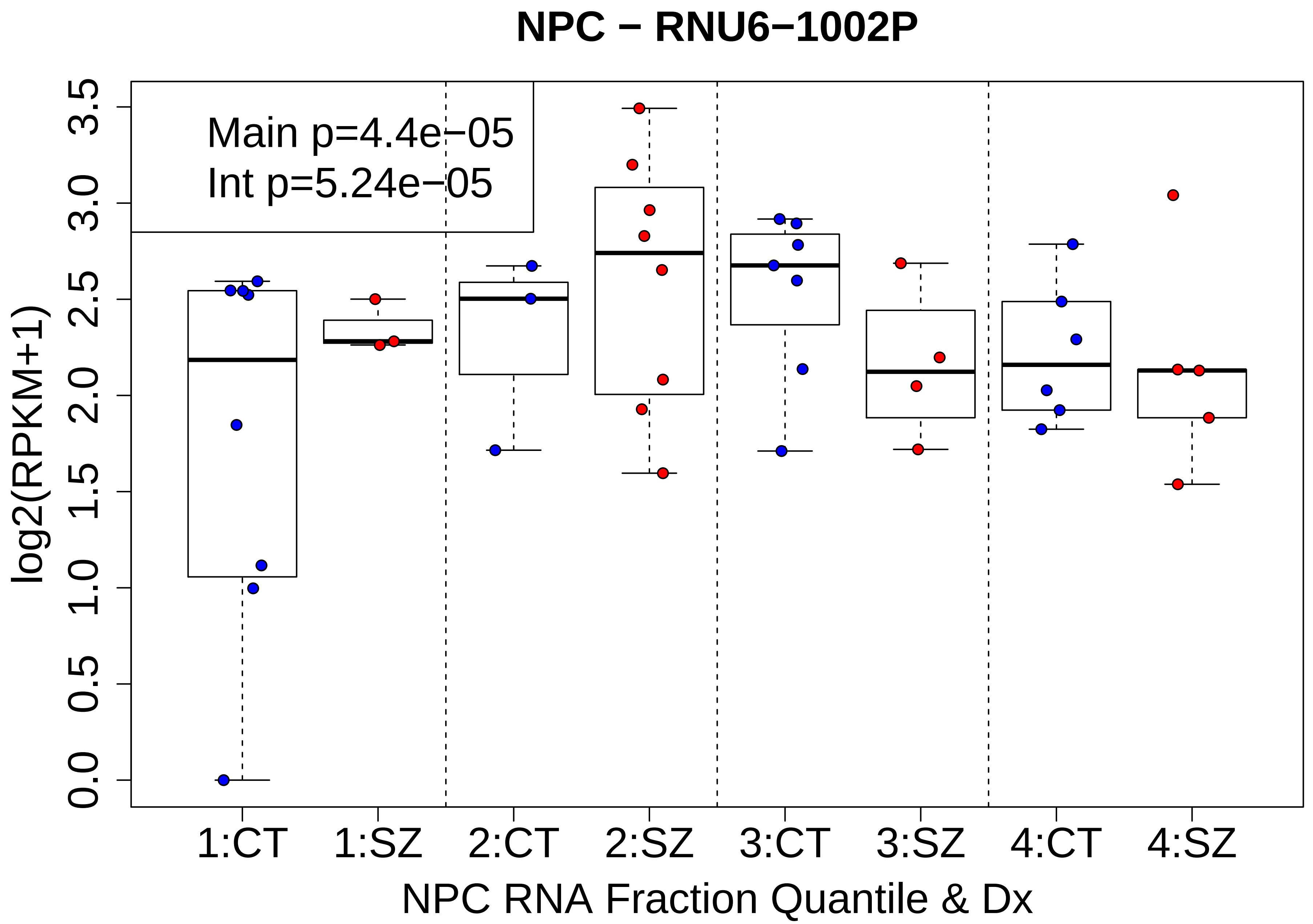
NPC − RNU6−1002P
Main p=4.4e−05
Int p=5.24e−05
log2(RPKM+1)
0.0
0.5
1.0
1.5
2.0
2.5
3.0
3.5
1:CT
1:SZ
2:CT
2:SZ
3:CT
3:SZ
4:CT
4:SZ
NPC RNA Fraction Quantile & Dx

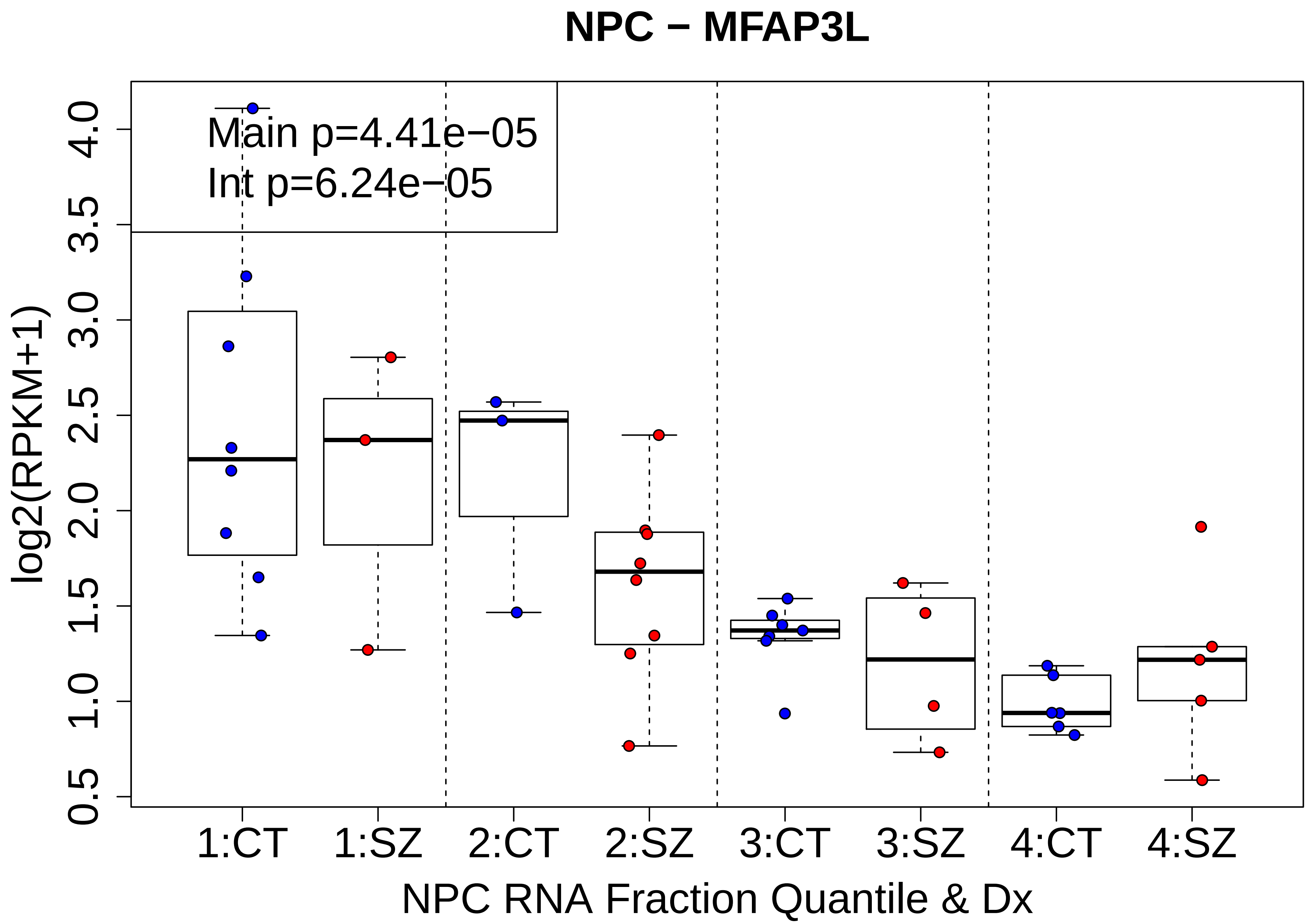
NPC − MFAP3L
Main p=4.41e−05
Int p=6.24e−05
log2(RPKM+1)
0.5
1.0
1.5
2.0
2.5
3.0
3.5
4.0
1:CT
1:SZ
2:CT
2:SZ
3:CT
3:SZ
4:CT
4:SZ
NPC RNA Fraction Quantile & Dx

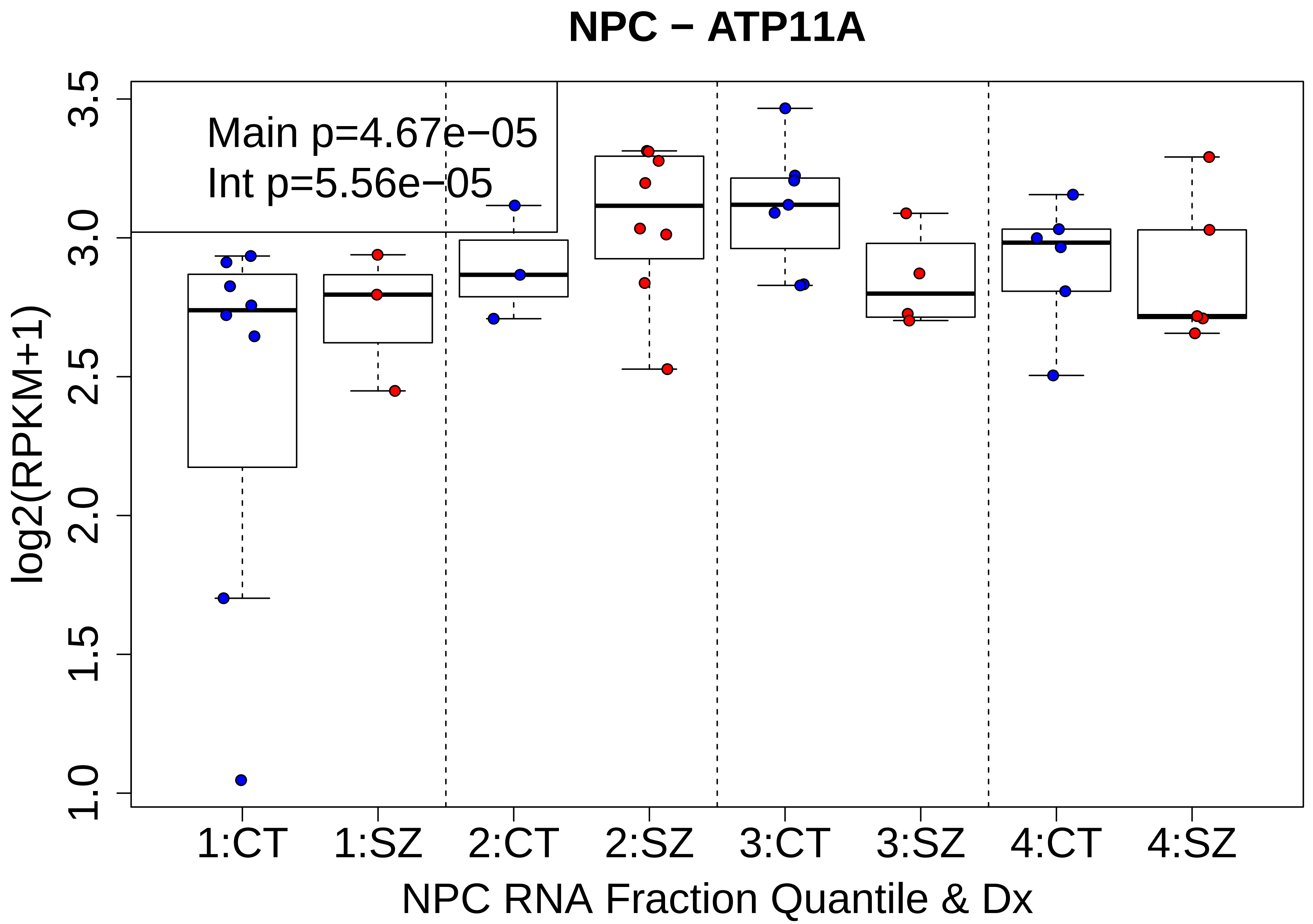

NPC − ATP11A
Main p=4.67e−05
Int p=5.56e−05
log2(RPKM+1)
3.5
3.0
2.5
2.0
1.5
1.0
1:CT
1:SZ
2:CT
2:SZ
3:CT
3:SZ
4:CT
4:SZ
NPC RNA Fraction Quantile & Dx

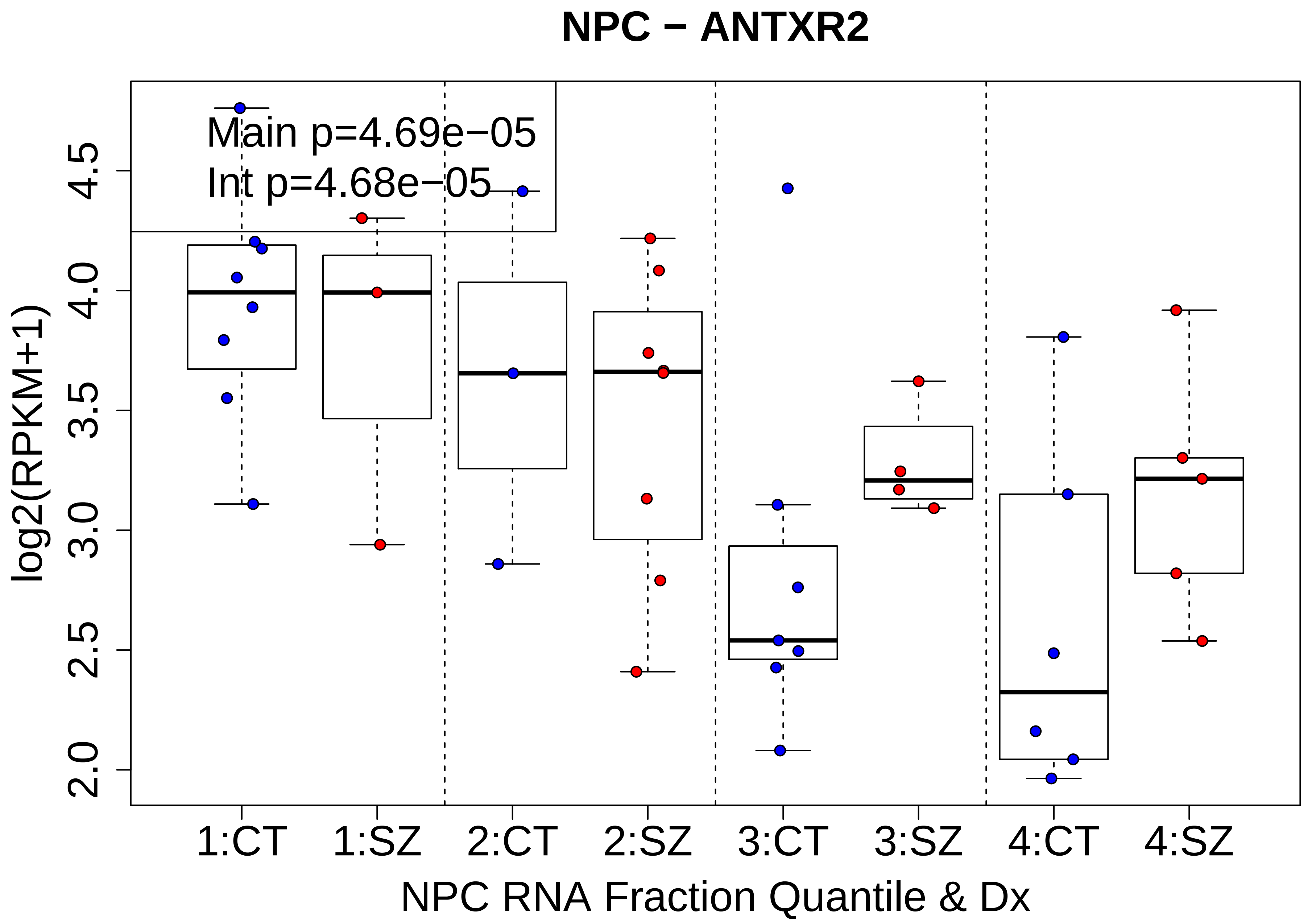

NPC − ANTXR2
Main p=4.69e−05
Int p=4.68e−05
log2(RPKM+1)
2.0
2.5
3.0
3.5
4.0
4.5
1:CT
1:SZ
2:CT
2:SZ
3:CT
3:SZ
4:CT
4:SZ
NPC RNA Fraction Quantile & Dx

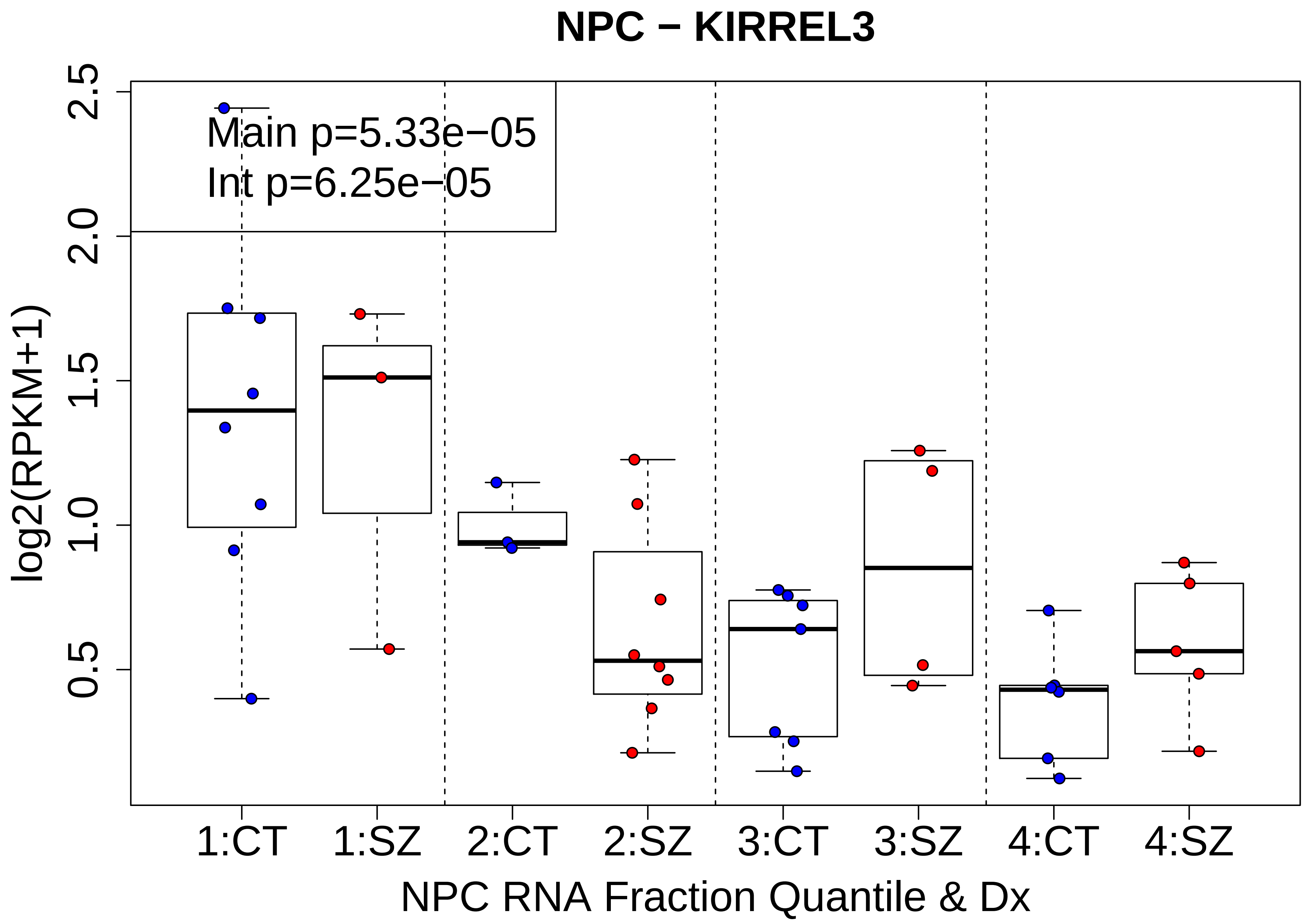
NPC − KIRREL3
Main p=5.33e−05
Int p=6.25e−05
log2(RPKM+1)
2.5
2.0
1.5
1.0
0.5
1:CT
1:SZ
2:CT
2:SZ
3:CT
3:SZ
4:CT
4:SZ
NPC RNA Fraction Quantile & Dx

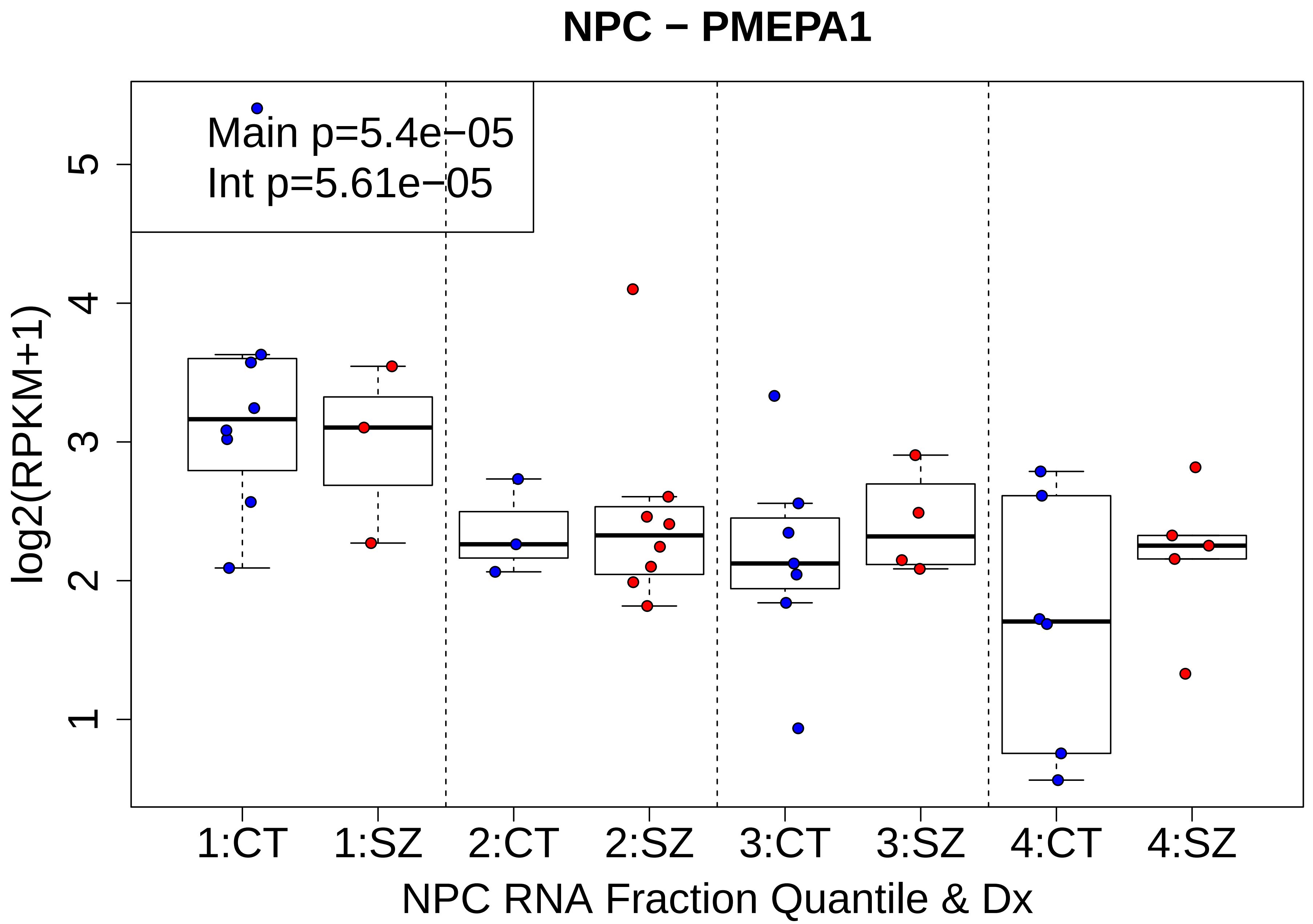

NPC − PMEPA1
Main p=5.4e−05
Int p=5.61e−05
log2(RPKM+1)
1
2
3
4
5
1:CT
1:SZ
2:CT
2:SZ
3:CT
3:SZ
4:CT
4:SZ
NPC RNA Fraction Quantile & Dx

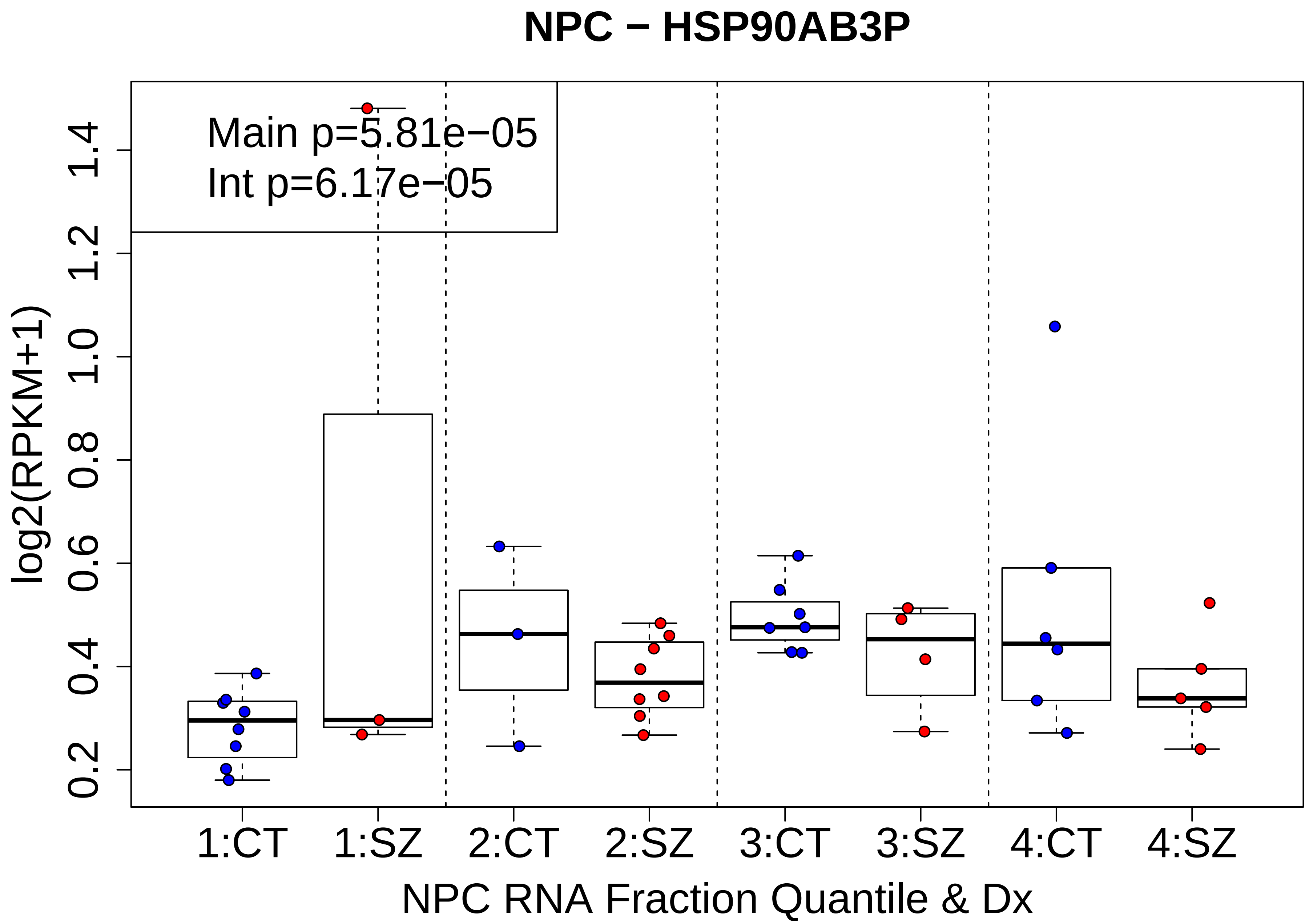
NPC − HSP90AB3P
Main p=5.81e−05
Int p=6.17e−05
log2(RPKM+1)
0.2
0.4
0.6
0.8
1.0
1.2
1.4
1:CT
1:SZ
2:CT
2:SZ
3:CT
3:SZ
4:CT
4:SZ
NPC RNA Fraction Quantile & Dx

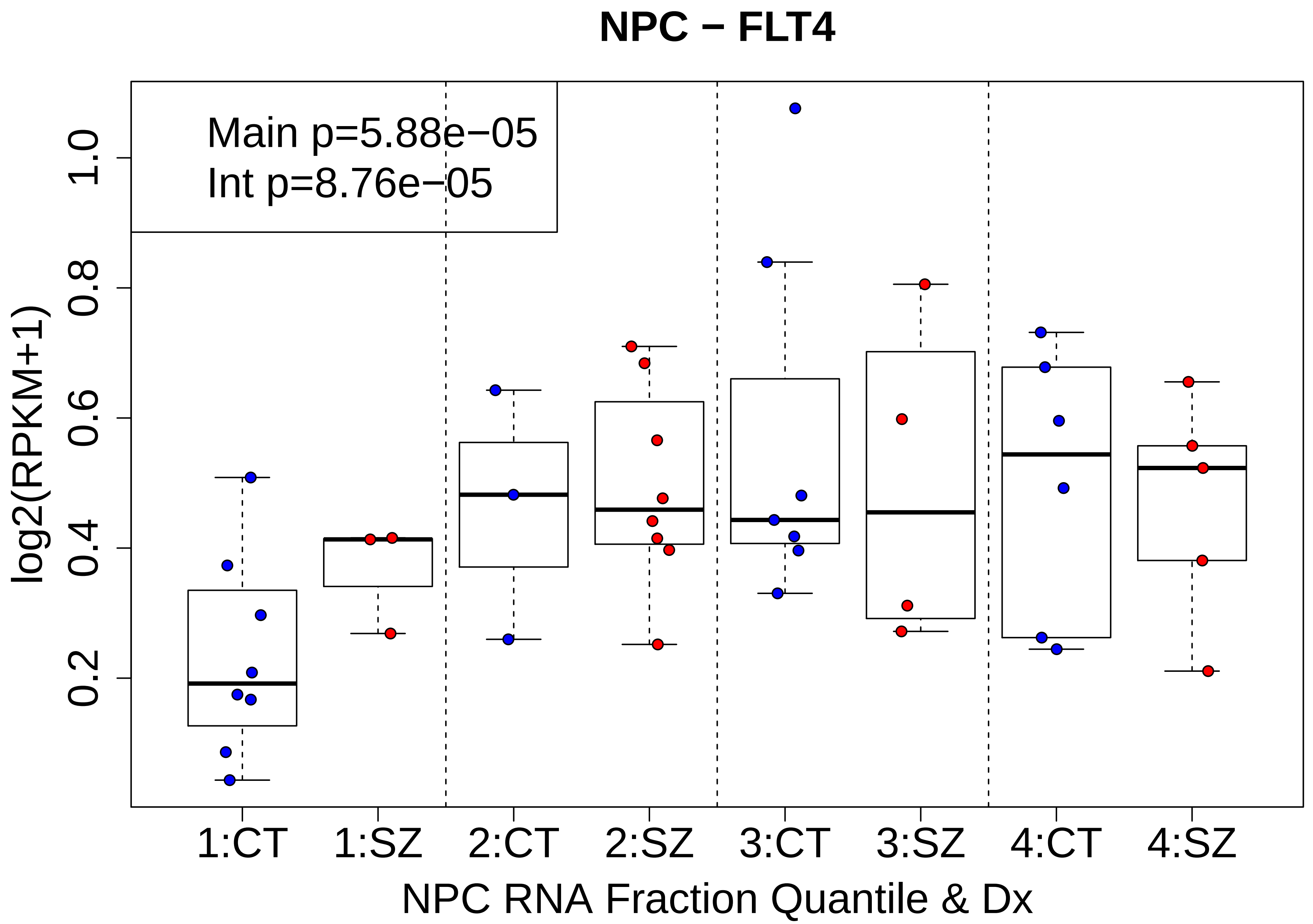

NPC − FLT4
Main p=5.88e−05
Int p=8.76e−05
log2(RPKM+1)
0.2
0.4
0.6
0.8
1.0
1:CT
1:SZ
2:CT
2:SZ
3:CT
3:SZ
4:CT
4:SZ
NPC RNA Fraction Quantile & Dx

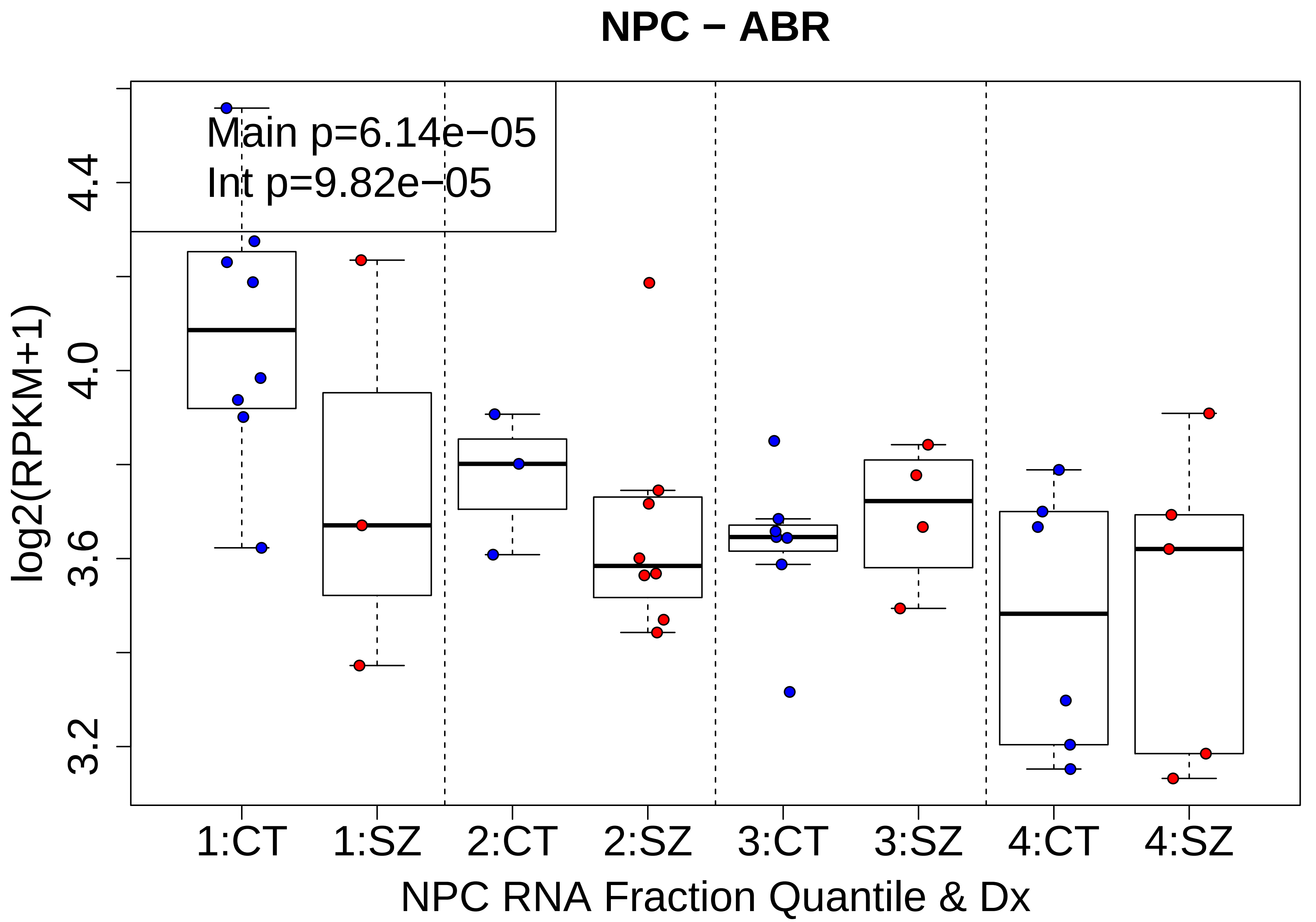

NPC − ABR
Main p=6.14e−05
Int p=9.82e−05
log2(RPKM+1)
4.4
4.0
3.6
3.2
1:CT
1:SZ
2:CT
2:SZ
3:CT
3:SZ
4:CT
4:SZ
NPC RNA Fraction Quantile & Dx

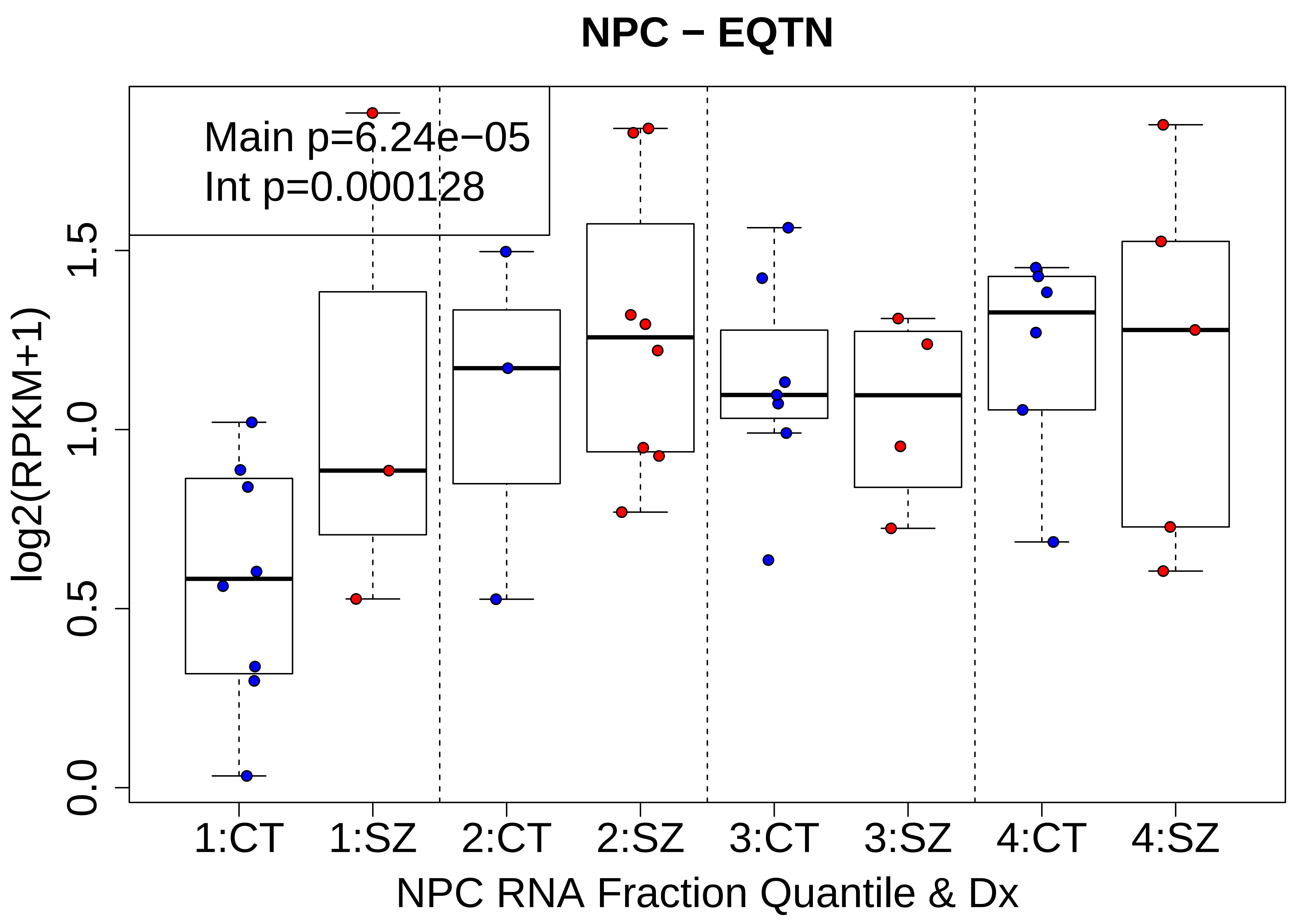
NPC − EQTN
Main p=6.24e−05
Int p=0.000128
log2(RPKM+1)
0.0
0.5
1.0
1.5
1:CT
1:SZ
2:CT
2:SZ
3:CT
3:SZ
4:CT
4:SZ
NPC RNA Fraction Quantile & Dx

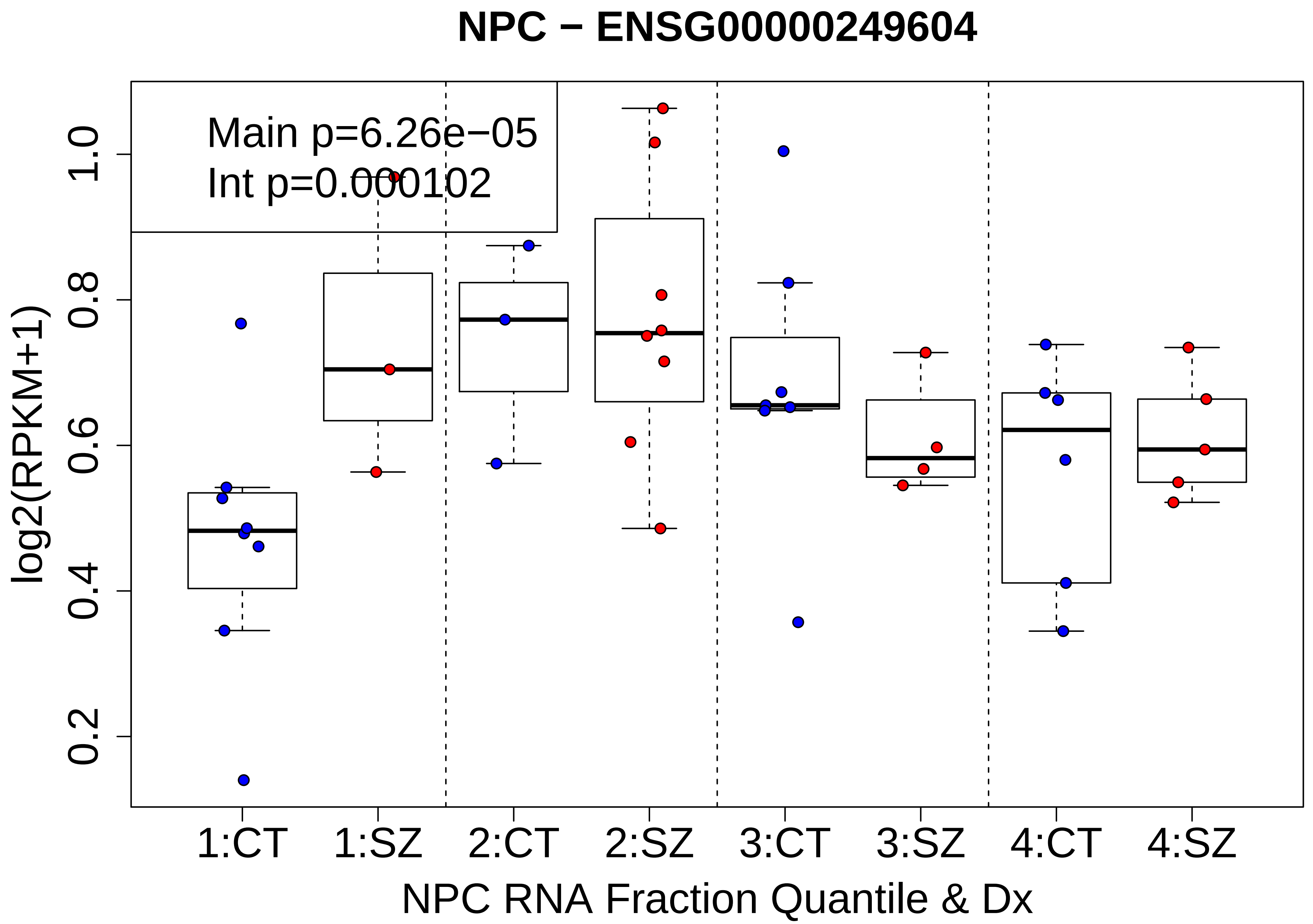
NPC − ENSG00000249604
Main p=6.26e−05
Int p=0.000102
log2(RPKM+1)
0.2
0.4
0.6
0.8
1.0
1:CT
1:SZ
2:CT
2:SZ
3:CT
3:SZ
4:CT
4:SZ
NPC RNA Fraction Quantile & Dx

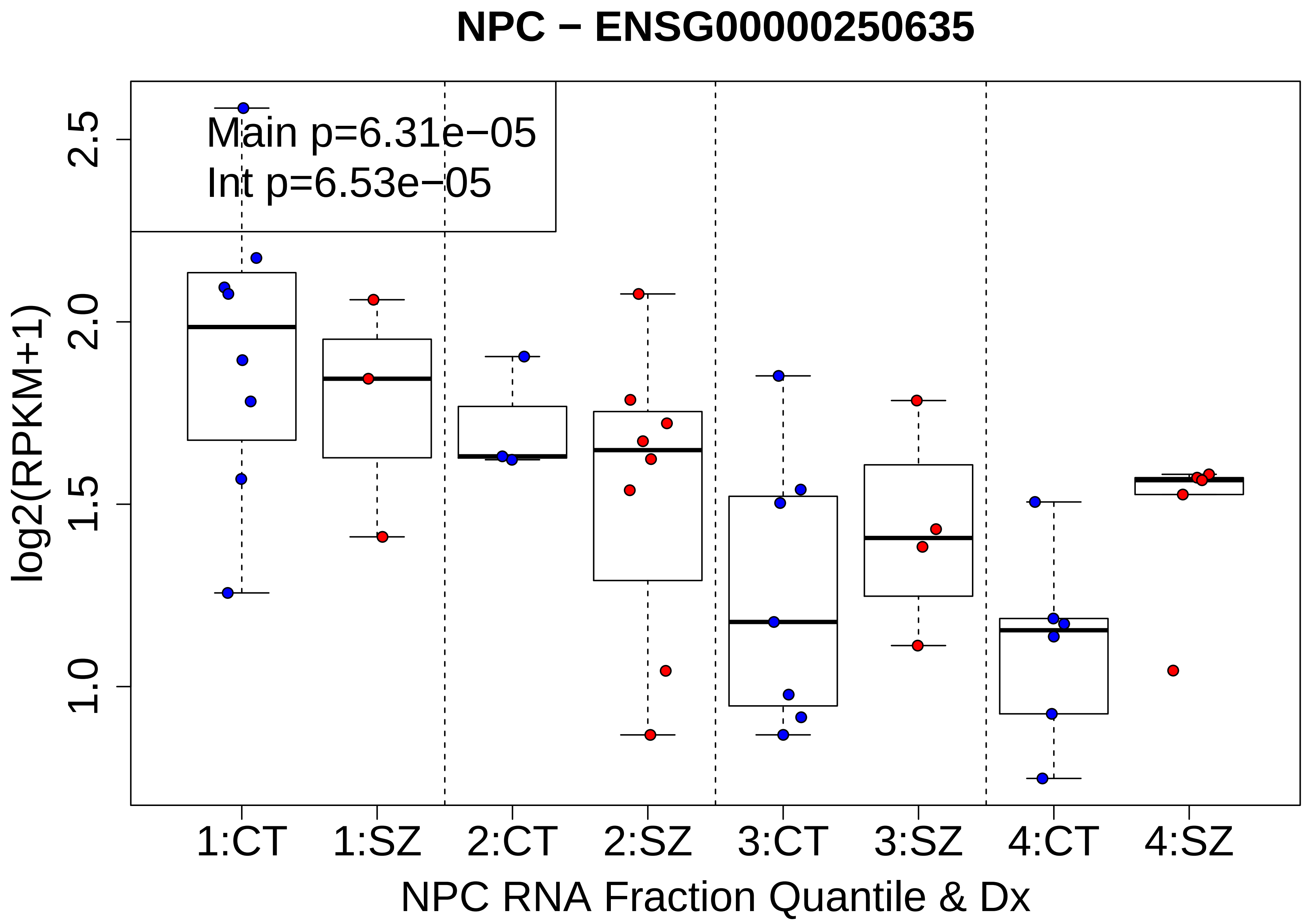

NPC − ENSG00000250635
Main p=6.31e−05
Int p=6.53e−05
log2(RPKM+1)
2.5
2.0
1.5
1.0
1:CT
1:SZ
2:CT
2:SZ
3:CT
3:SZ
4:CT
4:SZ
NPC RNA Fraction Quantile & Dx

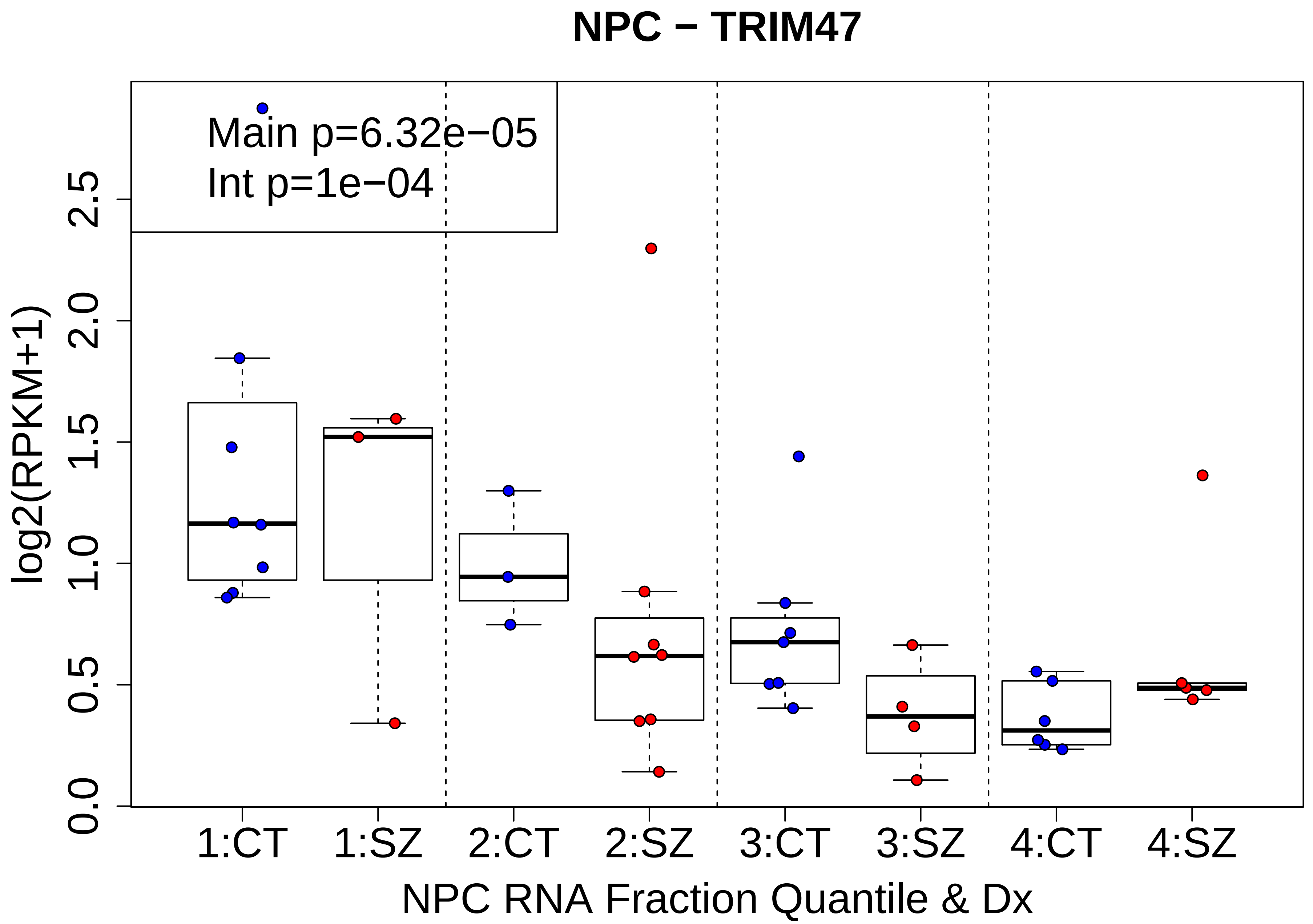
NPC − TRIM47
Main p=6.32e−05
Int p=1e−04
log2(RPKM+1)
0.0
0.5
1.0
1.5
2.0
2.5
1:CT
1:SZ
2:CT
2:SZ
3:CT
3:SZ
4:CT
4:SZ
NPC RNA Fraction Quantile & Dx

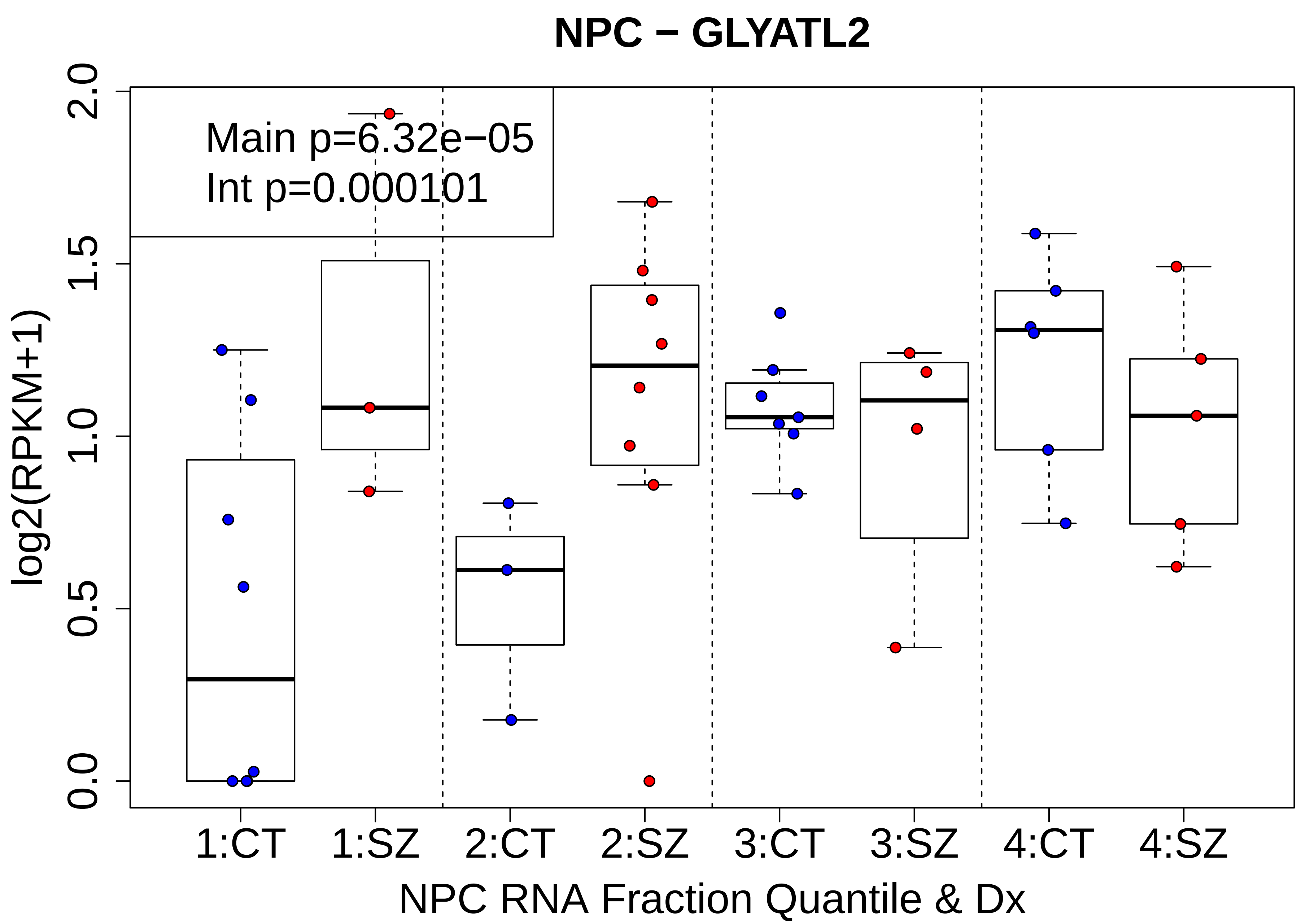

NPC − GLYATL2
Main p=6.32e−05
Int p=0.000101
log2(RPKM+1)
0.0
0.5
1.0
1.5
2.0
1:CT
1:SZ
2:CT
2:SZ
3:CT
3:SZ
4:CT
4:SZ
NPC RNA Fraction Quantile & Dx

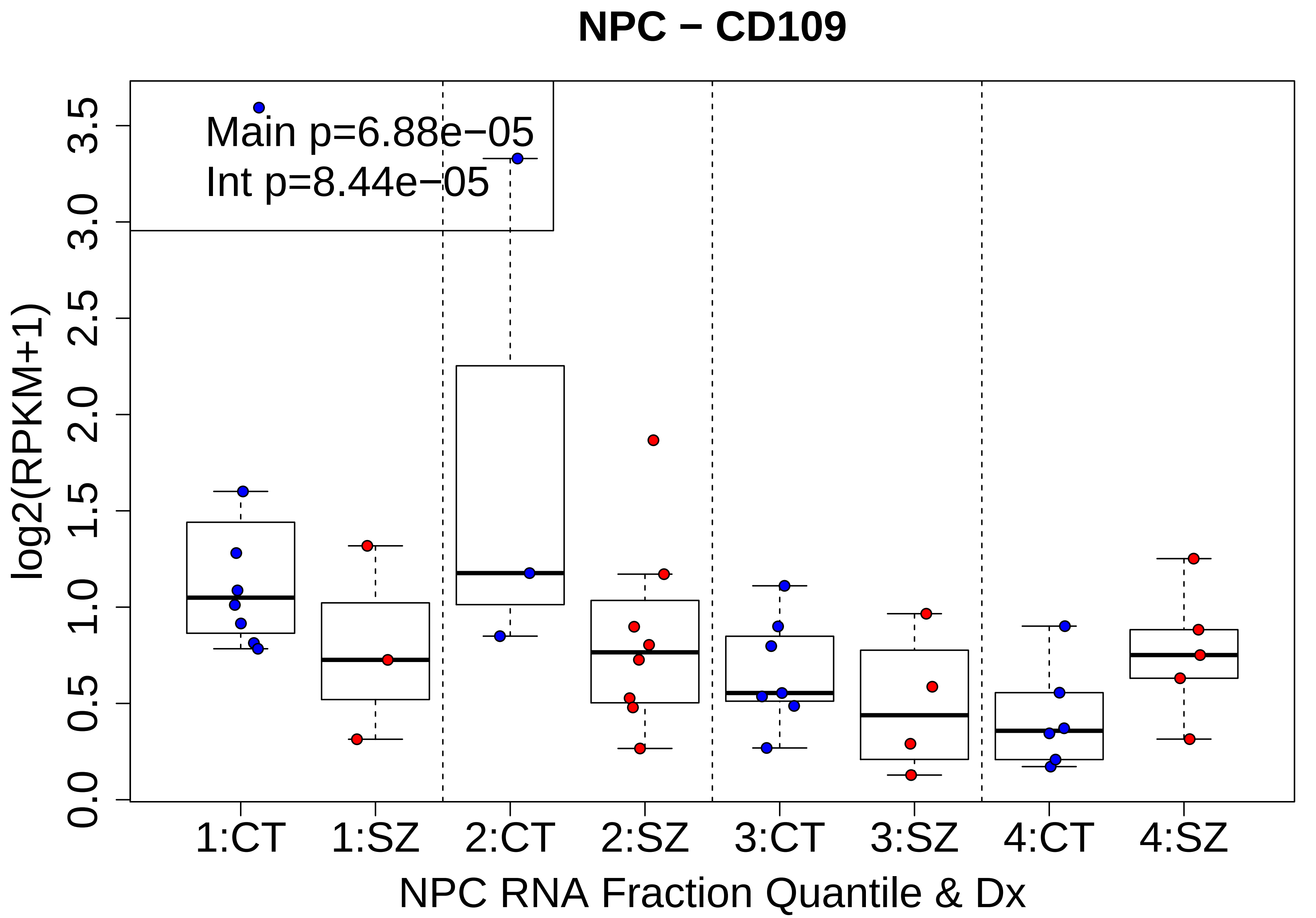

NPC − CD109
Main p=6.88e−05
Int p=8.44e−05
log2(RPKM+1)
0.0
0.5
1.0
1.5
2.0
2.5
3.0
3.5
1:CT
1:SZ
2:CT
2:SZ
3:CT
3:SZ
4:CT
4:SZ
NPC RNA Fraction Quantile & Dx

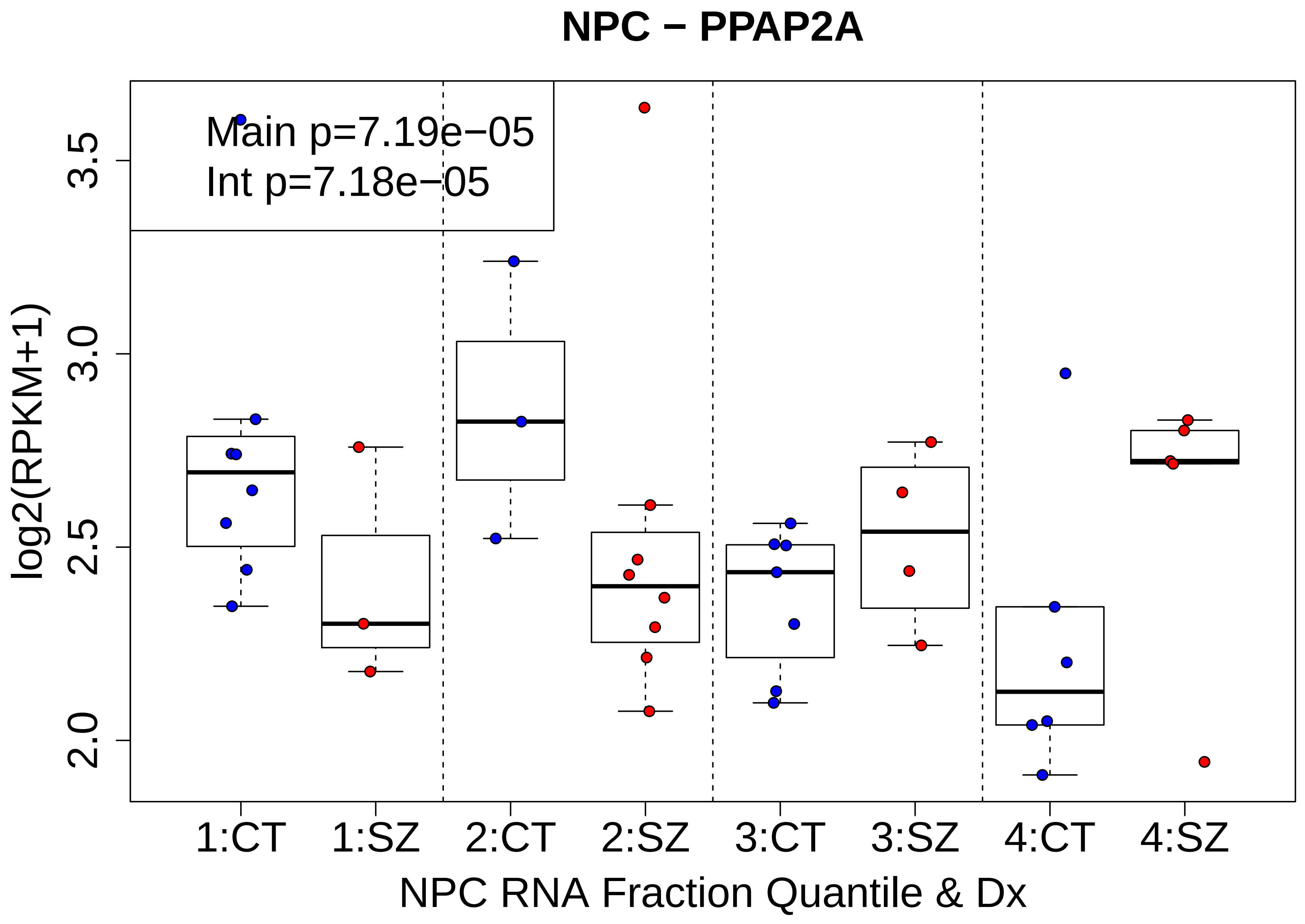

NPC − PPAP2A
Main p=7.19e−05
Int p=7.18e−05
log2(RPKM+1)
3.5
3.0
2.5
2.0
1:CT
1:SZ
2:CT
2:SZ
3:CT
3:SZ
4:CT
4:SZ
NPC RNA Fraction Quantile & Dx

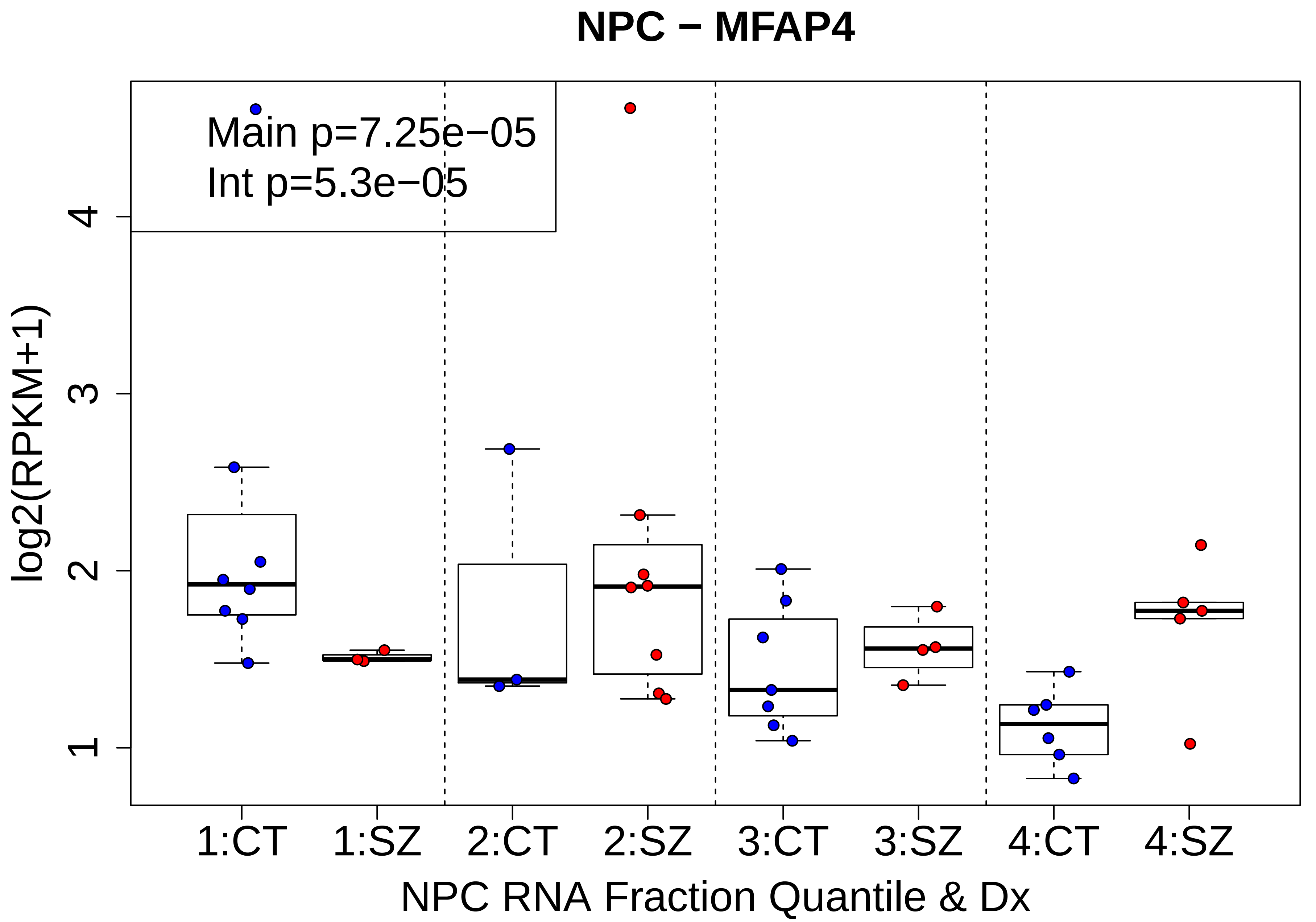
NPC − MFAP4
Main p=7.25e−05
Int p=5.3e−05
log2(RPKM+1)
1
2
3
4
1:CT
1:SZ
2:CT
2:SZ
3:CT
3:SZ
4:CT
4:SZ
NPC RNA Fraction Quantile & Dx

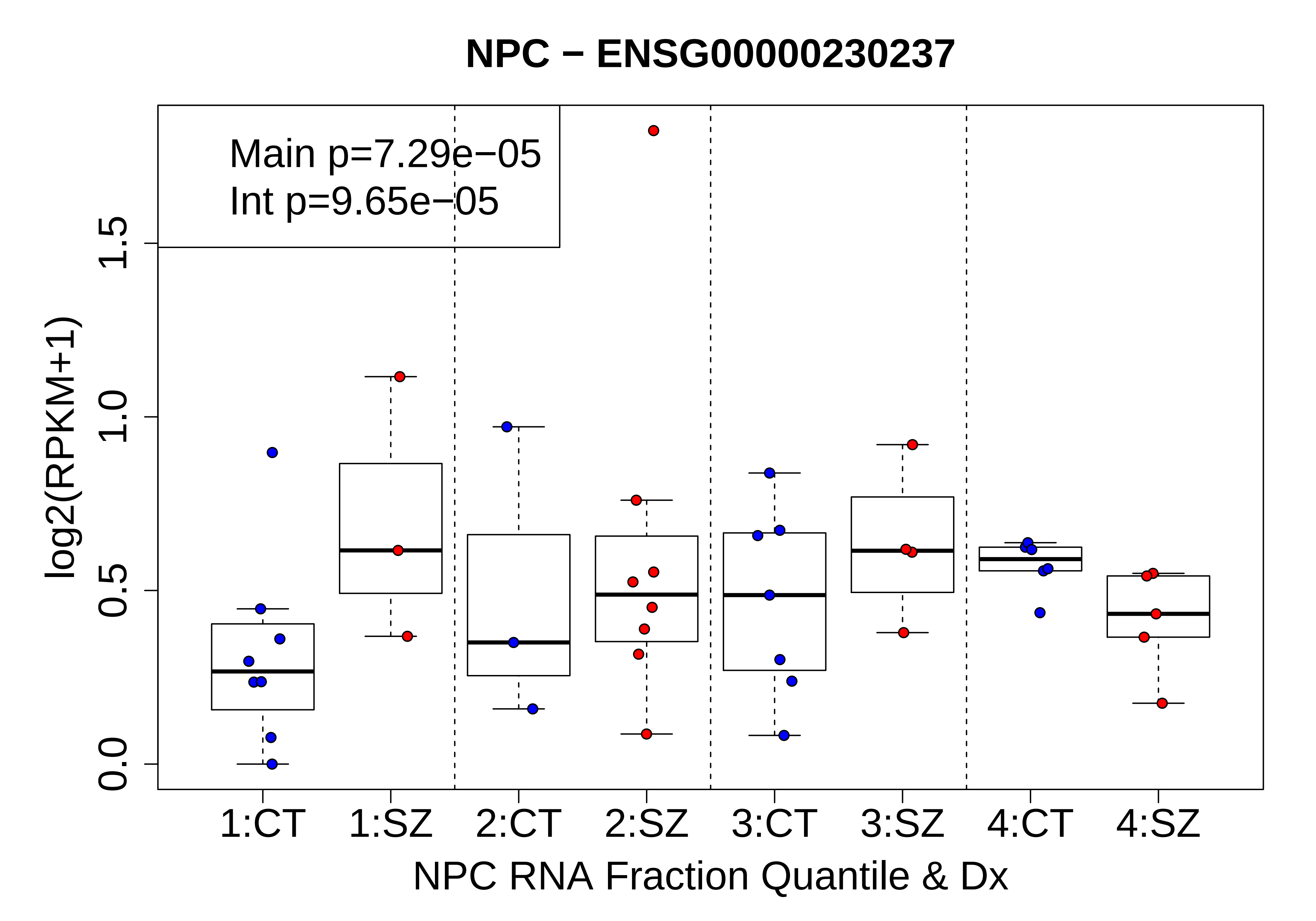

NPC − ENSG00000230237
Main p=7.29e−05
Int p=9.65e−05
log2(RPKM+1)
0.0
0.5
1.0
1.5
1:CT
1:SZ
2:CT
2:SZ
3:CT
3:SZ
4:CT
4:SZ
NPC RNA Fraction Quantile & Dx

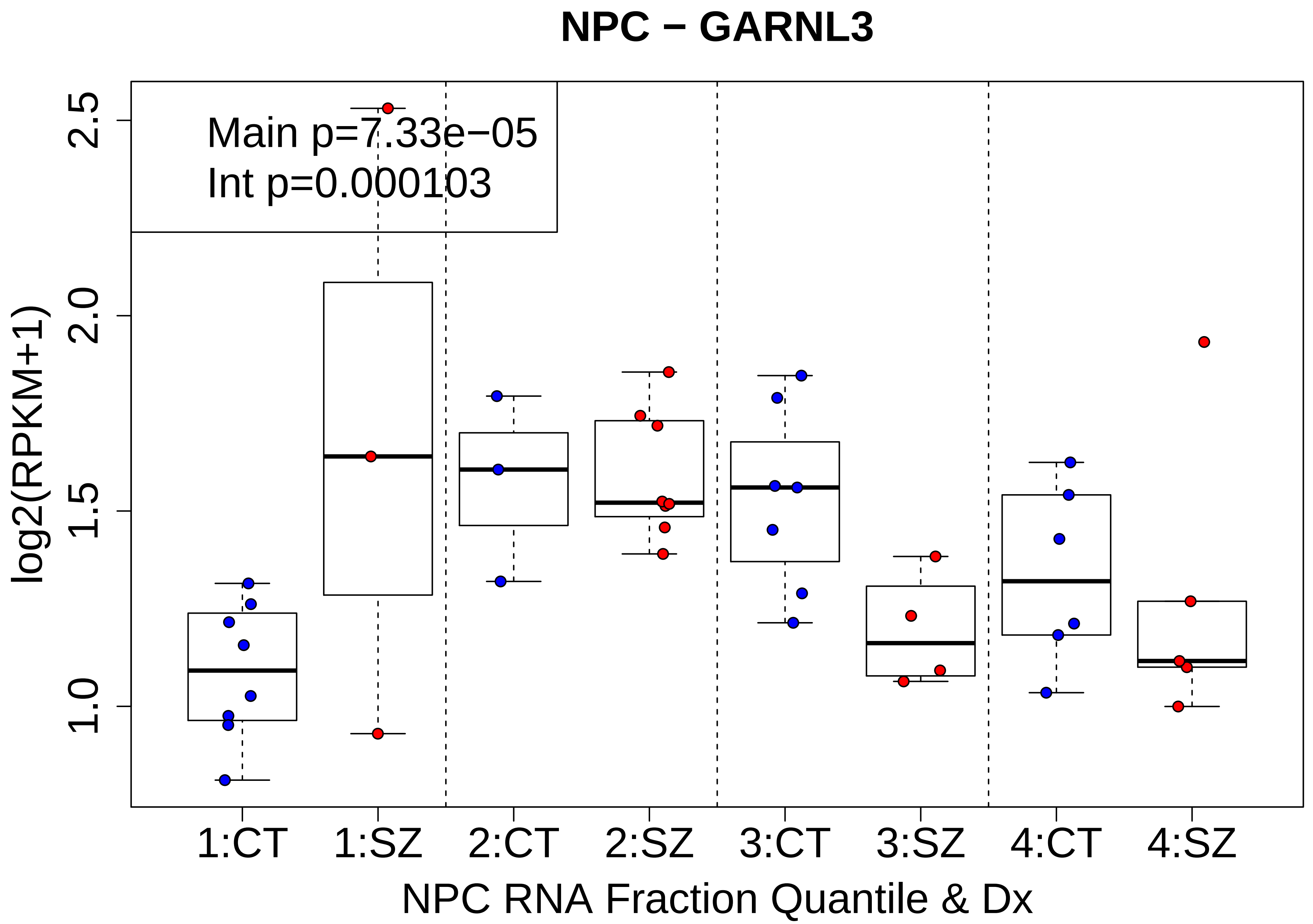

NPC − GARNL3
Main p=7.33e−05
Int p=0.000103
log2(RPKM+1)
2.5
2.0
1.5
1.0
1:CT
1:SZ
2:CT
2:SZ
3:CT
3:SZ
4:CT
4:SZ
NPC RNA Fraction Quantile & Dx

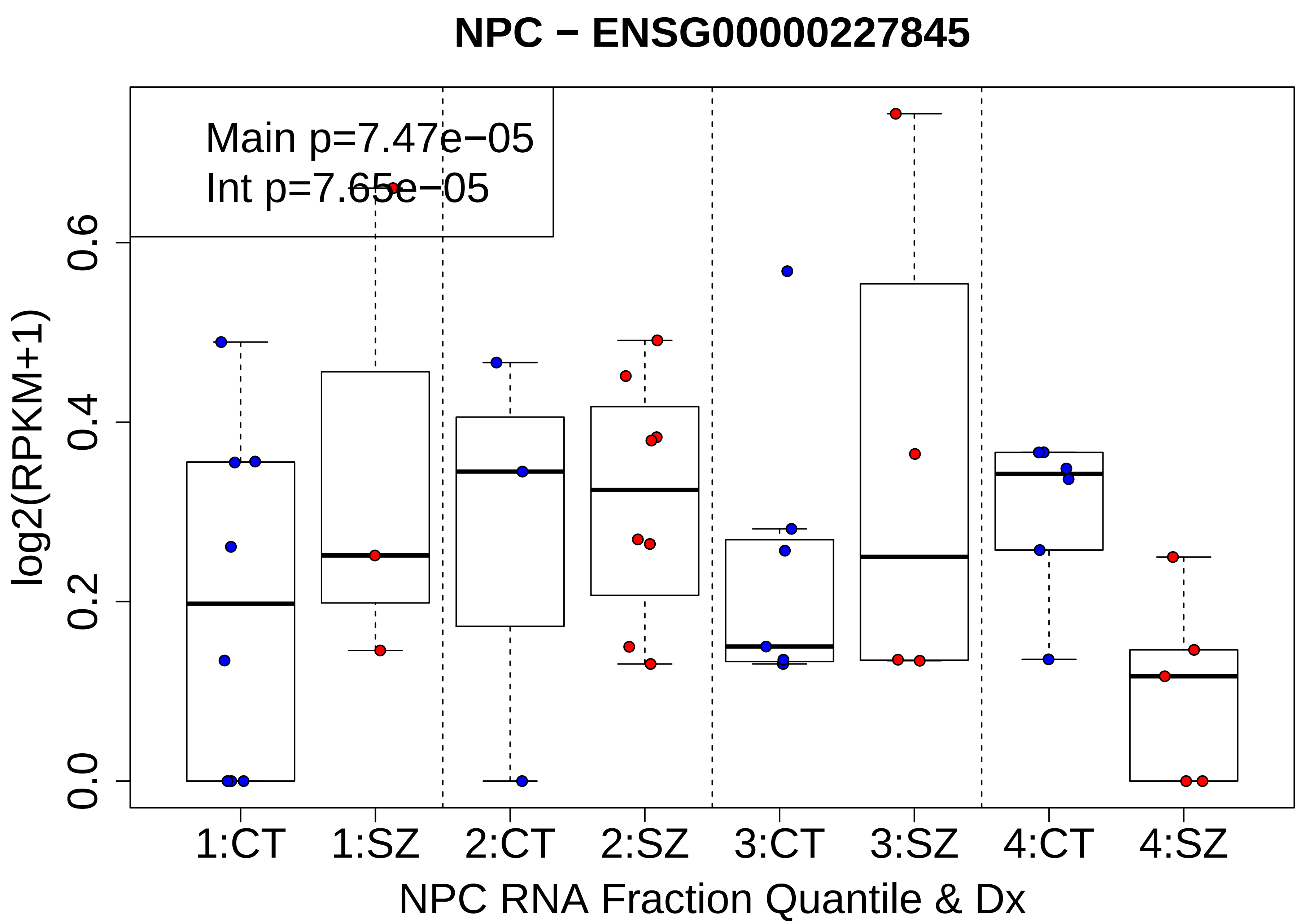
NPC − ENSG00000227845
Main p=7.47e−05
Int p=7.65e−05
log2(RPKM+1)
0.0
0.2
0.4
0.6
1:CT
1:SZ
2:CT
2:SZ
3:CT
3:SZ
4:CT
4:SZ
NPC RNA Fraction Quantile & Dx

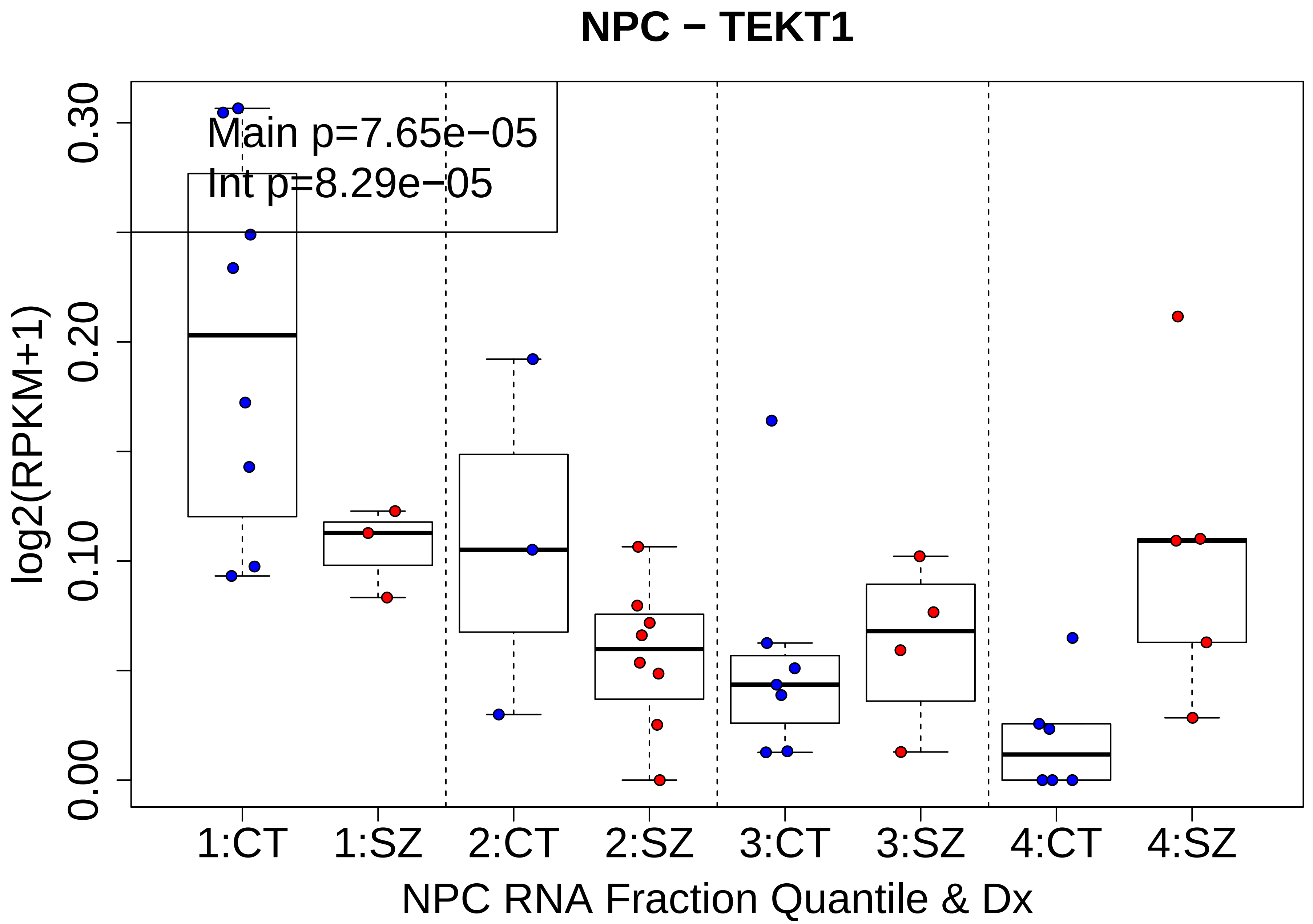

NPC − TEKT1
Main p=7.65e−05
Int p=8.29e−05
log2(RPKM+1)
0.00
0.10
0.20
0.30
1:CT
1:SZ
2:CT
2:SZ
3:CT
3:SZ
4:CT
4:SZ
NPC RNA Fraction Quantile & Dx

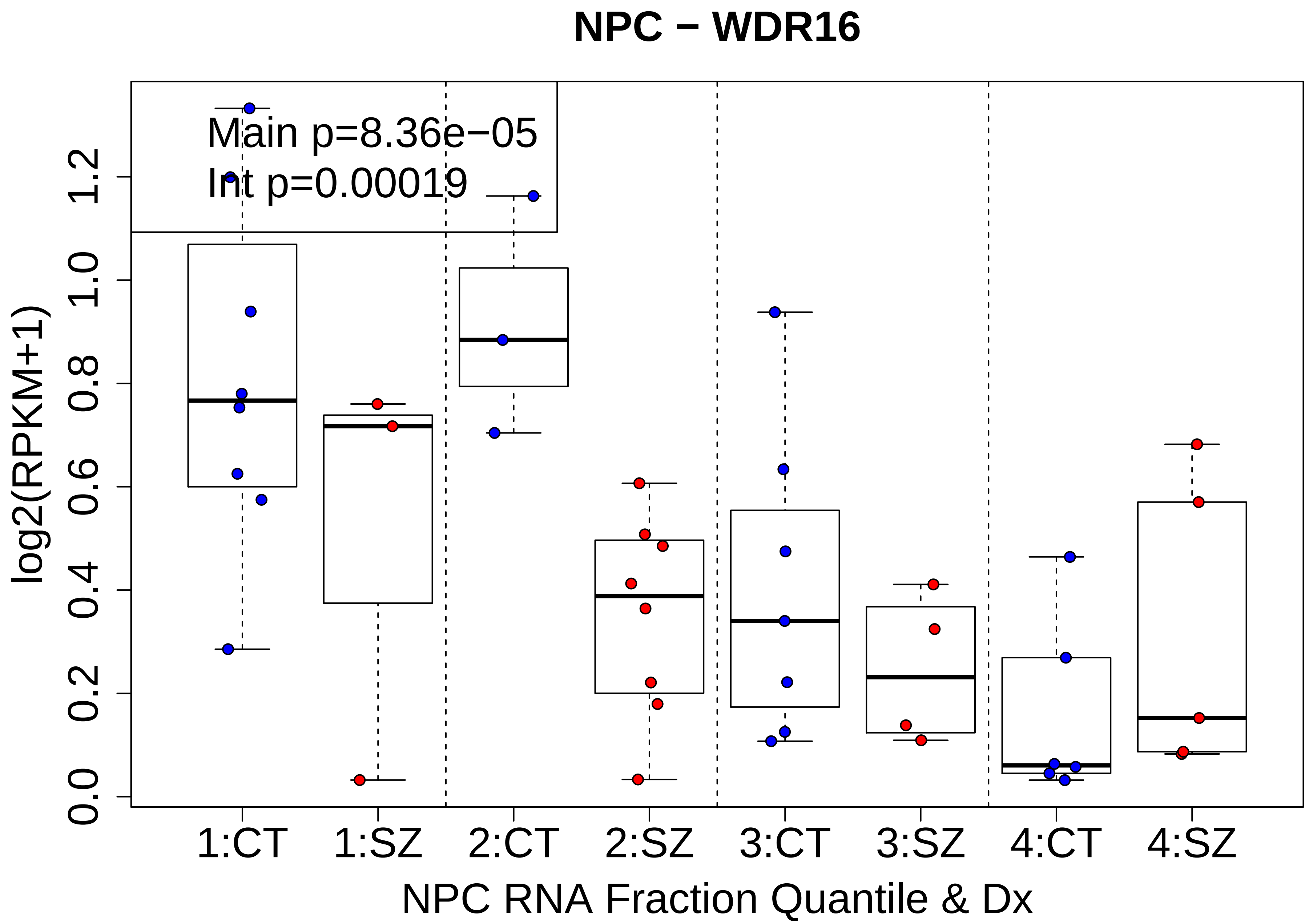
NPC − WDR16
Main p=8.36e−05
Int p=0.00019
log2(RPKM+1)
0.0
0.2
0.4
0.6
0.8
1.0
1.2
1:CT
1:SZ
2:CT
2:SZ
3:CT
3:SZ
4:CT
4:SZ
NPC RNA Fraction Quantile & Dx

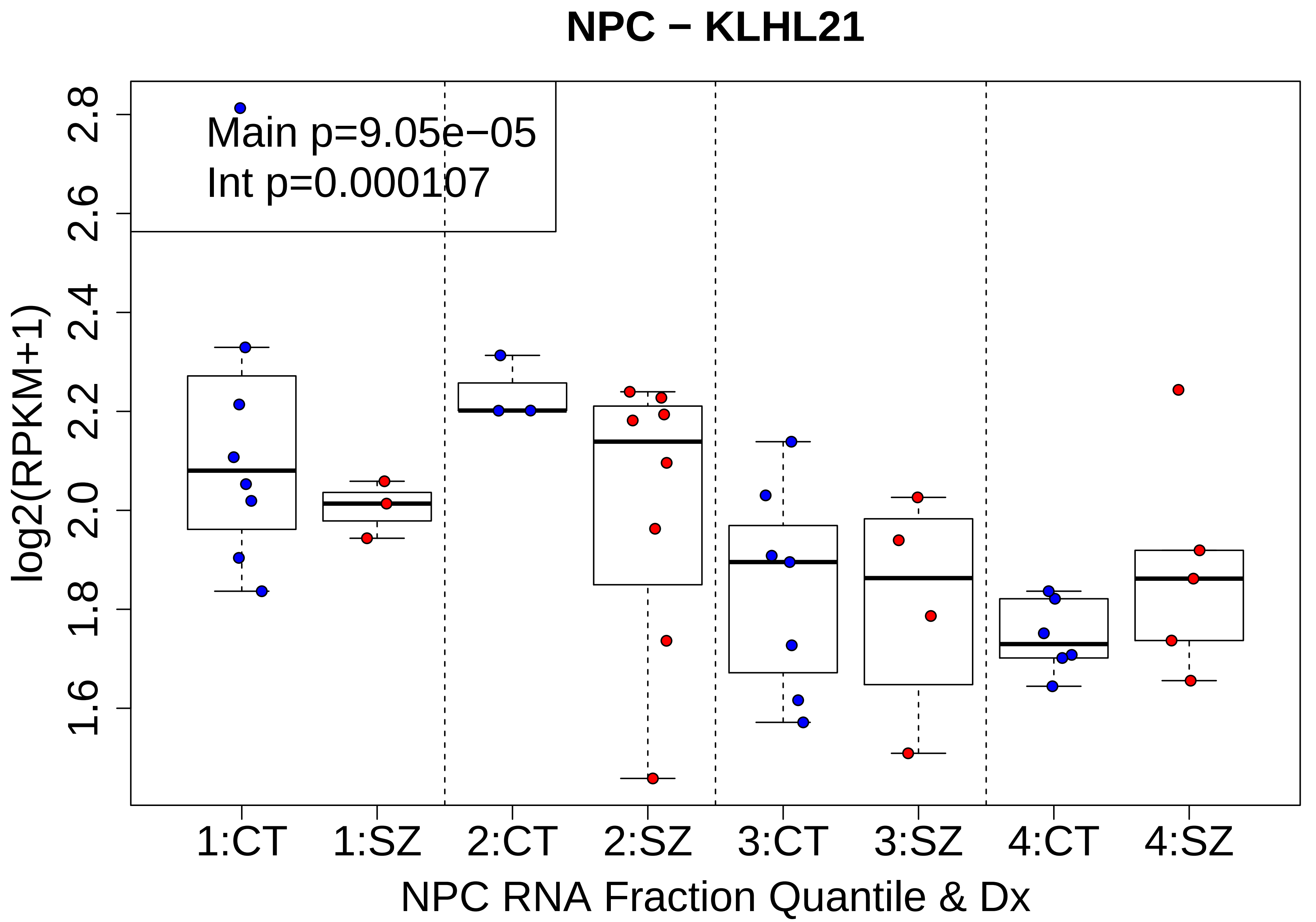
NPC − KLHL21
Main p=9.05e−05
Int p=0.000107
log2(RPKM+1)
2.8
2.6
2.4
2.2
2.0
1.8
1.6
1:CT
1:SZ
2:CT
2:SZ
3:CT
3:SZ
4:CT
4:SZ
NPC RNA Fraction Quantile & Dx

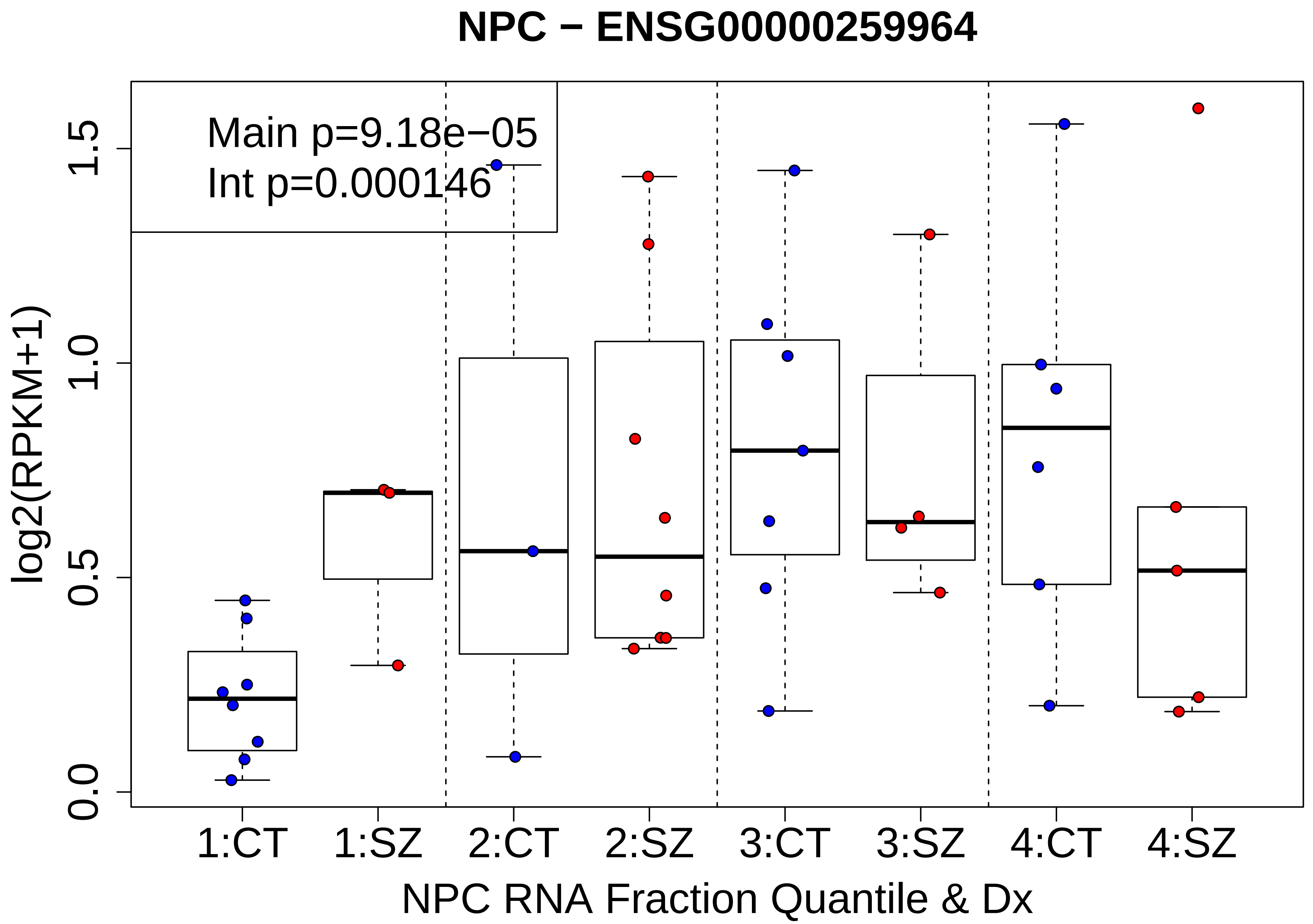
NPC − ENSG00000259964
Main p=9.18e−05
Int p=0.000146
log2(RPKM+1)
0.0
0.5
1.0
1.5
1:CT
1:SZ
2:CT
2:SZ
3:CT
3:SZ
4:CT
4:SZ
NPC RNA Fraction Quantile & Dx

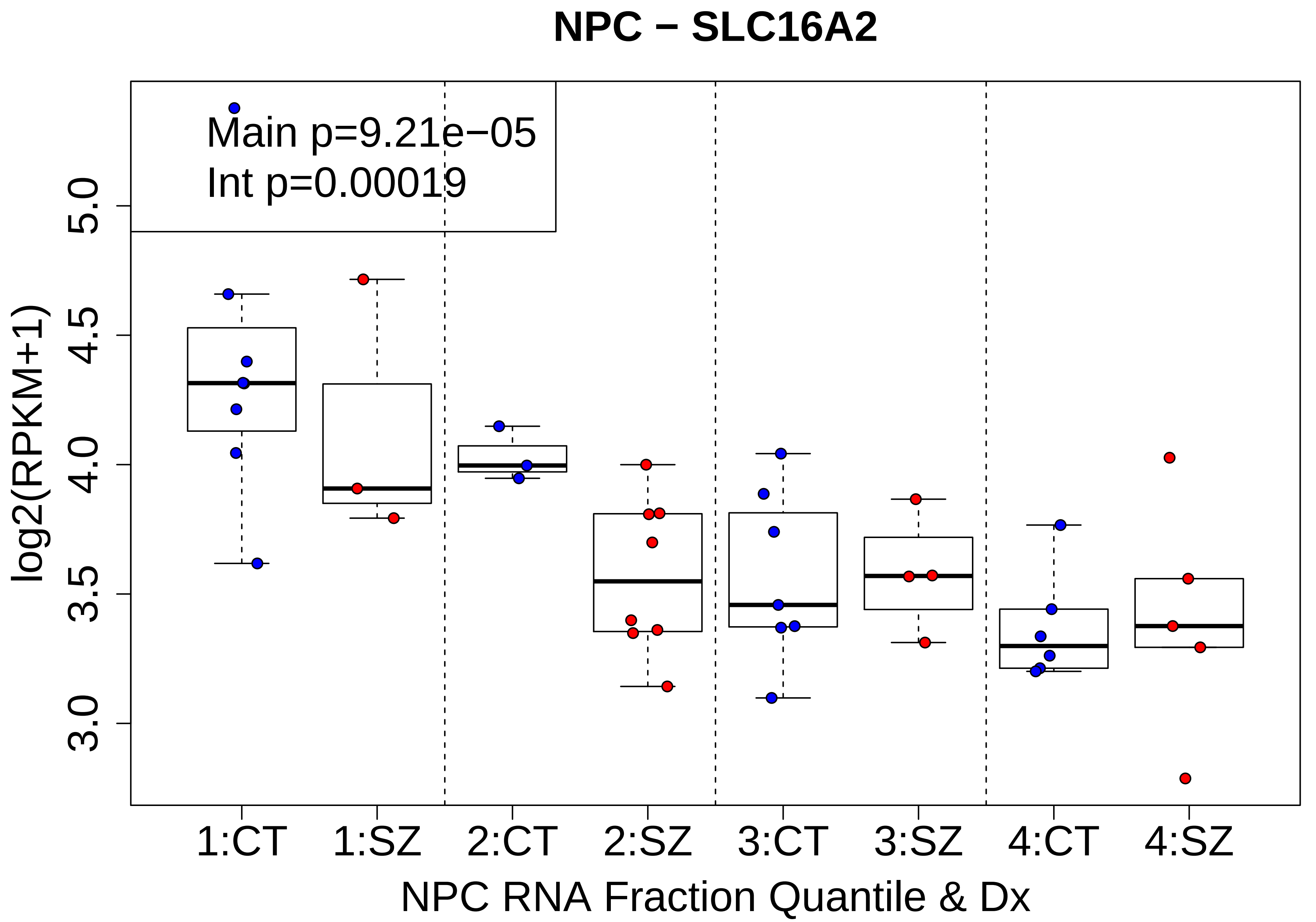

NPC − SLC16A2
Main p=9.21e−05
Int p=0.00019
log2(RPKM+1)
5.0
4.5
4.0
3.5
3.0
1:CT
1:SZ
2:CT
2:SZ
3:CT
3:SZ
4:CT
4:SZ
NPC RNA Fraction Quantile & Dx

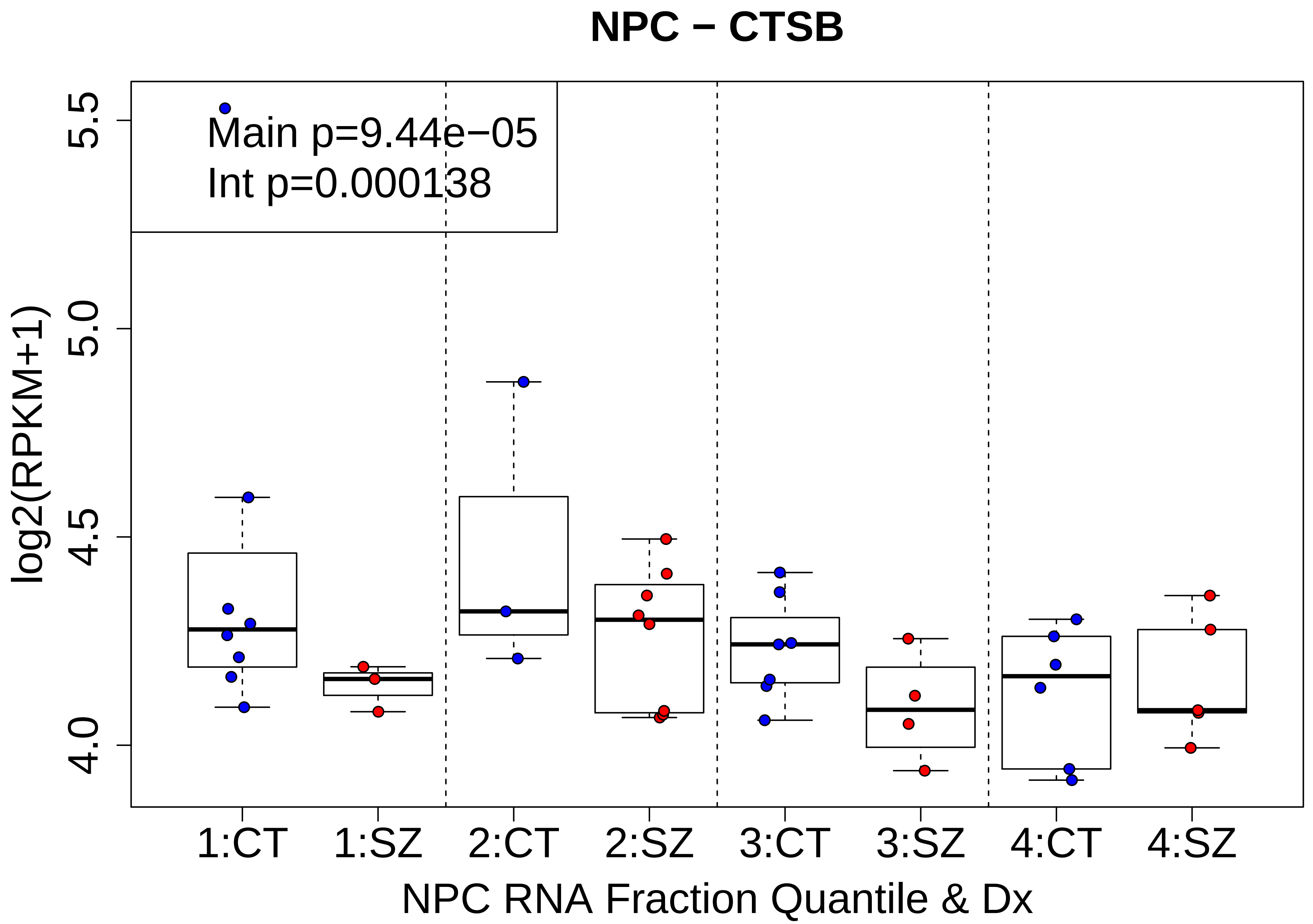
NPC − CTSB
Main p=9.44e−05
Int p=0.000138
log2(RPKM+1)
5.5
5.0
4.5
4.0
1:CT
1:SZ
2:CT
2:SZ
3:CT
3:SZ
4:CT
4:SZ
NPC RNA Fraction Quantile & Dx

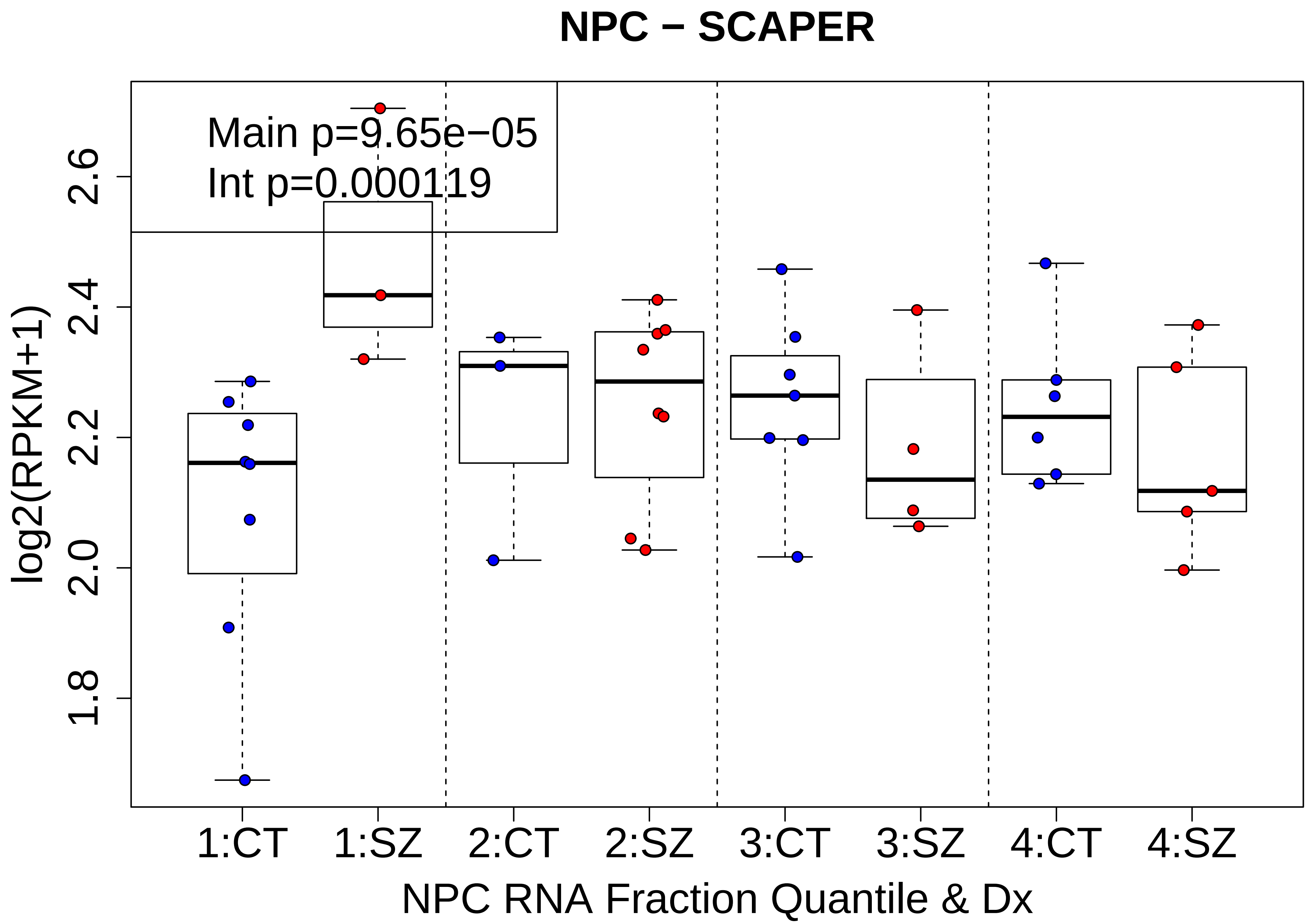
NPC − SCAPER
Main p=9.65e−05
Int p=0.000119
log2(RPKM+1)
1.8
2.0
2.2
2.4
2.6
1:CT
1:SZ
2:CT
2:SZ
3:CT
3:SZ
4:CT
4:SZ
NPC RNA Fraction Quantile & Dx

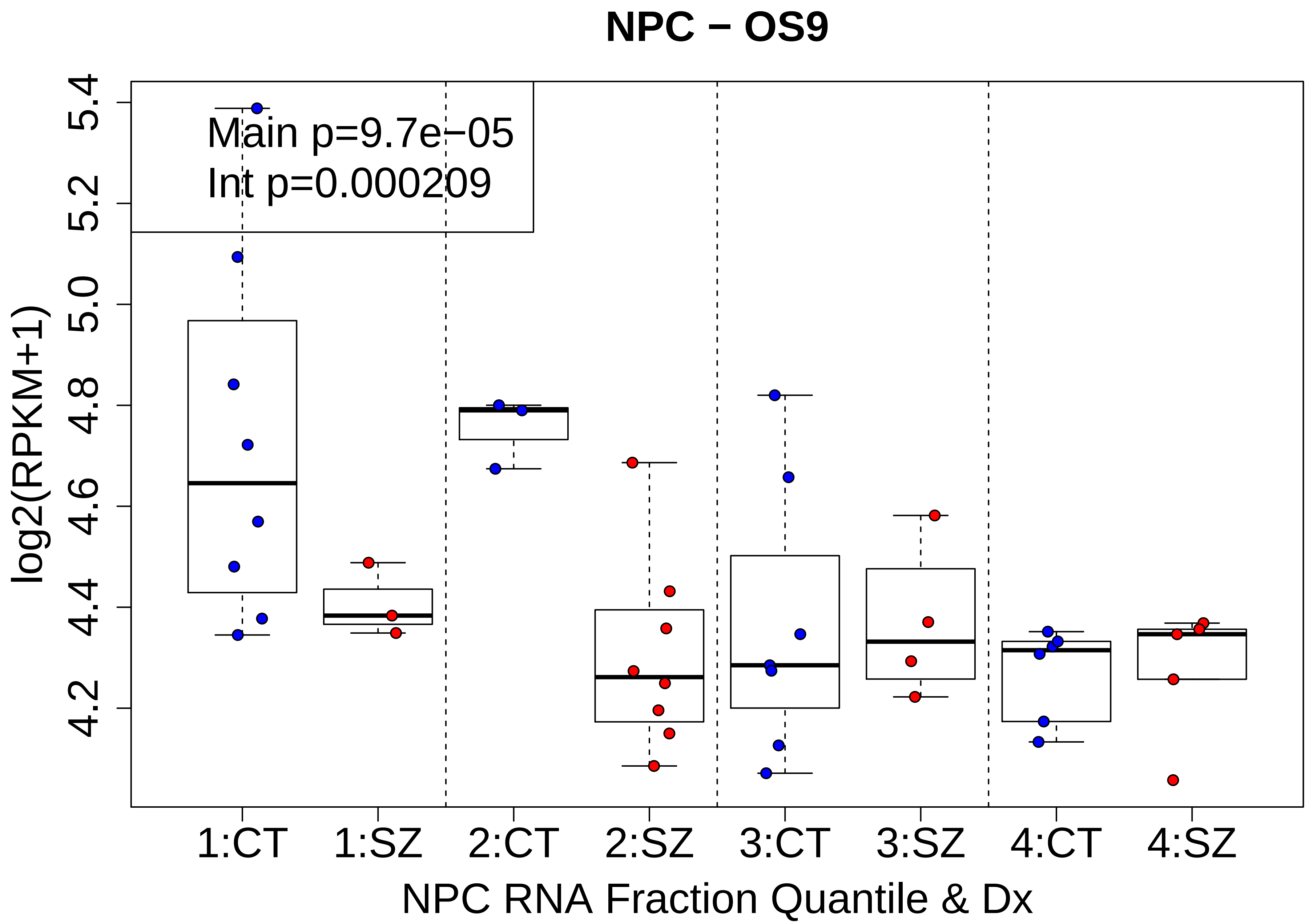
NPC − OS9
Main p=9.7e−05
Int p=0.000209
log2(RPKM+1)
5.4
5.2
5.0
4.8
4.6
4.4
4.2
1:CT
1:SZ
2:CT
2:SZ
3:CT
3:SZ
4:CT
4:SZ
NPC RNA Fraction Quantile & Dx

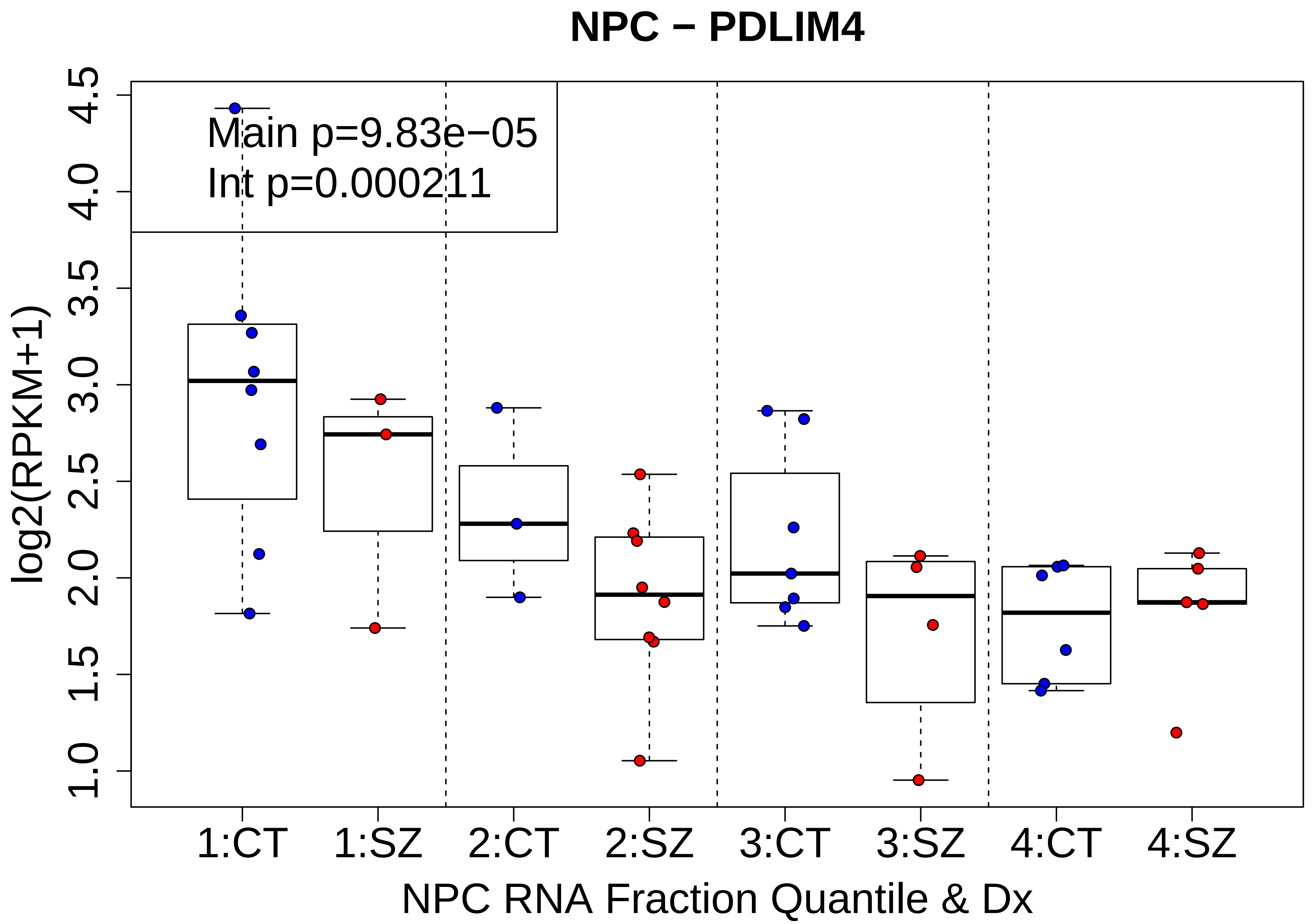
NPC − PDLIM4
Main p=9.83e−05
Int p=0.000211
log2(RPKM+1)
1.0
1.5
2.0
2.5
3.0
3.5
4.0
4.5
1:CT
1:SZ
2:CT
2:SZ
3:CT
3:SZ
4:CT
4:SZ
NPC RNA Fraction Quantile & Dx

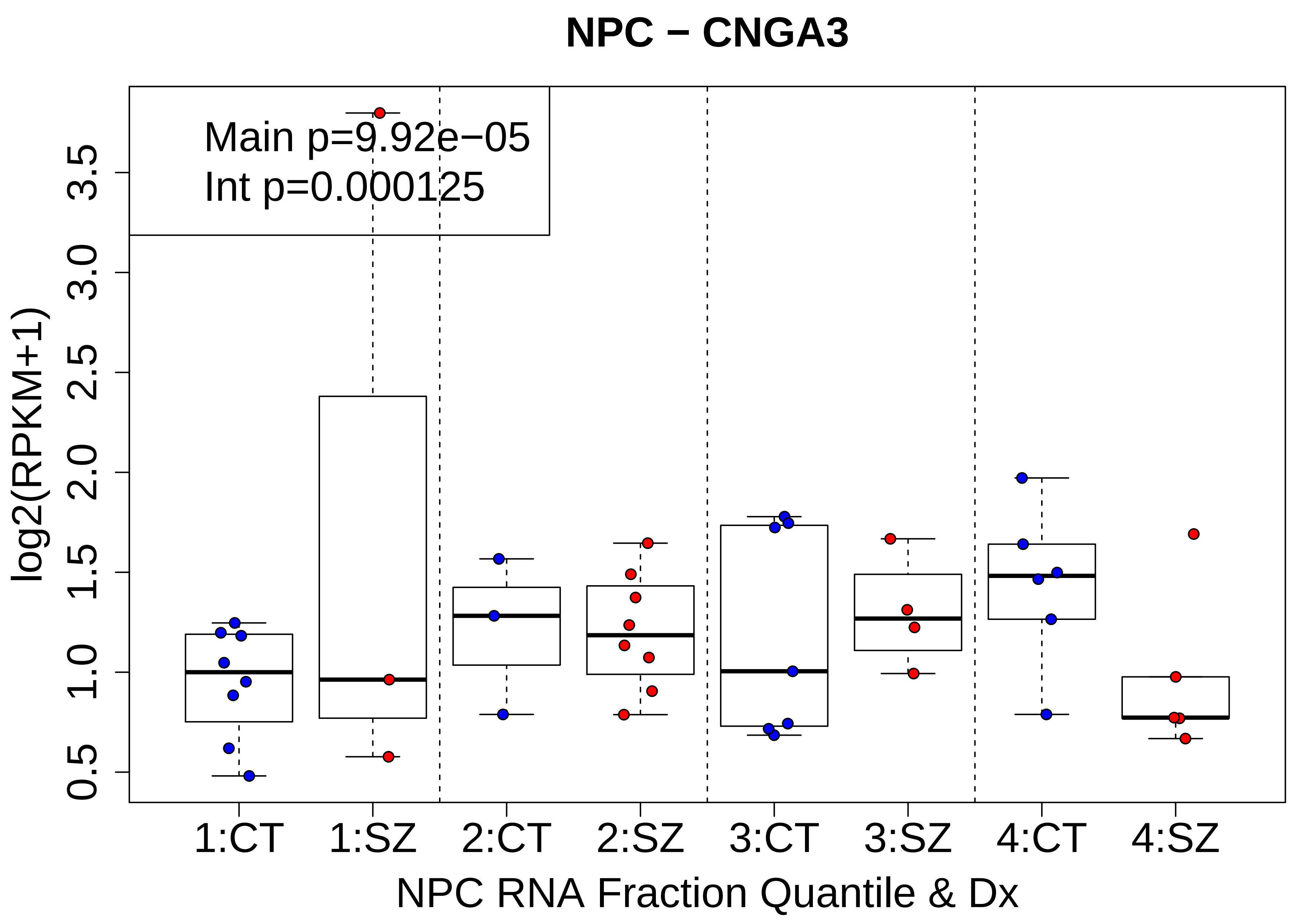

NPC − CNGA3
Main p=9.92e−05
Int p=0.000125
log2(RPKM+1)
0.5
1.0
1.5
2.0
2.5
3.0
3.5
1:CT
1:SZ
2:CT
2:SZ
3:CT
3:SZ
4:CT
4:SZ
NPC RNA Fraction Quantile & Dx

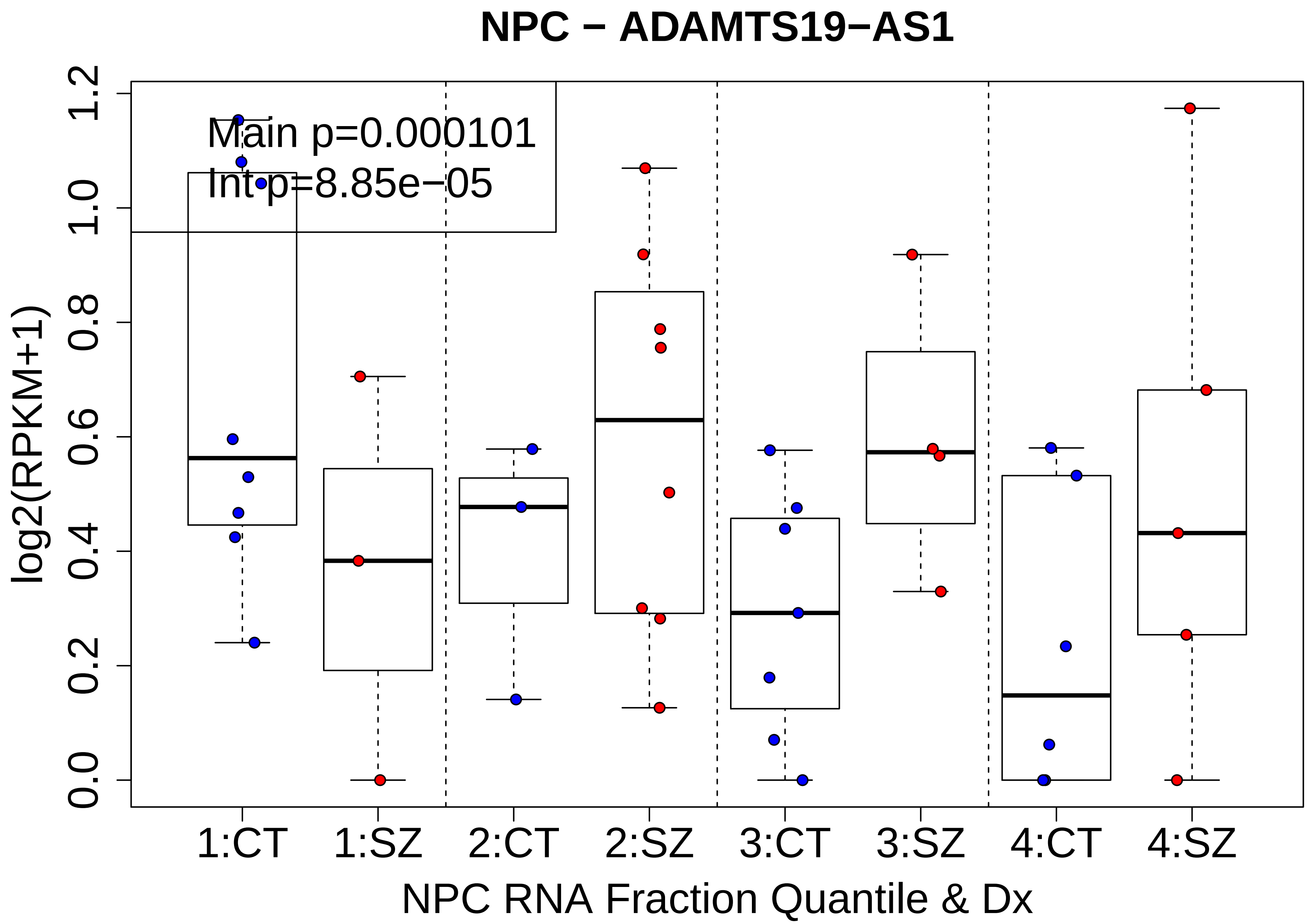
NPC − ADAMTS19−AS1
Main p=0.000101
Int p=8.85e−05
log2(RPKM+1)
0.0
0.2
0.4
0.6
0.8
1.0
1.2
1:CT
1:SZ
2:CT
2:SZ
3:CT
3:SZ
4:CT
4:SZ
NPC RNA Fraction Quantile & Dx

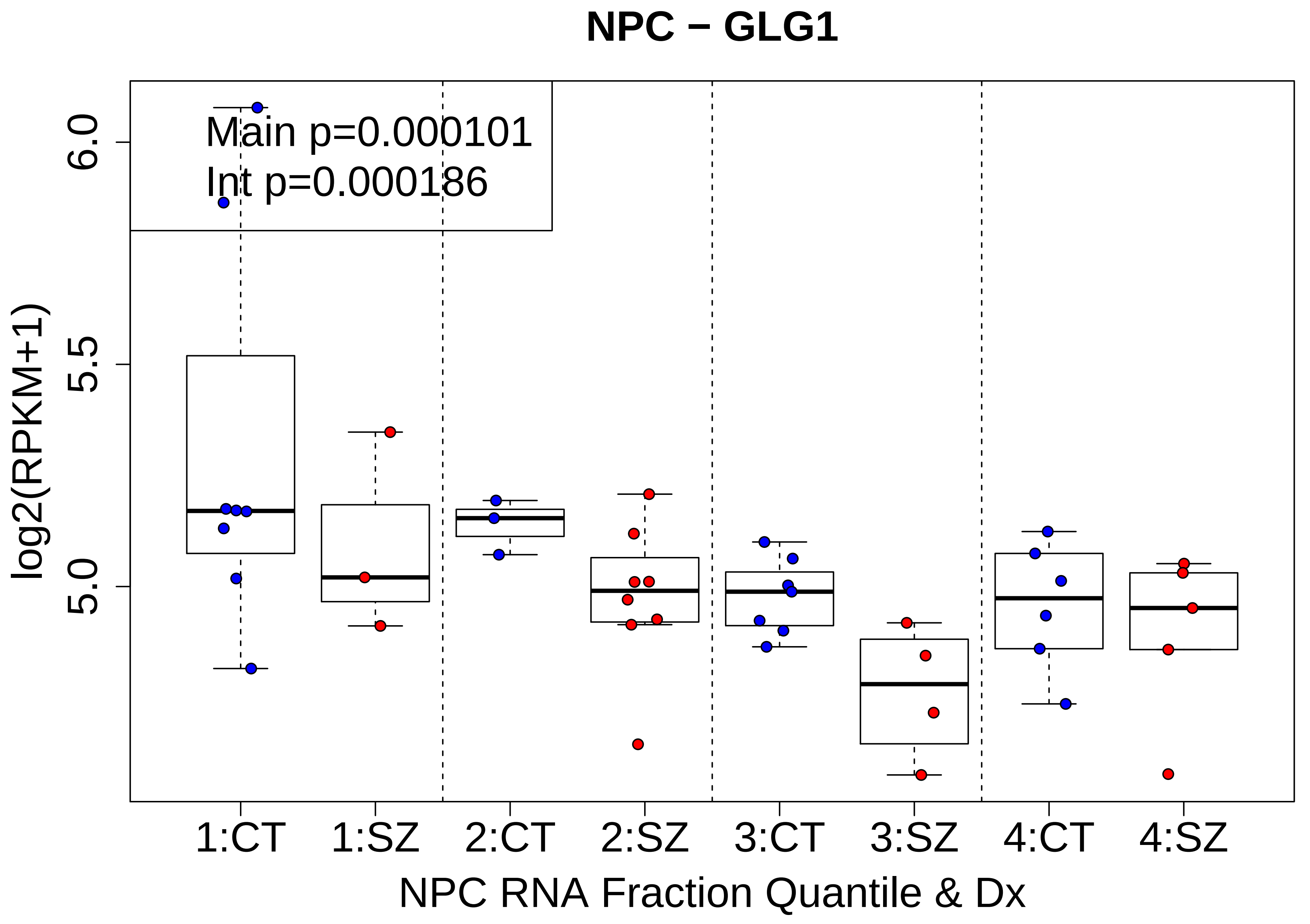
NPC − GLG1
Main p=0.000101
Int p=0.000186
log2(RPKM+1)
6.0
5.5
5.0
1:CT
1:SZ
2:CT
2:SZ
3:CT
3:SZ
4:CT
4:SZ
NPC RNA Fraction Quantile & Dx

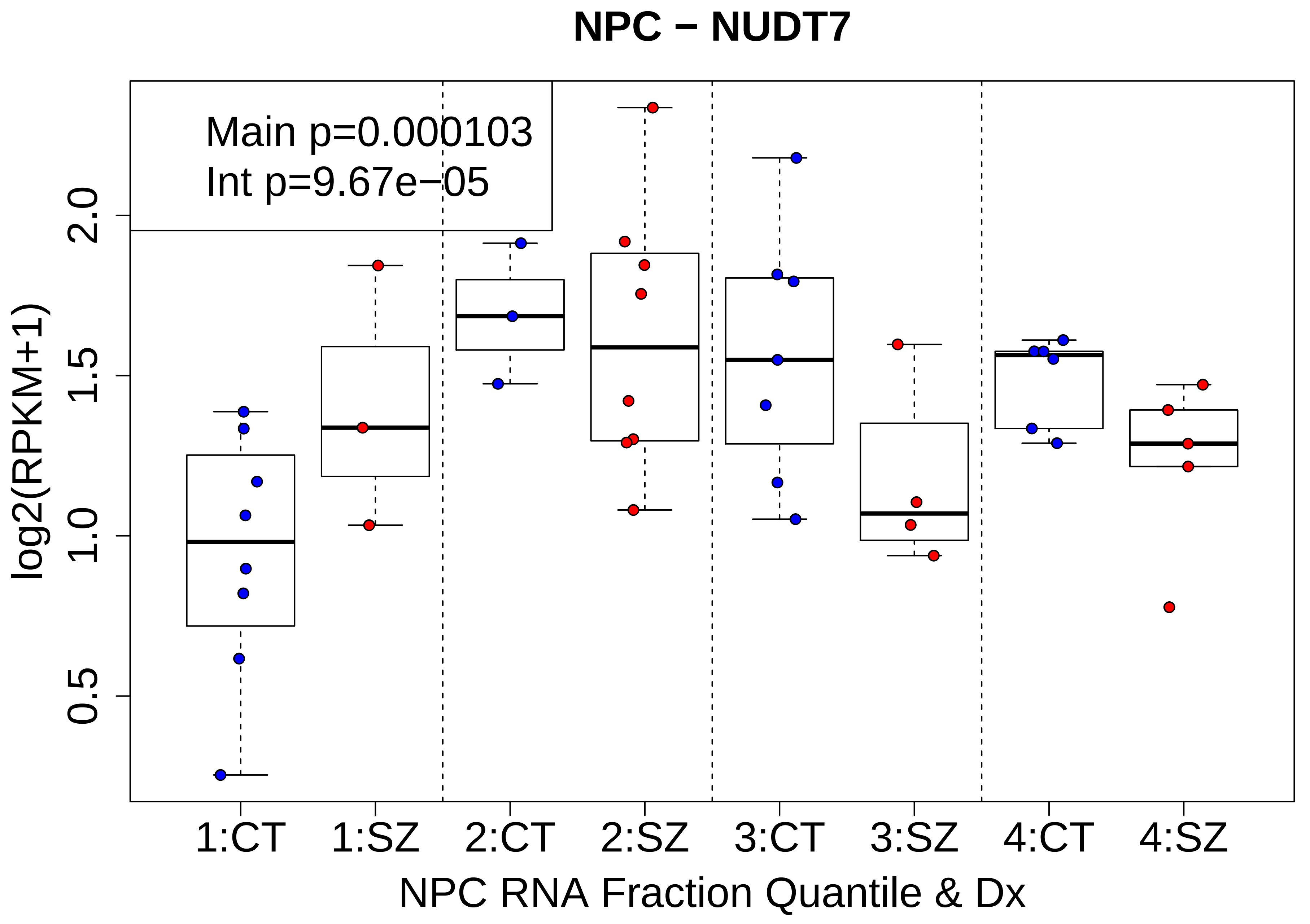
NPC − NUDT7
Main p=0.000103
Int p=9.67e−05
log2(RPKM+1)
0.5
1.0
1.5
2.0
1:CT
1:SZ
2:CT
2:SZ
3:CT
3:SZ
4:CT
4:SZ
NPC RNA Fraction Quantile & Dx

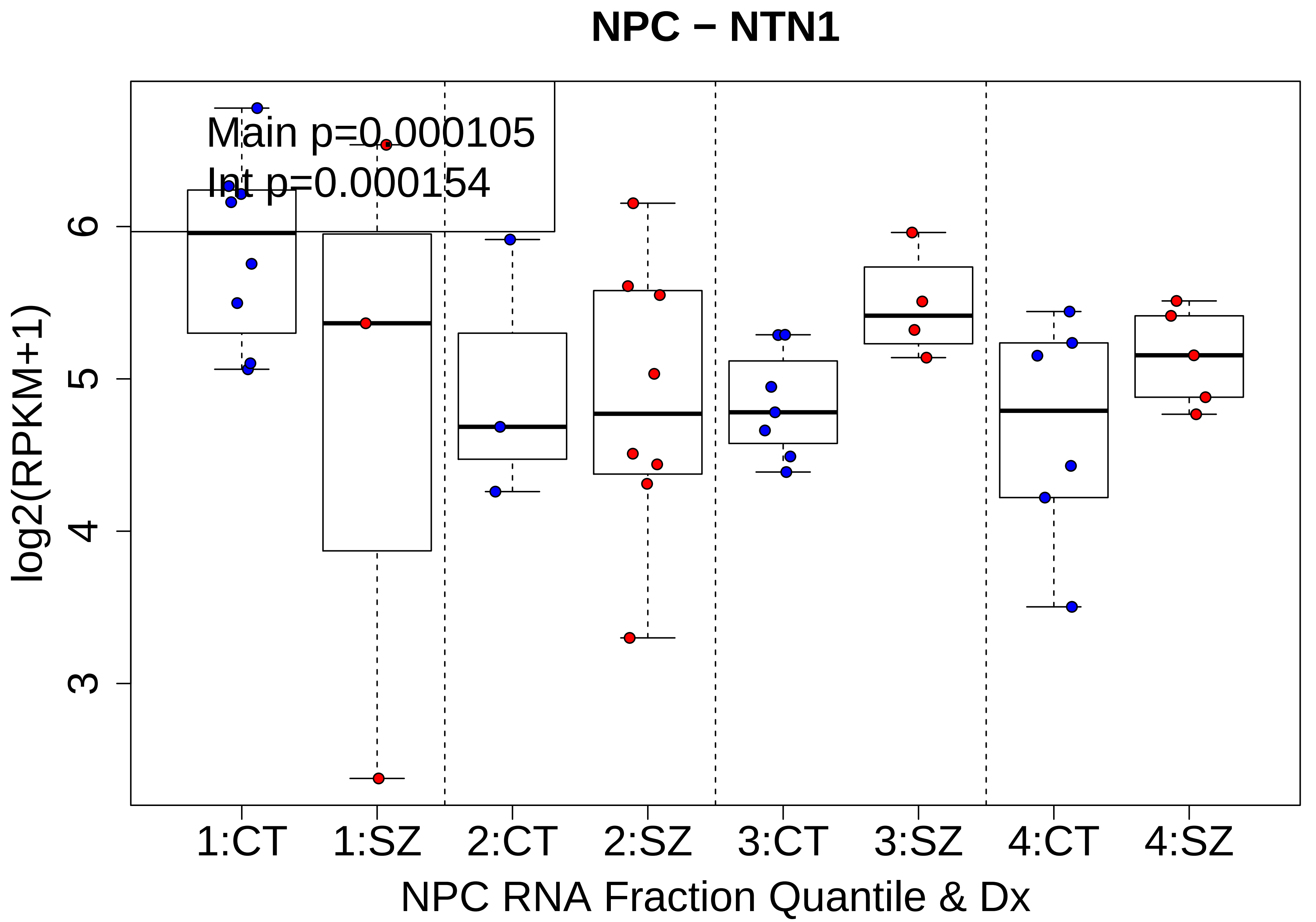

NPC − NTN1
Main p=0.000105
Int p=0.000154
log2(RPKM+1)
3
4
5
6
1:CT
1:SZ
2:CT
2:SZ
3:CT
3:SZ
4:CT
4:SZ
NPC RNA Fraction Quantile & Dx

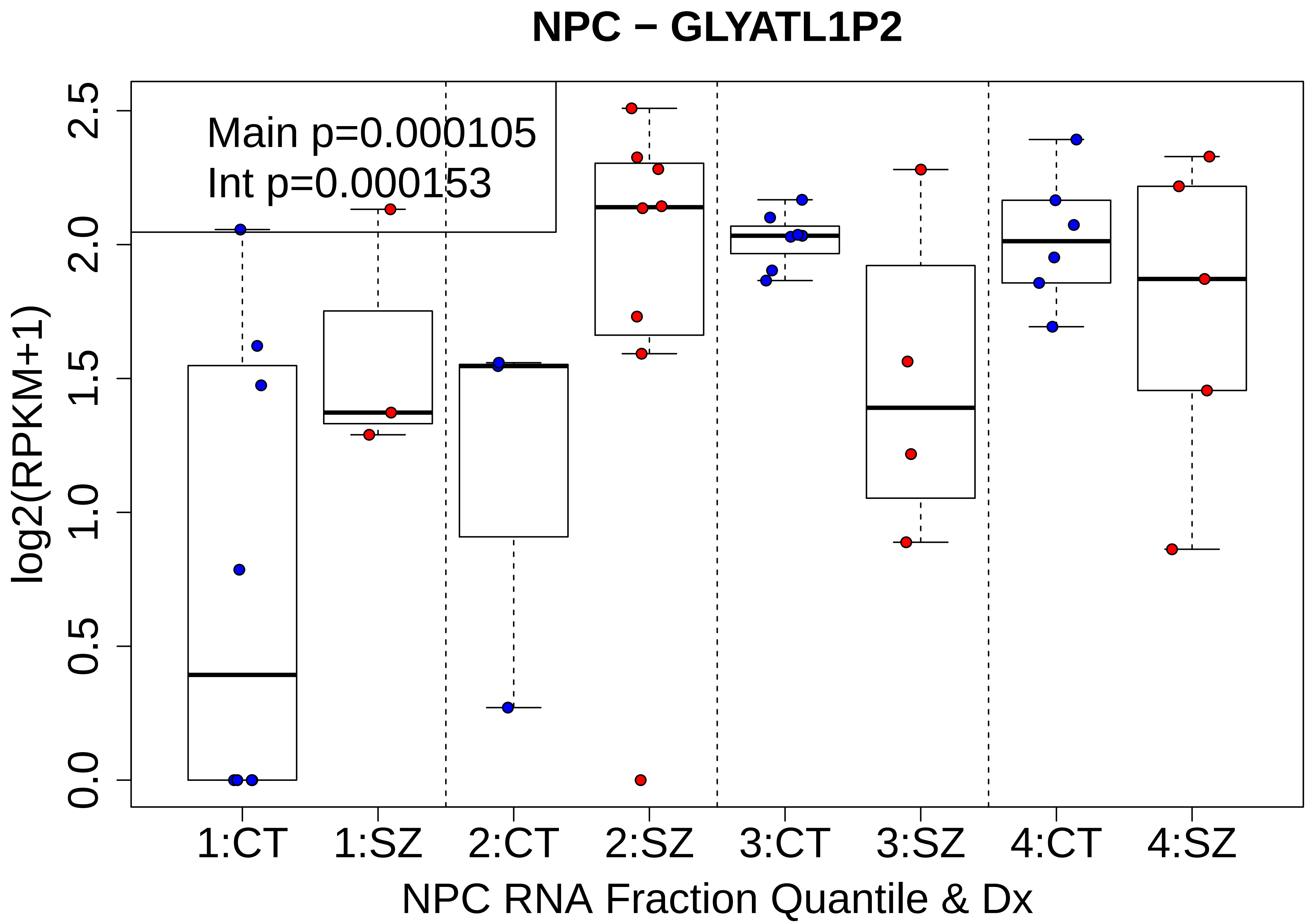

NPC − GLYATL1P2
Main p=0.000105
Int p=0.000153
log2(RPKM+1)
0.0
0.5
1.0
1.5
2.0
2.5
1:CT
1:SZ
2:CT
2:SZ
3:CT
3:SZ
4:CT
4:SZ
NPC RNA Fraction Quantile & Dx

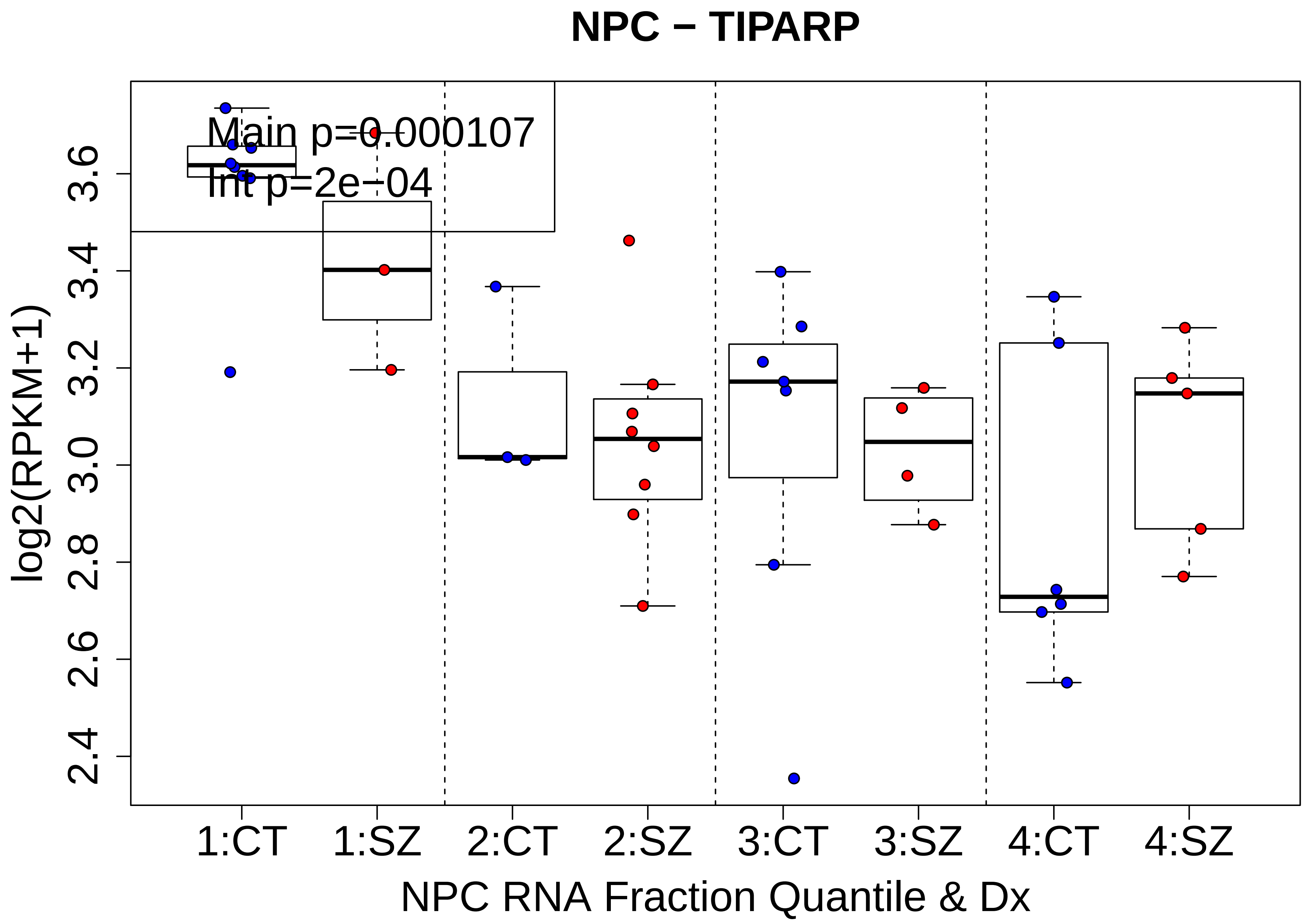
NPC − TIPARP
Main p=0.000107
Int p=2e−04
log2(RPKM+1)
3.6
3.4
3.2
3.0
2.8
2.6
2.4
1:CT
1:SZ
2:CT
2:SZ
3:CT
3:SZ
4:CT
4:SZ
NPC RNA Fraction Quantile & Dx

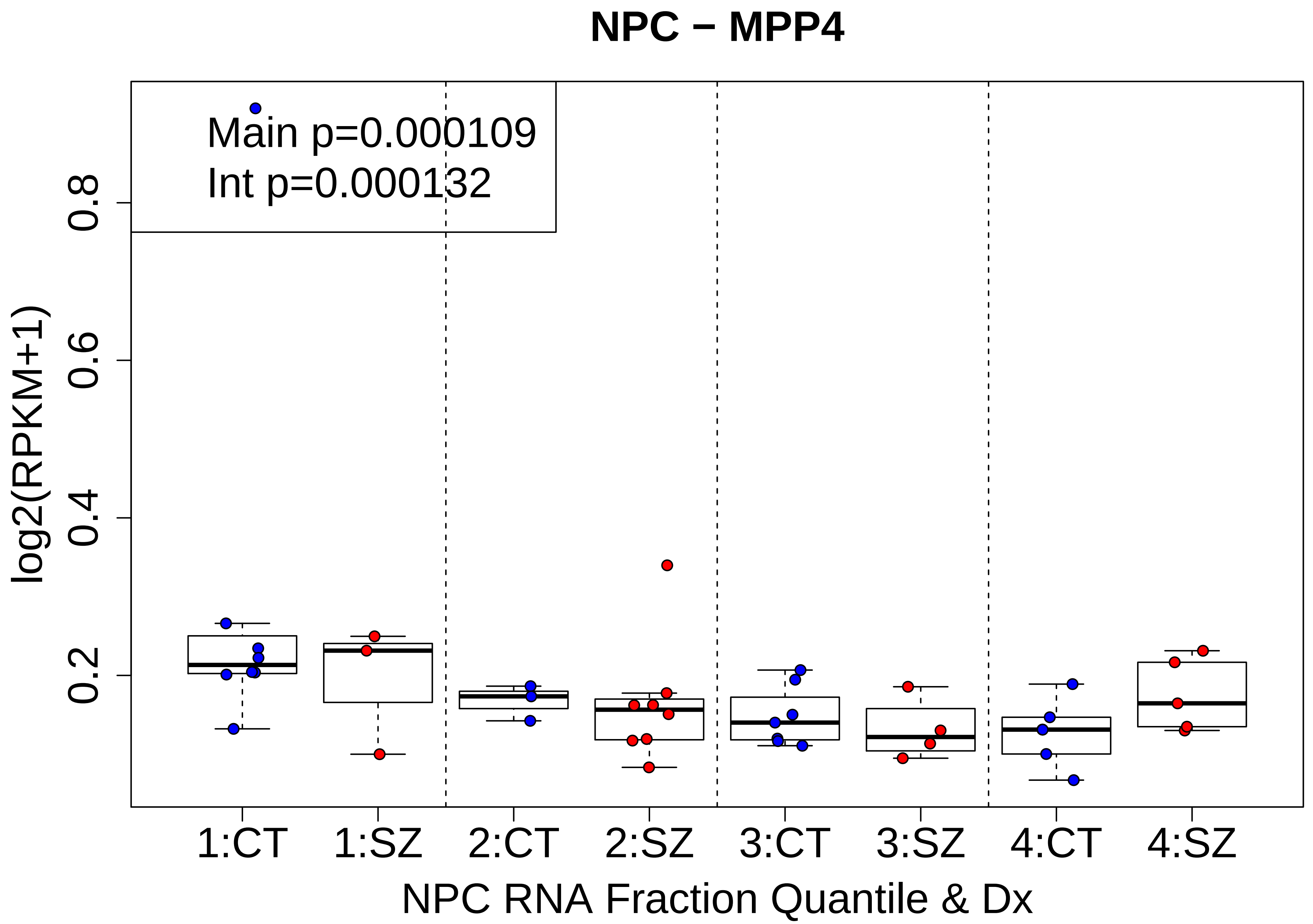

NPC − MPP4
Main p=0.000109
Int p=0.000132
log2(RPKM+1)
0.2
0.4
0.6
0.8
1:CT
1:SZ
2:CT
2:SZ
3:CT
3:SZ
4:CT
4:SZ
NPC RNA Fraction Quantile & Dx

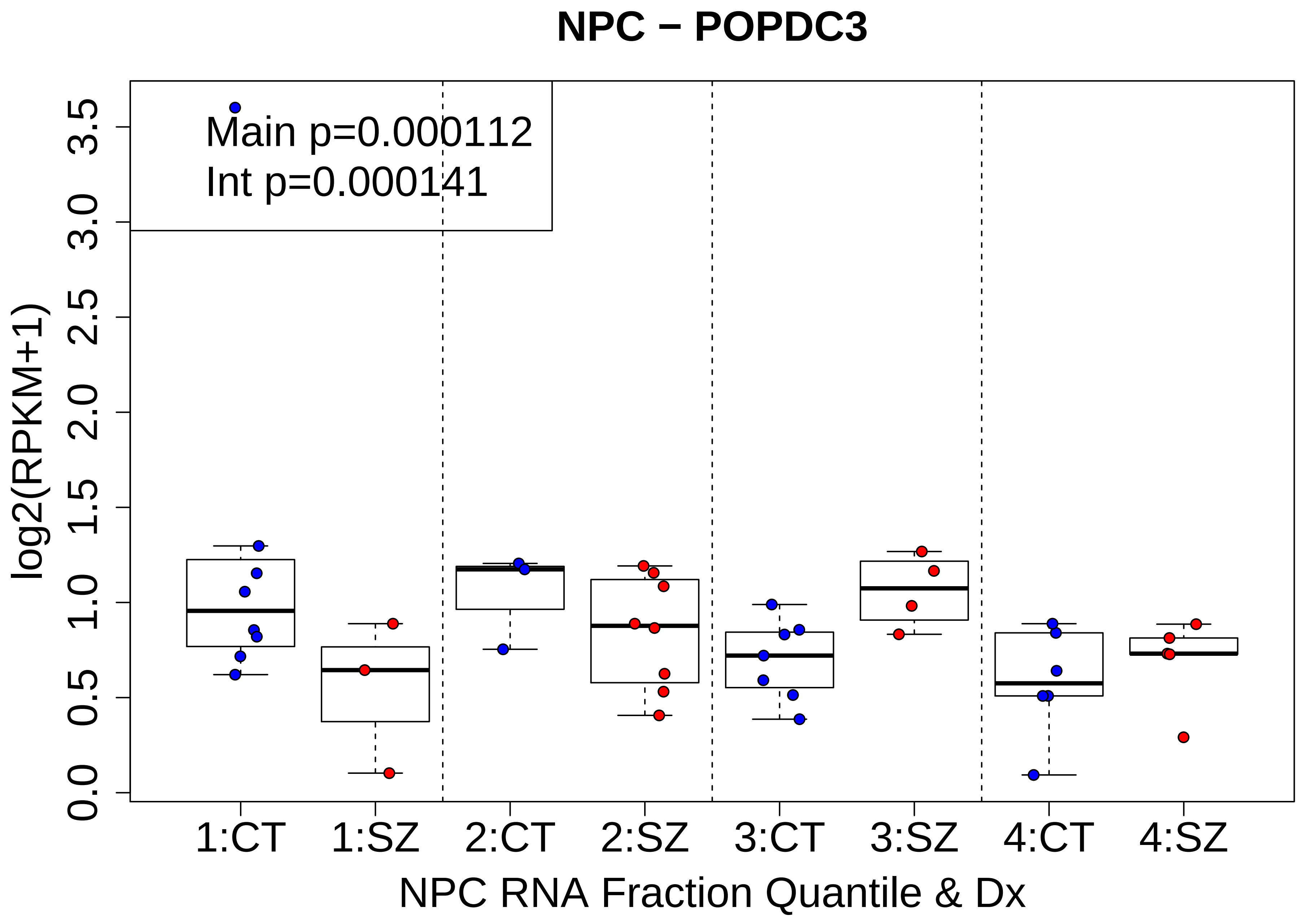

NPC − POPDC3
Main p=0.000112
Int p=0.000141
log2(RPKM+1)
0.0
0.5
1.0
1.5
2.0
2.5
3.0
3.5
1:CT
1:SZ
2:CT
2:SZ
3:CT
3:SZ
4:CT
4:SZ
NPC RNA Fraction Quantile & Dx

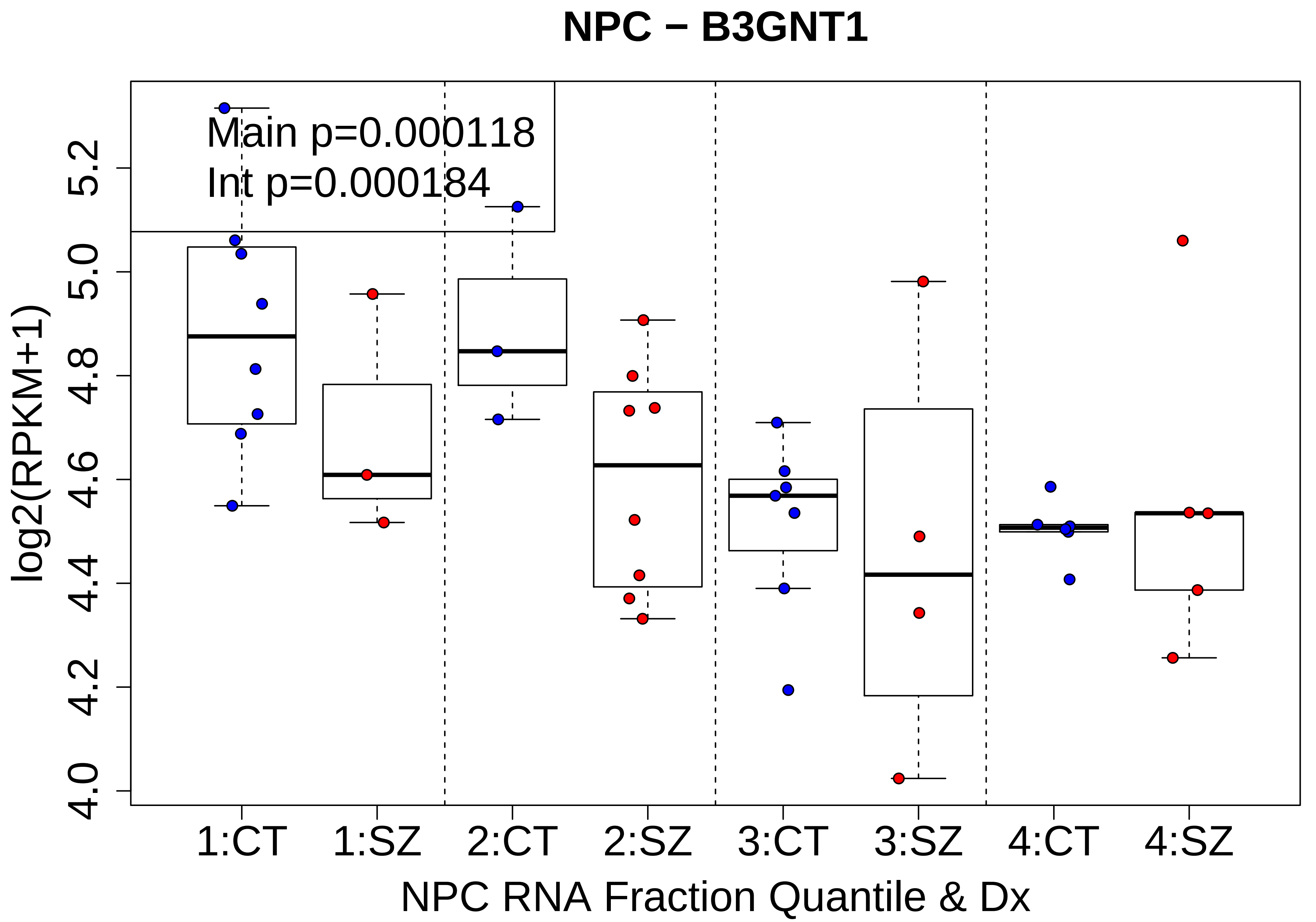
NPC – B3GNT1
Main p=0.000118
Int p=0.000184
log2(RPKM+1)
4.0
4.2
4.4
4.6
4.8
5.0
5.2
1:CT
1:SZ
2:CT
2:SZ
3:CT
3:SZ
4:CT
4:SZ
NPC RNA Fraction Quantile & Dx

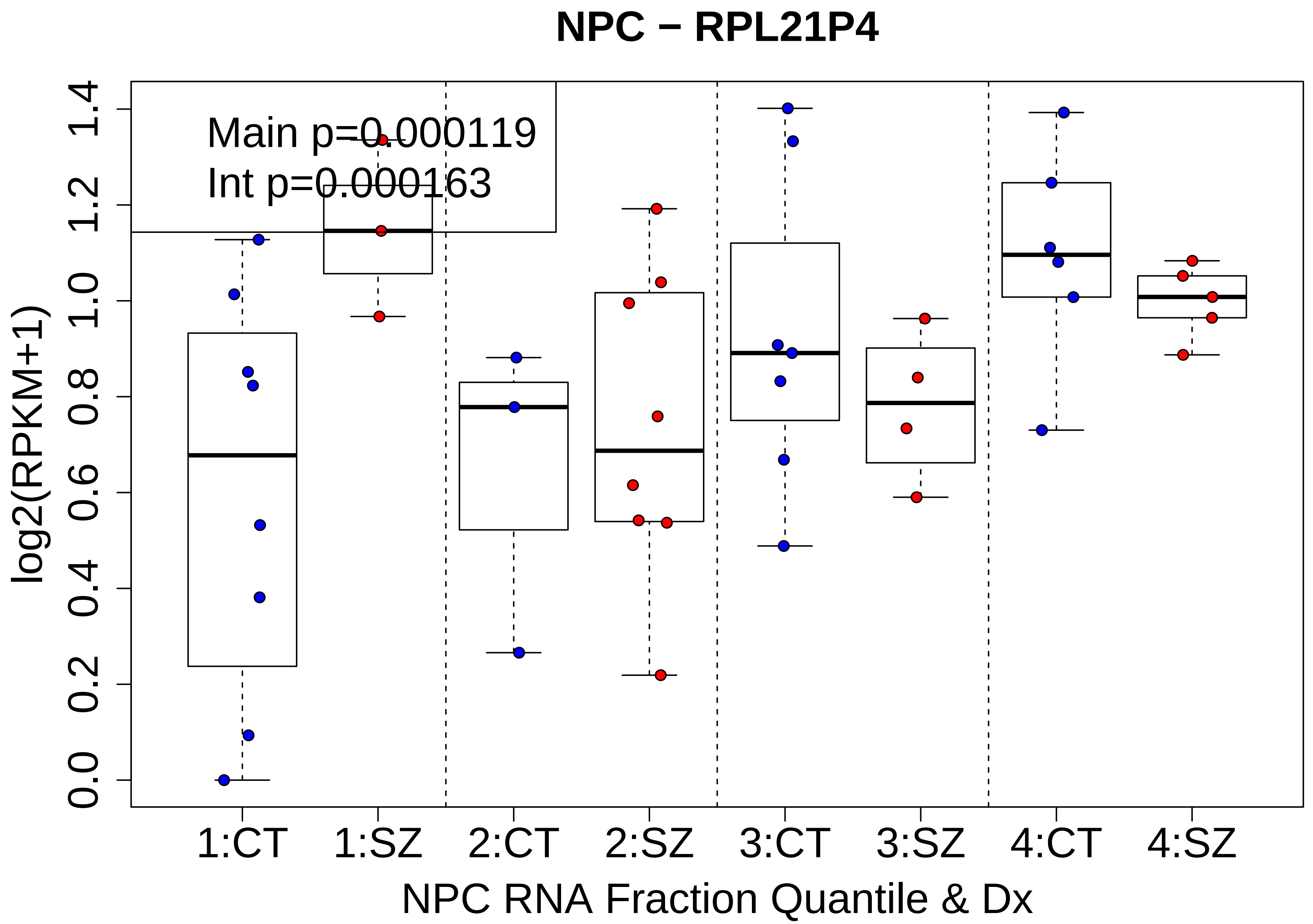
NPC − RPL21P4
Main p=0.000119
Int p=0.000163
log2(RPKM+1)
0.0
0.2
0.4
0.6
0.8
1.0
1.2
1.4
1:CT
1:SZ
2:CT
2:SZ
3:CT
3:SZ
4:CT
4:SZ
NPC RNA Fraction Quantile & Dx
